# Straightforward construction of functionalized *γ*-lactams via conjugated-engineered covalent organic framework photocatalysed cascade reactions

**DOI:** 10.1038/s41467-025-66469-2

**Published:** 2025-12-01

**Authors:** Xiangfeng Lin, Jianguo Li, Jiaxian Zheng, Xiaowei Cai, Liwei Wang, Rongjian Sa, Chuanling Si, Dong Jiang, Yunqing Kang, Jie Wang, Yusuke Yamauchi, Zhanhui Yuan

**Affiliations:** 1 College of Materials Engineering, Fujian Agriculture and Forestry University, Fuzhou, P. R. China; 2 College of Materials and Chemical Engineering, Minjiang University, Fuzhou, P. R. China; 3 State Key Laboratory of Biobased Fiber Materials, Tianjin Key Laboratory of Pulp and Paper, Tianjin University of Science and Technology, Tianjin, P. R. China; 4 Department of Materials Process Engineering, Graduate School of Engineering, Nagoya University, Nagoya, Japan; 5 Australian Institute for Bioengineering and Nanotechnology (AIBN), The University of Queensland, Brisbane, QLD Australia; 6 Department of Chemical and Biomolecular Engineering, Yonsei University, Seoul, South Korea

**Keywords:** Organic molecules in materials science, Heterogeneous catalysis, Polymers

## Abstract

Straightforward synthetic approach for direct constructing functionalized *γ*-lactams is highly valuable given their ubiquity in bioactive molecules yet challenging due to instability of the products and difficulties in controlling diastereoselectivity. We herein report the design and synthesis of a naphthyl-based pyrene-containing covalent organic framework and utilize it as heterogeneous photocatalyst in visible-light catalytic cascade reactions between tryptamine-derived isocyanides and phosphine oxides. A broad range of 3-(2-aminophenyl)-*γ*-lactams are isolated in good yields under mild conditions and 3-(2-isocyanobenzyl)-indoles are also tolerated, giving (2-aminophenyl)-tetrahydroquinolines in moderate yield. Experimental studies and theoretical calculations reveal that a proton-coupled electron transfer process occurs between the excited covalent organic framework and phosphine oxide, which subsequently triggers a series of cascade reactions involving cyclization, semipinacol rearrangement, and dehydrogenation/hydration processes. Notably, the in situ generated spiroindolenine is identified to be the key intermediate. Illustrated by the efficient synthesis of 3-(2-aminophenyl)-*γ*-lactams, this work sets a precedent for the development of heterogeneous photocatalytic strategies for constructing complex bioactive molecules.

## Abstract (web summary)

The synthesis of functionalized γ-lactams is challenging due to instability of the products and difficulties in controlling diastereoselectivity. Here, the authors report on the design and synthesis of a naphthyl-based pyrene-containing covalent organic framework and utilize it as a heterogeneous photocatalyst for synthesis of γ-lactams.

## Introduction

**Fig. 1:**
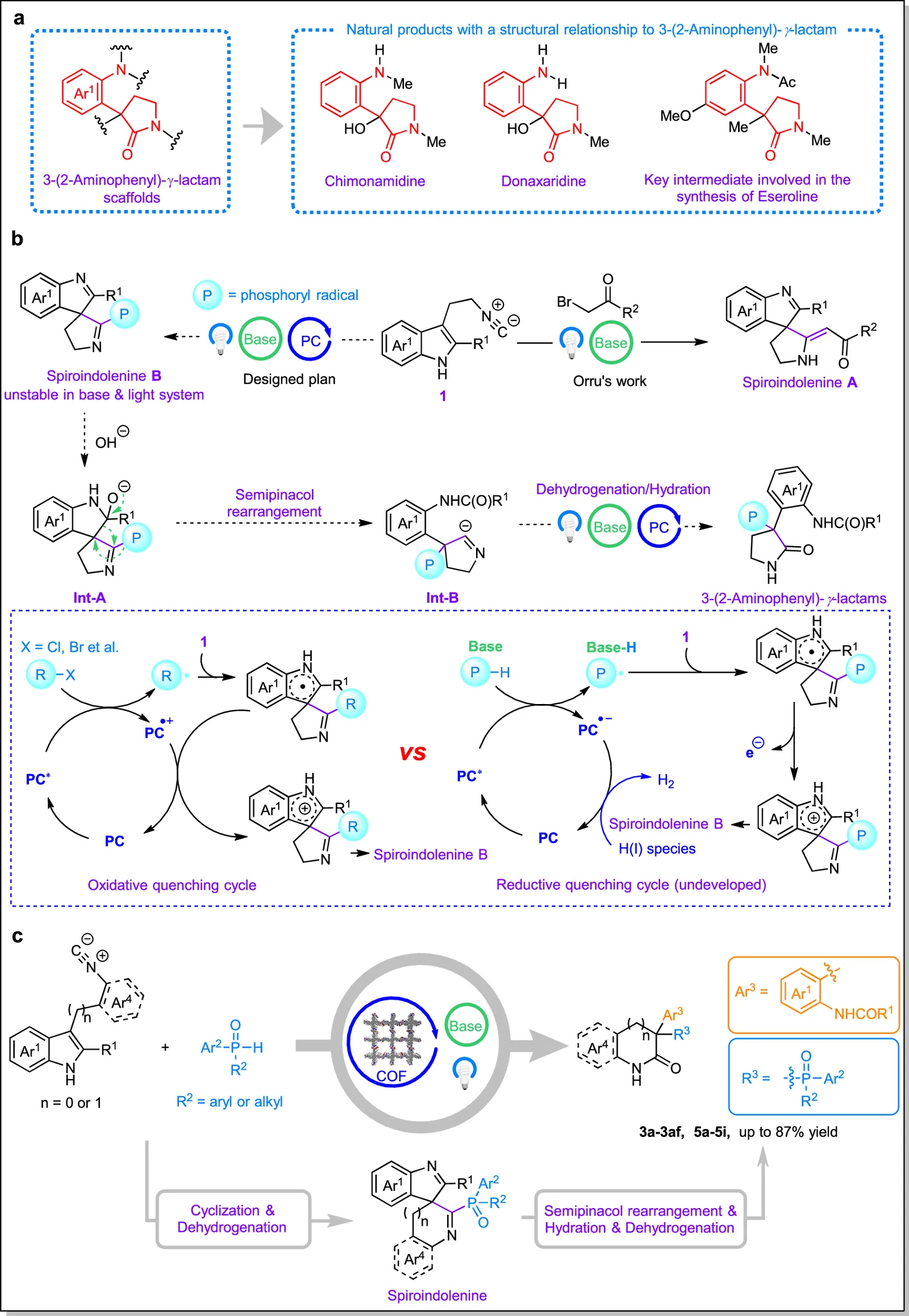
The synthesis of 3-(2-aminophenyl)-*γ*-lactams via conjugated-engineered covalent organic framework photocatalysed cascade reactions. **a** 3-(2-Aminophenyl)-*γ*-lactams and several relevant natural products. **b** The synthesis of spiroindolenines via photoinduced cyclizations and designed plan involving the synthesis of 3-(2-aminophenyl)-*γ*-lactams via photocatalytic cascade reactions. **c** This work: The synthesis of 3-(2-aminophenyl)-*γ*-lactams and derivatives via NPy-DMTP-COF photocatalytic cascade reactions.

Cyclic amides are core pharmacophores found across natural products and clinically used drugs. *β*-lactams anchor the antibiotic arsenal^1,2^, whereas the five-membered *γ*-lactam ring continues to serve as the critical scaffold in marketed agents for cancer, depression, and other diseases^3–16^. As an essential structural framework of functionalized *γ*-lactams, the 3-(2-aminophenyl)-*γ*-lactams are known for their pronounced biological activity (Fig. 1a)^17–19^. Several examples involving the synthesis of *α*-OH substituted (2-aminophenyl)-*γ*-lactams such as chimonamidine and donaxaridine, have been developed through a multi-step functionalization process of 3-hydroxy-2-oxindoles^20–24^. Ren et al. developed a copper/iodine co-catalyzed oxygenation transannulation, providing direct access to the donaxaridine and its derivatives^25^. Owing to the intrinsic lability of the target intermediates, extra functional groups must be pre-embedded in the substrates, a requirement that inevitably compromises both atom economy and overall cost efficiency^21^. Therefore, the development of a sustainable and straightforward synthetic approach for synthesizing this subclass of functionalized *γ*-lactams from widely available starting materials would provide significant insights and inspiration in this domain.

Our initial proposal for constructing 3-(2-aminophenyl)-*γ*-lactams was transformation from spiroindolenines, which can be formed via a photocatalytic cascade reaction between tryptamine-derived isocyanide and nucleophilic radical via dearomatization reactions^26–34^. Tryptamine-derived isocyanides, featured an indole core with an isocyanide chain at the C3 position, have exhibited exceptional efficacy in various chemical reactions. Diverging from conventional catalytic methods, Orru et al. innovated a catalyst-free dearomatization under visible light that yields a series of spiroindolenines **A** (Fig. 1b, black solid arrow)^35^. The crux of this process is primarily attributed to the formation of an imidoyl radical intermediate, arising from the interaction between phenacyl radical and tryptamine-derived isocyanide. Despite these advances, the development of a photocatalytic cascade reaction between tryptamine-derived isocyanide and radical precursor initiated by a single electron transfer (SET) oxidation or PCET process involving reductive quenching cycle, such as the reaction with in-situ generated phosphoryl radical from phosphine oxides, remains a significant challenge (Fig. 1b, blue dashed-line box). Notably, Yu et al. have developed a photocatalytic synthesis of phosphorylated *N*-heteroarenes from aryl isocyanides and phosphine oxides via a 4CzIPN-catalyzed PCET mechanism^36^. Wu et al. achieved similar outcomes using cobaloxime photocatalysis with H_2_ as byproduct^37^. Building on existing examples of photocatalytic cascade reactions involving aryl isocyanides^38–44^, we envision that spiroindolenine **B** could be constructed via a photocatalytic cascade reaction between the tryptamine-derived isocyanide and phosphine oxide (Fig. 1b, black dashed arrow). Subsequently, **Int-B** could be generated through hydration of spiroindolenine **B**, followed by a semipinacol rearrangement, which would be a powerful method for constructing ketones with quaternary centers and can occur in both acidic^45–62^ and basic^63^ environments. Ultimately, the **Int-B** would undergo a dehydrogenation/hydration process, forming the (2-aminophenyl)-*γ*-lactams. Realizing the above transformation critically depends on the development of an efficient photocatalyst.

Covalent organic frameworks (COFs) are porous crystalline materials composed of organic building blocks, known for their tunable porosity, extended π-conjugation, adjustable bandgaps, high surface areas and stability. These properties make COFs highly attractive for photocatalysis^64–75^. Recently, two-dimensional COFs (2D-COFs) have shown exceptional performance as heterogeneous photocatalysts, driving various chemical transformations under light irradiation^76–81^. Among them, benzyl-based pyrene-containing COFs (Py-COFs) synthesized by the condensation between 4,4’,4”,4”‘-(pyrene-1,3,6,8-tetrayl)tetraaniline (Py) and diarylglyoxal has demonstrated impressive photocatalytic activity in a range of organic reactions^82–87^. One of the key advantages of Py-COFs lies in their broad π-conjugated systems, which possess electron-withdrawing characteristics that readily enable the capture of electrons in PCET events. Moreover, the photocatalytic properties of pyrene-containing COFs can be precisely tuned by introducing aryl groups with diverse conjugated systems. In addition, Py-COFs have demonstrated remarkable efficacy as photocatalysts in reducing H(I) species to hydrogen gas^88–90^. It is believed that meticulously engineered pyrene-based COF catalysts, featuring optimized conjugation systems and pore structures, will significantly enhance the efficiency and sustainability of our designed cascade catalytic pathways.

Herein, we substituted the phenyl group with a naphthyl group at the 1,3,6,8 positions of pyrene and synthesized NPy-DMTP-COF. A series of heterogeneous photocatalytic cascade reactions between tryptamine-derived isocyanides and phosphine oxides mediated by NPy-DMTP-COF was established. This reaction affords a broad range of 3-(2-aminophenyl)-*γ*-lactams in moderate to high yields with excellent diastereoselectivities ( > 20: 1) (Fig. 1c). Specifically, 3-(2-isocyanobenzyl)-indoles are also tolerated, delivering a variety of (2-aminophenyl)-tetrahydroquinolines in moderate yield. Comprehensive mechanistic investigations indicate that the in-situ generated spiroindolenine serves as a pivotal intermediate in the cascade reaction, which encompasses cyclization, semipinacol rearrangement, hydration and dehydrogenation processes.

## Results and discussion

### Synthesis and characterizations of NPy-DMTP-COF

**Fig. 2:**
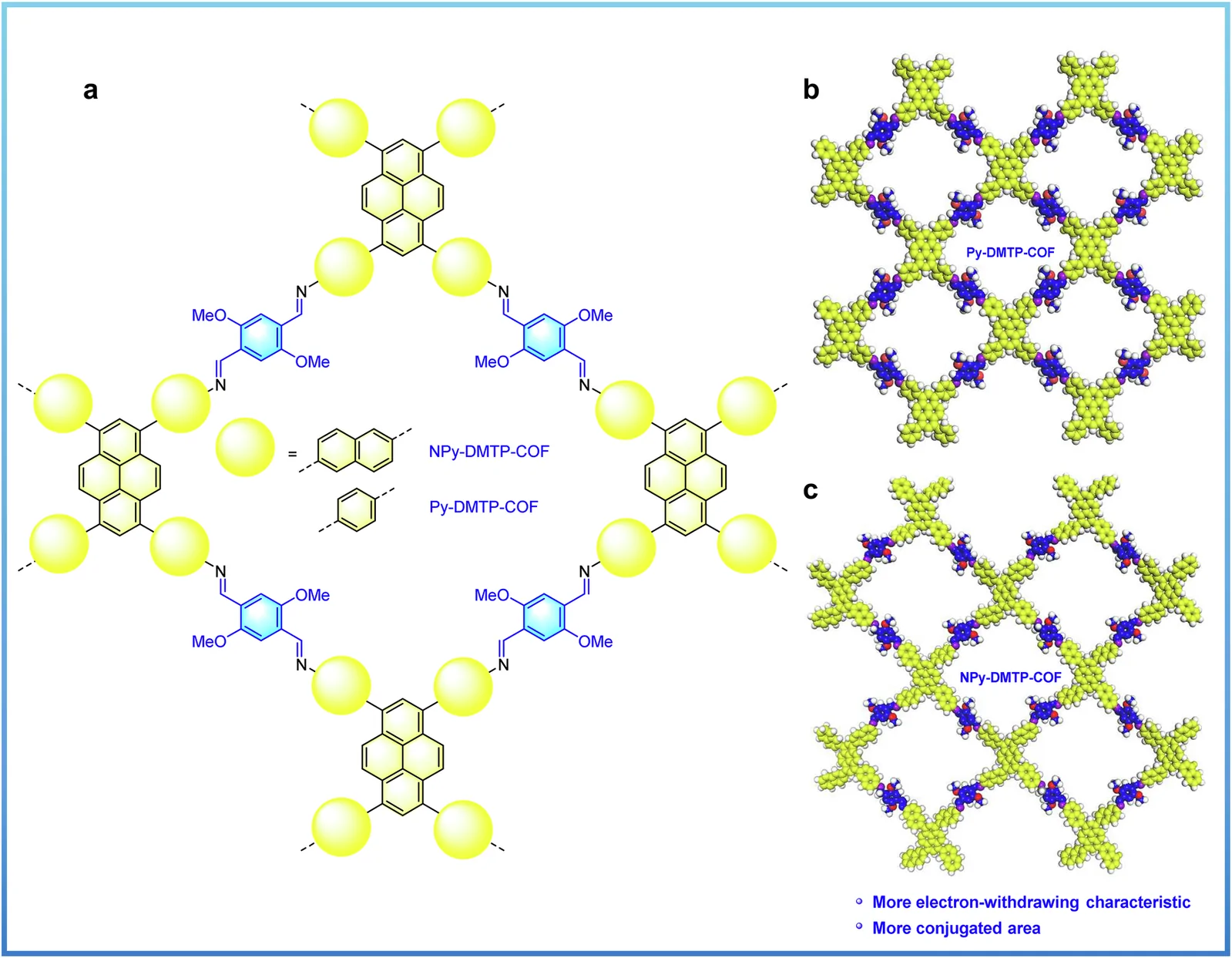
Structures of Py-DMTP-COF and NPy-DMTP-COF. **a** Chemical structures of Py-DMTP-COF and NPy-DMTP-COF. **b**, **c** Graphic view of one layer of Py-DMTP-COF and NPy-DMTP-COF simulated by Material Studio. (blue, yellow, C; purple, N; red, O; white, H).

Initially, NPy-DMTP-COF was synthesized via a condensation reaction between 1, 3, 6, 8-tetrakis(2-aminonaphthyl)pyrene (NPy) and 2, 5-dimethoxyterephthalaldehyde (DMTP) (Fig. 2a and Supplementary Fig. 1). Compared to the reported Py-DMTP-COF (Fig. 2b), NPy-DMTP-COF (Fig. 2c) exhibits stronger electron-withdrawing characteristics, suggesting a greater ability to accept electrons during the PCET process. Additionally, we envisage that its more extensively conjugated π-system would enhance visible-light absorption, making NPy-DMTP-COF a promising photocatalyst. The PXRD pattern of NPy-DMTP-COF exhibits four prominent diffraction peaks, with the most-intensive one at 3.28° and the three other peaks at 5.12°, 6.60°, and 23.64°. These peaks are assigned to the (100), (110), (220), and (001) facets, respectively (Supplementary Fig. 2a). The simulated PXRD pattern of the eclipsed AA stacking conducted by Materials Studio Software is in good agreement with the experimental PXRD patterns with a P1 space group. The Pawley refinement cell parameters are a = 26.9 Å, b = 3.8 Å, c = 27.0 Å, *α* = 90°, *β* = 90° and *γ* = 90°, with good agreement factors *R*_*wp*_ = 5.00% and *R*_*p*_ = 2.73% for NPy-DMTP-COF (Supplementary Table 1). The NPy-DMTP-COF shows remarkable chemical and structural stability, as almost identical PXRD patterns are obtained after treatment with 14 M NaOH, triethylamine, conc. HCl, trifluoroacetic acid and boiling water (Supplementary Fig. 2b). Scanning electron microscopy (SEM) images reveal that NPy-DMTP-COF adopts a uniform rod-like morphology with an average diameter of approximately 65 nm (Supplementary Fig. 3). High resolution transmission electron microscopy (HR-TEM) images show distinct ordered fringes, confirming its high crystallinity and revealing a quadrilateral pore structure with a periodicity of approximately 2.6 nm and particle size of around 60 nm (Supplementary Fig. 4). Dynamic light scattering (DLS) measurements, performed on NPy-DMTP-COF dispersed in acetonitrile at room temperature, reveal a Z-average size of 41.05 nm and a polydispersity index (PDI) of 0.115 (Supplementary Fig. 5). The formation of imine linkage of NPy-DMTP-COF was confirmed by Fourier transform infrared (FT-IR) spectra. The disappearance of the N−H vibration at 1630, 3460 and 3370 cm^-1^ and appearance of the C=N vibration at 1590 cm^-1^ confirm the high condensation degree of NPy-DMTP-COF (Supplementary Fig. 6). The chemical structure was further elucidated using solid-state ^13^C cross-polarization/total sideband suppression (^13^C-CP/TOSS) spectroscopy. Chemical shifts at 152.4 ppm assigned to ^13^C signal of C=N bond indicate the successful formation of NPy-DMTP-COF with the imine linkage (Supplementary Fig. 7). Nitrogen adsorption-desorption isotherms demonstrate that the NPy-DMTP-COF has a mesoporous structure with a BET surface area of 1334 m^2^/g, a pore volume of 1.27 cm^3^/g and a pore width of 2.35 nm (Supplementary Fig. 8 and Supplementary Fig. 9), matching well with the simulated values and HR-TEM results. Thermogravimetric analysis (TGA) shows that NPy-DMTP-COF remains stable up to a temperature of 350 °C under both nitrogen and oxygen atmospheres, confirming its excellent thermal stability (Supplementary Fig. 10).

### Reaction optimizations

Upon successfully synthesizing the NPy-DMTP-COF, we next evaluated its photocatalytic activity in the cascade reaction. The tryptamine-derived isocyanide **1a** was selected the as model substrate, along with 2.0 equivalents of diphenylphosphine oxide **2a** as the radical precursor and 2.0 equivalents of NaHCO_3_ as the base. The reaction was carried out under a 40 W blue LED light source with a wavelength of 420 nm at room temperature (Table 1). The cascade reaction catalyzed by NPy-DMTP-COF yielded the *γ*-lactam **3a** as the predominant product with a satisfactory yield of 73% in CH_3_CN at ambient temperature (Table 1, entry 1). Py-DMTP-COF, obtained through the condensation between 4,4’,4”,4”‘-(pyrene-1,3,6,8-tetrayl)tetraaniline (Py) and 2, 5-dimethoxyterephthalaldehyde (DMTP) according to Yang’s procedure^82^ produced **3a** in a yield of only 23% (Table 1, entry 2). Several representative homogeneous photocatalysts displayed disappointing results (Table 1, entries 3–5). We speculate that the photocatalyst primarily facilitates the generation of phosphoryl radicals from compound **2a**. Diffuse-reflectance UV0-vis spectroscopy (UV-vis DRS) indicates that NPy-DMTP-COF and Py-DMTP-COF exhibit comparable absorption maxima around 420 nm (Supplementary Fig. 11). Therefore, the underlying rationale behind this disparity may be attributed to the redox potential of the photocatalysts. Based on an excitation energy (E^0−0^) of 2.28 eV obtained through absorption and emission spectra of NPy-DMTP-COF (Supplementary Fig. 12 and Supplementary Table 2, the excitation and emission spectra of Py-DMTP-COF are shown in Supplementary Fig. 13), the excited-state redox potential of the PC^*^/PC^−^ couple is calculated to be 1.56 V. Cyclic voltammetry (CV) studies reveal that NPy-DMTP-COF possesses a redox potential of −0.72 V, stemming from the PC/PC^−^ redox couple (Supplementary Fig. 14, orange line). Electrochemical measurements further reveal that compound **2a** exhibits an oxidation potential of 1.20 V (Supplementary Fig. 15). These results imply the potential for an electron transfer from **2a** to the excited-state COF (COF^*^), coupled with a proton transfer from **2a** to the base (HCO_3_^−^), resulting in the formation of the phosphoryl radical and the COF radical anion COF^•−^. The inefficacy of Py-DMTP-COF and representative homogeneous photocatalysts further supports this mechanistic rationale. For instance, *fac-*Ir(ppy)_3_ and [Ir(ppy)_2_(dtbbpy)]PF_6_ exhibit excited-state redox potentials of only + 0.31 V (*vs.* SCE) and + 0.66 V (*vs.* SCE)^91^, respectively, which are insufficient to oxidize **2a** (E_1/2_(PC^*^/PC^−^) = + 1.20 V *vs.* SCE) to generate the phosphoryl radical. Similarly, Py-DMTP-COF shows a relatively low excited-state potential (E_1/2_(PC^*^/PC^−^) = 1.04 V *vs.* SCE), accounting for its poor catalytic performance. In addition, 4CzlPN displays a reduction potential of −1.21 V^92^, implying that its radical anion (4CzlPN^•−^) is less prone to return to the ground state via electron transfer, further limiting its activity under these conditions. To evaluate the structural contribution of the extended COF, we tested the activity of the discrete molecular analog (NPy-MP) and the monomer (NPy). Neither exhibited noticeable catalytic activity, highlighting the importance of the ordered, porous framework in promoting the photocatalytic cascade reaction (Table 1, entries 6 and 7). Moreover, commercially available g-C_3_N_4_ and graphene oxide also failed to produce detectable amounts of **3a** (Table 1, entries 8 and 9), probably owing to their lack of well-defined porosity and extended π-conjugation.

**Table 1:** Optimizations of the reaction


Entry	PC	Base	Solvent	Yield/%^*a*^
1	NPy-DMTP-COF	NaHCO_3_	CH_3_CN	73
2^*b*^	Py-DMTP-COF	NaHCO_3_	CH_3_CN	23
3	*fac*-Ir(ppy)_3_	NaHCO_3_	CH_3_CN	0
4	[Ir(ppy)_2_dtbbpy)]PF_6_	NaHCO_3_	CH_3_CN	0
5	4CzIPN	NaHCO_3_	CH_3_CN	0
6	NPy	NaHCO_3_	CH_3_CN	0
7	NPy-MP	NaHCO_3_	CH_3_CN	0
8	g-C_3_N_4_	NaHCO_3_	CH_3_CN	0
9	Graphene oxide	NaHCO_3_	CH_3_CN	0
10	NPy-DMTP-COF	Na_2_HPO_4_	CH_3_CN	70
11	NPy-DMTP-COF	KHCO_3_	CH_3_CN	Trace
12	NPy-DMTP-COF	CsHCO_3_	CH_3_CN	0
13	NPy-DMTP-COF	Na_2_CO_3_	CH_3_CN	0
14	NPy-DMTP-COF	NaHCO_3_	DCM	0
15	NPy-DMTP-COF	NaHCO_3_	Toluene	0
16	NPy-DMTP-COF	NaHCO_3_	THF	21
17	NPy-DMTP-COF	NaHCO_3_	DMF	Trace
18	NPy-DMTP-COF	-	CH_3_CN	0
19^*b*^	NPy-DMTP-COF	NaHCO_3_	CH_3_CN	43
20^*c*^	NPy-DMTP-COF	NaHCO_3_	CH_3_CN	0
21 ^*d*^	NPy-DMTP-COF	NaHCO_3_	CH_3_CN	0

All reactions were carried out on a 0.1 mmol scale with 1.0 eq. isocyanide **1a**, 2.0 eq. diphenylphosphine oxide **2a**, 2 mol% catalyst and 2.0 eq. base in 1.0 mL solvent under irradiation of blue LEDs with N_2_ protection at room temperature. DCM is Dichloromethane. THF is tetrahydrofuran. DMF is *N*,*N*-dimethylformamide. 4CzlPN is commercial 2,4,5,6-tetrakis(carbazol-9-yl)-1,3-dicyanobenzene. NPy [6,6’,6”,6”‘-(pyrene-1,3,6,8-tetrayl)tetrakis(naphthalen-2-amine)] and NPy-MP [(1*E*,1’*E*,1“E,1’“E)-N,N’,N”,N”‘-(pyrene-1,3,6,8-tetrayltetrakis(naphthalene-6,2-diyl))tetrakis(1-(2-methoxyphenyl)methanimine)] are both illustrated in the Supplementary Information. ^*a*^Isolated yield. ^*b*^The reaction was performed with 1.0 eq. base. ^*c*^The reaction was performed in the dark. ^*d*^The reaction was performed under air.

The optical band gap of NPy-DMTP-COF is determined to be 2.12 eV through a Tauc plot analysis (Supplementary Fig. 16). Furthermore, electrochemical Mott-Schottky plots, combined with the optical band gaps, reveal that the conduction band (CB) and valence band (VB) positions of NPy-DMTP-COF are well aligned to facilitate the PCET process (Supplementary Fig. 17 and Supplementary Fig. 18).

Subsequently, a series of bases were evaluated for their ability to facilitate the generation of the phosphoryl radical. Among them, only NaHCO_3_ and Na_2_HPO_4_ produced the desired lactams with satisfactory results (Table 1, entry 1 *vs* entries 10-13). The solvent screening further revealed that the catalytic reactions proceeded efficiently in polar solvents, with acetonitrile affording the optimal yield (Table 1, entry 1 *vs* entries 14-17). The essential role of the base for the cascade reaction was confirmed by control experiments (Table 1, entry 18), and a significant reduction in yield was observed when using 1.0 equivalent of NaHCO_3_ (Table 1, entry 19). These results indicate that the base might participate in both the cyclization and subsequent processes. As anticipated, no product was formed in the absence of light or under air atmosphere (Table 1, entries 20 and 21). Therefore, the optimized conditions involved the use of NPy-DMTP-COF as catalyst and NaHCO_3_ as the base in CH_3_CN at room temperature (Table 1, entry 1).

### Substrate scope

**Fig. 3:**
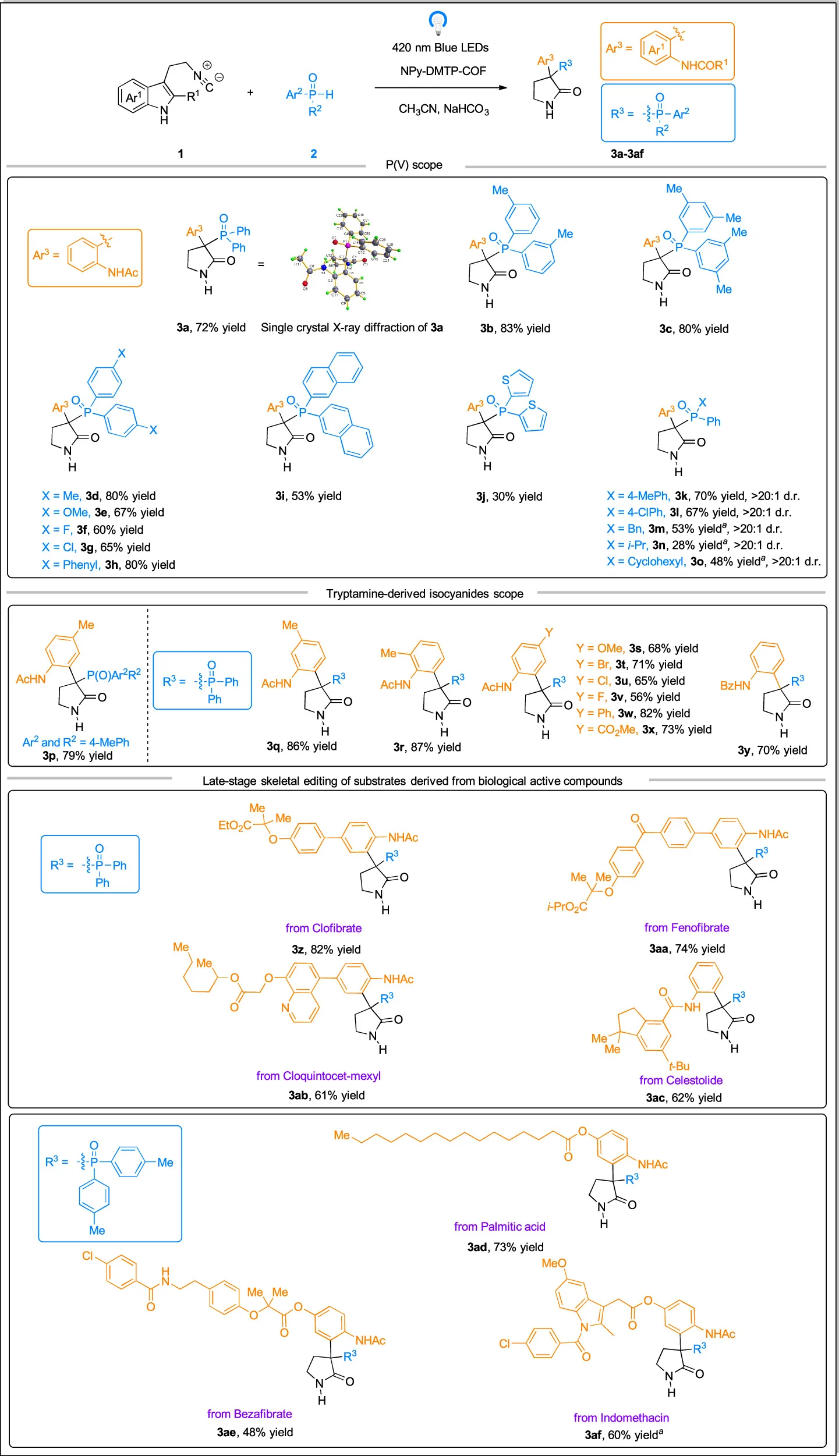
Substrates scope of photocatalytic reactions between tryptamine-derived isocyanides and phosphine oxides. All reactions were carried out on a 0.3 mmol scale with 1.0 eq. isocyanides **1**, 2.0 eq. phosphine oxides **2**, 2 mol% NPy-DMTP-COF and 2.0 eq. NaHCO_3_ in 4.0 mL CH_3_CN under irradiation of 420 nm blue LEDs with N_2_ protection at room temperature for 48–96 h. Isolated yield. ^a^2.0 eq. Na_2_HPO_4_ served as base instead of NaHCO_3_.

Under the optimized reaction conditions, we next explored the substrate scope with various phosphine oxides and tryptamine-derived isocyanides to investigate the generality of this photocatalytic cascade reaction (Fig. 3). The protocol proved applicable to a broad range of diarylphosphine oxides bearing diverse substituents, including methyl, halogens, alkoxy groups and phenyl (**3a**−**3i**), affording the corresponding lactams in 53-83% yields. Both electron-donating and electron-withdrawing substituents at either the *para-* or *meta-*positions of the benzene ring were well tolerated. In addition, a 2-thienyl substituted phosphine oxide also participated smoothly, affording the desired lactam **3j** in 30% yield. Furthermore, phosphine oxides featuring two different aryl substituents demonstrated excellent reactivity, successfully yielding products **3k** and **3 l** in good yields with d.r. values exceeding 20: 1. Notably, alkylphenylphosphine oxides were identified as suitable phosphorus reagents for this protocol, affording the corresponding products **3m–3o** in moderate yields while maintaining d.r. values exceeding 20: 1. In contrast, dialkylphosphine oxides remained unreactive toward 1a, probably because the required PCET process between dialkylphosphine oxide and NPy-DMTP-COF^*^ is disfavored (Supplementary Fig. 19). Single-crystal X-ray diffraction analysis of **3a** verified the structure.

After establishing the scope of phosphine oxides, the effects of tryptamine-derived isocyanides on the reaction outcome were subsequently evaluated. As shown in Fig. 3, a wide range of tryptamine-derived isocyanides bearing different substituted aryl groups reacted smoothly with diarylphosphine oxides, affording the corresponding 3-(2-aminophenyl)-*γ*-lactams **3p**−**3x** in moderate to good yields (56-86%). In particular, 2-phenyl substituted tryptamine-derived isocyanide was well tolerated, affording the product **3y** in 70% yield.

The mild reaction conditions and high functional group tolerance could facilitate the late-stage functionalization of complicated drug or bioactive molecules. The reactions were highly compatible with tryptamine-derived isocyanides from Clofibrate, Fenofibrate and Cloquintocet-mexyl, giving the desired lactams **3z**−**3ab** in 61-82% yields. In addition, the 2-aryl substituted Celestolide-derived isocyanide was proved to be suitable for the reaction, affording the lactam **3ac** in 62% yield. Furthermore, acyloxy substituted isocyanides from Palmitic acid, Bezafibrate and Indomethacin were also well tolerated in the optimized conditions and the corresponding lactams **3ad**−**3af** were obtained in 48-73% yield. These results demonstrate that the current method provides a reliable and powerful protocol for access to functionalized lactams.

**Fig. 4:**
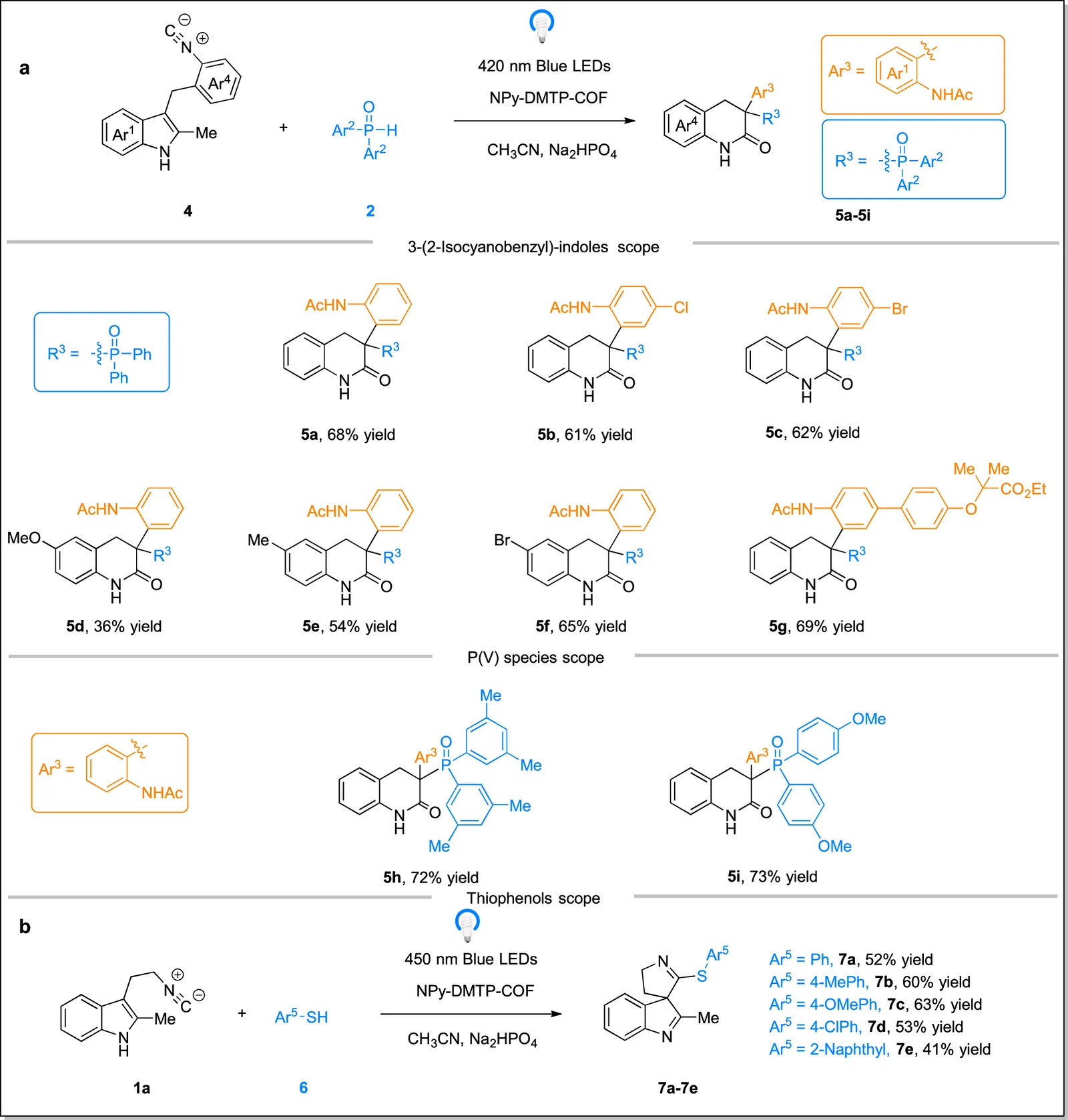
Substrate scope for (a) photocatalytic reactions of 3-(2-isocyanobenzyl)-indoles with phosphine oxides and (b) photocatalytic cyclizations of tryptamine-derived isocyanides with thiophenols. **a** All reactions were carried out on a 0.3 mmol scale with 1.0 eq. isocyanides **4**, 2.0 eq. phosphine oxide**s 2**, 2 mol% NPy-DMTP-COF and 2.0 eq. Na_2_HPO_4_ in 4.0 mL CH_3_CN under irradiation of 420 nm blue LEDs with N_2_ protection at room temperature for 48–96 h. **b** All reactions were carried out on a 0.3 mmol scale with 1.0 eq. isocyanides 1, 2.0 eq. thiophenols 6, 2 mol% NPy-DMTP-COF and 2.0 eq. Na_2_HPO_4_ in 4.0 mL CH_3_CN under irradiation of 450 nm blue LEDs with N_2_ protection at room temperature for 48–96 h. Isolated yield.

The substrates scope of the cascade reactions was further extended to 3-(2-isocyanobenzyl)-indoles (Fig. 4a). A variety of 3-(2-aminophenyl)-tetrahydroquinolines **5a**−**5 f** were successfully synthesized via a cascade reaction between a diverse array of 3-(2-isocyanobenzyl)-indoles and diphenylphosphine oxide **2a**. The reaction was highly compatible with 3-(2-isocyanobenzyl)-indole from Clofibrate, yielding the desired tetrahydroquinoline **5 g** with a 69% yield. Moreover, diarylphosphine oxides with different groups demonstrated high efficacy in the reaction, giving tetrahydroquinolines **5 h** or **5i** with high yields. To further demonstrate the versatility of this catalytic platform, the scope was expanded to include additional radical precursors (Supplementary Fig. 19). Notably, a diverse set of thiophenols **6a−6e** proved competent after optimization, delivering the corresponding spiroindolenines **7a−7e** in moderate yields (Fig. 4b).

### Practicality of the reactions

**Fig. 5:**
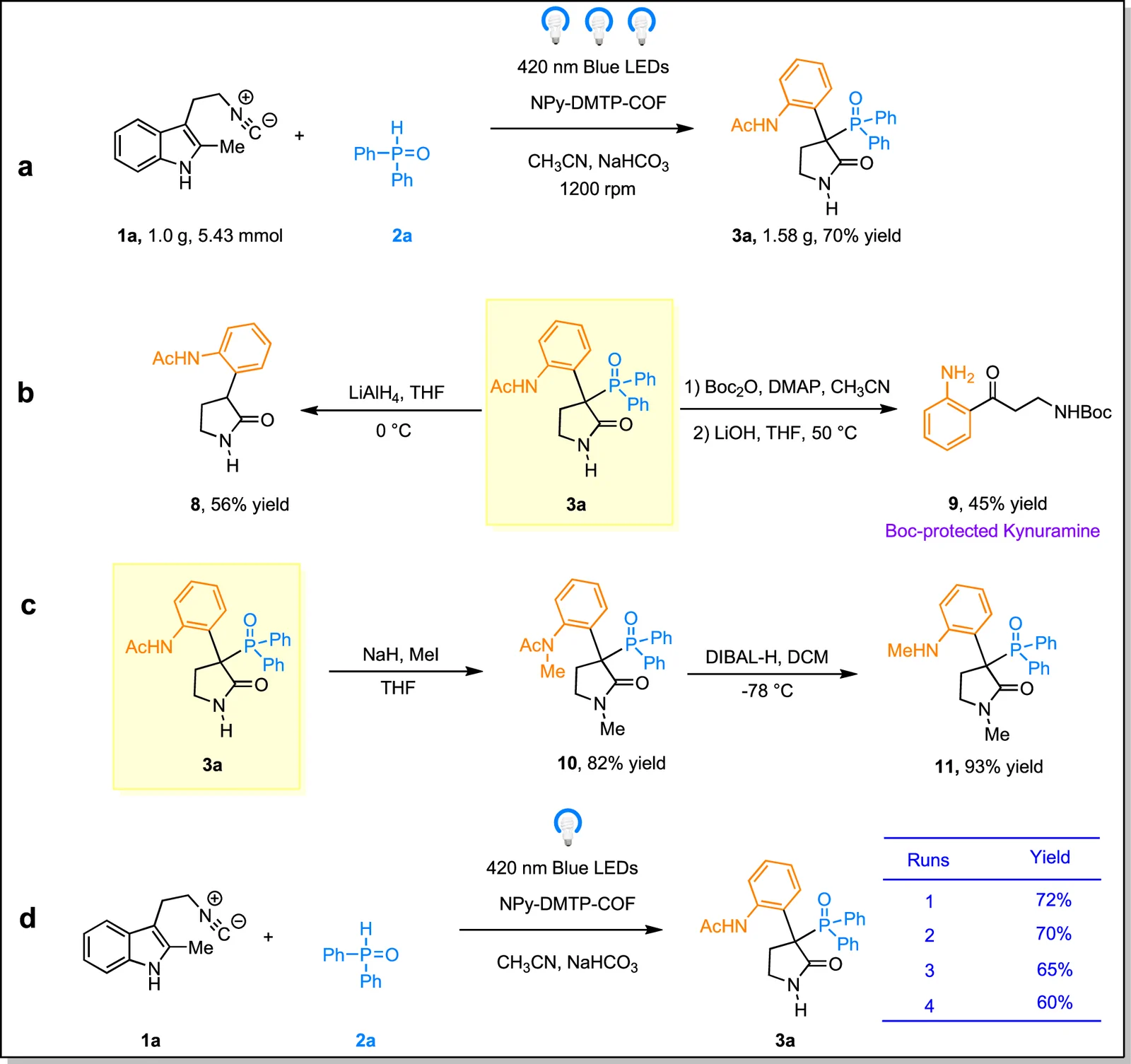
Practicality of the reactions. **a** Gram scale reaction. **b**, **c** Synthetic transformation. **d** Yields of **3a** with recovered NPy-DMTP-COF in four consecutive runs.

To further demonstrate the practicality of this reaction, the cascade reaction between **1a** and **2a** was carried out on a gram scale under three blue LED lamps and 1200 rpm. The corresponding product **3a**, could be obtained in a 70% yield (Fig. 5a). To assess the synthetic utility of this methodology, lactam **3a** was subjected to a reduction with LiAlH_4_, delivering the lactam **8** with a yield of 56% (Fig. 5b). Moreover, the Boc-protected Kynuramine **9**, identified as a potential inhibitor of neural nitric oxide synthase, was synthesized with a yield of 45% via a deprotection of the Boc-protected precursor **3a** using lithium hydroxide (Fig. 5b). Functionalized lactam **11**, which shares a structural resemblance with Chimonamidine, was successfully synthesized through the reduction of the *N*-methyl protected intermediate **3a** using DIBAL (Fig. 5c). Finally, the heterogeneous nature of COF endowed it with recyclability and reusability. After the completion of the reaction, NPy-DMTP-COF could be readily recovered by simple centrifugation. In the reaction between **1a** and **2a**, NPy-DMTP-COF was successfully retrieved and reused across four subsequent cycles, with little diminution in its catalytic performance (Fig. 5d). PXRD analysis of the recycled NPy-DMTP-COF displays only a slight decline in crystallinity (Supplementary Fig. 20), while BET measurements confirm the preservation of its porous architecture, with a surface area of 1214 m^2^/g, pore volume of 0.85 cm^3^/g, and pore width of 2.27 nm (Supplementary Fig. 21, Supplementary Fig. 22 and Supplementary Table 3).

### Mechanistic studies

**Fig. 6:**
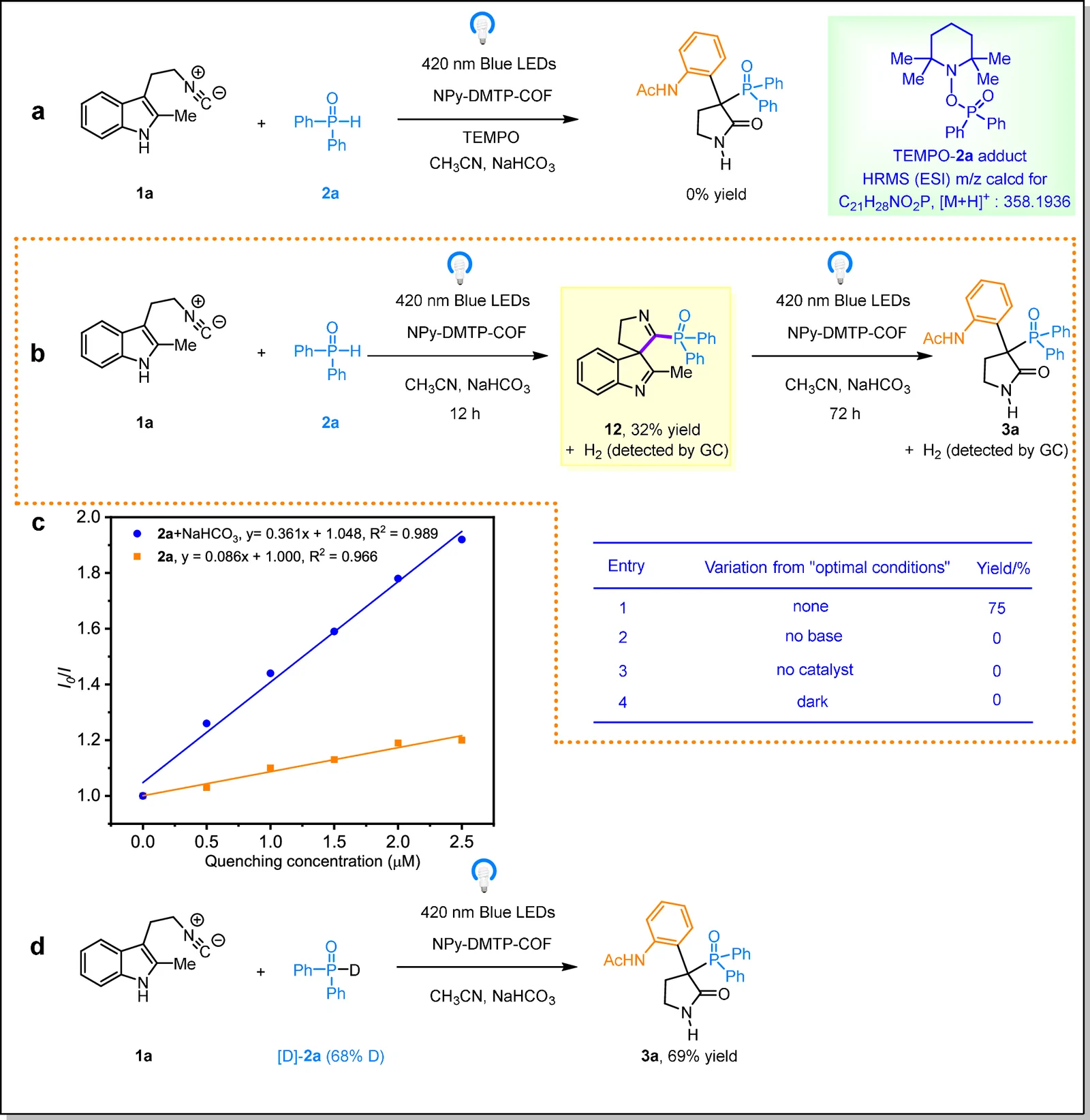
Controlled experiments. **a** Radical prevention experiment. **b** The experiment involving in the isolation of intermediate **12** and the synthesis of **3a** from **12** via photocatalytic reaction. **c** Stern-Volmer quenching studies. **d** Deuterium labeling experiment.

To gain insight into the reaction mechanism, a series of mechanistic studies were carried out (Fig. 6). Initially, 2.0 equiv of 2,2,6,6-tetramethylpiperidin-1-yl)-oxidanyl (TEMPO) was enough to quench the reaction of **1a** and **2a** (Fig. 6a). A TEMPO-**2a** adduct was detected by high-resolution mass spectrometry, indicating that phosphoryl radical might be generated photochemically (Supplementary Fig. 23). Furthermore, when the reaction between **1a** and **2a** was carried out under optimal conditions for 12 h, the spiroindolenine **12** was isolated with a yield of 32% (Fig. 6b) and H_2_ was detected by gas chromatography (GC) (Supplementary Fig. 24). Spiroindolenine **12** was then employed as a substrate in a photocatalytic reaction, successfully yielding lactam **3a** with a 75% yield, and H_2_ was again identified by GC analysis (Supplementary Fig. 25). The reaction could not proceed in the absence of base, photocatalyst or blue LEDs. These experiments indicate that spiroindolenine **12** is the key intermediate in this cascade reaction. Moreover, it suggests that the HCO_3_^−^ may act as an electron acceptor, abstracting an electron from COF ^−^ to regenerate the ground state COF, while simultaneously producing H_2_ as a byproduct. Subsequently, to elucidate the interaction between NPy-DMTP-COF and the phosphine oxide, fluorescence quenching experiments (Supplementary Figs. 26-28) and Stern-Volmer analysis (Fig. 6c) were performed. The luminescence emission of NPy-DMTP-COF was quenched effectively by [**2a** + NaHCO_3_] mixture instead of **2a**, indicating that an interaction between NPy-DMTP-COF and [**2a** + NaHCO_3_] mixture might exist to promote this reaction. The results above hint at an exclusive PCET process between NPy-DMTP-COF and phosphine oxide. Moreover, an isotope-labeling study was carried out using deuterated D-P(O)Ph_2_ and isocyanide **1a** under standard reaction conditions, leading to the synthesis of **3a** in a 69% yield with no hydrogen atom being deuterated (Fig. 6d, Supplementary Fig. 29 and Supplementary Fig. 30). It suggests that the deuterium atom in D-P(O)Ph_2_ is likely captured by NaHCO_3_.

**Fig. 7:**
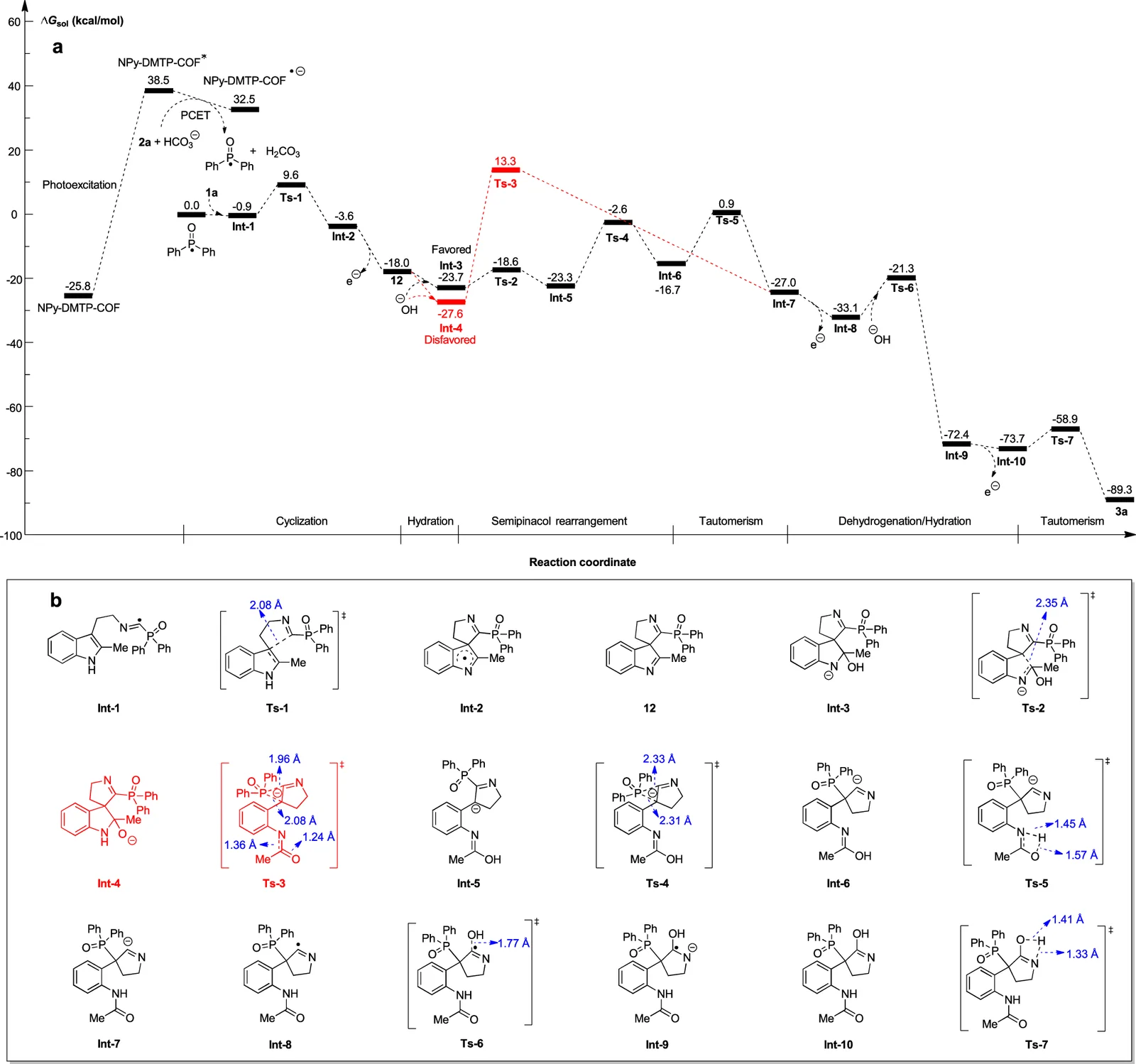
The DFT studies of photocatalytic cascade reaction between 1a and 2a. All energies were calculated at the M06-2X-D3 functional level, utilizing the def2-TZVP basis set. **a** Gibbs free energy of reaction paths. **b** Intermediates and transition states for this catalytic system.

To explore the potential pathways involved in the photocatalytic cascade reaction, we employed DFT calculations, incorporating Grimme’s D3 dispersion correction (Fig. 7). All calculations were carried out with the Gaussian16 program package^93^. All the structures were fully optimized based on PBE0-D3/6-311 G(d,p) basis set level^94,95^. All optimized geometries were characterized as either local minima (no imaginary frequencies) or transition states (one imaginary frequency) through vibrational frequency analysis at the same level of theory. The connection of each transition state was validated by intrinsic reaction coordinate (IRC) calculations. Subsequently, Gibbs free energies were computed via M06-2X-D3/def2TZVP single-point energy calculations on the optimized structures, incorporating thermal corrections at 298.15 K and 1.0 atm. TD-DFT study was conducted at same level within the adiabatic approximation to predict the excitation energies. The Marcus theory was applied to evaluate the PCET process and the single electron transfer process. To evaluate solvation effects of acetonitrile, the IEFPCM model was applied based on the gas-phase optimized structures of all the species.

Initially, NPy-DMTP-COF is selectively excited under blue LEDs, with a free energy excitation of 64.3 kcal/mol, which is significantly lower than that of the reactants **1a** and **2a**, at 126.3 kcal/mol and 145.1 kcal/mol, respectively. This initiates a PCET process, where an electron is transferred from **2a** to NPy-DMTP-COF, accompanied by a proton transfer from **2a** to HCO_3_^−^. This results in the formation of a phosphoryl radical and a COF radical anion, NPy-DMTP-COF^•−^. The phosphoryl radical then reacts with **1a** to form **Int-1**, with a Gibbs free energy change of –0.9 kcal/mol. Potential energy surface (PES) scanning confirms that this reaction step is barrier-free. **Int-1** subsequently undergoes cyclization to form **Int-2** via the transition state **Ts-1**, requiring an activation energy of 10.5 kcal/mol. **Int-2** undergoes an electron transfer with NPy-DMTP-COF^*^ and HCO_3_^−^ to yield spiroindolenine **12**, a spontaneous process with a Gibbs free energy change of 14.4 kcal/mol (Fig. 7a). The formation of spiroindolenine **12** has been confirmed experimentally (Fig. 6b).

A nucleophilic addition then occurs, where a hydroxide anion attacks spiroindolenine **12**, followed by semipinacol rearrangement and tautomerism to yield **Int-7**. This reaction pathway splits into two routes. The favored route (black line) involves the formation of **Int-3**, followed by a ring-opening reaction via **Ts-2**, with an energy barrier of 5.1 kcal/mol, leading to **Int-5**. The subsequent rate-determining step involves 1,2-phosphonyl migration through **Ts-4**, with an energy barrier of 20.7 kcal/mol, leading to the formation of **Int-6**. Tautomerization through **Ts-5**, with an energy barrier of 17.6 kcal/mol yields amide **Int-7**. The alternative route (red line), involving a one-step rearrangement from **Int-4** to **Ts-3**, is less favored due to a high energy barrier of 40.9 kcal/mol.

**Int-7** undergoes a SET process to generate the radical intermediate **Int-8**. Nucleophilic attack by a hydroxide anion at the radical center then proceeds via **Ts-6** with an energy barrier of 11.8 kcal/mol to afford the stabilized anionic species **Int-9**. A subsequent SET process from **Int-9** yields the neutral intermediate **Int-10**, which tautomerizes through **Ts-7** with an energy barrier of 14.8 kcal/mol to deliver the final product **3a**. All the intermediates and transition states for this catalytic system are illustrated in Fig. 7b.

Here, we report the construction of a well-ordered framework, NPy-DMTP-COF, with designed porosity that can be efficiently excited under visible light. This catalyst facilitates a photocatalytic cascade reaction between tryptamine-derived isocyanides and phosphine oxides, yielding a diverse array of 3-(2-aminophenyl)-*γ*-lactams with moderate to high yields and excellent diastereoselectivities ( > 20:1). 3-(2-isocyanobenzyl)-indoles are also tolerated, giving (2-aminophenyl)-tetrahydroquinolines in moderate yield. Notably, NPy-DMTP-COF could be readily recovered via simple centrifugation and reused over four consecutive cycles without noticeable loss of catalytic activity, highlighting its advantage over conventional homogeneous catalysts. The controlled experiments and DFT studies reveal that a PCET process occurs between the excited NPy-DMTP-COF^*^ and phosphine oxide, initiating a cascade reaction including cyclization, semipinacol rearrangement and dehydrogenation/hydration processes. Notably, the in situ generated spiroindolenine serves as the key intermediate in this reaction. The current work not only expands the repertoire of 3-(2-aminophenyl)-*γ*-lactams and (2-aminophenyl)-tetrahydroquinolines but also establishes a COF-based photocatalytic system for cascade reactions.

## Methods

### General information

Reactions were monitored by thin layer chromatography (TLC), and column chromatography purifications were carried out using silica gel. Column chromatography was performed on silica gel (300–400 mesh). ^1^H, ^13^C, ^19^F and ^31^P NMR spectra were collected on a Bruker AV 400, 600 or 700 MHz NMR spectrometer using residue solvent peaks as an internal standard (^1^H NMR: CDCl_3_ at 7.26 ppm, DMSO-*d*_6_ at 2.50 ppm; ^13^C NMR: CDCl_3_ at 77.00 ppm, DMSO-*d*_6_ at 30.92 ppm). Data for ^1^H and ^13^C NMR were recorded as follows: chemical shift (δ, ppm), multiplicity (s = singlet; d = doublet; t = triplet; q = quarter; m = multiplet; br = broad), coupling constant (Hz), integration. High resolution mass spectra for all the new compounds were done by an LTQ-Orbitrap instrument (ESI) (Thermo Fisher Scientific, USA). Catalysts and substrates **2** and **6** were purchased from J&K Scientific (China) Co., LTD. Substrates **1** and **4** were synthesized by following the published procedures^35^.

Powder X-ray powder diffraction (PXRD) patterns were measured on a Rigaku RINT Smartlab SE powder diffraction system, equipped with Cu Kα radiation (λ = 1.54 Å). Fourier-transform infrared spectrometer (FT-IR) data were collected using a Bruker VERTEX 70 spectrophotometer with KBr disks. Solid-state ^13^C-CP/TOSS NMR spectra were performed on a Bruker 500 MHz spectrometer. Nitrogen sorption isotherms were recorded on a Quantachrome Autosorb 1 at 77 K. Prior to the measurements, the samples were outgassed for 12 h at 120 °C under high vacuum. Scanning electron microscopy (SEM) was performed using a ZEISS Gemini 560 operated at an accelerating voltage of 2.0 kV. High resolution transmission electron microscopy (HR-TEM) was performed using a JEM-2100F instrument (JEOL, Japan) operated at 200 kV. Dynamic light scattering (DLS) was performed on Zeta-potential & Particle size analyzer (Malvern ZS90). NPy-DMTP-COF was dispersed in CH_3_CN (0.05 mg/mL), sonicated for 60 min, and left to stand for 10 min prior to DLS measurements. Thermal gravimetric analyses (TGA) were performed on a Mettler Toledo’s TGA/DSC 3+ Synchronous Thermal Analyzer. The process of simulating COF structure was performed by the Materials Studio software. The triclinic lattice with P1 symmetry group was set as the initial eclipsed COF structure. After the smallest asymmetric fragment was filled into the blank cell, the Forcite tools package was employed to optimize the cell geometry including energy minimization. The cell optimized from the Universal force fields was subsequently refined using the Pawley refinement method in Reflex tools.

### Preparation of NPy-DMTP-COF

6,6’,6”,6”‘-(pyrene-1,3,6,8-tetrayl)tetrakis(naphthalen-2-amine) (NPy) (38.3 mg, 0.05 mmol) and 2,5-dimethoxyterephthalaldehyde (DMTP) (19.4 mg, 0.1 mmol) and CH_3_COOH (0.1 mL, 6 M) were mixed in a mixture of solvents containing 0.25 mL of *o*-dichlorobenzene and 0.75 mL of 1-butanol in a sealed tube. The mixture was ultrasonicated for 10 min and then flash frozen at 77 K (liquid N_2_ bath) and degassed through three freeze-pump-thaw cycles. The mixture was then heated at 120 °C for 72 h under an N_2_ atmosphere. The resulting solid product was recovered by filtration, washed with methanol and DCM, and subjected to Soxhlet extraction using THF for 24 h. After drying under vacuum for 6 h, a brown solid was obtained with a yield of 87%. This material was designated as NPy-DMTP-COF.

### General experimental procedure of photocatalytic cascade reaction

At N_2_ atmosphere, NPy-DMTP-COF (5.3 mg, 0.006 mmol based on the repeating unit), **1** or **4** (0.3 mmol), **2** (0.6 mmol), and NaHCO_3_ (50.4 mg, 0.6 mmol) were mixed in acetonitrile (4.0 mL) in a Schlenk tube. The resulting mixture was stirred under blue LED irradiation (420 nm) at room temperature for 48–96 h. Upon completion, the reaction mixture was filtered through celite pad and the filtrate was concentrated. The mixture was purified by silica gel flash chromatography (petroleum ether:ethyl acetate, 1:3) to afford the desired product **3** or **5**.

## Supplementary information

Supplementary Information

Transparent Peer review file

## Source data

Source data

## Data Availability

Crystallographic data for the structures reported in this article have been deposited at the Cambridge Crystallographic Data Center, under deposition numbers CCDC 2368727 (**3a**). Copies of the data can be obtained free of charge via https://www.ccdc.cam.ac.uk/structures/. The characterizations of NPy-DMTP-COF, spectral data of products and cartesian coordinates generated in this study are provided in the Supplementary Information and Source Data file. All data are available from the corresponding author upon request. Source data are provided with this paper.

## References

[1] Ojima, I. Recent advances in the.beta.-lactam synthon method. *Acc. Chem. Res.* **28**, 383–389 (1995).

[2] Ojima, I. & Delaloge, F. Asymmetric synthesis of building-blocks for peptides and peptidomimetics by means of the *β*-lactam synthon method. *Chem. Soc. Rev.* **26**, 377–386 (1997).

[3] Hong, S. Y. et al. Selective formation of *γ*-lactams via C−H amidation enabled by tailored iridium catalysts. *Science* **359**, 1016–1021 (2018). 29496875 10.1126/science.aap7503

[4] Caruano, J., Muccioli, G. G. & Robiette, R. Biologically active *γ*-lactams: Synthesis and natural sources. *Org. Biomol. Chem.* **14**, 10134–10156 (2016). 27748489 10.1039/c6ob01349j

[5] Hayashi, M., Bachman, S., Hashimoto, S., Eichman, C. C. & Stoltz, B. M. Ni-catalyzed enantioselective C-acylation of *α*-substituted lactams. *J. Am. Chem. Soc.* **138**, 8997–9000 (2016). 27373124 10.1021/jacs.6b02120 PMC4963289

[6] Chen, C. et al. Rhodium/Yanphos-catalyzed asymmetric interrupted intramolecular hydroaminomethylation of trans-1,2-disubstitutedalkenes. *J. Am. Chem. Soc.* **138**, 9017–9020 (2016). 27385399 10.1021/jacs.6b03596

[7] Wang, C. & Ge, S. Versatile cobalt-catalyzed enantioselective entry to boryl-functionalized all-carbon quaternary stereogenic centers. *J. Am. Chem. Soc.* **140**, 10687–10690 (2018). 30114355 10.1021/jacs.8b06814

[8] Wang, Z., Yin, H. & Fu, G. C. Catalytic enantioconvergent coupling of secondary and tertiary electrophiles with olefins. *Nature* **563**, 379–383 (2018). 30337711 10.1038/s41586-018-0669-y PMC6296363

[9] Jette, C. et al. Palladium-catalyzed construction of quaternary stereocenters by enantioselective arylation of *γ*-lactams with aryl Chlorides and Bromides. *Angew. Chem. Int. Ed.* **58**, 4297–4301 (2019). 10.1002/anie.201814475 30677201

[10] Park, Y. & Chang, S. Asymmetric formation of *γ*-lactams via C−H amidation enabled by chiral hydrogen-bond-donor catalysts. *Nat. Catal.* **2**, 219–227 (2019).

[11] Xing, Q., Chan, C.-M., Yeung, Y.-W. & Yu, W.-Y. Ruthenium(II)-catalyzed enantioselective *γ*-lactams formation by intramolecular C-H amidation of 1,4,2-dioxazol-5-ones. *J. Am. Chem. Soc.* **141**, 3849–3853 (2019). 30785737 10.1021/jacs.9b00535

[12] Wang, H. et al. Iridium-catalyzed enantioselective C(sp^3^)−H amidation controlled by attractive noncovalent interactions. *J. Am. Chem. Soc.* **141**, 7194–7201 (2019). 30978019 10.1021/jacs.9b02811

[13] Bartoszewicz, A., Matier, C. D. & Fu, G. C. Enantioconvergent alkylations of amines by alkyl electrophiles: Copper-catalyzed nucleophilic substitutions of racemic *α*-halolactams by indoles. *J. Am. Chem. Soc.* **141**, 14864–14869 (2019). 31496239 10.1021/jacs.9b07875 PMC7055584

[14] Ashida, K. et al. Enantioselective synthesis of polycyclic *γ*-lactams with multiple chiral carbon centers via Ni(0)-catalyzed asymmetric carbonylative cycloadditions without stirring. *J. Am. Chem. Soc.* **142**, 1594–1602 (2020). 31868355 10.1021/jacs.9b12493

[15] Wu, X., Qu, J. & Chen, Y. Quinim: A new ligand scaffold enables nickel-catalyzed enantioselective synthesis of *α*-alkylated *γ*-lactam. *J. Am. Chem. Soc.* **142**, 15654–15660 (2020). 32845137 10.1021/jacs.0c07126

[16] Li, Y. et al. Carbamoyl fluoride-enabled enantioselective Ni-catalyzed carbocarbamoylation of unactivated alkenes. *J. Am. Chem. Soc.* **142**, 19844–19849 (2020). 33170685 10.1021/jacs.0c09949

[17] Rhuzhaev, V. U., Tashkhodzhaev, B. & Aripova, S. F. Alkaloids of Arundo donax III. Reconsideration of the structures of donaxarine and donaxadidine. *Chem. Nat. Compd.* **31**, 604–609 (1995).

[18] Takayama, H. et al. Isolation, structure elucidation, and total synthesis of two new chimonanthus alkaloids, chimonamidine and chimonanthidine. *Tetrahedron* **60**, 893–900 (2004).

[19] Takano, S., Moriya, M.& Ogasawara, K. Enantiocontrolled total syntheses of (-)-physovenine and (-)-physostigmine. *J. Org. Chem*. **56**, 5982–5984 (1991).

[20] Subba Reddy, U. V. et al. Diamino alcohol catalyzed enantioselective crossed aldol reaction of acetaldehyde with isatins−aconcise total synthesis of antitumor agents. *Eur. J. Org. Chem.* **2017**, 3874–3885 (2017).

[21] Braire, J., Dorcet, V., Vidal, J., Lalli, C. & Carreaux, F. BINOL derivatives-catalysed enantioselective allylboration of isatins: Application to the synthesis of (*R*)-chimonamidine. *Org. Biomol. Chem.* **18**, 6042–6046 (2020). 32729604 10.1039/d0ob01386b

[22] Chen, W.-B., Du, X.-L., Cun, L.-F., Zhang, X.-M. & Yuan, W.-C. Highly enantioselective aldol reaction ofacetaldehyde and isatins only with 4-hydroxydiarylprolinol as catalyst: Concise stereoselective synthesis of (*R*)-convolutamydines B and E, (−)-donaxaridine and (*R*)-chimonamidine. *Tetrahedron* **66**, 1441–1446 (2010).

[23] Zhu, B. et al. Direct asymmetric vinylogous aldol reaction of allyl ketones with isatins: divergent synthesis of 3-hydroxy-2-oxindole derivatives. *Angew. Chem. Int. Ed.* **52**, 6666–6670 (2013). 10.1002/anie.201302274 23650192

[24] Peng, Y.-S. et al. Copper-catalyzed oxygenative skeletal rearrangement of tetrahydro-*β*-carbolines using H_2_O and O_2_ as oxygen sources. *Angew. Chem. Int. Ed.* **62**, e202313687 (2023). 10.1002/anie.202313687 37950324

[25] Sun, Y.-Z. et al. Copper/iodine Co-catalyzed oxygenative transannulation of tryptamines enables direct synthesis of donaxaridine and its derivatives. *Org. Lett.* **26**, 625–630 (2024). 38206574 10.1021/acs.orglett.3c03942

[26] Zhao, X. et al. Asymmetric dearomatization of indoles through a Michael/Friedel–Crafts-type cascade to construct polycyclic spiroindolines. *Angew. Chem. Int. Ed.* **54**, 4032–4035 (2015). 10.1002/anie.201410814 25650250

[27] Saya, J. M. et al. Iodospirocyclization of tryptamine-derived isocyanides: Formal total synthesis of aspidofractinine. *Angew. Chem. Int. Ed.* **57**, 15232–15236 (2018). 10.1002/anie.201809678 30273989

[28] Chen, G.-S. et al. Tandem cross-coupling/spirocyclization/Mannich-type reactions of 3-(2-isocyanoethyl) indoles with diazo compounds toward polycyclic spiroindolines. *Angew. Chem. Int. Ed.* **59**, 614–621 (2020). 10.1002/anie.201911614 31729132

[29] Jiang, S., Cao, W.-B., Li, H.-Y., Xu, X.-P. & Ji, S.-J. Convenient synthesis of spiroindolenines from tryptamine-derived isocyanides and organic azides by cobalt catalysis in pure water. *Green. Chem.* **23**, 2619–2623 (2021).

[30] Cao, W.-B. et al. *Syn*-Stereoselective C3-spirocyclization and C2-amination of 3-(2-isocyanoethyl)indole using C, N-cyclic azomethine imines. *Org. Lett.* **24**, 4620–4624 (2022). 35730796 10.1021/acs.orglett.2c01736

[31] Wang, X., Dong, J., Wu, T., Xu, X. & Tang, B. Divergent synthesis of chromenoindoles and spiroindolines via domino reaction of indolyl-substituted isocyanides with quinone esters. *Org. Lett.* **24**, 6700–6704 (2022). 36094394 10.1021/acs.orglett.2c02192

[32] Zhu, Y.-M., Xu, X.-P. & Ji, S.-J. Divergent synthesis of pentacyclic isoindolinones enabled by sequential insertion of two different isocyanides and acid promoted cyclization of ketenimines. *Org. Lett.* **25**, 2041–2046 (2023). 36946492 10.1021/acs.orglett.3c00393

[33] Zheng, J. et al. Diastereodivergent synthesis of pentacyclic spiroindolines via a magnesium(II)-catalyzed cascade reaction of *N*, *N*′-cyclic azomethine imines with indolyl-substituted isocyanides. *Org. Lett.* **25**, 3829–3834 (2023). 37196242 10.1021/acs.orglett.3c01085

[34] Yuan, L.-R., Ji, S.-J. & Xu, X.-P. Coupling-spirocyclization cascade of tryptamine-derived isocyanides with iodonium ylides and despirocyclization reactions. *Org. Lett.* **25**, 7858–7862 (2023). 37862138 10.1021/acs.orglett.3c03090

[35] Wu, M. et al. Shining light on tryptamine-derived isocyanides: Access to constrained spirocylic scaffolds. *Chem. Sci.* **15**, 6867–6873 (2024). 38725510 10.1039/d3sc06304f PMC11077512

[36] Liu, Y. et al. 4CzIPN-^*t*^Bu-catalyzed proton-coupled electron transfer for photosynthesis of phosphorylated *N*-heteroaromatics. *J. Am. Chem. Soc.* **143**, 964–972 (2021). 33373207 10.1021/jacs.0c11138

[37] Yu, J.-X., Cheng, Y.-Y., Chen, B., Tung, C.-H. & Wu, L.-Z. Cobaloxime photocatalysis for the synthesis of phosphorylated heteroaromatics. *Angew. Chem. Int. Ed.* **61**, e202209293 (2022). 10.1002/anie.202209293 35912895

[38] Jiang, H. et al. Synthesis of 6-alkylated phenanthridine derivatives using photoredox neutral somophilic isocyanide insertion. *Angew. Chem. Int. Ed.* **52**, 13289–13292 (2013). 10.1002/anie.201308376 24222447

[39] Li, C.-X., Tu, D.-S., Yao, R., Yan, H. & Lu, C.-S. Visible-light-induced cascade reaction of isocyanides and *N*-arylacrylamides with diphenylphosphine oxide via radical C−P and C−C bond formation. *Org. Lett.* **18**, 4928–4931 (2016). 27681056 10.1021/acs.orglett.6b02413

[40] Wang, C.-H., Li, Y.-H. & Yang, S.-D. Autoxidation photoredox catalysis for the synthesis of 2-phosphinoylindoles. *Org. Lett.* **20**, 2382–2385 (2018). 29624398 10.1021/acs.orglett.8b00722

[41] Cao, W.-B. et al. Hydrogen-bonding-promoted cascade rearrangement involving the enlargement of two rings: Efficient access to polycyclic quinoline derivatives. *Angew. Chem. Int. Ed.* **59**, 21425–21430 (2020). 10.1002/anie.202008110 32798266

[42] Zhang, Y. et al. Selective C(sp^3^)–H activation of simple alkanes: Visible light-induced metal-free synthesis of phenanthridines with H_2_O_2_ as a sustainable oxidant. *Green. Chem.* **23**, 6926–6930 (2021).

[43] Ye, H.-B., Zhou, X.-Y., Li, L., He, X.-K. & Xuan, J. Photochemical synthesis of succinic ester-containing phenanthridines from diazo compounds as 1, 4-dicarbonyl precursors. *Org. Lett.* **24**, 6018–6023 (2022). 35947775 10.1021/acs.orglett.2c02313

[44] Banjare, S. K. et al. Thermal and photoinduced radical cascade annulation using aryl isonitriles: an approach to quinoline-derived benzophosphole oxides. *Angew. Chem. Int. Ed.* **63**, e202404275 (2024). 10.1002/anie.202404275 38687058

[45] Zhang, E., Fan, C.-A., Tu, Y.-Q., Zhang, F.-M. & Song, Y.-L. Organocatalytic asymmetric vinylogous *α*-ketol rearrangement: Enantioselective construction of chiral all-carbon quaternary stereocenters in spirocyclic diketones via semipinacol-type 1,2-carbon migration. *J. Am. Chem. Soc.* **131**, 14626–14627 (2009). 19785419 10.1021/ja906291n

[46] Zhang, Q.-W. et al. Brønsted acid catalyzed enantioselective semipinacol rearrangement for the synthesis of chiral spiroethers. *Angew. Chem. Int. Ed.* **48**, 8572–8574 (2009). 10.1002/anie.200904565 19816900

[47] Chen, Z.-M. et al. Organocatalytic asymmetric halogenation/semipinacol rearrangement: Highly efficient synthesis of chiral *α*-oxa-quaternary *β*-haloketones. *J. Am. Chem. Soc.* **133**, 8818–8821 (2011). 21548637 10.1021/ja201794v

[48] Li, H. et al. Enantioselective bromination/semipinacol rearrangement for the synthesis of *β*-bromoketones containing an all-*α*-carbon quaternary center. *Chem. Sci.* **2**, 1839–1841 (2011).

[49] Chai, V. & Rainey, T. J. Pd(II)/Brønsted acid catalyzed enantioselective allylic C–H activation for the synthesis of spirocyclic rings. *J. Am. Chem. Soc.* **134**, 3615–3618 (2012). 22313246 10.1021/ja2102407

[50] Romanov-Michailidis, F., Guénée, L. & Alexakis, A. Enantioselective organocatalytic fluorination-induced Wagner-Meerwein rearrangement. *Angew. Chem. Int. Ed.* **52**, 9266–9270 (2013). 10.1002/anie.201303527 23852804

[51] Romanov-Michailidis, F., Guénée, L. & Alexakis, A. Enantioselective organocatalytic iodination-initiated Wagner-Meerwein rearrangement. *Org. Lett.* **15**, 5890–5893 (2013). 24195731 10.1021/ol402981z

[52] Chen, D.-F., Wu, P.-Y. & Gong, L.-Z. Rhodium/chiral urea relay catalysis enables an enantioselective semipinacol rearrangement/Michael addition cascade. *Org. Lett.* **15**, 3958–3961 (2013). 23886017 10.1021/ol4017386

[53] Yin, Q. & You, S.-L. Asymmetric chlorination/ring expansion for the synthesis of *α*-quaternary cycloalkanones. *Org. Lett.* **16**, 1810–1813 (2014). 24617607 10.1021/ol5005565

[54] Romanov-Michailidis, F., Pupier, M., Besnard, C., Bürgi, T. & Alexakis, A. Enantioselective catalytic fluorinative *aza*-semipinacol rearrangement. *Org. Lett.* **16**, 4988–4991 (2014). 25215413 10.1021/ol5022355

[55] Yang, B.-M. et al. Organocatalytic asymmetric tandem Nazarov cyclization/semipinacol rearrangement: Rapid construction of chiral spiro [4.4] nonane-1,6-diones. *J. Am. Chem. Soc.* **137**, 8344–8347 (2015). 26076169 10.1021/jacs.5b04049

[56] Wu, H., Wang, Q. & Zhu, J. Organocatalytic enantioselective vinylogous pinacol rearrangement enabled by chiral ion pairing. *Angew. Chem. Int. Ed.* **55**, 15411–15414 (2016). 10.1002/anie.201609911 27860238

[57] Yu, Y., Li, J., Jiang, L., Zhang, J.-R. & Zu, L. Catalytic enantioselective *aza*-pinacol rearrangement. *Angew. Chem. Int. Ed.* **56**, 9217–9221 (2017). 10.1002/anie.201705539 28618154

[58] Lukamto, D. H. & Gaunt, M. J. Enantioselective copper catalyzed arylation-driven semipinacol rearrangement of tertiary allylic alcohols with diaryliodonium salts. *J. Am. Chem. Soc.* **139**, 9160–9163 (2017). 28640609 10.1021/jacs.7b05340

[59] Liu, Y., Tse, Y.-L. S., Kwong, F. Y. & Yeung, Y.-Y. Accessing axially chiral biaryls *via* organocatalytic enantioselective dynamic kinetic resolution semipinacol rearrangement. *ACS Catal.* **7**, 4435–4440 (2017).

[60] Shim, S. Y., Choi, Y. & Ryu, D. H. Asymmetric synthesis of cyclobutanone *via* Lewis acid catalyzed tandem cyclopropanation/semipinacol rearrangement. *J. Am. Chem. Soc.* **140**, 11184–11188 (2018). 30089355 10.1021/jacs.8b06835

[61] Xie, Y.-Y. et al. Lewis base/Brønsted acid co-catalyzed enantioselective sulfenylation/semipinacol rearrangement of di- and trisubstituted allylic alcohols. *Angew. Chem. Int. Ed.* **58**, 12491–12496 (2019). 10.1002/anie.201907115 31293063

[62] Yang, J. et al. Enantioselective catalytic aldehyde *α*-alkylation/semipinacol rearrangement: Construction of *α*-quaternary-*δ*-carbonyl cycloketones and total synthesis of (+)-cerapicol. *Angew. Chem. Int. Ed.* **59**, 8471–8479 (2020). 10.1002/anie.202001100 32124524

[63] Gong, F. et al. Asymmetric semipinacol rearrangement enabled by copper-catalyzed propargylic alkylation. *ACS Catal.* **12**, 12036–12044 (2022).

[64] Côté, A. P. et al. Porous, crystalline, covalent organic frameworks. *Science* **310**, 1166–1170 (2005). 16293756 10.1126/science.1120411

[65] Feng, X., Ding, X. & Jiang, D. Covalent organic frameworks. *Chem. Soc. Rev.* **41**, 6010–6022 (2012). 22821129 10.1039/c2cs35157a

[66] Ding, S.-Y. & Wang, W. Covalent organic frameworks (COFs): From design to applications. *Chem. Soc. Rev.* **42**, 548–568 (2013). 23060270 10.1039/c2cs35072f

[67] Das, S., Heasman, P., Ben, T. & Qiu, S. Porous organic materials: Strategic design and structure-function correlation. *Chem. Rev.* **117**, 1515–1563 (2017). 28035812 10.1021/acs.chemrev.6b00439

[68] Rogge, S. M. J. et al. Metal–organic and covalent organic frameworks as single-site catalysts. *Chem. Soc. Rev.* **46**, 3134–3184 (2017). 28338128 10.1039/c7cs00033b PMC5708534

[69] Jin, Y., Hu, Y. & Zhang, W. Tessellated multiporous two-dimensional covalent organic frameworks. *Nat. Rev. Chem.* **1**, 56–67 (2017).

[70] Diercks, C. S. & Yaghi, O. M. The atom, the molecule, and the covalent organic framework. *Science* **355**, 923–931 (2017). 10.1126/science.aal1585 28254887

[71] Zhao, W., Xia, L. & Liu, X. Covalent organic frameworks (COFs): Perspectives of industrialization. *CrystEngComm* **20**, 1613–1634 (2018).

[72] Ding, S. Y. et al. Construction of covalent organic framework for catalysis: Pd/COF-LZU1 in Suzuki−Miyaura coupling reaction. *J. Am. Chem. Soc.* **133**, 19816–19822 (2011). 22026454 10.1021/ja206846p

[73] Xu, H., Gao, J. & Jiang, D. Stable, crystalline, porous, covalent organic frameworks as a platform for chiral organocatalysts. *Nat. Chem.* **7**, 905–912 (2015). 26492011 10.1038/nchem.2352

[74] Wang, X. et al. Homochiral 2D porous covalent organic frameworks for heterogeneous asymmetric catalysis. *J. Am. Chem. Soc.* **138**, 12332–12335 (2016). 27618953 10.1021/jacs.6b07714

[75] Pang, Z. F. et al. Construction of covalent organic frameworks bearing three different kinds of pores through the heterostructural mixed linker strategy. *J. Am. Chem. Soc.* **138**, 4710–4713 (2016). 27015785 10.1021/jacs.6b01244

[76] Wei, P.-F. et al. Benzoxazole-linked ultrastable covalent organic frameworks for photocatalysis. *J. Am. Chem. Soc.* **140**, 4623–4631 (2018). 29584421 10.1021/jacs.8b00571

[77] Kang, X. et al. Rational synthesis of interpenetrated 3D covalent organic frameworks for asymmetric photocatalysis. *Chem. Sci.* **11**, 1494–1502 (2020). 10.1039/c9sc04882k PMC8148036 34084378

[78] Wang, K. et al. Porous 2D and 3D covalent organic frameworks with dimensionality-dependent photocatalytic activity in promoting radical ring-opening polymerization. *Angew. Chem. Int. Ed.* **60**, 19466–19476 (2021). 10.1002/anie.202107915 34164891

[79] Wu, C.-J. Natural sunlight photocatalytic synthesis of benzoxazole-bridged covalent organic framework for photocatalysis. *J. Am. Chem. Soc.* **144**, 18750–18755 (2022). 36215722 10.1021/jacs.2c07893

[80] Wang, G.-B. et al. Construction of covalent organic frameworks via a visible-light-activated photocatalytic multicomponent reaction. *J. Am. Chem. Soc.* **145**, 4951–4956 (2023). 36847546 10.1021/jacs.2c13541

[81] Parvatkar, P. T. et al. A tailored COF for visible-light photosynthesis of 2,3-dihydrobenzofurans. *J. Am. Chem. Soc.* **145**, 5074–5082 (2023). 36827417 10.1021/jacs.2c10471 PMC9999419

[82] Guo, M. et al. The promotion effect of π-π interactions in Pd NPs catalysed selective hydrogenation. *Nat. Commun.* **13**, 1770–1779 (2022). 35365621 10.1038/s41467-022-29299-0 PMC8975908

[83] Gutierrez, L. et al. Low oxidation state cobalt center stabilized by a covalent organic framework to promote hydroboration of olefins. *ACS Catal.* **13**, 3044–3054 (2023).

[84] Fang, Y. et al. Design and synthesis of broadband absorption covalent organic framework for efficient artificial photocatalytic amine coupling. *Nat. Commun.* **15**, 4856–44865 (2024). 38849337 10.1038/s41467-024-49036-z PMC11161580

[85] Zhu, Y. et al. Sequential oxidation/cyclization of readily available imine linkages to access benzoxazole-linked covalent organic frameworks. *Angew. Chem. Int. Ed.* **63**, e202319909 (2024). 10.1002/anie.202319909 38243685

[86] Cheng, C. et al. Construction of benzoxazine-linked one-dimensional covalent organic frameworks using the Mannich reaction. *Angew. Chem. Int. Ed.* **63**, e202403473 (2024). 10.1002/anie.202403473 38829678

[87] Yang, M.-Y. et al. Three-motif molecular junction type covalent organic frameworks for efficient photocatalytic aerobic oxidation. *J. Am. Chem. Soc.* **146**, 3396–3404 (2024). 38266485 10.1021/jacs.3c12724

[88] Cheng, J. et al. Fully conjugated 2D sp^2^ carbon-linked covalent organic frameworks for photocatalytic overall water splitting. *Adv. Mater.* **36**, 2305313 (2024). 10.1002/adma.202305313 37818737

[89] He, H. et. al. Double enhancement of protonation and conjugation in donor–imine–donor covalent organic frameworks for photocatalytic hydrogen evolution. *Chem. Sci.* **15**, 20002–20012 (2024). 39568937 10.1039/d4sc07028c PMC11575607

[90] Gao, R. et. al. 2D/2D hydrogen-bonded organic frameworks/covalent organic frameworks S-scheme heterojunctions for photocatalytic hydrogen evolution. *Angew. Chem. Int. Ed.* **64**, e202414229 (2025). 10.1002/anie.202414229 39528399

[91] Prier, C. K., Rankic, D. A. & MacMillan, D. W. C. Visible light photoredox catalysis with transition metal complexes: Applications in organic synthesis. *Chem. Rev.* **113**, 5322–5363 (2013). 23509883 10.1021/cr300503r PMC4028850

[92] Luo, J. & Zhang, J. Donor-acceptor fluorophores for visible-light-promoted organic synthesis: Photoredox/Ni dual catalytic C(sp^3^)–C(sp^2^) cross-coupling. *ACS Catal.* **6**, 873–877 (2016).

[93] Frisch, M. J. et al. Gaussian 16 Rev. C.01, Wallingford, CT, 2016.

[94] Adamo, C. & Barone, V. Toward reliable density functional methods without adjustable parameters: The PBE0 model. *J. Chem. Phys.* **110**, 6158–6170 (1999).

[95] Ernzerhof, M. & Scuseria, G. E. Assessment of the Perdew−Burke−Ernzerh of exchange-correlation functional. *J. Chem. Phys.* **110**, 5029–5036 (1999). 10.1063/1.1634553 15268348

