# Author Correction: Straightforward construction of functionalized *γ*-lactams via conjugated-engineered covalent organic framework photocatalysed cascade reactions

**DOI:** 10.1038/s41467-026-77735-2

**Published:** 2026-09-17

**Authors:** Xiangfeng Lin, Jianguo Li, Jiaxian Zheng, Xiaowei Cai, Liwei Wang, Rongjian Sa, Chuanling Si, Dong Jiang, Yunqing Kang, Jie Wang, Yusuke Yamauchi, Zhanhui Yuan

**Affiliations:** 1 College of Materials Engineering, Fujian Agriculture and Forestry University, Fuzhou, P. R. China; 2 College of Materials and Chemical Engineering, Minjiang University, Fuzhou, P. R. China; 3 State Key Laboratory of Biobased Fiber Materials, Tianjin Key Laboratory of Pulp and Paper, Tianjin University of Science and Technology, Tianjin, P. R. China; 4 Department of Materials Process Engineering, Graduate School of Engineering, Nagoya University, Nagoya, Japan; 5 Australian Institute for Bioengineering and Nanotechnology (AIBN), The University of Queensland, Brisbane, QLD Australia; 6 Department of Chemical and Biomolecular Engineering, Yonsei University, Seoul, South Korea

**Keywords:** Organic molecules in materials science, Heterogeneous catalysis, Polymers

Correction to: *Nature Communications* https://doi.org/10.1038/s41467-025-66469-2, published online 1 December 2025

The initially published version of this article contained an error in Fig. 3 and in the first paragraph of the “Substrate scope” section, where for **3j**, the yield was wrongly reported as 56%. The correct yield is 30%. In the Supplementary Information section 27 the spirocyclic compound should have been numbered **12** and not **10**. On page S47 of the supplementary Information file the molecular formula for compound **3g** should have read C_24_H_21_Cl_2_N_2_O_3_P instead of C_24_H_21_F_2_N_2_O_3_P. The text, figure and Supplementary Information are now amended in the HTML and PDF versions of the article.

**Incorrect Figure 3**

**Figure:**
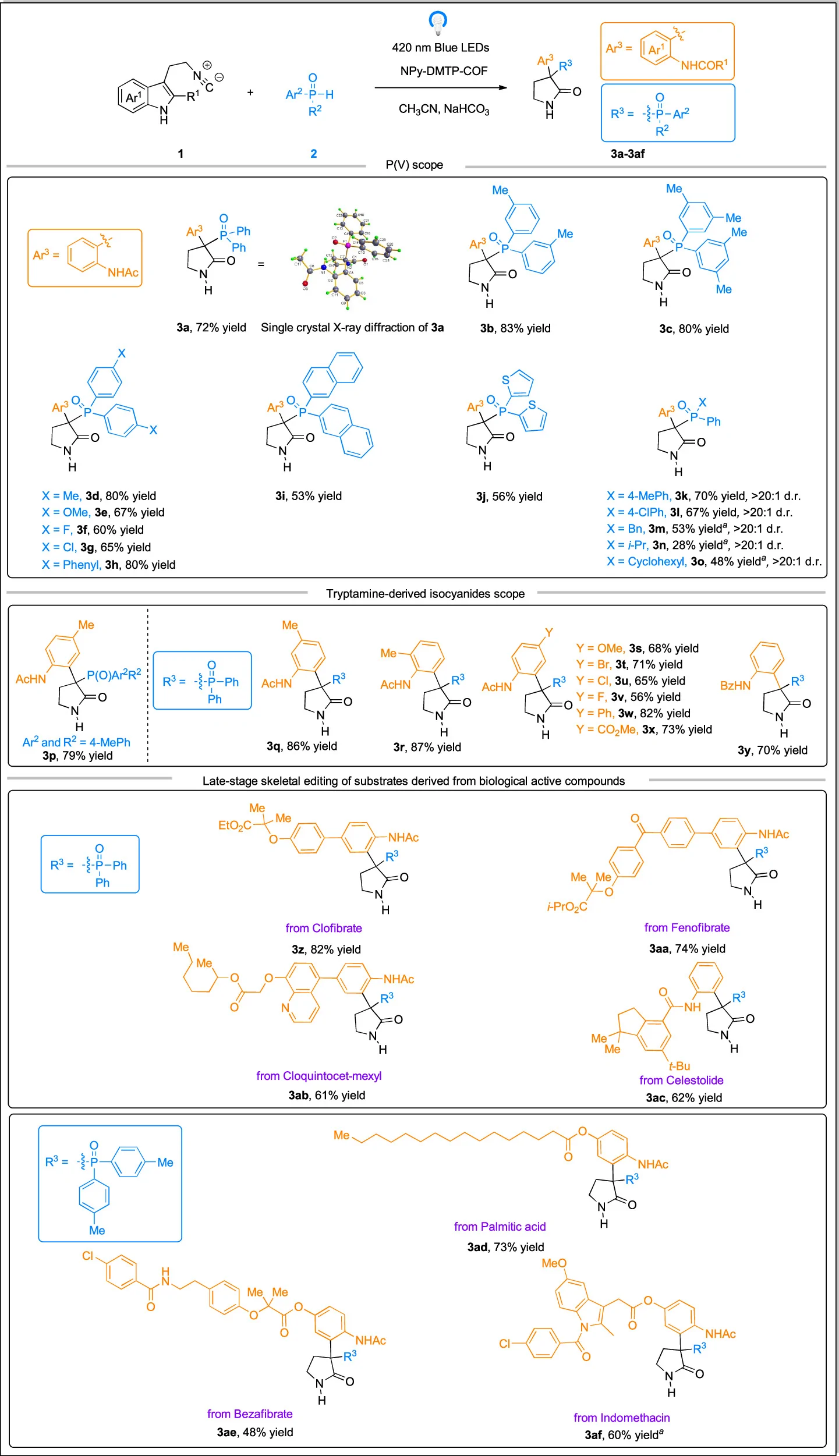

**Corrected Figure 3**

**Figure:**
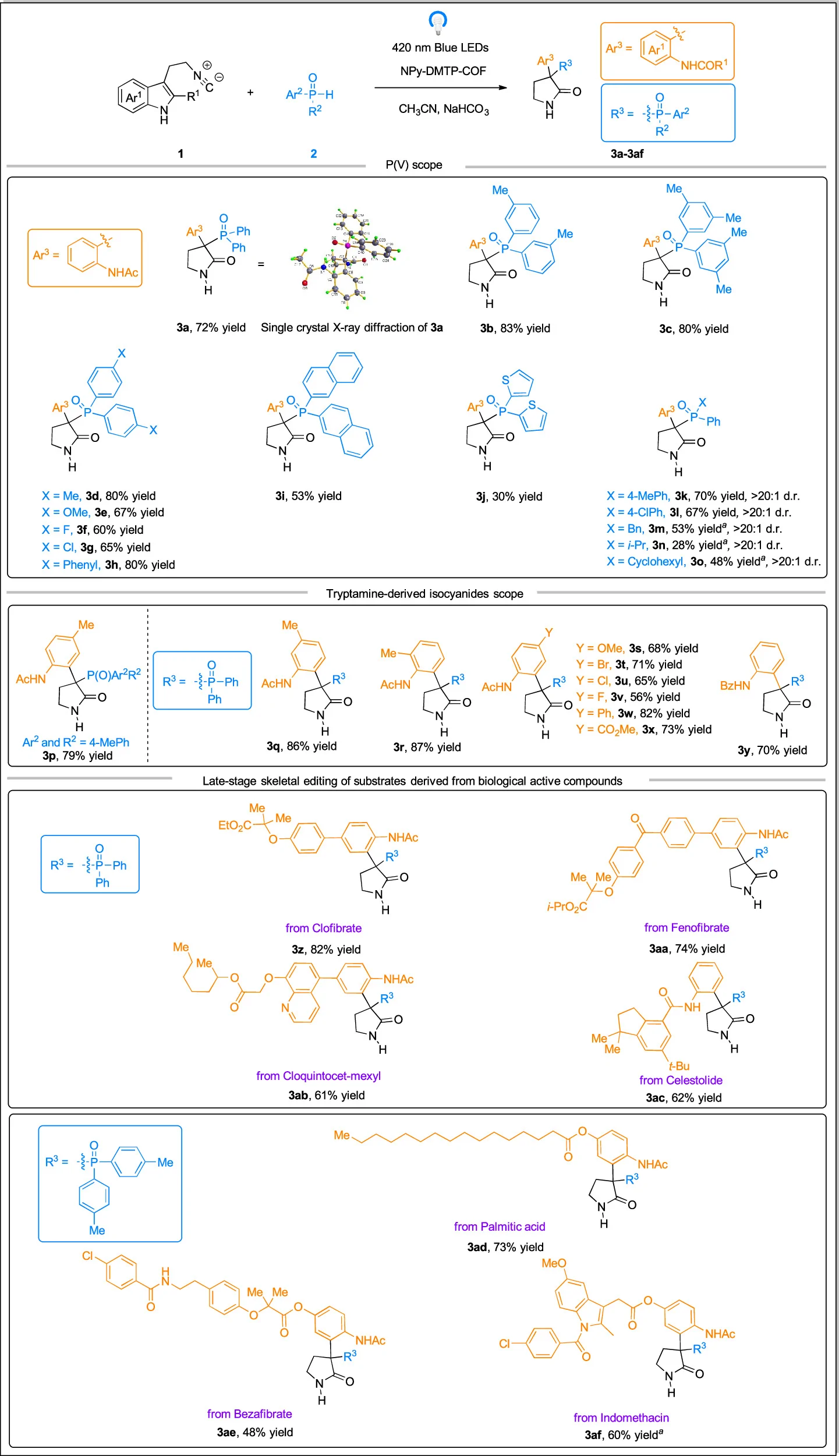

## Supplementary information

Supplementary Information

