# Supplementary material for: Author Correction: Straightforward construction of functionalized γ-lactams via conjugated-engineered covalent organic framework photocatalysed cascade reactions
Source: Nat Commun. 2026 Sep 17;17:9926. doi: 10.1038/s41467-026-77735-2 (PMC13586281; doi:10.1038/s41467-026-77735-2)
Supplement: Supplementary file 1 [file 41467_2026_77735_MOESM1_ESM.pdf]

Supplementary Information

Straightforward Construction of Functionalized γ-Lactams via Conjugated-engineered Covalent Organic Framework Photocatalysed Cascade Reactions

Xiangfeng Lin[#1], Jianguo Li[#1], Jiaxian Zheng[#1], Xiaowei Cai[1], Liwei Wang[2]*, Rongjian Sa[2]*, Chuanling Si[3]*, Dong Jiang[4,5]*, Yunqing Kang[4], Jie Wang[5], Yusuke Yamauchi[4,5,6] and Zhanhui Yuan[1]*

[1]College of Materials Engineering, Fujian Agriculture and Forestry University, Fuzhou 350002, P. R. China, *E-mail: zhanhuiyuan@fafu.edu.cn

[2]College of Materials and Chemical Engineering, Minjiang University, Fuzhou 350108, P. R. China, *E-mail: wlw@mju.edu.cn, rjsa@mju.edu.cn

[3]State Key Laboratory of Biobased Fiber Materials, Tianjin Key Laboratory of Pulp and Paper, Tianjin University of Science and Technology, Tianjin 300457, P. R. China, *E-mail: sichli@tust.edu.cn

[4]Department of Materials Process Engineering, Graduate School of Engineering, Nagoya University, Nagoya 464-8603, Japan, *E-mail: dongjiang@toki.waseda.jp

[5]Australian Institute for Bioengineering and Nanotechnology (AIBN), The University of Queensland, Brisbane, QLD 4072, Australia.

[6]Department of Chemical and Biomolecular Engineering, Yonsei University, Seoul, South Korea.

[#] These authors contributed equally.

Contents

1. Experimental procedures……………………………...…………………………………………………..……..S3

2. Characterizations of NPy-DMTP-COF……………………………………………..….……………....….S9

3. Mechanistic studies………………………………………………………………………………...…..S19

4. Spectral data of products ………………………………………………………………………..…......S33

5. X-ray crystallographic data of **3a**.………………..……………………………………………………..…...S71

6. NMR spectra ……………………………………………………………………………………………..S81

7. Supplementary references...S203

1. Experimental procedures

1.1 Synthesis of 6,6',6'',6'''-(pyrene-1,3,6,8-tetrayl)tetrakis(naphthalen-2-amine) (NPy)

Under a nitrogen atmosphere, 1,3,6,8-tetrabromopyrene (513.7 mg, 1.0 mmol), 6-(4,4,5,5-tetramethyl-1,3,2-dioxaborolan-2-yl)naphthalen-2-amine (1.07 g, 4.0 mmol), $Pd(PPh_3)_4$ (115.4 mg, 0.1 mmol) and potassium carbonate (607.2 mg, 4.4 mmol) were added to 5.0 mL dioxane and 1.0 mL water in a 50 mL round bottom flask. The mixture was stirred under reflux for 3 days. After cooling to room temperature, 30 mL of water were added. The precipitate was filtered, washed with water and methanol. The solid was dissolved in acetone and filtered through a short silica gel plug. The solvent was removed to afford the product with a yield of 72% (551.5 mg).

1.2 Synthesis of NPy-DMTP-COF

6,6',6'',6'''-(pyrene-1,3,6,8-tetrayl)tetrakis(naphthalen-2-amine) (NPy) (38.3 mg, 0.05 mmol) and 2,5-dimethoxyterephthalaldehyde (DMTP) (19.4 mg, 0.1 mmol) and CH_3COOH (0.1 mL, 6 M) were mixed in a mixture of solvents containing 0.25 mL of *o*-dichlorobenzene and 0.75 mL of 1-butanol in a seal tube. The mixture was ultrasonicated for 10 min and then flash frozen at 77 K (liquid N_2 bath) and degassed through three freeze-pump-thaw cycles. The mixture was then heated at 120 °C for 72 h under an N_2 atmosphere. The resulting solid product was recovered by filtration, washed with methanol and DCM, and subjected to Soxhlet extraction using THF for 24 hours. After drying under vacuum for 6 hours, a brown solid was obtained with a yield of 87%. This material was designated as NPy-DMTP-COF.

1.3 Synthesis of model compound of NPy-DMTP-COF (NPy-MP)

NPy-MP was synthesized according to the Bein's methods[1]. To a suspension of NPy (38.3 mg, 0.05 mmol) in $CHCl_3$ (2 mL) were added 2-methoxybenzaldehyde (54.4 mg, 0.40 mmol) and anhydrous $MgSO_4$ (100 mg). The vessel was sealed and stirred at 70 °C overnight. After cooling, the mixture was filtered through a Celite pad and the filtrate was concentrated under reduced pressure. Hexane (10 mL) was added to the residue to precipitate the product. The yellow solid was collected by filtration, washed with DCM/hexane (1 : 5) and dried under vacuum for 6 h to obtain NPy-MP as a yellow solid in 74 % yield (45.7 mg).

1.4 General experimental procedure of substrates 1 and 4

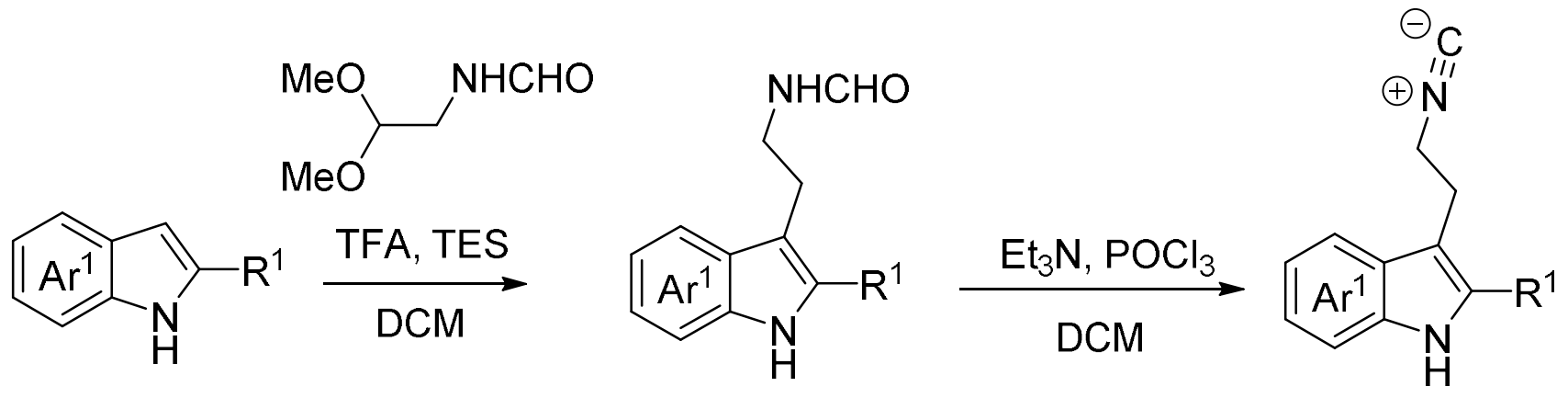

To a solution of triethylsilane (TES, 4.8 mL, 30 mmol) and trifluoroacetic acid (TFA, 3.7 mL, 50 mmol) in DCM (0.5 M) was added a solution of dimethyl acetal (11 mmol) and indole (10 mmol) in DCM (0.5 M) dropwise at room temperature. The reaction mixture was stirred for 16 hours and quenched at 0 °C with saturated $NaHCO_3$ solution. The aqueous layer was extracted three times with DCM. The organic layers were collected and washed with brine, dried over Na_2SO_4 and concentrated in vacuum without further purifications.

Next, the formamide was dissolved in anhydrous DCM (0.5 M). Subsequently triethylamine (6.9 mL, 50 mmol) and phosphoryl chloride (1.4 mL, 15 mmol) were respectively dropwise added at -78 °C. After mixing for 3 h at this temperature, the reaction was quenched by addition of the crude mixture in ice-cold water. The product was extracted three times with DCM, washed with water and brine, dried over sodium sulfate and concentrated. The mixture was purified by silica gel flash chromatography (petroleum ether : ethyl acetate, 3 : 1) to afford **1**.

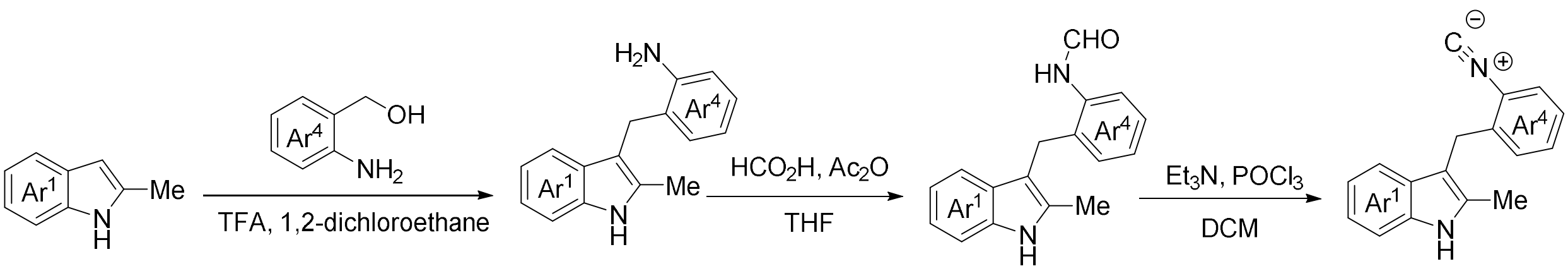

To a solution of (2-aminophenyl)methanol (20 mmol) and indole (24 mmol) in 1,2-dichloroethane (40 mL) was added TFA (0.59 mL, 8 mmol) was added dropwise, then the mixture was heated with heating mantle and stirred at 50 °C. When the reaction was complete as monitored by TLC, aqueous solution of saturated Na_2CO_3 was added, concentrated under reduced pressure, dilute hydrochloric acid was added, and the mixture was extracted with dichloromethane three times. The combined organic layer was dried over anhydrous Na_2SO_4, filtered, and evaporated, and the mixture was purified by silica gel flash chromatography (petroleum ether : ethyl acetate, 3 : 1) to afford 2-((2-methyl-1H-indol-3-yl)methyl)aniline.

A mixture of HCO_2H (1.3 mL, 35 mmol), Ac_2O (1.4 mL, 15 mmol) was stirred at 50 °C for 30 min. To this mixture, a solution of 2-((2-methyl-1H-indol-3-yl)methyl)aniline (10 mmol) in THF was added slowly. After 2 h, the reaction was quenched by adding saturated aqueous $NaHCO_3$. The organic layer was separated, and the

aqueous layer was extracted with DCM. The combined organic phase was washed with brine, dried over $MgSO_4$. The concentrated residue was used for next step without further purification. The product was dissolved in dry DCM (30 mL) and Et_3N (6.9 mL, 50 mmol), cooled to -20 °C. To mixture $POCl_3$ (1.4 mL, 15 mmol) was added, dropwise slowly. The reaction mixture was stirred for 2 h. Then, 10 mL of H_2O was added slowly to the reaction mixture at -20 °C. The crude reaction mixture was extracted with DCM and washed with brine. The organic phase was concentrated in vacuum and the mixture was purified by silica gel flash chromatography (petroleum ether : ethyl acetate, 3 : 1) to afford **4**.

1.5 General experimental procedure of photocatalyzed cascade reactions

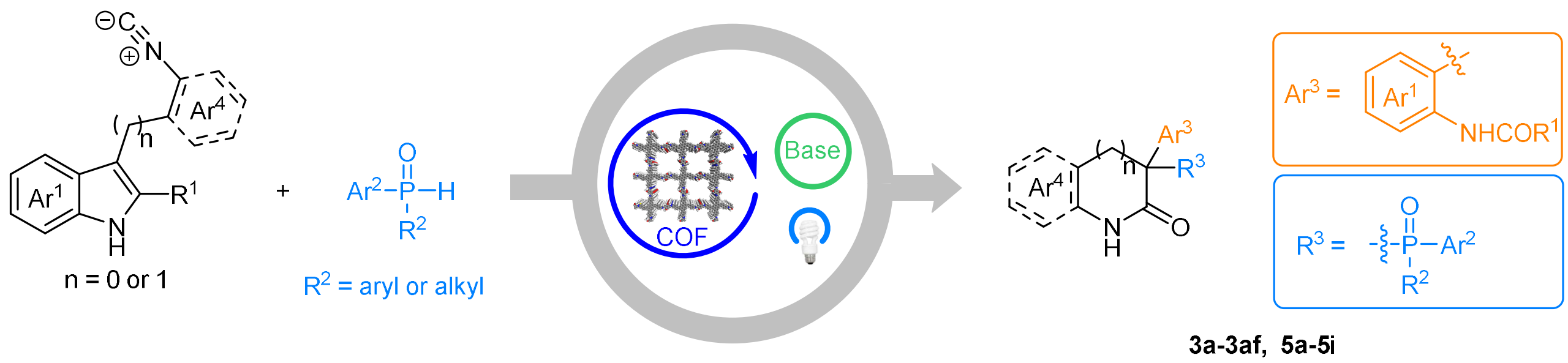

Under a nitrogen atmosphere, NPy-DMTP-COF (5.3 mg, 0.006 mmol based on the repeating unit), **1** or **4** (0.3 mmol), **2** (0.6 mmol) and $NaHCO_3$ (50.4 mg, 0.6 mmol) or Na_2HPO_4 (85.2 mg, 0.6 mmol) were mixed in acetonitrile (4.0 mL) in a Schlenk tube. The resulting mixture was stirred under blue LED irradiation (420 nm) at room temperature for 48-96 hours. Upon completion, the residual was filtered through celite pad and filtrate was concentrated. The mixture was purified by silica gel flash chromatography (petroleum ether : ethyl acetate, 1 : 3) to afford the desired product **3** or **5**.

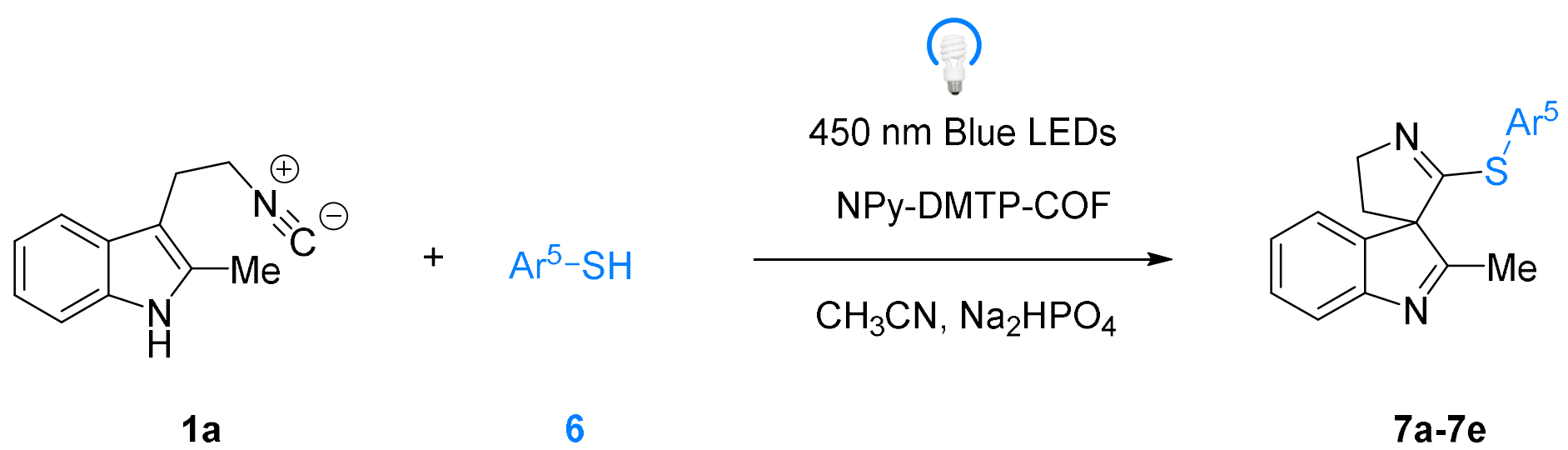

Under a nitrogen atmosphere, NPy-DMTP-COF (5.3 mg, 0.006 mmol based on the repeating unit), **1a** (0.3 mmol, 55.2 mg), **6** (0.6 mmol) and Na_2HPO_4 (85.2 mg, 0.6 mmol) were mixed in acetonitrile (4.0 mL) in a Schlenk tube. The resulting mixture was stirred under blue LED irradiation (450 nm) at room temperature for 48-96 hours. Upon completion, the residual was filtered through celite pad and filtrate was concentrated.

The mixture was purified by silica gel flash chromatography (petroleum ether : ethyl acetate, 2 : 1) to afford the desired product **7**.

1.6 Gram scale reaction

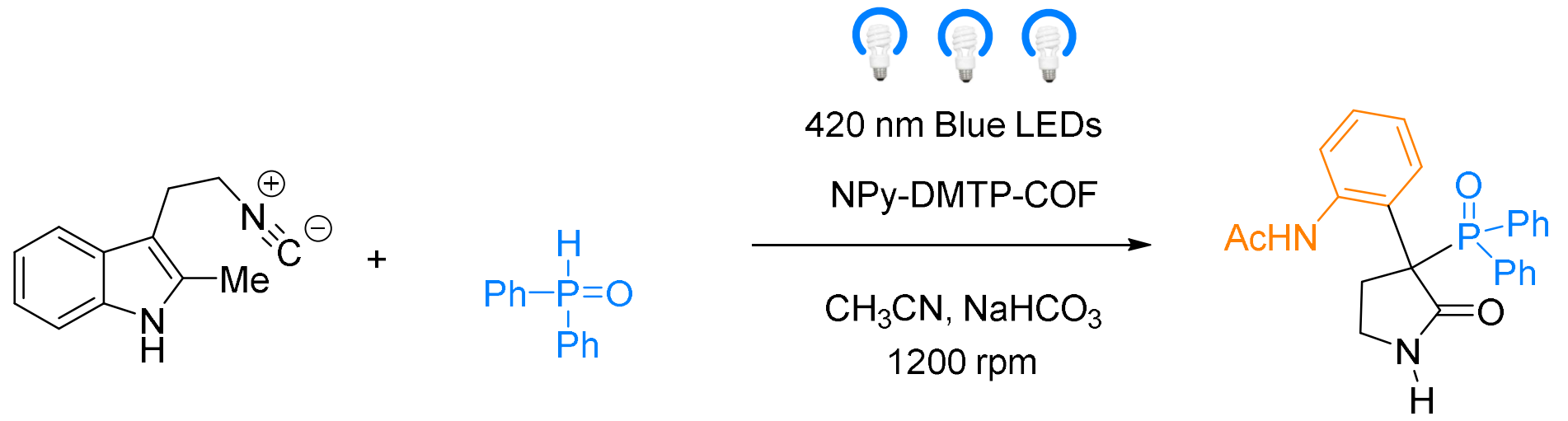

1a, 1.0 g, 5.43 mmol **2a** **3a,** 1.58 g, 70% yield

Under a nitrogen atmosphere, NPy-DMTP-COF (194.3 mg, 0.22 mmol based on the repeating unit), **1a** (1.0 g, 5.43 mmol), **2a** (2.18 g, 10.86 mmol) and $NaHCO_3$ (912.2 mg, 10.86 mmol) were mixed in acetonitrile (40.0 mL) in a Schlenk tube. The resulting mixture was stirred at 1200 rpm under three blue LED lamps (420 nm) at room temperature for 120 hours. Upon completion, the residual was filtered through celite pad and filtrate was concentrated. The mixture was purified by silica gel flash chromatography (petroleum ether : ethyl acetate, 1 : 3) to afford the desired product **3a** with a yield of 70% (1.58 g).

1.7 Synthesis of compound 8

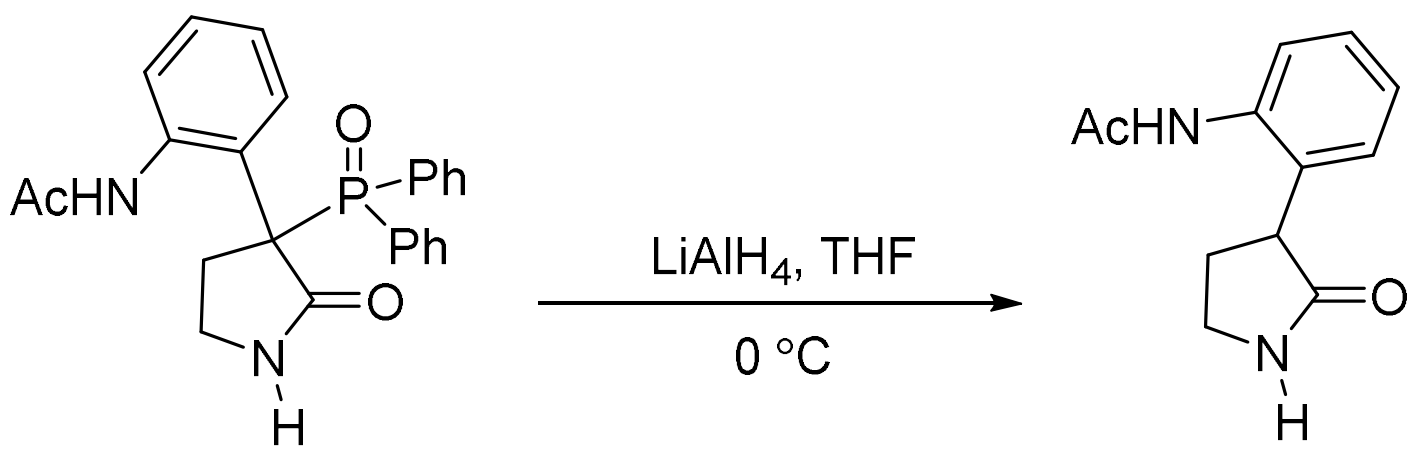

3a **8**

Under a nitrogen atmosphere, **3a** (62.7 mg, 0.15 mmol) was dissolved in anhydrous tetrahydrofuran (3.0 mL). Subsequently, $LiAlH_4$ in THF at a concentration of 1.0 M (0.9 mL, 3.0 eq.) was added at 0 °C. After 12 hours, the reaction was terminated by the addition of 1.0 mL of a 10% aqueous NaOH solution. The mixture was then desiccated using $MgSO_4$. The residual was filtered through celite pad and filtrate was concentrated. The mixture was purified by silica gel flash chromatography (ethyl acetate : methanol, 10 : 1) to afford the desired product **8** with a yield of 56% (18.3 mg).

1.8 Synthesis of compound 9

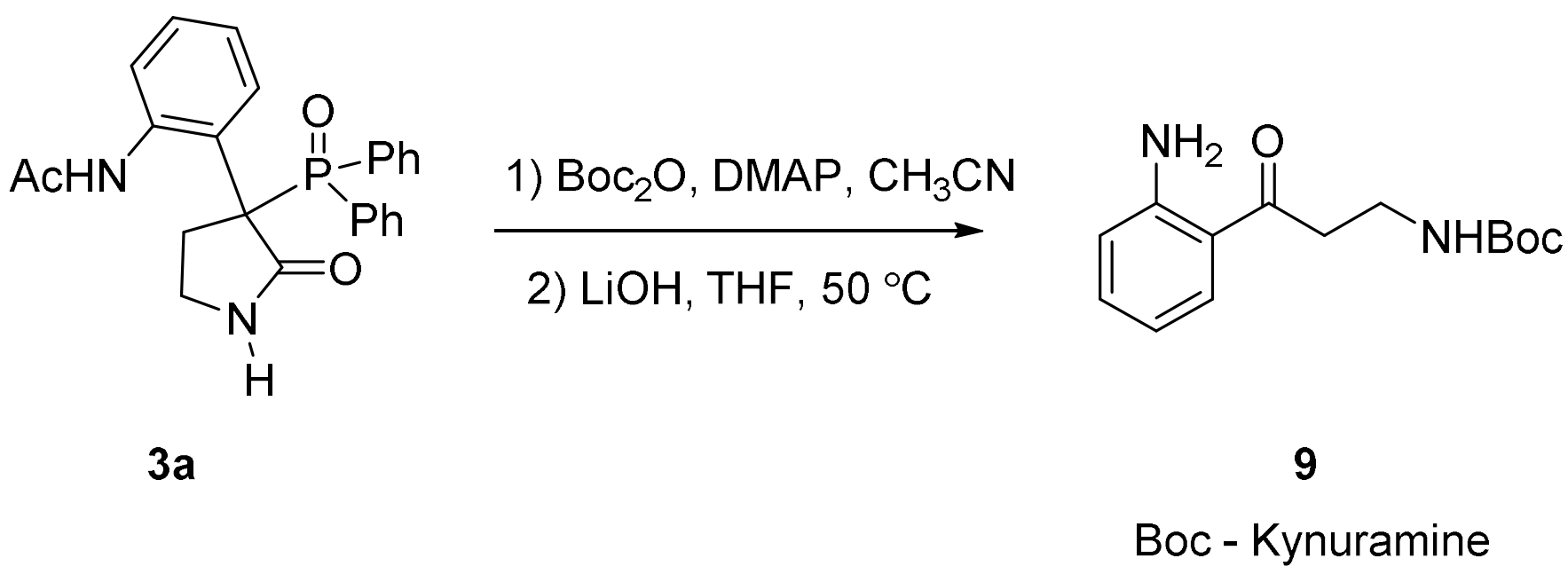

A solution of **3a** (83.6 mg, 0.20 mmol), DMAP (6.1 mg, 0.05 mmol) and Boc_2O (87.3 mg, 0.40 mmol) were dissolved in 2.0 mL CH_3CN. The mixture was stirred at room temperature for 12 h. Upon completion, the solvent was removed under reduced pressure, and the resulting mixture was purified by column chromatography on silica gel (petroleum ether : ethyl acetate, 5 : 1) to give Boc protected pyrrolidinone as a colorless oil.

To a solution of Boc protected pyrrolidinone in 3.0 mL THF was added 1 M aq. LiOH (2.0 mL, 2.0 mmol), and the resulting solution was stirred at 50 °C for 5 h. Then the aqueous layer was acidified with 1 M HCl and extracted with DCM three times. The combined organic layer was dried over Na_2SO_4, filtered, and concentrated under reduced pressure to give a residue. The mixture was purified by silica gel flash chromatography (petroleum ether : ethyl acetate, 3 : 1) to afford the desired product **9**. Total yield is 45% (23.6 mg).

1.9 Synthesis of compound 10 and 11

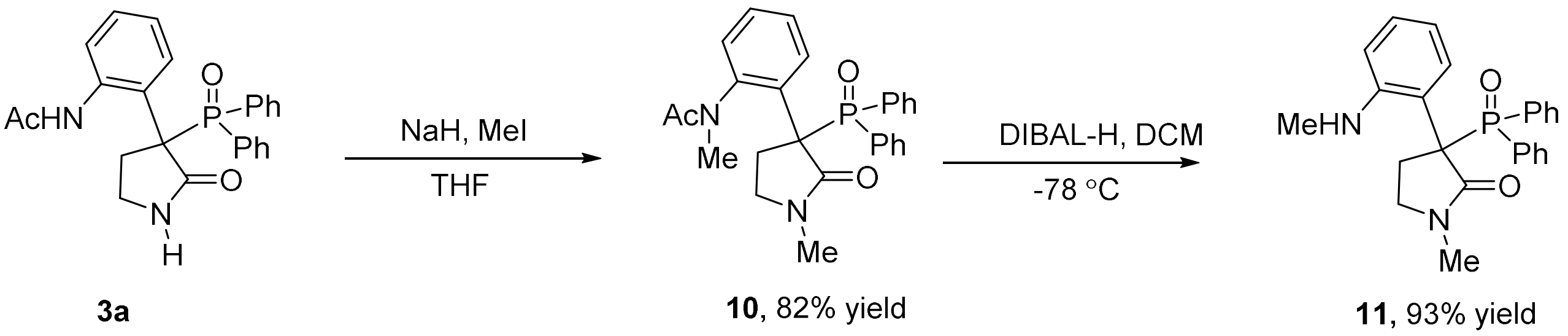

Under a nitrogen atmosphere, **3a** (62.7 mg, 0.15 mmol) and NaH (24.0 mg, 0.6 mmol, 60% dispersion in mineral oil) were dissolved in anhydrous tetrahydrofuran (3.0 mL) at 0 °C. Subsequently, MeI (85.2 mg, 0.6 mmol) was added and the mixture was stirred at 0 °C for 2 hours. Upon completion, the reaction was quenched by 2.0 mL H_2O and extracted with ethyl acetate three times. The combined organic layer was dried over Na_2SO_4, filtered, and concentrated under reduced pressure to give a residue. The mixture was purified by silica gel flash chromatography (petroleum ether : ethyl acetate, 1 : 1) to afford the desired product **10** with a yield of 82% (54.8 mg).

Under a nitrogen atmosphere, **3a** (54.8 mg, 0.12 mmol) was dissolved in anhydrous tetrahydrofuran (3.0 mL) at -78 °C. Subsequently, DIBAL-H (85.2 mg, 0.6 mmol) in toluene at a concentration of 1.0 M (0.36 mL, 3.0 eq.) was added and the mixture was stirred at -78 °C for 2 hours. Upon completion, the reaction was quenched by 2.0 mL H_2O and extracted with ethyl acetate three times. The combined organic layer was dried over Na_2SO_4, filtered, and concentrated under reduced pressure to give a residue. The mixture was purified by silica gel flash chromatography (petroleum ether : ethyl acetate, 5 : 1) to afford the desired product **11** with a yield of 93% (46.2 mg).

2. Characterizations of NPy-DMTP-COF

Supplementary Table 1 Fractional atomic coordinated for unit cell of NPy-DMTP-COF (sql topology) calculated using the Materials Studio modeling program after performing the Pawley Refinement.

Space group	P1		
Calculated cell parameters	a = 26.9 Å, b = 3.8 Å, c = 27.0 Å, α = 90°, β = 90°, γ = 90°, R_{wp} = 5.00% and R_p = 2.73%		
Atoms	x	y	z
C1	0.94833	0.00000	0.18327
C2	0.92516	0.00000	0.26180
C3	0.96025	0.00000	0.23030
C4	0.00792	0.00000	0.24603
C5	0.01996	0.00000	0.29308
C6	0.98489	0.00000	0.32474
C7	0.93739	0.00000	0.30885
N8	0.99839	0.00000	0.37272
C9	0.96824	0.00000	0.40438
C10	0.98297	0.00000	0.45354
C11	0.03125	0.00000	0.46547
C12	0.04928	0.00000	0.51094
C13	0.95716	0.00000	0.78554
C14	0.96955	0.00000	0.83254
C15	0.01683	0.00000	0.84911
C16	0.03112	0.00000	0.89949
C17	0.17301	0.00000	0.00775
C18	0.24334	0.00000	0.05820
C19	0.22189	0.00000	0.01400
C20	0.24968	0.00000	0.97551
C21	0.29859	0.00000	0.98173
C22	0.32019	0.00000	0.02623
C23	0.29233	0.00000	0.06422

N24	0.37023	0.00000	0.03378
C25	0.39859	0.00000	0.00084
C26	0.45061	0.00000	0.00536
C27	0.46932	0.00000	0.96219
C28	0.51730	0.00000	0.95496
C29	0.77185	0.00000	0.06858
C30	0.82083	0.00000	0.07442
C31	0.84919	0.00000	0.03643
C32	0.90150	0.00000	0.04386
C33	0.07941	0.00000	0.91114
C34	0.05167	0.00000	0.81673
C35	0.07484	0.00000	0.73820
C36	0.03975	0.00000	0.76970
C37	0.99208	0.00000	0.75397
C38	0.98004	0.00000	0.70692
C39	0.01511	0.00000	0.67526
C40	0.06261	0.00000	0.69115
N41	0.00161	0.00000	0.62728
C42	0.03176	0.00000	0.59562
C43	0.01703	0.00000	0.54646
C44	0.96875	0.00000	0.53453
C45	0.95072	0.00000	0.48906
C46	0.04284	0.00000	0.21446
C47	0.03045	0.00000	0.16746
C48	0.98317	0.00000	0.15089
C49	0.96888	0.00000	0.10051
C50	0.82699	0.00000	0.99225
C51	0.75666	0.00000	0.94180
C52	0.77811	0.00000	0.98600
C53	0.75032	0.00000	0.02449

C54	0.70141	0.00000	0.01827
C55	0.67981	0.00000	0.97377
C56	0.70767	0.00000	0.93578
N57	0.62977	0.00000	0.96622
C58	0.60141	0.00000	0.99916
C59	0.54939	0.00000	0.99464
C60	0.53068	0.00000	0.03781
C61	0.48270	0.00000	0.04504
C62	0.22815	0.00000	0.93142
C63	0.17917	0.00000	0.92558
C64	0.15081	0.00000	0.96357
C65	0.09850	0.00000	0.95614
C66	0.92059	0.00000	0.08886
C67	0.98387	0.00000	0.01783
C68	0.00071	0.00000	0.06461
C69	0.93505	0.00000	0.00646
C70	0.04929	0.00000	0.07429
C71	0.08068	0.00000	0.03966
O72	0.09878	0.00000	0.51664
C73	0.12054	0.00000	0.56163
O74	0.47353	0.00000	0.09205
C75	0.42729	0.00000	0.10688
C76	0.01613	0.00000	0.98217
C77	0.99929	0.00000	0.93539
C78	0.06495	0.00000	0.99354
C79	0.95071	0.00000	0.92571
C80	0.91932	0.00000	0.96034
O81	0.90122	0.00000	0.48336
C82	0.87946	0.00000	0.43837
O83	0.52647	0.00000	0.90795

C84	0.57271	0.00000	0.89312
H85	0.90981	0.00000	0.17044
H86	0.88660	0.00000	0.24913
H87	0.05850	0.00000	0.30583
H88	0.90858	0.00000	0.33441
H89	0.92929	0.00000	0.39316
H90	0.05761	0.00000	0.43749
H91	0.91854	0.00000	0.77307
H92	0.94104	0.00000	0.85843
H93	0.15045	0.00000	0.03879
H94	0.22091	0.00000	0.08934
H95	0.32112	0.00000	0.95066
H96	0.30956	0.00000	0.10043
H97	0.37971	0.00000	-0.03465
H98	0.44491	0.00000	0.92988
H99	0.74958	0.00000	0.09983
H100	0.83852	0.00000	0.11044
H101	0.10354	0.00000	0.88127
H102	0.09019	0.00000	0.82956
H103	0.11340	0.00000	0.75087
H104	0.94150	0.00000	0.69417
H105	0.09142	0.00000	0.66559
H106	0.07071	0.00000	0.60684
H107	0.94239	0.00000	0.56251
H108	0.08146	0.00000	0.22693
H109	0.05896	0.00000	0.14157
H110	0.84955	0.00000	0.96121
H111	0.77909	0.00000	0.91066
H112	0.67888	0.00000	0.04934
H113	0.69044	0.00000	0.89957

H114	0.62029	0.00000	1.03465
H115	0.55509	0.00000	0.07012
H116	0.25042	0.00000	0.90017
H117	0.16148	0.00000	0.88956
H118	0.89646	0.00000	0.11873
H119	0.06291	0.00000	0.11177
H120	0.11996	0.00000	0.04944
H121	0.13342	-0.30095	0.57072
H122	0.09405	0.09516	0.58718
H123	0.15162	0.20579	0.56311
H124	0.41241	-0.30275	0.10463
H125	0.40447	0.19875	0.08407
H126	0.42759	0.10400	0.14393
H127	0.93709	0.00000	0.88823
H128	0.88004	0.00000	0.95056
H129	0.84132	0.09516	0.43984
H130	0.88069	-0.30095	0.42338
H131	0.89889	0.20579	0.41577
H132	0.57482	0.19875	0.86232
H133	0.58276	-0.30275	0.88288
H134	0.59794	0.10400	0.92218

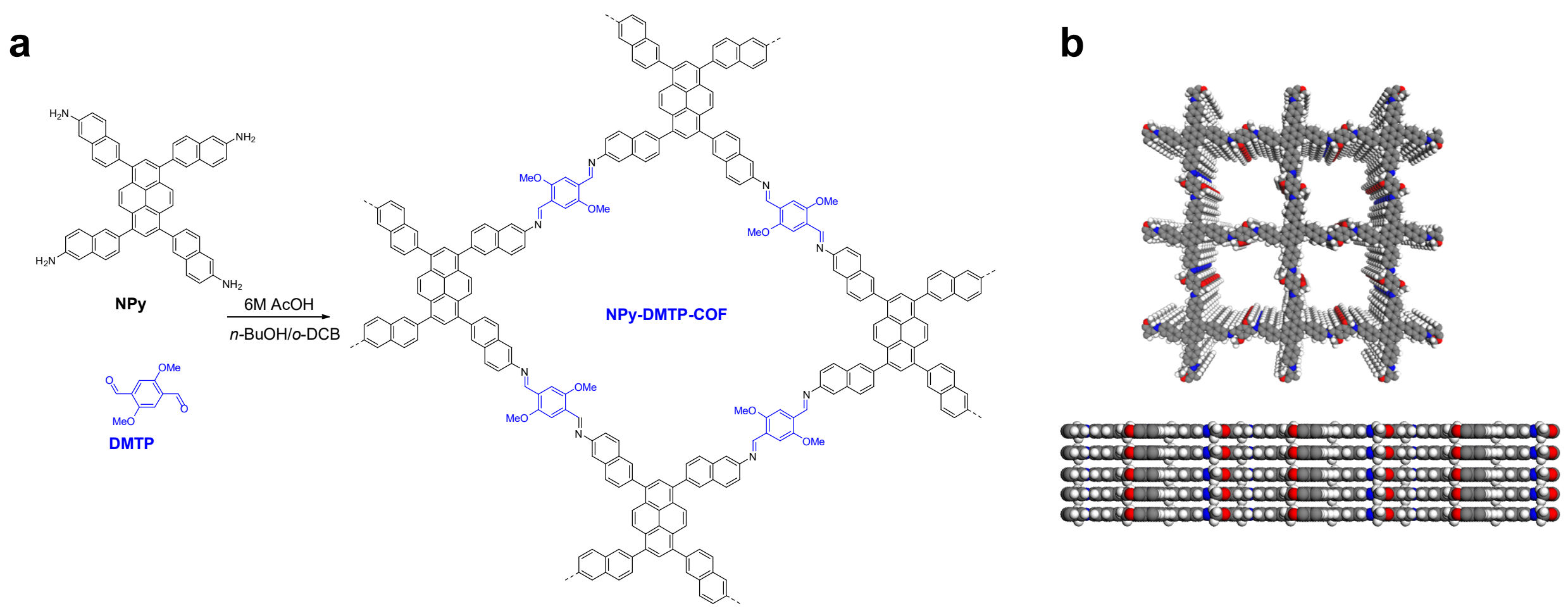

Supplementary Fig. 1 Design and synthesis of NPy-DMTP-COF. (a) The synthesis of NPy-DMTP-COF. (b) Graphic view of NPy-DMTP-COF simulated by Material Studio. (White, H; red, O; blue, N; grey, C). AcOH is acetic acid. *n*-BuOH is *n*-butanol. *o*-DCB is *o*-dichlorobenzene.

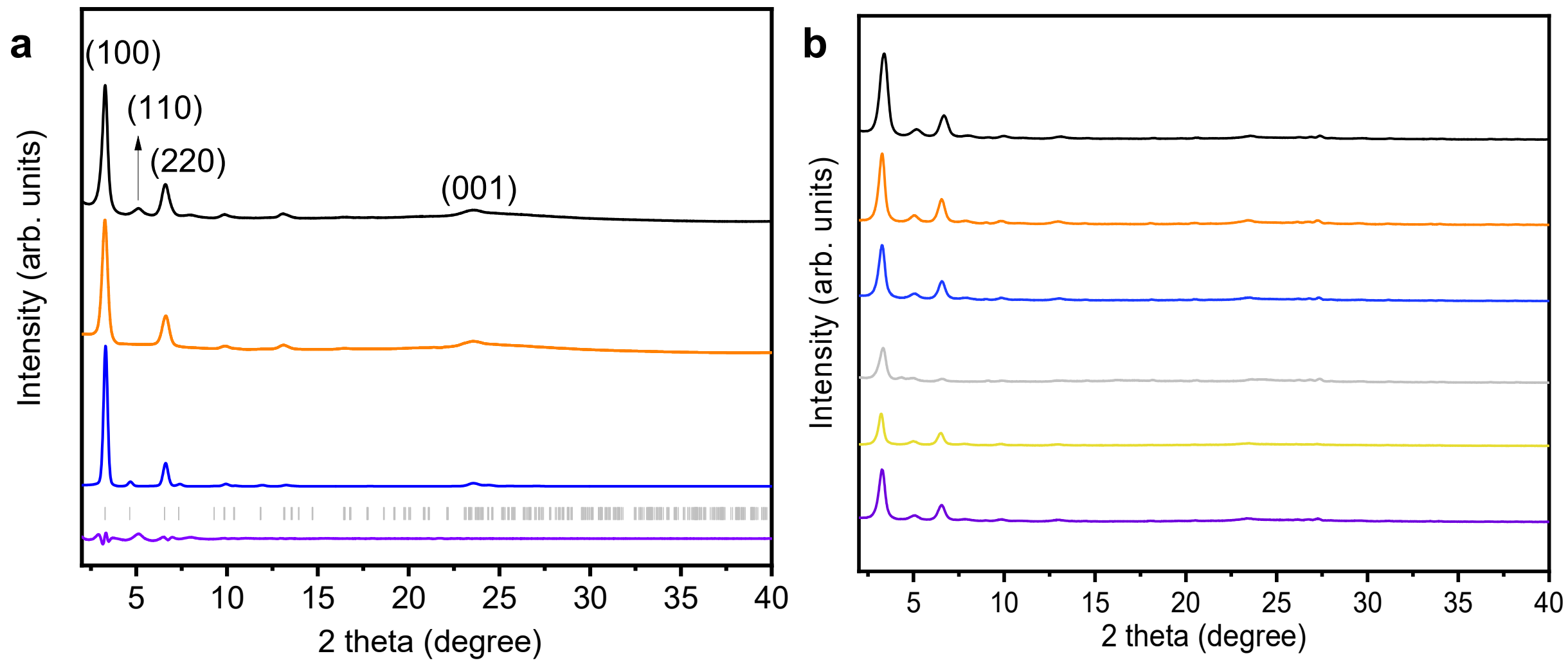

Supplementary Fig. 2 PXRD patterns of NPy-DMTP-COF. (a) The experimental data (black), the Pawley refined profiles (orange), the calculated patterns of the eclipsed AA stacking (blue), the Bragg diffractions (grey) and the refinement differences (purple). arb. units = arbitrary units. (b) PXRD patterns of NPy-DMTP-COF before (black) and after treatment in 14 N NaOH (orange), triethylamine (blue), Conc. HCl (grey), trifluoroacetic acid (yellow) and boiling water (purple) for 3 days.

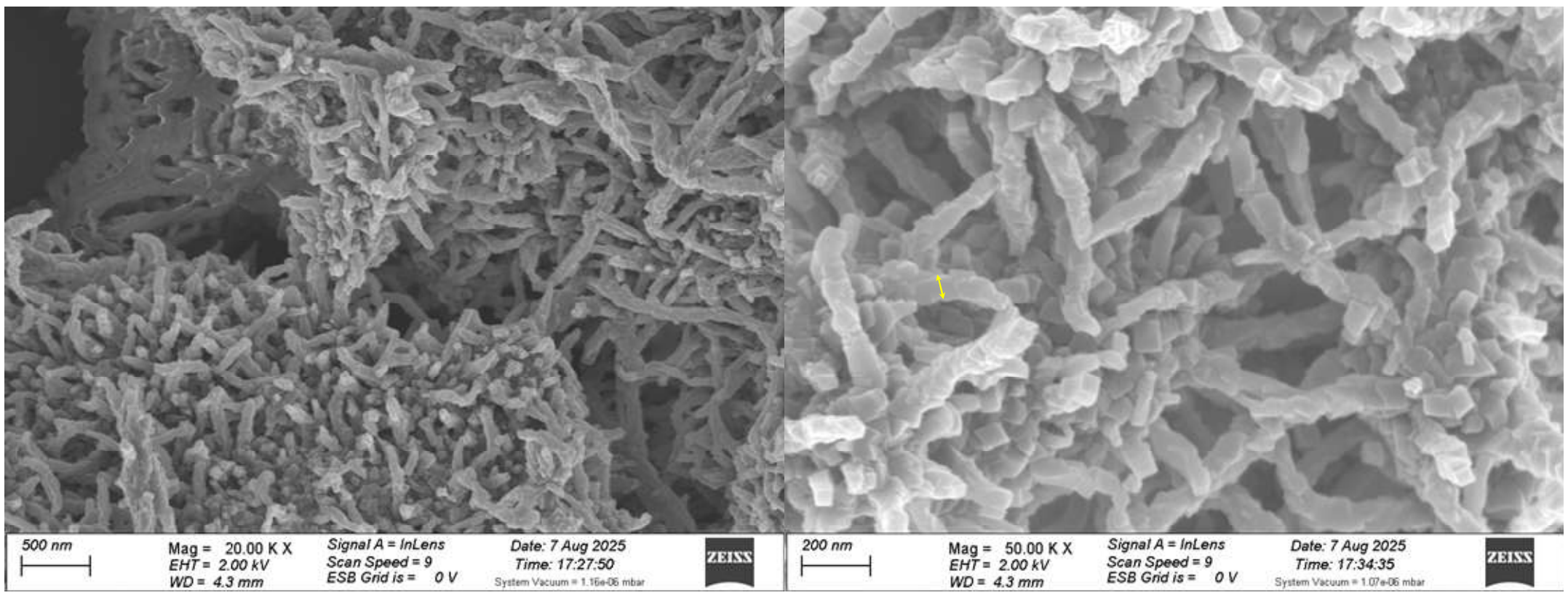

Supplementary Fig. 3 SEM images of NPy-DMTP-COF. The diameter of ~ 65 nm is indicated by the yellow outline. Scale bars, 500 nm (left) and 200 nm (right).

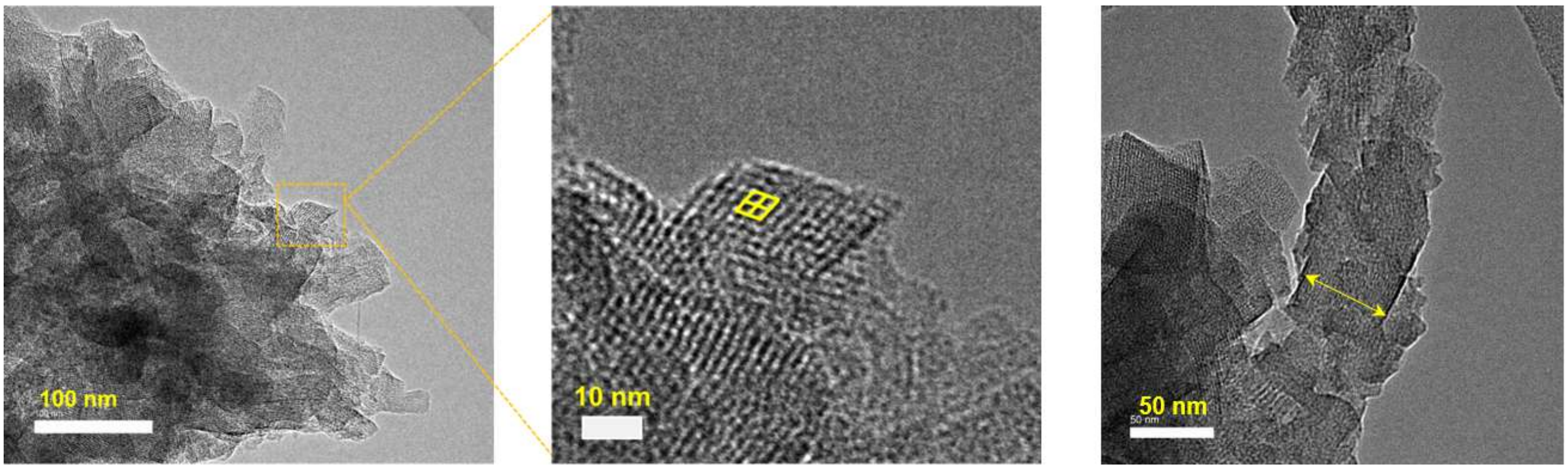

Supplementary Fig. 4 HR-TEM images of NPy-DMTP-COF. The quadrilateral pore structure with a periodicity of ~ 2.6 nm (middle) and a diameter of ~ 60 nm (right) is indicated by the yellow outline. Scale bars, 100 nm (left), 10 nm (middle) and 50 nm (right).

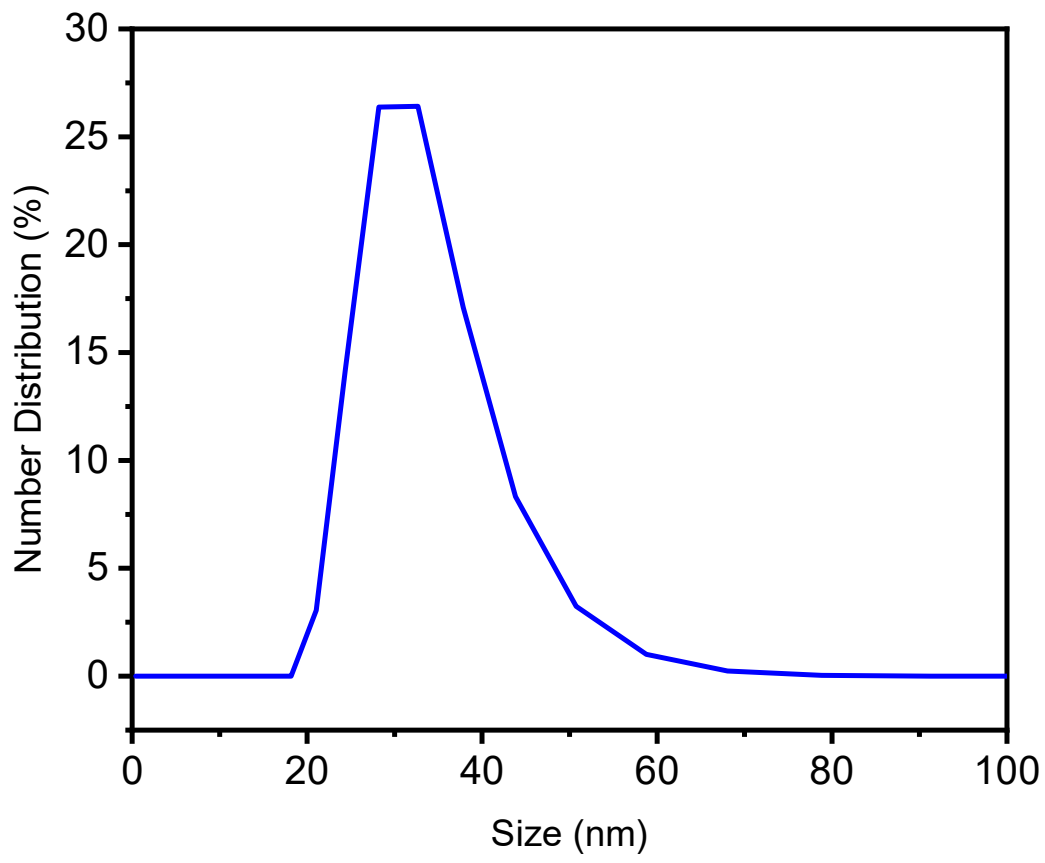

Supplementary Fig. 5 DLS number distributions (Z-Average is 41.05 nm, PDI is 0.115).

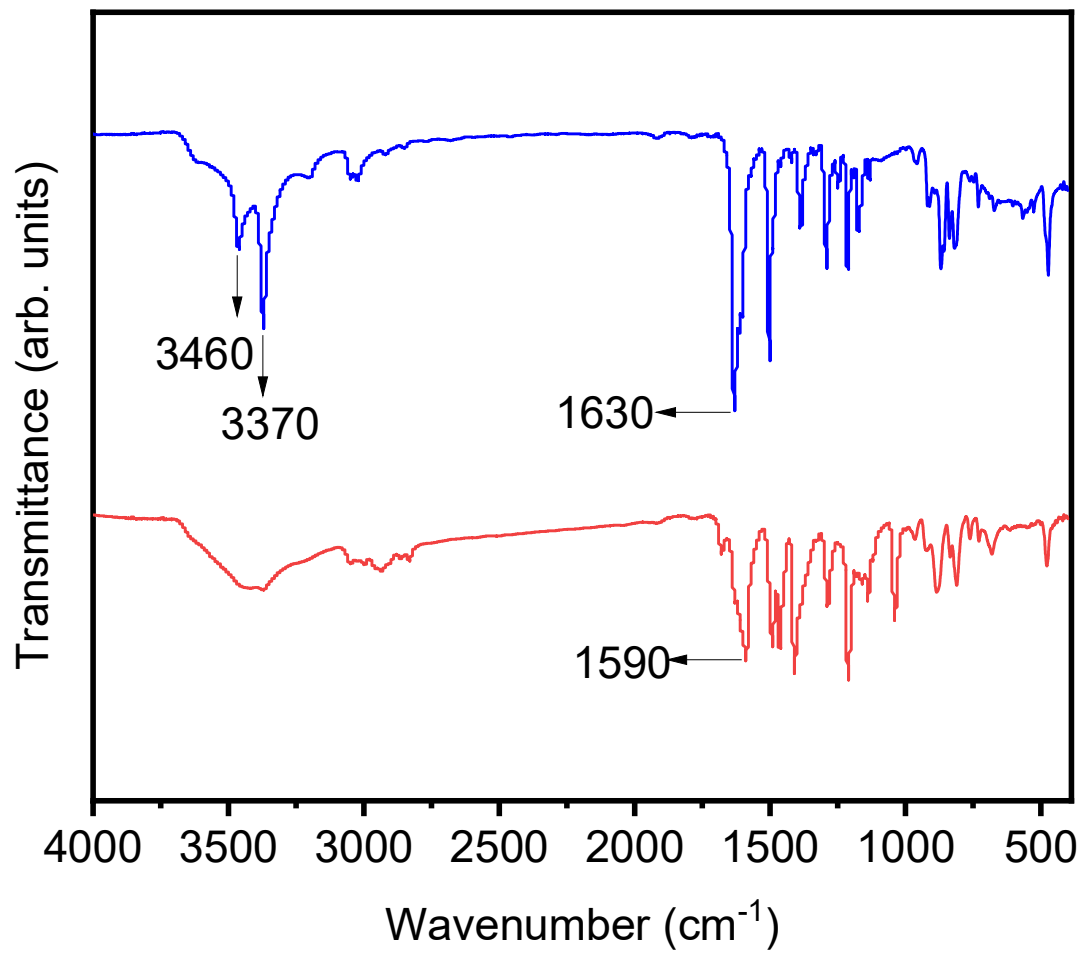

Supplementary Fig. 6 FT-IR spectrum of NPy (blue line) and NPy-DMTP-COF (red line). arb. units = arbitrary units.

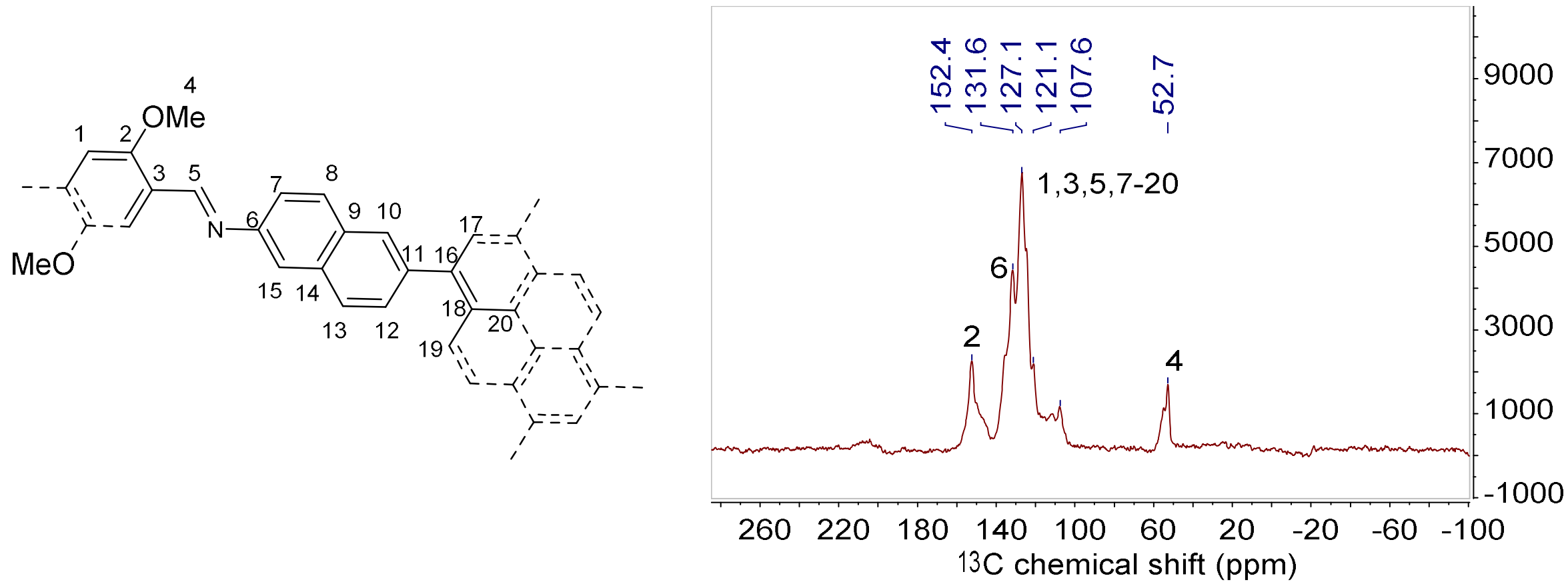

Supplementary Fig. 7 ^{13}C-CP/TOSS NMR spectrum of NPy-DMTP-COF.

The BET surface area was calculated from the adsorption data in a relative partial pressure (P/P_0) range of 1.00 to 0.00. The total pore volume was calculated at P/P_0 of 0.99 using a single-point adsorption value. The pore diameter was determined from the adsorption branch by using the Quenched Solid Density Functional Theory (QSDFT) method.

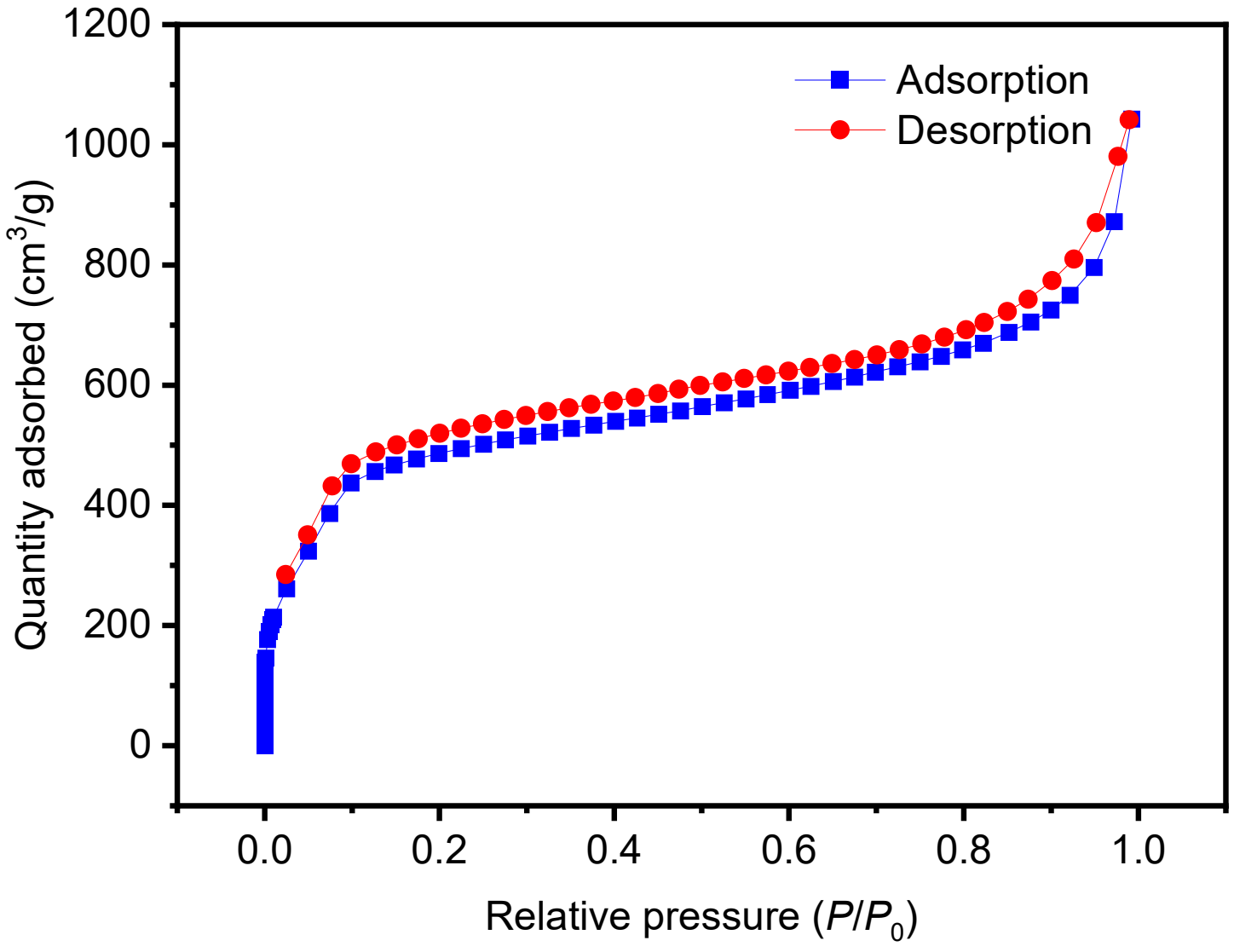

Supplementary Fig. 8 Nitrogen adsorption (blue line) and desorption (red line) isotherm at 77 K of NPy-DMTP-COF.

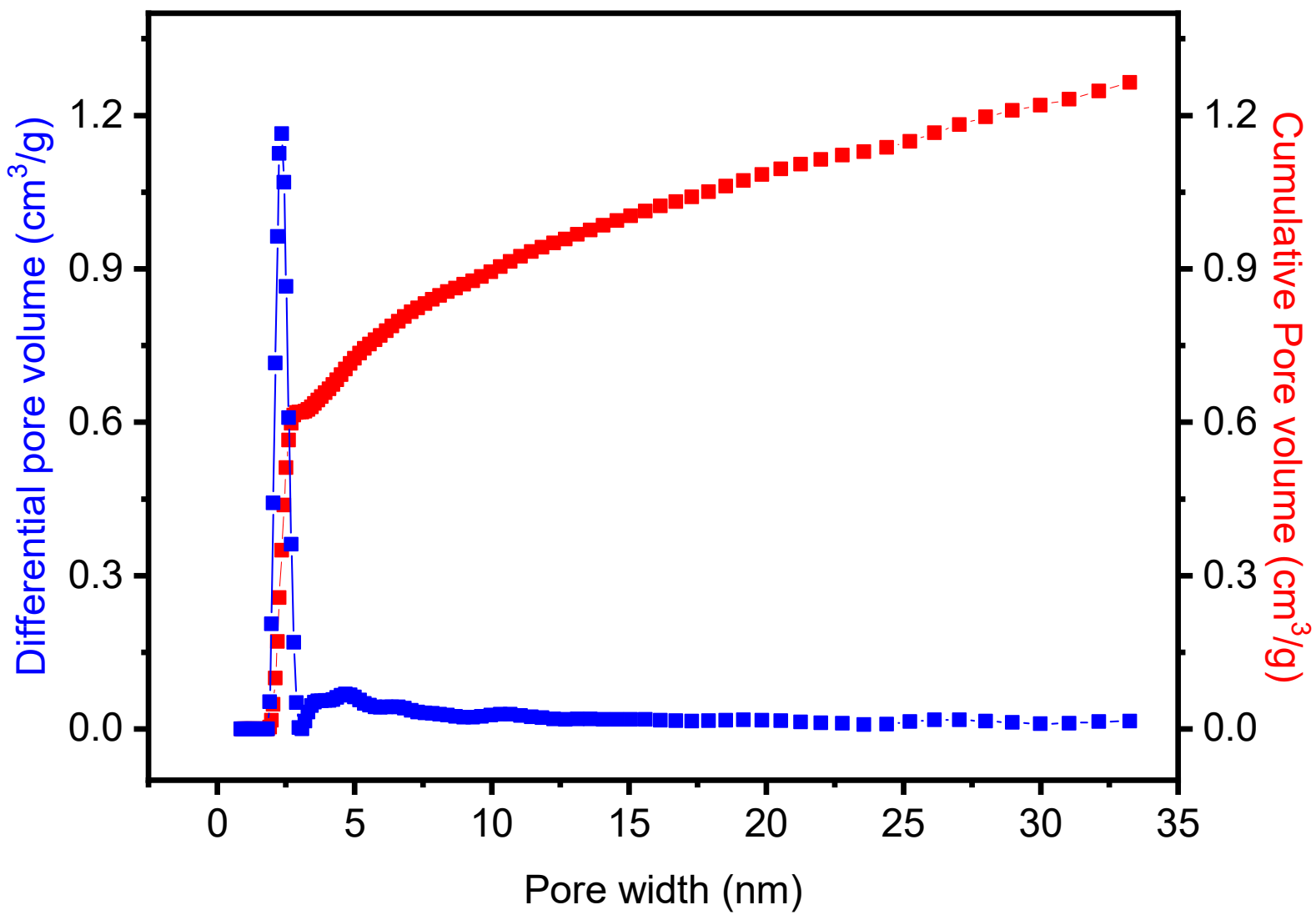

Supplementary Fig. 9 Pore size distribution of NPy-DMTP-COF.

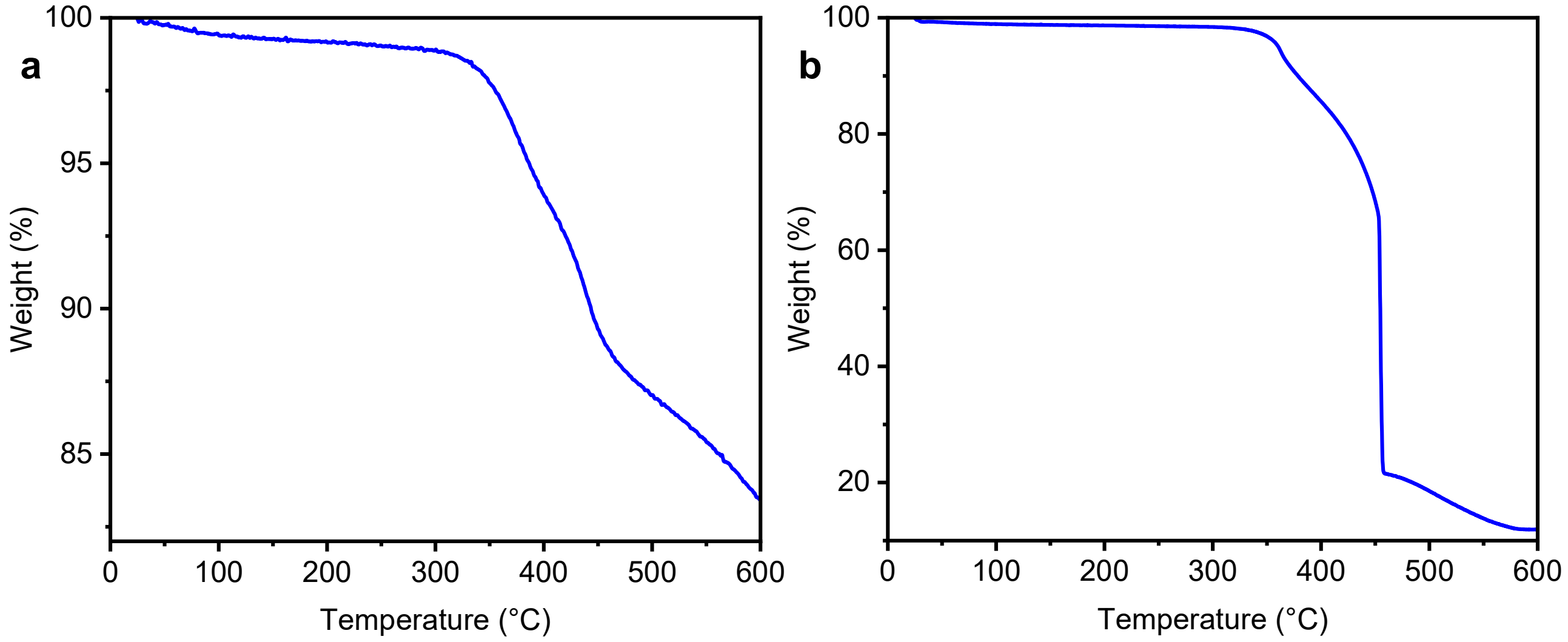

Supplementary Fig. 10 Thermogravimetric analysis of NPy-DMTP-COF. TGA were performed with a temperature range from 30 to 600 °C and a heating rate of 10 K/min in nitrogen (a) or oxygen (b) atmosphere.

3. Mechanistic studies

3.1 Spectroscopic characterization

UV-vis diffuse reflectance spectrophotometry (DRS) was recorded using Shimadzu UV-2600 UV-visible spectrum with $BaSO_4$ as a reference. Steady-state photoluminescence (PL) spectra were measured on a LS55 spectrophotometer (Perkin-Elmer, USA) at room temperature. The excitation energy change (E^{0-0}) was estimated by the point of intersection of the normalized absorption and emission spectra.

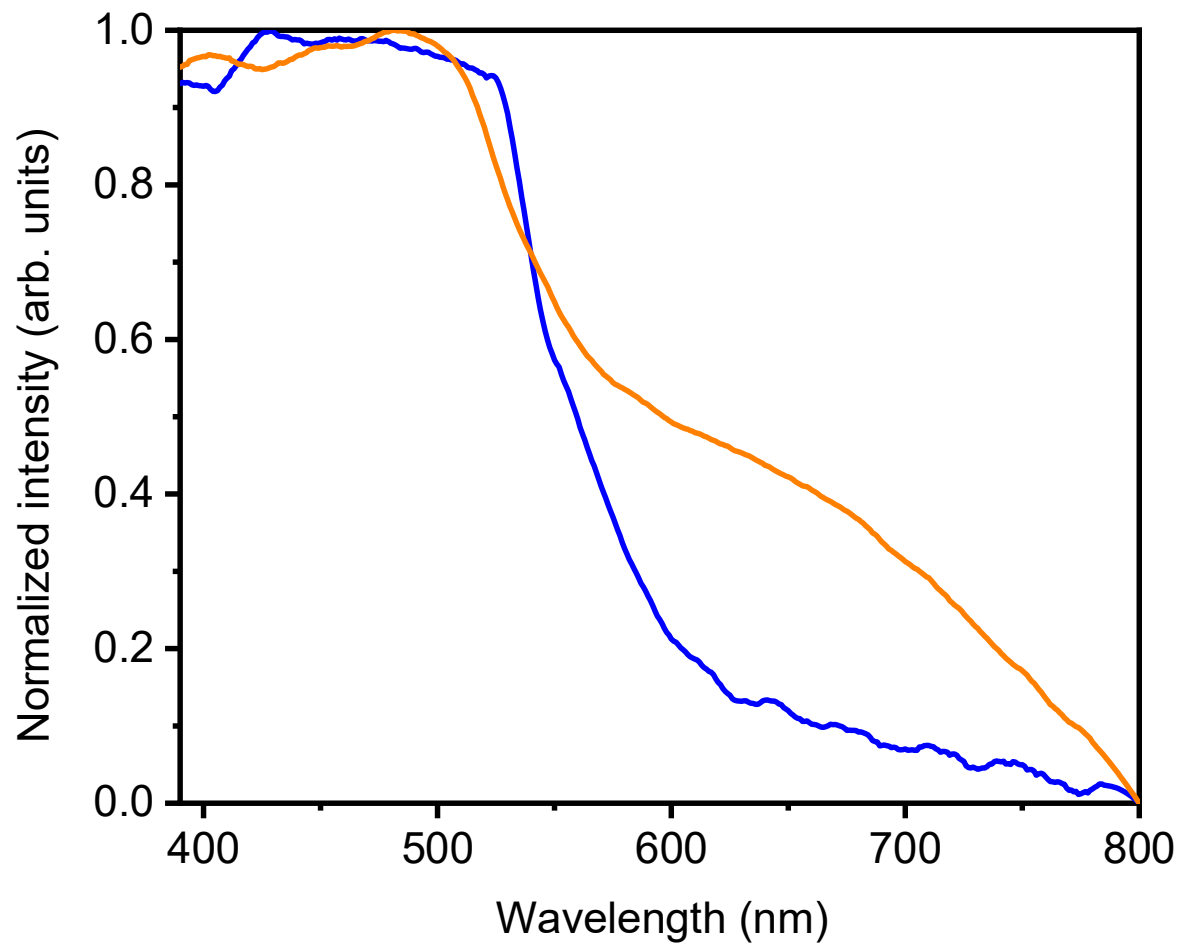

Supplementary Fig. 11 Normalized absorption spectra of NPy-DMTP-COF (orange line) and Py-DMTP-COF (blue line). arb. units = arbitrary units.

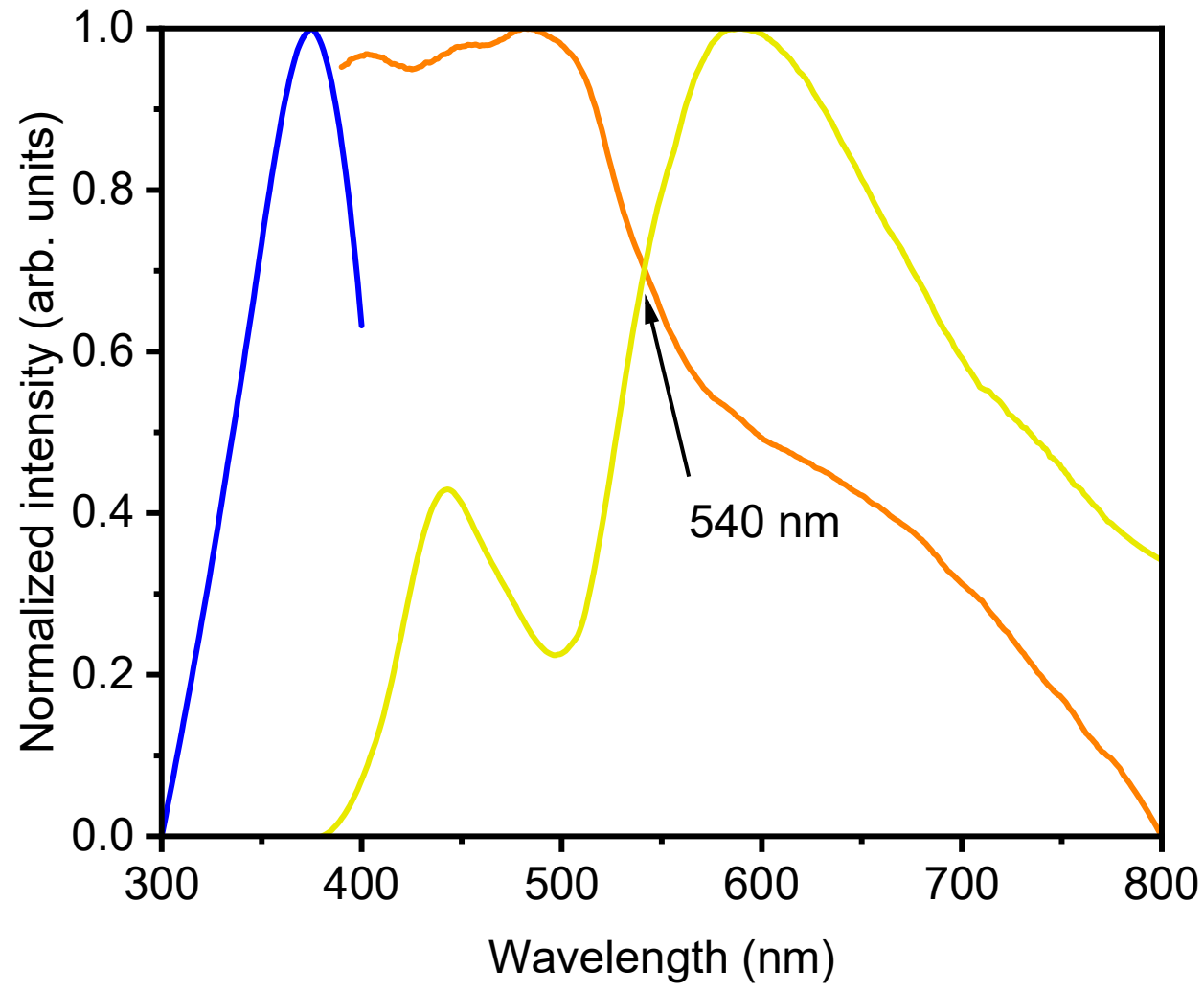

Supplementary Fig. 12 Normalized absorption (orange), excitation (blue), and emission (yellow) spectra of NPy-DMTP-COF. arb. units = arbitrary units.

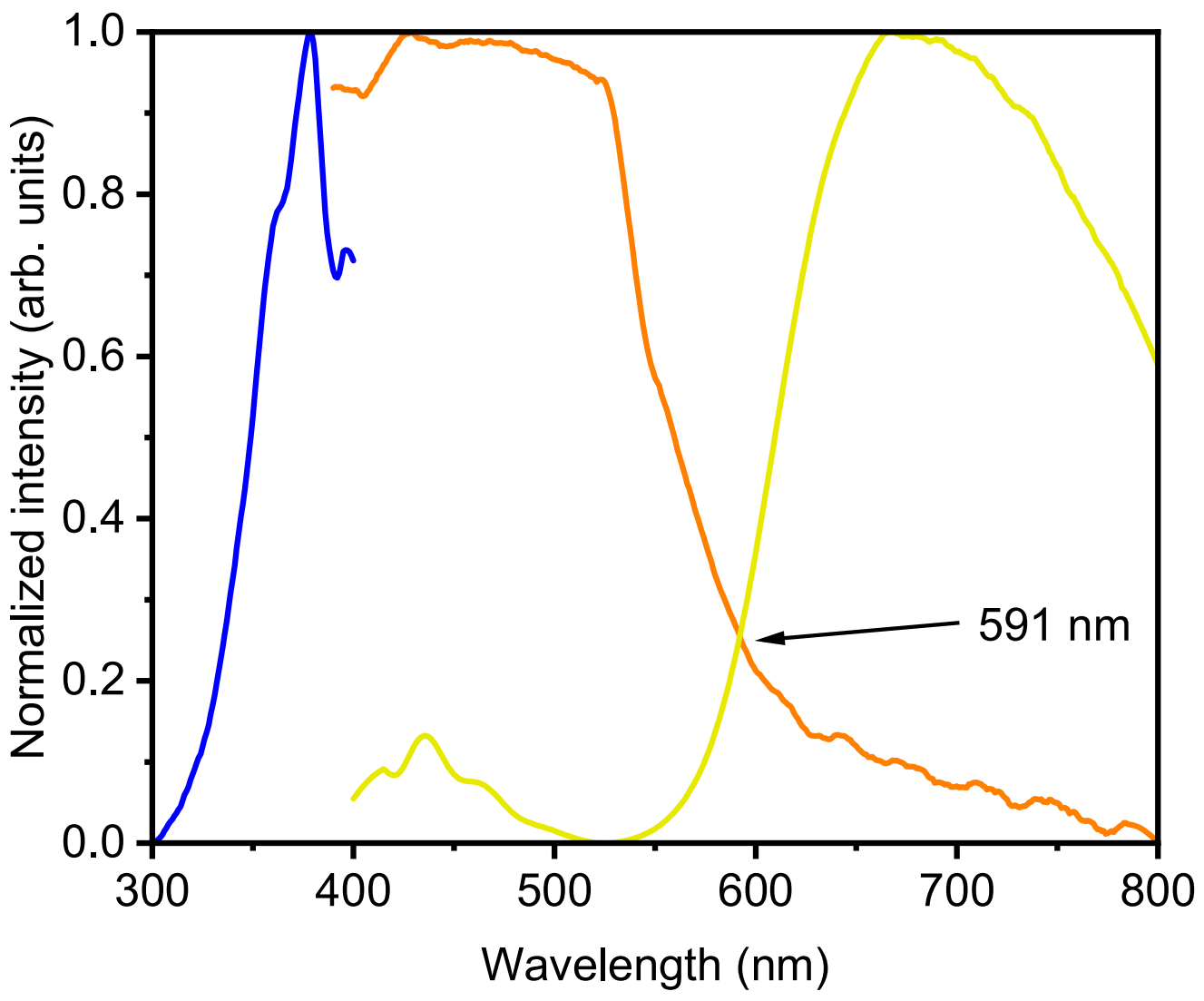

Supplementary Fig. 13 Normalized absorption (orange), excitation (blue), and emission (yellow) spectra of Py-DMTP-COF. arb. units = arbitrary units.

3.2 Cyclic voltammetry

A standard three-electrode cell configuration was used to collect cyclic voltammograms at room temperature with a Chi600e electrochemical workstation. Every electrolyte solution contains 0.1 M tetrabutylammonium hexafluorophosphate ($TBAPF_6$).

To determine the cyclic voltammetry curve for NPy-DMTP-COF. The 5 mg as-prepared materials were dispersed in a mixture of ethanol (2 mL) and Nafion solution (0.5 mL) by sonication and then casted onto the glassy carbon disc electrode surface. NPy-DMTP-COF had a reduction potential at -0.72 V, due to the reduction potential of the PC^*/PC^- couple. According to $E_{1/2}(PC^*/PC^-) = E^{0-0} + E_{1/2}(PC/PC^-)$, the redox potential of the excited-state PC^*/PC^- couple was calculated as 1.56 V depending on an excitation energy change (E^{0-0}) of 2.28 eV. The redox potential of the excited-state PC^*/PC^- couple of Py-DMTP-COF was measured by this method.

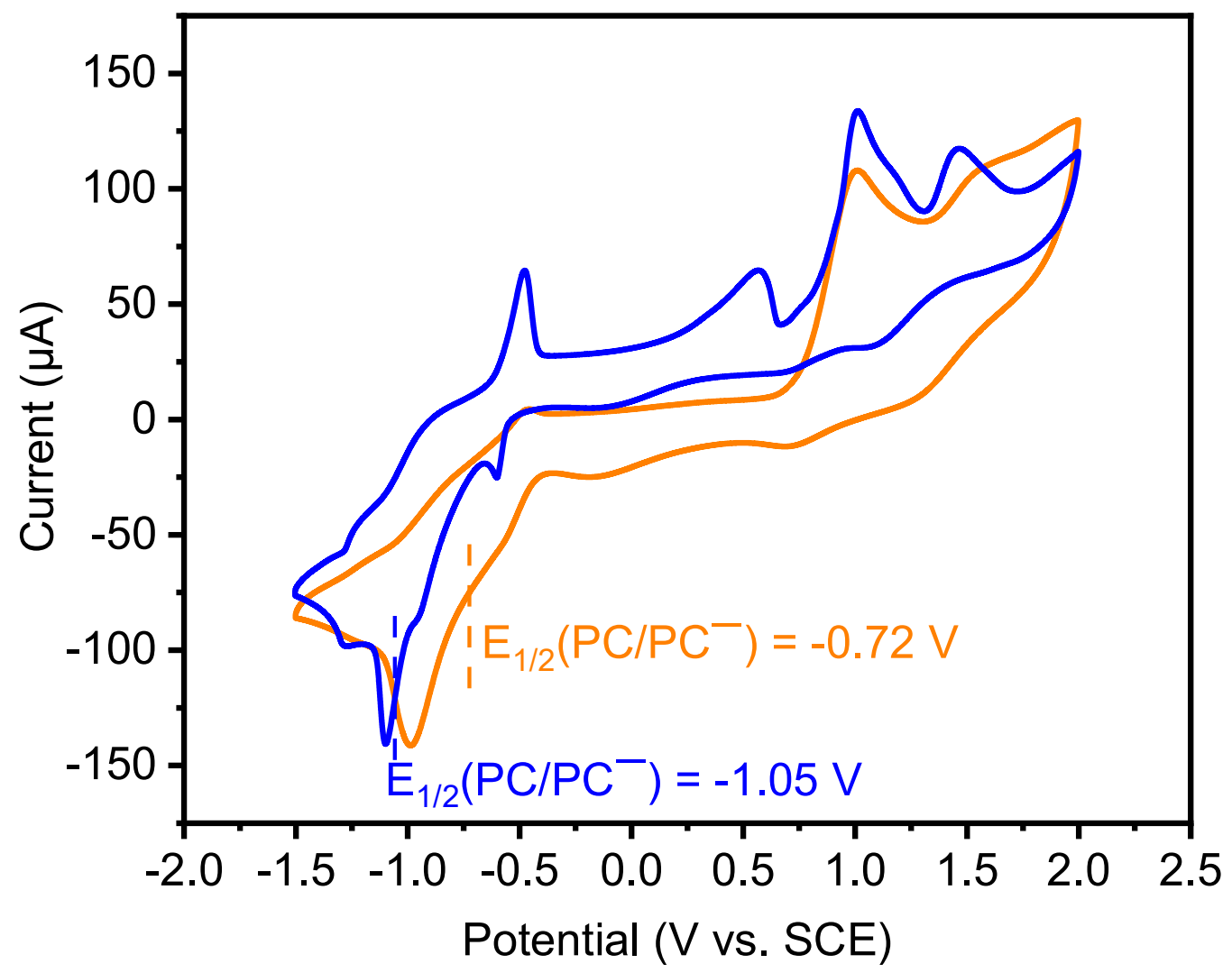

Supplementary Fig. 14 Cyclic voltammogram of NPy-DMTP-COF (orange line) and Py-DMTP-COF (blue line) in MeCN. PC = NPy-DMTP-COF or Py-DMTP-COF. A 3 mm glassy carbon disc electrode as the working electrode, a saturated calomel electrode (SCE) as the reference electrode, and a platinum wire as the counter electrode were employed. Measurement of the reduction potential was tested by cathodic reduction scan at a scan rate of 0.05 V/s.

2a (202.0 mg, 1.0 mmol) dissolved in MeCN (10 mL) with 0.1 M $TBAPF_6$ was transferred into the electrochemical cell to establish the cyclic voltammetry curve for **2a** in MeCN. Subsequently, $NaHCO_3$ (84.0 mg, 1.0 mmol) was introduced into the cell and the mixture was stirred for 10 minutes. The resulting cyclic voltammetry curve for the mixture of **2a** and $NaHCO_3$ in MeCN was then recorded.

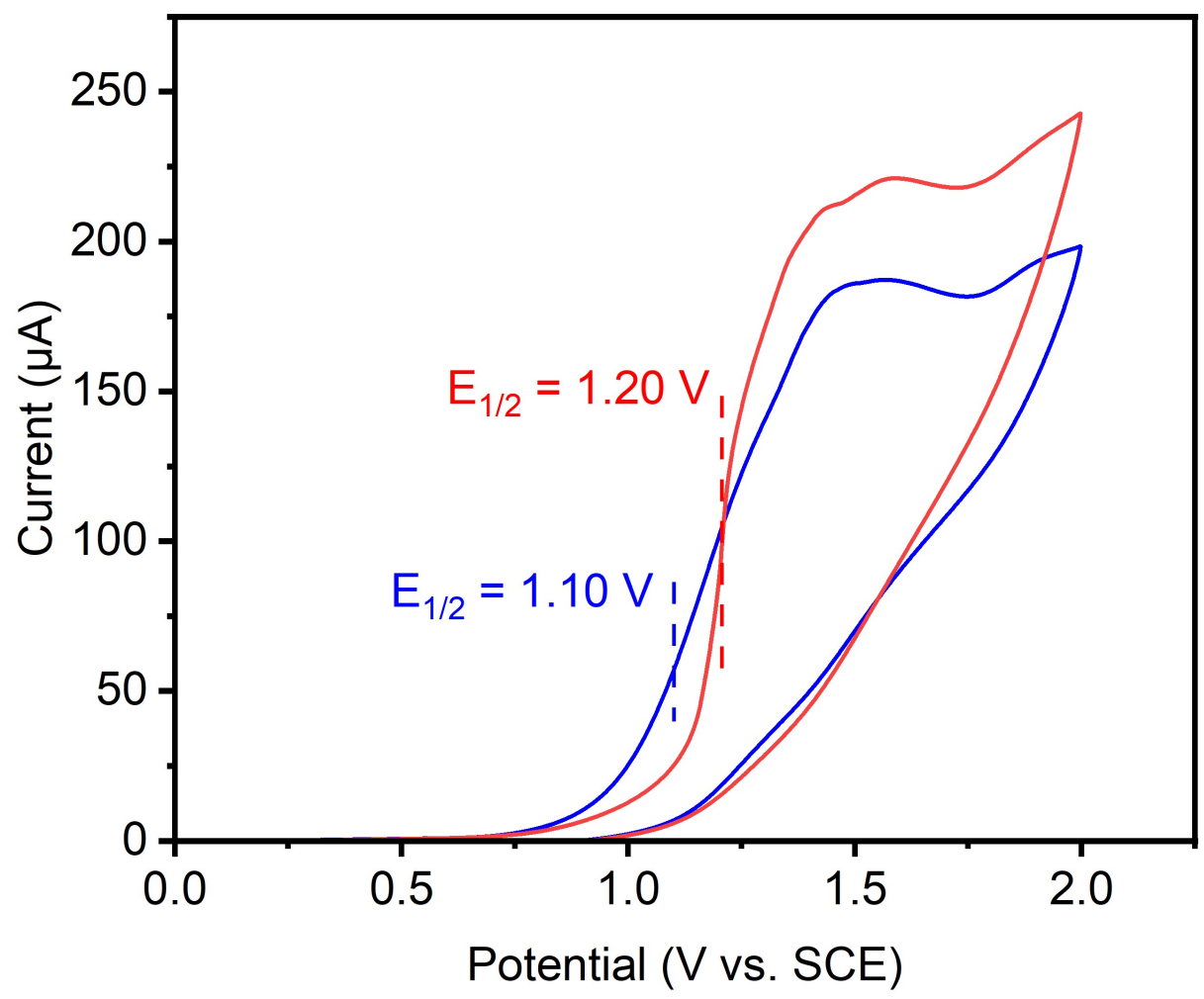

Supplementary Fig. 15 Cyclic voltammograms of **2a** in MeCN (blue line) and the mixture of **2a** and $NaHCO_3$ in MeCN (red line). A 3 mm glassy carbon disc electrode as the working electrode, a saturated calomel electrode (SCE) as the reference electrode, and a platinum wire as the counter electrode were employed. Measurement of the reduction potential was tested by cathodic reduction scan at a scan rate of 0.05 V/s.

Supplementary Table 2 Redox potentials of different photocatalysts and oxidation potential of **2a**[a] [2,3]

Photocatalyst	$E_{1/2}(PC^*/PC^-)$	E^{0-0}	$E_{1/2}(PC/PC^-)$	$E_{1/2}^{ox}$ for **2a**
NPy-DMTP-COF	1.56 V	2.28 eV	-0.72 V	
Py-DMTP-COF	1.04 V	2.09 eV	-1.05 V	
fac-Ir(ppy)$_3$	0.31 V	No detection	-2.19 V	1.20 V
[Ir(ppy)$_2$(dtbbpy)]PF$_6$	0.66 V	No detection	-1.50 V	
4CzlPN	1.35 V	No detection	-1.21 V	

[a] $E_{1/2}$ vs SCE. $E_{1/2}^{ox}$ vs SCE for **2a**.

3.3 Band structure of NPy-DMTP-COF

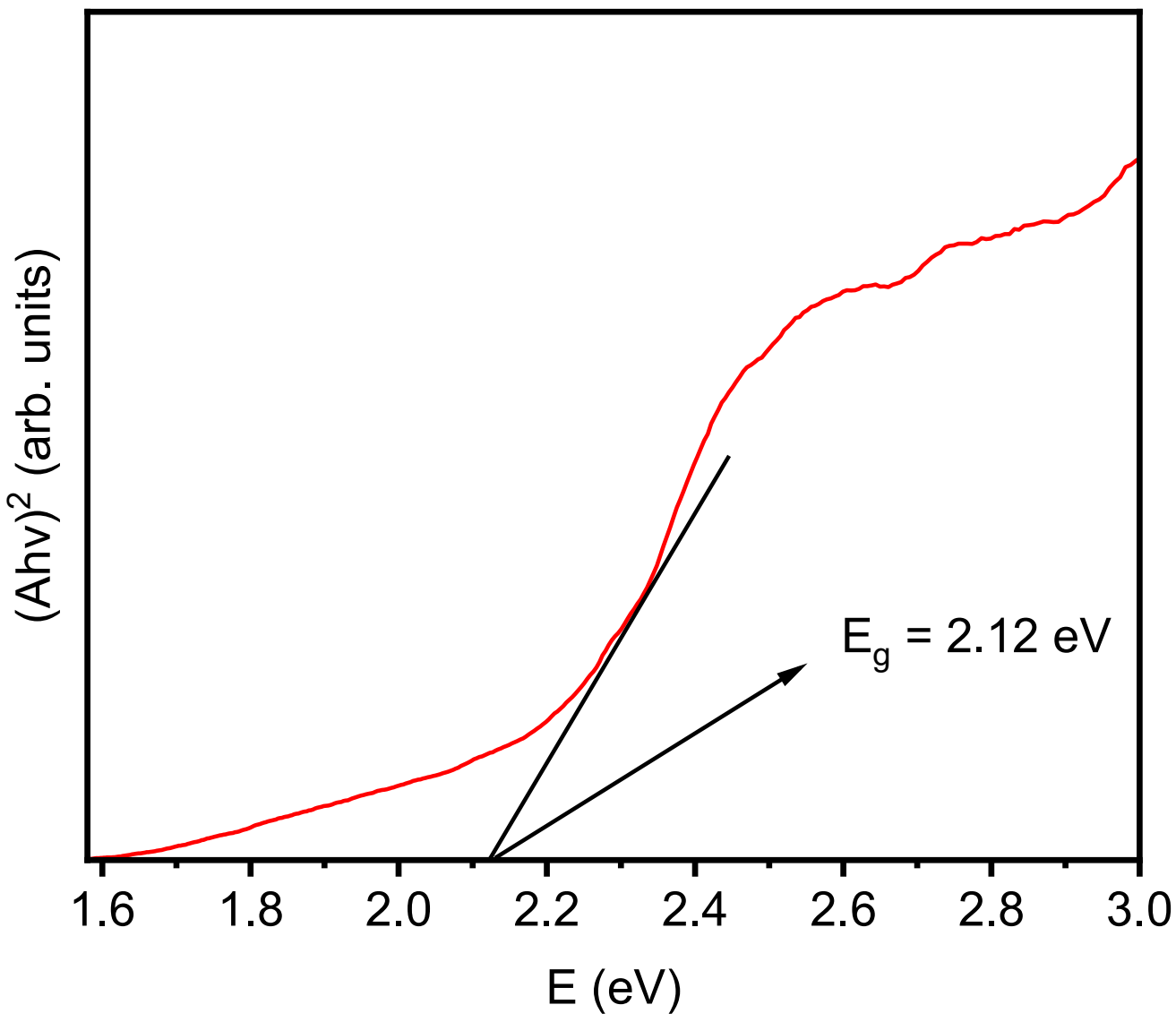

Supplementary Fig. 16 Tauc plots of NPy-DMTP-COF. arb. units = arbitrary units.

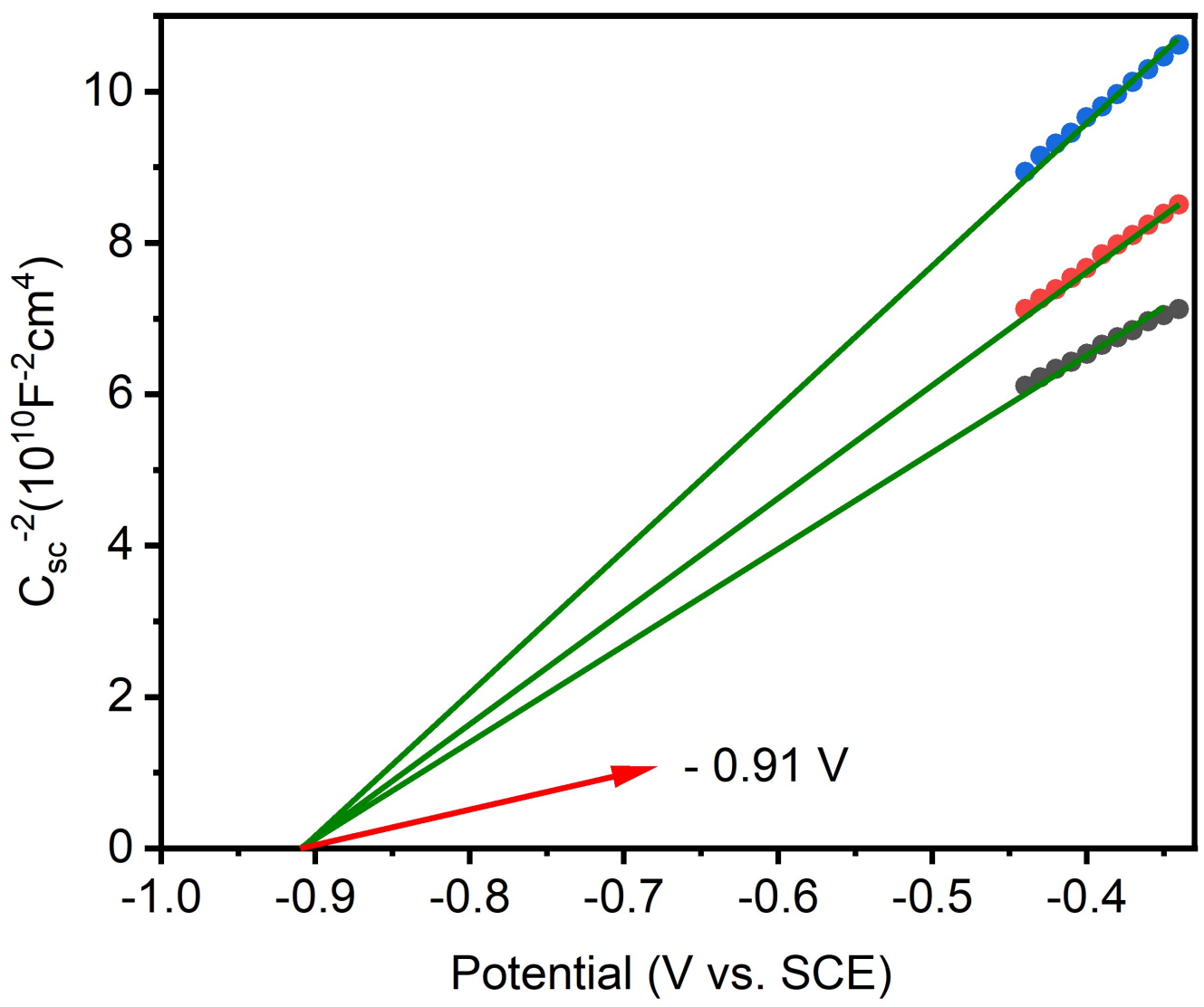

Supplementary Fig. 17 Mott-Schottky plots for NPy-DMTP-COF in 0.5 M Na_2SO_4 aqueous solution with the photocatalyst-coated FTO as work electrode, a platinum wire as the counter electrode, and a saturated calomel electrode (SCE) as the reference electrode at frequency of 1000 Hz (black point), 1500 Hz (red point) and 2000 Hz (blue point).

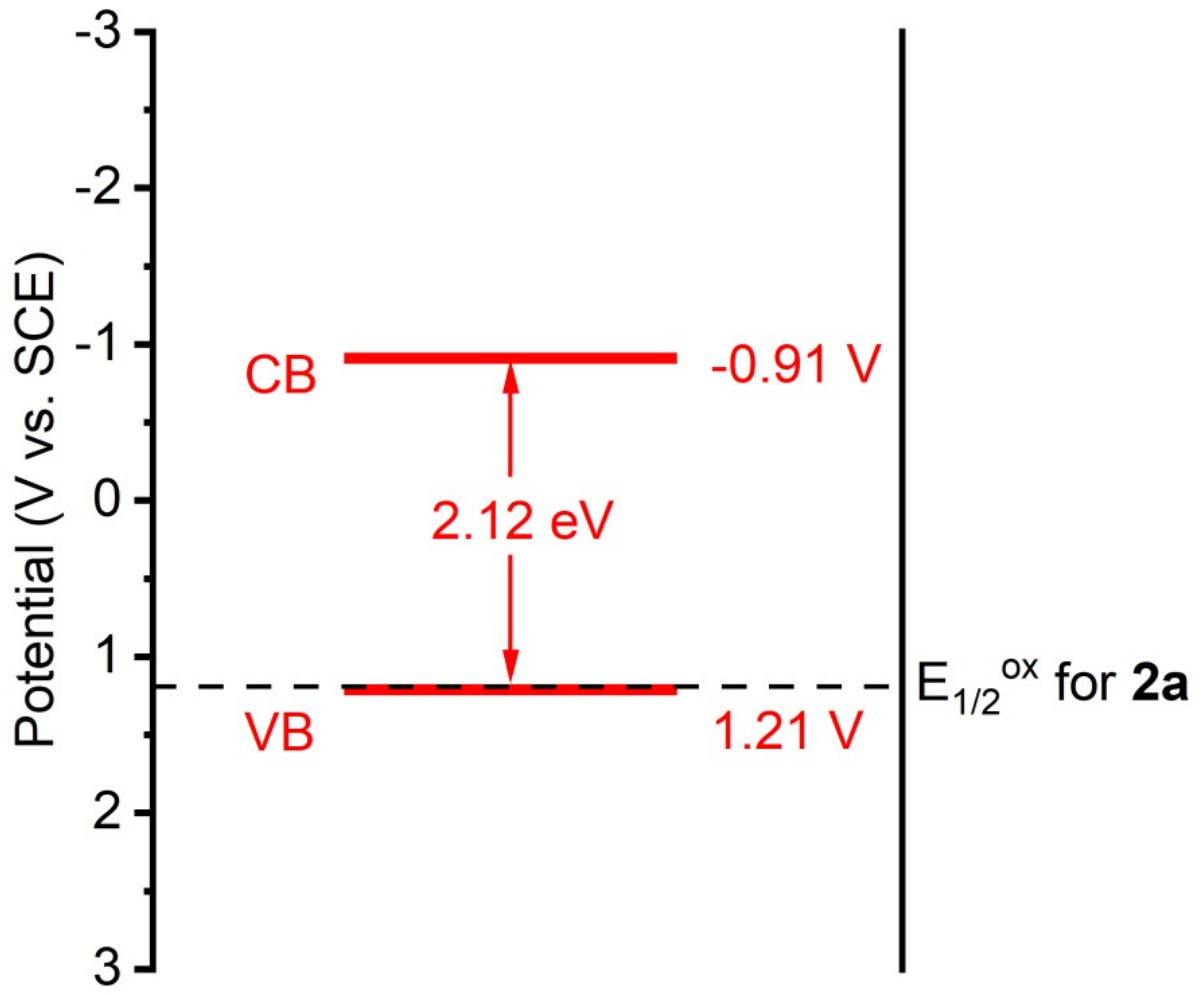

Supplementary Fig. 18 Band structure of NPy-DMTP-COF.

3.4 Unsuccessful radical precursors

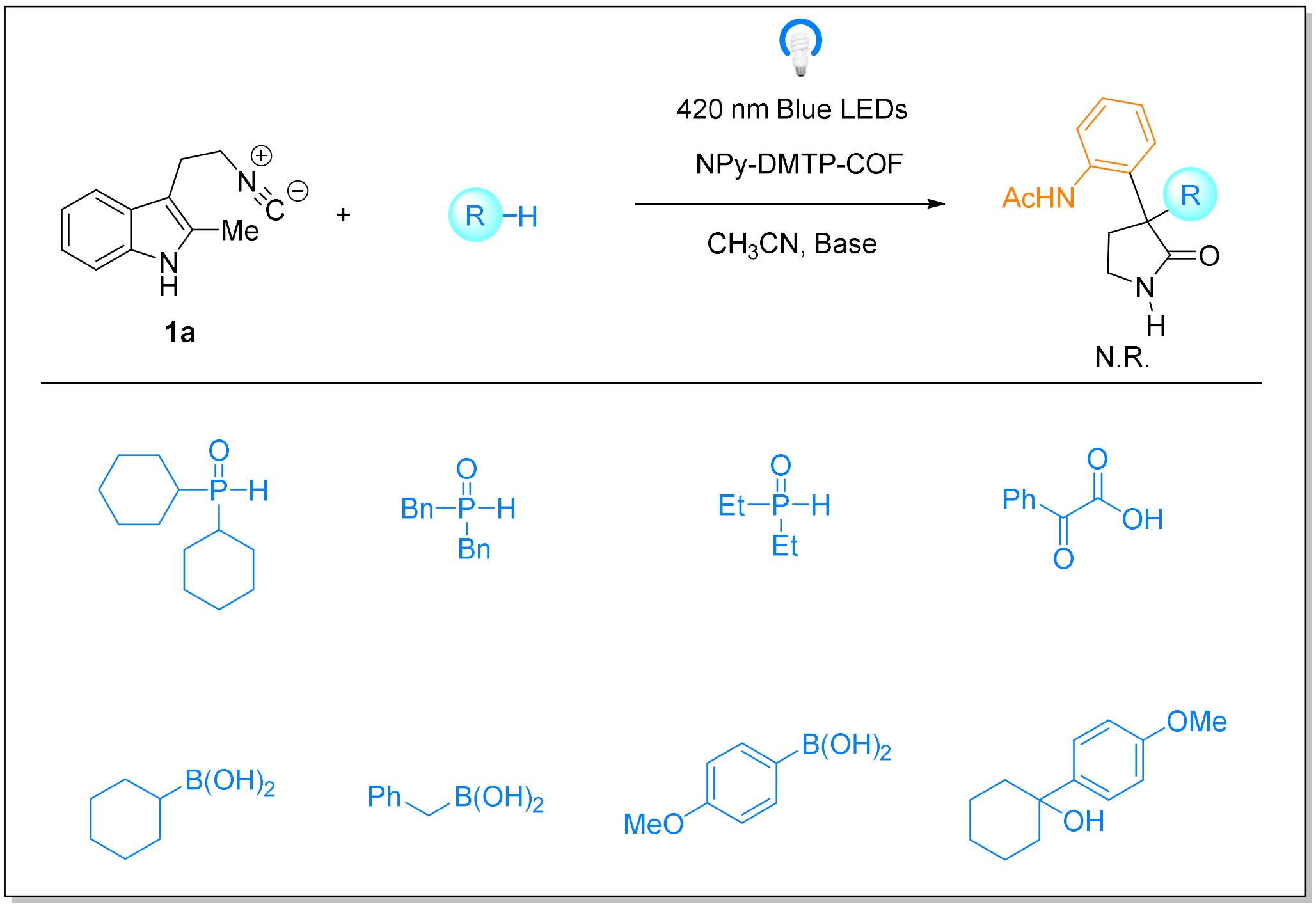

Supplementary Fig. 19 Unsuccessful radical precursors. All reactions were carried out on a 0.1 mmol scale with 1.0 eq. isocyanide **1a**, 2.0 eq. radical precursor, 2 mol% NPy-DMTP-COF and 2.0 eq. base in 4.0 mL solvent under irradiation of blue LEDs with N_2 protection at room temperature for 48 h. The bases Na_2CO_3, $NaHCO_3$, Na_2HPO_4, $KHCO_3$, $CsHCO_3$, DBU, and DABCO were each screened individually.

3.5 PXRD and BET Analysis of NPy-DMTP-COF before and after four consecutive photocatalytic cycles

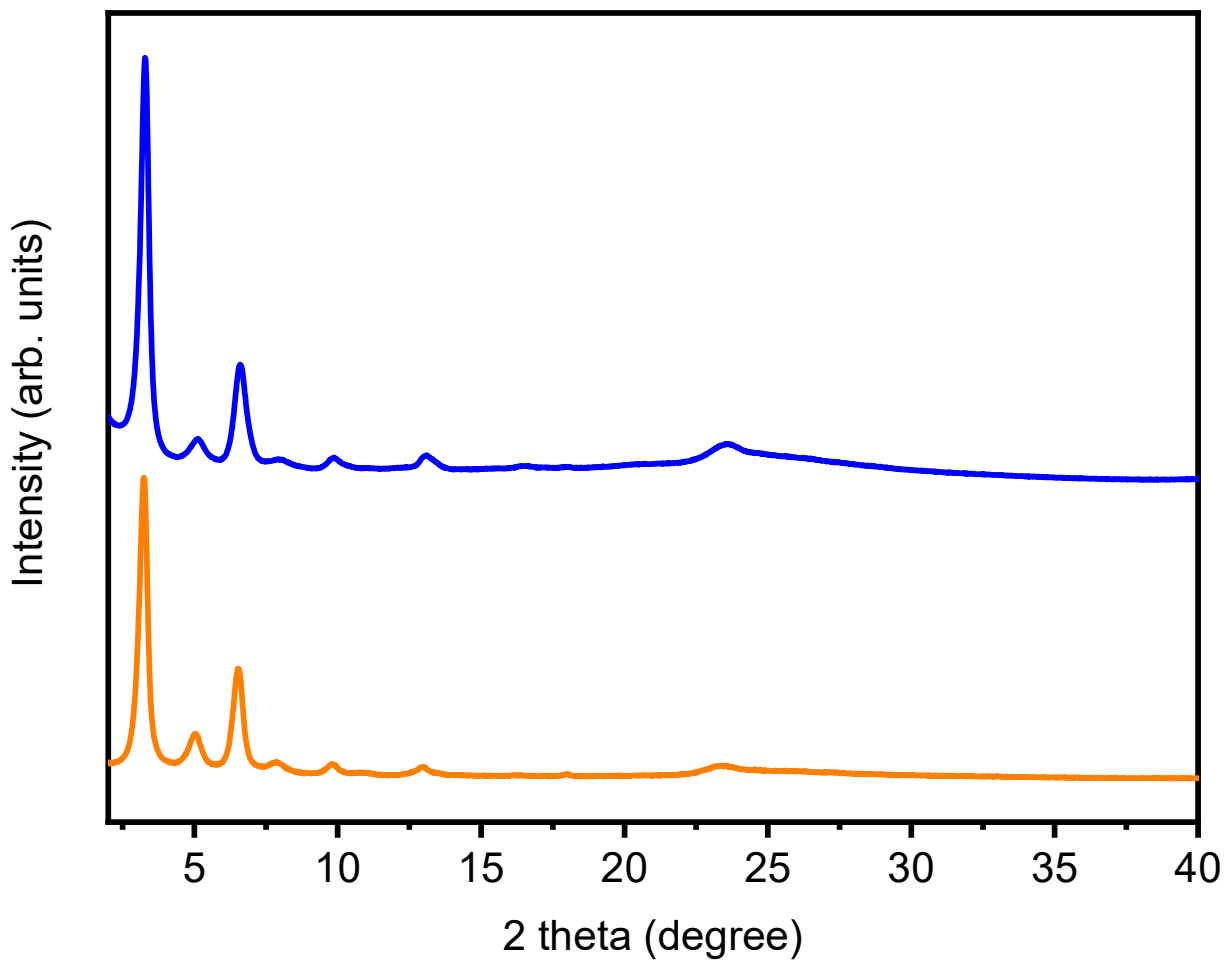

Supplementary Fig. 20 PXRD patterns of NPy-DMTP-COF before (blue line) and after (orange line) four consecutive photocatalytic cycles. arb. units = arbitrary units.

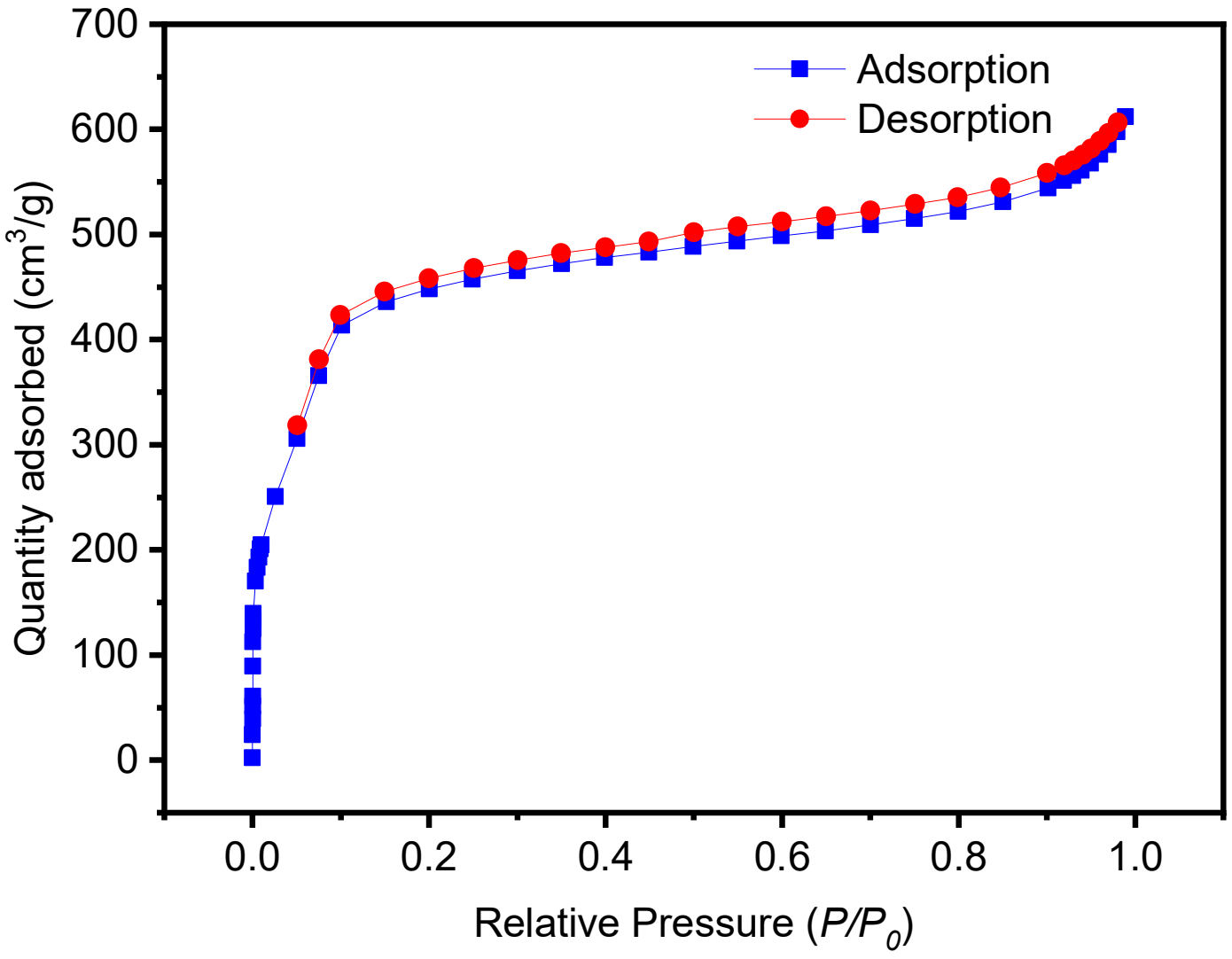

Supplementary Fig. 21 Nitrogen adsorption (blue line) and desorption (red line) isotherm at 77 K of NPy-DMTP-COF recycled after four consecutive photocatalytic cycles.

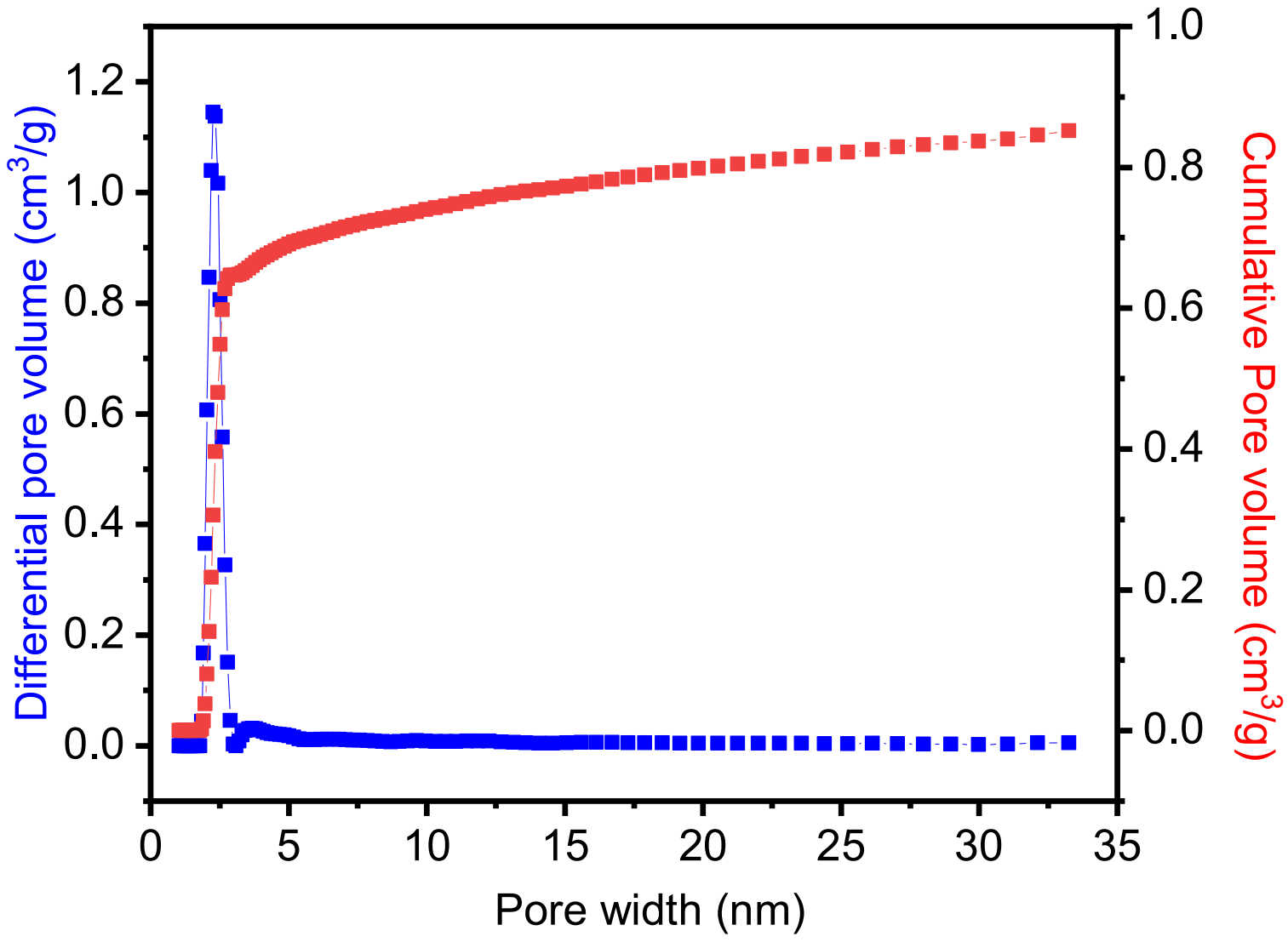

Supplementary Fig. 22 Pore size distribution of NPy-DMTP-COF recycled after four consecutive photocatalytic cycles.

Supplementary Table 3 Surface area, pore volume and pore width of NPy-DMTP-COF before and after four consecutive photocatalytic cycles.

	Before reaction	After four consecutive photocatalytic cycles
Surface area (m^2/g)	1334	1214
Pore Volume (cm^3/g)	1.27	0.85
Pore width (nm)	2.35	2.27

3.6 Radical prevention experiment

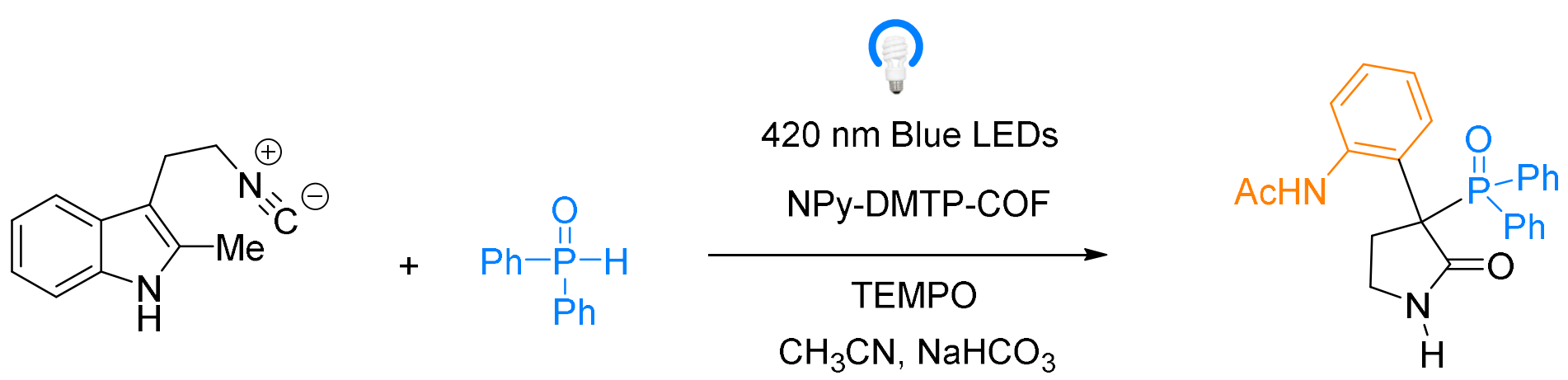

1a 2a 0% yield

Under a nitrogen atmosphere, NPy-DMTP-COF (5.3 mg, 0.006 mmol based on the repeating unit), **1** (0.3 mmol), **2** (0.6 mmol), TEMPO (93.6 mg, 0.6 mmol) and $NaHCO_3$ (50.4 mg, 0.6 mmol) were mixed in acetonitrile (4.0 mL) in a Schlenk tube. The resulting mixture was stirred under blue LED irradiation (420 nm) at room temperature for 48 hours. Upon completion, the residual was filtered through celite pad and filtrate was

concentrated. TLC and crude NMR results indicated that the yield of **3a** is 0%. A TEMPO-**2a** adduct was detected by high-resolution mass spectrometry of mixture.

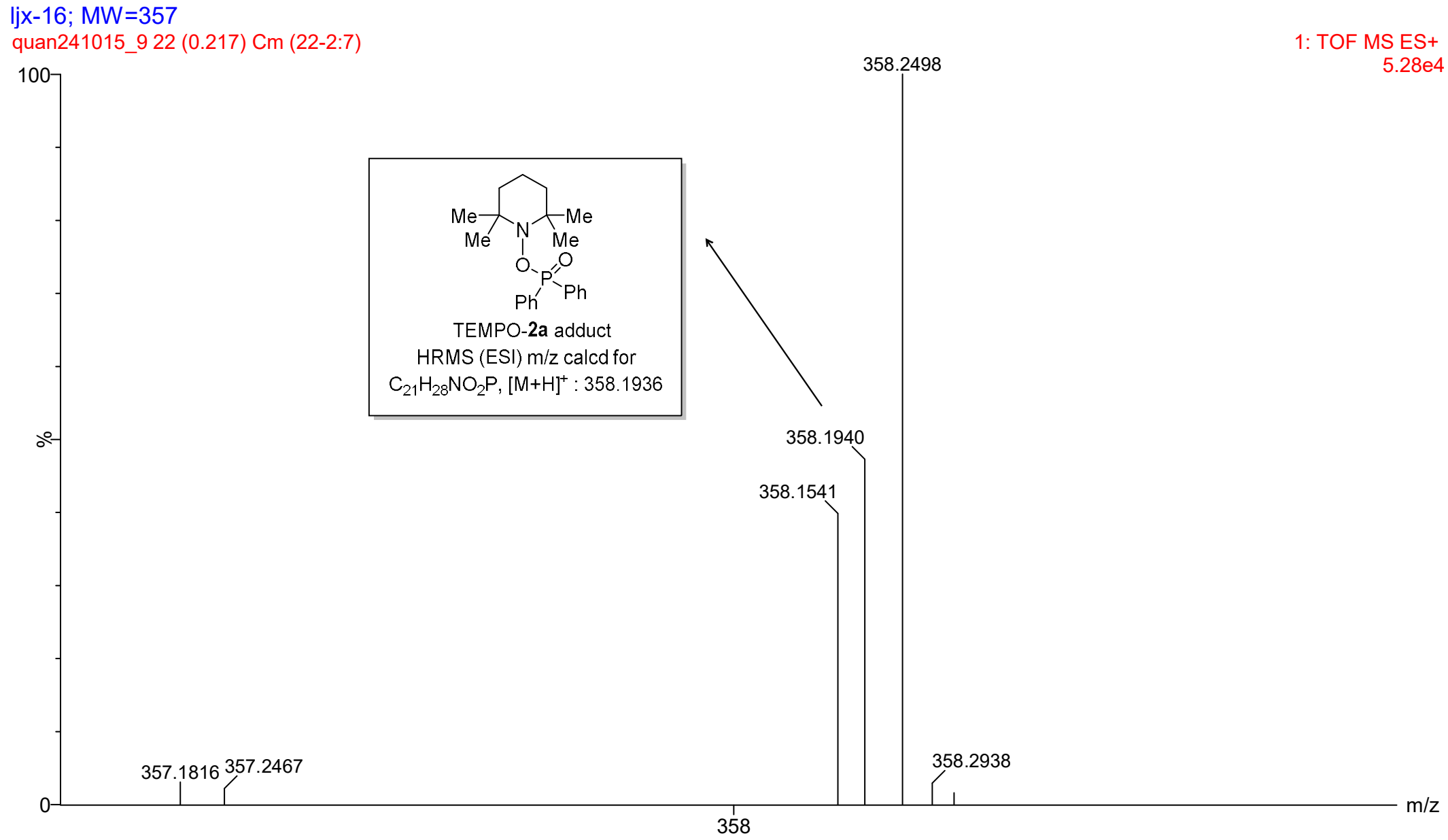

Supplementary Fig. 23 HRMS spectrum analysis of reaction in the presence of TEMPO. TEMPO is 2,2,6,6-tetramethylpiperidine 1-oxide.

3.7 The isolation of intermediate 12 and the synthesis of 3a from 12 *via* photocatalytic reaction

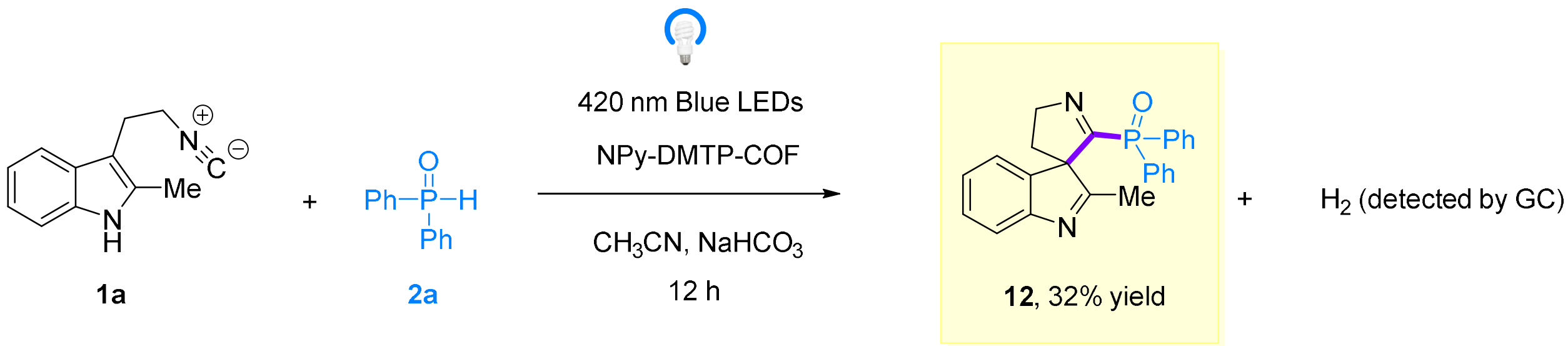

At N_2 atmosphere, NPy-DMTP-COF (5.3 mg, 0.006 mmol based on the repeating unit), **1a** (55.2 mg, 0.3 mmol), **2a** (121.2 mg, 0.6 mmol) and $NaHCO_3$ (50.4 mg, 0.6 mmol) were mixed in acetonitrile (4.0 mL) in a Schlenk tube. The resulting mixture was stirred under blue LED irradiation (420 nm) at room temperature for 12 hours. The residual was filtered through celite pad and filtrate was concentrated. The mixture was purified by silica gel flash chromatography (petroleum ether : ethyl acetate, 1 : 5) to afford the desired product **12**.

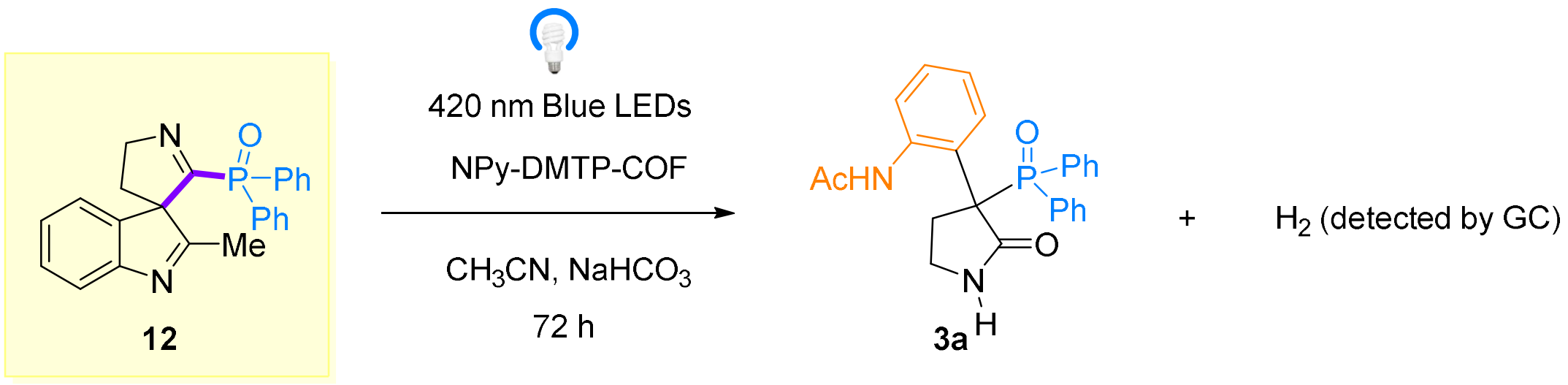

At N_2 atmosphere, NPy-DMTP-COF (5.3 mg, 0.006 mmol based on the repeating unit), **12** (55.2 mg, 0.3 mmol) and $NaHCO_3$ (50.4 mg, 0.6 mmol) were mixed in acetonitrile (4.0 mL) in a Schlenk tube. The resulting mixture was stirred under blue LED irradiation (420 nm) at room temperature for 72 hours. Upon completion, the residual was filtered through celite pad and filtrate was concentrated. The mixture was purified by silica gel flash chromatography (petroleum ether : ethyl acetate, 1 : 3) to afford the desired product **3a**. Other controlled experiments were carried out following the same protocol.

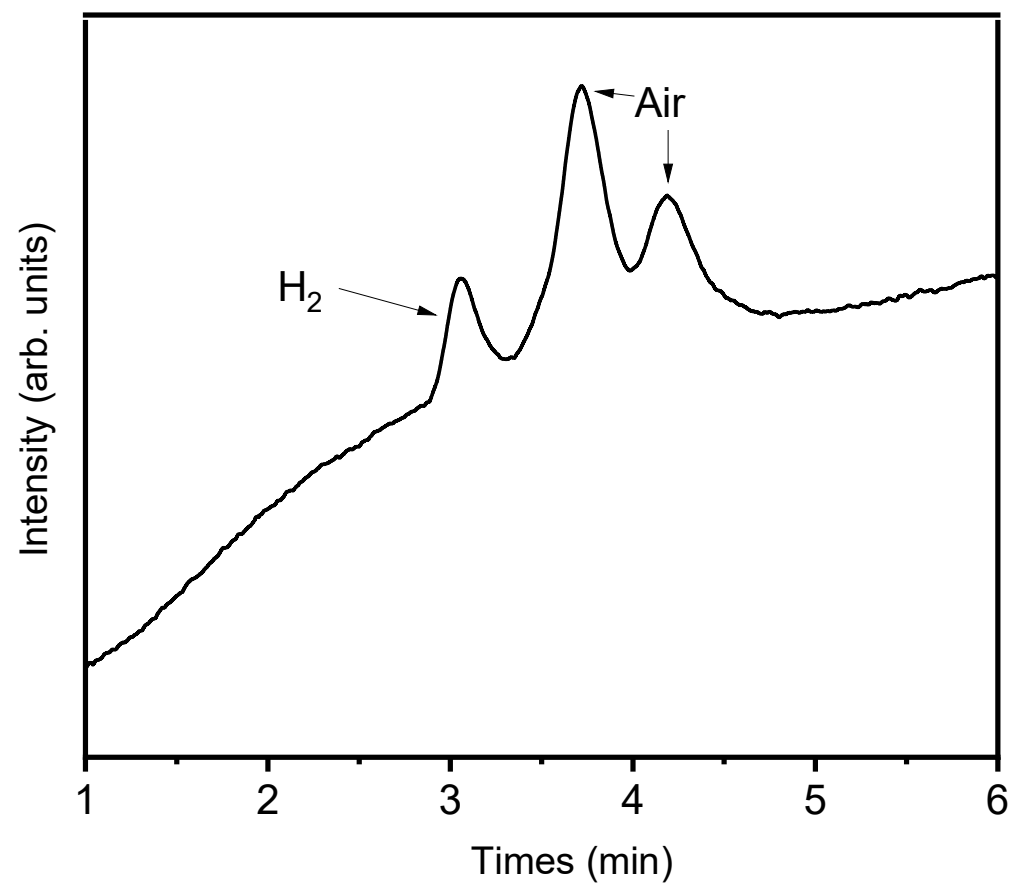

Supplementary Fig. 24 The GC of reaction between **1a** and **2a**. The reaction was carried out on a 0.3 mmol scale with 1.0 eq. isocyanide **1a**, 2.0 eq. phosphine oxide **2a**, 2 mol% NPy-DMTP-COF and 2.0 eq. $NaHCO_3$ in 4.0 mL solvent under irradiation of blue LEDs with N_2 protection at room temperature for 12 h. arb. units = arbitrary units.

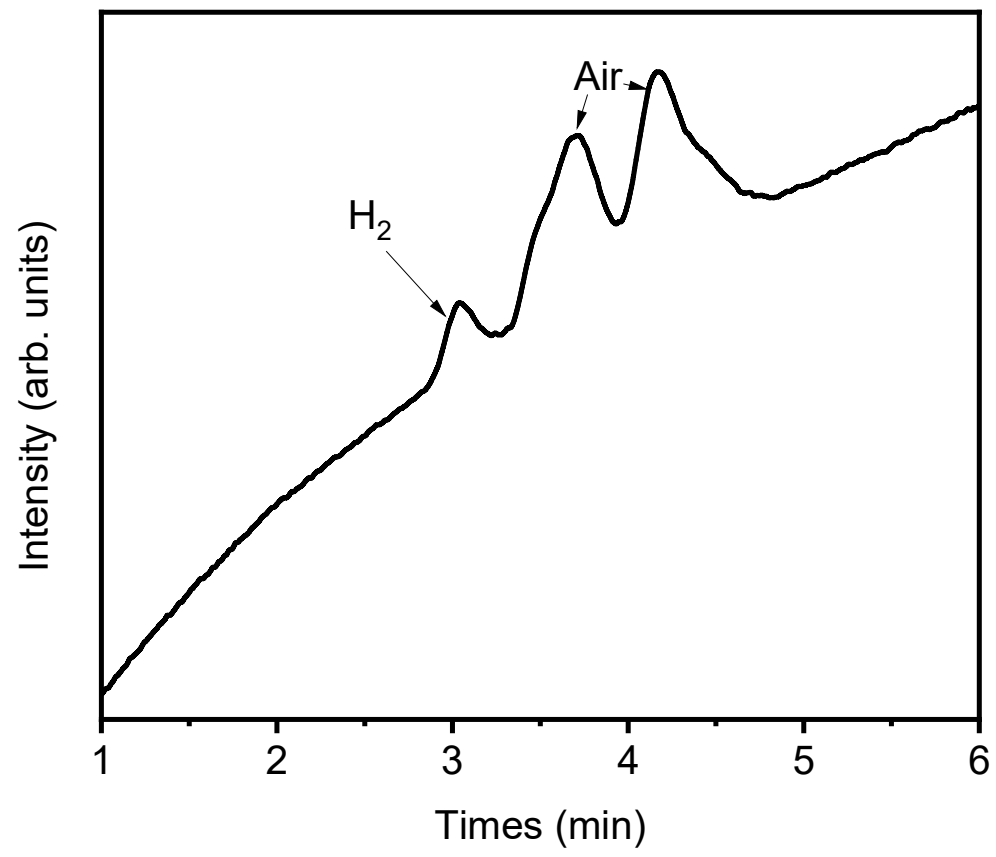

Supplementary Fig. 25 The GC of reaction from **12** to **3a**. The reaction was carried out on a 0.3 mmol scale with 1.0 eq. **12**, 2 mol% NPy-DMTP-COF and 2.0 eq. Na_2HPO_4 in 4.0 mL solvent under irradiation of blue LEDs with N_2 protection at room temperature for 48 h. arb. units = arbitrary units.

3.8 Stern-Volmer fluorescence quenching studies

Stern-Volmer experiments for all the components of the reaction mixture were carried out to monitor the emission intensity of solutions of NPy-DMTP-COF (0.5 μM) containing variable amounts of the quencher in CH_3CN. The emission intensity at 564 nm was collected with excited wavelength of 406 nm in CH_3CN using a LS55 spectrophotometer (Perkin-Elmer, USA). After degassing the sample with a stream of nitrogen for 10 minutes, the emission intensity of the sample was collected and plots were constructed according to the Stern-Volmer equation $I_0/I = 1 + K_q t_0[Q]$.

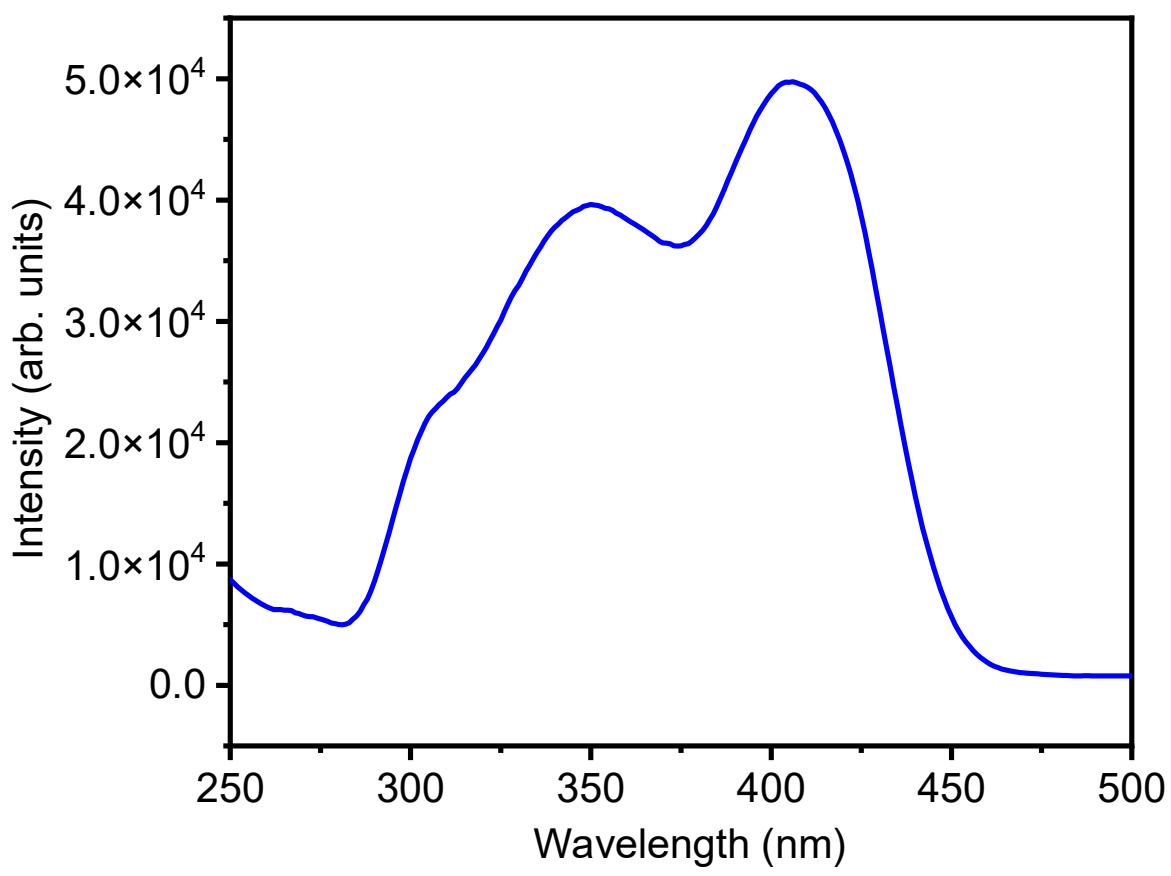

Supplementary Fig. 26 NPy-DMTP-COF excitation spectrum in CH_3CN. arb. units = arbitrary units.

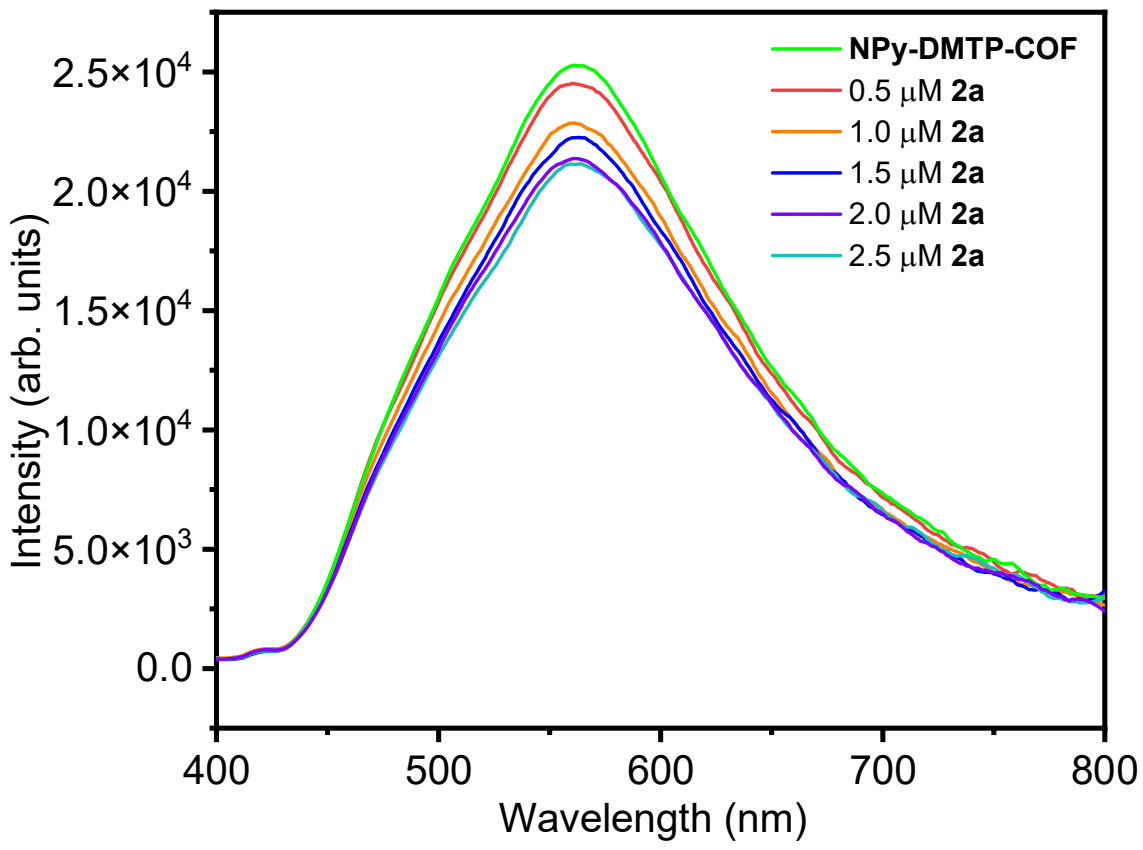

Supplementary Fig. 27 NPy-DMTP-COF emission quenching with **2a**. arb. units = arbitrary units.

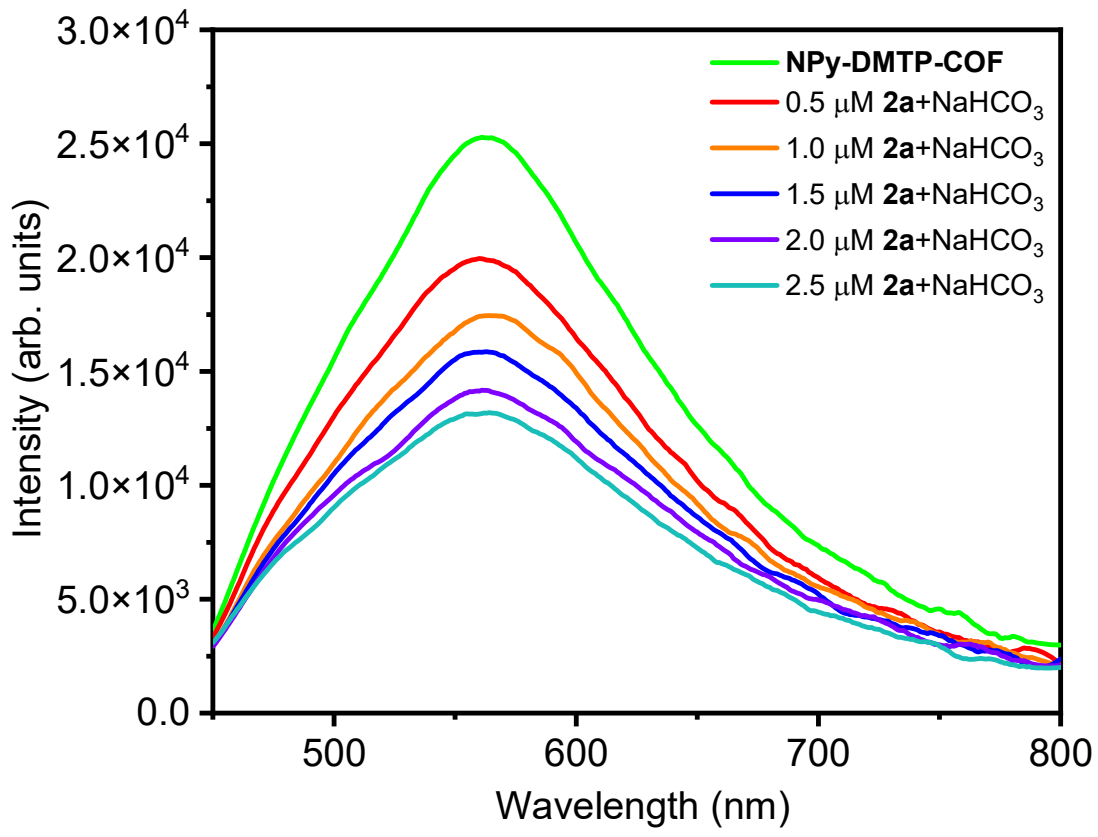

Supplementary Fig. 28 NPy-DMTP-COF emission quenching with **2a** and $NaHCO_3$ mixture. arb. units = arbitrary units.

3.9 Isotope labeling experiment

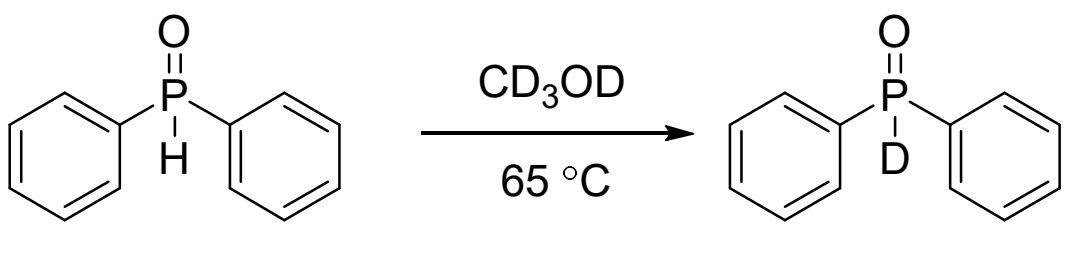

2a [D]-**2a** (68% D)

Diphenylphosphine oxide-d_1 was synthesized according to known procedure[4]. Diphenylphosphine oxide **2a** (101.0 mg, 0.5 mmol) was dissolved in CD_3OD (99.8% D, 2.0 mL) under oxygen atmosphere, and stir at 65 °C for 12 h. Then the solvent was removed through the vacuum (this process was repeated one time). ^{1}H NMR (400 MHz, $CDCl_3$) δ 8.69 (s, 0.32H), 7.76 – 7.68 (m, 4H), 7.63 – 7.55 (m, 2H), 7.54 – 7.47 (m, 4H).

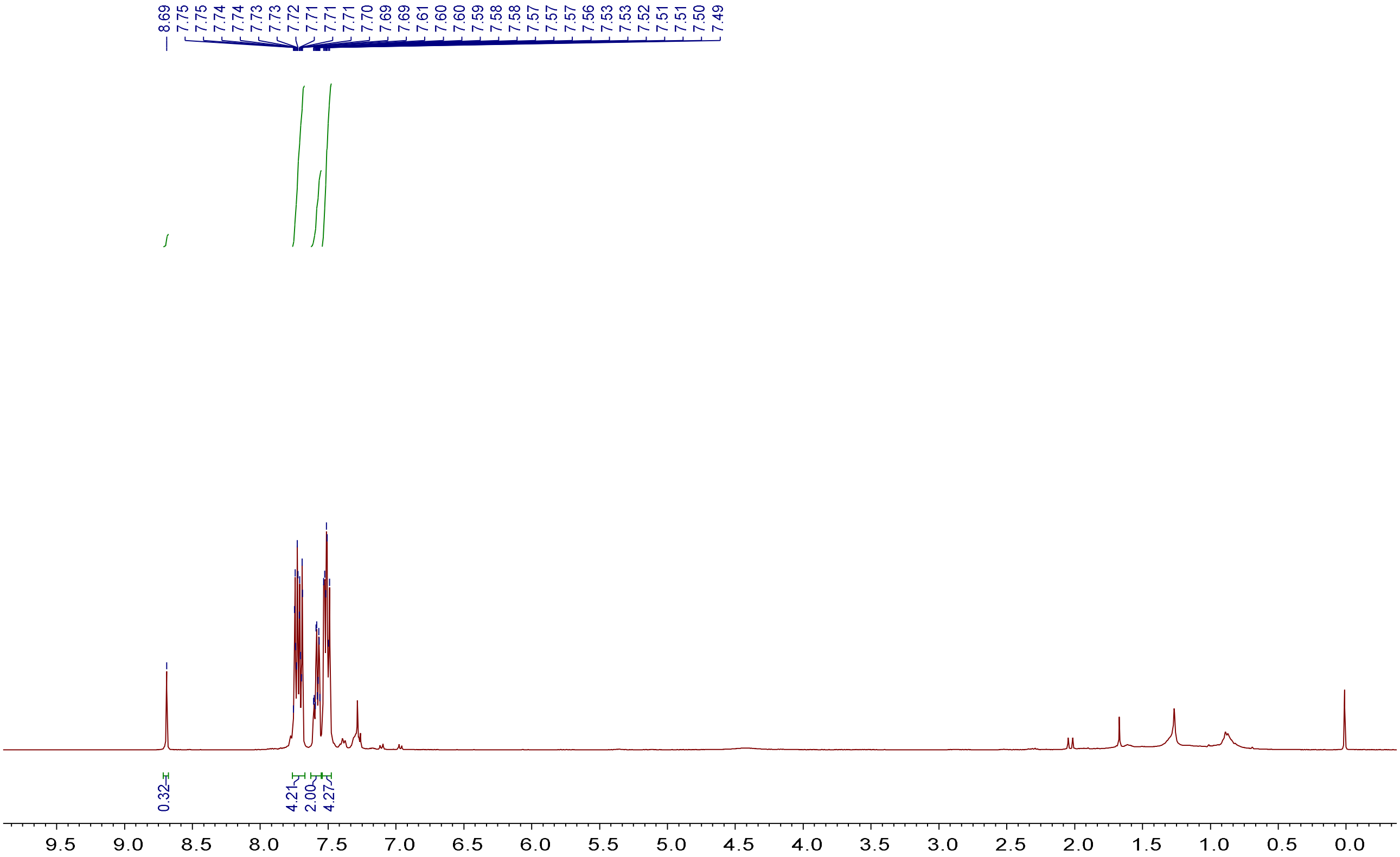

Supplementary Fig. 29 ^{1}H NMR spectra of [D]-**2a** (68% D).

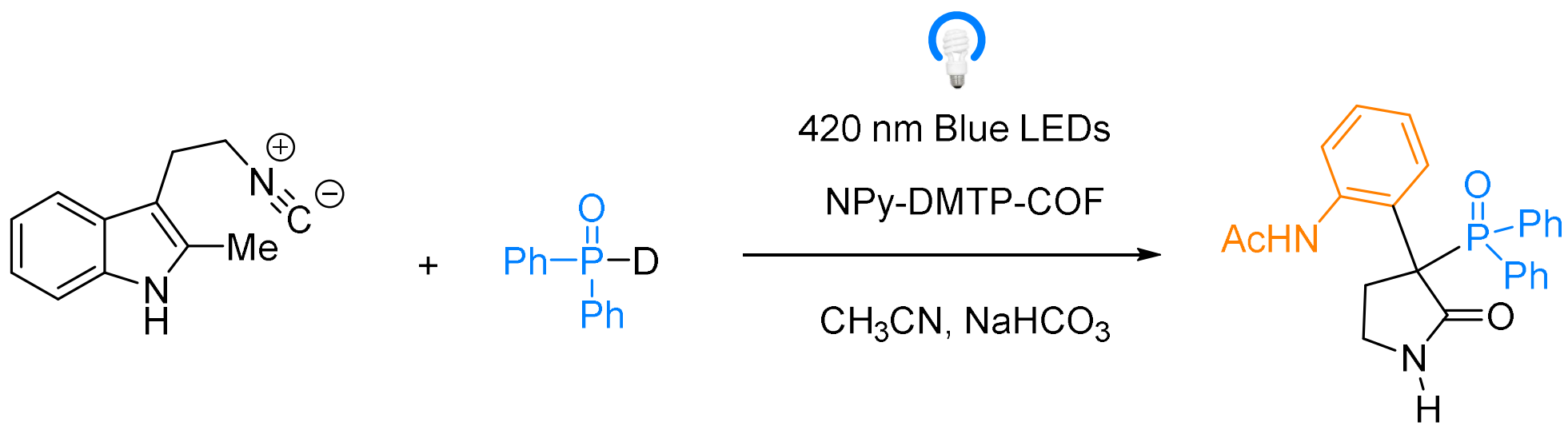

1a [D]-**2a** (68% D) **3a**, 69% yield

At N_2 atmosphere, NPy-DMTP-COF (5.3 mg, 0.006 mmol based on the repeating unit), **1a** (55.2 mg, 0.3 mmol), [D]-**2a** (68% D) (121.8 mg, 0.6 mmol) and $NaHCO_3$ (50.4 mg, 0.6 mmol) were mixed in acetonitrile (4.0 mL) in a Schlenk tube. The resulting mixture was stirred under blue LED irradiation (420 nm) at room temperature for 72 hours. Upon completion, the mixture was filtered with 0.25 μ m filtration membrane, concentrated the filtrate through the vacuum, and checked by ^{1}H NMR.

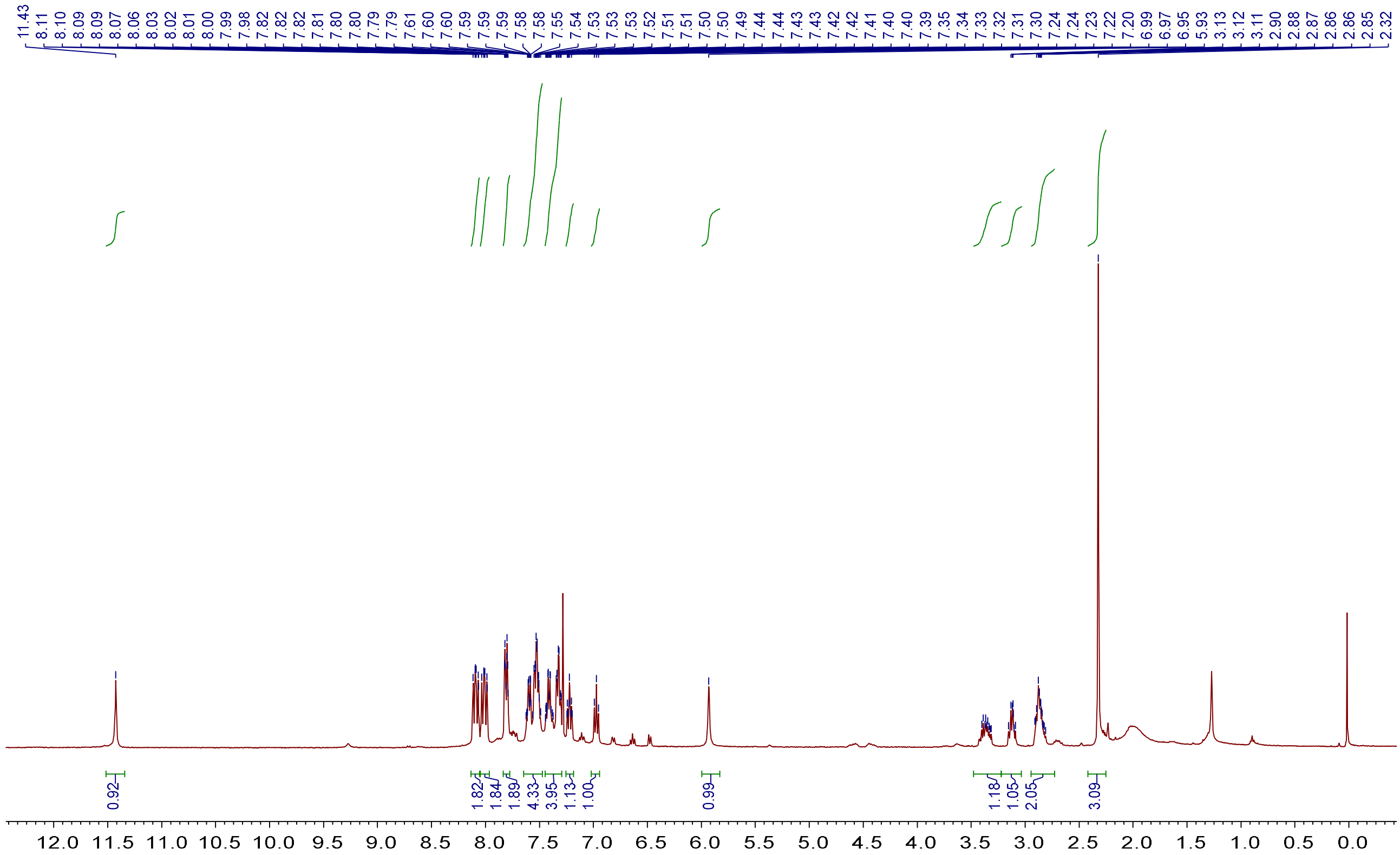

Supplementary Fig. 30 Crude ^{1}H NMR spectra of the reaction.

4. Spectral data of products

3-(2-Isocyanoethyl)-2-methyl-1H-indole

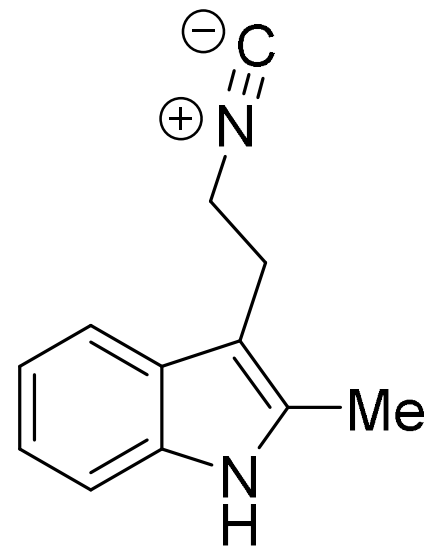

1a

White solid, 956.8 mg, 52% yield, ^{1}H NMR (400 MHz, Chloroform-*d*) δ 7.91 (s, 1H), 7.47 (d, *J* = 7.6 Hz, 1H), 7.32 (d, *J* = 7.4 Hz, 1H), 7.21 – 7.11 (m, 2H), 3.68 – 3.57 (m, 2H), 3.20 – 3.13 (m, 2H), 2.46 (s, 3H). ^{13}C NMR (101 MHz, Chloroform-*d*) δ 156.0 (t, *J* = 5.9 Hz), 135.3, 132.8, 127.9, 121.4, 119.6, 117.3, 110.5, 106.6, 42.1 (t, *J* = 6.3 Hz), 24.9, 11.8. HRMS (ESI) *m/z* calcd for $C_{12}H_{12}N_2$, $[M+H]^+$: 185.1079, found: 185.1075.

3-(2-Isocyanoethyl)-2,5-dimethyl-1H-indole

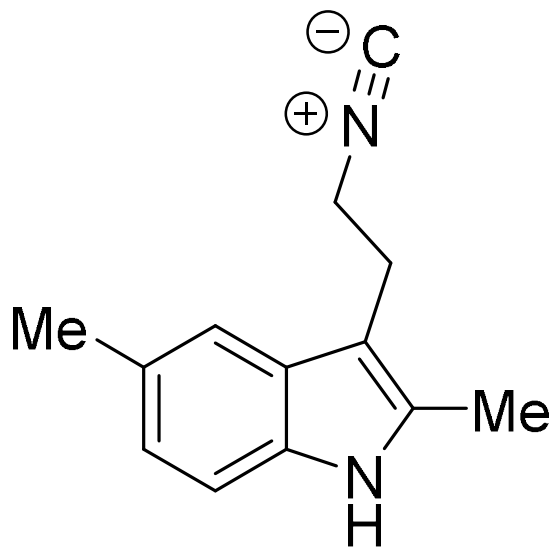

1b

Colorless oil, 1.13 g, 57% yield, ^{1}H NMR (400 MHz, Chloroform-*d*) δ 7.81 (s, 1H), 7.25 (s, 1H), 7.20 (d, *J* = 8.2 Hz, 1H), 7.00 (d, *J* = 8.2 Hz, 1H), 3.65 – 3.56 (m, 2H), 3.17 – 3.06 (m, 2H), 2.49 (s, 3H), 2.44 (s, 3H). ^{13}C NMR (101 MHz, Chloroform-*d*) δ 155.9 (t, *J* = 5.9 Hz), 133.6, 132.9, 128.8, 128.1, 122.9, 117.1, 110.2, 106.2, 42.0 (t, *J* = 6.3 Hz), 25.0, 21.6, 11.8. HRMS (ESI) *m/z* calcd for $C_{13}H_{14}N_2$, $[M+H]^+$: 199.1235, found: 199.1230.

3-(2-Isocyanoethyl)-2,6-dimethyl-1H-indole

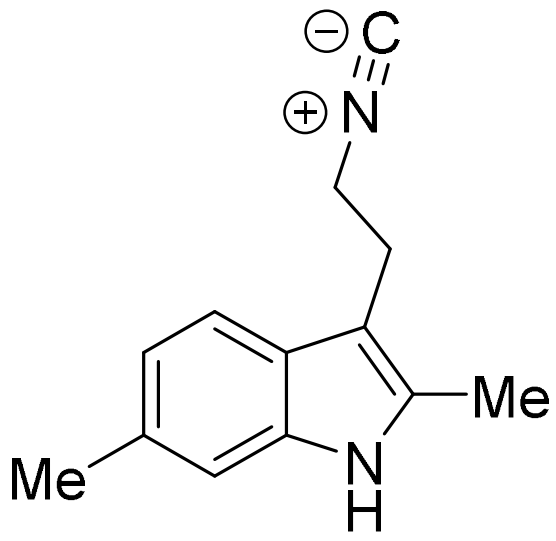

1c

Yellow oil, 1.19 g, 60% yield, ^{1}H NMR (400 MHz, Chloroform-*d*) δ 7.82 (s, 1H), 7.41 (d, *J* = 8.0 Hz, 1H), 7.10 (s, 1H), 7.05 (d, *J* = 8.1 Hz, 1H), 3.62 (t, *J* = 7.1 Hz, 2H), 3.15 (t, *J* = 7.0 Hz, 2H), 2.56 (s, 3H), 2.43 (s, 3H). ^{13}C NMR (101 MHz, Chloroform-*d*) δ 155.8 (t, *J* = 5.4 Hz), 135.8, 132.2, 131.1, 125.8, 121.2, 117.0, 110.8, 106.3, 42.2 (t, *J* = 6.5 Hz), 25.0, 21.8, 11.7. HRMS (ESI) *m/z* calcd for $C_{13}H_{14}N_2$, $[M+H]^+$: 199.1235, found: 199.1236.

3-(2-Isocyanoethyl)-2,7-dimethyl-1H-indole

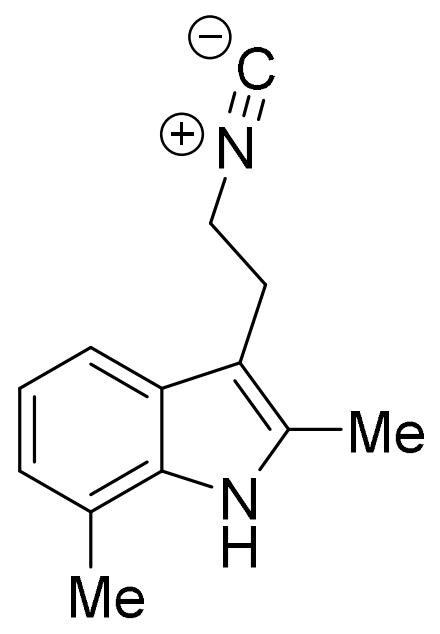

1d

Colorless oil, 1.08 g, 55% yield, ^{1}H NMR (400 MHz, Chloroform-*d*) δ 7.93 (s, 1H), 7.33 (d, *J* = 7.8 Hz, 1H), 7.07 (t, *J* = 7.4 Hz, 1H), 7.00 (d, *J* = 7.6 Hz, 1H), 3.63 (t, *J* = 7.1 Hz, 2H), 3.16 (t, *J* = 7.1 Hz, 2H), 2.55 – 2.46 (m, 6H). ^{13}C NMR (101 MHz, Chloroform-*d*) δ 155.9 (t, *J* = 5.8 Hz), 134.8, 132.5, 127.4, 122.1, 119.84, 119.82, 115.0, 107.1, 42.1 (t, *J* = 5.9 Hz), 25.1, 16.7, 11.8. HRMS (ESI) *m/z* calcd for $C_{13}H_{14}N_2$, $[M+H]^+$: 199.1235, found: 199.1235.

3-(2-Isocyanoethyl)-5-methoxy-2-methyl-1H-indole

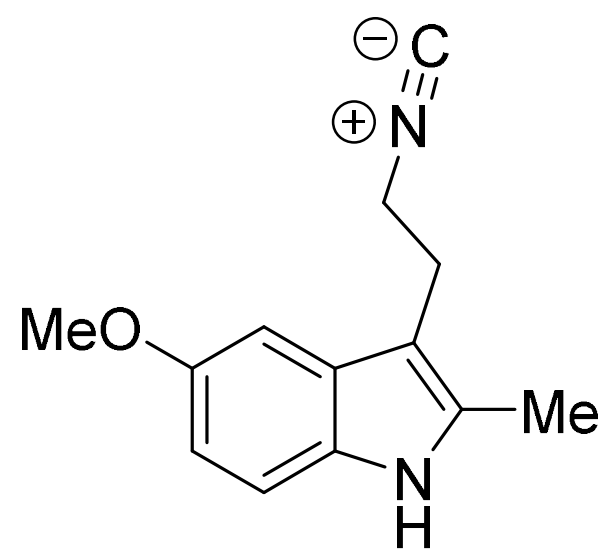

1e

Colorless oil, 791.8 mg, 37% yield, ^{1}H NMR (400 MHz, Chloroform-*d*) δ 7.83 (s, 1H), 7.20 (d, *J* = 8.7 Hz, 1H), 6.91 (d, *J* = 2.4 Hz, 1H), 6.82 (dd, *J* = 8.7, 2.4 Hz, 1H), 3.89 (s, 3H), 3.60 (t, *J* = 7.1 Hz, 2H), 3.11 (t, *J* = 7.1 Hz, 2H), 2.44 (s, 3H). ^{13}C NMR (101 MHz, Chloroform-*d*) δ 156.0 (t, *J* = 5.8 Hz), 154.2, 133.7, 130.3, 128.4, 111.2, 110.8, 106.5, 99.94, 56.0, 41.9 (d, *J* = 6.4 Hz), 25.0, 11.9. HRMS (ESI) *m/z* calcd for $C_{13}H_{14}N_2O$, $[M+H]^+$: 215.1184, found: 215.1187.

5-Bromo-3-(2-isocyanoethyl)-2-methyl-1H-indole

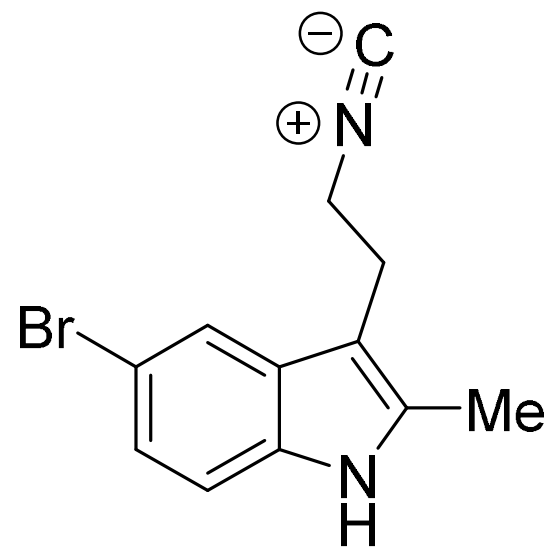

1f

White solid, 1.15 g, 44% yield, ^{1}H NMR (400 MHz, Chloroform-*d*) δ 8.02 (s, 1H), 7.56 (d, *J* = 1.8 Hz, 1H), 7.24 (dd, *J* = 8.5, 1.9 Hz, 1H), 7.17 (d, *J* = 8.6 Hz, 1H), 3.67 – 3.51 (m, 2H), 3.12 – 3.00 (m, 2H), 2.46 (s, 3H). ^{13}C NMR (101 MHz, Chloroform-*d*) δ 156.3 (t, *J* = 5.2 Hz), 134.4, 133.9, 129.7, 124.2, 119.9, 112.8, 112.0, 106.4, 42.0 (t, *J* = 6.5 Hz), 24.7, 11.9. HRMS (ESI) *m/z* calcd for $C_{12}H_{11}BrN_2$, $[M+H]^+$: 263.0184, found: 263.0182.

5-Chloro-3-(2-isocyanoethyl)-2-methyl-1H-indole

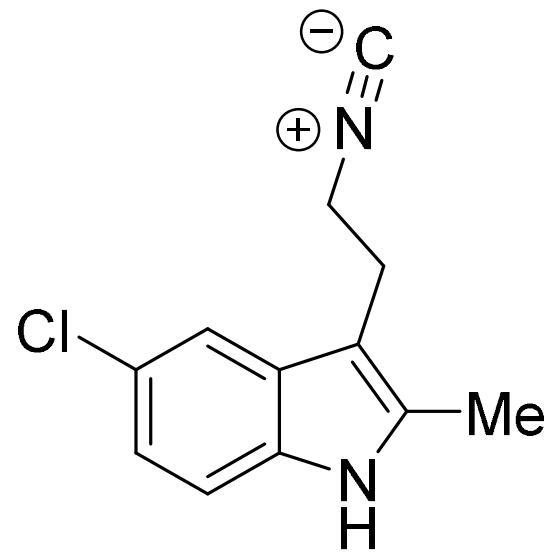

1g

White solid, 1.37 g, 63% yield, ^{1}H NMR (400 MHz, Chloroform-*d*) δ 8.01 (s, 1H), 7.41 (d, *J* = 2.0 Hz, 1H), 7.21 (d, *J* = 8.5 Hz, 1H), 7.11 (dd, *J* = 8.6, 2.0 Hz, 1H), 3.67 – 3.53 (m, 2H), 3.13 – 3.00 (m, 2H), 2.45 (s, 3H). ^{13}C NMR (101 MHz, Chloroform-*d*) δ 156.2 (d, *J* = 5.0 Hz), 134.6, 133.6, 129.1, 125.3, 121.6, 116.8, 111.5, 106.5, 42.0 (t, *J* = 6.5 Hz), 24.7, 11.9. HRMS (ESI) *m/z* calcd for $C_{12}H_{11}ClN_2$, $[M+H]^+$: 219.0689, found: 219.0691.

5-Fluoro-3-(2-isocyanoethyl)-2-methyl-1H-indole

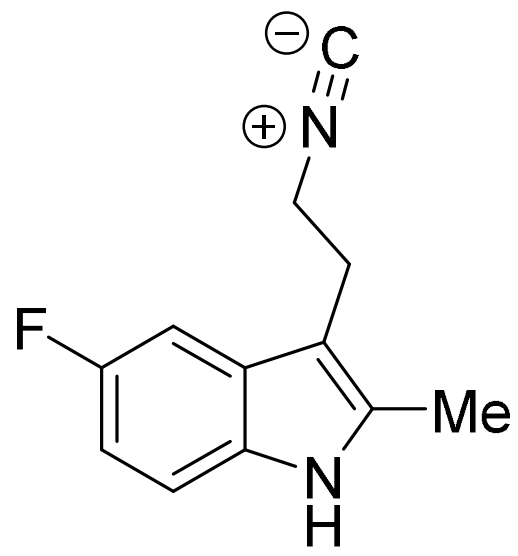

1h

White solid, 1.23 g, 61% yield, 1H NMR (400 MHz, Chloroform-*d*) δ 8.12 (s, 1H), 7.20 (dd, *J* = 8.8, 4.4 Hz, 1H), 7.12 (dd, *J* = 9.6, 2.5 Hz, 1H), 6.92 (td, *J* = 9.1, 2.5 Hz, 1H), 3.65 – 3.55 (m, 2H), 3.13 – 3.02 (m, 2H), 2.43 (s, 3H). ^{13}C NMR (101 MHz, Chloroform-*d*) δ 157.9 (d, $J_{C\text{-}F}$ = 234.1 Hz), 155.9 (t, *J* = 5.6 Hz), 135.1, 131.8, 128.4 (d, $J_{C\text{-}F}$ = 9.5 Hz), 111.2 (d, $J_{C\text{-}F}$ = 9.6 Hz), 109.3 (d, $J_{C\text{-}F}$ = 26.1 Hz), 106.8 (d, $J_{C\text{-}F}$ = 4.5 Hz), 102.4 (d, $J_{C\text{-}F}$ = 23.5 Hz), 42.0 (t, *J* = 6.4 Hz), 24.7, 11.8. ^{19}F NMR (376 MHz, Chloroform-*d*) δ -124.54. HRMS (ESI) *m/z* calcd for $C_{12}H_{11}FN_2$, $[M+H]^+$: 203.0985, found: 203.0983.

3-(2-Isocyanoethyl)-2-methyl-5-phenyl-1H-indole

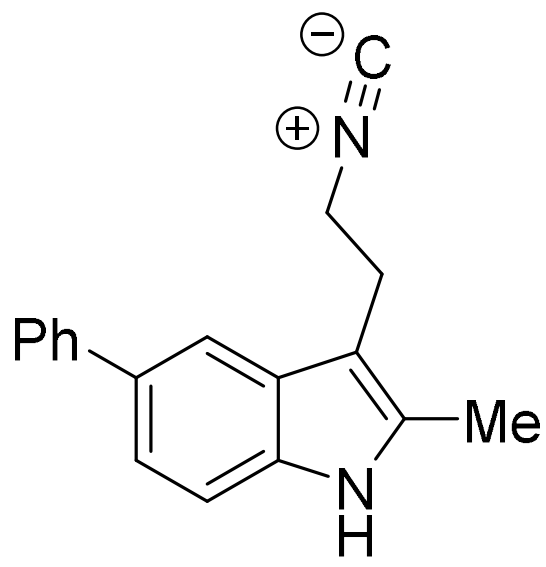

1i

White solid, 1.69 g, 65% yield, ^{1}H NMR (400 MHz, Chloroform-*d*) δ 7.97 (s, 1H), 7.73 – 7.62 (m, 3H), 7.53 – 7.42 (m, 3H), 7.41 – 7.33 (m, 2H), 3.65 (t, *J* = 7.0 Hz, 2H), 3.19 (t, *J* = 7.0 Hz, 2H), 2.48 (s, 3H). ^{13}C NMR (101 MHz, Chloroform-*d*) δ 156.1 (t, *J* = 4.4 Hz), 142.6, 134.8, 133.7, 133.3, 128.7, 128.5, 127.4, 126.5, 121.3, 115.9, 110.8, 107.0, 42.1 (t, *J* = 6.5 Hz), 24.9, 11.9. HRMS (ESI) *m/z* calcd for $C_{18}H_{16}N_2$, $[M+H]^+$: 261.1392, found: 261.1396.

Methyl 3-(2-isocyanoethyl)-2-methyl-1H-indole-5-carboxylate

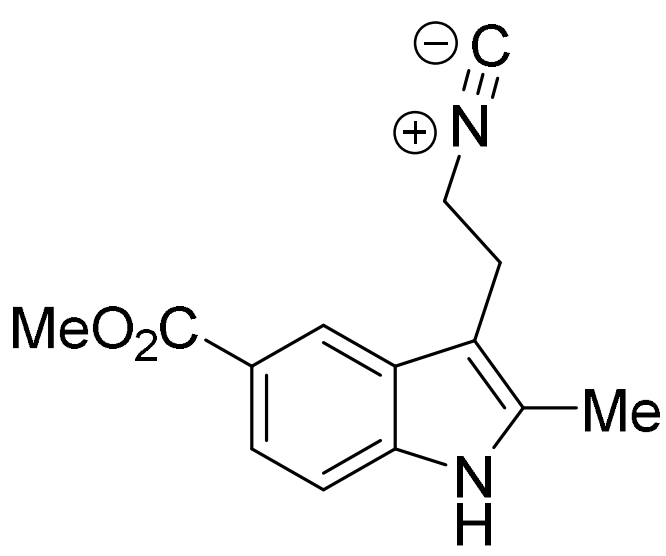

1j

White solid, 1.33 g, 55% yield, ^{1}H NMR (400 MHz, Chloroform-*d*) δ 8.45 (s, 1H), 8.21 (s, 1H), 7.87 (d, *J* = 10.1 Hz, 1H), 7.30 (d, *J* = 8.5 Hz, 1H), 3.96 (s, 3H), 3.62 (t, *J* = 6.8 Hz, 2H), 3.14 (t, *J* = 6.8 Hz, 2H), 2.46 (s, 3H). ^{13}C NMR (101 MHz, Chloroform-*d*) δ 168.3, 156.1 (t, *J* = 5.9 Hz), 138.1, 134.6, 127.6, 122.9, 121.5, 120.1, 110.2,

107.8, 52.0, 42.1 (t, J = 6.3 Hz), 24.6, 11.8. HRMS (ESI) m/z calcd for $C_{14}H_{14}N_2O_2$, $[M+H]^+$: 243.1134, found: 243.1134.

3-(2-Isocyanoethyl)-2-phenyl-1H-indole

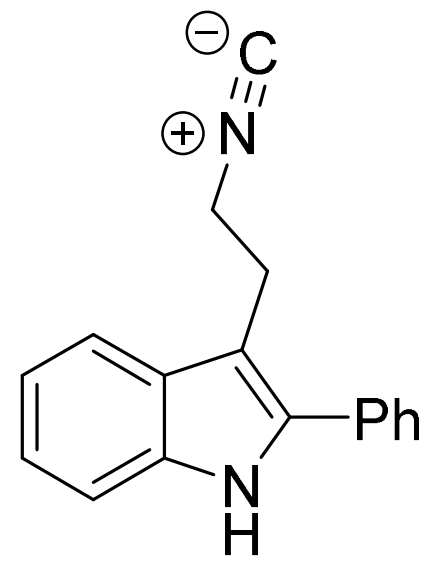

1k

White solid, 1.27 g, 52% yield, ^1H NMR (400 MHz, Chloroform-d) δ 8.38 (s, 1H), 7.67 (d, J = 7.9 Hz, 1H), 7.65 – 7.42 (m, 6H), 7.36 – 7.22 (m, 2H), 3.68 (t, J = 7.7 Hz, 2H), 3.39 (t, J = 7.6 Hz, 2H). ^{13}C NMR (101 MHz, Chloroform-d) δ 156.0 (t, J = 5.2 Hz), 136.2, 135.9, 132.5, 129.2, 128.4, 128.33, 128.31, 122.7, 120.6, 118.5, 111.4, 107.5, 41.9 (t, J = 6.7 Hz), 25.4. HRMS (ESI) m/z calcd for $C_{17}H_{14}N_2$, $[M+H]^+$: 247.1235, found: 247.1238.

Ethyl 2-(4-(3-(2-isocyanoethyl)-2-methyl-1H-indol-5-yl)phenyl)-2-methylpropanoate

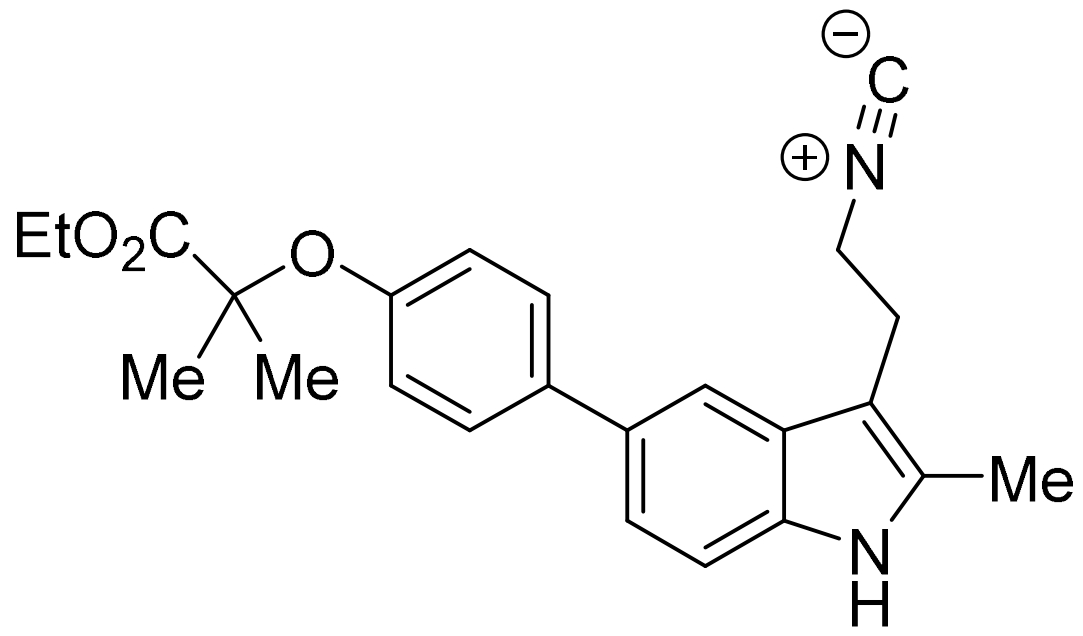

1l

White solid, 1.87 g, 50% yield, 1H NMR (400 MHz, Chloroform-d) δ 8.19 (s, 1H), 7.60 (s, 1H), 7.56 (d, J = 8.7 Hz, 2H), 7.36 (dd, J = 8.4, 1.5 Hz, 1H), 7.29 (d, J = 9.3 Hz, 1H), 6.98 (d, J = 8.7 Hz, 2H), 4.33 (q, J = 7.1 Hz, 2H), 3.62 (t, J = 6.9 Hz, 2H), 3.15 (t, J = 6.8 Hz, 2H), 2.43 (s, 3H), 1.69 (s, 6H), 1.33 (t, J = 7.1 Hz, 3H). ^{13}C NMR (101 MHz, Chloroform-d) δ 174.6, 156.0 (t, J = 4.9 Hz), 154.2, 136.7, 134.7, 133.7, 132.5, 128.4, 128.0, 120.9, 119.6, 115.4, 110.8, 106.8, 79.3, 61.6, 42.1 (t, J = 6.3 Hz), 25.5, 24.9, 14.2, 11.8. HRMS (ESI) m/z calcd for $C_{24}H_{26}N_2O_3$, $[M+H]^+$: 391.2022, found: 391.2024.

Isopropyl 2-(4-(4-(3-(2-isocyanoethyl)-2-methyl-1H-indol-5-yl)benzoyl)phenoxy)-2-methylpropanoate

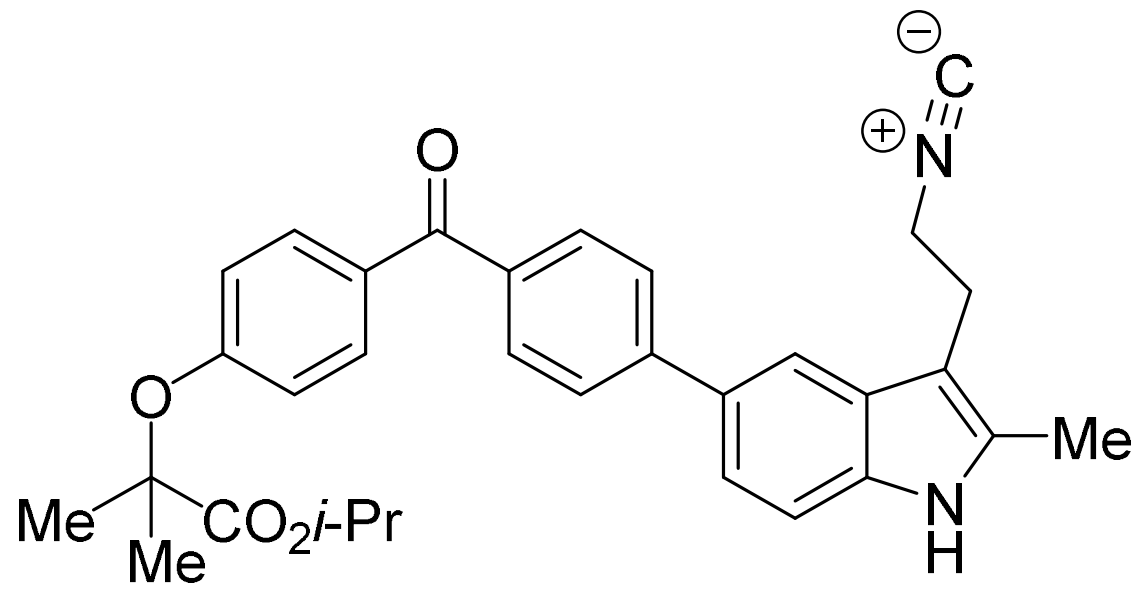

1m

Yellow solid, 2.43 g, 48% yield, ^{1}H NMR (400 MHz, Chloroform-*d*) δ 8.14 (s, 1H), 7.90 – 7.82 (m, 4H), 7.76 (d, *J* = 8.4 Hz, 2H), 7.71 (s, 1H), 7.46 (dd, *J* = 8.4, 1.6 Hz, 1H), 7.39 (d, *J* = 8.4 Hz, 1H), 6.91 (d, *J* = 8.8 Hz, 2H), 5.17 – 5.09 (m, 1H), 3.66 (t, *J* = 7.0 Hz, 2H), 3.19 (t, *J* = 7.1 Hz, 2H), 2.50 (s, 3H), 1.70 (s, 6H), 1.24 (d, *J* = 6.2 Hz, 6H). ^{13}C NMR (101 MHz, Chloroform-*d*) δ 195.4, 173.3, 159.4, 156.3 (t, *J* = 5.8 Hz), 146.3, 135.9, 135.3, 134.0, 132.02, 131.96, 131.0, 130.5, 128.6, 127.0, 121.2, 117.2, 116.2, 111.0, 107.2, 79.4, 69.4, 42.1 (t, *J* = 6.2 Hz), 25.4, 24.8, 21.6, 11.9. HRMS (ESI) *m/z* calcd for $C_{32}H_{32}N_2O_4$, $[M+H]^+$: 509.2440, found: 509.2440.

Heptan-2-yl 2-((5-(3-(2-isocyanoethyl)-2-methyl-1H-indol-5-yl)quinolin-8-yl)oxy)acetate

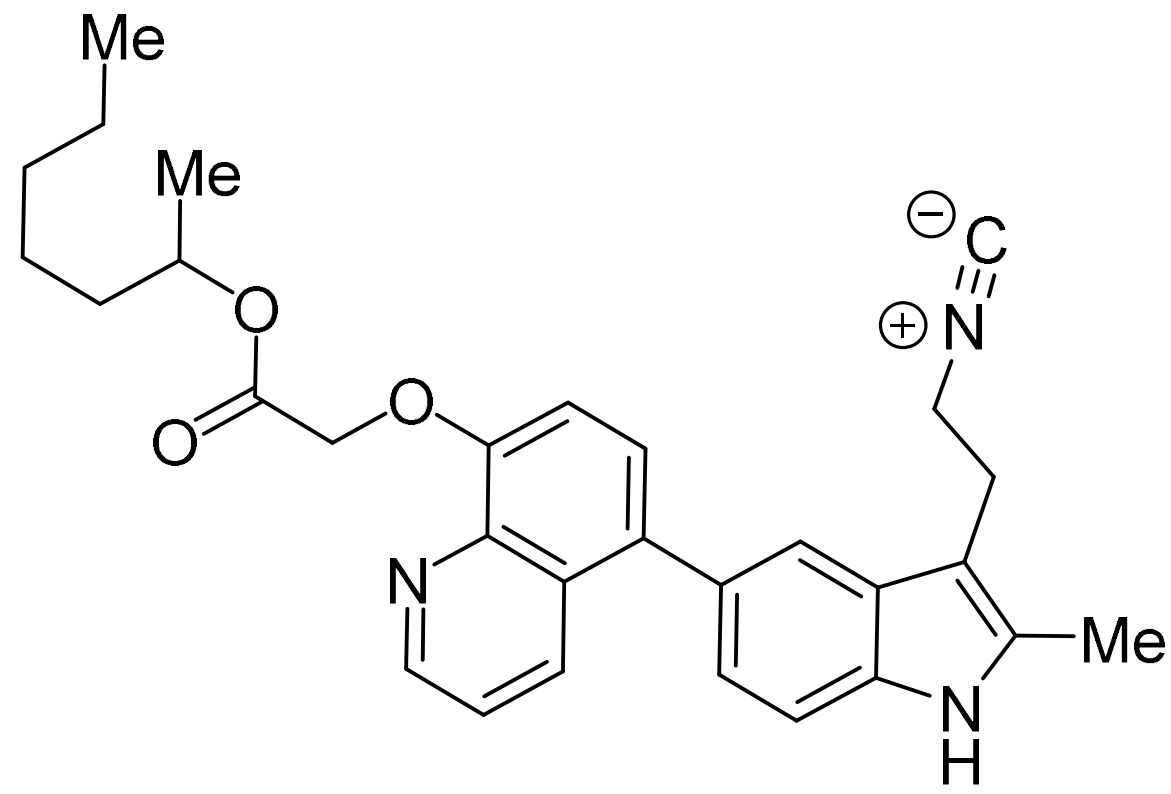

1n

Yellow solid, 2.89 g, 60% yield, ^{1}H NMR (400 MHz, Chloroform-*d*) δ 8.98 (dd, *J* = 4.1, 1.7 Hz, 1H), 8.30 (dd, *J* = 8.5, 1.7 Hz, 1H), 8.23 (s, 1H), 7.49 – 7.43 (m, 2H), 7.43 – 7.36 (m, 2H), 7.20 (dd, *J* = 8.2, 1.6 Hz, 1H), 7.04 (d, *J* = 8.0 Hz, 1H), 5.12 – 5.03 (m, 1H), 5.01 (s, 2H), 3.62 (t, *J* = 6.8 Hz, 2H), 3.15 (t, *J* = 6.8 Hz, 2H), 2.52 (s, 3H), 1.70 – 1.56 (m, 1H), 1.56 – 1.41 (m, 1H), 1.33 – 1.13 (m, 9H), 0.91 – 0.84 (m, 3H). ^{13}C NMR (101 MHz, Chloroform-*d*) δ 168.7, 156.3 (t, *J* = 5.6 Hz), 152.7, 149.1, 140.2, 134.9, 134.8, 134.7, 133.8, 130.8, 128.4, 128.2, 127.0, 123.9, 121.5, 118.8, 110.3, 108.9, 106.8, 72.6, 66.4, 42.1 (t, *J* = 5.8 Hz), 35.8, 31.5, 25.0, 24.8, 22.5, 20.0, 14.0, 11.9. HRMS (ESI) *m/z* calcd for $C_{30}H_{33}N_3O_3$, $[M+H]^+$: 484.2600, found: 484.2603.

2-(6-(*tert*-Butyl)-1,1-dimethyl-2,3-dihydro-1H-inden-4-yl)-3-(2-isocyanoethyl)-1H-indole

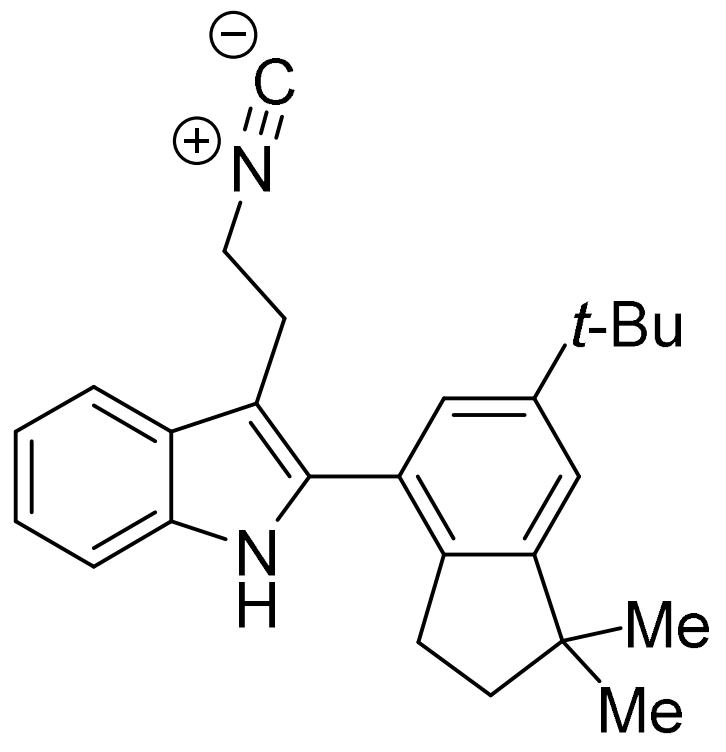

1o

Yellow solid, 1.77 g, 48% yield, ^{1}H NMR (400 MHz, Chloroform-*d*) δ 8.08 (s, 1H), 7.63 (d, J = 8.1 Hz, 1H), 7.42 (d, J = 7.8 Hz, 1H), 7.31 – 7.28 (m, 2H), 7.27 – 7.18 (m, 2H), 3.64 (t, J = 7.7 Hz, 2H), 3.24 (t, J = 7.7 Hz, 2H), 2.84 (t, J = 7.1 Hz, 2H), 1.97 (t, J = 7.1 Hz, 2H), 1.41 (s, 9H), 1.36 (s, 6H). ^{13}C NMR (101 MHz, Chloroform-*d*) δ 156.0 (t, J = 5.1 Hz), 153.5, 150.3, 139.1, 136.4, 135.6, 127.84, 127.77, 125.0, 122.3, 119.9, 119.4, 118.3, 111.1, 107.7, 44.2, 41.8 (t, J = 6.0 Hz), 41.6, 34.8, 31.6, 29.3, 28.6, 25.6. HRMS (ESI) *m/z* calcd for $C_{26}H_{30}N_2$, $[M+H]^+$: 371.2487, found: 371.2486.

3-(2-Isocyanoethyl)-2-methyl-1H-indol-5-yl palmitate

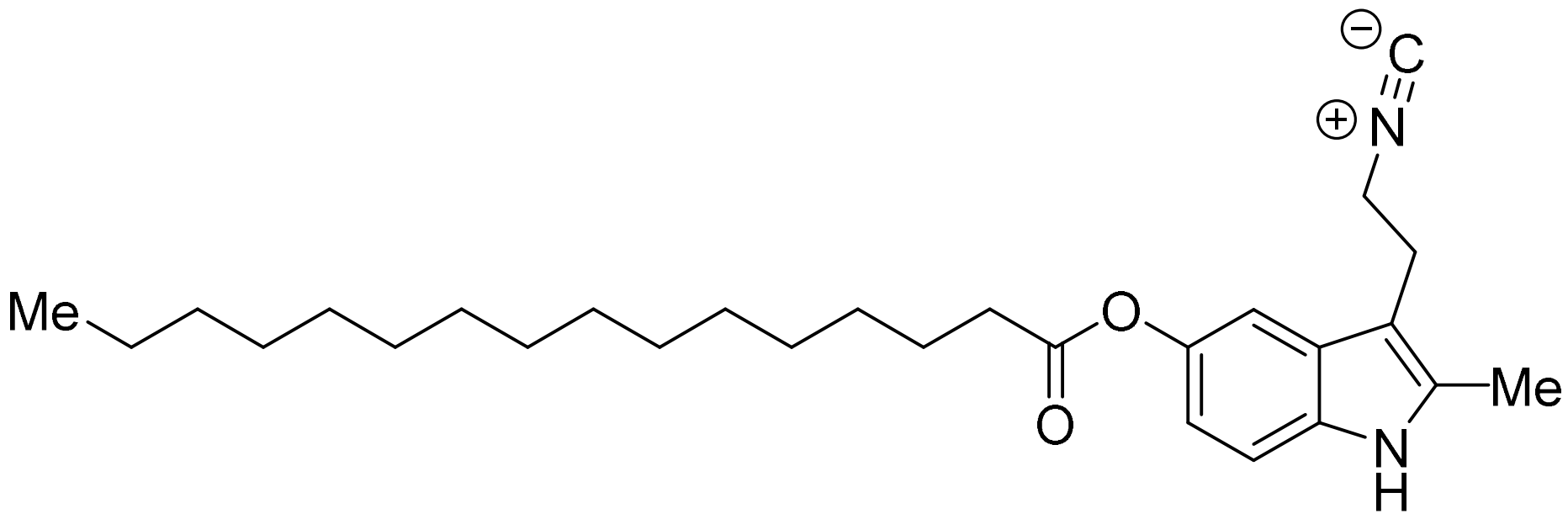

1p

Colorless oil, 2.93 g, 67% yield, ^{1}H NMR (400 MHz, Chloroform-*d*) δ 7.95 (s, 1H), 7.24 (d, J = 8.6 Hz, 1H), 7.12 (s, 1H), 6.85 (d, J = 10.8 Hz, 1H), 3.58 (t, J = 7.0 Hz, 2H), 3.09 (t, J = 7.0 Hz, 2H), 2.60 (t, J = 7.6 Hz, 2H), 2.45 (s, 3H), 1.80 (p, J = 7.5 Hz, 2H), 1.48 – 1.27 (m, 24H), 0.90 (t, J = 7.1 Hz, 3H). ^{13}C NMR (101 MHz, Chloroform-*d*) δ 173.3, 156.2 (t, J = 6.1 Hz), 144.4, 134.3, 133.0, 128.3, 115.4, 110.8, 109.5, 107.0, 41.9 (d, J = 6.2 Hz), 34.5, 32.0, 29.7 (4C), 29.69, 29.65, 29.5, 29.4, 29.3, 29.2, 25.1, 24.9, 22.7, 14.2, 11.9. HRMS (ESI) *m/z* calcd for $C_{28}H_{42}N_2O_2$, $[M+H]^+$: 439.3325, found: 439.3328.

3-(2-Isocyanoethyl)-2-methyl-1H-indol-5-yl

2-(4-(2-(4-Chlorobenzamido)ethyl)phenoxy)-2-methylpropanoate

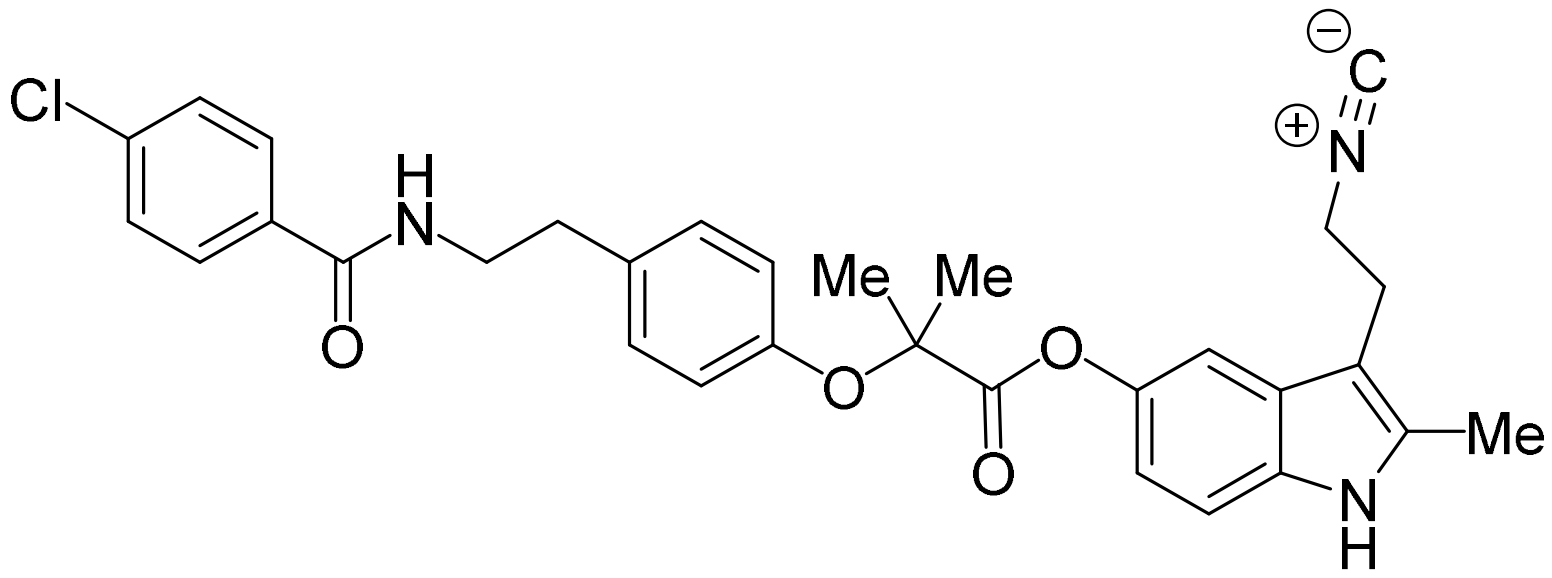

1q

Yellow solid, 2.44 g, 45% yield, ^{1}H NMR (400 MHz, Chloroform-*d*) δ 8.04 (s, 1H), 7.61 (d, *J* = 8.5 Hz, 2H), 7.34 (d, *J* = 8.5 Hz, 2H), 7.22 – 7.15 (m, 3H), 7.05 (d, *J* = 2.2 Hz, 1H), 6.99 (d, *J* = 8.5 Hz, 2H), 6.75 (dd, *J* = 8.6, 2.2 Hz, 1H), 6.18 (t, *J* = 6.0 Hz, 1H), 3.75 – 3.65 (m, 2H), 3.55 (t, *J* = 7.2 Hz, 2H), 3.06 (t, *J* = 7.2 Hz, 2H), 2.92 (t, *J* = 6.9 Hz, 2H), 2.44 (s, 3H), 1.80 (s, 6H). ^{13}C NMR (101 MHz, $CDCl_3$) δ 173.8, 166.4, 156.1 (t, *J* = 5.3 Hz), 154.3, 144.2, 137.6, 134.5, 133.1, 133.0, 132.6, 129.6, 128.8, 128.3 (2C), 119.5, 114.7, 110.9, 109.2, 107.0, 79.4, 42.0 (t, *J* = 6.3 Hz), 41.3, 34.8, 25.6, 24.8, 11.9. HRMS (ESI) *m/z* calcd for $C_{31}H_{30}ClN_3O_4$, $[M+H]^+$: 544.2003, found: 544.2006.

3-(2-Isocyanoethyl)-2-methyl-1H-indol-5-yl

2-(1-(4-chlorobenzoyl)-5-methoxy-2-methyl-1H-indol-3-yl)acetate

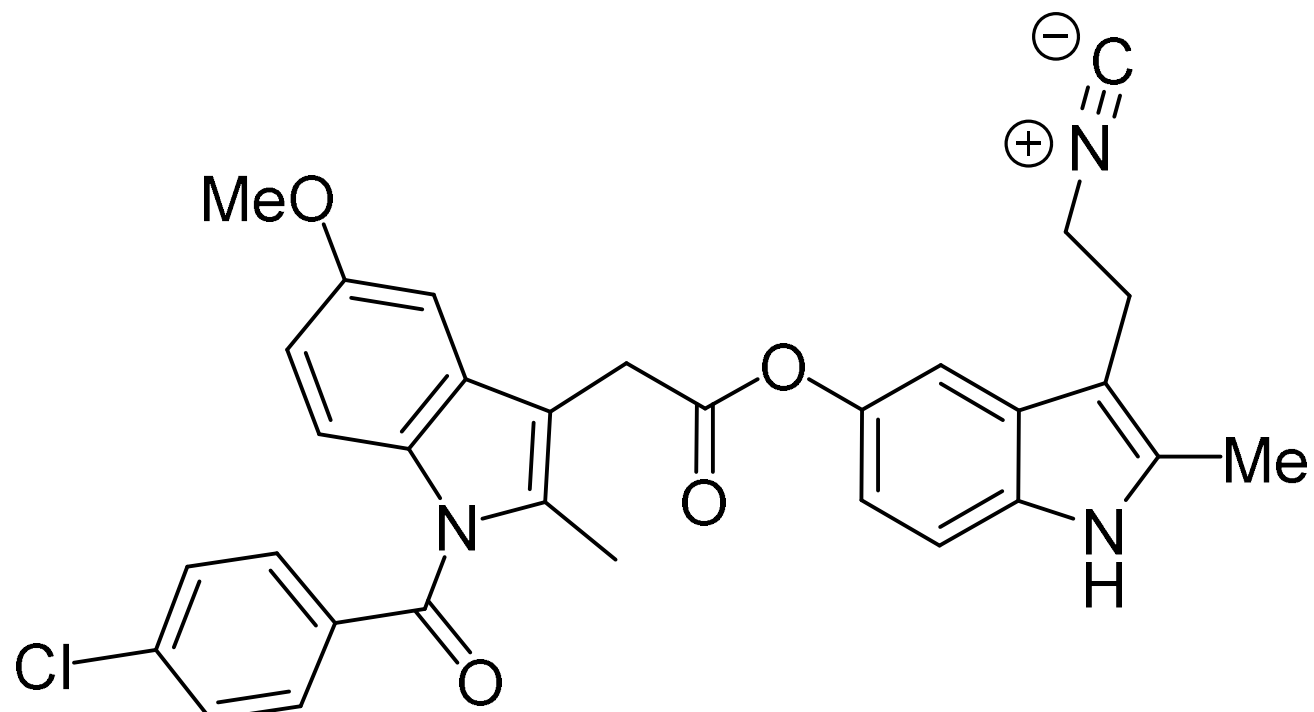

1r

Yellow solid, 2.04 g, 38% yield, ^{1}H NMR (400 MHz, Chloroform-*d*) δ 8.00 (s, 1H), 7.70 (d, *J* = 8.5 Hz, 2H), 7.50 (d, *J* = 8.5 Hz, 2H), 7.19 (d, *J* = 8.7 Hz, 1H), 7.12 (dd, *J* = 9.7, 2.4 Hz, 2H), 6.95 (d, *J* = 9.0 Hz, 1H), 6.80 (dd, *J* = 8.7, 2.2 Hz, 1H), 6.73 (dd, *J* = 9.0, 2.5 Hz, 1H), 3.95 (s, 2H), 3.87 (s, 3H), 3.55 (t, *J* = 7.0 Hz, 2H), 3.05 (t, *J* = 6.9 Hz, 2H), 2.49 (s, 3H), 2.41 (s, 3H). ^{13}C NMR (101 MHz, Chloroform-*d*) δ 170.3, 168.4, 156.2 (t, *J* = 5.7 Hz), 156.1, 144.3, 139.4, 136.2, 134.5, 133.9, 133.1, 131.2, 130.9, 130.7, 129.2, 128.2, 115.1, 115.0, 112.4, 111.9,

110.9, 109.4, 107.0, 101.4, 55.8, 41.9 (t, J = 6.6 Hz), 30.7, 24.8, 13.6, 11.9. HRMS (ESI) m/z calcd for $C_{31}H_{26}ClN_3O_4$, $[M+H]^+$: 540.1690, found: 540.1695.

3-(2-Isocyanobenzyl)-2-methyl-1H-indole

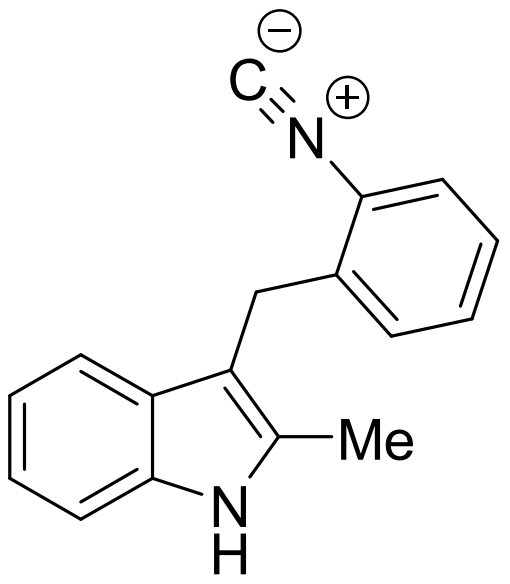

4a

Colorless oil, 787.2 mg, 32% yield, ^1H NMR (400 MHz, Chloroform-d) δ 7.93 (s, 1H), 7.43 (dd, J = 5.8, 3.5 Hz, 1H), 7.40 – 7.32 (m, 2H), 7.25 – 7.16 (m, 3H), 7.13 – 7.06 (m, 2H), 4.24 (s, 2H), 2.43 (s, 3H). ^{13}C NMR (101 MHz, Chloroform-d) δ 166.2, 137.9, 135.4, 132.6, 129.6, 129.4, 128.7, 126.8, 126.7, 126.0, 121.3, 119.5, 118.2, 110.4, 107.9, 26.5, 11.9. HRMS (ESI) m/z calcd for $C_{17}H_{14}N_2$, $[M+H]^+$: 247.1235, found: 247.1234.

5-Chloro-3-(2-isocyanobenzyl)-2-methyl-1H-indole

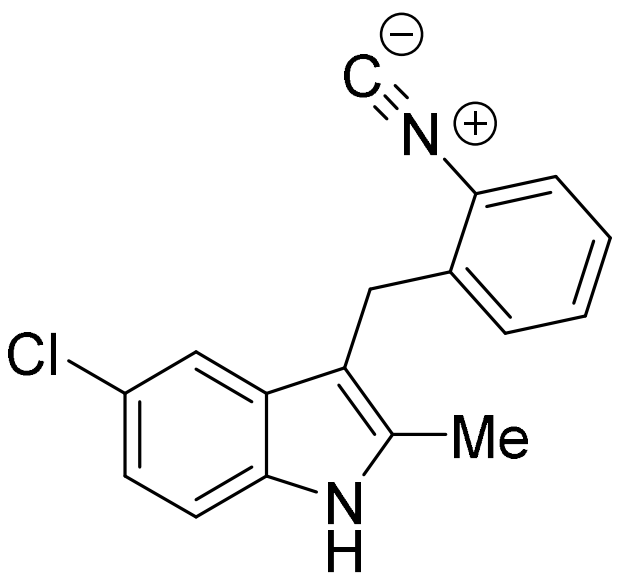

4b

White solid, 1.04 g, 37% yield, ^1H NMR (400 MHz, Chloroform-d) δ 8.04 (s, 1H), 7.42 (dd, J = 5.7, 3.5 Hz, 1H), 7.31 – 7.19 (m, 4H), 7.12 – 7.00 (m, 2H), 4.16 (s, 2H), 2.40 (s, 3H). ^{13}C NMR (101 MHz, Chloroform-d) δ 166.3, 137.3, 134.3, 133.7, 129.8, 129.5, 129.3, 126.98, 126.96, 126.8, 126.0, 125.2, 121.4, 117.6, 111.4, 107.7, 26.4, 11.9. HRMS (ESI) m/z calcd for $C_{17}H_{13}ClN_2$, $[M+H]^+$: 281.0846, found: 281.0843.

5-Bromo-3-(2-isocyanobenzyl)-2-methyl-1H-indole

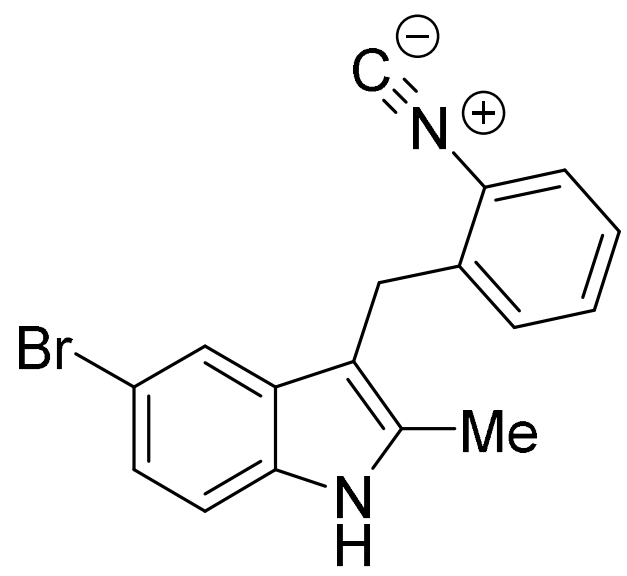

4c

White solid, 1.32 g, 41% yield, ^{1}H NMR (400 MHz, Chloroform-*d*) δ 8.02 (s, 1H), 7.46 – 7.37 (m, 2H), 7.27 – 7.15 (m, 4H), 7.02 (dd, *J* = 5.5, 3.7 Hz, 1H), 4.15 (s, 2H), 2.40 (s, 3H). ^{13}C NMR (101 MHz, Chloroform-*d*) δ 166.4, 137.3, 134.1, 134.0, 130.5, 129.5, 129.3, 127.0, 126.8, 126.0, 124.1, 120.7, 112.8, 111.8, 107.6, 26.3, 11.9. HRMS (ESI) *m/z* calcd for $C_{17}H_{13}BrN_2$, $[M+H]^+$: 325.0340, found: 325.0345.

3-(2-Isocyano-5-methoxybenzyl)-2-methyl-1H-indole

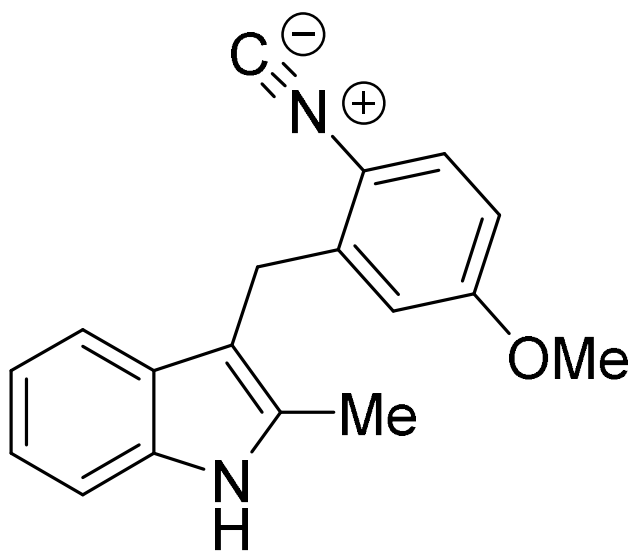

4d

Colorless oil, 910.8 mg, 33% yield, ^{1}H NMR (400 MHz, Chloroform-*d*) δ 8.04 (s, 1H), 7.43 – 7.35 (m, 2H), 7.32 (d, *J* = 8.0 Hz, 1H), 7.21 – 7.14 (m, 1H), 7.13 – 7.06 (m, 1H), 6.72 (dd, *J* = 8.7, 2.8 Hz, 1H), 6.63 (d, *J* = 2.8 Hz, 1H), 4.20 (s, 2H), 3.66 (s, 3H), 2.41 (s, 3H). ^{13}C NMR (101 MHz, Chloroform-*d*) δ 164.9, 160.0, 139.8, 135.5, 132.6, 128.7, 128.1, 121.2, 119.5, 119.0, 118.1, 115.4, 111.5, 110.5, 107.7, 55.4, 26.6, 11.9. HRMS (ESI) *m/z* calcd for $C_{18}H_{16}N_2O$, $[M+H]^+$: 277.1341, found: 277.1340.

3-(2-Isocyano-5-methylbenzyl)-2-methyl-1H-indole

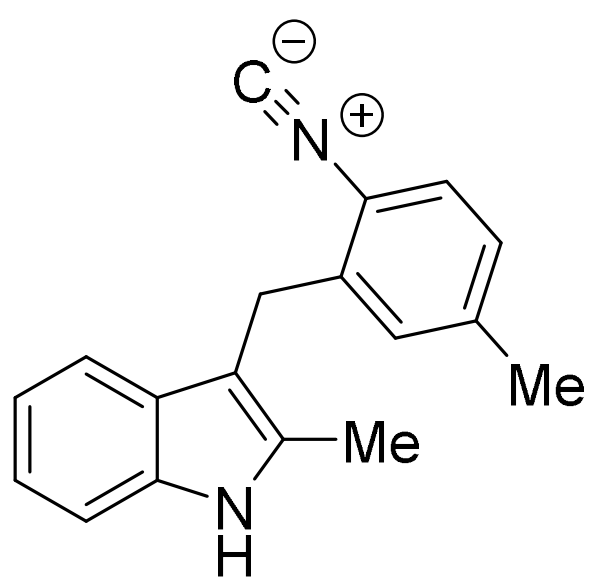

4e

Colorless oil, 1.04 g, 40% yield, ^{1}H NMR (400 MHz, Chloroform-*d*) δ 7.82 (s, 1H), 7.48 – 7.37 (m, 1H), 7.32 – 7.18 (m, 3H), 7.16 – 7.07 (m, 2H), 6.94 (d, *J* = 8.2 Hz, 1H), 4.23 (s, 2H), 2.51 (s, 3H), 2.41 (s, 3H). ^{13}C NMR (101 MHz, $CDCl_3$) δ 166.2, 138.0, 135.9, 131.9, 131.0, 129.6, 129.4, 126.7, 126.7, 126.6, 126.0, 121.1, 117.9, 110.6, 107.6, 26.5, 21.8, 11.9. HRMS (ESI) *m/z* calcd for $C_{18}H_{16}N_2$, $[M+H]^+$: 261.1392, found: 261.1391.

3-(5-Bromo-2-isocyanobenzyl)-2-methyl-1H-indole

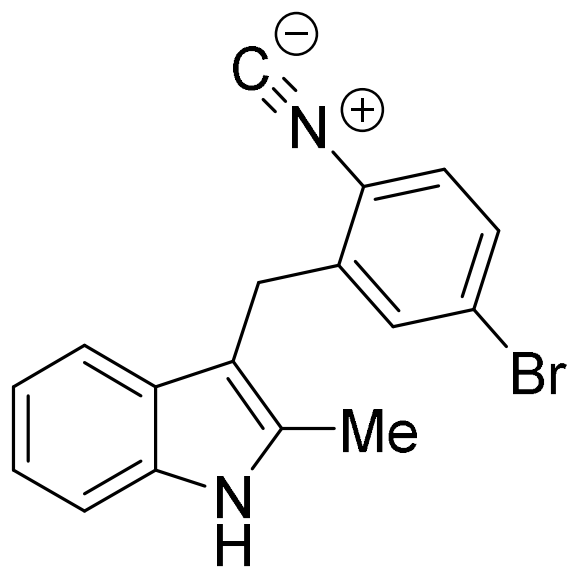

4f

White solid, 1.26 g, 39% yield, ^{1}H NMR (400 MHz, DMSO-*d*$_6$) δ 11.18 (s, 1H), 7.52 (d, *J* = 7.7 Hz, 1H), 7.41 (s, 1H), 7.38 – 7.23 (m, 3H), 7.11 (t, *J* = 8.1 Hz, 2H), 4.11 (s, 2H), 2.37 (s, 3H). ^{13}C NMR (101 MHz, DMSO-*d*$_6$) δ 167.1, 138.1, 135.5, 134.5, 130.5, 130.2, 130.0, 127.7, 127.3, 125.6, 123.0, 120.2, 113.0, 111.6, 107.0, 26.3, 12.0. HRMS (ESI) *m/z* calcd for $C_{17}H_{13}BrN_2$, $[M+H]^+$: 325.0340, found: 325.0340.

6,6',6'',6'''-(Pyrene-1,3,6,8-tetrayl)tetrakis(naphthalen-2-amine)

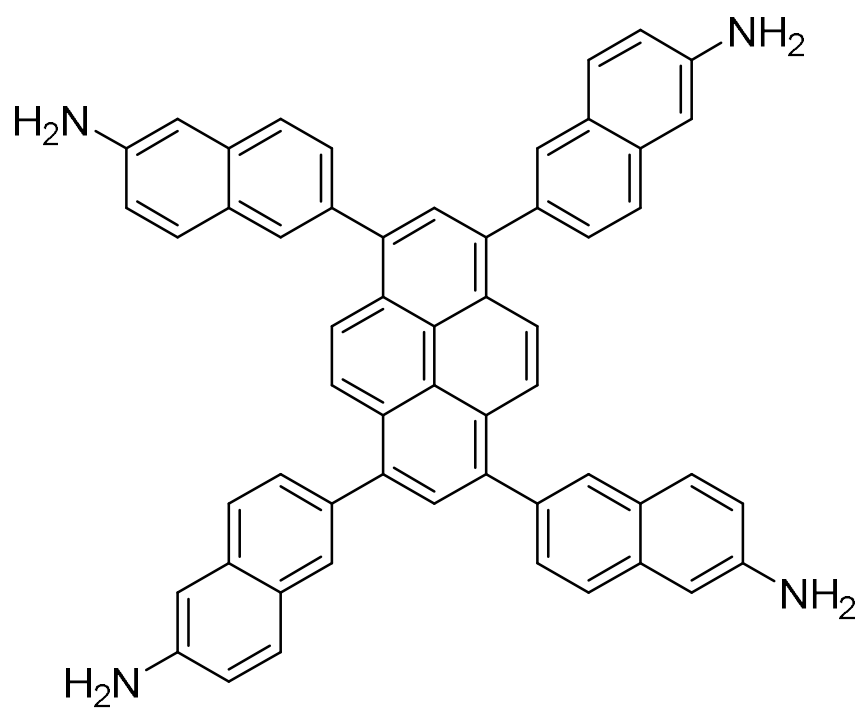

Yellow solid, 551.5 mg, 72% yield, ^{1}H NMR (400 MHz, DMSO-*d*$_6$) δ 8.23 (s, 4H), 8.10 (s, 2H), 7.99 (s, 4H), 7.81 – 7.53 (m, 12H), 7.11 – 6.83 (m, 8H), 5.52 (s, 8H). ^{13}C NMR (101 MHz, DMSO-*d*$_6$) 147.7, 137.7, 134.8, 133.1, 130.4, 129.6, 129.5, 129.1, 127.7, 126.9, 126.3, 125.7, 125.5, 119.4, 106.0. HRMS (ESI) *m/z* calcd for $C_{56}H_{38}N_4$, $[M+Na]^+$: 789.2994, found: 789.2997.

(1*E*,1'*E*,1''*E*,1'''*E*)-N,N',N'',N'''-(Pyrene-1,3,6,8-tetrayltetrakis(naphthalene-6,2-diyl))tetrakis(1-(2-methoxy phenyl)methanimine) NPy-MP

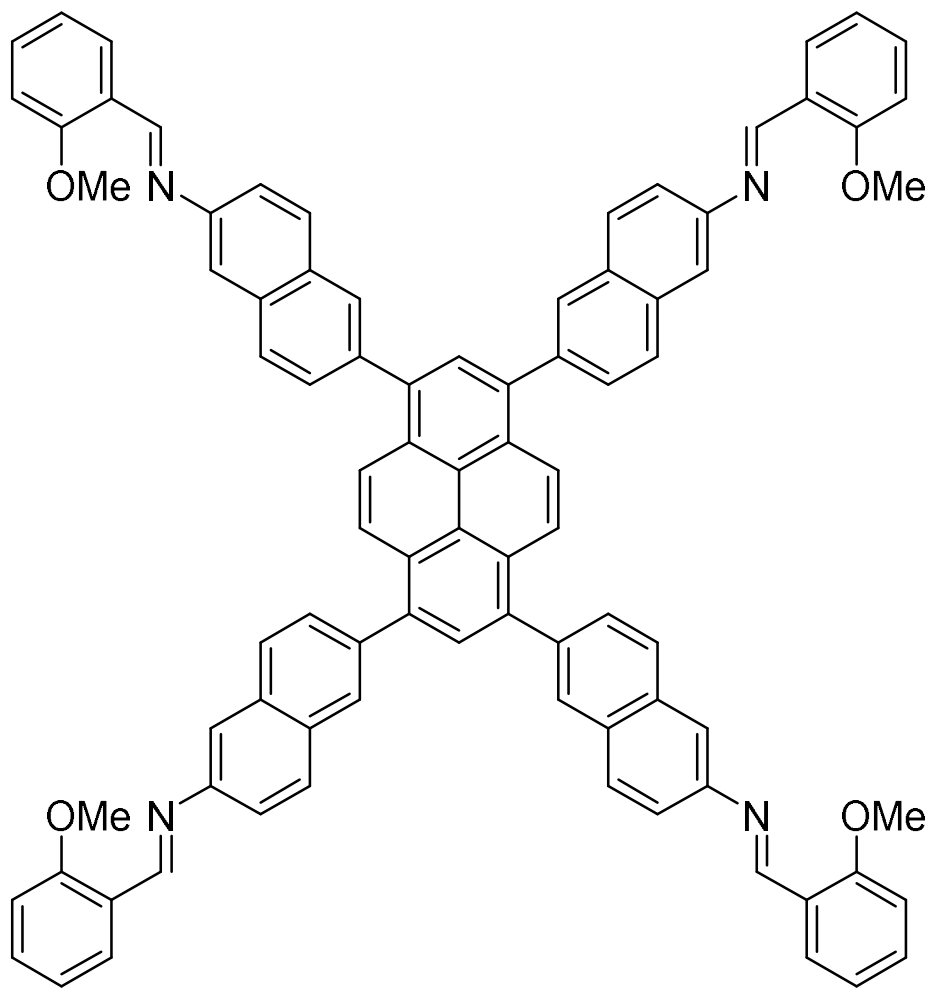

Yellow solid, 45.7 mg, 74% yield, ^{1}H NMR (400 MHz, Chloroform-*d*) δ 9.13 (s, 4H), 8.41 – 7.38 (m, 38H), 7.19 – 6.86 (m, 8H), 3.94 (s, 12H). ^{13}C NMR (101 MHz, Chloroform-*d*) δ 159.6, 156.7, 150.6, 138.0, 137.3, 133.3, 132.9, 132.0, 129.5, 129.4, 129.1, 128.4, 127.9, 127.6 (2C), 125.6, 124.8, 122.1, 121.0 (2C), 117.9, 111.2, 55.6. HRMS (ESI) *m/z* calcd for $C_{88}H_{62}N_4O_4$, $[M+Na]^+$: 1239.4849, found: 1239.4839.

***N*-(2-(3-(Diphenylphosphoryl)-2-oxopyrrolidin-3-yl)phenyl)acetamide**

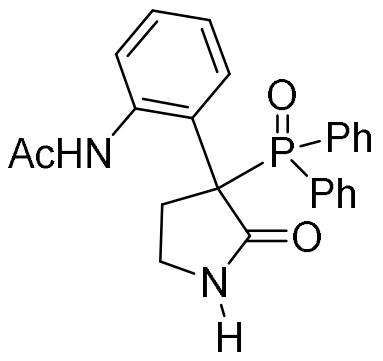

3a

White solid, 90.2 mg, 72% yield, ^{1}H NMR (400 MHz, Chloroform-*d*) δ 11.42 (s, 1H), 8.16 – 7.95 (m, 4H), 7.81 (d, *J* = 8.2 Hz, 2H), 7.67 – 7.48 (m, 3H), 7.46 – 7.29 (m, 3H), 7.22 (t, *J* = 7.7 Hz, 1H), 6.98 (t, *J* = 7.7 Hz, 1H), 5.82 (s, 1H), 3.45 – 3.30 (m, 1H), 3.20 – 3.08 (m, 1H), 2.93 – 2.80 (m, 2H), 2.32 (s, 3H). ^{13}C NMR (101 MHz, Chloroform-*d*) δ 174.3 (d, *J* = 2.7 Hz), 169.2, 136.8 (d, *J* = 3.5 Hz), 133.0 (d, *J* = 8.9 Hz), 132.7 (d, *J* = 2.8 Hz), 132.6 (d, *J* = 9.6 Hz), 132.2 (d, *J* = 3.1 Hz), 129.2 (d, *J* = 4.3 Hz), 129.0 (d, *J* = 93.9 Hz), 128.7 (d, *J* = 103.4 Hz), 128.6 (d, *J* = 11.8 Hz), 128.5 (d, *J* = 2.5 Hz), 128.0 (d, *J* = 12.6 Hz), 127.0 (d, *J* = 2.0 Hz), 127.6 (d, *J* = 4.1 Hz), 124.6 (d, *J* = 1.4 Hz), 57.9 (d, *J* = 61.6 Hz), 39.5 (d, *J* = 4.7 Hz), 30.4, 24.4. ^{31}P NMR (162 MHz, Chloroform-*d*) δ 39.42. HRMS (ESI) *m/z* calcd for $C_{24}H_{23}N_2O_3P$, $[M+Na]^+$: 441.1344, found: 441.1348.

***N*-(2-(3-(Di-*m*-Tolylphosphoryl)-2-oxopyrrolidin-3-yl)phenyl)acetamide**

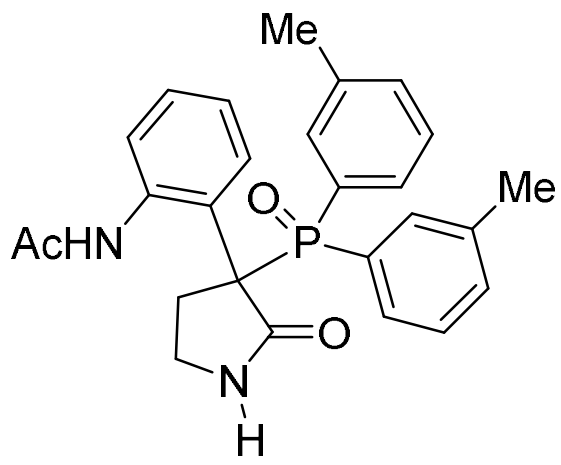

3b

^{1}H NMR (400 MHz, Chloroform-*d*) δ 11.46 (s, 1H), 7.94 – 7.78 (m, 5H), 7.65 (d, *J* = 12.1 Hz, 1H), 7.42 – 7.37 (m, 2H), 7.25 – 7.18 (m, 3H), 6.98 (t, *J* = 7.7 Hz, 1H), 5.93 (s, 1H), 3.44 – 3.26 (m, 1H), 3.16 – 3.06 (m, 1H), 2.90 – 2.80 (m, 2H), 2.44 (s, 3H), 2.32 (s, 3H), 2.24 (s, 3H). ^{13}C NMR (101 MHz, Chloroform-*d*) δ 174.3 (d, *J* = 2.6 Hz), 169.2, 138.4 (d, *J* = 11.6 Hz), 137.7 (d, *J* = 12.4 Hz), 136.9 (d, *J* = 3.6 Hz), 133.5 (d, *J* = 2.9 Hz), 133.3 (d, *J* = 8.5 Hz), 133.0 (d, *J* = 9.0 Hz), 132.9 (d, *J* = 3.0 Hz), 130.1 (d, *J* = 9.5 Hz), 129.6 (d, *J* = 10.2 Hz), 129.2 (d, *J* = 3.0 Hz), 128.8 (d, *J* = 95.4 Hz), 128.6 (d, *J* = 102.8 Hz), 128.39 (d, *J* = 12.4 Hz), 128.38 (d, *J* = 2.4 Hz), 127.9 (d, 3.1 Hz), 127.8 (d, *J* = 13.6 Hz), 127.0 (d, *J* = 1.8 Hz), 124.5 (d, *J* = 1.6 Hz), 57.9 (d, *J* = 60.8 Hz), 39.5 (d, *J* = 4.5 Hz), 30.5, 24.5, 21.6, 21.4. ^{31}P NMR (162 MHz, Chloroform-*d*) δ 39.85. HRMS (ESI) *m/z* calcd for $C_{26}H_{27}N_2O_3P$, $[M+Na]^+$: 469.1657, found: 469.1658.

***N*-(2-(3-(Bis(3,5-dimethylphenyl)phosphoryl)-2-oxopyrrolidin-3-yl)phenyl)acetamide**

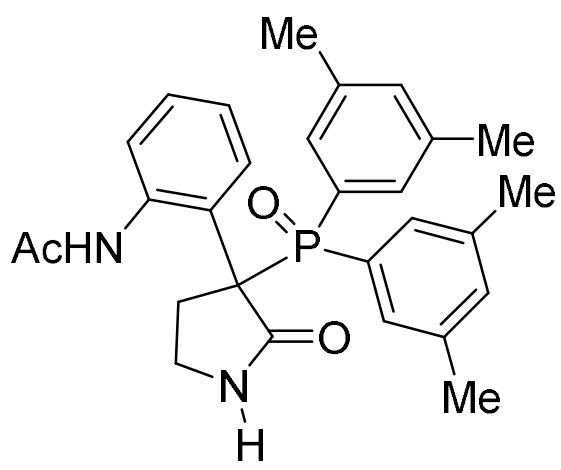

3c

White solid, 113.2 mg, 80% yield, ^{1}H NMR (400 MHz, Chloroform-*d*) δ 11.50 (s, 1H), 7.90 – 7.78 (m, 2H), 7.65 (d, *J* = 11.3 Hz, 2H), 7.50 (d, *J* = 11.9 Hz, 2H), 7.28 – 7.16 (m, 2H), 7.08 – 6.96 (m, 2H), 5.97 (s, 1H), 3.45 – 3.28 (m, 1H), 3.19 – 3.03 (m, 1H), 2.90 – 2.75 (m, 2H), 2.38 (s, 6H), 2.32 (s, 3H), 2.21 (s, 6H). ^{13}C NMR (101 MHz, Chloroform-*d*) δ 174.4 (d, *J* = 2.4 Hz), 169.2, 138.2 (d, *J* = 12.6 Hz), 137.5 (d, *J* = 13.3 Hz), 136.9 (d, *J* = 3.0 Hz), 134.5 (d, *J* = 2.8 Hz), 133.9 (d, *J* = 2.6 Hz), 130.5 (d, *J* = 9.0 Hz), 130.1 (d, *J* = 9.6 Hz), 129.1 (d, *J* = 2.2 Hz), 128.8 (d, *J* = 109.7 Hz), 128.7 (d, *J* = 109.7 Hz), 128.3 (d, *J* = 2.2 Hz), 128.1 (d, *J* = 3.3 Hz), 127.0, 124.3, 57.8 (d, *J* = 60.3 Hz), 39.5 (d, *J* = 3.9 Hz), 30.6, 24.5, 21.5, 21.3. ^{31}P NMR (162 MHz, Chloroform-*d*) δ 38.91. HRMS (ESI) *m/z* calcd for $C_{28}H_{31}N_2O_3P$, $[M+Na]^+$: 497.1971, found: 497.1975.

***N*-(2-(3-(Di-*p*-tolylphosphoryl)-2-oxopyrrolidin-3-yl)phenyl)acetamide**

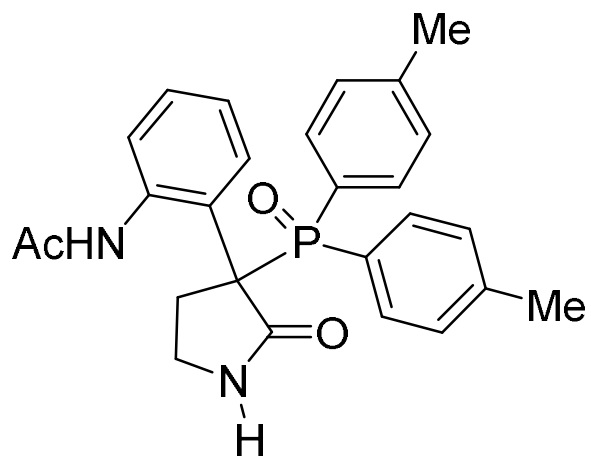

3d

White solid, 107.1 mg, 72% yield, ^{1}H NMR (400 MHz, Chloroform-*d*) δ 11.51 (s, 1H), 7.95 (dd, *J* = 10.8, 7.9 Hz, 2H), 7.90 – 7.75 (m, 4H), 7.31 (dd, *J* = 8.1, 2.9 Hz, 2H), 7.23 (t, *J* = 7.7 Hz, 1H), 7.11 (dd, *J* = 8.3, 3.2 Hz, 2H), 6.98 (t, *J* = 7.7 Hz, 1H), 5.90 (s, 1H), 3.45 – 3.25 (m, 1H), 3.16 – 3.05 (m, 1H), 2.90 – 2.75 (m, 2H), 2.42 (s, 3H), 2.35 – 2.26 (m, 6H).^{13}C NMR (101 MHz, Chloroform-*d*) δ 174.4 (d, *J* = 2.8 Hz), 169.2, 143.3 (d, *J* = 2.8 Hz), 142.6 (d, *J* = 2.8 Hz), 136.9 (d, *J* = 3.3 Hz), 133.0 (d, *J* = 9.3 Hz), 132.5 (d, *J* = 9.9 Hz), 129.3 (d, *J* = 12.2 Hz), 129.2 (d, *J* = 4.4 Hz), 128.8 (d, *J* = 12.9 Hz), 128.3 (d, *J* = 2.2 Hz), 128.0 (d, *J* = 4.4 Hz), 126.9 (d, *J* = 1.9 Hz),125.8 (d, *J* = 96.2 Hz), 125.6 (d, *J* = 105.4 Hz), 124.5, 57.9 (d, *J* = 61.5 Hz), 39.5 (d, *J* = 4.6 Hz), 30.5, 24.4, 21.7, 21.6. ^{31}P NMR (162 MHz, Chloroform-*d*) δ 40.00. HRMS (ESI) *m/z* calcd for $C_{26}H_{27}N_2O_3P$, $[M+Na]^+$: 469.1657, found: 469.1663.

N-(2-(3-(bis(4-methoxyphenyl)phosphoryl)-2-oxopyrrolidin-3-yl)-4-methylphenyl)acetamide

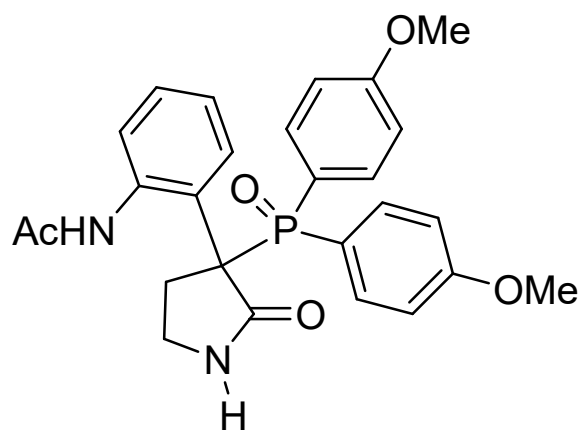

3e

White solid, 79.7 mg, 54% yield, ^{1}H NMR (400 MHz, Chloroform-*d*) δ 11.40 (s, 1H), 8.01 – 7.85 (m, 4H), 7.66 (d, *J* = 8.2 Hz, 1H), 7.58 (s, 1H), 7.10 – 6.95 (m, 3H), 6.82 (dd, *J* = 9.0, 2.6 Hz, 2H), 5.68 (s, 1H), 3.87 (s, 3H), 3.78 (s, 3H), 3.42 – 3.20 (m, 1H), 3.20 – 3.03 (m, 1H), 2.97 – 2.70 (m, 2H), 2.29 (s, 3H), 2.22 (s, 3H).^{13}C NMR (101 MHz, Chloroform-*d*) δ 174.6 (d, *J* = 2.8 Hz), 169.1, 162.9 (d, *J* = 2.9 Hz), 162.4 (d, *J* = 3.2 Hz), 134.8 (d, *J* = 10.2 Hz), 134.4 (d, *J* = 11.0 Hz), 134.14 (d, *J* = 1.2 Hz), 134.11,129.4 (d, *J* = 4.4 Hz), 128.5 (d, *J* = 100.7 Hz), 128.4 (d, *J* = 102.9 Hz), 126.9, 120.8, 119.8 (d, *J* = 8.9 Hz), 114.0 (d, *J* = 12.7 Hz), 113.5 (d, *J* = 13.7 Hz), 57.9 (d, *J* = 62.5 Hz), 55.4, 55.2, 39.5 (d, *J* = 4.5 Hz), 30.4, 24.4, 21.1. ^{31}P NMR (162 MHz, Chloroform-*d*) δ 39.63. HRMS (ESI) *m/z* calcd for $C_{27}H_{29}N_2O_5P$, $[M+Na]^+$: 515.1712, found: 515.1713.

N-(2-(3-(Bis(4-fluorophenyl)phosphoryl)-2-oxopyrrolidin-3-yl)phenyl)acetamide

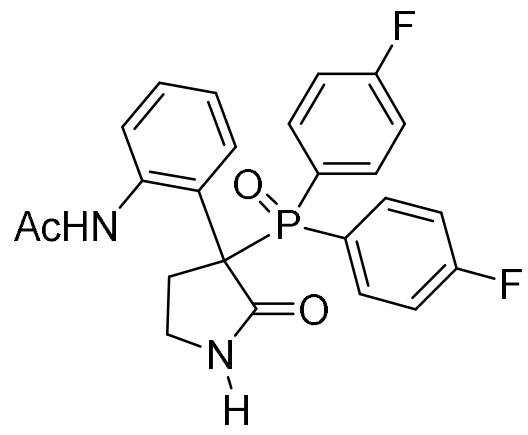

3f

White solid, 81.2 mg, 80% yield, ^{1}H NMR (400 MHz, Chloroform-*d*) δ 11.28 (s, 1H), 8.12 – 8.00 (m, 4H), 7.81 (d, J = 8.0 Hz, 1H), 7.71 (d, J = 8.2 Hz, 1H), 7.25 – 7.15 (m, 3H), 7.05 – 6.95 (m, 3H), 5.96 (s, 1H), 3.40 – 3.10 (m, 2H), 3.00 – 2.80 (m, 2H), 2.30 (s, 3H). ^{13}C NMR (101 MHz, Chloroform-*d*) δ 174.3 (d, $J_{C\text{-}P}$ = 2.8 Hz), 169.1, 165.6 (dd, $J_{C\text{-}P}$ = 3.4 Hz, $J_{C\text{-}F}$ = 256.7 Hz), 165.15 (dd, $J_{C\text{-}P}$ = 3.7 Hz, $J_{C\text{-}F}$ = 256.0 Hz), 136.7 (d, $J_{C\text{-}P}$ = 3.4 Hz), 135.6 (dd, $J_{C\text{-}P}$ = 10.4 Hz, $J_{C\text{-}F}$ = 9.1 Hz), 135.2 (dd, $J_{C\text{-}P}$ = 11.2 Hz, $J_{C\text{-}F}$ = 8.9 Hz), 129.1 (d, $J_{C\text{-}P}$ = 4.7 Hz), 128.7 (d, $J_{C\text{-}P}$ = 2.4 Hz), 127.1 (d, $J_{C\text{-}P}$ = 2.0 Hz), 126.8 (d, $J_{C\text{-}P}$ = 4.2 Hz), 124.74, 124.69 (dd, $J_{C\text{-}P}$ = 97.5 Hz, $J_{C\text{-}F}$ = 3.8 Hz), 124.4 (dd, $J_{C\text{-}P}$ = 107.2 Hz, $J_{C\text{-}F}$ = 3.2 Hz), 116.1 (dd, $J_{C\text{-}P}$ = 13.0 Hz, $J_{C\text{-}F}$ = 21.3 Hz), 115.6 (dd, $J_{C\text{-}P}$ = 13.8 Hz, $J_{C\text{-}F}$ = 21.3 Hz), 58.3 (d, $J_{C\text{-}P}$ = 63.9 Hz), 39.6 (d, $J_{C\text{-}P}$ = 5.3 Hz), 30.1, 24.4.^{19}F NMR (376 MHz, Chloroform-*d*) δ -104.91, -105.58. ^{31}P NMR (162 MHz, Chloroform-*d*) δ 38.76. HRMS (ESI) *m/z* calcd for $C_{24}H_{21}F_2N_2O_3P$, $[M+Na]^+$: 477.1516, found: 477.1516.

***N*-(2-(3-(Bis(4-chlorophenyl)phosphoryl)-2-oxopyrrolidin-3-yl)phenyl)acetamide**

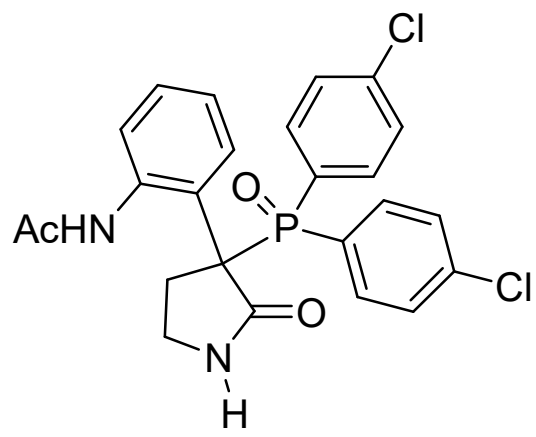

3g

White solid, 94.8 mg, 65% yield, ^{1}H NMR (400 MHz, Chloroform-*d*) δ 11.23 (s, 1H), 8.09 – 7.93 (m, 4H), 7.83 (d, J = 7.9 Hz, 1H), 7.72 (d, J = 7.7 Hz, 1H), 7.52 (d, J = 7.2 Hz, 2H), 7.38 – 7.24 (m, 3H), 7.01 (t, J = 7.3 Hz, 1H), 5.77 (s, 1H), 3.35 – 3.10 (m, 2H), 3.05 – 3.80 (m, 2H), 2.31 (s, 3H). ^{13}C NMR (101 MHz, Chloroform-*d*) δ 174.1 (d, J = 2.9 Hz), 169.1, 139.8 (d, J = 3.7 Hz), 139.2 (d, J = 3.5 Hz), 136.8 (d, J = 3.9 Hz), 134.4 (d, J = 9.9 Hz), 134.0 (d, J = 10.5 Hz), 129.2, 129.0 (d, J = 12.5 Hz), 128.8 (d, J = 2.4 Hz), 128.6 (d, J = 13.2 Hz), 127.2 (d, J = 3.8 Hz), 127.16 (d, J = 95.5 Hz), 127.15 (d, J = 1.2 Hz), 126.9 (d, J = 88.3 Hz), 124.8, 58.3 (d, J = 64.4 Hz), 39.7 (d, J = 5.3 Hz), 30.2, 24.4. ^{31}P NMR (162 MHz, Chloroform-*d*) δ 38.88. HRMS (ESI) *m/z* calcd for $C_{24}H_{21}Cl_2N_2O_3P$, $[M+Na]^+$: 509.0565, found: 509.0564.

N-(2-(3-(Di([1,1'-biphenyl]-4-yl)phosphoryl)-2-oxopyrrolidin-3-yl)phenyl)acetamide

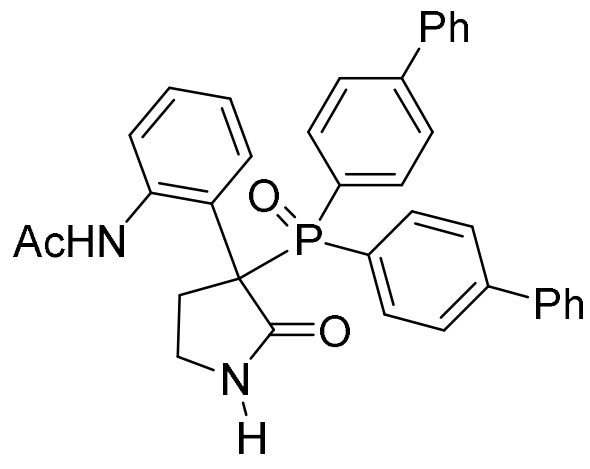

3h

White solid, 136.8 mg, 53% yield, ^{1}H NMR (400 MHz, Chloroform-*d*) δ 11.50 (s, 1H), 8.18 (dd, *J* = 10.7, 8.1 Hz, 2H), 8.09 (dd, *J* = 11.4, 8.1 Hz, 2H), 7.85 (d, *J* = 8.1 Hz, 2H), 7.77 (dd, *J* = 8.4, 2.8 Hz, 2H), 7.65 (d, *J* = 7.7 Hz, 2H), 7.60 – 7.53 (m, 4H), 7.50 (t, *J* = 7.5 Hz, 2H), 7.47 – 7.41 (m, 3H), 7.38 (d, *J* = 7.1 Hz, 1H), 7.24 (t, *J* = 7.8 Hz, 1H), 7.00 (t, *J* = 7.7 Hz, 1H), 5.83 (s, 1H), 3.52 – 3.32 (m, 1H), 3.25 – 3.08 (m, 1H), 3.07 – 2.86 (m, 2H), 2.36 (s, 3H). ^{13}C NMR (101 MHz, Chloroform-*d*) δ 174.4 (d, *J* = 2.8 Hz), 169.2, 145.4 (d, *J* = 2.9 Hz), 144.7 (d, *J* = 3.1 Hz), 139.8, 139.5, 136.9 (d, *J* = 3.6 Hz), 133.5 (d, *J* = 9.3 Hz), 133.1 (d, *J* = 9.9 Hz), 129.2 (d, *J* = 4.4 Hz), 129.1, 128.9, 128.6 (d, *J* = 1.9 Hz), 128.5, 128.1, 128.0, 127.28, 127.27 (d, *J* = 43.2 Hz), 127.22, 127.15 (d, *J* = 44.1 Hz), 127.1 (d, *J* = 12.2 Hz), 127.0 (d, *J* = 2.8 Hz), 126.7 (d, *J* = 12.8 Hz), 124.7, 58.1 (d, *J* = 62.1 Hz), 39.6 (d, *J* = 5.0 Hz), 30.5, 24.5. ^{31}P NMR (162 MHz, Chloroform-*d*) δ 39.79. HRMS (ESI) *m/z* calcd for $C_{36}H_{31}N_2O_3P$, $[M+Na]^+$: 593.1970, found: 593.1972.

N-(2-(3-(Di(naphthalen-2-yl)phosphoryl)-2-oxopyrrolidin-3-yl)phenyl)acetamide

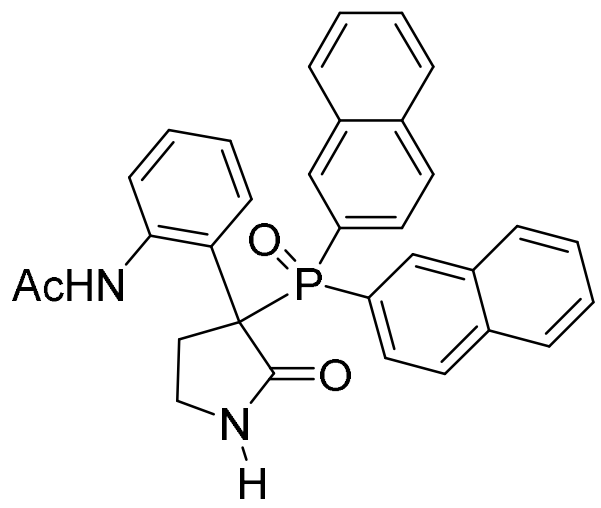

3i

White solid, 82.2 mg, 53% yield, ^{1}H NMR (400 MHz, Chloroform-*d*) δ 11.54 (s, 1H), 8.75 (d, *J* = 13.1 Hz, 1H), 8.55 (d, *J* = 13.8 Hz, 1H), 8.18 (t, *J* = 9.1 Hz, 1H), 8.10 – 7.77 (m, 9H), 7.68 – 7.46 (m, 4H), 7.20 (t, *J* = 7.7 Hz, 1H), 6.98 (t, *J* = 7.7 Hz, 1H), 5.89 (s, 1H), 3.57 – 3.40 (m, 1H), 3.18 – 3.08 (m, 1H), 3.00 – 2.82 (m, 2H), 2.39 (s, 3H). ^{13}C NMR (101 MHz, Chloroform-*d*) δ 174.4 (d, *J* = 2.9 Hz), 169.2, 136.9 (d, *J* = 3.3 Hz), 135.4 (d, *J* = 8.0 Hz), 135.1 (d, *J* = 8.9 Hz), 135.0 (d, *J* = 2.3 Hz), 134.7 (d, *J* = 2.6 Hz), 132.4 (d, *J* = 12.8 Hz), 132.2 (d, *J* = 13.7 Hz), 129.23, 129.20 (d, *J* = 3.6 Hz), 129.18, 128.8, 128.7 (d, *J* = 11.0 Hz), 128.54 (d, *J* = 2.0 Hz), 128.3, 128.2 (d, *J* = 11.7 Hz), 127.9 (d, *J* = 3.5 Hz), 127.6, 127.20, 127.16 (d, *J* = 65.2 Hz), 127.10, 127.07 (d = 2.1 Hz), 127.06 (d,

J = 64.5 Hz), 126.6, 124.7 (d, J = 1.4 Hz), 58.2 (d, J = 61.9 Hz), 39.6 (d, J = 4.6 Hz), 30.6, 24.5. ^{31}P NMR (162 MHz, Chloroform-*d*) δ 37.81. HRMS (ESI) *m/z* calcd for $C_{32}H_{27}N_2O_3P$, [M+Na]$^+$:541.1657, found: 541.1661.

***N*-(2-(3-(Di(thiophen-2-yl)phosphoryl)-2-oxopyrrolidin-3-yl)phenyl)acetamide**

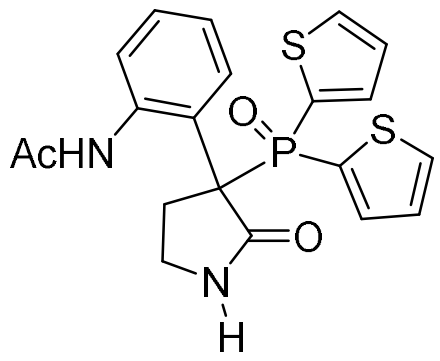

3j

Yellow solid, 38.7 mg, 30% yield, ^{1}H NMR (400 MHz, Chloroform-*d*) δ 11.23 (s, 1H), 8.01 – 7.79 (m, 3H), 7.79 – 7.58 (m, 3H), 7.10 – 6.95 (m, 2H), 5.78 (s, 1H), 3.37 – 3.11 (m, 2H), 3.11 – 2.88 (m, 2H), 2.31 (s, 3H). ^{13}C NMR (101 MHz, Chloroform-*d*) δ 174.2, 169.3, 138.5 (d, J = 9.7 Hz), 138.1 (d, J = 10.3 Hz), 137.0 (d, J = 2.2 Hz), 135.5 (d, J = 4.8 Hz), 135.0 (d, J = 5.7 Hz), 130.2 (d, J = 114.4 Hz), 129.6 (d, J = 4.7 Hz), 129.1 (d, J = 97.8 Hz), 128.8, 128.7 (d, J = 13.8 Hz), 127.8 (d, J = 15.6 Hz), 127.2, 126.4 (d, J = 4.2 Hz), 124.9, 58.3 (d, J = 75.1 Hz), 39.6 (d, J = 6.5 Hz), 29.7, 24.4. ^{31}P NMR (162 MHz, Chloroform-*d*) δ 33.48. HRMS (ESI) *m/z* calcd for $C_{20}H_{19}N_2O_3PS_2$, [M+Na]$^+$: 453.0472, found: 453.0471.

***N*-(2-(2-Oxo-3-(phenyl(*p*-tolyl)phosphoryl)pyrrolidin-3-yl)phenyl)acetamide**

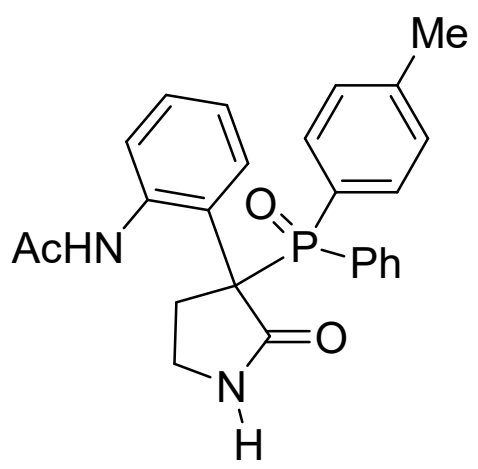

3k

White solid, 90.8 mg, 70% yield, >20:1 d.r.. ^{1}H NMR (400 MHz, Chloroform-*d*) δ 11.46 (s, 1H), 8.06 – 7.90 (m, 4H), 7.80 (t, J = 7.3 Hz, 2H), 7.44 – 7.37 (m, 1H), 7.37 – 7.25 (m, 4H), 7.22 (t, J = 7.6 Hz, 1H), 6.97 (t, J = 7.5 Hz, 1H), 5.70 (s, 1H), 3.45 – 3.30 (m, 1H), 3.20 – 3.10 (m, 1H), 2.96 – 2.78 (m, 2H), 2.44 (s, 3H), 2.32 (s, 3H). ^{13}C NMR (101 MHz, Chloroform-*d*) δ 174.4 (d, J = 2.9 Hz), 169.2, 143.4 (d, J = 3.0 Hz), 136.8, 133.0 (d, J = 9.4 Hz), 132.5 (d, J = 9.7 Hz), 132.1 (d, J = 3.0 Hz), 129.4 (d, J = 12.2 Hz), 129.1 (d, J = 4.4 Hz), 128.9 (d, J = 103.2 Hz), 128.4 (d, J = 2.5 Hz), 128.0 (d, J = 12.6 Hz), 127.8 (d, J = 4.2 Hz), 127.0 (d, J = 2.0 Hz), 125.5 (d, J = 96.1 Hz), 124.5 (d, J = 1.6 Hz), 57.9 (d, J = 61.6 Hz), 39.5 (d, J = 4.6 Hz), 30.4, 24.4, 21.7. ^{31}P NMR (162 MHz, Chloroform-*d*) δ 39.67. HRMS (ESI) *m/z* calcd for $C_{25}H_{25}N_2O_3P$, [M+Na]$^+$: 455.1500, found: 455.1503.

N-(2-(3-((4-Chlorophenyl)(phenyl)phosphoryl)-2-oxopyrrolidin-3-yl)phenyl)acetamide

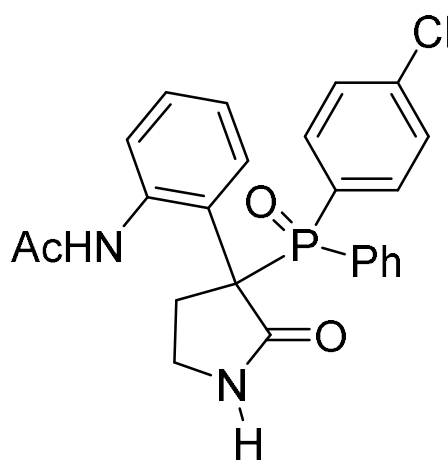

3l

White solid, 90.9 mg, 67% yield, >20:1 d.r.. ^{1}H NMR (400 MHz, Chloroform-*d*) δ 11.33 (s, 1H), 8.13 – 7.92 (m, 4H), 7.87 – 7.75 (m, 2H), 7.66 – 7.59 (m, 1H), 7.59 – 7.52 (m, 2H), 7.31 (dd, *J* = 8.6, 2.5 Hz, 2H), 7.25 (t, *J* = 7.7 Hz, 1H), 7.00 (t, *J* = 7.6 Hz, 1H), 5.79 (s, 1H), 3.45 – 3.25 (m, 1H), 3.23 – 3.05 (m, 1H), 3.00 – 2.82 (m, 2H), 2.32 (s, 3H). ^{13}C NMR (101 MHz, Chloroform-*d*) δ 174.2 (d, *J* = 3.1 Hz), 169.1, 138.9 (d, *J* = 3.7 Hz), 136.8 (d, *J* = 3.7 Hz), 134.1 (d, *J* = 10.5 Hz), 132.90, 132.85 (d, *J* = 9.0 Hz), 129.10 (d, *J* = 4.7 Hz), 129.07, 128.69 (d, *J* = 2.5 Hz), 128.67 (d, *J* = 11.8 Hz), 128.4 (d, *J* = 13.1 Hz), 127.7 (d, *J* = 95.0 Hz), 127.22 (d, *J* = 104.2 Hz), 127.18 (d, *J* = 2.0 Hz), 124.8 (d, *J* = 1.3 Hz), 58.0 (d, *J* = 62.5 Hz), 39.6 (d, *J* = 4.8 Hz), 30.3, 24.4. ^{31}P NMR (162 MHz, Chloroform-*d*) δ 39.24. HRMS (ESI) *m/z* calcd for $C_{24}H_{22}ClN_2O_3P$, $[M+Na]^+$: 475.0954, found: 475.0956.

N-(2-(3-(Benzyl(phenyl)phosphoryl)-2-oxopyrrolidin-3-yl)phenyl)acetamide

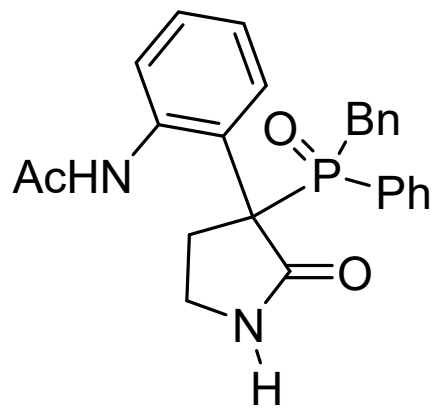

3m

White solid, 68.5 mg, 53% yield, >20:1 d.r.. ^{1}H NMR (400 MHz, Chloroform-*d*) δ 11.27 (s, 1H), 8.16 (d, *J* = 8.1 Hz, 1H), 7.96 (t, *J* = 9.1 Hz, 2H), 7.82 (d, *J* = 8.1 Hz, 1H), 7.62 – 7.46 (m, 3H), 7.40 (t, *J* = 7.9 Hz, 1H), 7.22 (t, *J* = 7.6 Hz, 1H), 7.14 – 6.94 (m, 5H), 5.65 (s, 1H), 4.10 (dd, *J* = 15.4, 8.9 Hz, 1H), 3.34 (dd, *J* = 19.2, 15.5 Hz, 1H), 3.17 – 2.98 (m, 2H), 2.78 – 2.62 (m, 1H), 2.55 – 2.42 (m, 1H), 2.28 (s, 3H). ^{13}C NMR (101 MHz, Chloroform-*d*) δ 174.2 (d, *J* = 3.4 Hz), 169.2, 137.1 (d, *J* = 3.5 Hz), 132.91 (d, *J* = 8.9 Hz), 132.87, 130.16 (d, *J* = 5.8 Hz), 130.15 (d, *J* = 9.1 Hz), 128.88, 128.86 (d, *J* = 4.1 Hz), 128.7, 128.6, 128.4 (d, *J* = 4.6 Hz), 128.3 (d, *J* = 2.2 Hz), 127.0 (d, *J* = 89.2 Hz), 126.7 (d, *J* = 3.0 Hz), 125.4, 55.8 (d, *J* = 56.4 Hz), 39.0 (d, *J* = 3.2 Hz), 32.8 (d, *J* = 64.3 Hz), 29.5, 24.4. ^{31}P NMR (162 MHz, Chloroform-*d*) δ 46.80. HRMS (ESI) *m/z* calcd for $C_{25}H_{25}N_2O_3P$, $[M+Na]^+$: 455.1500, found: 455.1503.

***N*-(2-(3-(Isopropyl(phenyl)phosphoryl)-2-oxopyrrolidin-3-yl)phenyl)acetamide**

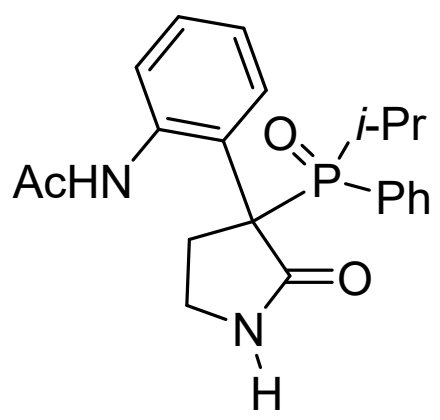

3n

White solid, 32.3 mg, 28% yield, >20:1 d.r.. ^{1}H NMR (400 MHz, Chloroform-*d*) δ 11.08 (s, 1H), 7.91 – 7.82 (m, 2H), 7.77 (d, *J* = 8.0 Hz, 1H), 7.31 (t, *J* = 7.4 Hz, 1H), 7.26 – 7.15 (m, 3H), 7.02 (t, *J* = 7.7 Hz, 1H), 6.74 (t, *J* = 7.6 Hz, 1H), 6.58 (s, 1H), 3.54 – 3.44 (m, 1H), 3.39 – 3.22 (m, 2H), 2.92 – 2.81 (m, 2H), 2.32 (s, 3H), 1.50 (dd, *J* = 15.8, 7.0 Hz, 3H), 1.00 (dd, *J* = 17.0, 7.2 Hz, 3H). ^{13}C NMR (101 MHz, Chloroform-*d*) δ 175.6 (d, *J* = 3.9 Hz), 169.2, 136.3 (d, *J* = 3.7 Hz), 131.7 (d, *J* = 2.9 Hz), 131.4 (d, *J* = 9.1 Hz), 128.5 (d, *J* = 95.0 Hz), 128.2 (d, *J* = 4.6 Hz), 128.0 (d, *J* = 2.3 Hz), 127.9 (d, *J* = 11.7 Hz), 127.2 (d, *J* = 4.3 Hz), 125.6 (d, *J* = 2.2 Hz), 124.1 (d, *J* = 1.8 Hz), 57.2 (d, *J* = 58.2 Hz), 40.0 (d, *J* = 7.0 Hz), 30.8, 28.5 (d, *J* = 62.2 Hz), 24.5, 16.5 (d, *J* = 3.3 Hz), 15.7 (d, *J* = 4.2 Hz). ^{31}P NMR (162 MHz, Chloroform-*d*) δ 56.06. HRMS (ESI) *m/z* calcd for $C_{21}H_{25}N_2O_3P$, $[M+Na]^+$: 407.1500, found: 407.1502.

***N*-(2-(3-(Cyclohexyl(phenyl)phosphoryl)-2-oxopyrrolidin-3-yl)phenyl)acetamide**

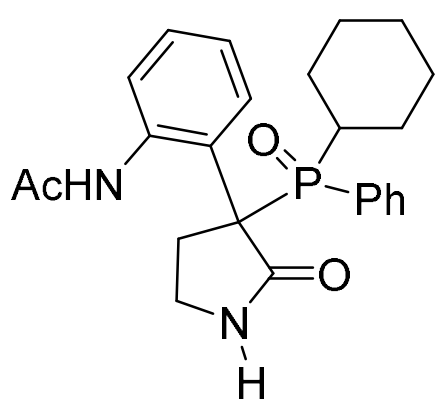

3o

White solid, 60.7 mg, 48% yield, ^{1}H NMR (400 MHz, Chloroform-*d*) δ 11.09 (s, 1H), 7.88 (dd, *J* = 11.0, 7.5 Hz, 2H), 7.78 (d, *J* = 8.1 Hz, 1H), 7.27 – 7.17 (m, 3H), 7.02 (t, *J* = 7.8 Hz, 1H), 6.75 (t, *J* = 7.7 Hz, 1H), 6.22 (s, 1H), 3.56 – 3.42 (m, 1H), 3.42 – 3.20 (m, 2H), 2.89 (dd, *J* = 13.3, 5.7 Hz, 1H), 2.67 (d, *J* = 14.2 Hz, 1H), 2.32 (s, 3H), 2.20 – 2.14 (m, 1H), 1.96 – 1.81 (m, 4H), 1.75 – 1.65 (m, 3H), 1.55 – 1.41 (m, 2H). ^{13}C NMR (101 MHz, Chloroform-*d*) ^{13}C NMR (101 MHz, Chloroform-*d*) δ 175.5 (d, *J* = 3.6 Hz), 169.2, 136.4 (d, *J* = 3.3 Hz), 131.6 (d, *J* = 2.7 Hz), 131.5 (d, *J* = 9.1 Hz), 128.4 (d, *J* = 94.2 Hz), 128.2 (d, *J* = 4.6 Hz), 128.0 (d, *J* = 2.1 Hz), 127.8 (d, *J* = 11.9 Hz), 127.0 (d, *J* = 4.2 Hz), 125.6 (d, *J* = 2.1 Hz), 124.1 (d, *J* = 1.3 Hz), 57.1 (d, *J* = 58.5 Hz), 40.0 (d, *J* = 7.1 Hz), 38.4 (d, *J* = 62.1 Hz), 31.0, 26.2 (d, *J* = 4.7 Hz), 26.0 (d, *J* = 5.6 Hz), 25.5, 25.3, 25.1, 24.5. ^{31}P NMR

(162 MHz, Chloroform-*d*) δ 53.52. HRMS (ESI) *m/z* calcd for $C_{24}H_{29}N_2O_3P$, $[M+Na]^+$: 447.1813, found: 447.1817.

***N*-(2-(3-(Di-*p*-tolylphosphoryl)-2-oxopyrrolidin-3-yl)-4-methylphenyl)acetamide**

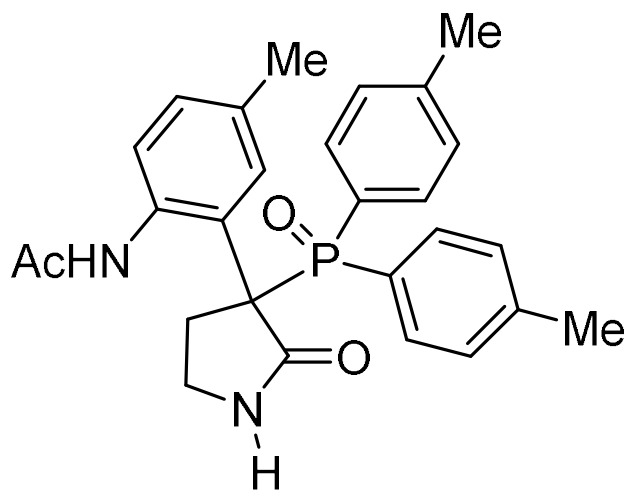

3p

White solid, 109.0 mg, 79% yield, ^{1}H NMR (400 MHz, Chloroform-*d*) δ 11.37 (s, 1H), 7.93 (dd, *J* = 10.8, 7.9 Hz, 2H), 7.83 (dd, *J* = 11.6, 8.0 Hz, 2H), 7.65 (d, *J* = 8.2 Hz, 1H), 7.58 (s, 1H), 7.31 (d, *J* = 5.8 Hz, 2H), 7.12 (d, *J* = 7.6 Hz, 2H), 7.03 (d, *J* = 8.0 Hz, 1H), 5.73 (s, 1H), 3.40 – 3.25 (m, 1H), 3.15 – 3.05 (m, 1H), 2.92 – 2.73 (m, 2H), 2.42 (s, 3H), 2.37 – 2.24 (m, 6H), 2.21 (s, 3H). ^{13}C NMR (101 MHz, Chloroform-*d*) δ 174.4 (d, *J* = 2.7 Hz), 169.2, 143.2 (d, *J* = 2.8 Hz), 142.5 (d, *J* = 2.8 Hz), 134.2, 134.1 (d, *J* = 1.4 Hz), 133.0 (d, *J* = 9.3 Hz), 132.5 (d, *J* = 9.9 Hz), 129.5 (d, *J* = 4.4 Hz), 129.3 (d, *J* = 12.2 Hz), 129.0 (d, *J* = 2.2 Hz), 128.7 (d, *J* = 13.0 Hz), 127.8 (d, *J* = 4.0 Hz), 126.9 (d, *J* = 1.9 Hz), 125.9 (d, *J* = 96.4 Hz), 125.7 (d, *J* = 105.9 Hz), 57.8 (d, *J* = 61.9 Hz), 39.5 (d, *J* = 4.7 Hz), 30.5, 24.3, 21.7, 21.6, 21.1. ^{31}P NMR (162 MHz, Chloroform-*d*) δ 39.97. HRMS (ESI) *m/z* calcd for $C_{27}H_{29}N_2O_3P$, $[M+Na]^+$: 483.1813, found: 483.1813.

***N*-(2-(3-(Diphenylphosphoryl)-2-oxopyrrolidin-3-yl)-5-methylphenyl)acetamide**

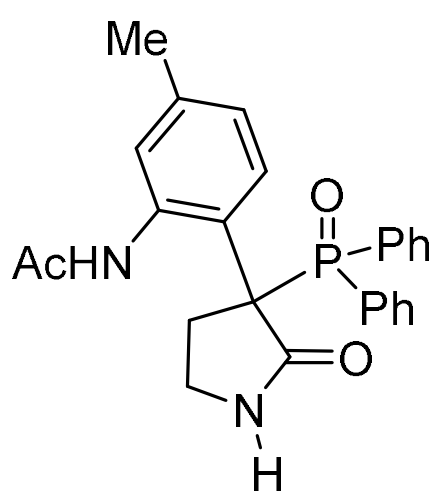

3q

White solid, 111.3 mg, 86% yield, ^{1}H NMR (400 MHz, Chloroform-*d*) δ 11.36 (s, 1H), 8.18 – 7.96 (m, 4H), 7.69 – 7.31 (m, 8H), 6.78 (d, *J* = 8.4 Hz, 1H), 5.77 (s, 1H), 3.40 – 3.25 (m, 1H), 3.17 – 3.05 (m, 1H), 2.90 – 2.75 (m, 2H), 2.40 – 2.15 (m, 6H). ^{13}C NMR (101 MHz, Chloroform-*d*) δ 174.4 (d, *J* = 3.0 Hz), 169.1, 138.4 (d, *J* = 2.2 Hz), 136.6 (d, *J* = 3.4 Hz), 132.9 (d, *J* = 9.0 Hz), 132.62 (d, *J* = 3.2 Hz), 132.60 (d, *J* = 9.5 Hz), 132.1 (d, *J* = 3.0

Hz), 129.1 (d, J = 93.3 Hz), 129.0 (d, J = 4.6 Hz), 128.9 (d, J = 103.1 Hz), 128.5 (d, J = 11.8 Hz), 128.0 (d, J = 12.5 Hz), 127.5, 125.6, 124.5 (d, J = 4.5 Hz), 57.6 (d, J = 62.1 Hz), 39.5 (d, J = 4.8 Hz), 30.6, 24.4, 21.0. ^{31}P NMR (162 MHz, Chloroform-*d*) δ 39.40. HRMS (ESI) *m/z* calcd for $C_{25}H_{25}N_2O_3P$, $[M+Na]^+$: 455.1500, found: 455.1501.

***N*-(2-(3-(Diphenylphosphoryl)-2-oxopyrrolidin-3-yl)-6-methylphenyl)acetamide**

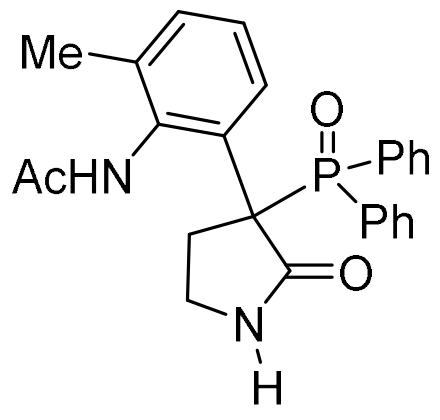

3r

White solid, 112.5 mg, 87% yield, ^{1}H NMR (400 MHz, Chloroform-*d*) δ 11.06 (s, 1H), 8.08 (t, J = 9.2 Hz, 2H), 7.96 – 7.74 (m, 3H), 7.66 – 7.47 (m, 3H), 7.41 (t, J = 7.4 Hz, 1H), 7.24 – 7.26 (m, 2H), 7.14 (d, J = 7.5 Hz, 1H), 6.99 (t, J = 7.8 Hz, 1H), 6.01 (s, 1H), 3.50 – 3.30 (m, 1H), 3.15 – 3.05 (m, 1H), 2.88 – 2.58 (m, 2H), 2.35 – 2.17 (m, 6H). ^{13}C NMR (101 MHz, Chloroform-*d*) δ 174.3, 169.2, 138.1 (d, J = 2.2 Hz), 135.4 (d, J = 4.1 Hz), 132.8 (d, J = 8.8 Hz), 132.7 (d, J = 2.8 Hz), 132.6 (d, J = 9.5 Hz), 132.1 (d, J = 2.6 Hz), 131.8, 130.9 (d, J = 1.8 Hz), 129.1 (d, J = 93.3 Hz), 129.0 (d, J = 88.8 Hz), 128.6 (d, J = 11.7 Hz), 127.9 (d, J = 12.5 Hz), 126.8 (d, J = 3.9 Hz), 126.0, 57.3 (d, J = 59.4 Hz), 39.2 (d, J = 2.5 Hz), 30.5, 23.8, 19.2. ^{31}P NMR (162 MHz, Chloroform-*d*) δ 39.36. HRMS (ESI) *m/z* calcd for $C_{25}H_{25}N_2O_3P$, $[M+Na]^+$: 455.1500, found: 455.1504.

***N*-(2-(3-(Diphenylphosphoryl)-2-oxopyrrolidin-3-yl)-4-methoxyphenyl)acetamide**

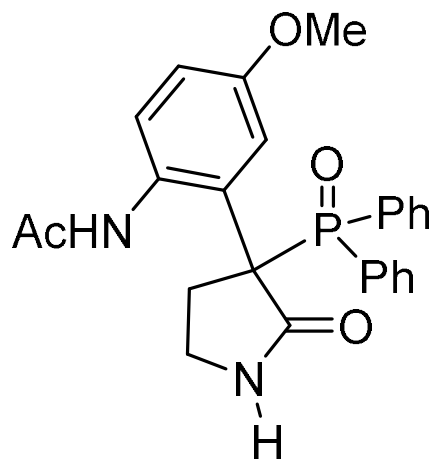

3s

White solid, 91.3 mg, 68% yield, ^{1}H NMR (400 MHz, Chloroform-*d*) δ 11.07 (s, 1H), 8.15 – 8.00 (m, 4H), 7.65 – 7.41 (m, 8H), 7.40-7.30 (m, 2H), 6.77 (d, J = 8.7 Hz, 1H), 5.73 (s, 1H), 3.68 (s, 3H), 3.50 – 3.35 (m, 1H), 3.20 – 3.10 (m, 1H), 2.90 – 2.78 (m, 2H), 2.30 (s, 3H). ^{13}C NMR (101 MHz, Chloroform-*d*) δ 174.2 (d, J = 2.9 Hz), 169.2, 156.3 (d, J = 1.9 Hz), 132.8 (d, J = 9.1 Hz), 132.7 (d, J = 2.7 Hz), 132.6 (d, J = 9.7 Hz), 132.2 (d, J = 3.1

Hz), 129.7 (d, J = 4.0 Hz), 129.07 (d, J = 90.4 Hz), 129.05 (d, J = 93.3 Hz), 128.6 (d, J = 11.6 Hz), 128.1, 128.0 (d, J = 12.6 Hz), 114.80, 114.75, 113.8 (d, J = 2.3 Hz), 57.5 (d, J = 60.2 Hz), 55.5, 39.3 (d, J = 3.9 Hz), 30.3, 24.2. ^{31}P NMR (162 MHz, Chloroform-*d*) δ 38.48. HRMS (ESI) *m/z* calcd for $C_{25}H_{25}N_2O_4P$, $[M+Na]^+$: 471.1450, found: 471.1453.

N-(4-Bromo-2-(3-(diphenylphosphoryl)-2-oxopyrrolidin-3-yl)phenyl)acetamide

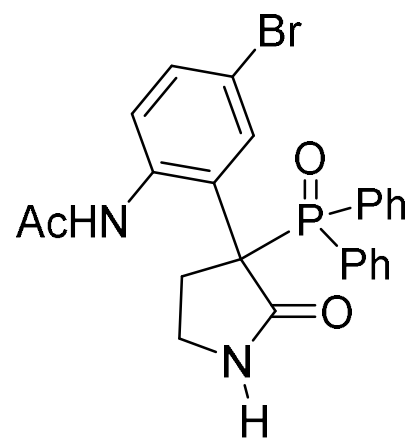

3t

Yellow solid, 105.4 mg, 86% yield, ^{1}H NMR (400 MHz, Chloroform-*d*) δ 11.37 (s, 1H), 8.12 – 8.02 (m, 4H), 7.99 (s, 1H), 7.71 (d, J = 8.7 Hz, 1H), 7.61 (t, J = 7.2 Hz, 1H), 7.55 – 7.42 (m, 3H), 7.42 – 7.29 (m, 3H), 5.90 (s, 1H), 3.54 – 3.36 (m, 1H), 3.20 – 3.10 (m, 1H), 2.93 – 2.74 (m, 2H), 2.31 (s, 3H). ^{13}C NMR (101 MHz, Chloroform-*d*) δ 173.7 (d, J = 2.4 Hz), 169.1, 135.9 (d, J = 3.7 Hz), 132.9 (d, J = 2.9 Hz), 132.8 (d, J = 9.3 Hz), 132.53 (d, J = 9.5 Hz), 132.52 (d, J = 3.0 Hz), 131.8(d, J = 4.8 Hz), 131.4 (d, J = 2.5 Hz), 130.0 (d, J = 4.1 Hz), 128.6 (d, J = 11.9 Hz),128.7 (d, J = 101.4 Hz) 128.3, 128.2 (d, J = 12.5 Hz), 128.1 (d, J = 103.5 Hz), 117.7 (d, J = 1.7 Hz), 57.8 (d, J = 59.7 Hz), 39.4 (d, J = 4.1 Hz), 30.2, 24.4. ^{31}P NMR (162 MHz, Chloroform-*d*) δ 39.68. HRMS (ESI) *m/z* calcd for $C_{24}H_{22}BrN_2O_3P$, $[M+Na]^+$: 519.0449, found: 519.0449.

N-(4-Chloro-2-(3-(diphenylphosphoryl)-2-oxopyrrolidin-3-yl)phenyl)acetamide

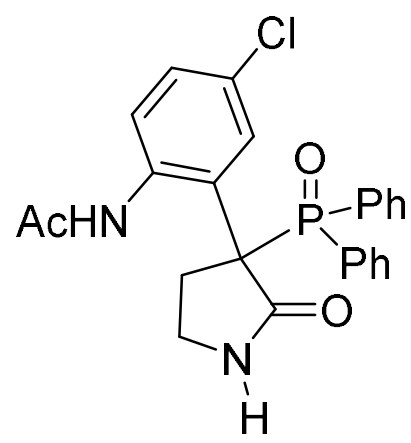

3u

Yellow solid, 88.1 mg, 65% yield, ^{1}H NMR (400 MHz, Chloroform-*d*) δ 11.38 (s, 1H), 8.15 – 8.00 (m, 4H), 7.86 (s, 1H), 7.76 (d, J = 8.7 Hz, 1H),7.64 – 7.50 (m, 3H), 7.49 – 7.34 (m, 3H), 7.17 (d, J = 8.5 Hz, 1H), 5.83 (s, 1H), 3.50 – 3.38 (m, 1H), 3.20 – 3.10 (m, 1H), 2.94 – 2.72 (m, 2H), 2.31 (s, 3H). ^{13}C NMR (101 MHz, Chloroform-*d*) δ 173.7 (d, J = 2.7 Hz), 169.2, 135.4 (d, J = 3.3 Hz), 132.9 (d, J = 4.2 Hz), 132.8 (d, J = 9.3 Hz), 132.52 (d, J = 9.5 Hz), 132.49 (d, J = 3.5 Hz), 129.8 (d, J = 1.7 Hz), 129.7 (d, J = 4.0 Hz), 128.9 (d, J = 4.7 Hz), 128.62 (d, J = 11.8

Hz), 128.55 (d, J = 131.4 Hz), 128.4 (d, J = 2.4 Hz), 128.3 (d, J = 127.5 Hz), 128.2 (d, J = 12.6 Hz), 127.9 (d, J = 1.9 Hz), 57.7 (d, J = 59.9 Hz), 39.4 (d, J = 4.0 Hz), 30.2, 24.4. ^{31}P NMR (162 MHz, Chloroform-*d*) δ 39.00. HRMS (ESI) *m/z* calcd for $C_{24}H_{22}ClN_2O_3P$, $[M+Na]^+$: 475.0954, found: 475.0955.

_N_-(2-(3-(Diphenylphosphoryl)-2-oxopyrrolidin-3-yl)-4-fluorophenyl)acetamide

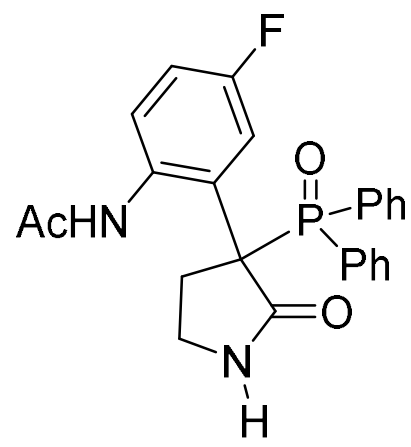

3v

Yellow solid, 81.2 mg, 80% yield, ^{1}H NMR (400 MHz, Chloroform-*d*) δ 11.25 (s, 1H), 8.18 – 7.96 (m, 4H), 7.77 – 7.58 (m, 3H), 7.58 – 7.50 (m, 2H), 7.45 (d, J = 7.4 Hz, 1H), 7.40 – 7.32 (m, 2H), 6.91 (t, J = 7.1 Hz, 1H), 5.86 (s, 1H), 3.55 – 3.35 (m, 1H), 3.20 – 3.10 (m, 1H), 2.90 – 2.75 (m, 2H), 2.31 (s, 3H). ^{13}C NMR (101 MHz, Chloroform-*d*) δ 173.8 (d, J_{C-P} = 2.5 Hz), 169.2, 159.1 (d, J_{C-F} = 243.9 Hz), 132.9 (d, J_{C-P} = 2.9 Hz), 132.76 (d, J_{C-P} = 4.4 Hz), 132.75 (d, J_{C-P} = 9.0 Hz), 132.5 (d, J_{C-P} = 9.4 Hz), 132.4 (d, J_{C-P} = 2.0 Hz), 130.4 (dd, J_{C-P} = 4.1 Hz, J_{C-F} = 7.7 Hz), 128.74 (J_{C-P} = 94.2 Hz), 128.70 (dd, J_{C-P} = 4.0 Hz, J_{C-F} = 8.0 Hz), 128.6 (d, J = 11.9 Hz), 128.24 (J_{C-P} = 101.8 Hz), 128.18 (d, J = 12.6 Hz), 116.0 (dd, J_{C-P} = 4.3 Hz, J_{C-F} = 25.8 Hz), 115.2 (dd, J_{C-P} = 2.2 Hz, J_{C-F} = 22.2 Hz), 57.5 (d, J = 58.8 Hz), 39.3 (d, J = 3.6 Hz), 30.2, 24.3.^{19}F NMR (376 MHz, Chloroform-*d*) δ -116.43. ^{31}P NMR (162 MHz, Chloroform-*d*) δ 38.56. HRMS (ESI) *m/z* calcd for $C_{24}H_{22}FN_2O_3P$, $[M+Na]^+$: 459.1250, found: 459.1252.

_N_-(3-(3-(Diphenylphosphoryl)-2-oxopyrrolidin-3-yl)-[1,1'-biphenyl]-4-yl)acetamide

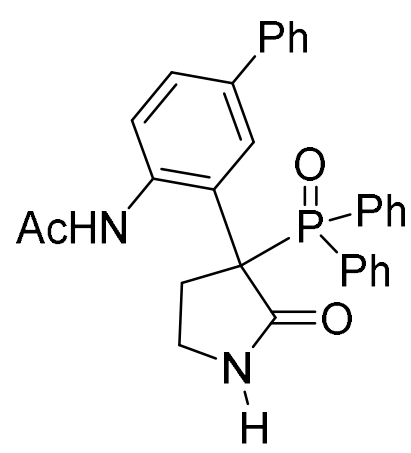

3w

Yellow solid, 121.7 mg, 82% yield, ^{1}H NMR (400 MHz, Chloroform-*d*) δ 11.40 (s, 1H), 8.15 – 8.04 (m, 5H), 7.87 (d, J = 8.3 Hz, 1H), 7.63 – 7.48 (m, 4H), 7.46 – 7.30 (m, 9H), 5.96 (s, 1H), 3.50 – 3.30 (m, 1H), 3.23 – 3.08 (m, 1H), 3.00 – 2.85 (m, 2H), 2.34 (s, 3H). ^{13}C NMR (101 MHz, Chloroform-*d*) δ 174.2 (d, J = 2.6 Hz), 169.3, 140.5, 137.2 (d, J = 1.4 Hz), 135.9 (d, J = 3.6 Hz), 132.9 (d, J = 9.0 Hz), 132.8 (d, J = 2.8 Hz), 132.6 (d, J = 9.5 Hz),

132.3 (d, *J* = 2.9 Hz), 128.9 (d, *J* = 93.4 Hz), 128.7, 128.56 (d, *J* = 12.0 Hz), 128.55 (d, *J* = 96.9 Hz), 128.03 (d, *J* = 2.6 Hz), 128.02 (d, *J* = 12.6 Hz), 128.00, 127.3 (d, *J* = 2.3 Hz), 127.2, 127.03 (d, *J* = 2.4 Hz), 126.97, 58.0 (d, *J* = 61.0 Hz), 39.5 (d, *J* = 4.3 Hz), 30.3, 24.5. ^{31}P NMR (162 MHz, Chloroform-*d*) δ 39.32. HRMS (ESI) *m/z* calcd for $C_{30}H_{27}N_2O_3P$, $[M+Na]^+$: 517.1657, found: 517.1658.

Methyl 4-acetamido-3-(3-(diphenylphosphoryl)-2-oxopyrrolidin-3-yl)benzoate

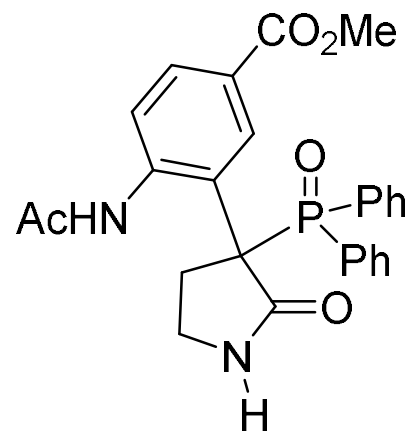

3x

White solid, 104.2 mg, 82% yield, ^{1}H NMR (400 MHz, Chloroform-*d*) δ 11.70 (s, 1H), 8.49 (s, 1H), 8.12 – 8.00 (m, 4H), 7.85 (d, *J* = 8.6 Hz, 1H), 7.65 – 7.29 (m, 7H), 6.20 (s, 1H), 3.86 (s, 3H), 3.50 – 3.37 (m, 1H), 3.20 – 3.08 (m, 1H), 3.05 – 2.92 (m, 1H), 2.85 – 2.72(m, 1H), 2.34 (s, 3H). ^{13}C NMR (101 MHz, Chloroform-*d*) δ 173.9 (d, *J* = 2.2 Hz), 169.4, 166.5, 141.3 (d, *J* = 3.2 Hz), 132.9 (d, *J* = 9.5 Hz),132.8 (d, *J* = 3.5 Hz), 132.6 (d, *J* = 9.6 Hz), 132.4 (d, *J* = 2.7 Hz), 130.7 (d, *J* = 4.6 Hz), 129.8 (d, *J* = 1.9 Hz), 128.8 (d, *J* = 95.3 Hz), 128.5 (d, *J* = 12.1 Hz), 128.2 (d, *J* = 12.6 Hz), 128.1 (d, *J* = 103.0 Hz), 127.2 (d, *J* = 3.2 Hz), 125.5, 125.4, 58.5 (d, *J* = 60.8 Hz), 52.1, 39.6 (d, *J* = 4.6 Hz), 30.3, 24.6. ^{31}P NMR (162 MHz, Chloroform-*d*) δ 39.77. HRMS (ESI) *m/z* calcd for $C_{26}H_{25}N_2O_5P$, $[M+Na]^+$: 499.1399, found: 499.1402.

***N*-(2-(3-(Diphenylphosphoryl)-2-oxopyrrolidin-3-yl)phenyl)benzamide**

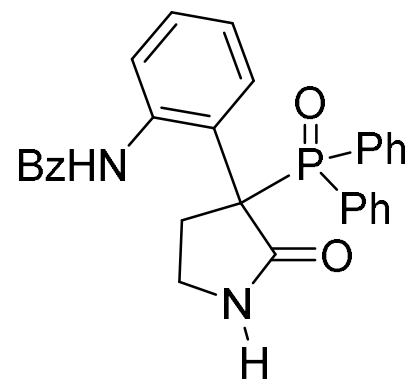

3y

White solid, 100.2 mg, 70% yield, ^{1}H NMR (400 MHz, Chloroform-*d*) δ 12.0 (s, 1H), 8.50 – 8.35 (m, 2H), 8.19 – 8.07 (m, 2H), 7.98 – 7.86 (m, 3H), 7.82 (d, *J* = 8.0 Hz, 1H), 7.63 – 7.49 (m, 6H), 7.43 (t, *J* = 7.3 Hz, 1H), 7.36 – 7.27 (m, 3H), 7.01 (t, *J* = 7.6 Hz, 1H), 6.04 (s, 1H), 3.23 – 3.16 (m, 1H), 3.10 – 3.00 (m, 1H), 2.96 – 2.82 (m, 1H), 2.68 – 2.56 (m, 1H). ^{13}C NMR (101 MHz, Chloroform-*d*) δ 174.2 (d, *J* = 2.7 Hz), 165.8, 137.0 (d, *J* = 3.8 Hz), 134.4, 132.9 (d, *J* = 8.7 Hz), 132.8 (d, *J* = 2.7 Hz), 132.6 (d, *J* = 9.5 Hz), 132.2 (d, *J* = 2.7 Hz), 131.8, 129.3 (d, *J*

= 3.7 Hz), 128.8 (d, J = 2.4 Hz), 128.71 (d, J = 3.5 Hz), 128.67 (d, J = 12.0 Hz), 128.64, 128.60 (d, J = 69.1 Hz), 128.4 (d, J = 2.2 Hz), 128.1, 128.0 (d, J = 86.9 Hz), 127.9 (d, J = 12.6 Hz), 125.1 (d, J = 1.8 Hz), 57.2 (d, J = 61.2 Hz), 39.3 (d, J = 3.9 Hz), 30.4. ^{31}P NMR (162 MHz, Chloroform-*d*) δ 39.16. HRMS (ESI) *m/z* calcd for $C_{29}H_{25}N_2O_3P$, $[M+Na]^+$: 503.1500, found: 503.1501.

Ethyl 2-((4'-acetamido-3'-(3-(diphenylphosphoryl)-2-oxopyrrolidin-3-yl)-[1,1'-biphenyl]-4-yl)oxy)-2-methylpropanoate

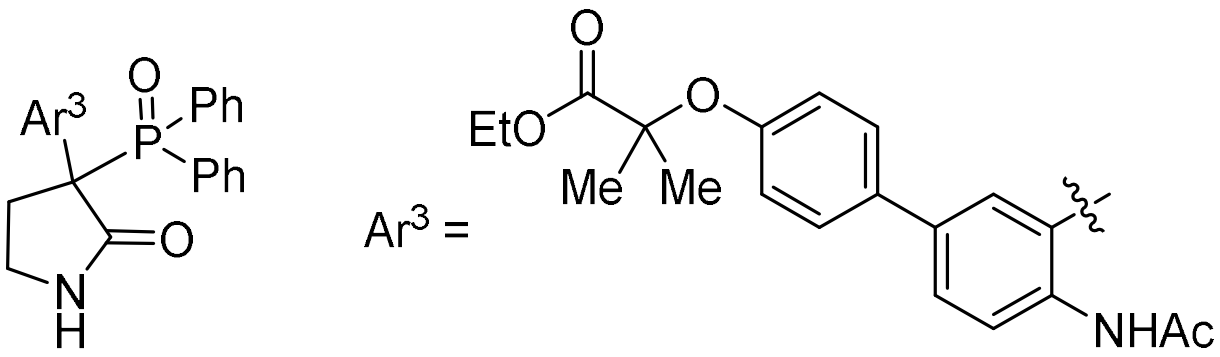

from Clofibrate
3z

White solid, 153.5 mg, 70% yield, ^{1}H NMR (400 MHz, Chloroform-*d*) δ 11.37 (s, 1H), 8.15 – 8.04 (m, 4H), 8.01 (s, 1H), 7.83 (d, J = 8.4 Hz, 1H), 7.65 – 7.48 (m, 3H), 7.45 – 7.29 (m, 6H), 6.87 (d, J = 8.3 Hz, 2H), 5.98 (s, 1H), 4.27 (q, J = 7.0 Hz, 2H), 3.16 – 3.02 (m, 1H), 3.13 – 3.07 (m, 1H), 2.95 – 2.80 (m, 2H), 2.33 (s, 3H), 1.63 (s, 6H), 1.29 (t, J = 7.2 Hz, 3H). ^{13}C NMR (101 MHz, Chloroform-*d*) δ 174.3, 174.2 (d, J = 2.6 Hz), 169.2, 154.9, 136.7, 135.5 (d, J = 3.0 Hz), 134.4, 132.9 (d, J = 9.1 Hz), 132.7 (d, J = 2.9 Hz), 132.6 (d, J = 9.6 Hz), 132.3 (d, J = 2.7 Hz), 128.9 (d, J = 102.3 Hz), 128.6 (d, J = 11.9 Hz), 128.0 (d, J = 12.6 Hz), 127.7 (d, J = 78.1 Hz), 127.64, 127.61 (d, J = 4.8 Hz), 127.2 (d, J = 1.6 Hz), 126.7 (d, J = 1.7 Hz), 119.27, 119.25 (d, J = 4.7 Hz), 79.2, 61.5, 58.0 (d, J = 60.9 Hz), 39.5 (d, J = 4.8 Hz), 30.3, 25.4, 24.4, 14.1. ^{31}P NMR (162 MHz, Chloroform-*d*) δ 33.55. HRMS (ESI) *m/z* calcd for $C_{36}H_{37}N_2O_6P$, $[M+Na]^+$: 647.2287, found: 647.2289.

Isopropyl 2-(3-(4'-acetamido-3'-(3-(diphenylphosphoryl)-2-oxopyrrolidin-3-yl)-[1,1'-biphenyl]-4-carbonyl)phenoxy)-2-methylpropanoate

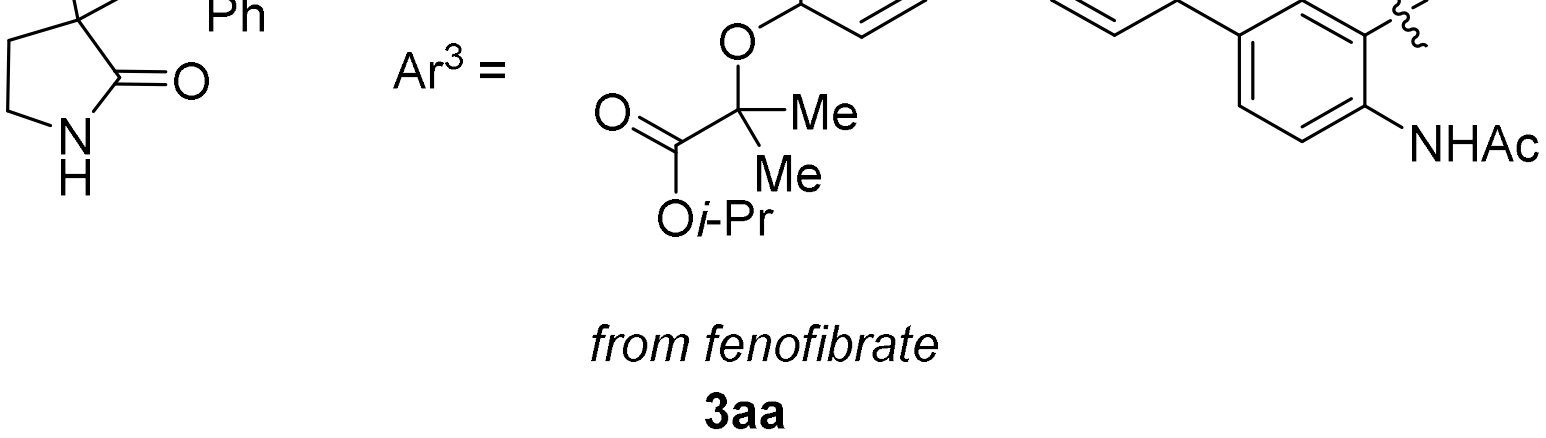

from fenofibrate
3aa

White solid, 164.8 mg, 74% yield, ^{1}H NMR (400 MHz, Chloroform-*d*) δ 11.42 (s, 1H), 8.17 – 8.05 (m, 4H), 7.91 (d, *J* = 8.4 Hz, 1H), 7.85 – 7.70 (m, 4H), 7.65 – 7.30 (m, 9H), 6.89 (d, *J* = 8.5 Hz, 2H), 5.96 (s, 1H), 5.17 – 5.05 (m, 1H), 3.53 – 3.38 (m, 1H), 3.22 – 3.10 (m, 1H), 3.00 – 2.82 (m, 2H), 2.35 (s, 3H), 1.68 (s, 6H), 1.23 (d, *J* = 6.3 Hz, 6H). ^{13}C NMR (101 MHz, Chloroform-*d*) δ 195.1, 174.1 (d, *J* = 2.4 Hz), 173.2, 169.3, 159.5, 144.1, 136.8 (d, *J* = 3.5 Hz), 136.6, 136.0, 132.9 (d, *J* = 9.0 Hz), 132.8 (d, *J* = 2.9 Hz), 132.6 (d, *J* = 9.5 Hz), 132.4 (d, *J* = 2.9 Hz), 132.0, 130.7, 130.4, 128.9 (d, *J* = 61.9 Hz), 128.7 (d, *J* = 56.1 Hz), 128.6 (d, *J* = 11.8 Hz), 128.3 (d, *J* = 3.8 Hz), 128.2 (d, *J* = 4.3 Hz), 128.1 (d, *J* = 12.5 Hz), 128.0 (d, *J* = 4.3 Hz), 127.4 (d, *J* = 1.5 Hz), 127.1 (d, *J* = 2.1 Hz), 126.7, 117.2 (2C), 79.4, 69.4, 57.8 (d, *J* = 60.2 Hz), 39.4 (d, *J* = 3.6 Hz), 30.3, 25.4, 24.5, 21.6. ^{31}P NMR (162 MHz, Chloroform-*d*) δ 39.04. HRMS (ESI) *m/z* calcd for $C_{44}H_{43}N_2O_7P$, $[M+Na]^+$: 765.2706, found:765.2708.

Heptan-2-yl 2-((5-(4-acetamido-3-(3-(diphenylphosphoryl)-2-oxopyrrolidin-3-yl)phenyl)quinolin-8-yl)oxy)acetate

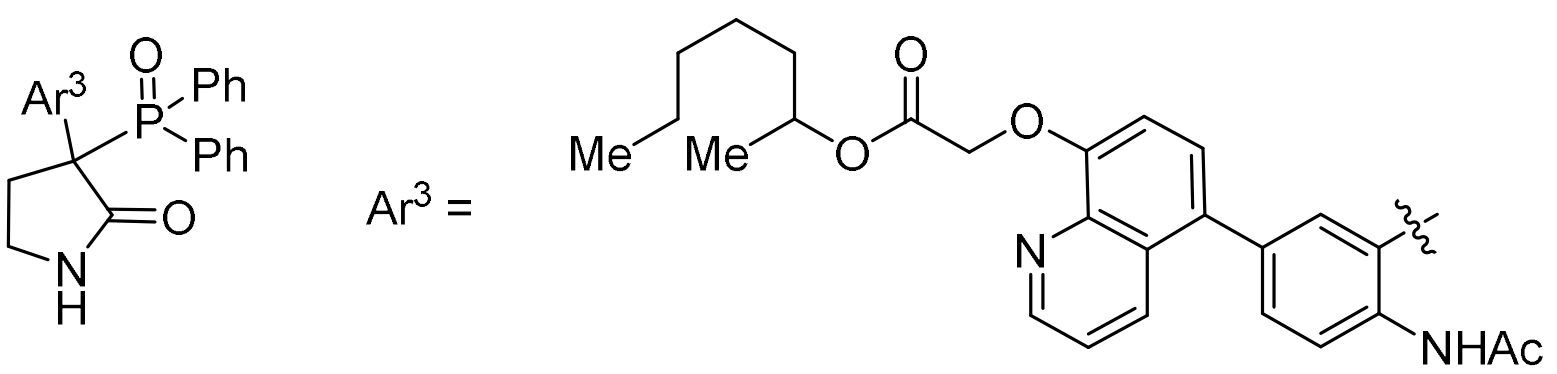

from Cloquintocet-mexyl
3ab

White solid, 135.2 mg, 61% yield, ^{1}H NMR (400 MHz, Chloroform-*d*) δ 11.57 (s, 1H), 8.94 (d, *J* = 4.1 Hz, 1H), 8.18 – 8.08 (m, 4H), 7.92 (d, *J* = 8.4 Hz, 1H), 7.85 (s, 1H), 7.79 – 7.69 (m, 1H), 7.63 – 7.49 (m, 4H), 7.45 – 7.25 (m, 5H), 7.18 (d, *J* = 7.7 Hz, 1H), 6.96 (d, *J* = 7.9 Hz, 1H), 5.79 (m, 1H), 5.14 – 5.00 (m, 1H), 4.97 (s, 2H), 3.50 – 3.30 (m, 1H), 3.20 – 3.10 (m, 1H), 3.00 – 2.82 (m, 2H), 2.37 (s, 3H), 1.32 – 1.21 (m, 11H), 0.85 (t, *J* = 6.2 Hz, 3H). ^{13}C NMR (101 MHz, Chloroform-*d*) δ 173.9 (d, *J* = 2.6 Hz), 169.3, 168.5, 153.1, 149.2, 140.1, 136.1 (d, *J* = 3.2 Hz), 135.4, 134.2, 132.9 (d, *J* = 9.3 Hz), 132.80 (d, *J* = 2.3 Hz), 132.79 (d, *J* = 9.7 Hz), 132.2 (d, *J* = 2.4 Hz), 130.8 (d, *J* = 4.7 Hz), 130.0, 128.9 (d, *J* = 103.0 Hz), 128.7 (d, *J* = 11.8 Hz), 128.6 (d, *J* = 94.1 Hz), 128.1 (d, *J* = 12.5 Hz), 127.9 (d, *J* = 2.8 Hz), 127.8, 127.3 (2C), 126.9, 121.7, 108.8, 72.7, 66.3, 57.7 (d, *J* = 61.1 Hz), 39.4 (d, *J* = 4.3 Hz), 35.7, 31.5, 30.2, 25.0, 24.5, 22.5, 20.0, 14.0. ^{31}P NMR (162 MHz, Chloroform-*d*) δ 39.01. HRMS (ESI) *m/z* calcd for $C_{42}H_{44}N_3O_6P$, $[M+Na]^+$: 740.2865, found: 740.2864.

6-(*tert*-Butyl)-*N*-(2-(3-(diphenylphosphoryl)-2-oxopyrrolidin-3-yl)phenyl)-1,1-dimethyl-2,3-dihydro-1H-indene-4-carboxamide

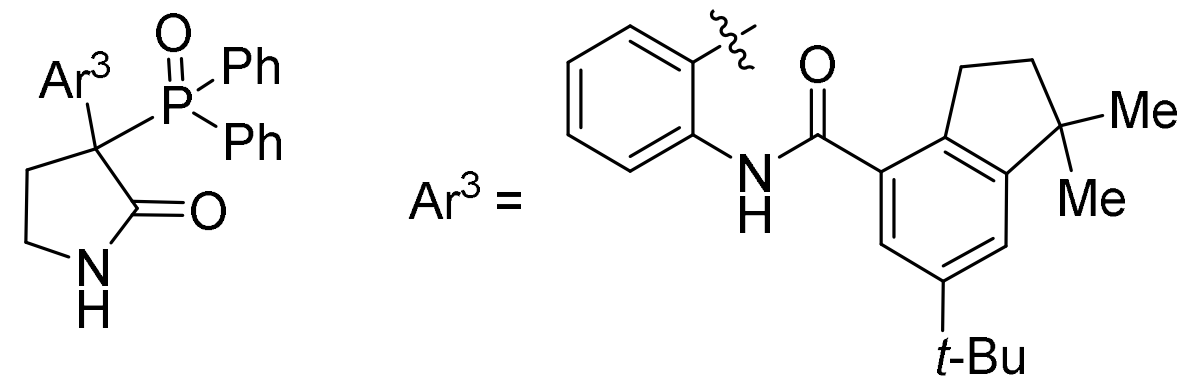

from celestolide

3ac

White solid, 112.1 mg, 62% yield, ^{1}H NMR (400 MHz, Chloroform-*d*) δ 11.79 (s, 1H), 8.24 – 8.12 (m, 3H), 7.95 – 7.81 (m, 4H), 7.60 (t, *J* = 7.4 Hz, 1H), 7.52 (td, *J* = 7.8, 2.9 Hz, 2H), 7.41 (t, *J* = 7.6 Hz, 1H),7.39 (s, 1H), 7.35-7.25 (m, 3H), 6.98 (t, *J* = 7.8 Hz, 1H), 5.84 (s, 1H), 3.45 – 3.35 (m, 1H), 3.34 – 3.18 (m, 2H), 3.16 – 2.93 (m, 2H), 2.75 – 2.62 (m, 1H), 2.05 – 1.90 (m, 2H), 1.53 (s, 9H), 1.33 (d, *J* = 10.7 Hz, 6H). ^{13}C NMR (101 MHz, Chloroform-*d*) δ 174.4 (d, *J* = 2.7 Hz), 167.4, 154.1, 150.0, 141.7, 137.4 (d, *J* = 3.6 Hz), 133.1 (d, *J* = 8.7 Hz), 132.6 (d, *J* = 7.8 Hz), 132.6, 132.0 (d, *J* = 2.7 Hz), 130.3, 129.4 (d, *J* = 4.1 Hz), 129.2 (d, *J* = 103.1 Hz), 128.9 (d, *J* = 91.9 Hz), 128.7 (d, *J* = 2.1 Hz), 128.5 (d, *J* = 11.6 Hz), 128.3 (d, *J* = 2.4 Hz), 128.2 (d, *J* = 5.0 Hz), 127.8 (d, *J* = 12.5 Hz), 124.8, 123.1, 121.8, 57.1 (d, *J* = 61.2 Hz), 43.6, 41.6, 39.3 (d, *J* = 4.2 Hz), 35.1, 31.8, 30.4, 30.2, 29.0, 28.7. ^{31}P NMR (162 MHz, Chloroform-*d*) δ 38.02. HRMS (ESI) *m/z* calcd for $C_{38}H_{41}N_2O_3P$, $[M+Na]^+$: 627.2752, found: 627.2755.

4-Acetamido-3-(3-(di-*p*-tolylphosphoryl)-2-oxopyrrolidin-3-yl)phenyl palmitate

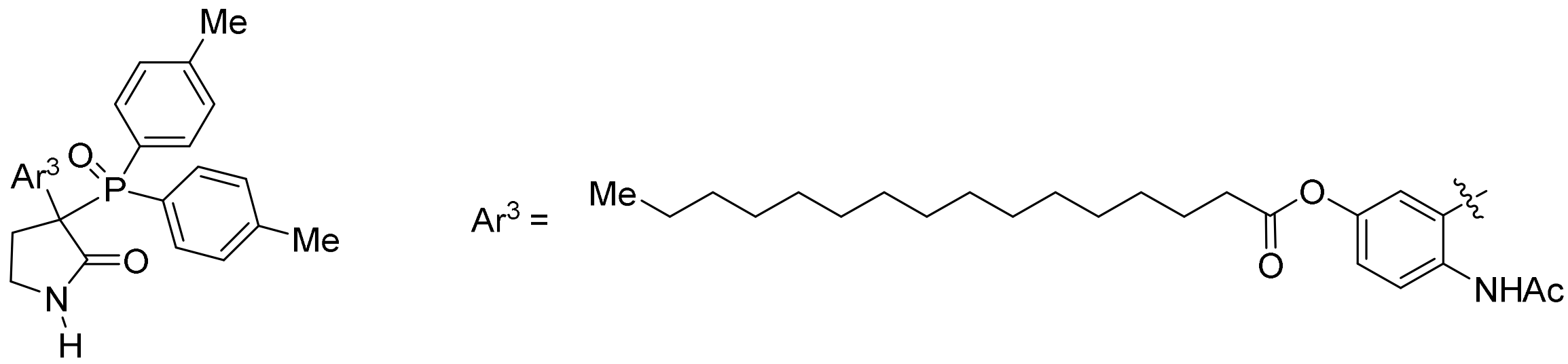

from Palmitic acid

3ad

White solid, 153.0 mg, 73% yield, ^{1}H NMR (400 MHz, Chloroform-*d*) δ 11.45 (s, 1H), 7.95 – 7.79 (m, 4H), 7.59 (s, 1H), 7.35 – 7.25 (m, 3H), 7.18 – 7.10 (m, 2H), 6.96 (d, *J* = 8.9 Hz, 1H), 5.80 (s, 1H), 3.50 – 3.32 (m, 1H), 3.16 – 3.06 (m, 1H), 2.86 – 2.70 (m, 2H), 2.50 (t, *J* = 7.6 Hz, 2H), 2.42 (s, 3H), 2.30 (m, 6H), 1.28 (s, 26H), 0.89 (t, *J* = 6.7 Hz, 3H). ^{13}C NMR (101 MHz, Chloroform-*d*) δ 174.0 (d, *J* = 2.3 Hz), 172.1, 169.2, 147.0 (d, *J* = 1.7 Hz), 143.4 (d, *J* = 3.0 Hz), 142.8 (d, *J* = 2.9 Hz), 134.4 (d, *J* = 3.4 Hz), 132.9 (d, *J* = 9.4 Hz), 132.5 (d, *J* = 10.0 Hz),

129.5 (d, J = 4.4 Hz), 129.3 (d, J = 12.2 Hz), 129.0 (d, J = 13.0 Hz), 127.6, 125.6 (d, J = 96.5 Hz), 125.3 (d, J = 105.7 Hz), 122.4 (d, J = 4.3 Hz), 121.6, 57.6 (d, J = 60.3 Hz), 39.4 (d, J = 3.4 Hz), 34.3, 31.9, 30.3, 29.7 – 29.6 (m, 6C), 29.5, 29.4, 29.3, 29.1, 24.8, 24.3, 22.7, 21.7, 21.6, 14.1. ^{31}P NMR (162 MHz, Chloroform-*d*) δ 39.32. HRMS (ESI) *m/z* calcd for $C_{42}H_{57}N_2O_5P$, $[M+Na]^+$: 723.3903, found: 723.3904.

4-Acetamido-3-(3-(di-*p*-tolylphosphoryl)-2-oxopyrrolidin-3-yl)phenyl 2-(4-(2-(4-chlorobenzamido)ethyl)-phenoxy)-2-methylpropanoate

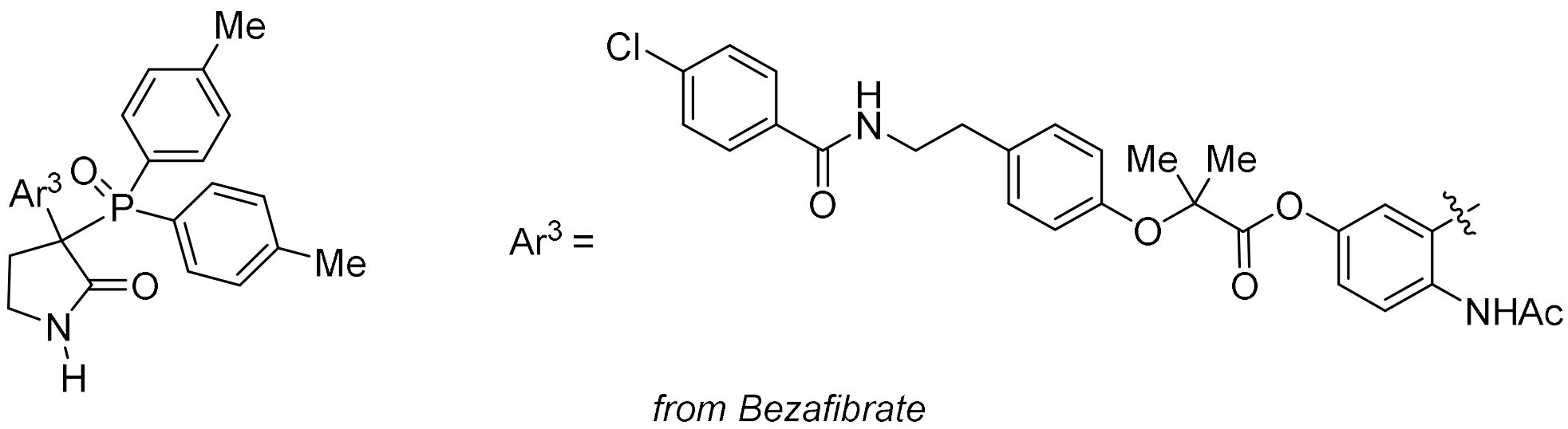

3ae

White solid, 153.0 mg, 73% yield, ^{1}H NMR (400 MHz, Chloroform-*d*) δ 11.40 (s, 1H), 7.90 – 7.73 (m, 5H), 7.58 (d, J = 8.5 Hz, 2H), 7.53 (s, 1H), 7.36 – 7.26 (m, 5H), 7.20 – 7.10 (m, 3H), 6.96 – 6.82 (m, 3H), 6.68 (t, J = 5.5 Hz, 1H), 5.62 (s, 1H), 3.85 – 3.75 (m, 1H), 3.66 – 3.56 (m, 1H), 3.50 – 3.34 (m, 1H), 3.08 – 2.98 (m, 1H), 2.91 (t, J = 6.8 Hz, 2H), 2.80 – 2.64 (m, 2H), 2.41 (s, 3H), 2.32 (s, 3H), 2.29 (s, 3H), 1.74 (s, 6H). ^{13}C NMR (101 MHz, Chloroform-*d*) δ 173.8 (d, J = 2.7 Hz), 172.8, 169.2, 166.7, 154.0, 146.7 (d, J = 1.9 Hz), 143.5 (d, J = 2.9 Hz), 142.9 (d, J = 2.9 Hz), 137.6, 134.8 (d, J = 3.5 Hz), 133.3, 132.8 (d, J = 9.4 Hz), 132.5, 132.4 (d, J = 10.0 Hz), 129.9 (d, J = 4.3 Hz), 129.8, 129.3 (d, J = 12.1 Hz), 129.0 (d, J = 13.1 Hz), 128.8, 128.4, 127.7 (d, J = 1.7 Hz), 125.4 (d, J = 96.7 Hz), 125.3 (d, J = 105.8 Hz), 122.1 (d, J = 4.8 Hz), 121.3, 118.8, 79.1, 57.5 (d, J = 59.3 Hz), 41.2, 39.3 (d, J = 3.4 Hz), 34.6, 30.2, 25.9, 24.8, 24.3, 21.7, 21.6. ^{31}P NMR (162 MHz, Chloroform-*d*) δ 39.76. HRMS (ESI) *m/z* calcd for $C_{45}H_{45}ClN_3O_7P$, $[M+Na]^+$: 828.2581, found: 828.2584.

4-Acetamido-3-(3-(di-*p*-tolylphosphoryl)-2-oxopyrrolidin-3-yl)phenyl 2-(1-(4-chlorobenzoyl)-5-methoxy-2-methyl-1H-indol-3-yl)acetate

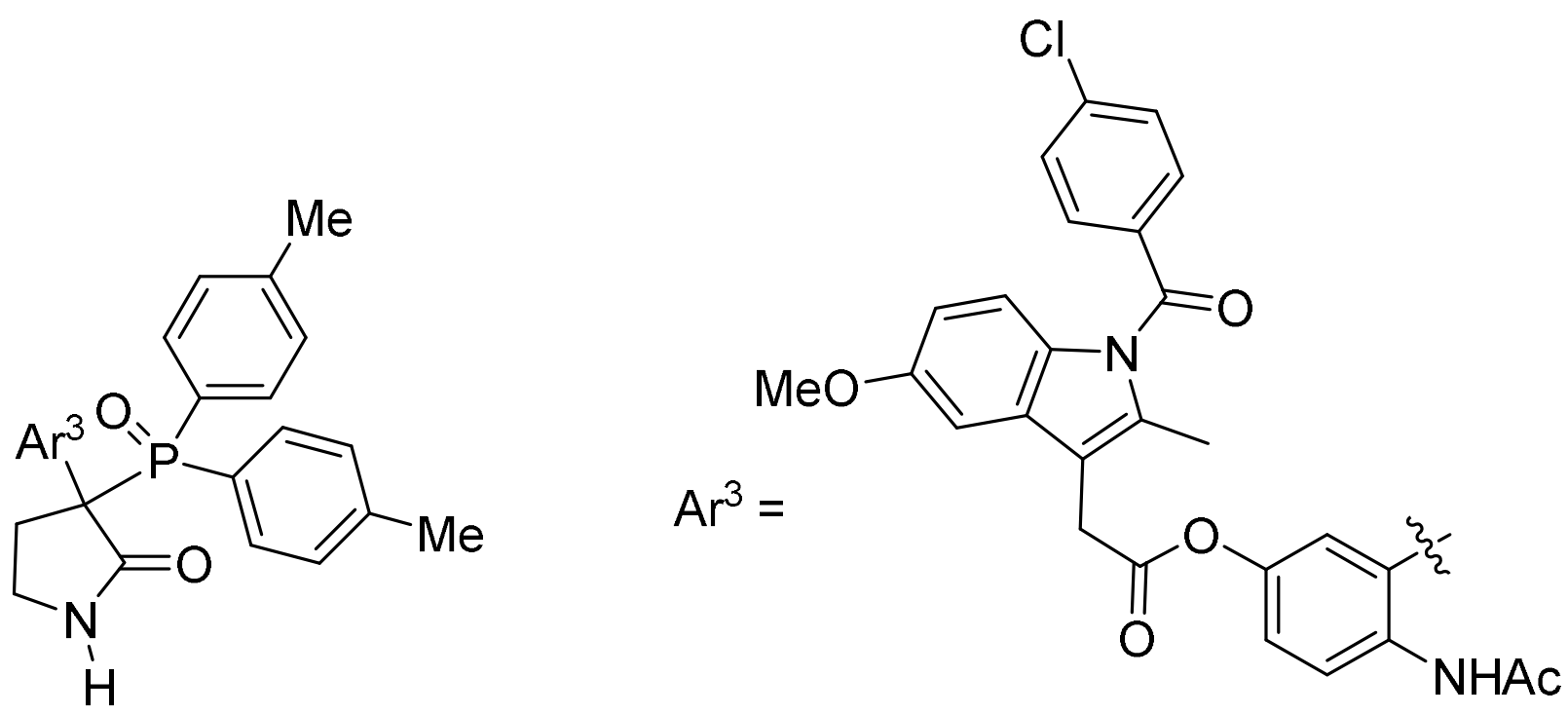

from Indomethacin
3af

White solid, 144.3 mg, 60% yield, ^{1}H NMR (400 MHz, Chloroform-*d*) δ 11.44 (s, 1H), 7.96 – 7.86 (m, 2H), 7.85 – 7.77 (m, 3H), 7.70 (d, *J* = 8.2 Hz, 2H), 7.63 (s, 1H), 7.49 (d, *J* = 8.2 Hz, 2H), 7.32 (d, *J* = 6.0 Hz, 2H), 7.11 (d, *J* = 7.5 Hz, 2H), 7.03 (s, 1H), 6.94 (d, *J* = 5.6 Hz, 2H), 6.71 (d, *J* = 9.0 Hz, 1H), 5.70 (s, 1H), 3.88 – 3.83 (m, 5H), 3.46-3.30 (m, 1H), 3.14-3.04 (m, 1H), 2.90-2.70 (m, 2H), 2.46 – 2,40 (m, 6H), 2.32 – 2.24 (m, 6H). ^{13}C NMR (101 MHz, Chloroform-*d*) δ 173.9 (d, *J* = 2.2 Hz), 169.2, 169.1, 168.4, 156.1, 146.9, 143.5 (d, *J* = 2.5 Hz), 142.9 (d, *J* = 2.9 Hz), 139.3, 136.1, 134.6 (d, *J* = 3.5 Hz), 133.9, 132.9 (d, *J* = 9.3 Hz), 132.4 (d, *J* = 10.1 Hz), 131.3, 130.9, 130.6, 129.6, 129.3 (d, *J* = 12.4 Hz), 129.2, 129.0 (d, *J* = 13.0 Hz), 127.7, 125.5 (d, *J* = 96.6 Hz), 125.2 (d, *J* = 105.4 Hz), 122.2 (d, *J* = 4.0 Hz), 121.3 (d, *J* = 2.1 Hz), 115.1, 112.1, 111.9, 101.2, 57.6 (d, *J* = 60.0 Hz), 55.8, 39.3 (d, *J* = 3.5 Hz), 30.4, 24.3, 21.7, 21.5, 13.5. ^{31}P NMR (162 MHz, Chloroform-*d*) δ 39.85. HRMS (ESI) *m/z* calcd for $C_{45}H_{41}ClN_3O_7P$, $[M+Na]^+$: 824.2268, found: 824.2269.

***N*-(2-(3-(Diphenylphosphoryl)-2-oxo-1,2,3,4-tetrahydroquinolin-3-yl)phenyl)acetamide**

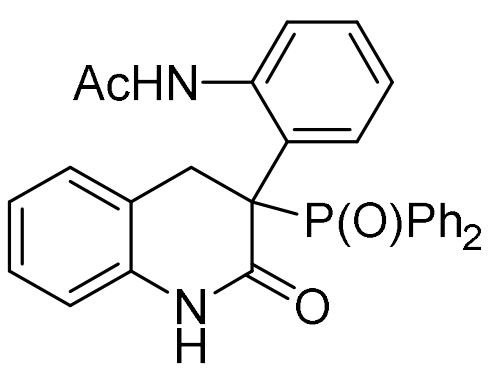

5a

White solid, 97.7 mg, 72% yield, ^{1}H NMR (400 MHz, Chloroform-*d*) δ 11.62 (s, 1H), 9.22 (s, 1H), 8.40 – 8.10 (s, 2H), 7.90 – 7.65 (m, 4H), 7.65 – 7.20 (m, 7H), 7.10 – 6.98 (m, 2H), 6.92 – 6.80 (m, 3H), 6.68 – 6.52 (s, 1H), 6.41 (d, *J* = 7.7 Hz, 1H), 4.38 (s, 1H), 3.40 (d, *J* = 14.8 Hz, 1H), 2.42 (s, 3H). ^{13}C NMR (101 MHz, Chloroform-*d*) δ 170.5, 168.5, 137.8 (d, *J* = 3.3 Hz), 135.2, 133.5 (d, *J* = 9.1 Hz), 132.8 (d, *J* = 9.5 Hz), 132.6 (d, *J* = 2.9 Hz), 131.9 (d, *J* = 2.9 Hz), 130.3 (d, *J* = 100.8 Hz), 130.0 (d, *J* = 4.3 Hz), 129.3 (d, *J* = 91.5 Hz), 128.9 (d, *J* = 2.5 Hz), 128.6

(d, *J* = 12.0 Hz), 128.2, 127.9 (d, *J* = 2.7 Hz), 127.84, 127.81 (d, *J* = 12.4 Hz), 127.2, 124.7 (d, *J* = 2.1 Hz), 124.2, 122.8, 121.9 (d, *J* = 11.7 Hz), 114.7, 58.5 (d, *J* = 76.5 Hz), 30.6, 24.6. ^{31}P NMR (162 MHz, Chloroform-*d*) δ 44.30. HRMS (ESI) *m/z* calcd for $C_{29}H_{25}N_2O_3P$, $[M+Na]^+$: 503.1500, found: 503.1501.

N-(4-Chloro-2-(3-(diphenylphosphoryl)-2-oxo-1,2,3,4-tetrahydroquinolin-3-yl)phenyl)acetamide

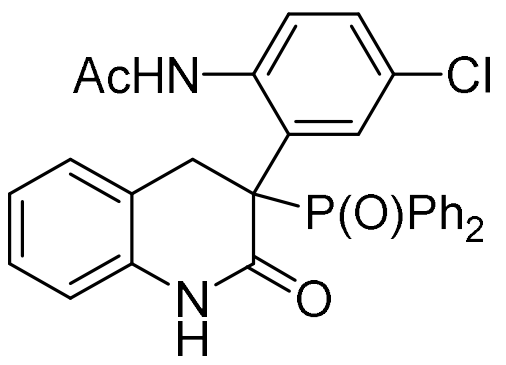

5b

Yellow solid, 94.0 mg, 61% yield, ^{1}H NMR (400 MHz, Chloroform-*d*) δ 11.58 (s, 1H), 9.07 (s, 1H), 8.30 – 8.10 (m, 2H), 7.90 (t, *J* = 10.0 Hz, 2H), 7.68 (d, *J* = 8.8 Hz, 1H), 7.62 – 7.54 (m, 1H), 7.54 – 7.41 (m, 3H), 7.40 – 7.30 (s, 2H), 7.03 (d, *J* = 7.8 Hz, 2H), 6.98 – 6.80 (m, 3H), 6.47 (s, 1H), 4.36 (s, 1H), 3.43 (d, *J* = 12.2 Hz, 1H), 2.41 (s, 3H). ^{13}C NMR (101 MHz, Chloroform-*d*) δ 169.7, 168.5, 136.5, 135.1, 133.4 (d, *J* = 9.3 Hz), 132.8 (d, *J* = 9.5 Hz), 132.7 (d, *J* = 2.9 Hz), 132.3 (d, *J* = 2.9 Hz), 129.9 (d, *J* = 2.2 Hz), 129.8, 129.4 (d, *J* = 104.5 Hz), 129.1 (d, *J* = 96.2 Hz), 128.8 (d, *J* = 1.4 Hz), 128.6 (d, *J* = 12.0 Hz), 128.2, 128.1 (2C), 128.0 (d, *J* = 11.6 Hz), 124.9, 124.3, 121.5 (d, *J* = 11.5 Hz), 114.9, 58.6 (d, *J* = 72.0 Hz), 30.4, 24.6. ^{31}P NMR (162 MHz, Chloroform-*d*) δ 44.30. HRMS (ESI) *m/z* calcd for $C_{29}H_{24}ClN_2O_3P$, $[M+Na]^+$: 537.1111, found: 537.1114.

N-(4-Bromo-2-(3-(diphenylphosphoryl)-2-oxo-1,2,3,4-tetrahydroquinolin-3-yl)phenyl)acetamide

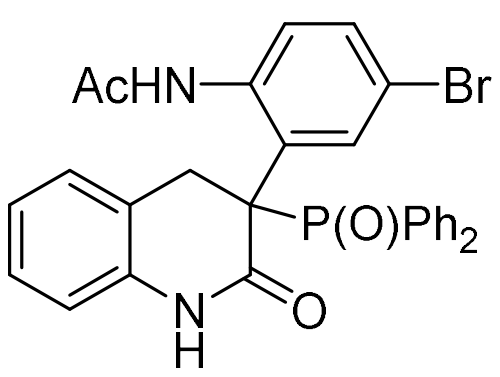

5c

Yellow solid, 103.0 mg, 62% yield, ^{1}H NMR (400 MHz, Chloroform-*d*) δ 11.57 (s, 1H), 8.90 (s, 1H), 8.30 – 8.02 (m, 2H), 7.95 – 7.80 (m, 2H), 7.65 – 7.42 (m, 5H), 7.40 – 7.30 (m, 2H), 7.17 (d, *J* = 6.7 Hz, 1H), 7.08 – 6.82 (m, 4H), 6.55 – 6.40 (m, 1H), 4.37 (s, 1H), 3.43 (d, *J* = 16.3 Hz, 1H), 2.41 (s, 3H). ^{13}C NMR (101 MHz, Chloroform-*d*) δ 169.6, 168.5, 137.0, 135.1, 133.4 (d, *J* = 9.2 Hz), 132.8 (d, *J* = 9.4 Hz), 132.7 (d, *J* = 2.8 Hz), 132.6, 132.3 (d, *J* = 2.5 Hz), 131.8 (d, *J* = 2.1 Hz), 129.3 (d, *J* = 104.4 Hz), 129.0 (d, *J* = 107.5 Hz), 128.6 (d, *J* = 12.0 Hz), 128.1 (3C), 128.0 (d, *J* = 14.6 Hz), 125.2 (d, *J* = 1.7 Hz), 124.3, 121.5 (d, *J* = 11.2 Hz), 117.6, 114.9, 58.6 (d, *J* = 83.2

Hz), 30.4, 24.6. ^{31}P NMR (162 MHz, Chloroform-*d*) δ 44.32. HRMS (ESI) *m/z* calcd for $C_{29}H_{24}BrN_2O_3P$, $[M+Na]^+$: 581.0606, found: 581.0607.

***N*-(2-(3-(Diphenylphosphoryl)-6-methoxy-2-oxo-1,2,3,4-tetrahydroquinolin-3-yl)phenyl)acetamide**

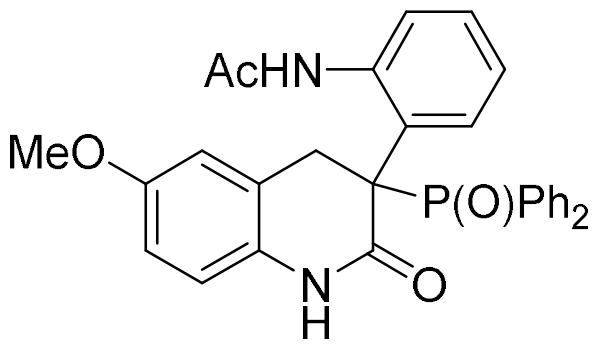

5d

Yellow solid, 55.0 mg, 36% yield, ^{1}H NMR (600 MHz, Chloroform-*d*) δ 11.53 (s, 1H), 8.32 – 8.08 (m, 3H), 7.82 – 7.73 (m, 2H), 7.67 (d, *J* = 8.1 Hz, 1H), 7.57 (d, *J* = 6.8 Hz, 1H), 7.54 – 7.45 (m, 2H), 7.41 – 7.35 (m, 1H), 7.30 – 7.24 (m, 2H), 7.08 – 7.01 (m, 1H), 6.91 – 6.83 (m, 1H), 6.69 – 6.62 (m, 1H), 6.54 (s, 1H), 6.45 (d, *J* = 7.9 Hz, 1H), 6.35 (d, *J* = 8.3 Hz, 1H), 4.32 (s, 1H), 3.64 (s, 3H), 3.34 (d, *J* = 12.1 Hz, 1H), 2.38 (s, 3H). ^{13}C NMR (151 MHz, Chloroform-*d*) δ 169.8, 168.2, 156.2, 137.9, 133.5 (d, *J* = 9.1 Hz), 132.8 (d, *J* = 9.5 Hz), 132.6 (d, *J* = 2.9 Hz), 131.9, 130.12 (d, *J* = 104.7 Hz), 130.10 (d, *J* = 4.3 Hz), 129.4 (d, *J* = 95.2 Hz), 128.9 (d, *J* = 2.5 Hz), 128.5 (d, *J* = 12.0 Hz), 127.8 (d, *J* = 12.5 Hz), 127.2, 124.6, 123.4 (d, *J* = 11.8 Hz), 122.8, 115.5, 113.6, 113.5, 113.4, 58.6 (d, *J* = 63.4 Hz), 55.4, 30.8, 24.7. ^{31}P NMR (162 MHz, Chloroform-*d*) δ 44.72. HRMS (ESI) *m/z* calcd for $C_{30}H_{27}N_2O_4P$, $[M+Na]^+$: 533.1606, found: 533.1607.

***N*-(2-(3-(Diphenylphosphoryl)-6-methyl-2-oxo-1,2,3,4-tetrahydroquinolin-3-yl)phenyl)acetamide**

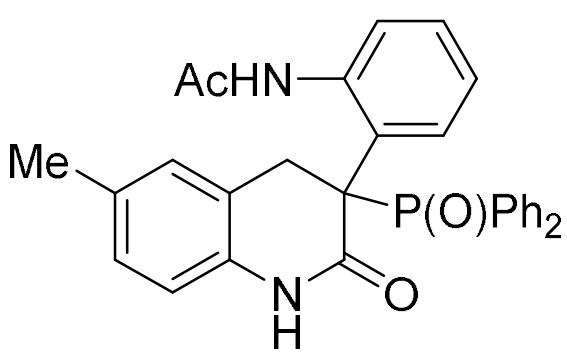

5e

Yellow solid, 79.8 mg, 54% yield, ^{1}H NMR (400 MHz, Chloroform-*d*) δ 11.63 (s, 1H), 8.56 (s, 1H), 8.30 – 8.10 (m, 2H), 7.83 (dd, *J* = 11.8, 7.7 Hz, 2H), 7.74 (d, *J* = 8.1 Hz, 1H), 7.64 – 7.48 (m, 3H), 7.42 (t, *J* = 7.5 Hz, 1H), 7.35 – 7.22 (m, 2H), 7.09 (t, *J* = 7.7 Hz, 1H), 6.92 (d, *J* = 8.2 Hz, 1H), 6.84 (s, 1H), 6.75 (d, *J* = 8.0 Hz, 1H), 6.66 (t, *J* = 7.7 Hz, 1H), 6.35 (d, *J* = 7.9 Hz, 1H), 4.32 (s, 1H), 3.40 (dd, *J* = 14.8, 6.6 Hz, 1H), 2.42 (s, 3H), 2.17 (s, 3H). ^{13}C NMR (101 MHz, Chloroform-*d*) δ 170.0, 168.4, 137.9, 134.0, 133.5 (d, *J* = 9.1 Hz), 132.8 (d, *J* = 9.6 Hz), 132.6 (d, *J* = 5.3 Hz), 132.5 (d, *J* = 2.9 Hz), 131.9 (d, *J* = 3.2 Hz), 130.2 (d, *J* = 104.6 Hz), 130.0 (d, *J* = 4.5 Hz), 129.5 (d, *J* = 104.1 Hz), 128.93 (d, *J* = 9.7 Hz), 128.90 (d, *J* = 11.8 Hz), 128.5 (d, *J* = 11.9 Hz), 128.3, 127.8 (d, *J*

= 12.6 Hz), 127.0, 124.6, 122.8, 121.7 (d, *J* = 11.8 Hz), 114.3, 58.7 (d, *J* = 67.5 Hz), 30.5, 24.6, 20.9. ^{31}P NMR (162 MHz, Chloroform-*d*) δ 44.38. HRMS (ESI) *m/z* calcd for $C_{30}H_{27}N_2O_3P$, $[M+Na]^+$: 517.1657, found: 517.1657.

***N*-(2-(6-Bromo-3-(diphenylphosphoryl)-2-oxo-1,2,3,4-tetrahydroquinolin-3-yl)phenyl)acetamide**

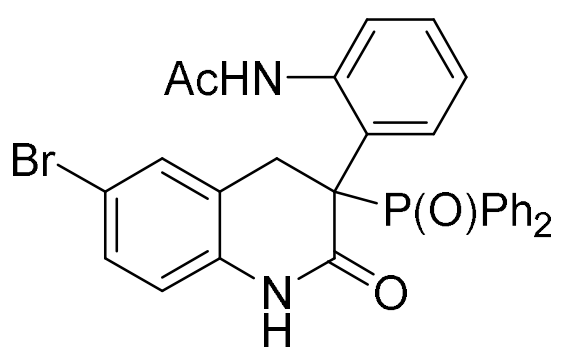

5f

White solid, 94.8 mg, 65% yield, ^{1}H NMR (700 MHz, DMSO-*d*$_6$) δ 11.48 (s, 1H), 11.00 (s, 1H), 8.16 – 7.95 (m, 2H) 7.95 – 7.85 (m, 2H), 7.66 (t, *J* = 7.2 Hz, 1H), 7.61 – 7.49 (m, 4H), 7.46 – 7.39 (m, 2H), 7.25 (d, *J* = 8.6 Hz, 1H), 7.09 – 7.02 (m, 2H), 6.97 (s, 1H), 6.84 (t, *J* = 7.5 Hz, 1H), 6.78 (d, *J* = 6.7 Hz, 1H), 4.22 (s, 1H), 3.37 (1H, overlap with water peak), 2.26 (s, 3H). ^{13}C NMR (176 MHz, DMSO-*d*$_6$) δ 168.9, 167.7, 137.6, 136.6, 133.5 (d, *J* = 9.1 Hz), 133.2, 133.0 (d, *J* = 9.1 Hz), 132.7, 132.56, 131.4, 130.0 (d, *J* = 101.9 Hz), 129.8 (d, *J* = 95.9 Hz), 129.0 (d, *J* = 11.6 Hz), 128.6 (d, *J* = 12.1 Hz), 128.41, 128.36, 128.3, 126.3, 123.5, 121.8 (d, *J* = 11.1 Hz), 116.4, 115.3, 58.5 (br), 30.3, 24.6. ^{31}P NMR (283 MHz, DMSO) δ 44.73. HRMS (ESI) *m/z* calcd for $C_{29}H_{24}BrN_2O_3P$, $[M+Na]^+$: 581.0606, found: 581.0601.

Ethyl 2-((4'-acetamido-3'-(3-(diphenylphosphoryl)-2-oxo-1,2,3,4-tetrahydroquinolin-3-yl)-[1,1'-biphenyl]-4-yl)oxy)-2-methylpropanoate

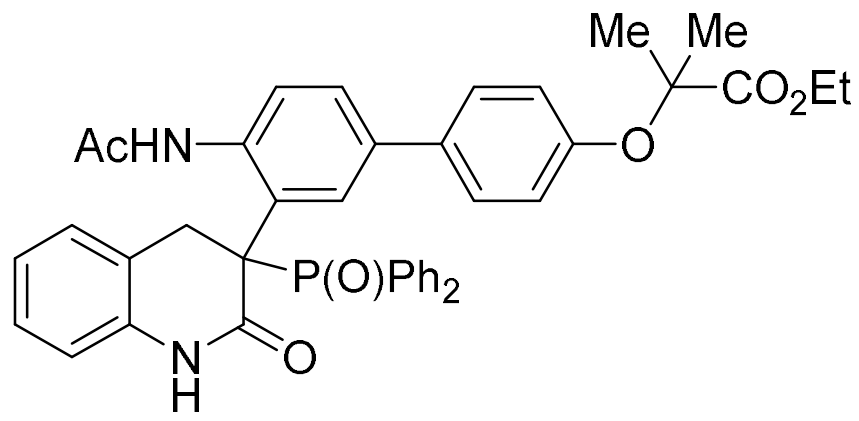

5g

Yellow solid, 142.0 mg, 69% yield, ^{1}H NMR (400 MHz, Chloroform-*d*) δ 11.47 (s, 1H), 8.69 (s, 1H), 8.18 (s, 2H), 7.97 – 7.82 (m, 2H), 7.71 (d, *J* = 8.4 Hz, 1H), 7.60 – 7.43 (m, 3H), 7.42 – 7.26 (m, 3H), 7.22 (d, *J* = 8.5 Hz, 1H), 7.10 – 7.00 (m, 2H), 7.00 – 6.82 (m, 4H), 6.72 (d, *J* = 7.3 Hz, 2H), 6.43 (d, *J* = 5.8 Hz, 1H), 4.46 (s, 1H), 4.26 (q, *J* = 6.8 Hz, 2H), 3.39 (d, *J* = 13.5 Hz, 1H), 2.43 (s, 3H), 1.60 (s, 6H), 1.28 (t, *J* = 6.2 Hz, 3H). ^{13}C NMR (101 MHz, Chloroform-*d*) δ 174.2, 170.0, 168.5, 154.8, 136.9 (d, *J* = 2.1 Hz), 136.6 (d, *J* = 3.2 Hz), 135.2, 134.1, 133.3 (d, *J* = 9.1 Hz), 133.0 (d, *J* = 9.6 Hz), 132.6 (d, *J* = 2.9 Hz), 132.0 (d, *J* = 3.3 Hz), 129.8 (d, *J* = 104.7 Hz), 129.4 (d, *J* =

95.4 Hz), 128.6, 128.5 (d, J = 12.0 Hz), 128.1, 127.9, 127.8 (d, J = 12.4 Hz), 127.54 (2C), 127.48, 124.2, 123.1, 122.0 (d, J = 11.6 Hz), 119.1, 114.8, 79.1, 61.5, 58.7 (d, J = 71.0 Hz), 25.5, 25.3, 24.6, 14.1. ^{31}P NMR (162 MHz, Chloroform-*d*) δ 44.22. HRMS (ESI) *m/z* calcd for $C_{41}H_{39}N_2O_6P$, $[M+Na]^+$: 709.2443, found: 709.2440.

***N*-(2-(3-(Bis(3,5-dimethylphenyl)phosphoryl)-2-oxo-1,2,3,4-tetrahydroquinolin-3-yl)phenyl)acetamide**

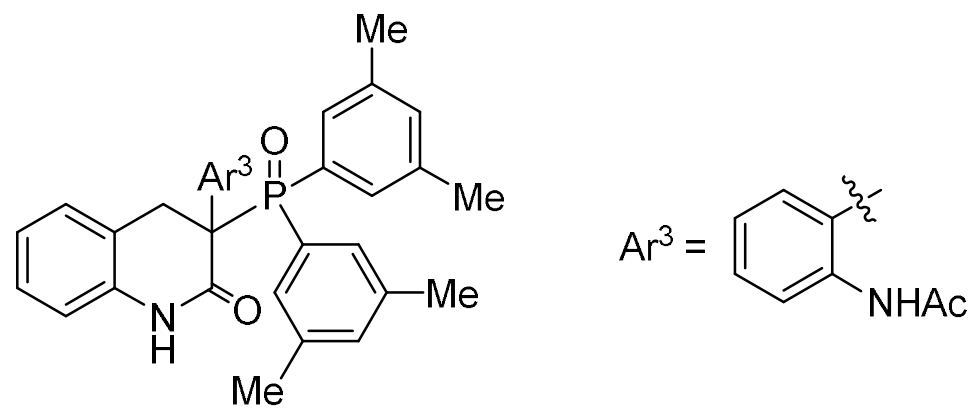

5h

Yellow solid, 115.3 mg, 72% yield, ^{1}H NMR (400 MHz, Chloroform-*d*) δ 11.70 (s, 1H), 8.79 (s, 1H), 7.87 – 7.67 (m, 3H), 7.27 – 7.19 (m, 2H), 7.12 (t, J = 7.9 Hz, 1H), 7.09 – 7.01 (m, 2H), 7.00 – 6.92 (m, 2H), 6.88 (t, J = 7.4 Hz, 1H), 6.72 (t, J = 7.8 Hz, 1H), 6.49 (d, J = 7.7 Hz, 1H), 4.36 (s, 1H), 3.38 (d, J = 15.6 Hz, 1H), 2.47 – 2.33 (m, 9H), 2.19 (s, 6H). ^{13}C NMR (151 MHz, Chloroform-*d*) δ 170.7, 168.5, 138.3, 138.2 (d, J = 7.8 Hz), 137.3 (d, J = 8.2 Hz), 135.4, 134.4, 133.6, 131.2 (d, J = 9.4 Hz), 130.5 (d, J = 100.8 Hz), 130.34, 130.33 (d, J = 9.0 Hz), 129.3, 129.0 (d, J = 94.7 Hz), 128.4, 127.9, 127.3, 124.5, 124.3, 123.2, 122.3 (d, J = 11.2 Hz), 114.5, 58.6 (d, J = 65.2 Hz), 30.5, 24.7, 21.5, 21.3. ^{31}P NMR (243 MHz, Chloroform-*d*) δ 45.62. HRMS (ESI) *m/z* calcd for $C_{33}H_{33}N_2O_3P$, $[M+H]^+$: 537.2307, found: 537.2312.

***N*-(2-(3-(Bis(4-methoxyphenyl)phosphoryl)-2-oxo-1,2,3,4-tetrahydroquinolin-3-yl)phenyl)acetamide**

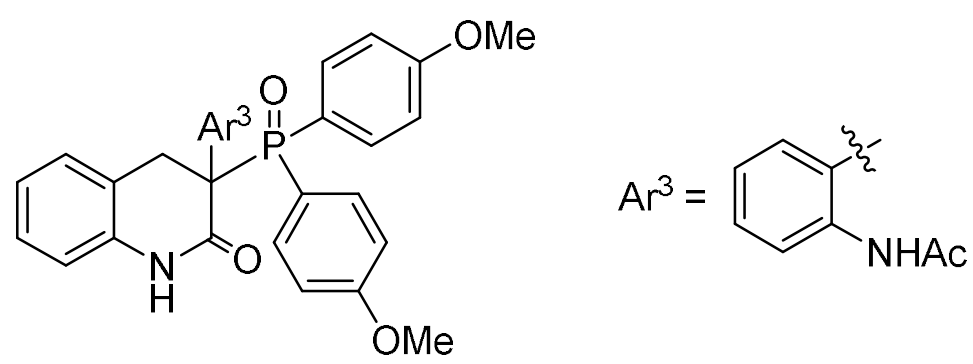

5i

White solid, 116.6 mg, 73% yield, ^{1}H NMR (400 MHz, Chloroform-*d*) δ 11.73 (s, 1H), 8.92 (s, 1H), 8.28 – 7.96 (m, 2H), 7.78 – 7.65 (m, 3H), 7.11 – 6.96 (m, 4H), 6.94 – 6.82 (m, 3H), 6.78 (d, J = 8.4 Hz, 2H), 6.62 (t, J = 7.8 Hz, 1H), 6.42 (d, J = 7.7 Hz, 1H), 4.36 (s, 1H), 3.85 (s, 3H), 3.76 (s, 3H), 3.36 (d, J = 10.4 Hz, 1H), 2.42 (s, 3H). ^{13}C NMR (101 MHz, Chloroform-*d*) δ 170.7, 168.3, 162.9 (d, J = 2.8 Hz), 162.3 (d, J = 2.7 Hz), 137.8, 135.4 (d, J = 10.5 Hz), 135.2, 134.6 (d, J = 11.0 Hz), 130.0 (d, J = 2.8 Hz), 128.7 (d, J = 2.0 Hz), 128.2, 127.7, 127.0, 124.6, 124.2, 123.0 (d, J = 3.2 Hz), 122.2 (d, J = 7.5 Hz), 121.5 (d, J = 107.8 Hz), 120.5 (d, J = 102.7 Hz), 114.5, 114.0

(d, J = 12.8 Hz), 113.3 (d, J = 13.5 Hz), 58.6 (d, J = 64.9 Hz), 55.4, 55.2, 30.4, 24.6. ^{31}P NMR (162 MHz, Chloroform-*d*) δ 43.95. HRMS (ESI) *m/z* calcd for $C_{31}H_{29}N_2O_5P$, $[M+Na]^+$: 563.1712, found: 563.1706.

2-Methyl-2'-(phenylthio)-4',5'-dihydrospiro[indole-3,3'-pyrrole]

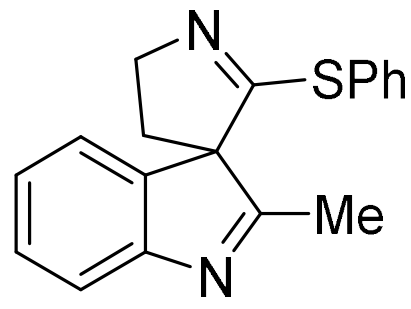

7a

White solid, 45.5 mg, 52% yield, ^{1}H NMR (400 MHz, Chloroform-*d*) δ 7.60 (d, J = 7.7 Hz, 1H), 7.49 – 7.40 (m, 3H), 7.40 – 7.33 (m, 4H), 7.31 (d, J = 7.3 Hz, 1H), 4.38 – 4.25 (m, 1H), 4.25 – 4.14 (m, 1H), 2.60 – 2.32 (m, 5H). ^{13}C NMR (101 MHz, Chloroform-*d*) δ 180.6, 171.9, 155.6, 140.0, 134.6, 129.4, 129.3, 129.0, 128.2, 126.2, 122.3, 120.3, 75.7, 61.1, 34.4, 16.4. HRMS (ESI) *m/z* calcd for $C_{18}H_{16}N_2S$, $[M+H]^+$: 293.1112, found: 293.1112.

2-Methyl-2'-(*p*-tolylthio)-4',5'-dihydrospiro[indole-3,3'-pyrrole]

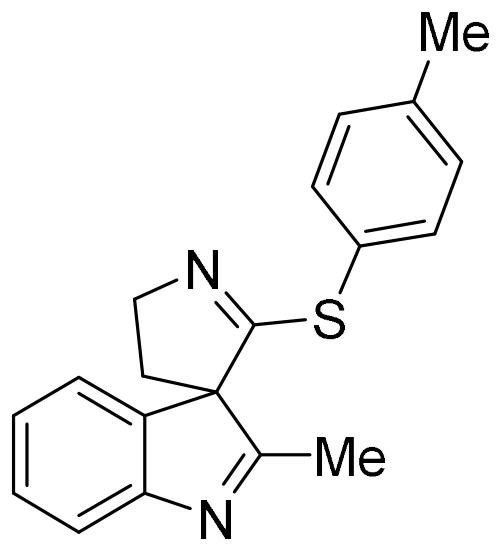

7b

White solid, 55.0 mg, 60% yield, ^{1}H NMR (400 MHz, Chloroform-*d*) δ 7.60 (d, J = 7.7 Hz, 1H), 7.43 (t, J = 7.5 Hz, 1H), 7.37 – 7.25 (m, 4H), 7.17 (d, J = 7.9 Hz, 2H), 4.38 – 4.24 (m, 1H), 4.22 – 4.12 (m, 1H), 2.62 – 2.22 (m, 8H). ^{13}C NMR (101 MHz, Chloroform-*d*) δ 180.7, 172.3, 155.6, 140.1, 139.7, 134.7, 130.1, 129.0, 126.1, 124.6, 122.3, 120.3, 75.7, 61.1, 34.4, 21.4, 16.4. HRMS (ESI) *m/z* calcd for $C_{19}H_{18}N_2S$, $[M+H]^+$: 307.1269, found: 307.1271.

2'-((4-Methoxyphenyl)thio)-2-methyl-4',5'-dihydrospiro[indole-3,3'-pyrrole]

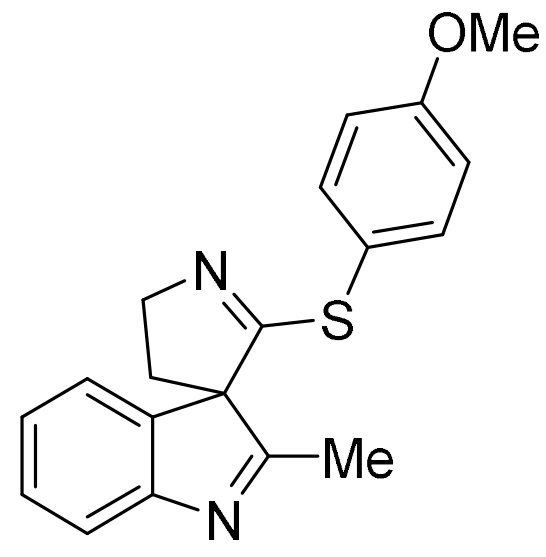

7c

White solid, 60.8 mg, 63% yield, ^{1}H NMR (400 MHz, Chloroform-*d*) δ 7.59 (d, *J* = 7.7 Hz, 1H), 7.46 – 7.37 (m, 1H), 7.38 – 7.32 (m, 3H), 7.29 (t, *J* = 8.1 Hz, 1H), 6.88 (d, *J* = 8.8 Hz, 2H), 4.34 – 4.24 (m, 1H), 4.24 – 4.11 (m, 1H), 3.80 (s, 3H), 2.51 (t, *J* = 7.5 Hz, 1H), 2.47 – 2.34 (m, 4H). ^{13}C NMR (101 MHz, Chloroform-*d*) δ 180.7, 172.8, 160.6, 155.6, 140.2, 136.4, 129.0, 126.1, 122.3, 120.3, 118.5, 114.9, 75.6, 61.0, 55.3, 34.5, 16.4. HRMS (ESI) *m/z* calcd for $C_{19}H_{18}N_2OS$, $[M+H]^+$: 323.1218, found: 323.1218.

2'-((4-Chlorophenyl)thio)-2-methyl-4',5'-dihydrospiro[indole-3,3'-pyrrole]

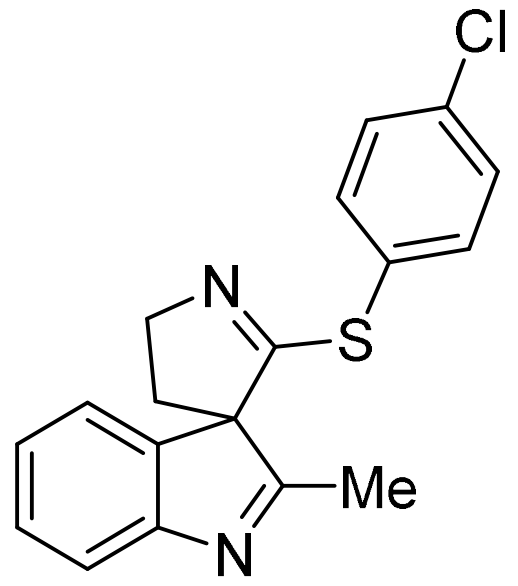

7d

White solid, 50.8 mg, 53% yield, ^{1}H NMR (400 MHz, Chloroform-*d*) δ 7.60 (d, *J* = 7.7 Hz, 1H), 7.48 – 7.37 (m, 3H), 7.37 – 7.26 (m, 4H), 4.37 – 4.25 (m, 1H), 4.25 – 4.14 (m, 1H), 2.59 – 2.33 (m, 5H). ^{13}C NMR (101 MHz, Chloroform-*d*) δ 180.4, 171.5, 155.6, 139.9, 135.8, 135.7, 129.5, 129.1, 126.7, 126.2, 122.3, 120.4, 75.7, 61.1, 34.3, 16.4. HRMS (ESI) *m/z* calcd for $C_{18}H_{15}ClN_2S$, $[M+H]^+$: 327.0723, found: 327.0729.

2-Methyl-2'-(naphthalen-2-ylthio)-4',5'-dihydrospiro[indole-3,3'-pyrrole]

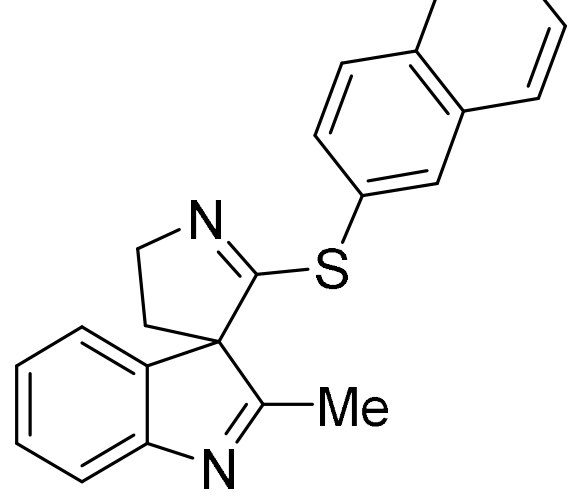

7e

White solid, 42.0 mg, 41% yield, ^{1}H NMR (400 MHz, Chloroform-*d*) δ 7.97 (s, 1H), 7.88 – 7.75 (m, 3H), 7.61 (d, *J* = 7.6 Hz, 1H), 7.56 – 7.36 (m, 5H), 7.32 (t, *J* = 7.4 Hz, 1H), 4.37 – 4.25 (m, 1H), 4.25 – 4.12 (m, 1H), 2.61 – 2.36 (m, 5H). ^{13}C NMR (101 MHz, Chloroform-*d*) δ 180.6, 172.0, 155.6, 140.1, 134.2, 133.5, 133.3, 131.2, 129.1, 128.9, 128.0, 127.8, 127.2, 126.6, 126.2, 125.5, 122.3, 120.4, 75.8, 61.2, 34.3, 16.5. HRMS (ESI) *m/z* calcd for $C_{22}H_{18}N_2S$, $[M+H]^+$: 343.1269, found: 343.1266.

***N*-(2-(2-Oxopyrrolidin-3-yl)phenyl)acetamide**

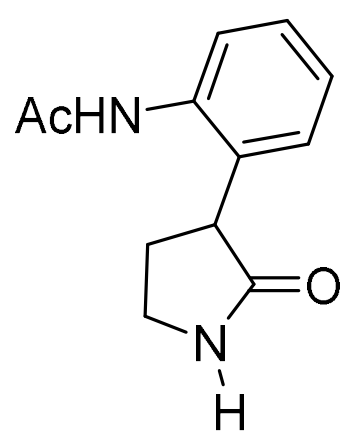

8

White solid, 18.3 mg, 56% yield, ^{1}H NMR (400 MHz, Chloroform-*d*) δ 9.69 (s, 1H), 7.76 (d, *J* = 8.0 Hz, 1H), 7.36 – 7.12 (m, 3H), 6.17 (s, 1H), 3.92 (t, *J* = 7.9 Hz, 1H), 3.73 – 3.37 (m, 2H), 2.70 – 2.50 (m, 2H), 2.22 (s, 3H). ^{13}C NMR (101 MHz, Chloroform-*d*) δ 179.3, 169.1, 137.1, 129.8, 127.9, 126.1, 125.5, 125.4, 42.6, 41.2, 26.3, 24.3. HRMS (ESI) *m/z* calcd for $C_{12}H_{14}N_2O_2$, $[M+Na]^+$: 241.0953, found: 241.0964.

***tert*-Butyl (3-(2-aminophenyl)-3-oxopropyl)carbamate**

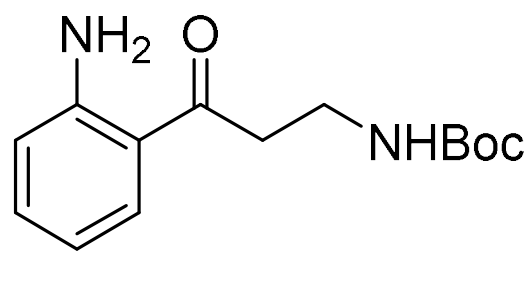

9

White solid, 23.6 mg, 45% yield, ^{1}H NMR (400 MHz, Chloroform-*d*) δ 7.73 (d, *J* = 8.0 Hz, 1H), 7.32 – 7.26 (m, 1H), 6.67 (dd, *J* = 7.7, 5.7 Hz, 2H), 6.30 (s, 2H), 5.16 (s, 1H), 3.60 – 3.50 (m, 2H), 3.19 (t, *J* = 5.7 Hz, 2H), 1.45 (s, 9H). ^{13}C NMR (101 MHz, Chloroform-*d*) δ 201.4, 156.0, 150.3, 134.6, 131.1, 117.8, 117.4, 116.1, 79.1, 39.1, 35.7, 28.4. HRMS (ESI) *m/z* calcd for $C_{14}H_{20}N_2O_3$, $[M+Na]^+$: 287.1372, found: 287.1385.

***N*-(2-(3-(Diphenylphosphoryl)-1-methyl-2-oxopyrrolidin-3-yl)phenyl)-N-methylacetamide**

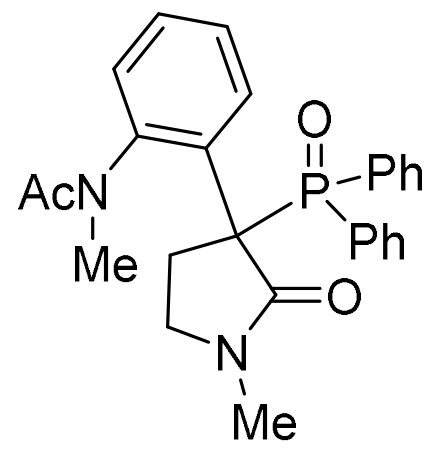

10

White solid, 54.8 mg, 82% yield, ^{1}H NMR (400 MHz, Chloroform-*d*) δ 8.34 – 8.10 (m, 3H), 7.93 – 7.73 (m, 2H), 7.53 – 6.99 (m, 9H), 3.81 – 3.64 (m, 1H), 3.28 – 3.15 (m, 1H), 2.97 (s, 3H), 2.71 – 2.56 (m, 1H), 2.40 – 2.20 (m, 4H), 1.98 (s, 3H). ^{13}C NMR (101 MHz, Chloroform-*d*) δ 173.2, 170.9, 141.7 (d, *J* = 8.2 Hz), 138.3, 133.2 (d, *J* = 9.9 Hz), 132.5 (d, *J* = 9.0 Hz), 132.3 (d, *J* = 2.7 Hz), 132.0 (d, *J* = 7.3 Hz), 131.7 (d, *J* = 3.1 Hz), 130.84 (d, *J* = 100.4 Hz), 130.83, 130.78 (d, *J* = 95.6 Hz), 129.0, 128.4, 128.1 (d, *J* = 6.6 Hz), 128.0 (d, *J* = 5.7 Hz), 58.7 (d, *J* = 58.5 Hz), 47.1, 37.1, 29.9, 29.5, 23.4. ^{31}P NMR (162 MHz, $CDCl_3$) δ 37.88. HRMS (ESI) *m/z* calcd for $C_{26}H_{27}N_2O_3P$, $[M+Na]^+$: 469.1657, found: 469.1658.

3-(Diphenylphosphoryl)-1-methyl-3-(2-(methylamino)phenyl)pyrrolidin-2-one

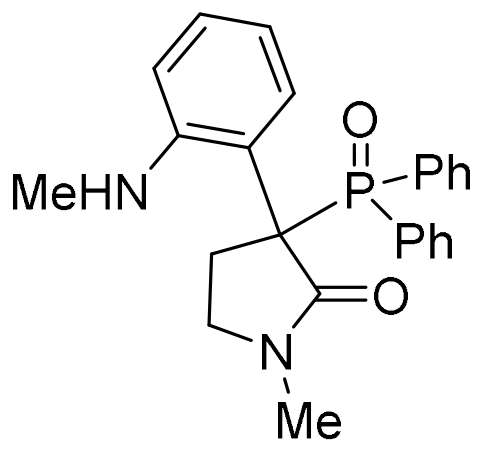

11

White solid, 46.2 mg, 93% yield, ^{1}H NMR (400 MHz, Chloroform-*d*) δ 8.11 (dd, *J* = 11.6, 7.6 Hz, 2H), 7.94 (dd, *J* = 11.0, 7.5 Hz, 2H), 7.58 – 7.44 (m, 3H), 7.42 – 7.25 (m, 3H), 7.06 (t, *J* = 7.5 Hz, 1H), 7.01 – 6.91 (m, 1H), 6.57 – 6.44 (m, 2H), 3.45 – 3.32 (m, 1H), 3.25 – 3.15 (m, 1H), 3.02 – 2.92 (m, 1H), 2.91 – 2.69 (m, 4H), 2.57 (s, 3H). ^{13}C NMR (101 MHz, Chloroform-*d*) δ 171.9 (d, *J* = 2.2 Hz), 148.5 (d, *J* = 2.5 Hz), 132.7 (d, *J* = 9.3 Hz, 2C), 132.0 (d, *J* = 2.8 Hz), 131.8 (d, *J* = 2.9 Hz), 130.9 (d, *J* = 92.4 Hz), 129.2 (d, *J* = 101.5 Hz), 129.0 (d, *J* = 5.5 Hz), 128.6 (d, *J* = 2.0 Hz), 128.0 (d, *J* = 11.6 Hz), 127.7 (d, *J* = 12.5 Hz), 121.4 (d, *J* = 3.4 Hz), 115.7, 111.3, 60.3 (d, *J* = 61.2 Hz), 47.0 (d, *J* = 4.1 Hz), 30.5, 30.0, 26.3. ^{31}P NMR (162 MHz, $CDCl_3$) δ 37.88. HRMS (ESI) *m/z* calcd for $C_{24}H_{25}N_2O_2P$, $[M+Na]^+$: 427.1551, found: 427.1550.

(2-Methyl-4',5'-dihydrospiro[indole-3,3'-pyrrol]-2'-yl)diphenylphosphine oxide

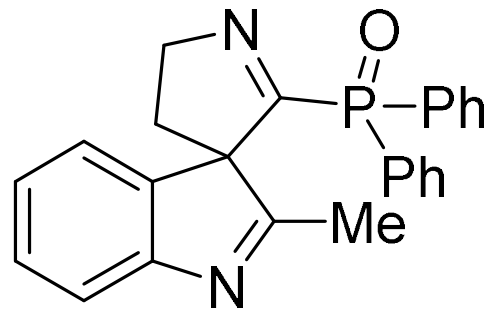

12

White solid, 36.7 mg, 32% yield, ^{1}H NMR (400 MHz, Chloroform-*d*) δ 7.81 (dd, *J* = 12.2, 7.6 Hz, 2H), 7.65 – 7.39 (m, 8H), 7.33 – 7.29 (m, 1H), 7.23 (t, *J* = 7.5 Hz, 1H), 6.88 (t, *J* = 7.4 Hz, 1H), 6.76 (d, *J* = 7.4 Hz, 1H), 4.57 (td, *J* = 7.2, 3.5 Hz, 2H), 2.50 – 2.41 (m, 1H), 2.32 (s, 3H), 2.27 – 2.17 (dt, *J* = 13.5, 6.8 Hz, 1H). ^{13}C NMR (101 MHz, Chloroform-*d*) δ 180.0, 176.7 (d, *J* = 110.4 Hz), 155.5, 139.5, 132.3 (d, *J* = 2.7 Hz), 132.0 (d, *J* = 2.9 Hz), 131.9 (d, *J* = 9.6 Hz), 131.3 (d, *J* = 9.9 Hz), 131.0 (d, *J* = 105.8 Hz), 130.4 (d, *J* = 103.3 Hz), 128.6, 128.4 (d, *J* = 12.4 Hz), 128.2 (d, *J* = 12.4 Hz), 125.2, 121.9, 120.3, 64.3, 64.1, 34.1 (d, *J* = 2.0 Hz), 17.0. ^{31}P NMR (162 MHz, $CDCl_3$) δ 18.10. HRMS (ESI) *m/z* calcd for $C_{24}H_{21}N_2OP$, $[M+Na]^+$: 407.1289, found: 407.1292.

5. X-ray crystallographic data of 3a

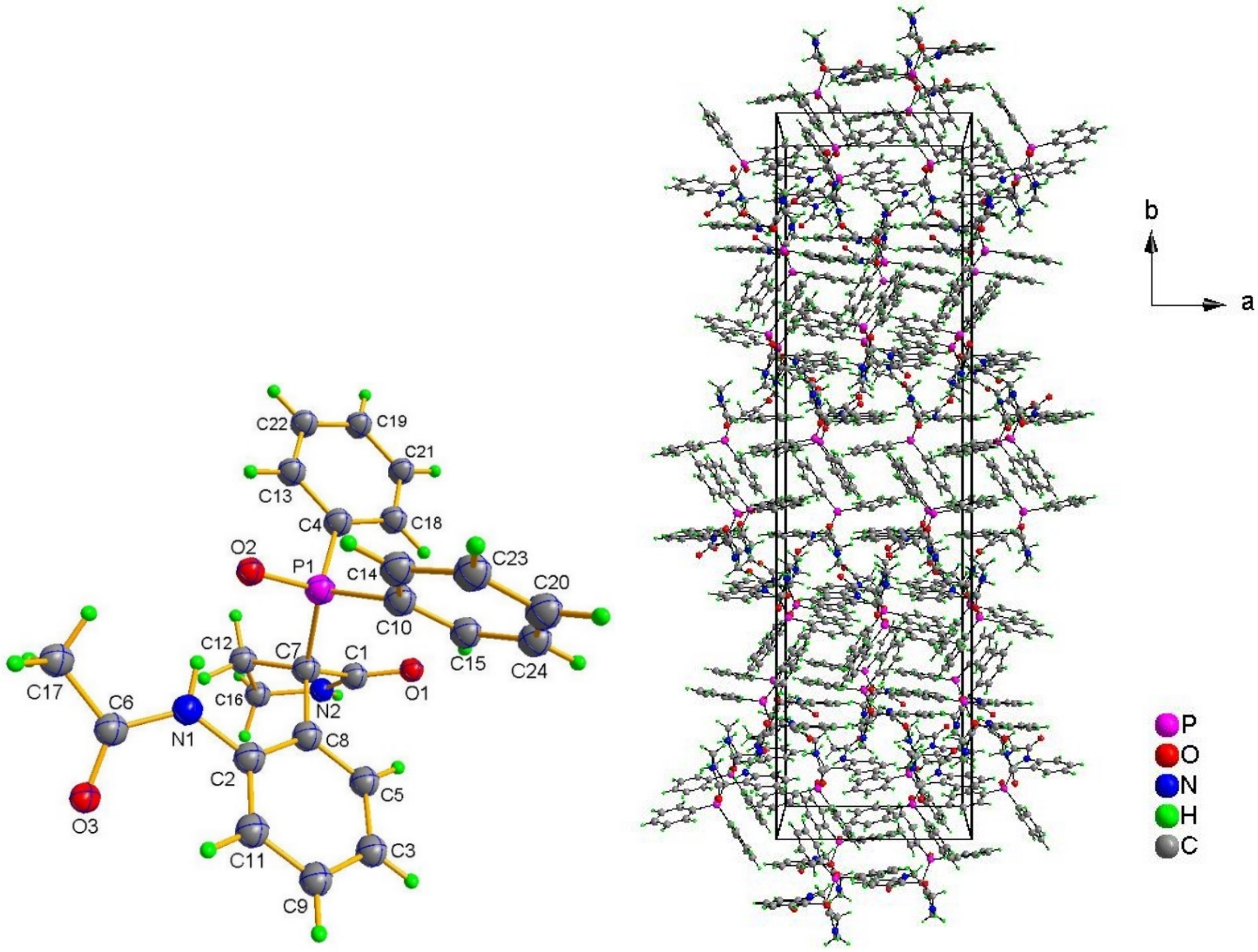

Supplementary Fig. 31 X-ray crystallography of **3a**. CCDC: 2368727.

Supplementary Table 4 Crystal data and structure refinement for **3a**

Identification code	**3a**
Empirical formula	$C_{24}H_{23}N_2O_3P$
Formula weight	418.41
Temperature/K	293(2)
Crystal system	orthorhombic
Space group	Fdd2
a/Å	14.1958(3)
b/Å	52.9160(12)
c/Å	10.9054(2)
α/°	90
β/°	90
γ/°	90
Volume/Å^3	8192.0(3)
Z	16
ρ_{calc}g/cm^3	1.357
μ/mm^{-1}	1.428
F(000)	3520.0
Crystal size/mm^3	0.200 × 0.200 × 0.100
Radiation	CuKα (λ = 1.54184)
2Θ range for data collection/°	6.682 to 153.128
Index ranges	$-15 \leq h \leq 17$, $-65 \leq k \leq 48$, $-10 \leq l \leq 13$
Reflections collected	6273
Independent reflections	2945 [R_{int} = 0.0206, R_{sigma} = 0.0293]
Data/restraints/parameters	2945/1/272
Goodness-of-fit on F^2	1.046
Final R indexes [I>=2σ (I)]	R_1 = 0.0312, wR_2 = 0.0833
Final R indexes [all data]	R_1 = 0.0316, wR_2 = 0.0842

Largest diff. peak/hole / e Å^{-3}	0.29/-0.18
Flack parameter	0.071(14)

Supplementary Table 5 Fractional atomic coordinates (×10^4) and equivalent isotropic displacement parameters ($Å^2×10^3$) for **3a**. U_{eq} is defined as 1/3 of of the trace of the orthogonalised U_{IJ} tensor.

Atom	*x*	*y*	*z*	U(eq)
P(1)	3025.8(4)	4490.5(2)	5535.5(5)	33.86(15)
O(1)	2665.8(14)	4296.9(3)	8383.4(16)	45.9(4)
O(2)	3060.3(13)	4469.9(3)	4164.6(17)	44.6(5)
O(3)	796.9(13)	3884.0(4)	2405.2(18)	48.7(4)
N(1)	1665.7(14)	4134.1(4)	3675.0(18)	35.4(4)
N(2)	3057.4(15)	3907.1(4)	7686(2)	43.4(5)
C(1)	2764.8(16)	4144.1(4)	7549(2)	34.6(5)
C(2)	1090.3(15)	4141.3(4)	4726(2)	33.3(4)
C(3)	-109.7(18)	4188.2(5)	6738(2)	43.1(6)
C(4)	4202.8(18)	4548.8(5)	6104(2)	39.7(5)
C(5)	855.4(18)	4199.7(4)	6889(2)	36.5(5)
C(6)	1481.7(16)	4018.3(4)	2590(2)	35.6(5)
C(7)	2558.3(16)	4187.5(4)	6158(2)	32.0(4)
C(8)	1483.2(15)	4173.7(4)	5913(2)	30.4(4)
C(9)	-481.5(16)	4155.2(5)	5575(3)	45.4(6)
C(10)	2272.2(16)	4749.0(4)	6005(2)	38.3(5)
C(11)	116.9(18)	4132.8(5)	4583(2)	42.4(5)
C(12)	3126.7(16)	3965.9(4)	5581(3)	38.5(5)
C(13)	4922(2)	4546.3(6)	5254(3)	57.8(7)
C(14)	2060(2)	4926.9(6)	5118(3)	58.7(7)
C(15)	1923(2)	4783.6(5)	7188(3)	49.0(6)
C(16)	3120(2)	3763.0(5)	6566(3)	48.8(6)
C(17)	2218(2)	4062.4(6)	1635(3)	50.2(6)
C(18)	4405(2)	4610.1(6)	7309(3)	56.7(7)
C(19)	6030(2)	4666.6(6)	6802(4)	61.2(8)

C(20)	1152(3)	5164.6(6)	6563(4)	66.8(9)
C(21)	5322(2)	4668.5(7)	7654(3)	64.5(9)
C(22)	5837(2)	4604.1(7)	5606(4)	70.5(9)
C(23)	1503(3)	5134.6(7)	5421(4)	78.5(11)
C(24)	1363(2)	4989.3(5)	7460(3)	58.0(7)

Supplementary Table 6 Anisotropic displacement parameters (Å^2×10^3) for **3a**. The Anisotropic displacement factor exponent takes the form: $-2\pi^2[h^2a^{*2}U_{11}+2hka^*b^*U_{12}+\ldots]$.

Atom	U_{11}	U_{22}	U_{33}	U_{23}	U_{13}	U_{12}
P(1)	36.0(3)	38.8(3)	26.8(3)	0.7(2)	-0.8(2)	-6.7(2)
O(1)	61.4(10)	48.6(9)	27.7(8)	-3.9(7)	-3.0(8)	8.1(8)
O(2)	53.3(11)	53.7(10)	26.8(9)	1.7(7)	1.0(7)	-17.4(8)
O(3)	48.5(9)	60.9(10)	36.8(9)	-15.5(8)	5.3(9)	-16.3(8)
N(1)	30.5(9)	49.0(10)	26.8(9)	-5.1(8)	3.0(8)	-6.1(8)
N(2)	53.9(12)	45.3(10)	31.1(11)	4.6(9)	-3.4(9)	10.8(9)
C(1)	34.8(10)	41.0(10)	28.1(11)	2.8(9)	-1.5(9)	0.4(8)
C(2)	34.7(10)	36.3(9)	29.1(10)	-3.5(8)	3.5(9)	-3.2(8)
C(3)	38.6(12)	52.4(13)	38.2(13)	-2.3(11)	12.2(11)	-2.2(10)
C(4)	37.2(11)	41.1(10)	40.9(13)	-1.3(10)	-1.3(11)	-5.2(9)
C(5)	41.2(12)	40.2(10)	28.0(11)	0.2(9)	6.3(10)	-0.8(9)
C(6)	36.4(11)	41.8(10)	28.7(11)	-4.0(9)	2.8(9)	-1.3(9)
C(7)	35.3(11)	36.6(10)	24.1(10)	-0.2(8)	2.0(9)	-0.9(8)
C(8)	33.9(11)	30.2(9)	27.3(11)	-0.1(8)	3.2(9)	-1.3(7)
C(9)	31.9(11)	60.1(14)	44.3(14)	-5.3(13)	4.1(11)	-4.1(9)
C(10)	37.3(11)	37.7(10)	39.9(12)	1.1(9)	-5.5(11)	-6.6(8)
C(11)	35.5(11)	57.9(13)	33.9(12)	-6.4(11)	0.8(10)	-5.3(10)
C(12)	39.5(11)	43.4(10)	32.6(11)	-1.0(10)	5.4(10)	5.2(9)
C(13)	45.2(14)	80.9(18)	47.3(16)	0.2(14)	2.8(12)	-10.6(13)
C(14)	68.1(18)	62.9(17)	45.0(16)	10.1(13)	-7.3(14)	7.8(14)
C(15)	59.9(15)	44.7(12)	42.5(14)	2.9(11)	1.4(13)	7.7(11)
C(16)	61.2(16)	44.3(12)	41.0(14)	1.6(11)	3.2(12)	11.8(11)
C(17)	51.4(15)	62.8(15)	36.4(14)	-9.2(12)	13.5(12)	-9.5(12)
C(18)	47.1(14)	79.6(19)	43.5(15)	-13.3(15)	-1.3(13)	-10.3(13)
C(19)	39.2(13)	68.0(17)	76(2)	1.5(16)	-9.7(15)	-10.6(12)

C(20)	68.6(19)	53.8(15)	78(2)	1.5(16)	-7.0(18)	20.4(14)
C(21)	57.2(17)	77(2)	59(2)	-13.1(16)	-16.0(15)	-13.6(15)
C(22)	42.2(14)	98(2)	71(2)	1(2)	7.5(17)	-12.8(15)
C(23)	93(3)	70.9(19)	71(2)	23.1(19)	-12(2)	27.0(19)
C(24)	65.7(17)	51.9(14)	56.2(18)	-4.5(13)	3.2(15)	11.4(13)

Supplementary Table 7 Bond lengths for **3a**.

Atom	Atom	Length/Å	Atom	Atom	Length/Å
P(1)	O(2)	1.4998(19)	C(5)	C(8)	1.395(3)
P(1)	C(4)	1.809(3)	C(6)	C(17)	1.494(3)
P(1)	C(10)	1.811(2)	C(7)	C(8)	1.551(3)
P(1)	C(7)	1.863(2)	C(7)	C(12)	1.556(3)
O(1)	C(1)	1.225(3)	C(9)	C(11)	1.380(4)
O(3)	C(6)	1.221(3)	C(10)	C(14)	1.383(4)
N(1)	C(6)	1.358(3)	C(10)	C(15)	1.393(4)
N(1)	C(2)	1.408(3)	C(12)	C(16)	1.519(4)
N(2)	C(1)	1.330(3)	C(13)	C(22)	1.389(4)
N(2)	C(16)	1.442(4)	C(14)	C(23)	1.393(5)
C(1)	C(7)	1.562(3)	C(15)	C(24)	1.380(4)
C(2)	C(11)	1.391(3)	C(18)	C(21)	1.390(4)
C(2)	C(8)	1.420(3)	C(19)	C(21)	1.368(5)
C(3)	C(5)	1.381(4)	C(19)	C(22)	1.373(6)
C(3)	C(9)	1.386(4)	C(20)	C(23)	1.350(6)
C(4)	C(13)	1.379(4)	C(20)	C(24)	1.380(5)
C(4)	C(18)	1.385(4)			

Supplementary Table 8 Bond angles for **3a**.

Atom	Atom	Atom	Angle/˚	Atom	Atom	Atom	Angle/˚
O(2)	P(1)	C(4)	108.88(12)	C(12)	C(7)	C(1)	100.63(18)
O(2)	P(1)	C(10)	110.87(12)	C(8)	C(7)	P(1)	109.19(14)
C(4)	P(1)	C(10)	108.66(11)	C(12)	C(7)	P(1)	108.46(15)
O(2)	P(1)	C(7)	108.19(10)	C(1)	C(7)	P(1)	114.42(15)
C(4)	P(1)	C(7)	110.56(11)	C(5)	C(8)	C(2)	117.2(2)
C(10)	P(1)	C(7)	109.67(10)	C(5)	C(8)	C(7)	119.5(2)
C(6)	N(1)	C(2)	127.59(19)	C(2)	C(8)	C(7)	123.29(19)
C(1)	N(2)	C(16)	115.0(2)	C(11)	C(9)	C(3)	119.6(2)
O(1)	C(1)	N(2)	125.1(2)	C(14)	C(10)	C(15)	118.7(2)
O(1)	C(1)	C(7)	127.1(2)	C(14)	C(10)	P(1)	116.4(2)
N(2)	C(1)	C(7)	107.84(19)	C(15)	C(10)	P(1)	124.86(19)
C(11)	C(2)	N(1)	119.0(2)	C(9)	C(11)	C(2)	121.4(2)
C(11)	C(2)	C(8)	119.7(2)	C(16)	C(12)	C(7)	104.1(2)
N(1)	C(2)	C(8)	121.17(19)	C(4)	C(13)	C(22)	120.3(3)
C(5)	C(3)	C(9)	119.5(2)	C(10)	C(14)	C(23)	119.6(3)
C(13)	C(4)	C(18)	119.2(3)	C(24)	C(15)	C(10)	120.5(3)
C(13)	C(4)	P(1)	116.9(2)	N(2)	C(16)	C(12)	103.0(2)
C(18)	C(4)	P(1)	123.8(2)	C(4)	C(18)	C(21)	120.2(3)
C(3)	C(5)	C(8)	122.6(2)	C(21)	C(19)	C(22)	120.0(3)
O(3)	C(6)	N(1)	124.0(2)	C(23)	C(20)	C(24)	119.6(3)
O(3)	C(6)	C(17)	122.2(2)	C(19)	C(21)	C(18)	120.2(3)
N(1)	C(6)	C(17)	113.7(2)	C(19)	C(22)	C(13)	120.2(3)
C(8)	C(7)	C(12)	113.90(18)	C(20)	C(23)	C(14)	121.4(3)
C(8)	C(7)	C(1)	110.15(18)	C(15)	C(24)	C(20)	120.1(3)

Supplementary Table 9 Hydrogen atom coordinates (Å×10^4) and isotropic displacement parameters (Å^2×10^3) for **3a**.

Atom	*x*	*y*	*z*	U(eq)
H(1)	2195.65	4212.13	3726.21	43
H(2)	3198.95	3844.38	8389.69	52
H(3)	-507.04	4202.38	7412.92	52
H(5)	1096.13	4225.96	7672.1	44
H(9)	-1130.41	4148.19	5462.17	55
H(11)	-135.91	4111.54	3803.46	51
H(12A)	2826.65	3905.87	4836.9	46
H(12B)	3765.48	4017.91	5391.11	46
H(13)	4793.35	4505.66	4440.79	69
H(14)	2286.84	4908	4323.53	70
H(15)	2068.56	4667.3	7797.93	59
H(16A)	2581.93	3651.64	6473.67	59
H(16B)	3693.03	3663.34	6543.29	59
H(17A)	2560.05	3908.69	1493.55	75
H(17B)	2643.9	4191.33	1910.22	75
H(17C)	1922.26	4115.61	885.99	75
H(18)	3925.49	4612.21	7890.63	68
H(19)	6641.38	4707.73	7033.51	73
H(20)	771.22	5302.57	6744.58	80
H(21)	5455.07	4708.89	8465.61	77
H(22)	6320.15	4600.48	5030.94	85
H(23)	1369.98	5255.07	4824.77	94
H(24)	1126.36	5009.8	8248.82	70

6. NMR spectra

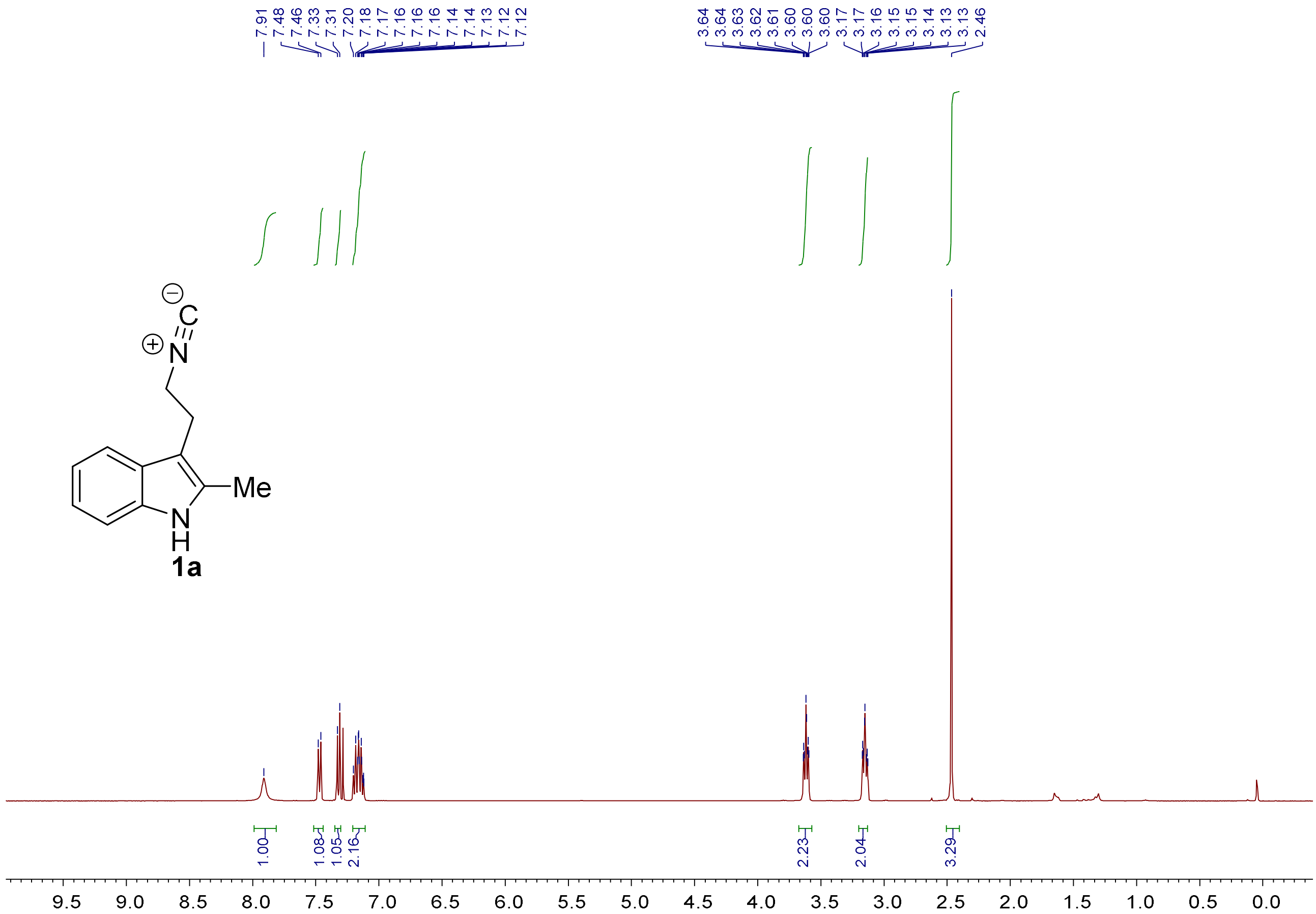

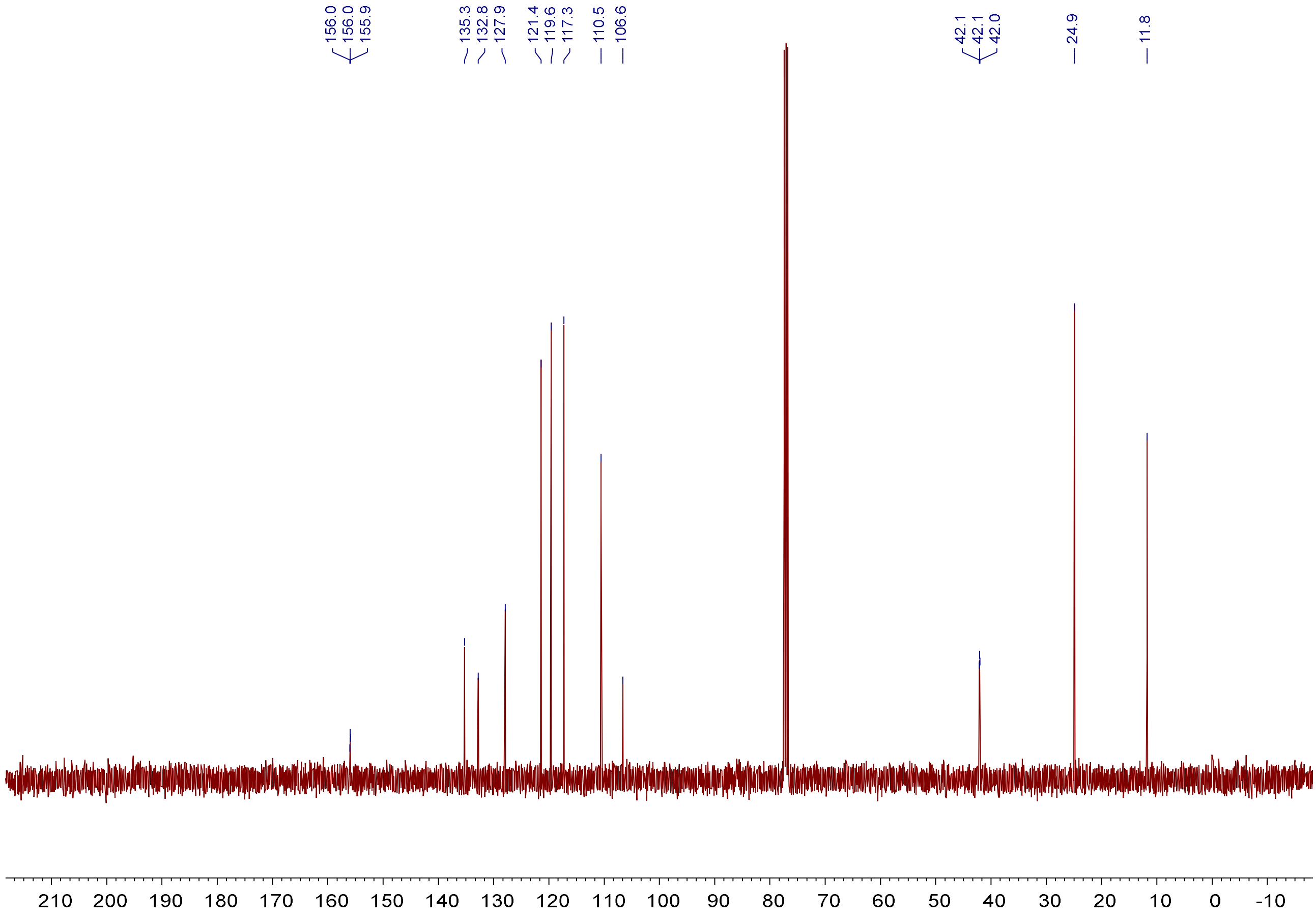

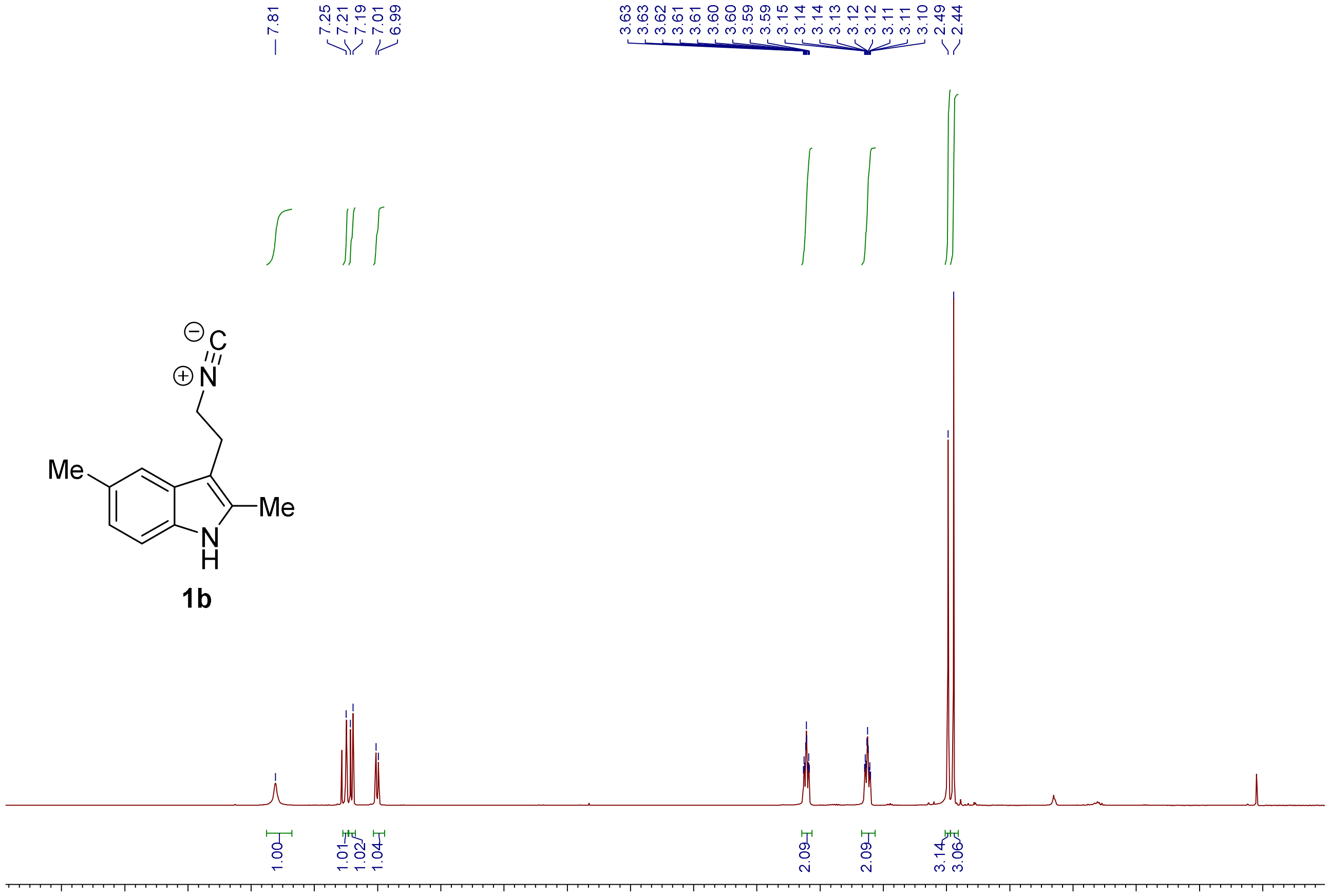
7.81
7.25
7.21
7.19
7.01
6.99
3.63
3.63
3.62
3.61
3.61
3.60
3.60
3.59
3.59
3.15
3.14
3.14
3.13
3.12
3.12
3.11
3.11
3.10
2.49
2.44
1.00
1.01
1.02
1.04
2.09
2.09
3.14
3.06
9.5 9.0 8.5 8.0 7.5 7.0 6.5 6.0 5.5 5.0 4.5 4.0 3.5 3.0 2.5 2.0 1.5 1.0 0.5 0.0
Me
Me
N
H
N
C

1b

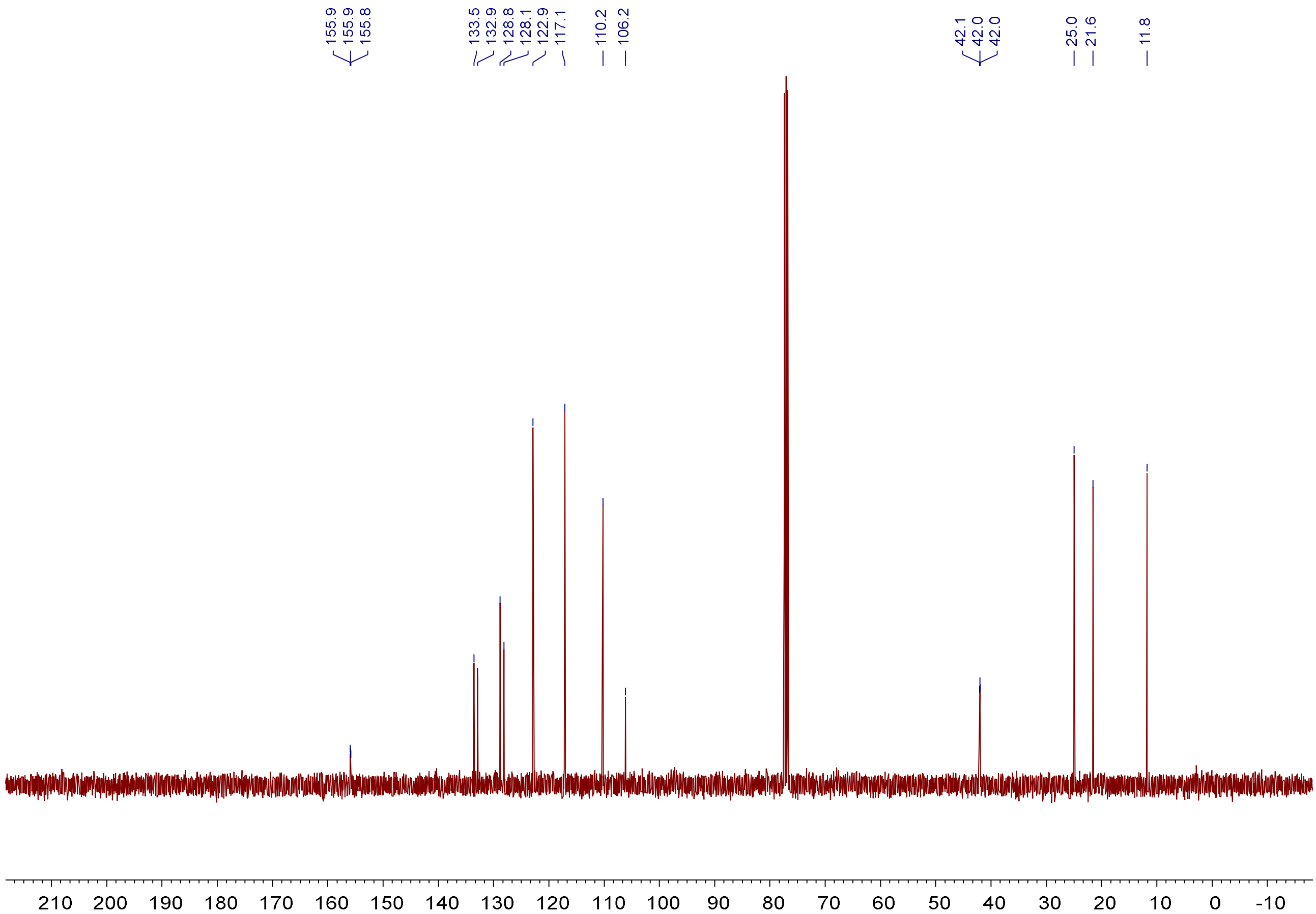
155.9
155.9
155.8
133.5
132.9
128.8
128.1
122.9
117.1
110.2
106.2
42.1
42.0
42.0
25.0
21.6
11.8
210 200 190 180 170 160 150 140 130 120 110 100 90 80 70 60 50 40 30 20 10 0 -10

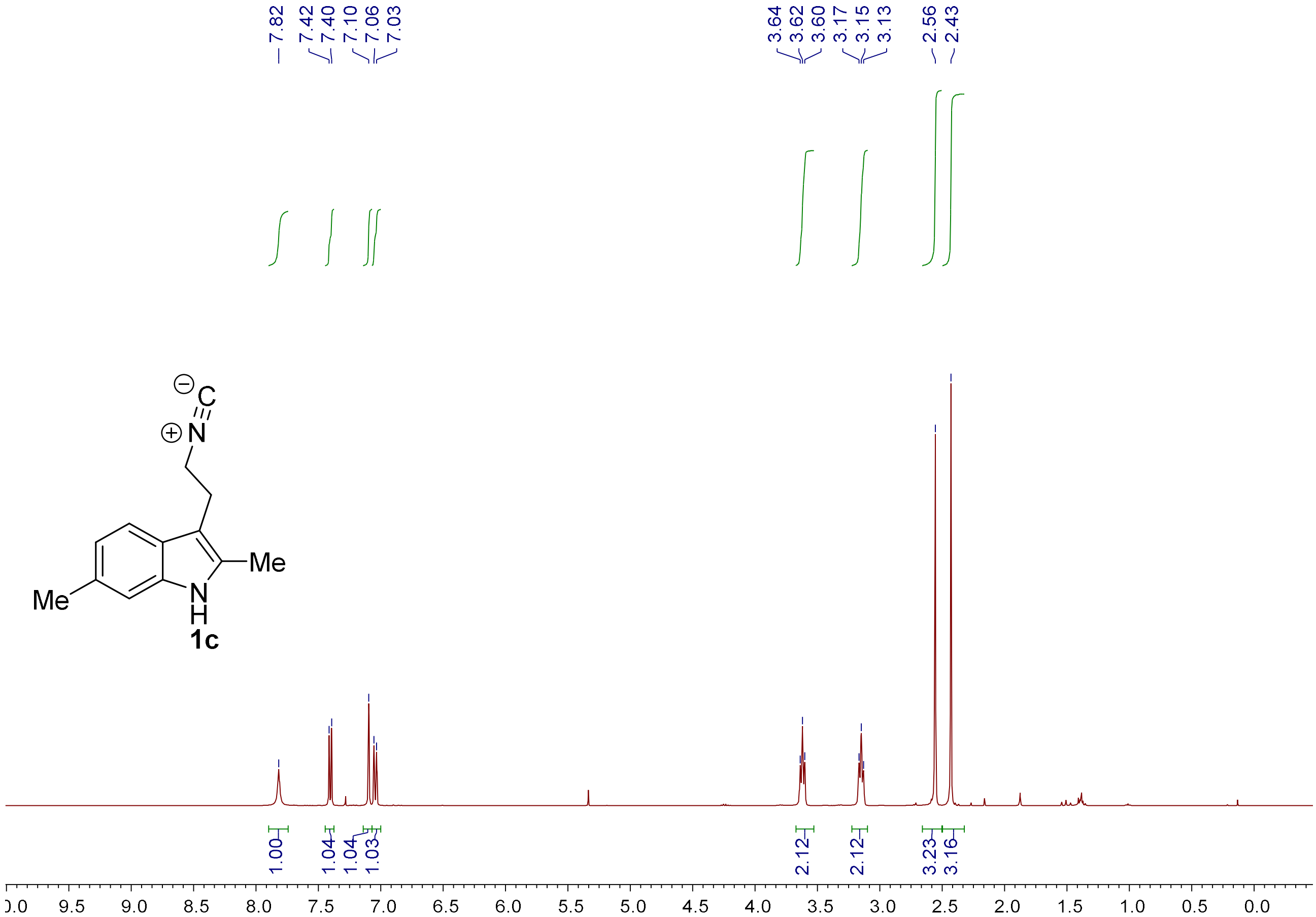
7.82
7.42
7.40
7.10
7.06
7.03
3.64
3.62
3.60
3.17
3.15
3.13
2.56
2.43
C
N
Me
Me
N
H
1c
1.00
1.04
1.04
1.03
2.12
2.12
3.23
3.16
0.0
9.5
9.0
8.5
8.0
7.5
7.0
6.5
6.0
5.5
5.0
4.5
4.0
3.5
3.0
2.5
2.0
1.5
1.0
0.5
0.0

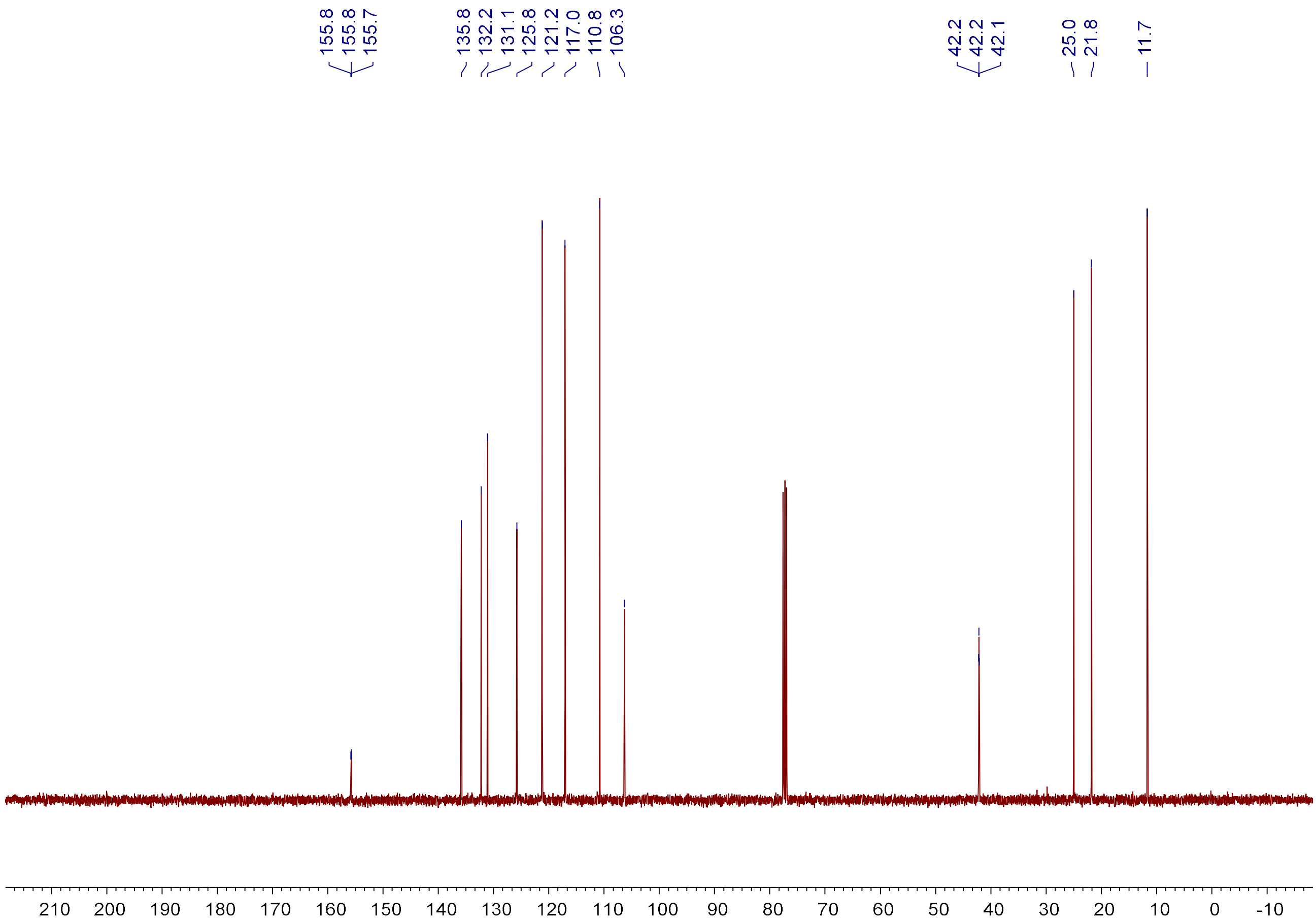
155.8
155.8
155.7
135.8
132.2
131.1
125.8
121.2
117.0
110.8
106.3
42.2
42.2
42.1
25.0
21.8
11.7
210
200
190
180
170
160
150
140
130
120
110
100
90
80
70
60
50
40
30
20
10
0
-10

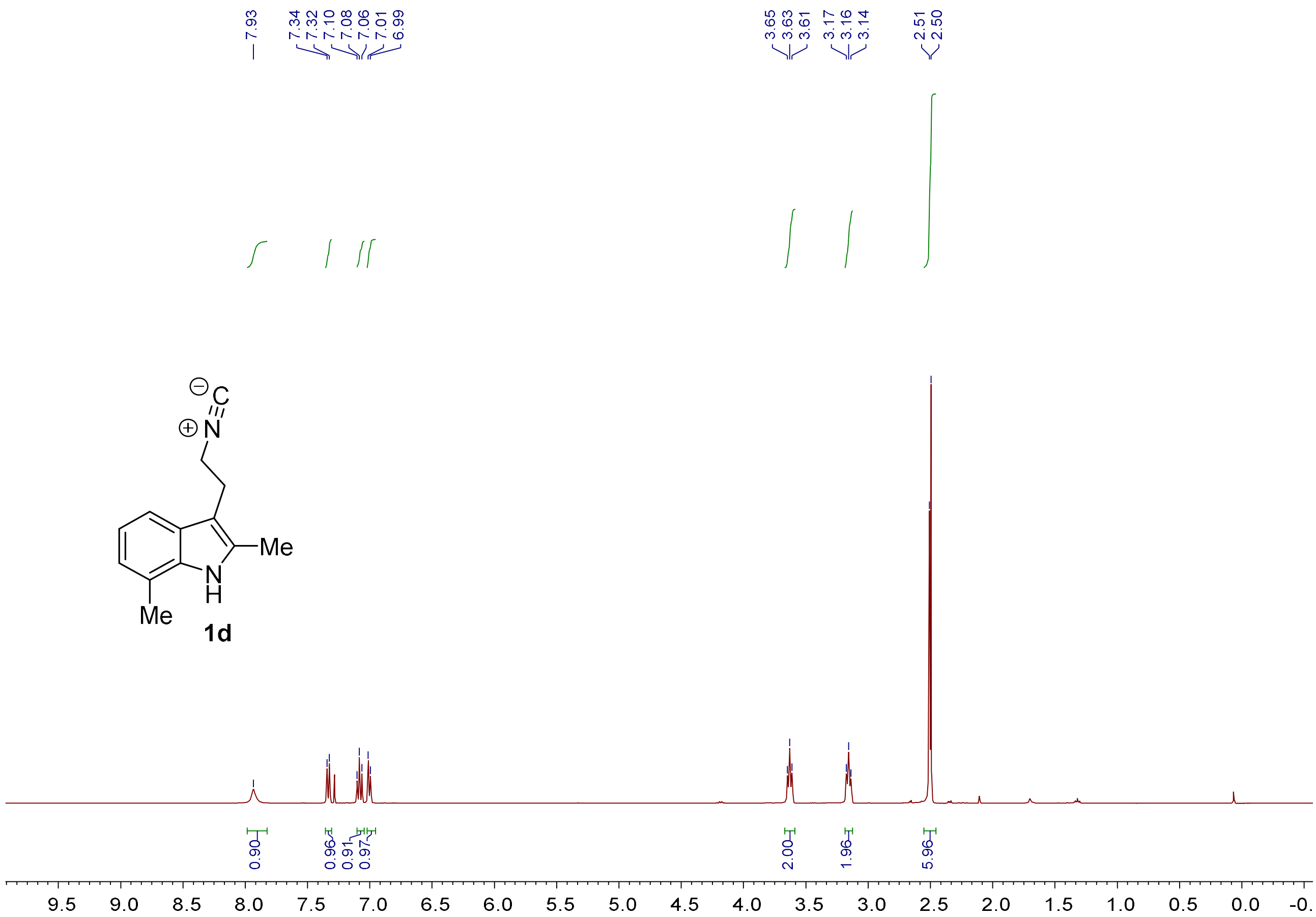
7.93
7.34
7.32
7.10
7.08
7.06
7.01
6.99
3.65
3.63
3.61
3.17
3.16
3.14
2.51
2.50
Me
Me
N
H
1d
0.90
0.96
0.91
0.97
2.00
1.96
5.96
9.5 9.0 8.5 8.0 7.5 7.0 6.5 6.0 5.5 5.0 4.5 4.0 3.5 3.0 2.5 2.0 1.5 1.0 0.5 0.0 -0.

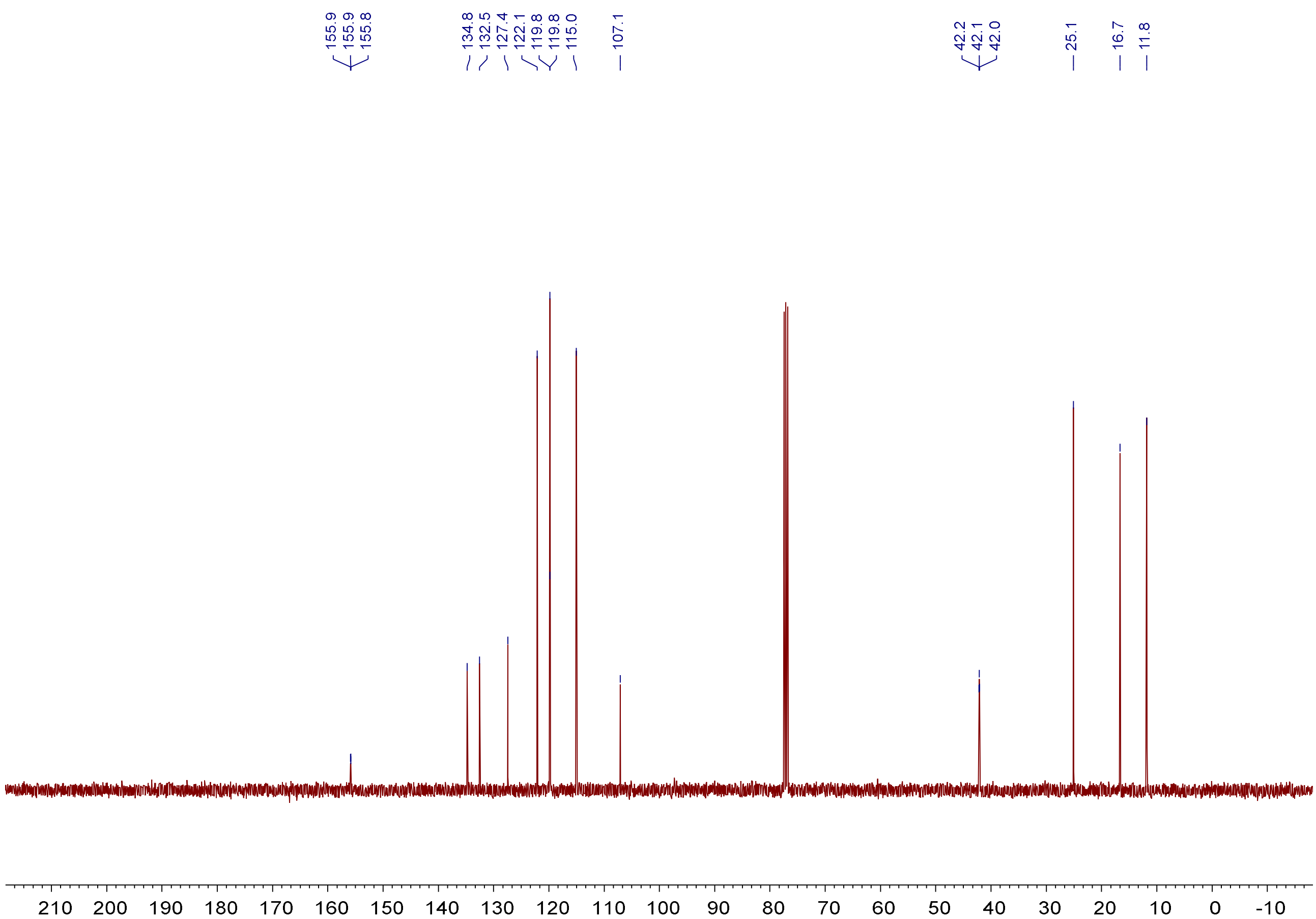
155.9
155.9
155.8
134.8
132.5
127.4
122.1
119.8
119.8
115.0
107.1
42.2
42.1
42.0
25.1
16.7
11.8
210 200 190 180 170 160 150 140 130 120 110 100 90 80 70 60 50 40 30 20 10 0 -10

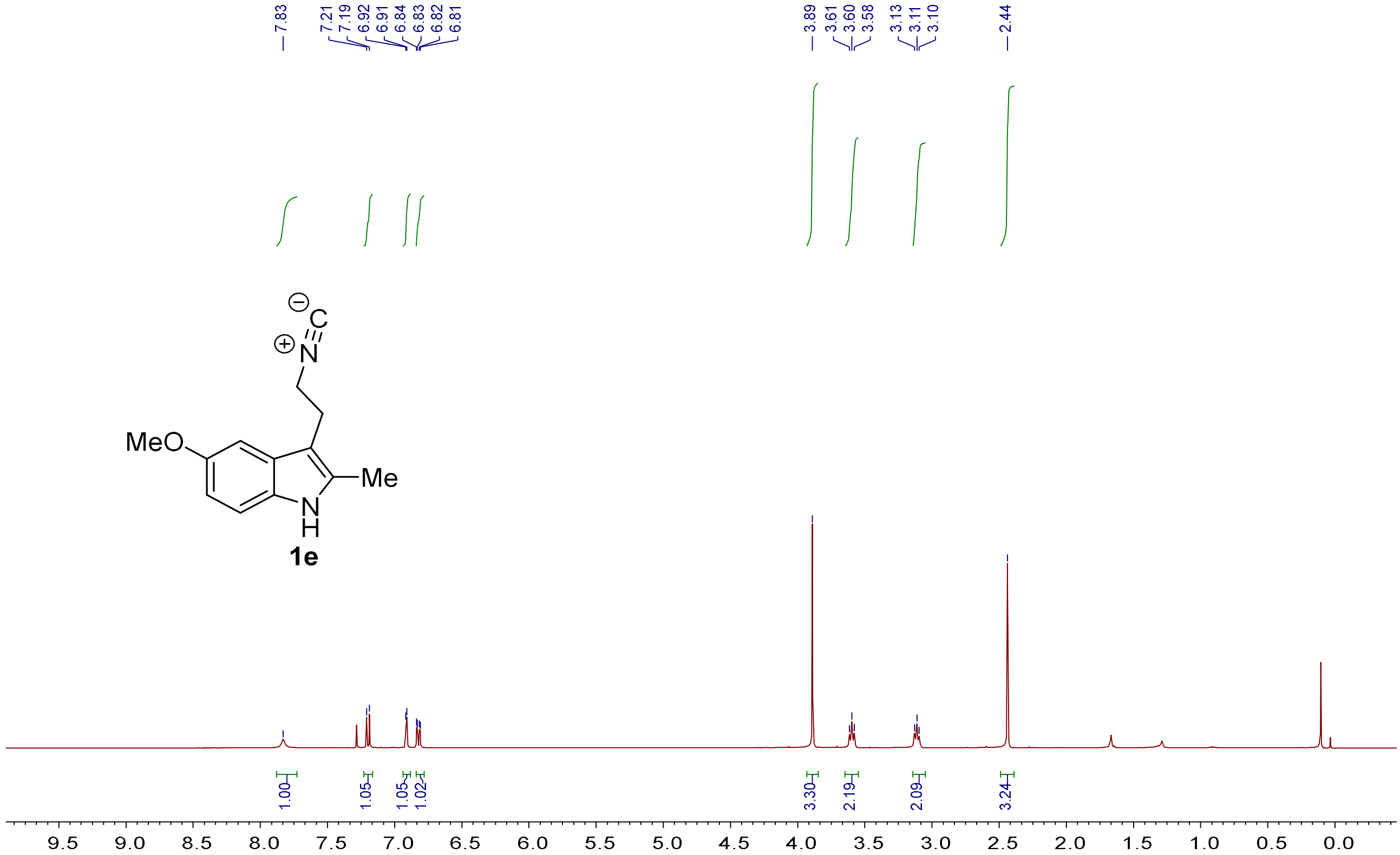
7.83
7.21
7.19
6.92
6.91
6.84
6.83
6.82
6.81
3.89
3.61
3.60
3.58
3.13
3.11
3.10
2.44
MeO
Me
N
H
1e
1.00
1.05
1.05
1.02
3.30
2.19
2.09
3.24
9.5 9.0 8.5 8.0 7.5 7.0 6.5 6.0 5.5 5.0 4.5 4.0 3.5 3.0 2.5 2.0 1.5 1.0 0.5 0.0

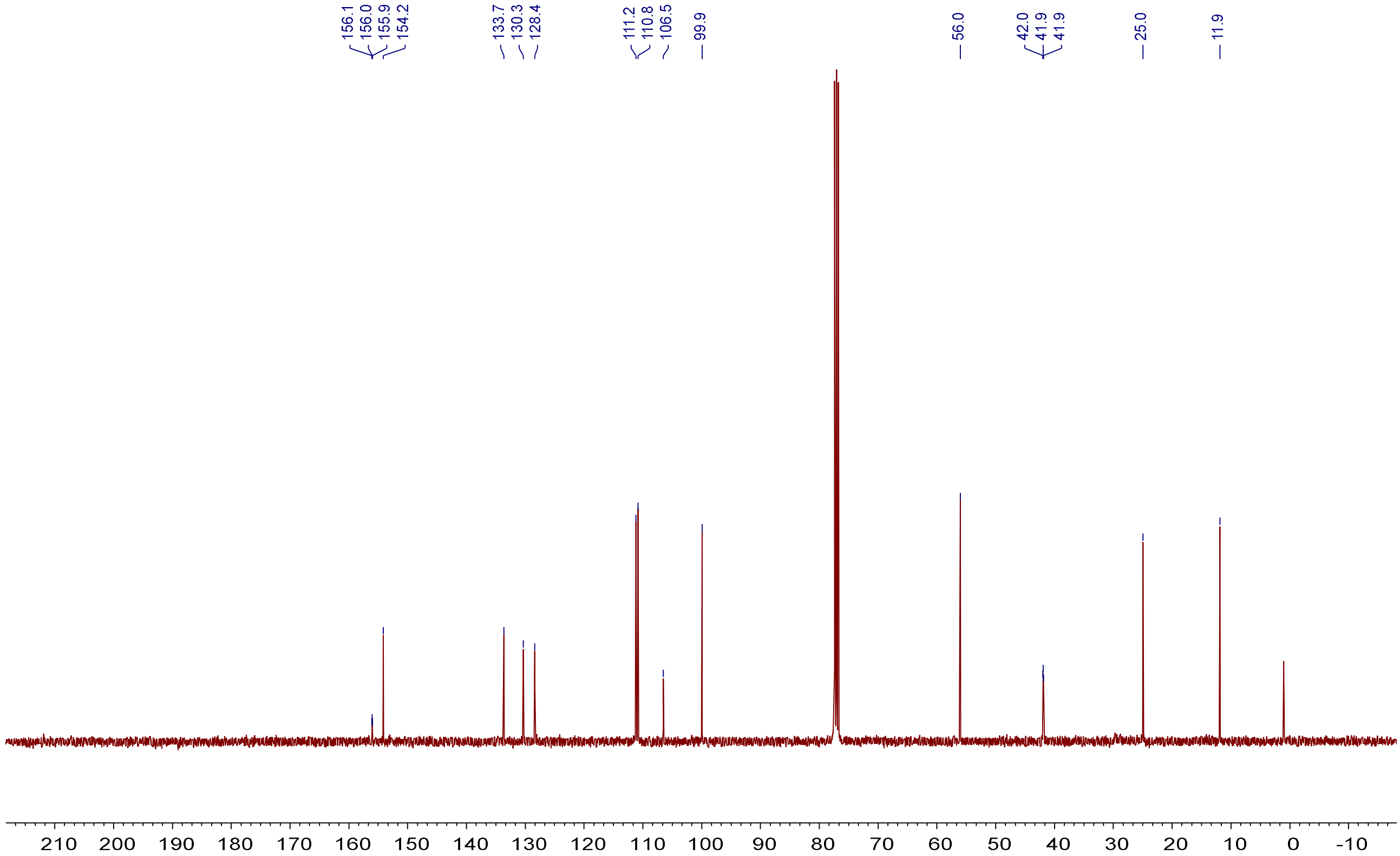
156.1
156.0
155.9
154.2
133.7
130.3
128.4
111.2
110.8
106.5
99.9
56.0
42.0
41.9
41.9
25.0
11.9
210 200 190 180 170 160 150 140 130 120 110 100 90 80 70 60 50 40 30 20 10 0 -10

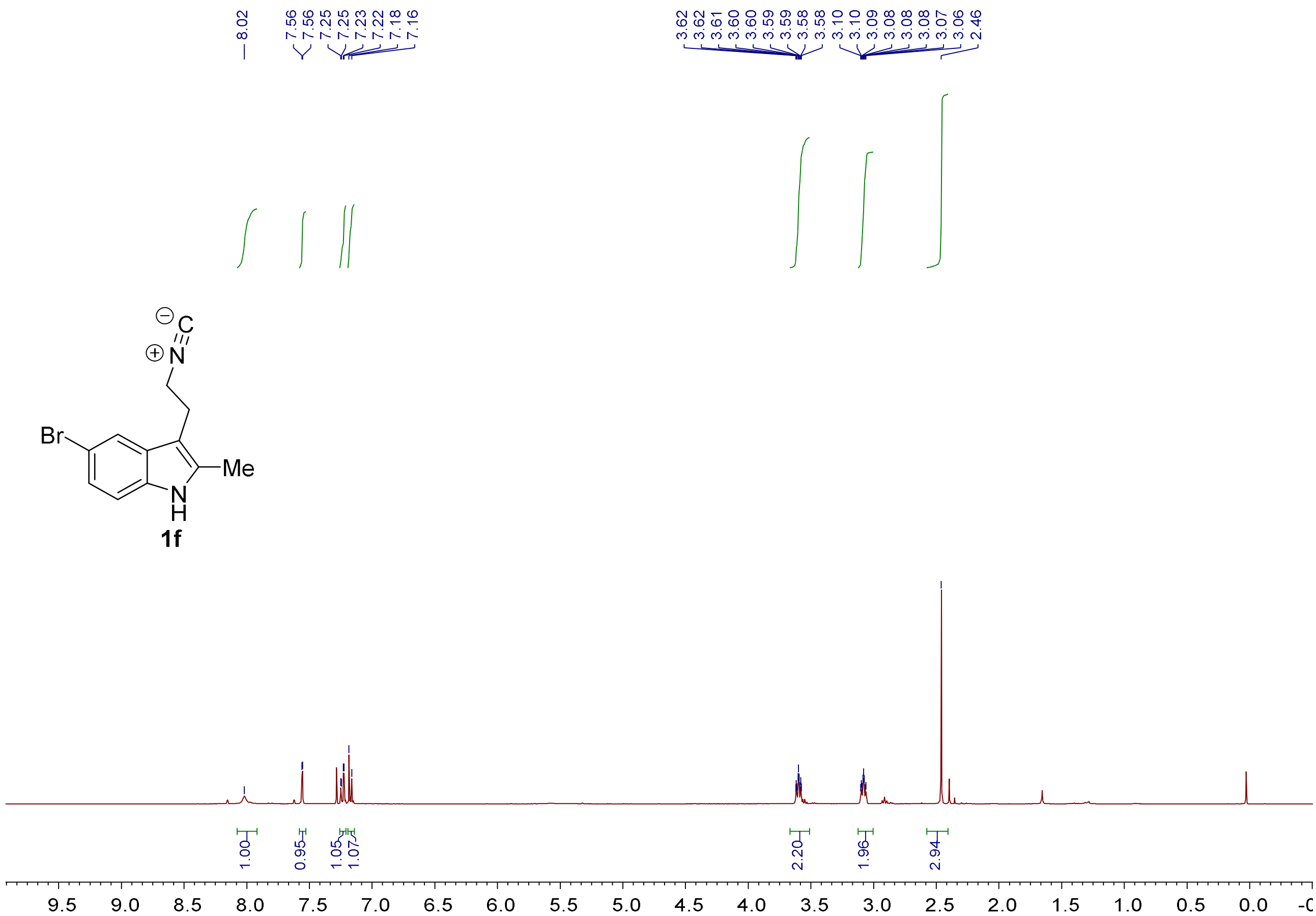

Br
N
H
Me
1f

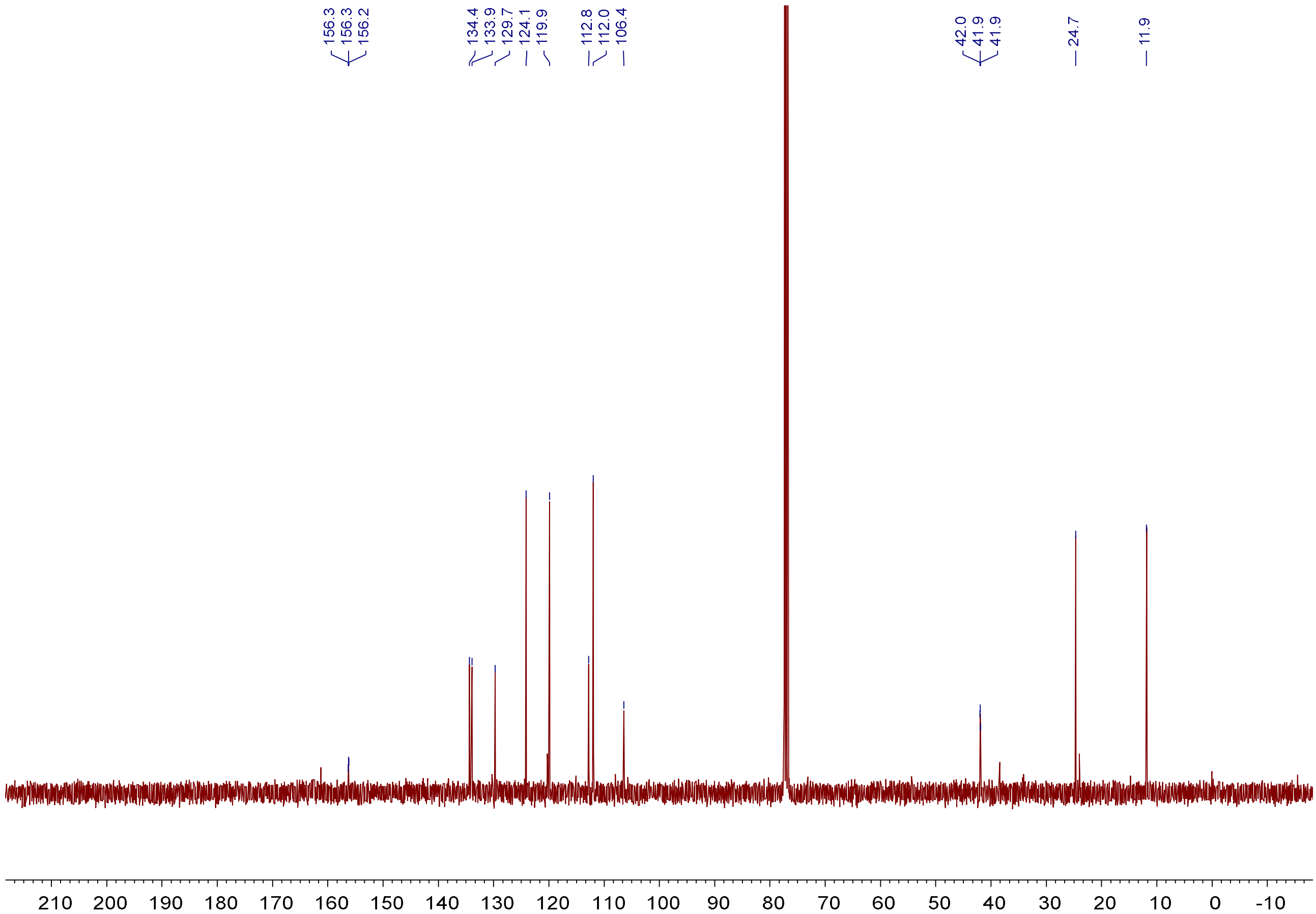

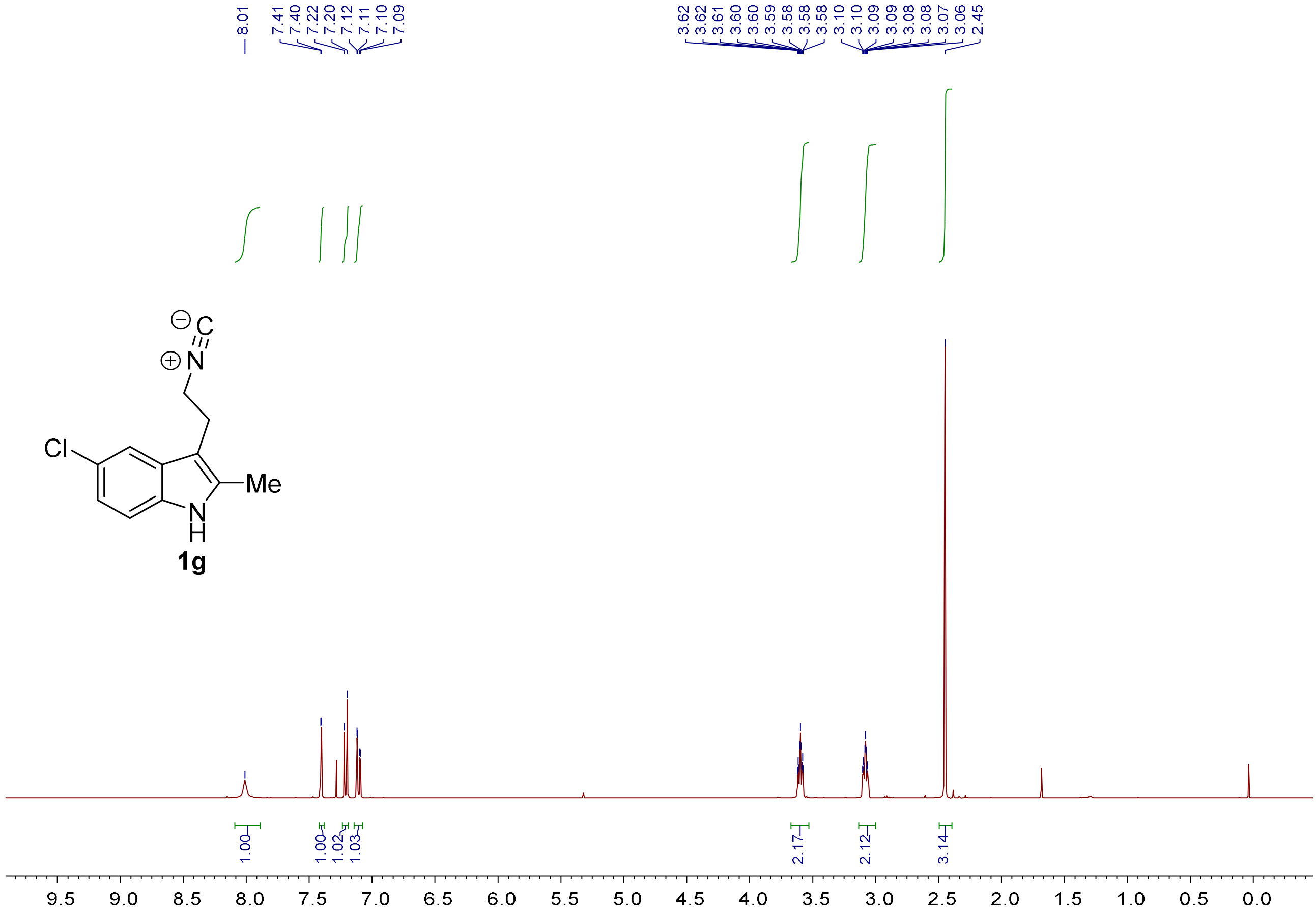
8.01
7.41 7.40 7.22 7.20 7.12 7.11 7.10 7.09
3.62 3.62 3.61 3.60 3.60 3.59 3.58 3.58 3.58 3.10 3.10 3.09 3.09 3.08 3.08 3.07 3.06 2.45
C
N
Cl
Me
N
H
1.00 1.00 1.02 1.03 2.17 2.12 3.14
9.5 9.0 8.5 8.0 7.5 7.0 6.5 6.0 5.5 5.0 4.5 4.0 3.5 3.0 2.5 2.0 1.5 1.0 0.5 0.0

1g

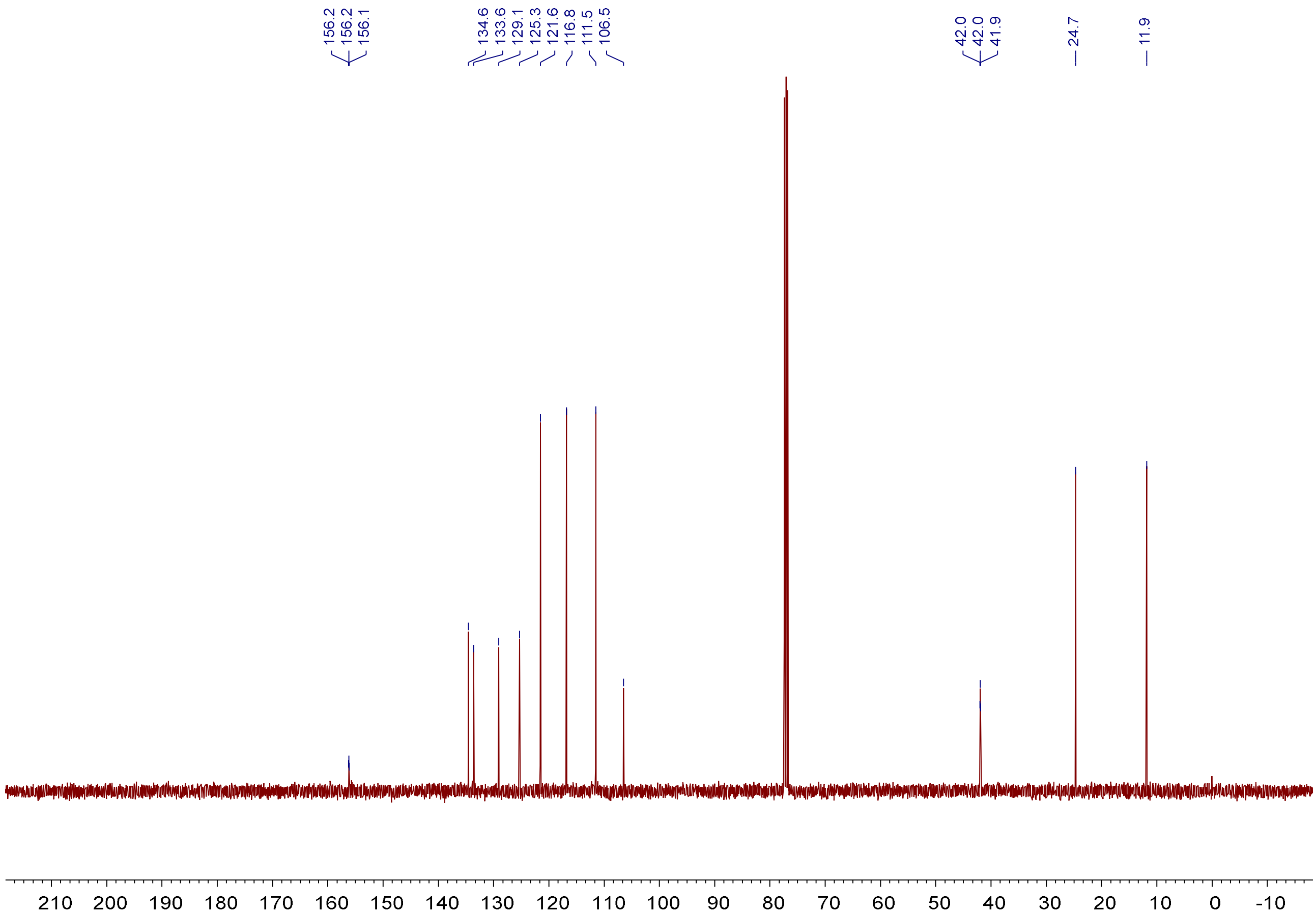
156.2 156.2 156.1
134.6 133.6 129.1 125.3 121.6 116.8 111.5 106.5
42.0 42.0 41.9
24.7
11.9
210 200 190 180 170 160 150 140 130 120 110 100 90 80 70 60 50 40 30 20 10 0 -10

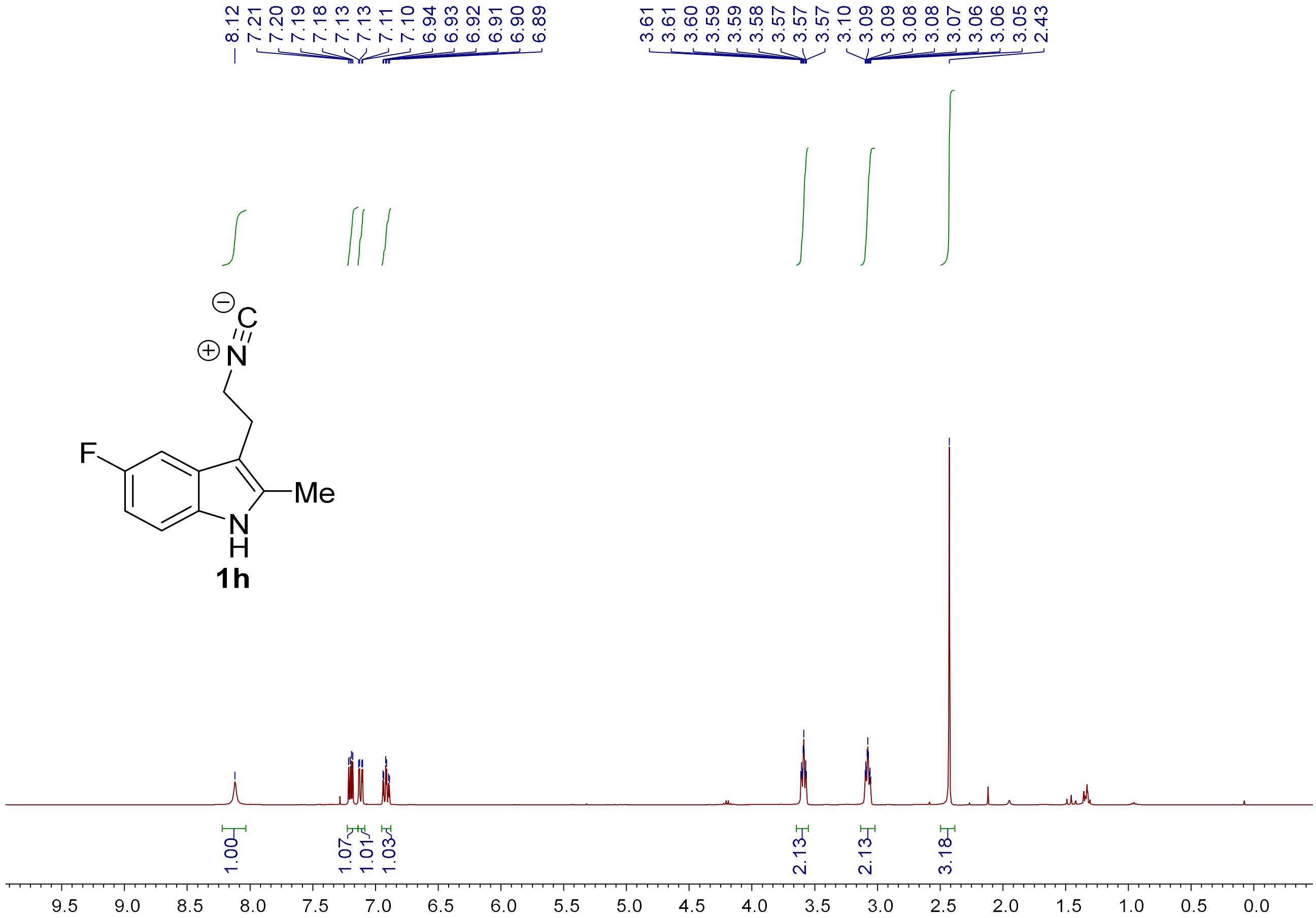

1h
8.12
7.21
7.20
7.19
7.18
7.13
7.13
7.11
7.10
6.94
6.93
6.92
6.91
6.90
6.89
3.61
3.61
3.60
3.59
3.59
3.58
3.57
3.57
3.57
3.10
3.09
3.09
3.08
3.08
3.07
3.06
3.06
3.05
2.43
1.00
1.07
1.01
1.03
2.13
2.13
3.18
9.5 9.0 8.5 8.0 7.5 7.0 6.5 6.0 5.5 5.0 4.5 4.0 3.5 3.0 2.5 2.0 1.5 1.0 0.5 0.0

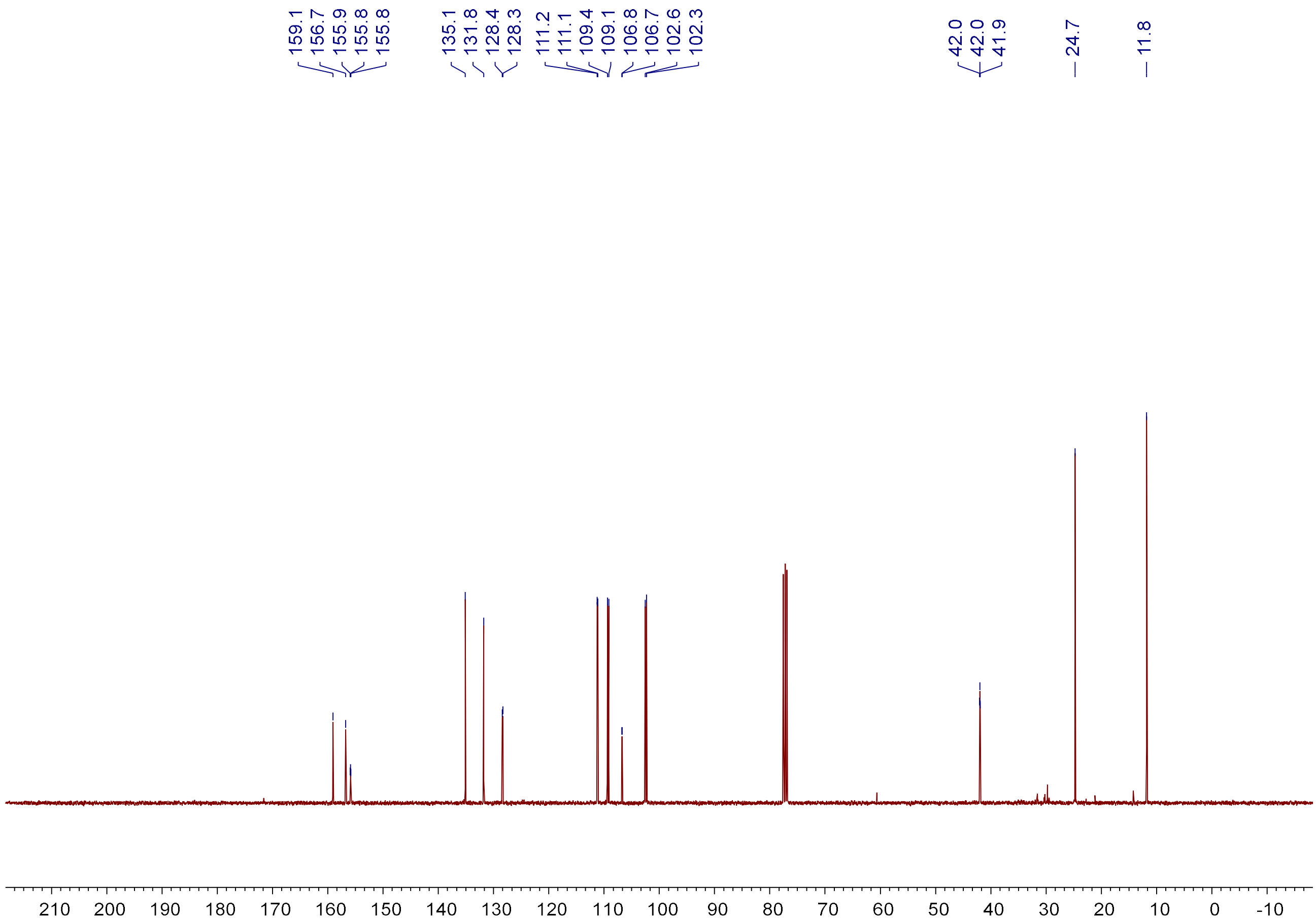
159.1
156.7
155.9
155.8
155.8
135.1
131.8
128.4
128.3
111.2
111.1
109.4
109.1
106.8
106.7
102.6
102.3
42.0
42.0
41.9
24.7
11.8
210 200 190 180 170 160 150 140 130 120 110 100 90 80 70 60 50 40 30 20 10 0 -10

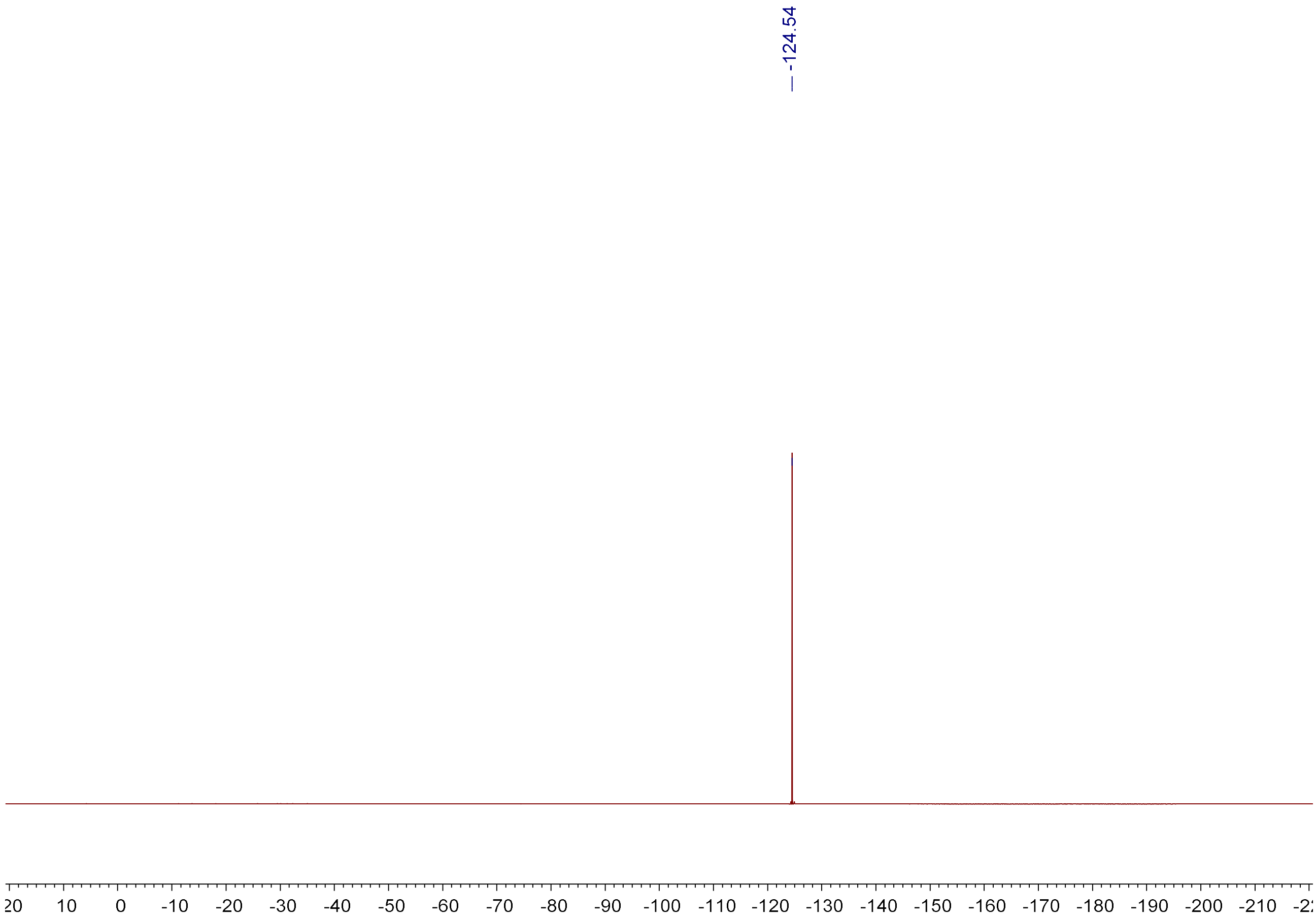
-124.54
20 10 0 -10 -20 -30 -40 -50 -60 -70 -80 -90 -100 -110 -120 -130 -140 -150 -160 -170 -180 -190 -200 -210

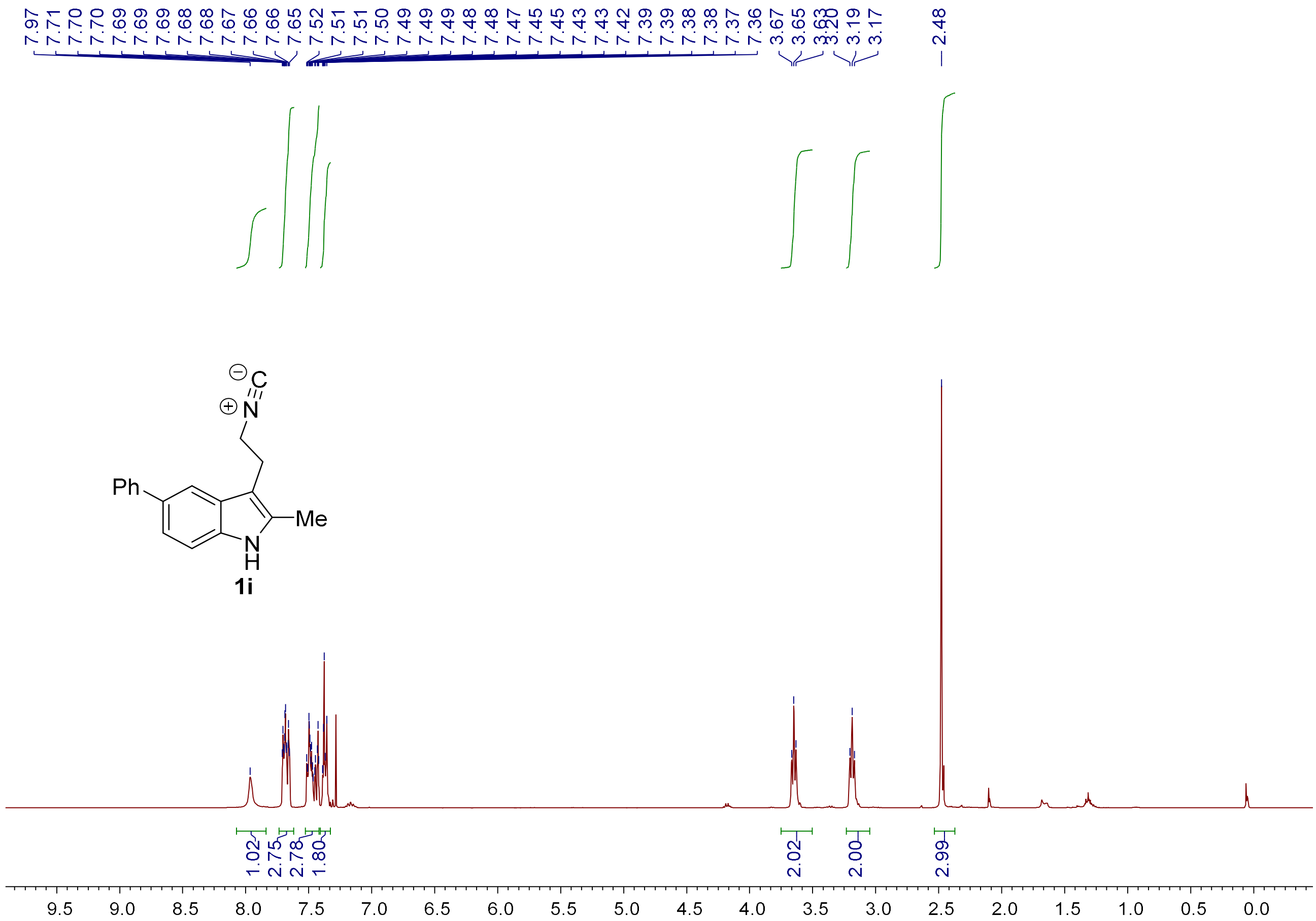
7.97 7.71 7.70 7.70 7.69 7.69 7.69 7.68 7.68 7.67 7.66 7.66 7.65 7.52 7.51 7.51 7.50 7.49 7.49 7.49 7.48 7.48 7.47 7.45 7.45 7.43 7.43 7.42 7.39 7.39 7.38 7.38 7.37 7.36 3.67 3.65 3.63 3.20 3.19 3.17 2.48
1.02 2.75 2.78 1.80 2.02 2.00 2.99
9.5 9.0 8.5 8.0 7.5 7.0 6.5 6.0 5.5 5.0 4.5 4.0 3.5 3.0 2.5 2.0 1.5 1.0 0.5 0.0
C
N
Ph
Me
N
H

1i

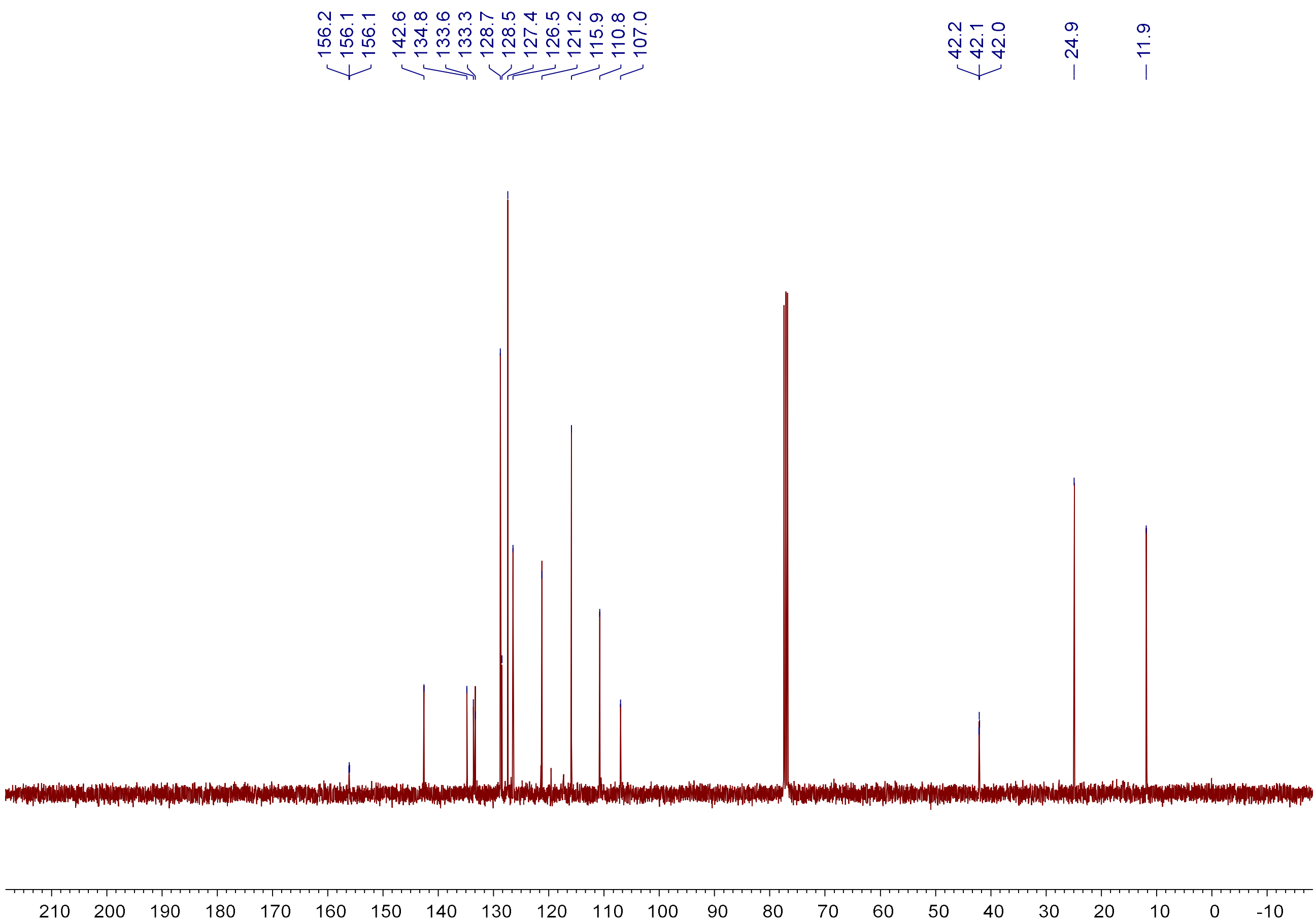
156.2 156.1 156.1 142.6 134.8 133.6 133.3 128.7 128.5 127.4 126.5 121.2 115.9 110.8 107.0 42.2 42.1 42.0 24.9 11.9
210 200 190 180 170 160 150 140 130 120 110 100 90 80 70 60 50 40 30 20 10 0 -10

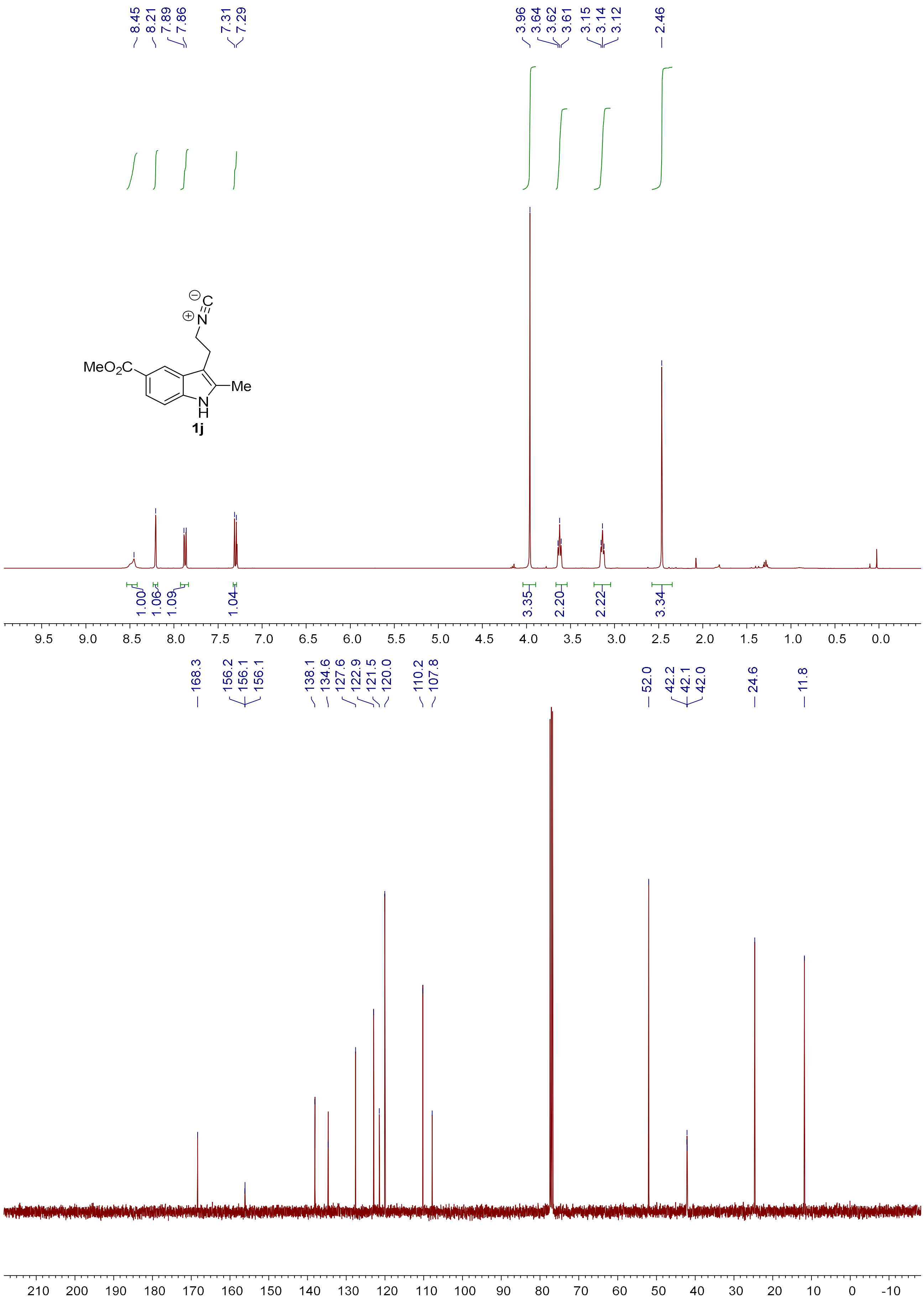
8.45
8.21
7.89
7.86
7.31
7.29
3.96
3.64
3.62
3.61
3.15
3.14
3.12
2.46
MeO2C
Me
N
H
1j
1.00
1.06
1.09
1.04
3.35
2.20
2.22
3.34
168.3
156.2
156.1
156.1
138.1
134.6
127.6
122.9
121.5
120.0
110.2
107.8
52.0
42.2
42.1
42.0
24.6
11.8

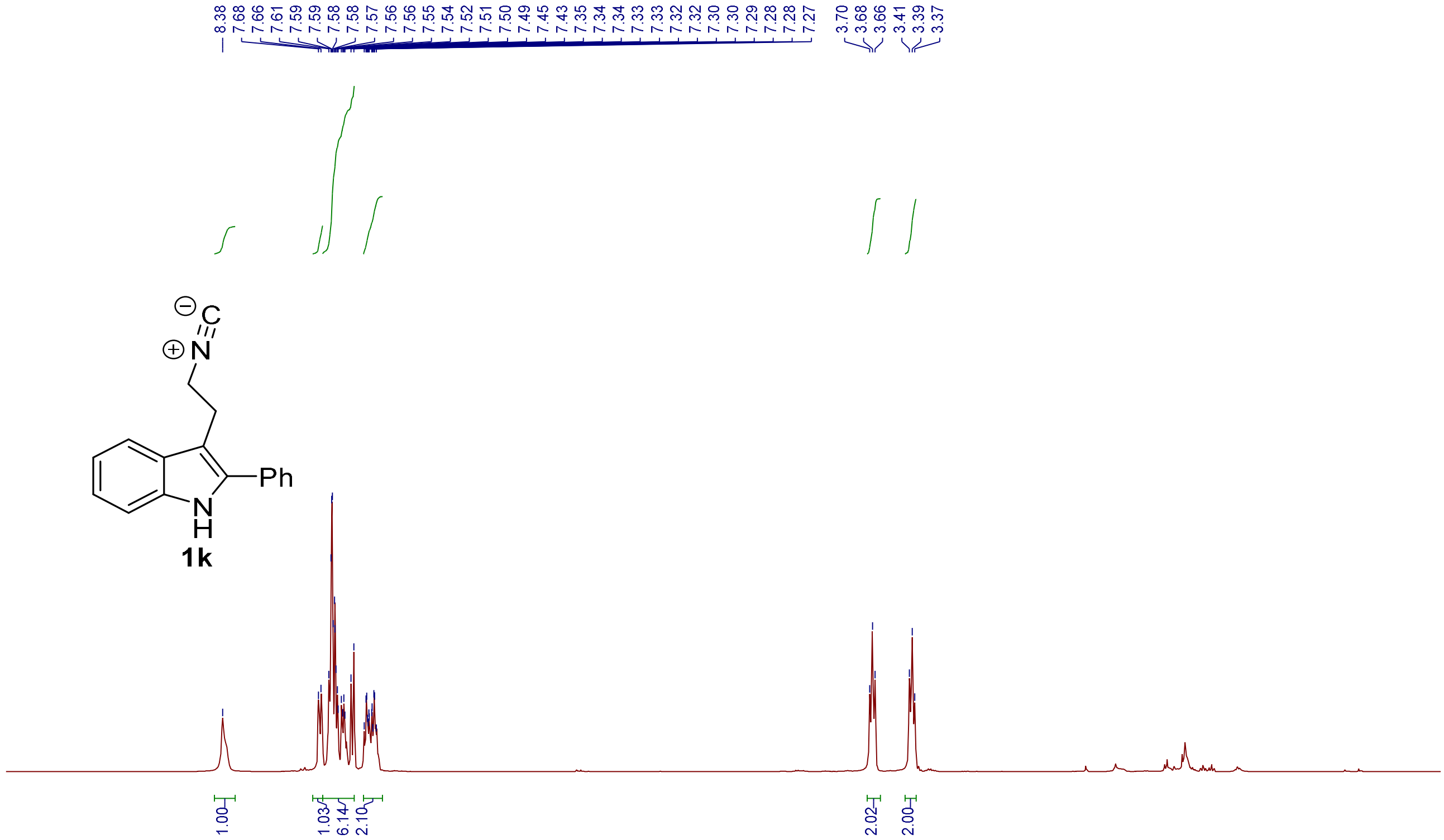
8.38
7.68 7.66 7.61 7.59 7.59 7.58 7.58 7.57 7.56 7.56 7.55 7.54 7.52 7.51 7.50 7.49 7.45 7.43 7.35 7.34 7.34 7.33 7.33 7.32 7.32 7.30 7.30 7.29 7.28 7.28 7.27
3.70 3.68 3.66 3.41 3.39 3.37
N
C
Ph
N
H
1k
1.00
1.03
6.14
2.10
2.02
2.00
9.5 9.0 8.5 8.0 7.5 7.0 6.5 6.0 5.5 5.0 4.5 4.0 3.5 3.0 2.5 2.0 1.5 1.0 0.5 0.0

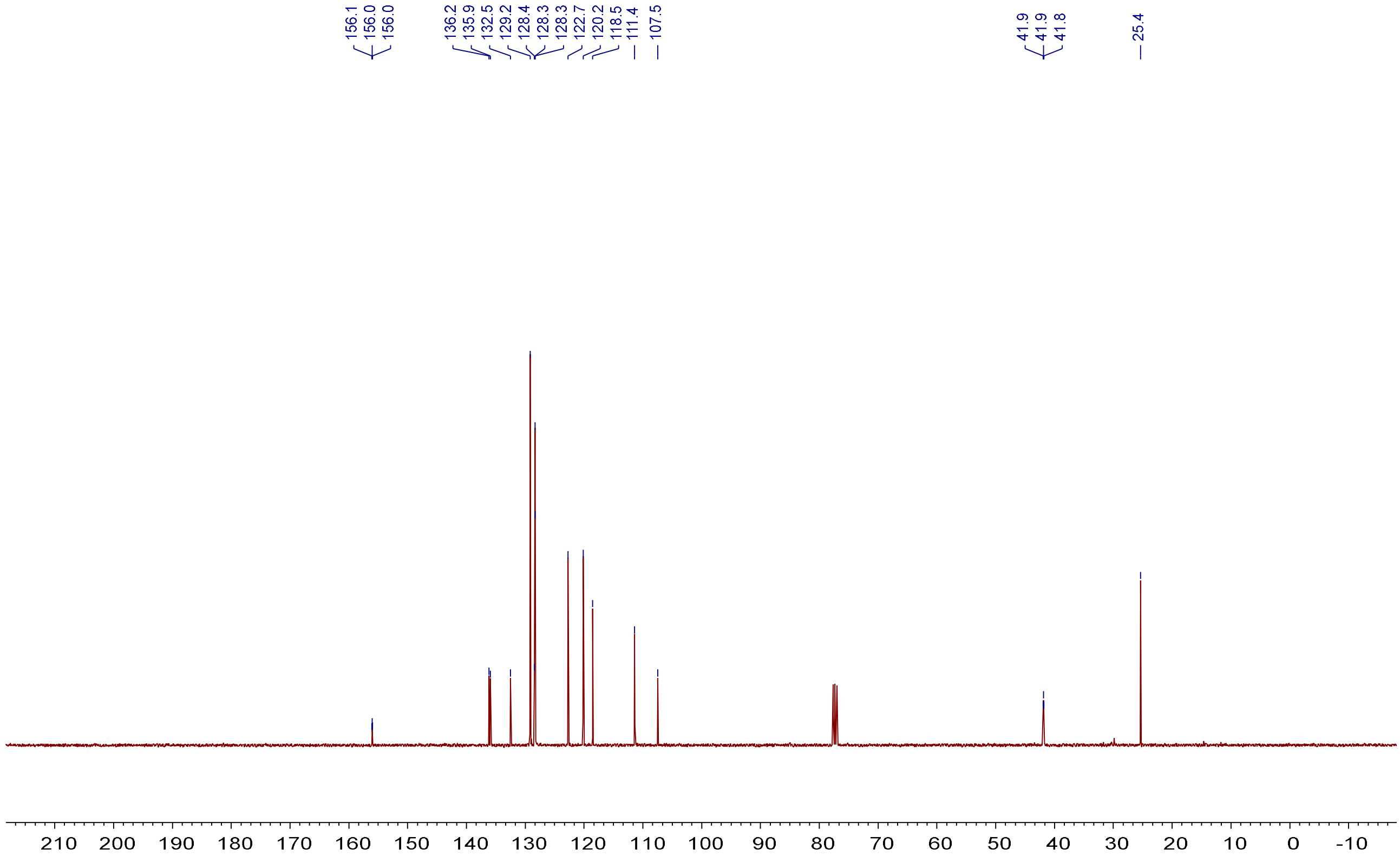
156.1 156.0 156.0
136.2 135.9 132.5 129.2 128.4 128.3 128.3 122.7 120.2 118.5 111.4 107.5
41.9 41.9 41.8
25.4
210 200 190 180 170 160 150 140 130 120 110 100 90 80 70 60 50 40 30 20 10 0 -10

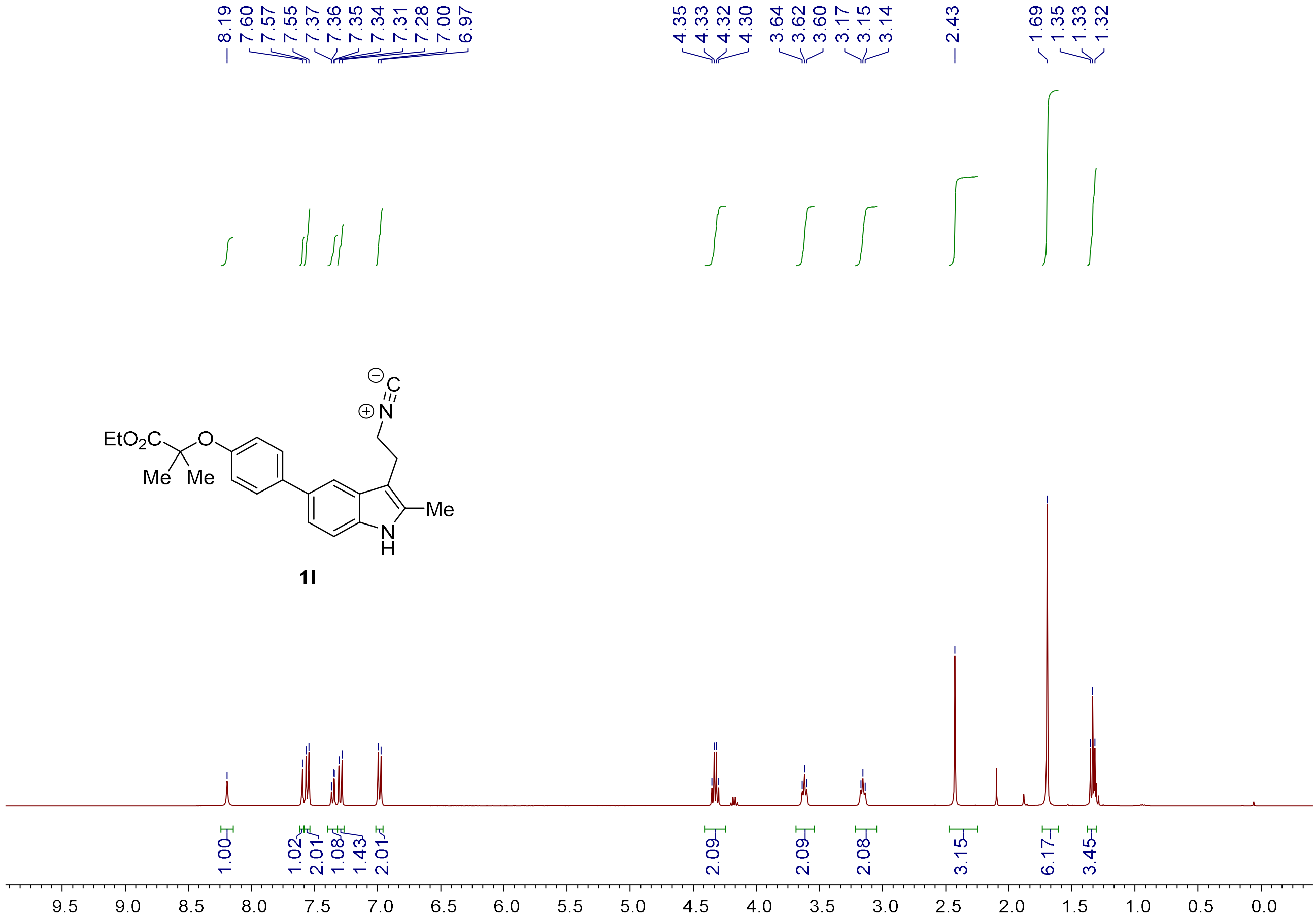
8.19 7.60 7.57 7.55 7.37 7.36 7.35 7.34 7.31 7.28 7.00 6.97
4.35 4.33 4.32 4.30 3.64 3.62 3.60 3.17 3.15 3.14
2.43
1.69 1.35 1.33 1.32
EtO2C
O
Me Me
N
C
Me
N
H
1l
1.00 1.02 2.01 1.08 1.43 2.01 2.09 2.09 2.08 3.15 6.17 3.45
9.5 9.0 8.5 8.0 7.5 7.0 6.5 6.0 5.5 5.0 4.5 4.0 3.5 3.0 2.5 2.0 1.5 1.0 0.5 0.0

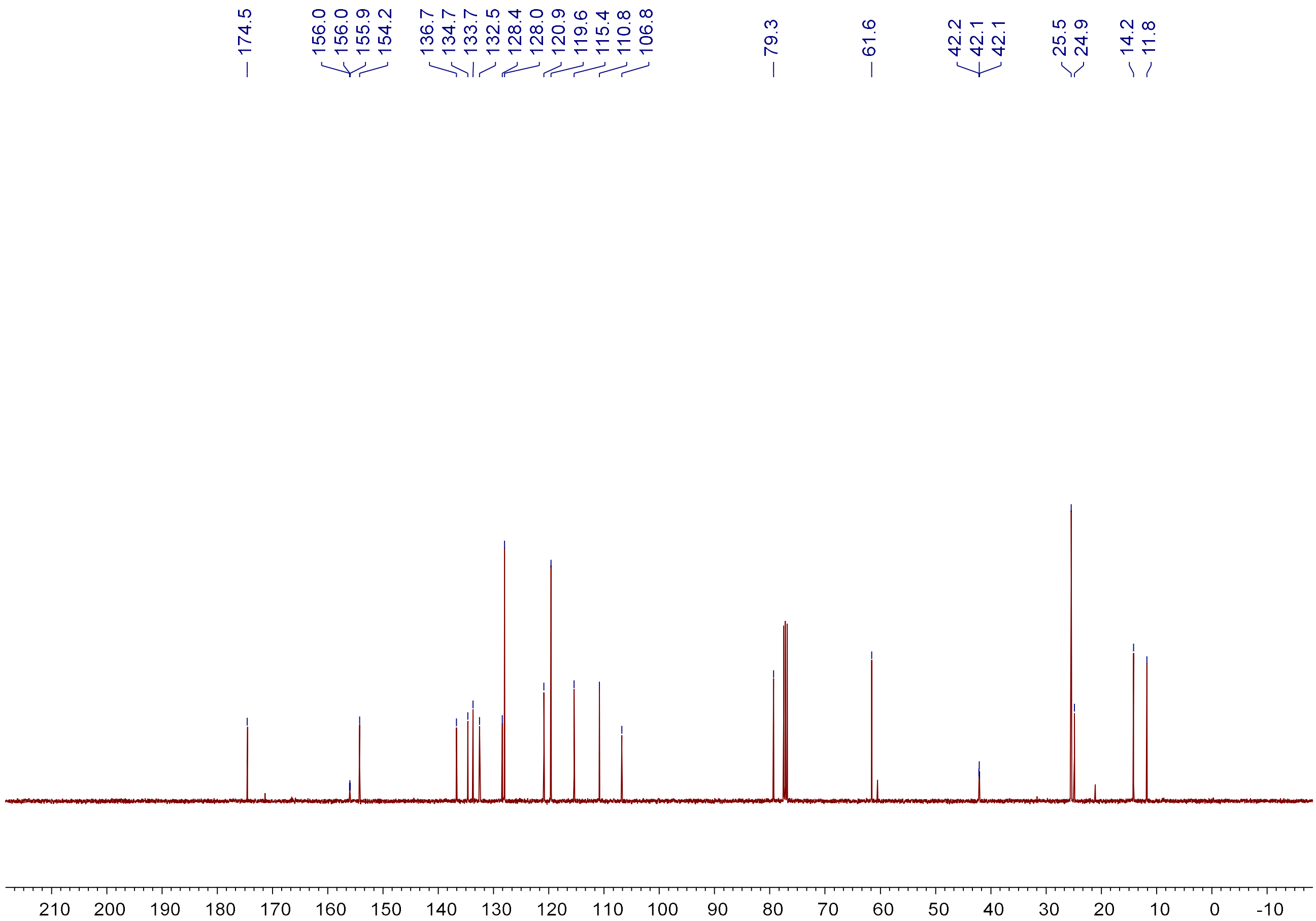
174.5
156.0 156.0 155.9 154.2
136.7 134.7 133.7 132.5 128.4 128.0 120.9 119.6 115.4 110.8 106.8
79.3
61.6
42.2 42.1 42.1
25.5 24.9
14.2 11.8
210 200 190 180 170 160 150 140 130 120 110 100 90 80 70 60 50 40 30 20 10 0 -10

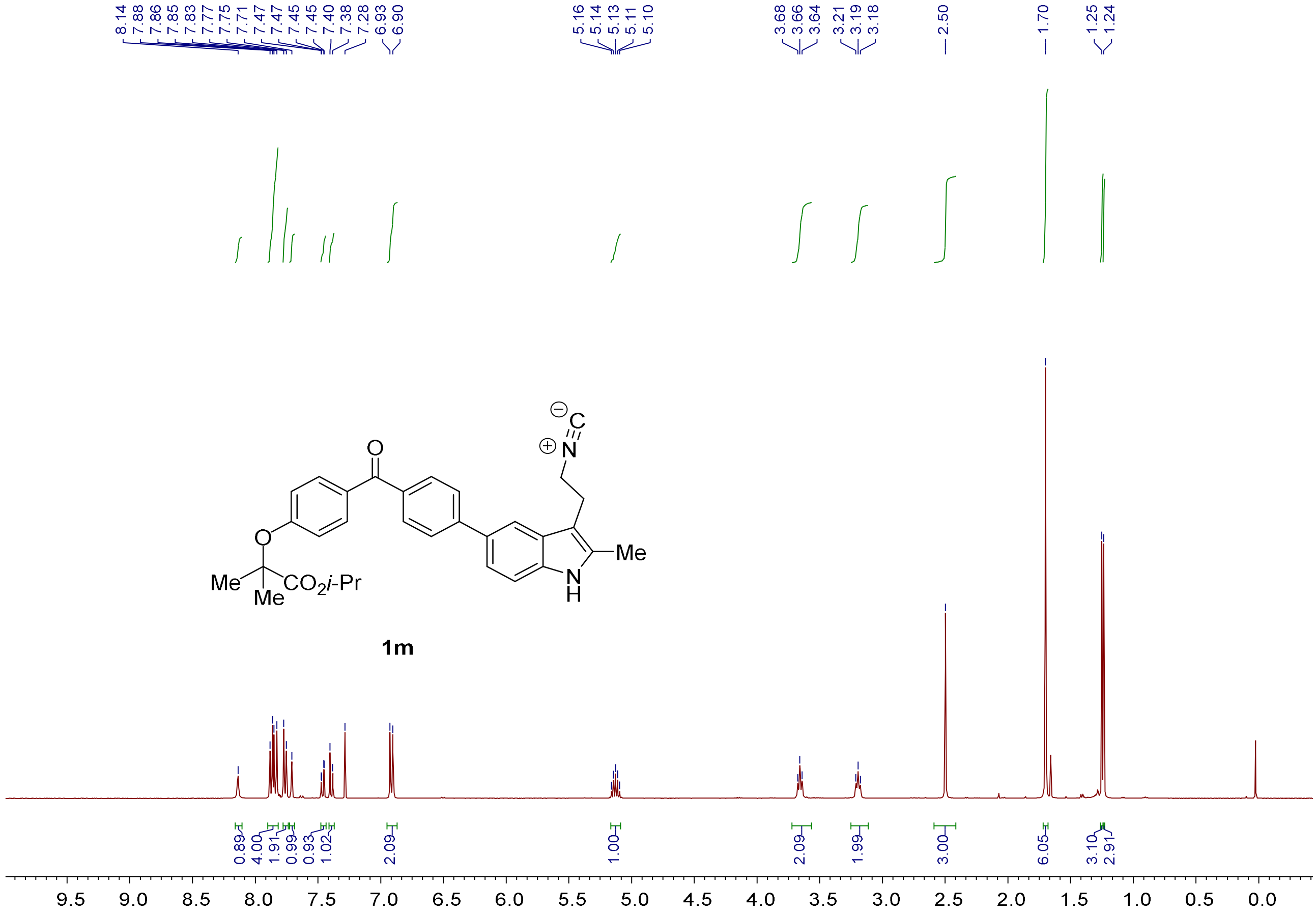
8.14 7.88 7.86 7.85 7.83 7.77 7.75 7.71 7.47 7.47 7.45 7.45 7.40 7.38 7.28 6.93 6.90
5.16 5.14 5.13 5.11 5.10
3.68 3.66 3.64 3.21 3.19 3.18
2.50
1.70
1.25 1.24
O
Me
CO2i-Pr
Me
N
C
H
1m
0.89 4.00 1.91 0.99 0.93 1.02 2.09 1.00 2.09 1.99 3.00 6.05 3.10 2.91
9.5 9.0 8.5 8.0 7.5 7.0 6.5 6.0 5.5 5.0 4.5 4.0 3.5 3.0 2.5 2.0 1.5 1.0 0.5 0.0

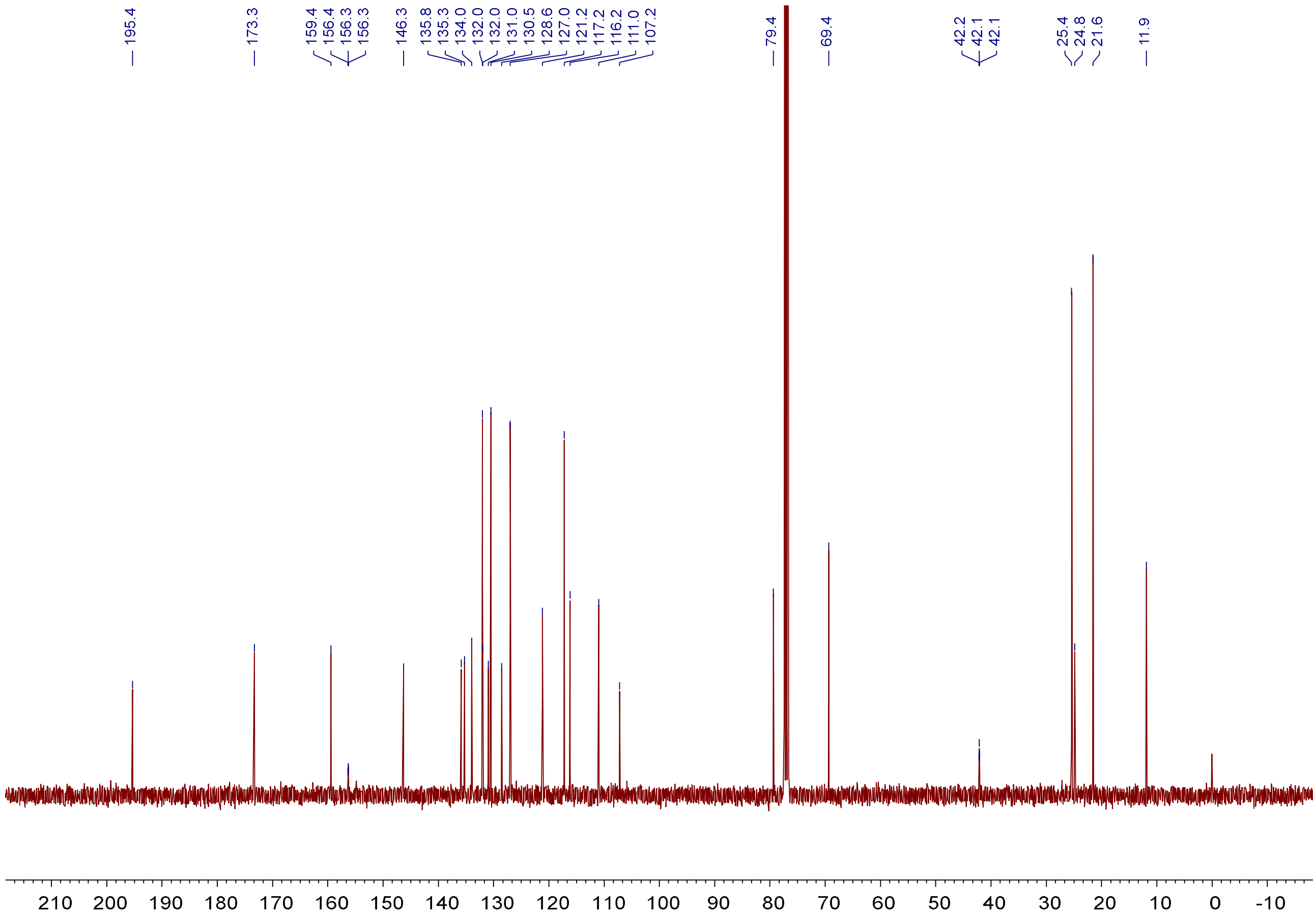
195.4
173.3
159.4 156.4 156.3 156.3
146.3
135.8 135.3 134.0 132.0 132.0 131.0 130.5 128.6 127.0 121.2 117.2 116.2 111.0 107.2
79.4
69.4
42.2 42.1 42.1
25.4 24.8 21.6
11.9
210 200 190 180 170 160 150 140 130 120 110 100 90 80 70 60 50 40 30 20 10 0 -10

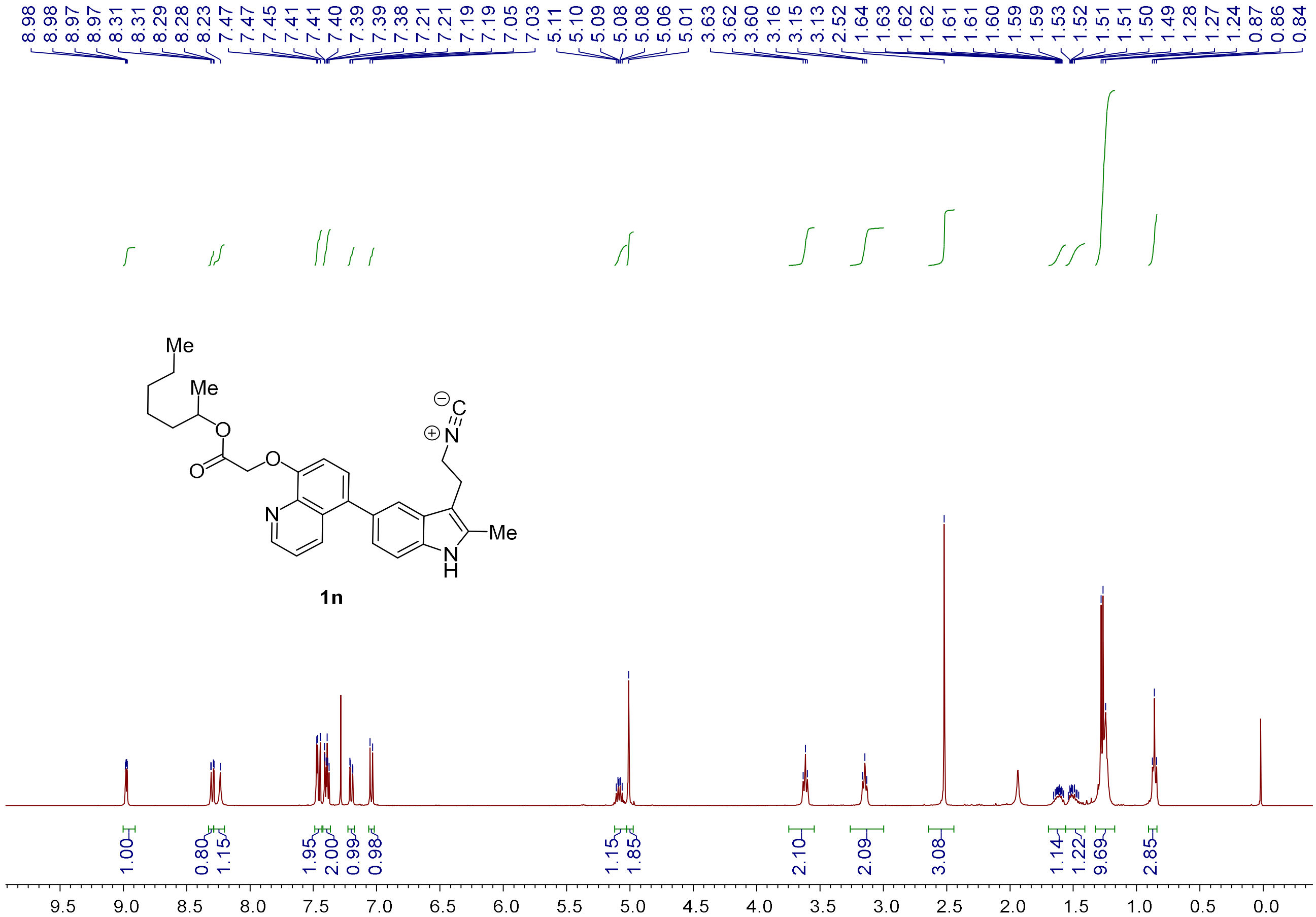
8.98 8.98 8.97 8.97 8.31 8.31 8.29 8.28 8.23 7.47 7.47 7.45 7.41 7.41 7.40 7.39 7.39 7.38 7.21 7.21 7.19 7.19 7.05 7.03 5.11 5.10 5.09 5.08 5.08 5.06 5.01 3.63 3.62 3.60 3.16 3.15 3.13 2.52 1.64 1.63 1.62 1.62 1.61 1.61 1.60 1.59 1.59 1.53 1.52 1.51 1.51 1.50 1.49 1.28 1.27 1.24 0.87 0.86 0.84
Me
Me
O
O
O
N
N
C
N
H
Me
1.00 0.80 1.15 1.95 2.00 0.99 0.98 1.15 1.85 2.10 2.09 3.08 1.14 1.22 9.69 2.85
9.5 9.0 8.5 8.0 7.5 7.0 6.5 6.0 5.5 5.0 4.5 4.0 3.5 3.0 2.5 2.0 1.5 1.0 0.5 0.0

1n

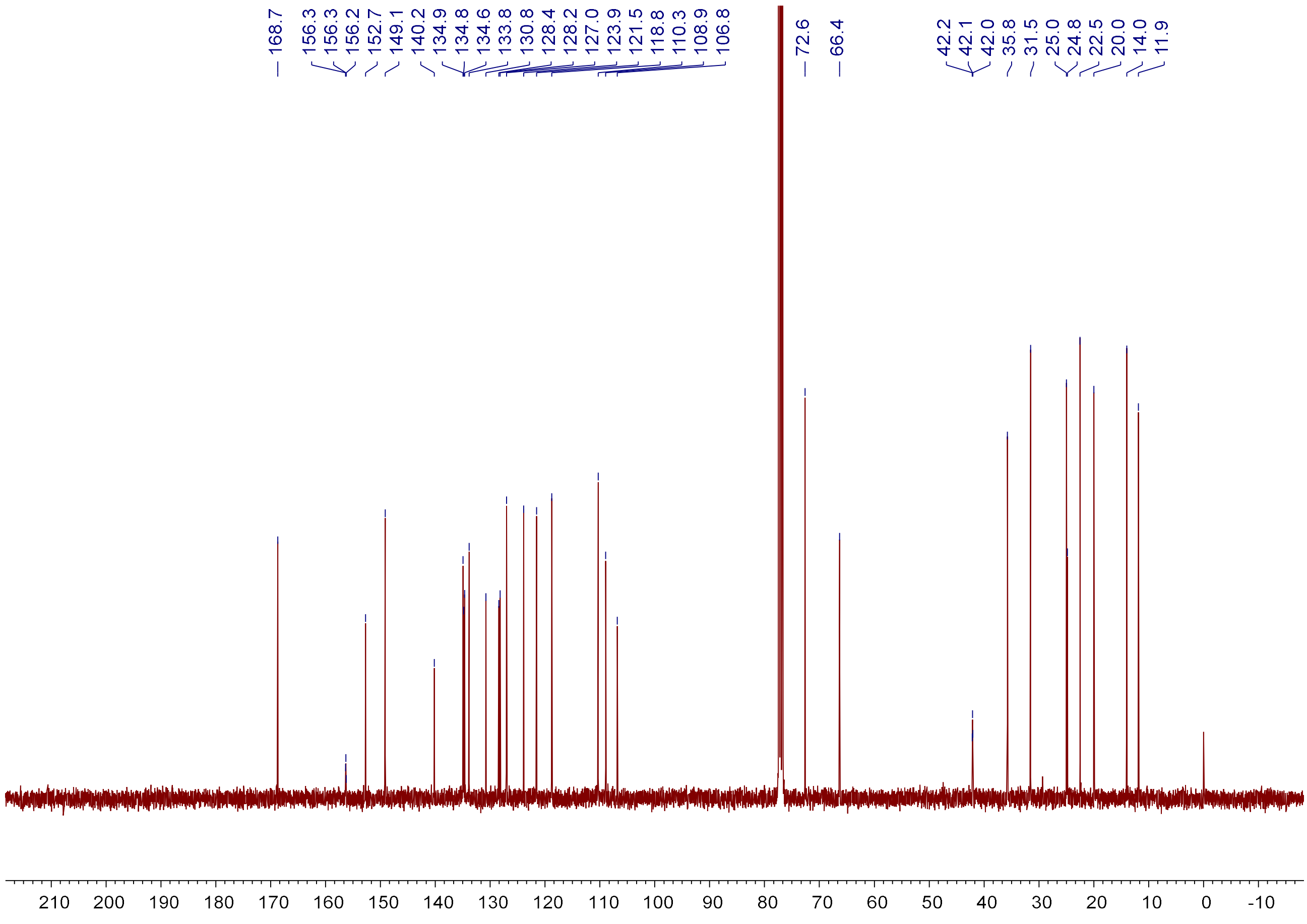
168.7 156.3 156.3 156.2 152.7 149.1 140.2 134.9 134.8 134.6 133.8 130.8 128.4 128.2 127.0 123.9 121.5 118.8 110.3 108.9 106.8 72.6 66.4 42.2 42.1 42.0 35.8 31.5 25.0 24.8 22.5 20.0 14.0 11.9
210 200 190 180 170 160 150 140 130 120 110 100 90 80 70 60 50 40 30 20 10 0 -10

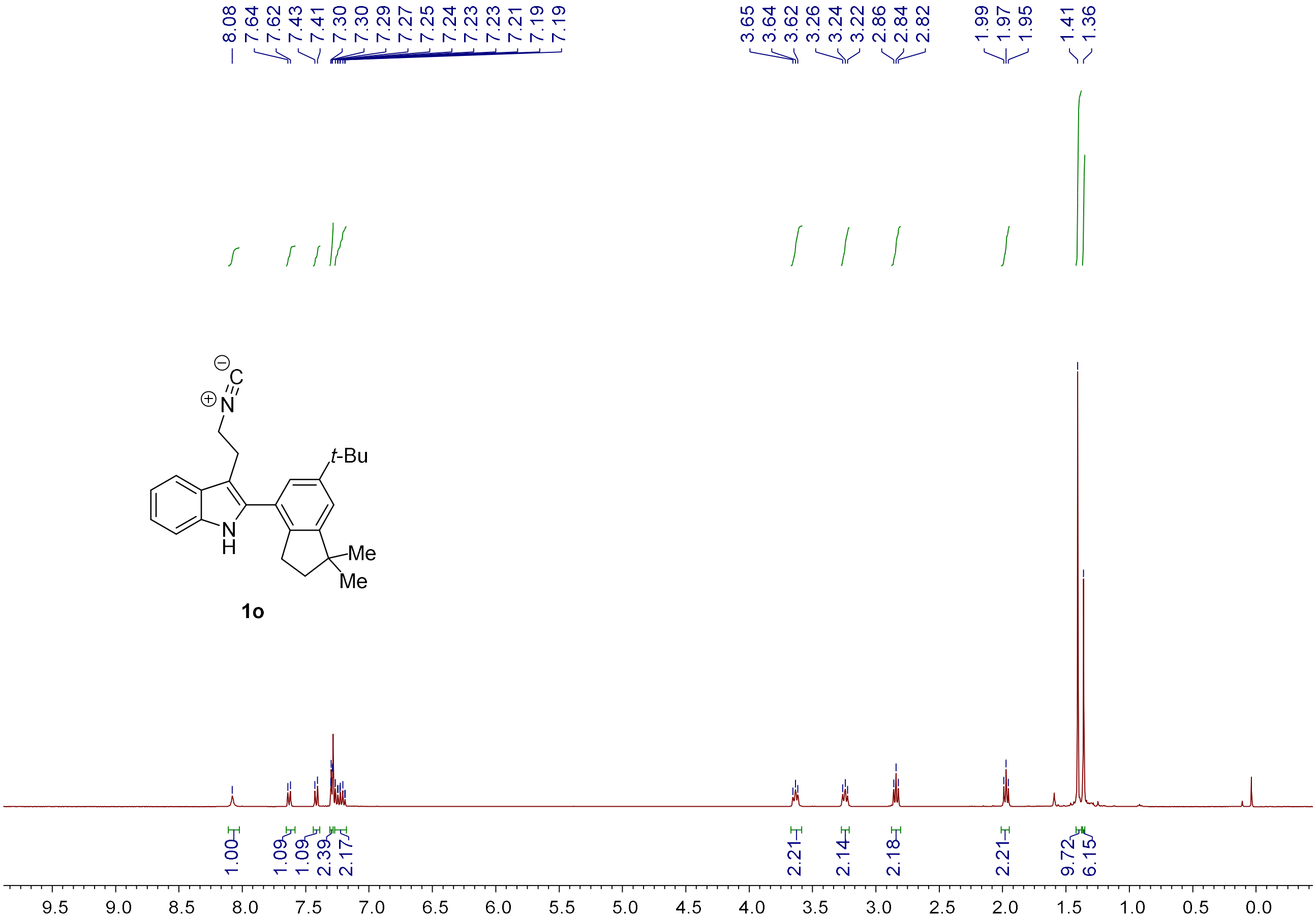
8.08 7.64 7.62 7.43 7.41 7.30 7.30 7.29 7.27 7.25 7.24 7.23 7.23 7.21 7.19 7.19
3.65 3.64 3.62 3.26 3.24 3.22 2.86 2.84 2.82
1.99 1.97 1.95
1.41 1.36
t-Bu
N
H
Me
Me
1o
1.00 1.09 1.09 2.39 2.17
2.21 2.14 2.18
2.21
9.72 6.15
9.5 9.0 8.5 8.0 7.5 7.0 6.5 6.0 5.5 5.0 4.5 4.0 3.5 3.0 2.5 2.0 1.5 1.0 0.5 0.0

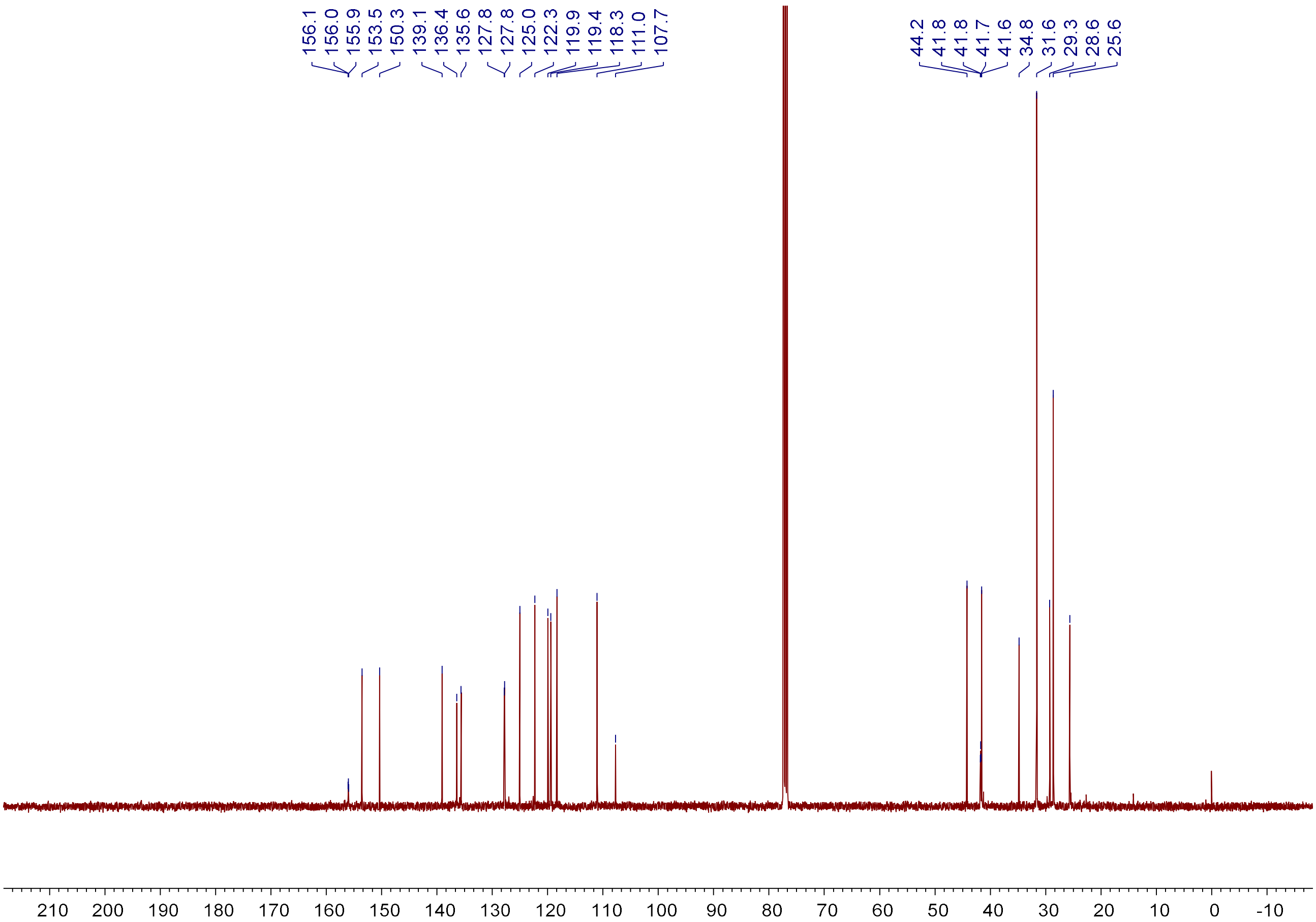
156.1 156.0 155.9 153.5 150.3 139.1 136.4 135.6 127.8 127.8 125.0 122.3 119.9 119.4 118.3 111.0 107.7
44.2 41.8 41.8 41.7 41.6 34.8 31.6 29.3 28.6 25.6
210 200 190 180 170 160 150 140 130 120 110 100 90 80 70 60 50 40 30 20 10 0 -10

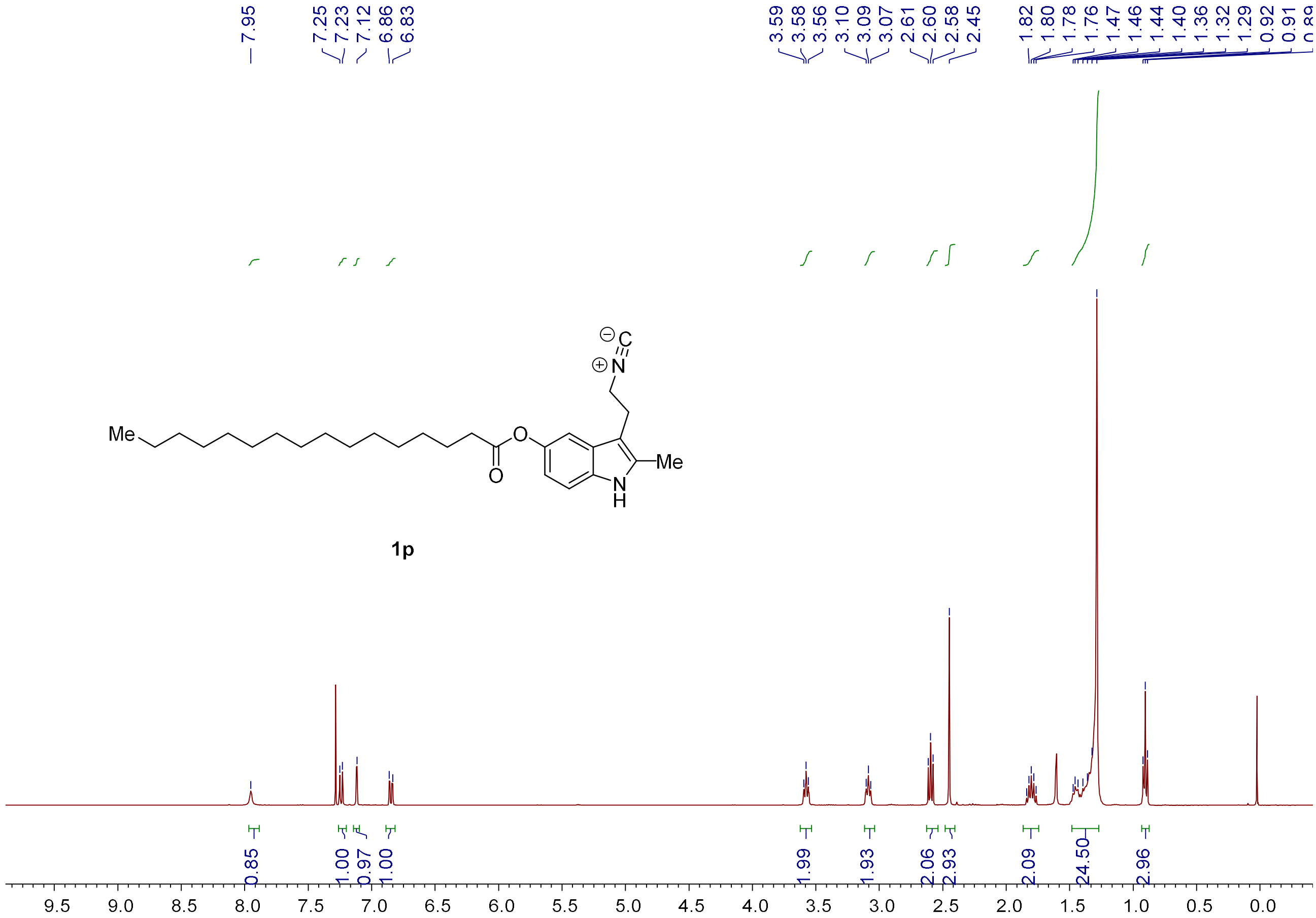
7.95
7.25
7.23
7.12
6.86
6.83
3.59
3.58
3.56
3.10
3.09
3.07
2.61
2.60
2.58
2.45
1.82
1.80
1.78
1.76
1.47
1.46
1.44
1.40
1.36
1.32
1.29
0.92
0.91
Me
O
O
N
C
H
Me
0.85
1.00
0.97
1.00
1.99
1.93
2.06
2.93
2.09
24.50
2.96
9.5
9.0
8.5
8.0
7.5
7.0
6.5
6.0
5.5
5.0
4.5
4.0
3.5
3.0
2.5
2.0
1.5
1.0
0.5
0.0

1p

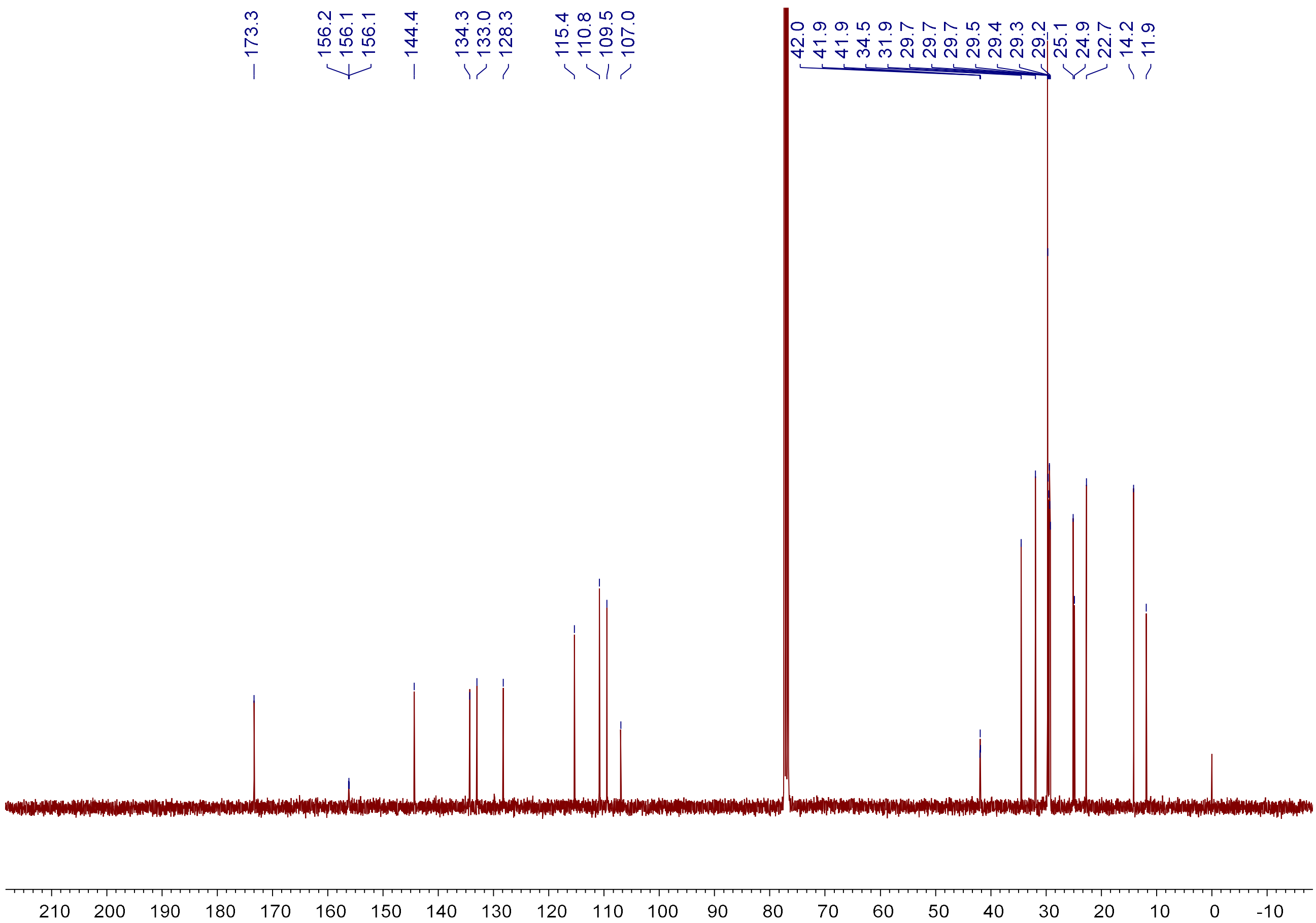
173.3
156.2
156.1
156.1
144.4
134.3
133.0
128.3
115.4
110.8
109.5
107.0
42.0
41.9
41.9
34.5
31.9
29.7
29.7
29.7
29.5
29.4
29.3
29.2
25.1
24.9
22.7
14.2
11.9
210
200
190
180
170
160
150
140
130
120
110
100
90
80
70
60
50
40
30
20
10
0
-10

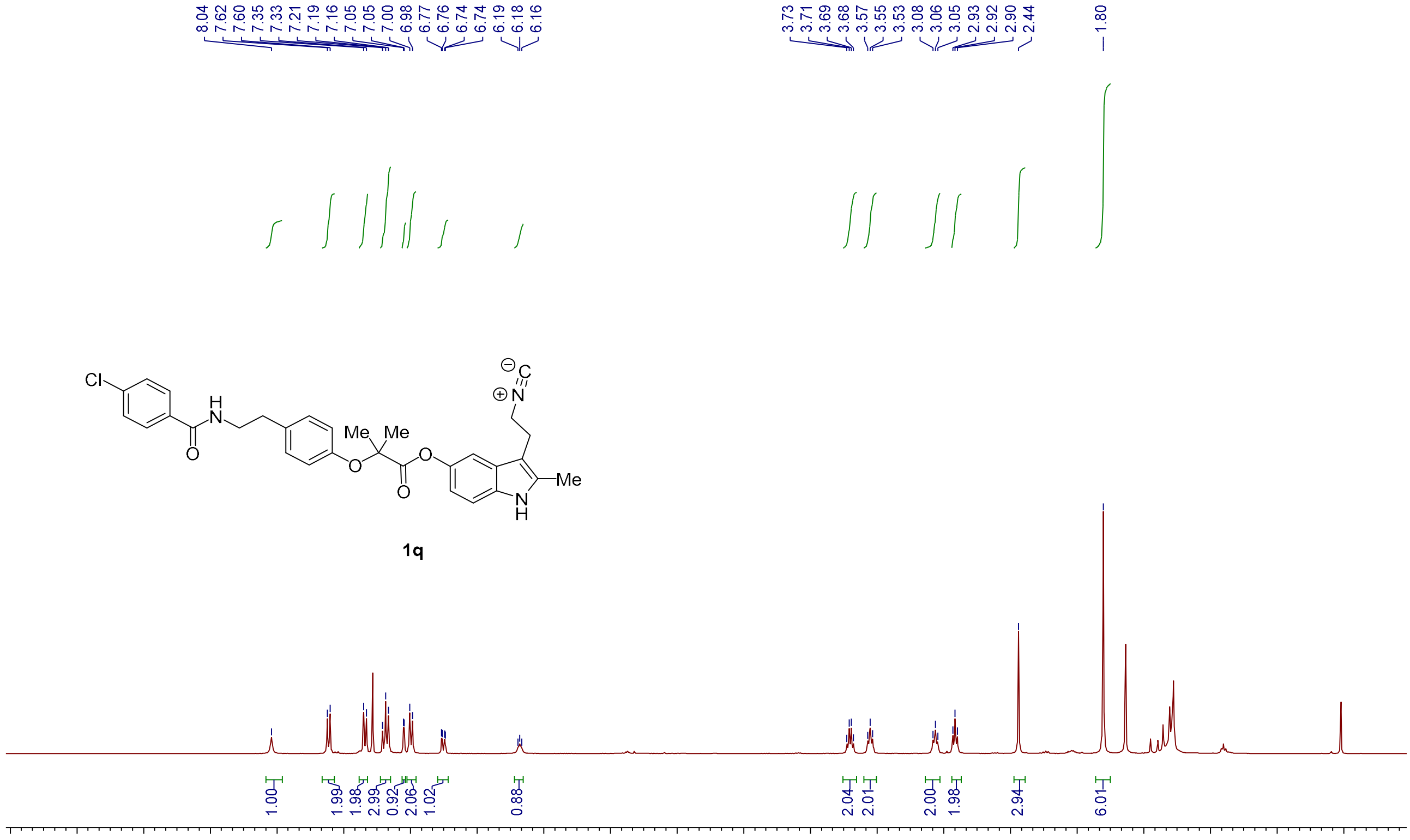

8.04
7.62
7.60
7.35
7.33
7.21
7.19
7.16
7.05
7.05
7.00
6.98
6.77
6.76
6.74
6.74
6.19
6.18
6.16
3.73
3.71
3.69
3.68
3.57
3.55
3.53
3.08
3.06
3.05
2.93
2.92
2.90
2.44
1.80
1.00
1.99
1.98
2.99
0.92
2.06
1.02
0.88
2.04
2.01
2.00
1.98
2.94
6.01
0.0 9.5 9.0 8.5 8.0 7.5 7.0 6.5 6.0 5.5 5.0 4.5 4.0 3.5 3.0 2.5 2.0 1.5 1.0 0.5 0.0
Cl
H
N
O
Me
Me
O
O
O
N
C
Me
N
H

1q

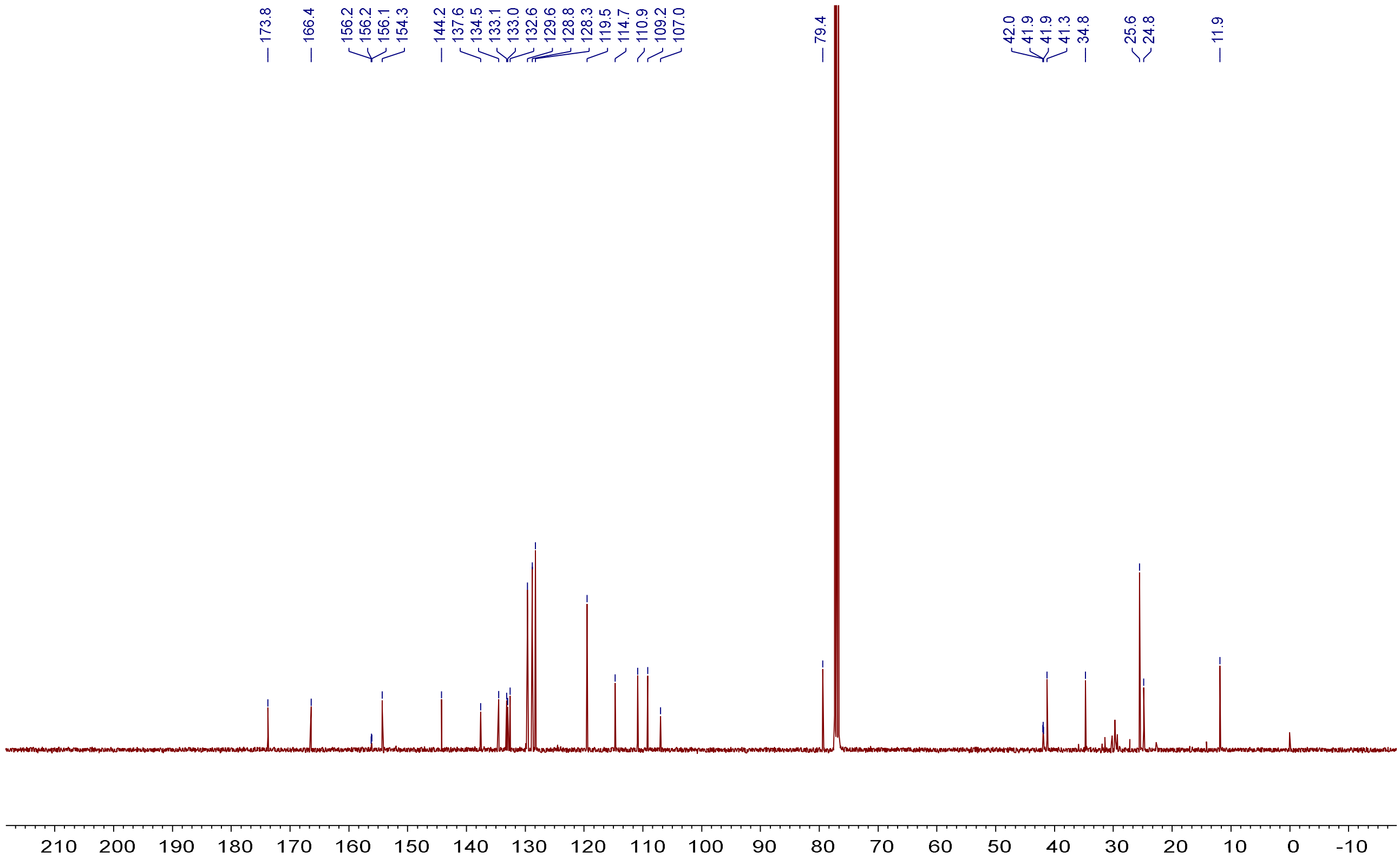

173.8
166.4
156.2
156.2
156.1
154.3
144.2
137.6
134.5
133.1
133.0
132.6
129.6
128.8
128.3
119.5
114.7
110.9
109.2
107.0
79.4
42.0
41.9
41.9
41.3
34.8
25.6
24.8
11.9
210 200 190 180 170 160 150 140 130 120 110 100 90 80 70 60 50 40 30 20 10 0 -10

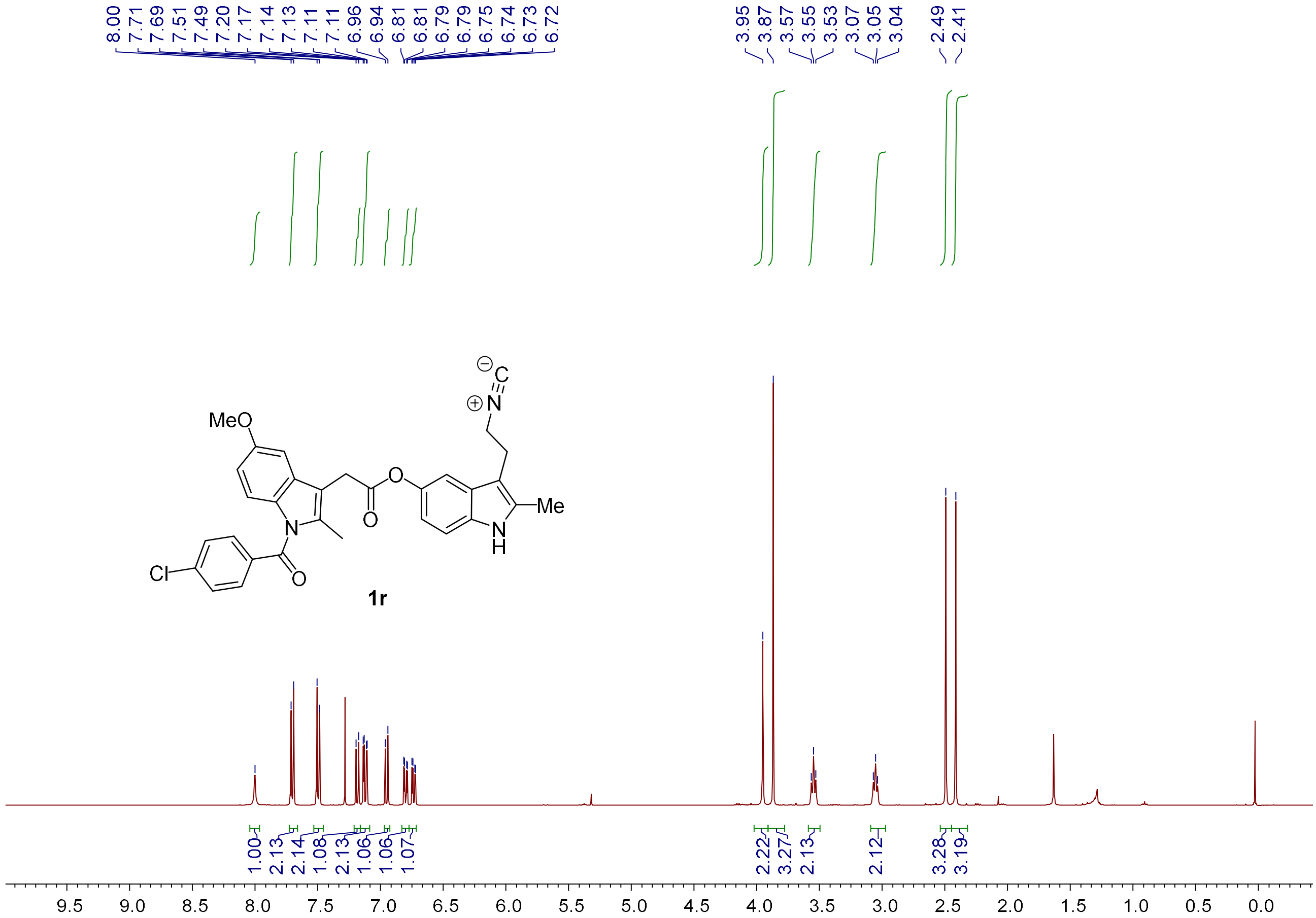
8.00 7.71 7.69 7.51 7.49 7.20 7.17 7.14 7.13 7.11 7.11 6.96 6.94 6.81 6.81 6.79 6.79 6.75 6.74 6.73 6.72
3.95 3.87 3.57 3.55 3.53 3.07 3.05 3.04 2.49 2.41
MeO
N
Cl
O
O
O
N
C
Me
N
H
1.00 2.13 2.14 1.08 2.13 1.06 1.06 1.07
2.22 3.27 2.13 2.12 3.28 3.19
9.5 9.0 8.5 8.0 7.5 7.0 6.5 6.0 5.5 5.0 4.5 4.0 3.5 3.0 2.5 2.0 1.5 1.0 0.5 0.0

1r

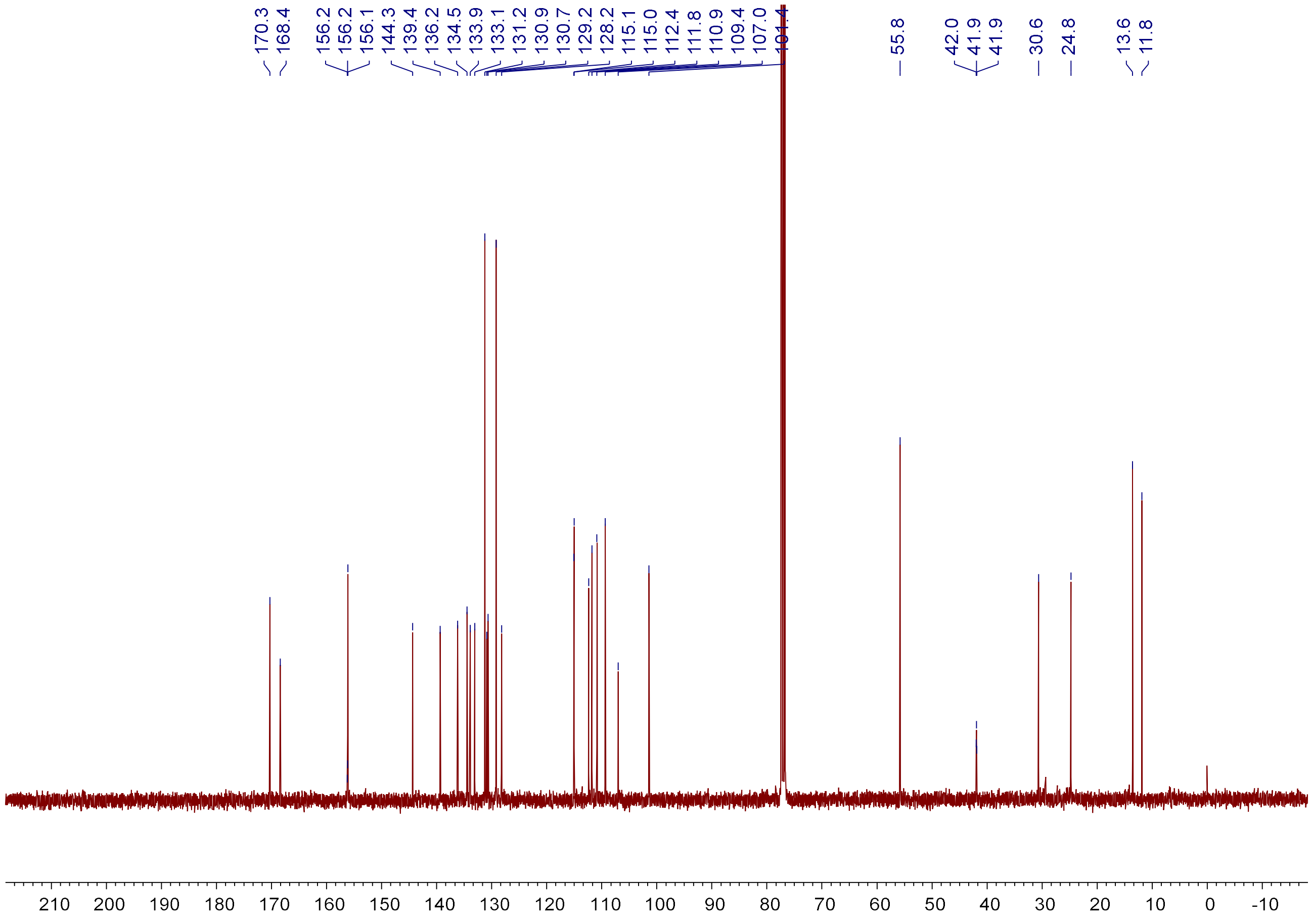
170.3 168.4 156.2 156.2 156.1 144.3 139.4 136.2 134.5 133.9 133.1 131.2 130.9 130.7 129.2 128.2 115.1 115.0 112.4 111.8 110.9 109.4 107.0 101.4
55.8 42.0 41.9 41.9 30.6 24.8 13.6 11.8
210 200 190 180 170 160 150 140 130 120 110 100 90 80 70 60 50 40 30 20 10 0 -10

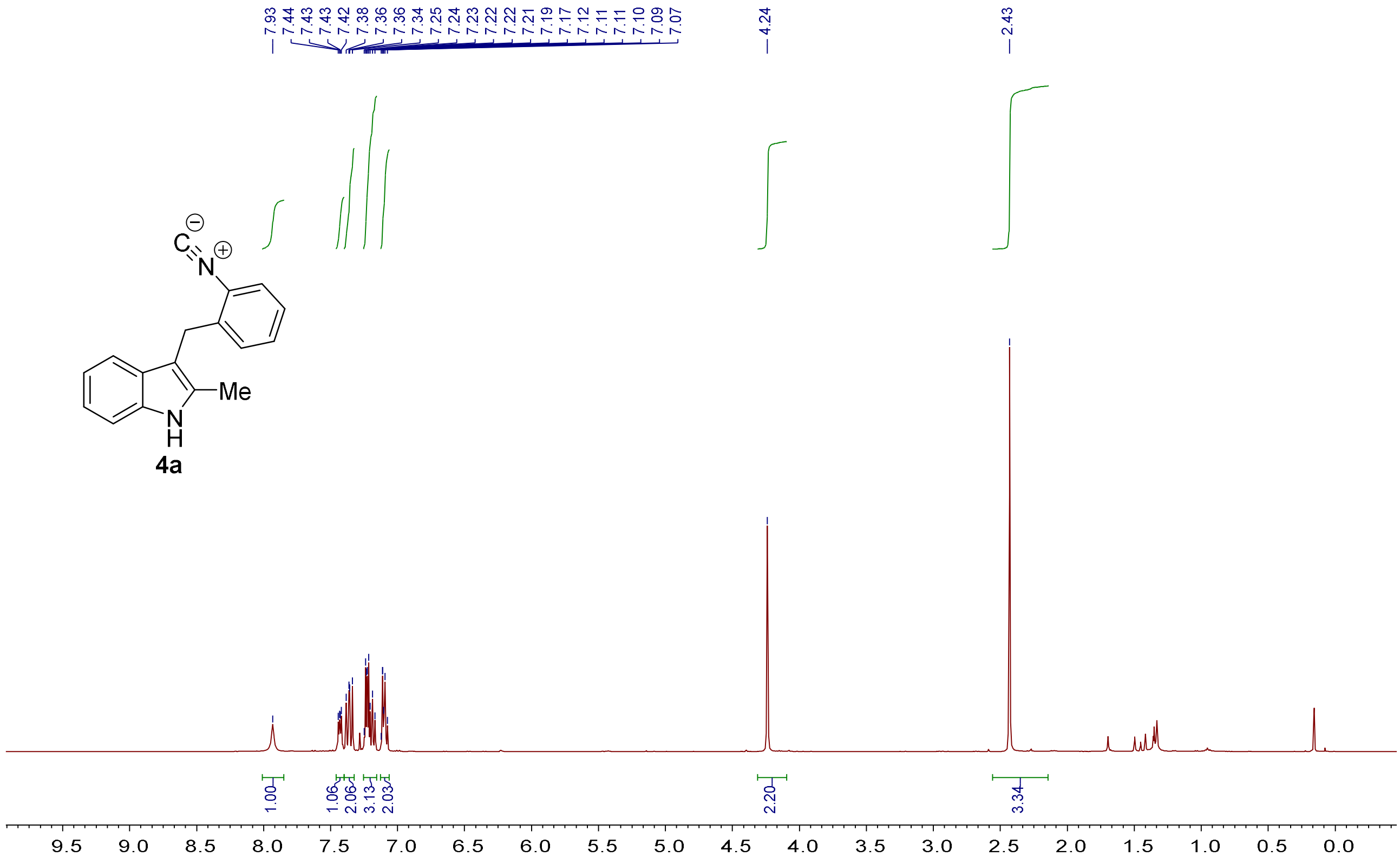
7.93
7.44
7.43
7.43
7.42
7.38
7.36
7.36
7.34
7.25
7.24
7.23
7.22
7.22
7.21
7.19
7.17
7.12
7.11
7.11
7.10
7.09
7.07
4.24
2.43
Me
N
H
4a
1.00
1.06
2.06
3.13
2.03
2.20
3.34
9.5 9.0 8.5 8.0 7.5 7.0 6.5 6.0 5.5 5.0 4.5 4.0 3.5 3.0 2.5 2.0 1.5 1.0 0.5 0.0

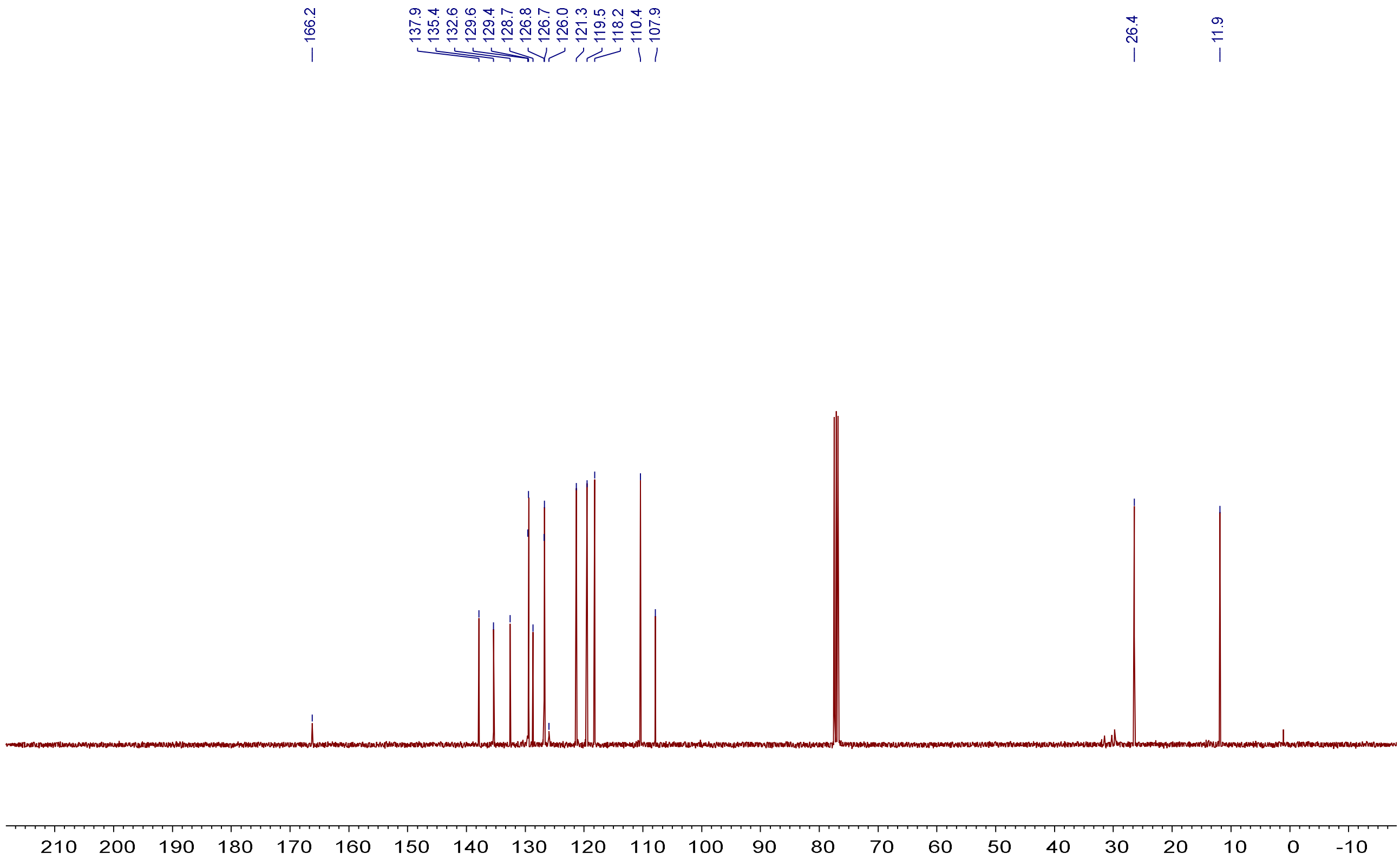
166.2
137.9
135.4
132.6
129.6
129.4
128.7
126.8
126.7
126.0
121.3
119.5
118.2
110.4
107.9
26.4
11.9
210 200 190 180 170 160 150 140 130 120 110 100 90 80 70 60 50 40 30 20 10 0 -10

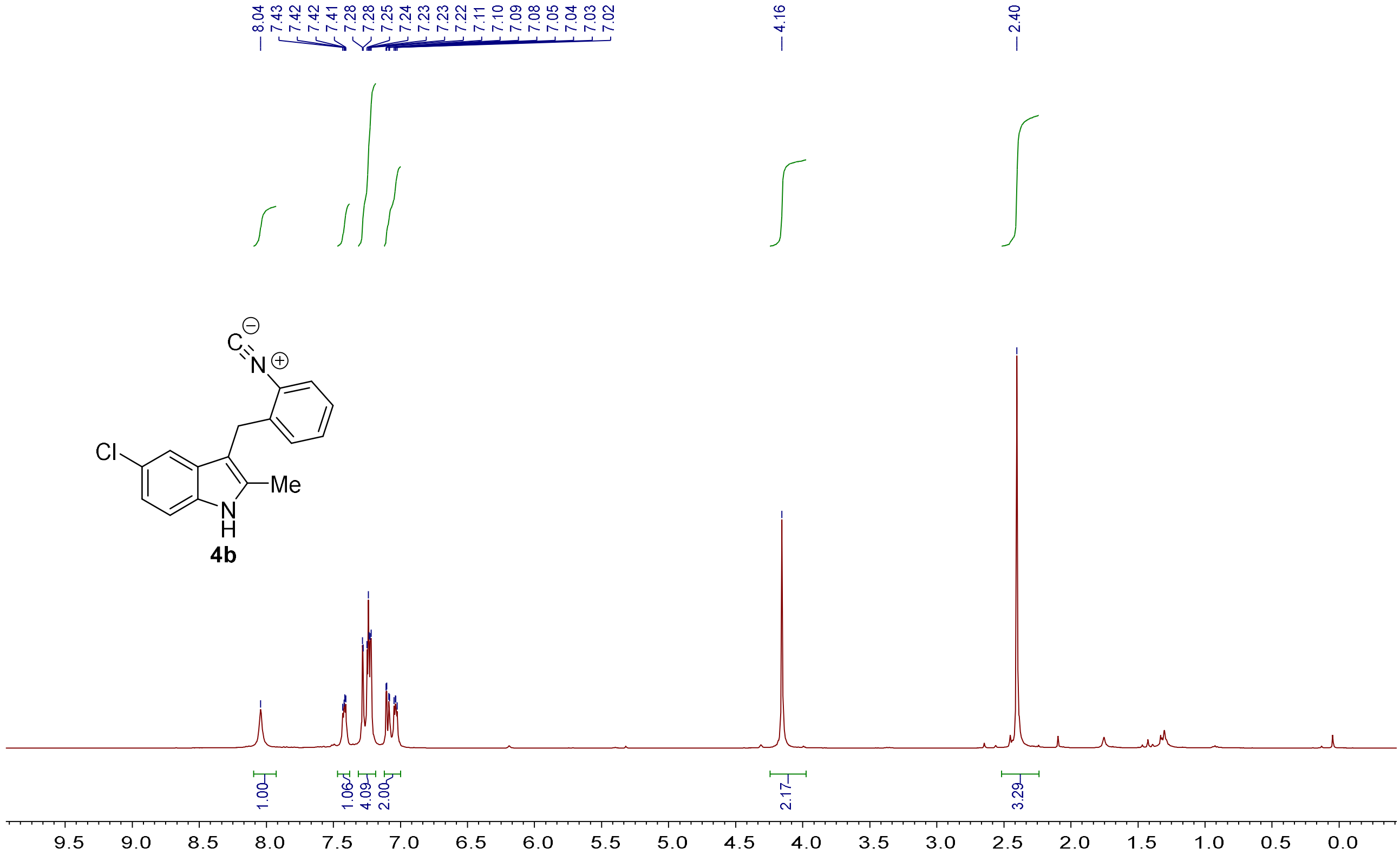
8.04
7.43
7.42
7.42
7.41
7.28
7.28
7.25
7.24
7.23
7.23
7.22
7.11
7.10
7.09
7.08
7.05
7.04
7.03
7.02
4.16
2.40
Cl
Me
N
H
4b
1.00
1.06
4.09
2.00
2.17
3.29
9.5 9.0 8.5 8.0 7.5 7.0 6.5 6.0 5.5 5.0 4.5 4.0 3.5 3.0 2.5 2.0 1.5 1.0 0.5 0.0

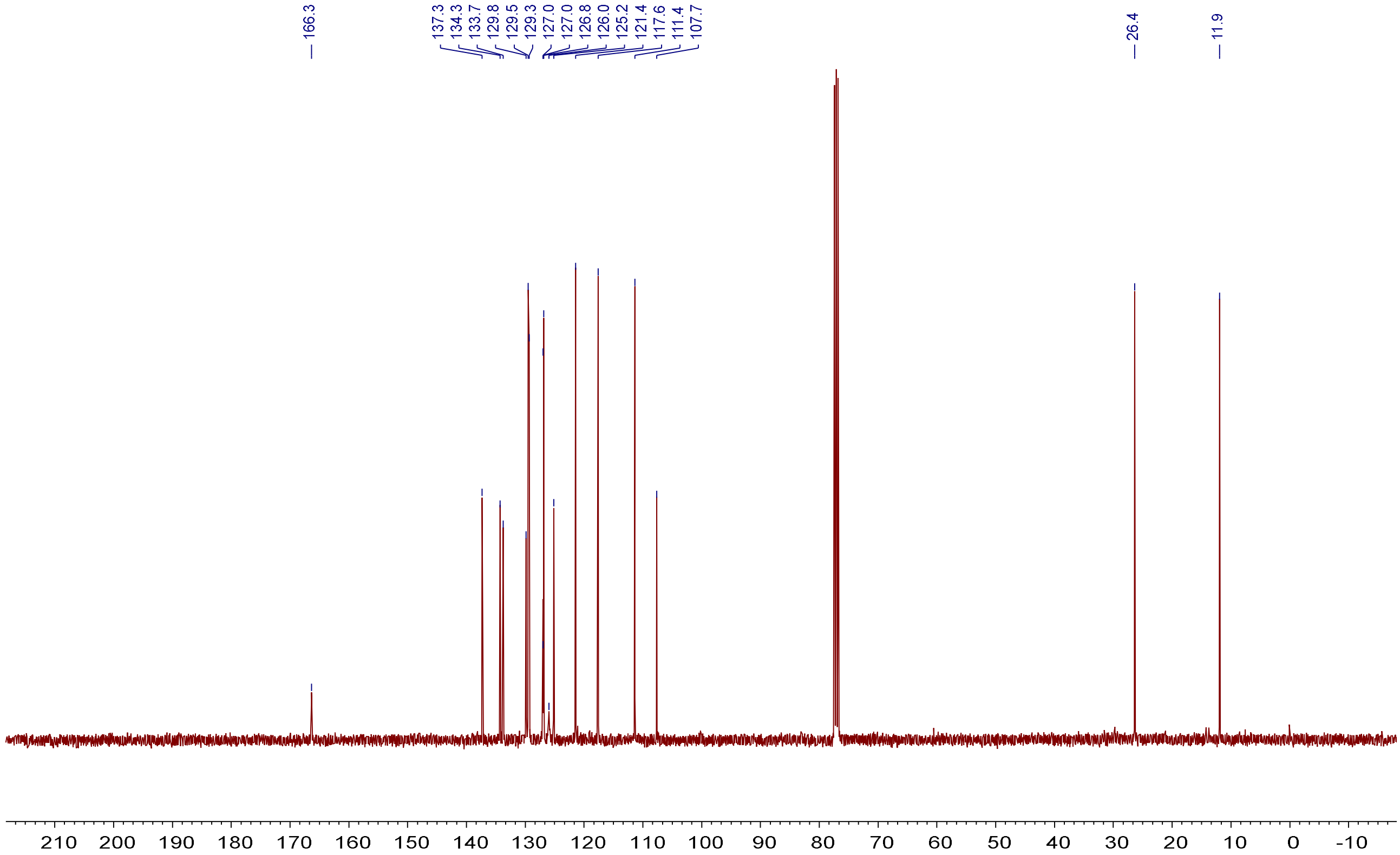
166.3
137.3
134.3
133.7
129.8
129.5
129.3
127.0
127.0
126.8
126.0
125.2
121.4
117.6
111.4
107.7
26.4
11.9
210 200 190 180 170 160 150 140 130 120 110 100 90 80 70 60 50 40 30 20 10 0 -10

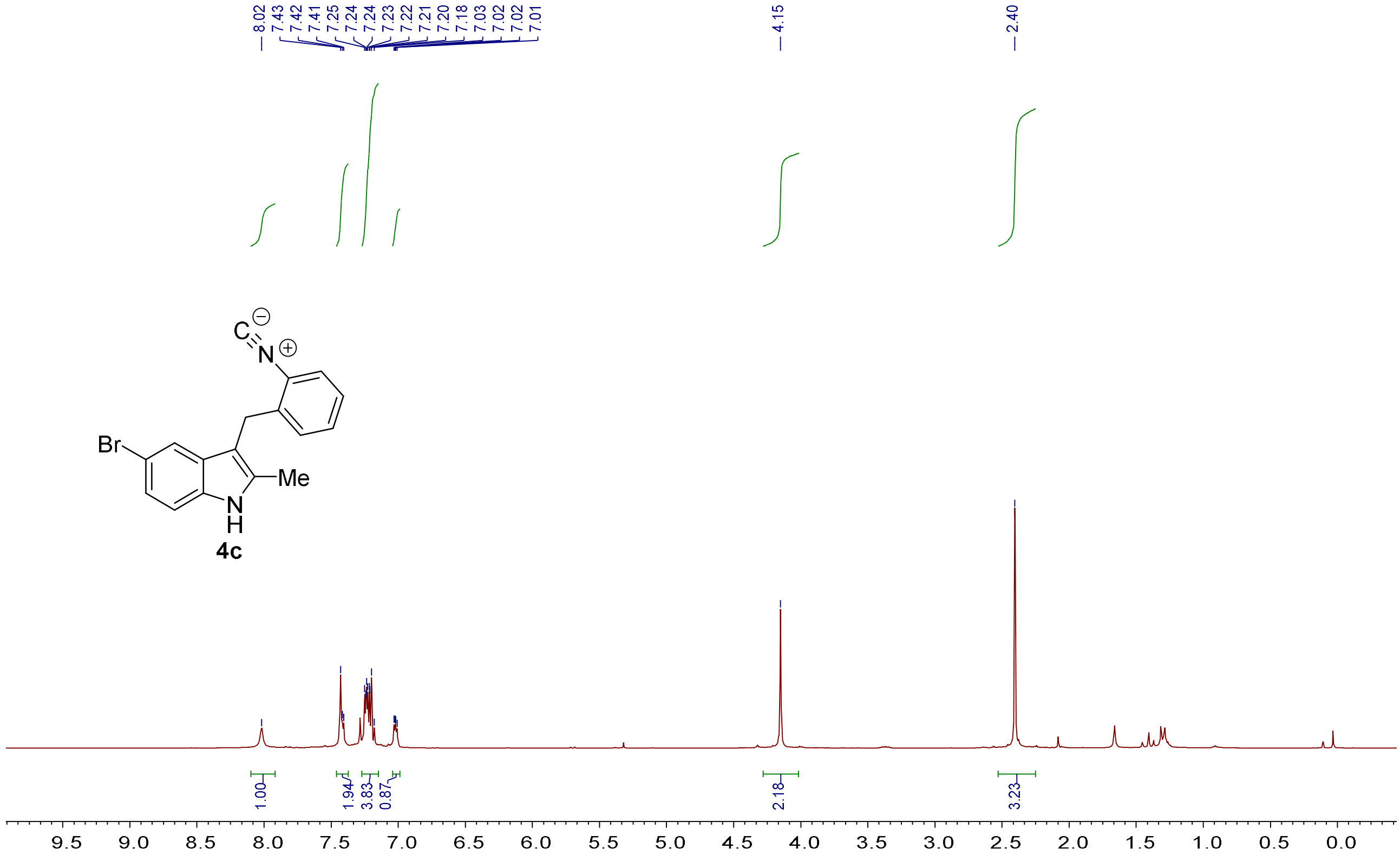
8.02
7.43
7.42
7.41
7.25
7.24
7.24
7.23
7.22
7.21
7.20
7.18
7.03
7.02
7.02
7.01
4.15
2.40
Br
Me
N
H
4c
1.00
1.94
3.83
0.87
2.18
3.23
9.5 9.0 8.5 8.0 7.5 7.0 6.5 6.0 5.5 5.0 4.5 4.0 3.5 3.0 2.5 2.0 1.5 1.0 0.5 0.0

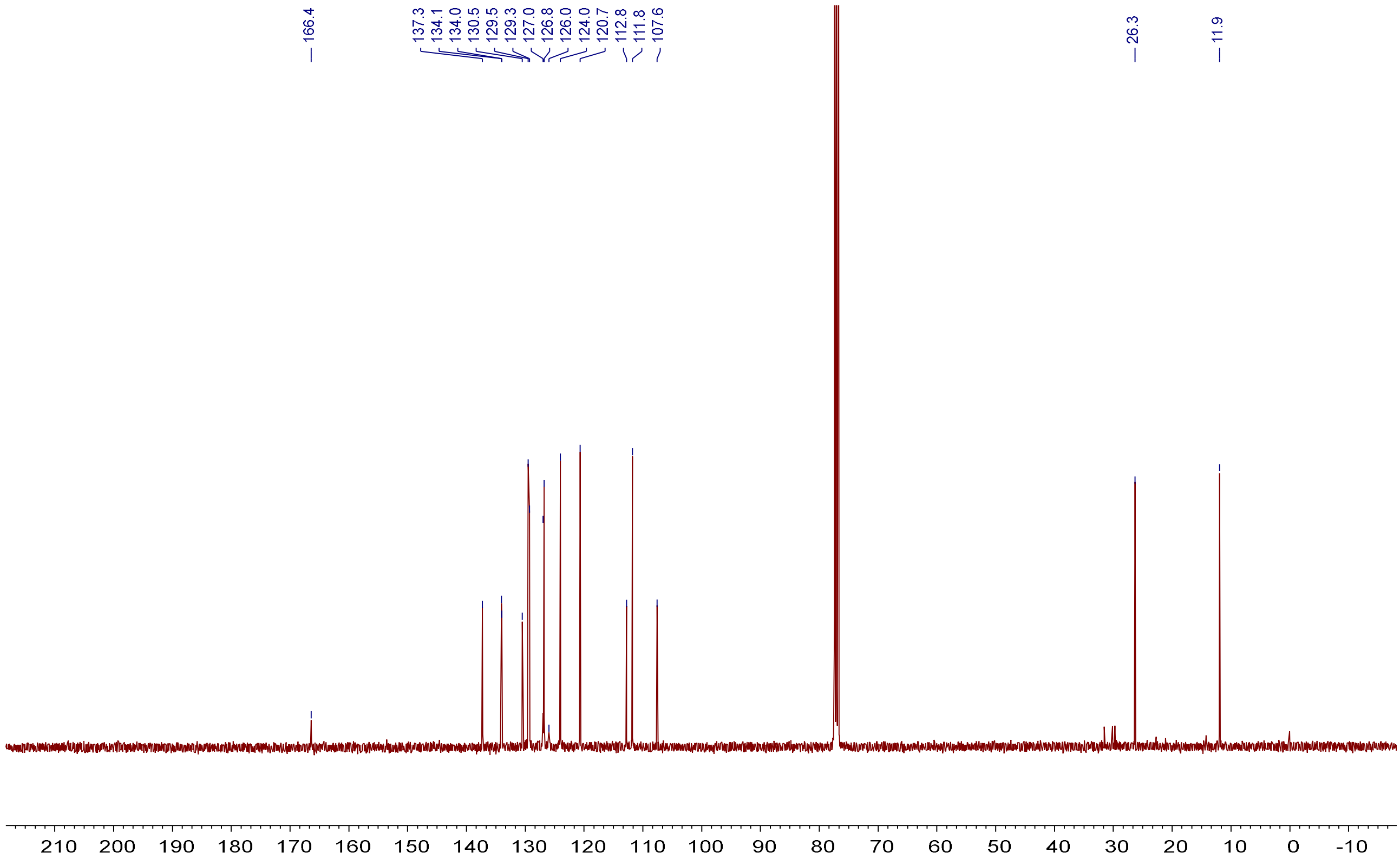
166.4
137.3
134.1
134.0
130.5
129.5
129.3
127.0
126.8
126.0
124.0
120.7
112.8
111.8
107.6
26.3
11.9
210 200 190 180 170 160 150 140 130 120 110 100 90 80 70 60 50 40 30 20 10 0 -10

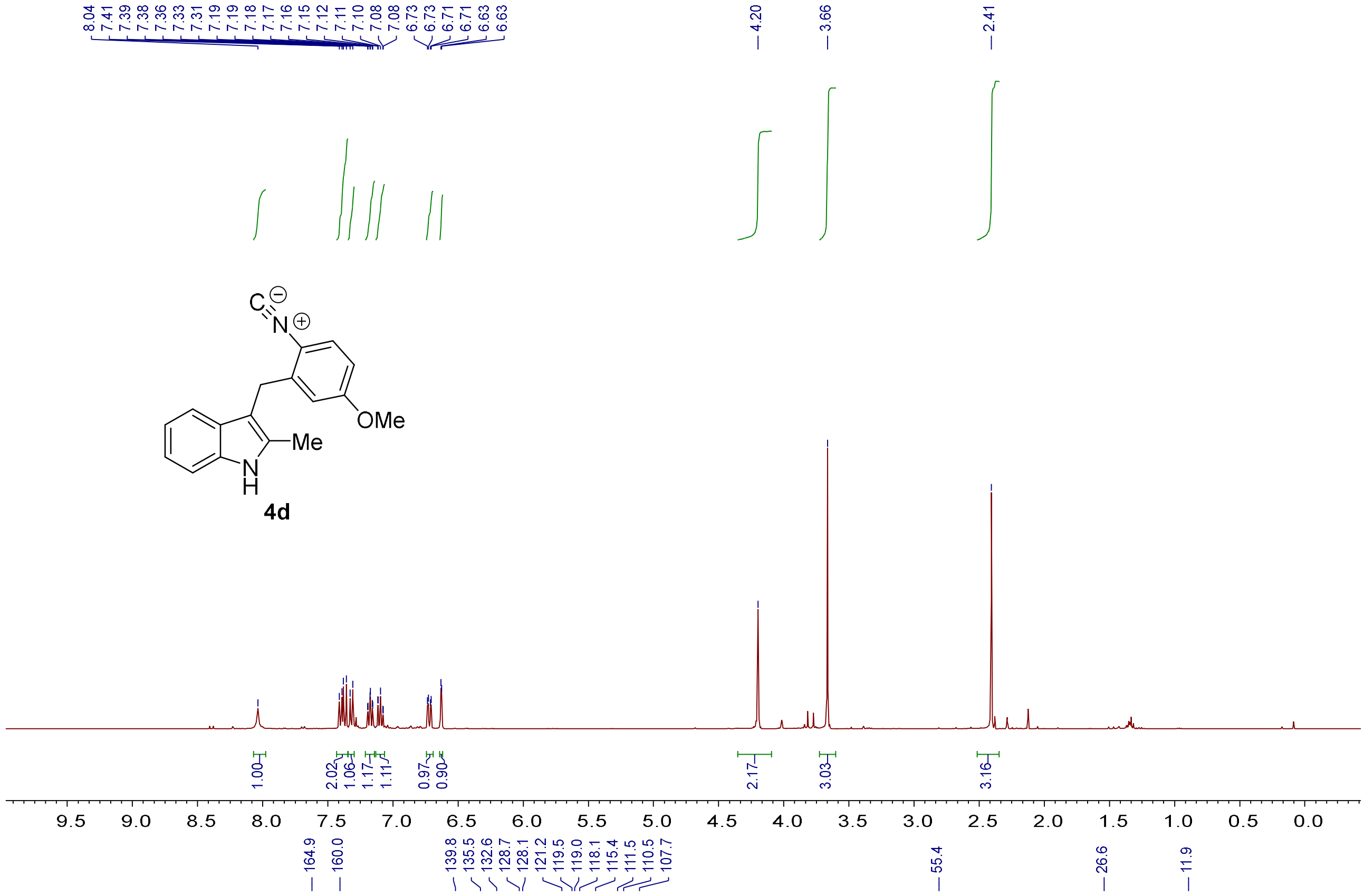
8.04 7.41 7.39 7.38 7.36 7.33 7.31 7.19 7.19 7.18 7.17 7.16 7.15 7.12 7.11 7.10 7.08 7.08 6.73 6.73 6.71 6.71 6.63 6.63
4.20
3.66
2.41
C
N
OMe
Me
N
H
4d
1.00
2.02 1.06 1.17 1.11
0.97 0.90
2.17
3.03
3.16
9.5 9.0 8.5 8.0 7.5 7.0 6.5 6.0 5.5 5.0 4.5 4.0 3.5 3.0 2.5 2.0 1.5 1.0 0.5 0.0

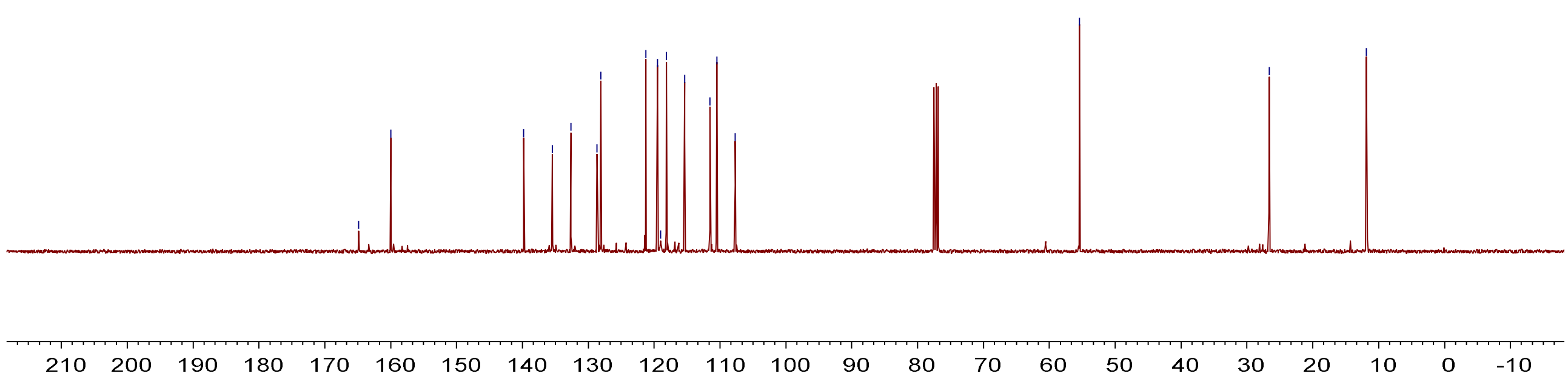
164.9
160.0
139.8 135.5 132.6 128.7 128.1 121.2 119.5 119.0 118.1 115.4 111.5 110.5 107.7
55.4
26.6
11.9
210 200 190 180 170 160 150 140 130 120 110 100 90 80 70 60 50 40 30 20 10 0 -10

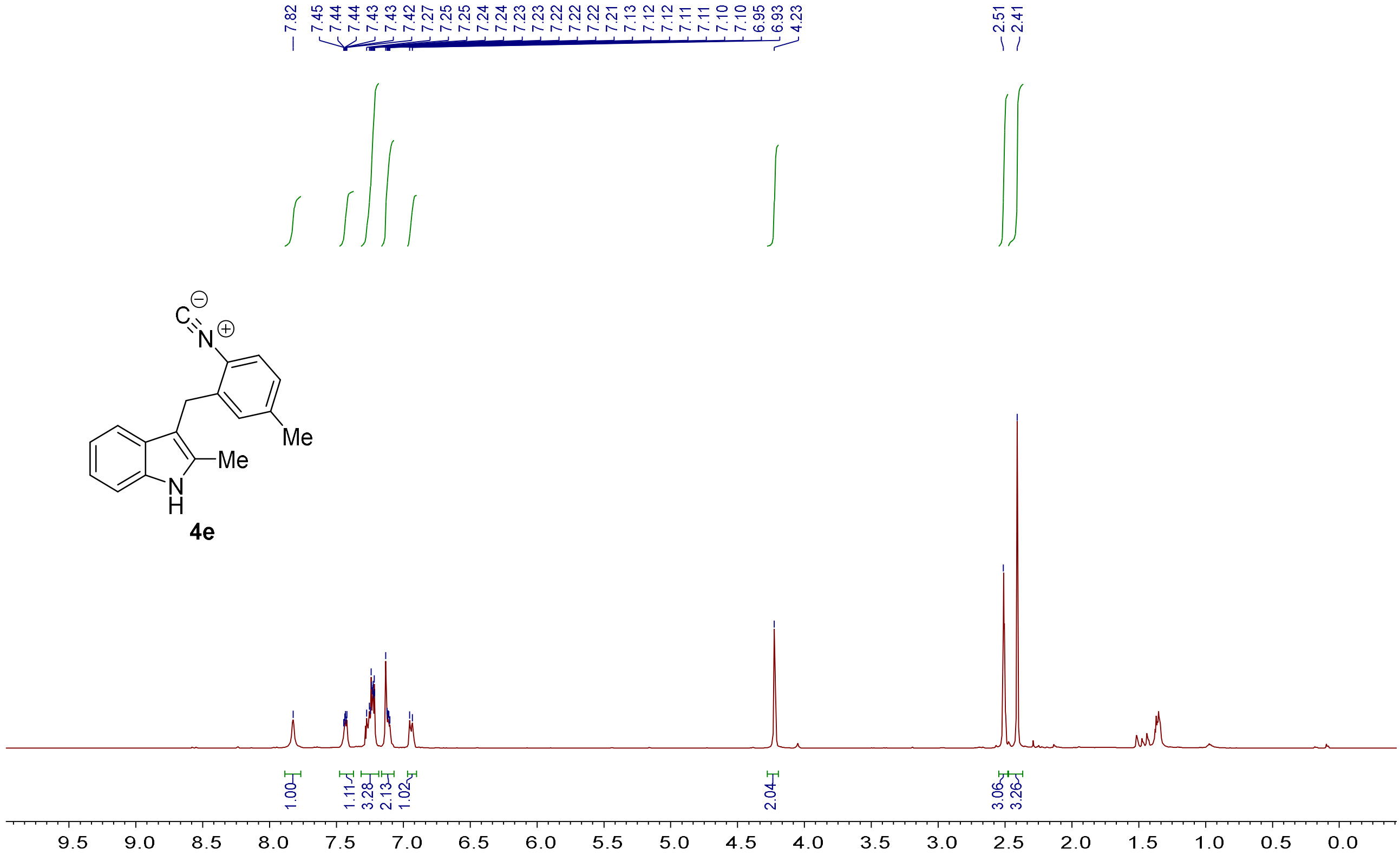
7.82
7.45
7.44
7.44
7.43
7.43
7.42
7.27
7.25
7.25
7.24
7.24
7.23
7.23
7.22
7.22
7.22
7.21
7.13
7.12
7.12
7.11
7.11
7.10
7.10
6.95
6.93
4.23
2.51
2.41
1.00
1.11
3.28
2.13
1.02
2.04
3.06
3.26
Me
Me
N
H

4e

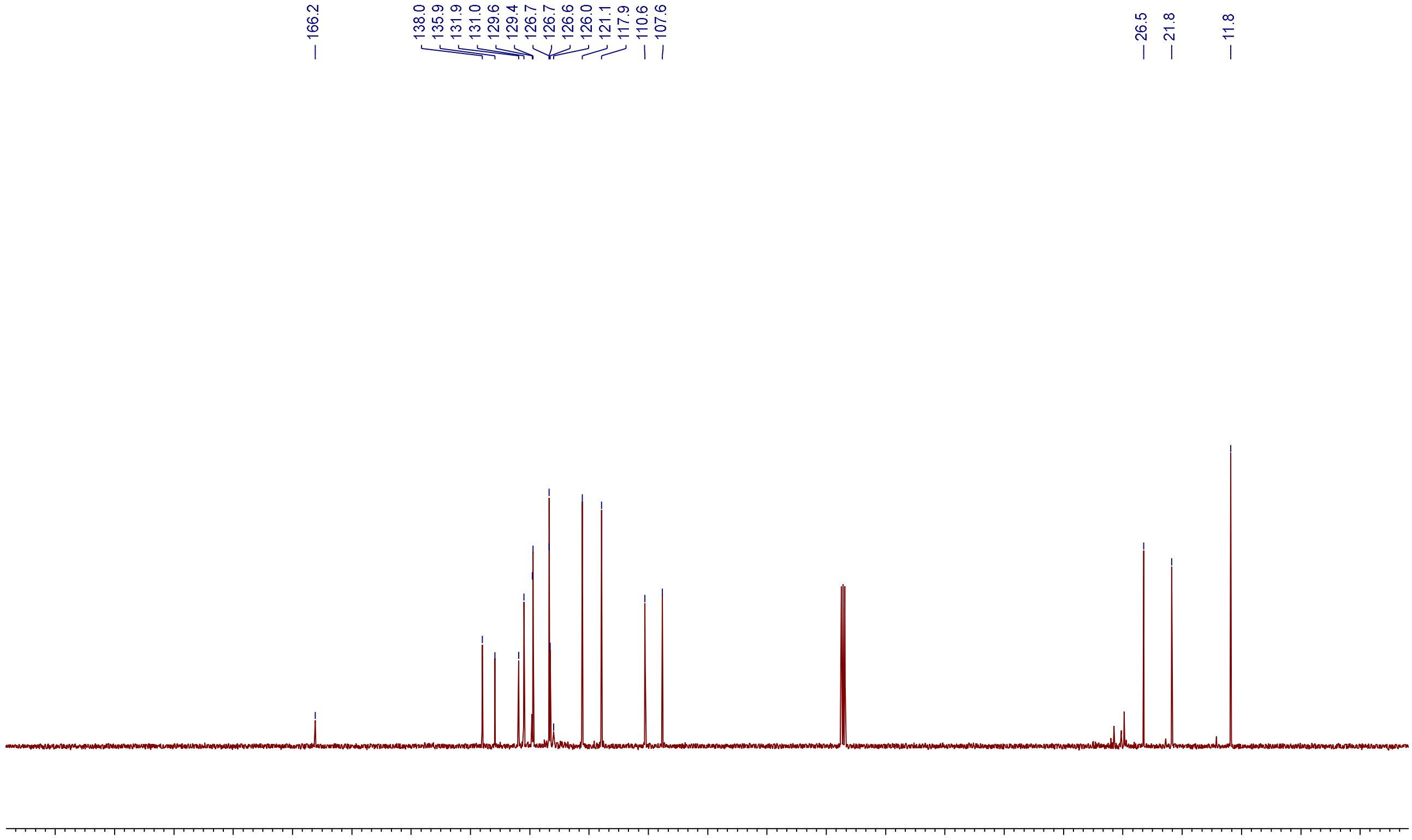
166.2
138.0
135.9
131.9
131.0
129.6
129.4
126.7
126.7
126.6
126.0
121.1
117.9
110.6
107.6
26.5
21.8
11.8

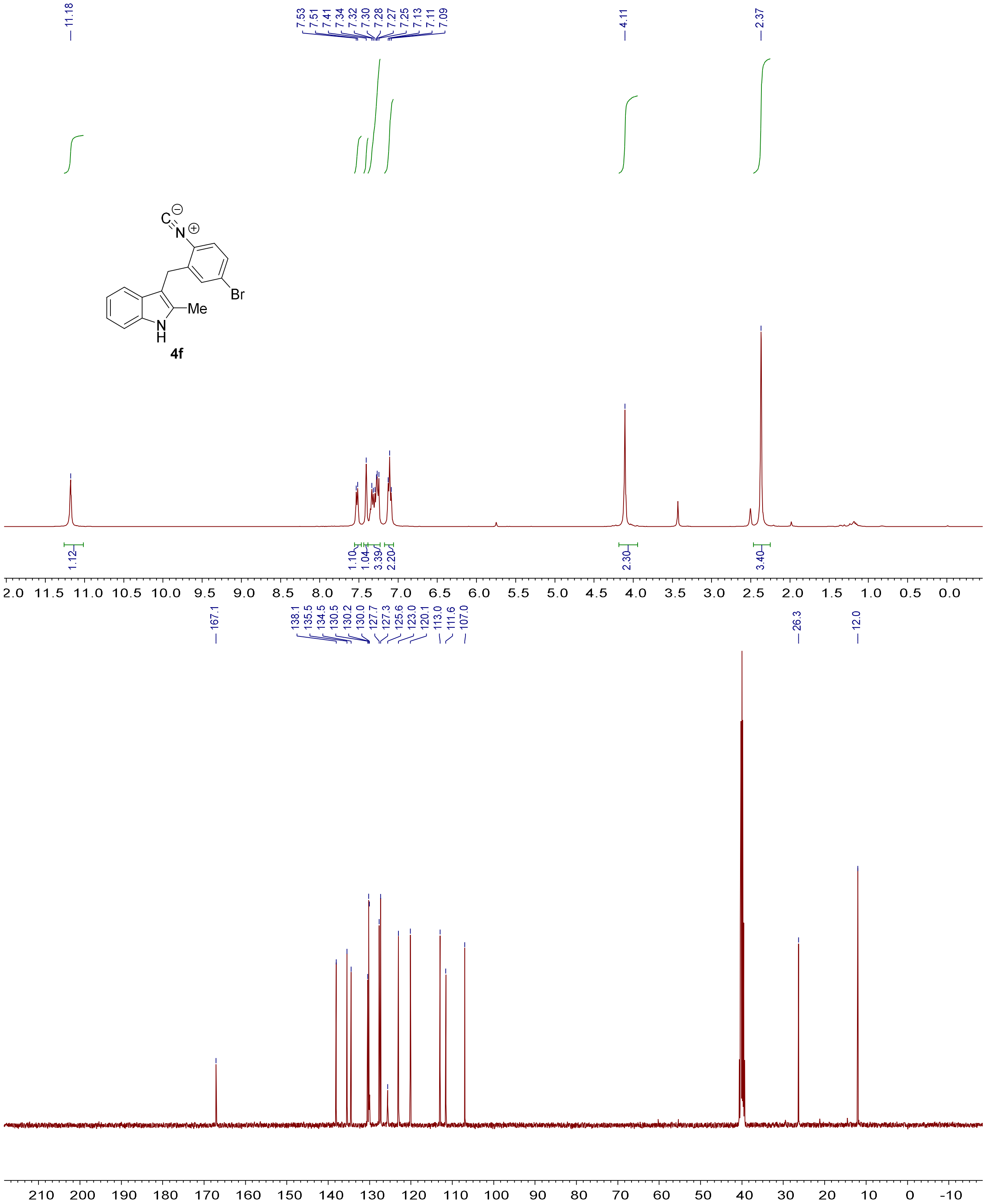
11.18
7.53
7.51
7.41
7.34
7.32
7.30
7.28
7.27
7.25
7.13
7.11
7.09
4.11
2.37
4f
1.12
1.10
1.04
3.39
2.20
2.30
3.40
167.1
138.1
135.5
134.5
130.5
130.2
130.0
127.7
127.3
125.6
123.0
120.1
113.0
111.6
107.0
26.3
12.0

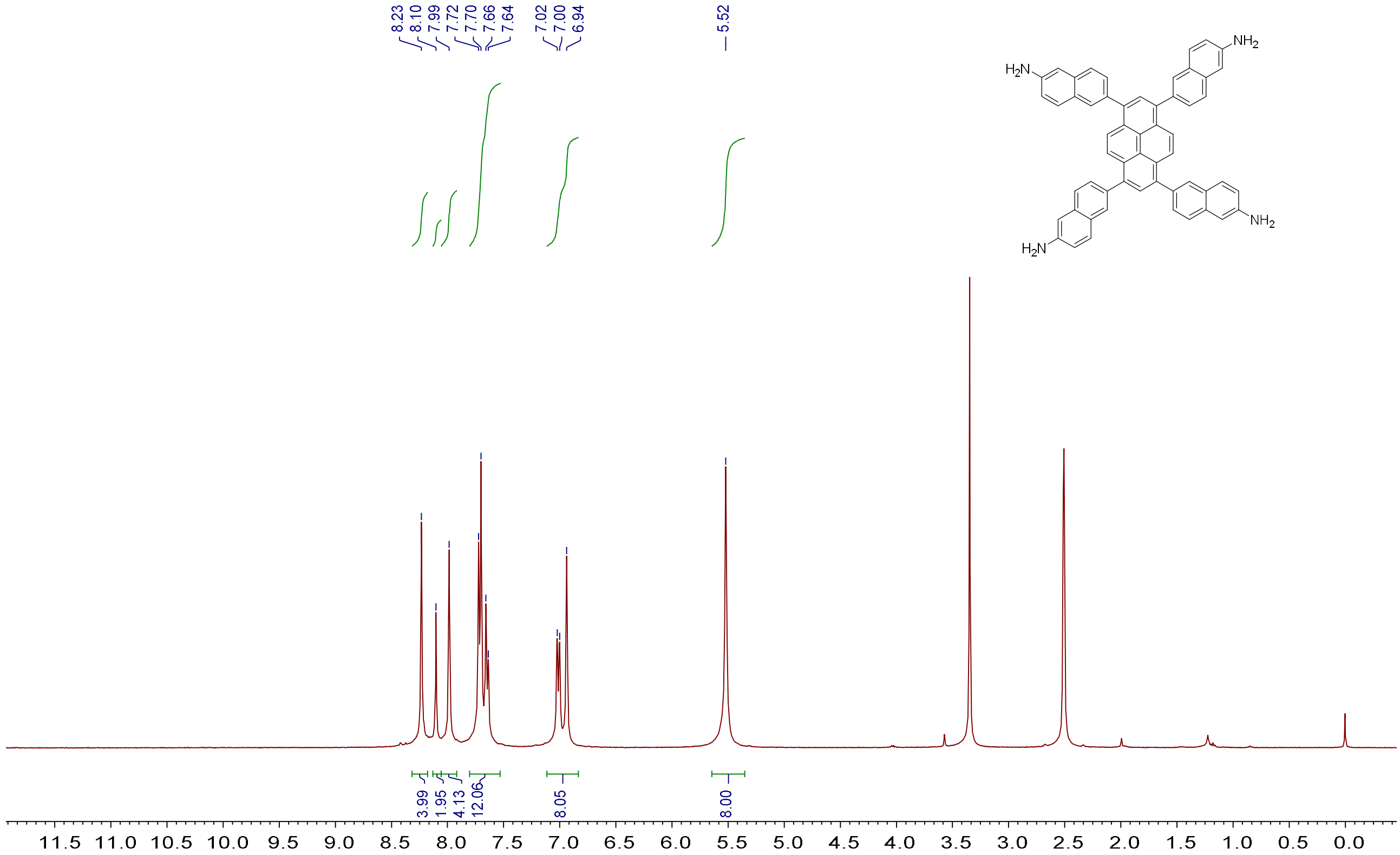
8.23
8.10
7.99
7.72
7.70
7.66
7.64
7.02
7.00
6.94
5.52
3.99
1.95
4.13
12.06
8.05
8.00
11.5 11.0 10.5 10.0 9.5 9.0 8.5 8.0 7.5 7.0 6.5 6.0 5.5 5.0 4.5 4.0 3.5 3.0 2.5 2.0 1.5 1.0 0.5 0.0
H_2N
NH_2
H_2N
NH_2

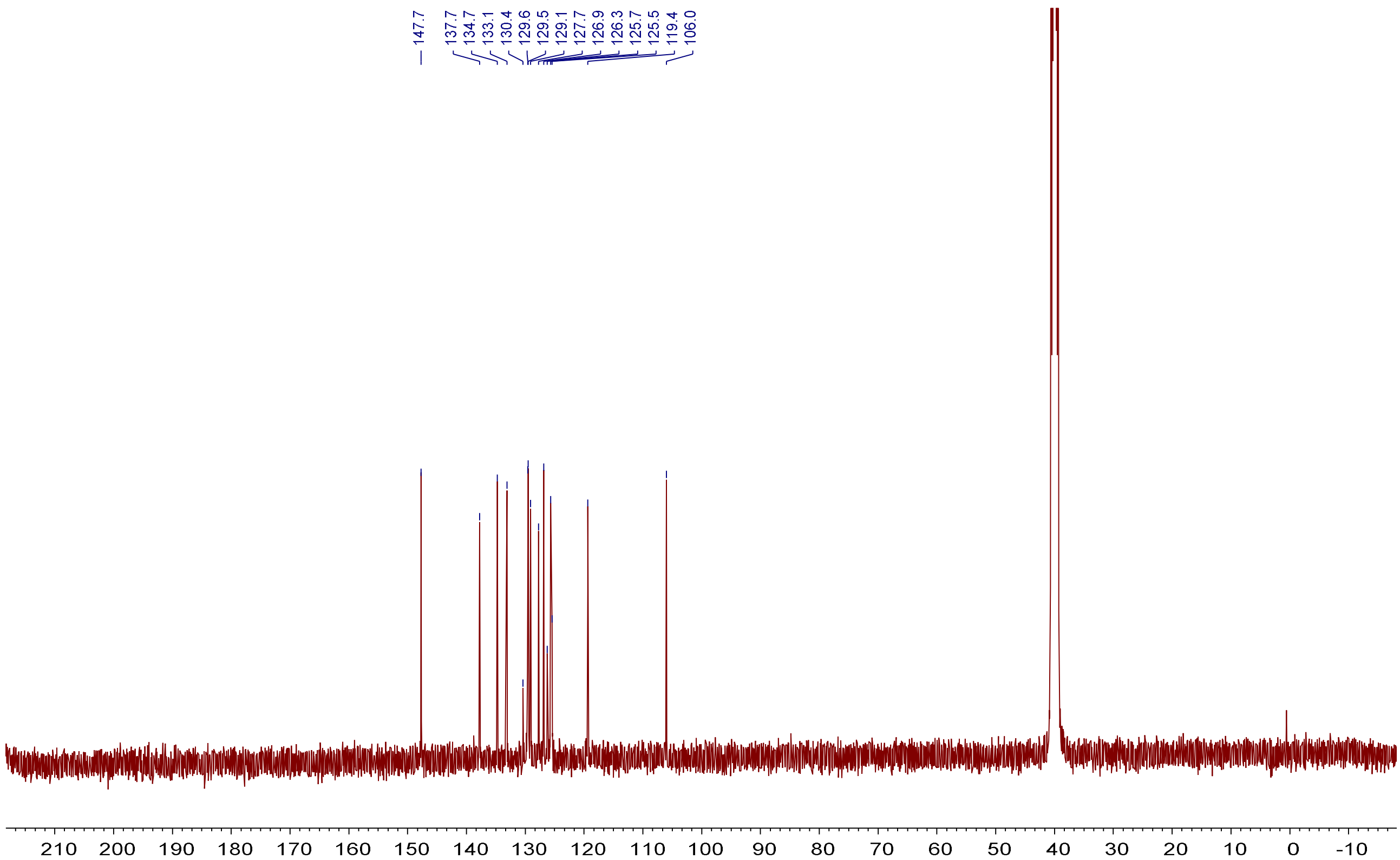
147.7
137.7
134.7
133.1
130.4
129.6
129.5
129.1
127.7
126.9
126.3
125.7
125.5
119.4
106.0
210 200 190 180 170 160 150 140 130 120 110 100 90 80 70 60 50 40 30 20 10 0 -10

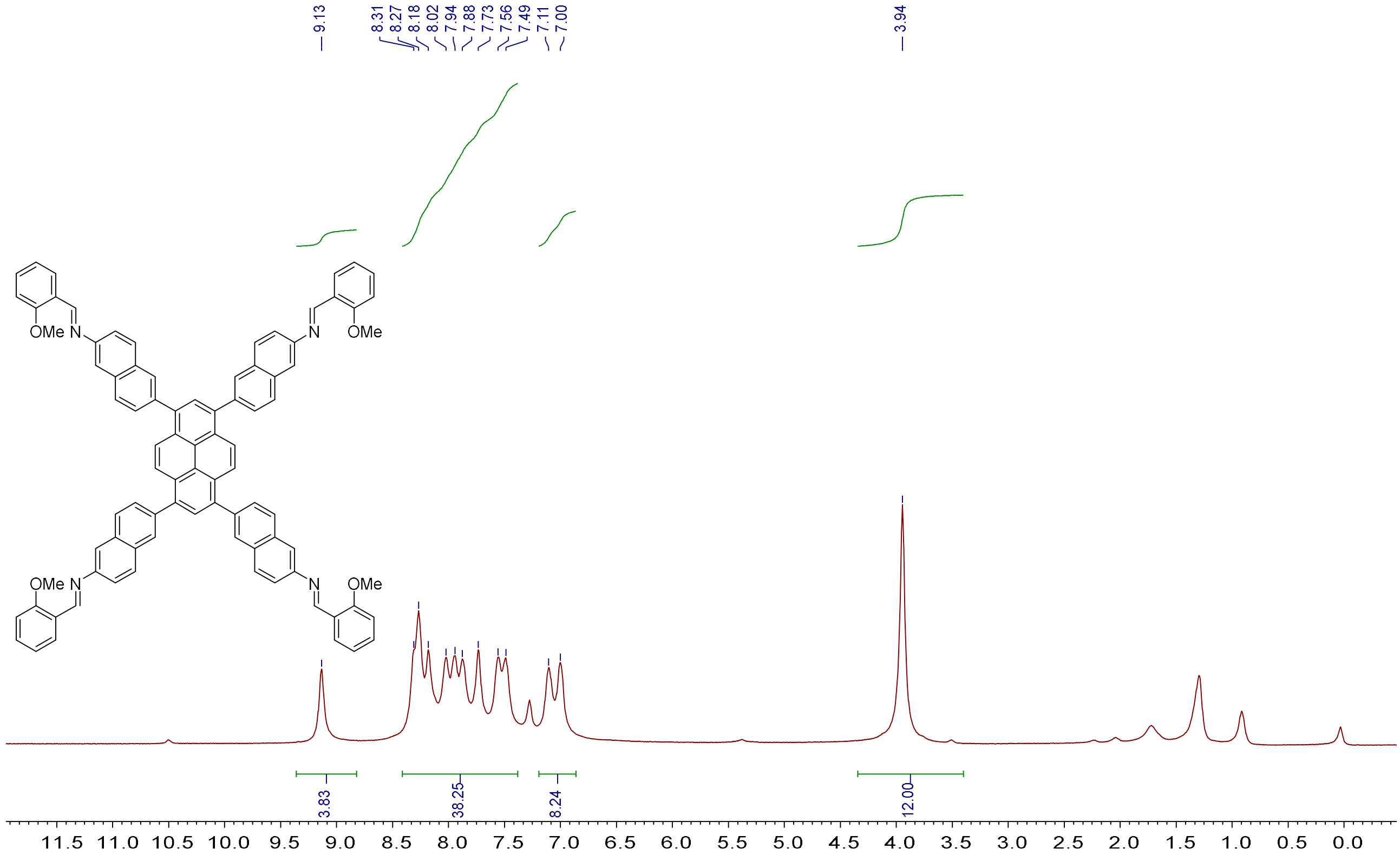
9.13
8.31
8.27
8.18
8.02
7.94
7.88
7.73
7.56
7.49
7.11
7.00
3.94
OMe
N
3.83
38.25
8.24
12.00
11.5 11.0 10.5 10.0 9.5 9.0 8.5 8.0 7.5 7.0 6.5 6.0 5.5 5.0 4.5 4.0 3.5 3.0 2.5 2.0 1.5 1.0 0.5 0.0

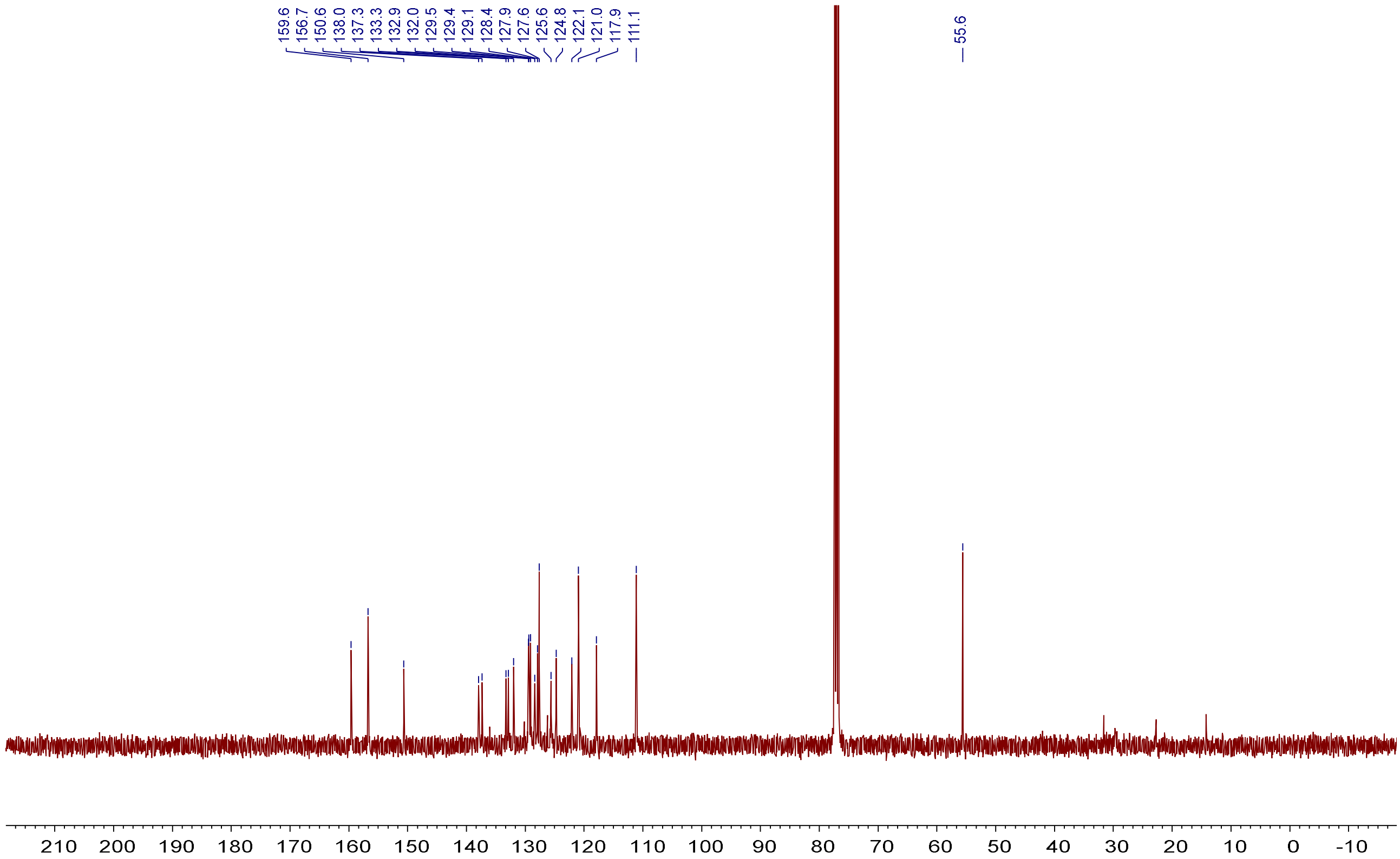
159.6
156.7
150.6
138.0
137.3
133.3
132.9
132.0
129.5
129.4
129.1
128.4
127.9
127.6
125.6
124.8
122.1
121.0
117.9
111.1
55.6
210 200 190 180 170 160 150 140 130 120 110 100 90 80 70 60 50 40 30 20 10 0 -10

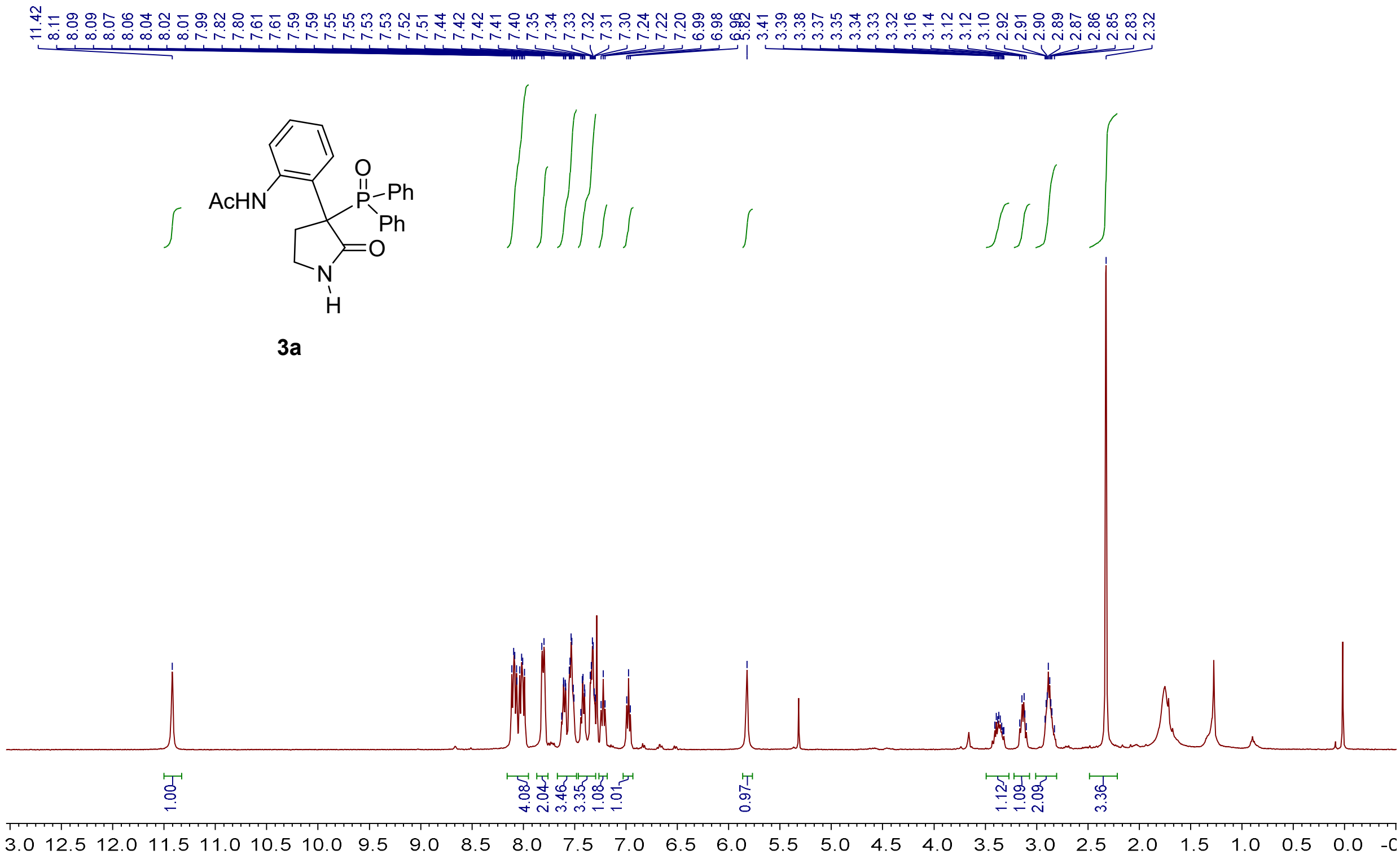
11.42 8.11 8.09 8.09 8.07 8.06 8.04 8.02 8.01 7.99 7.82 7.80 7.61 7.61 7.59 7.59 7.55 7.55 7.53 7.53 7.52 7.51 7.44 7.42 7.42 7.41 7.40 7.35 7.34 7.33 7.32 7.31 7.30 7.24 7.22 7.20 6.99 6.98 6.96 5.82 3.41 3.39 3.38 3.37 3.35 3.34 3.33 3.32 3.16 3.14 3.12 3.12 3.10 2.92 2.91 2.90 2.89 2.87 2.86 2.85 2.83 2.32
1.00 4.08 2.04 3.46 3.35 1.08 1.01 0.97 1.12 1.09 2.09 3.36
3.0 12.5 12.0 11.5 11.0 10.5 10.0 9.5 9.0 8.5 8.0 7.5 7.0 6.5 6.0 5.5 5.0 4.5 4.0 3.5 3.0 2.5 2.0 1.5 1.0 0.5 0.0
AcHN
O
P
Ph
Ph
O
N
H

3a

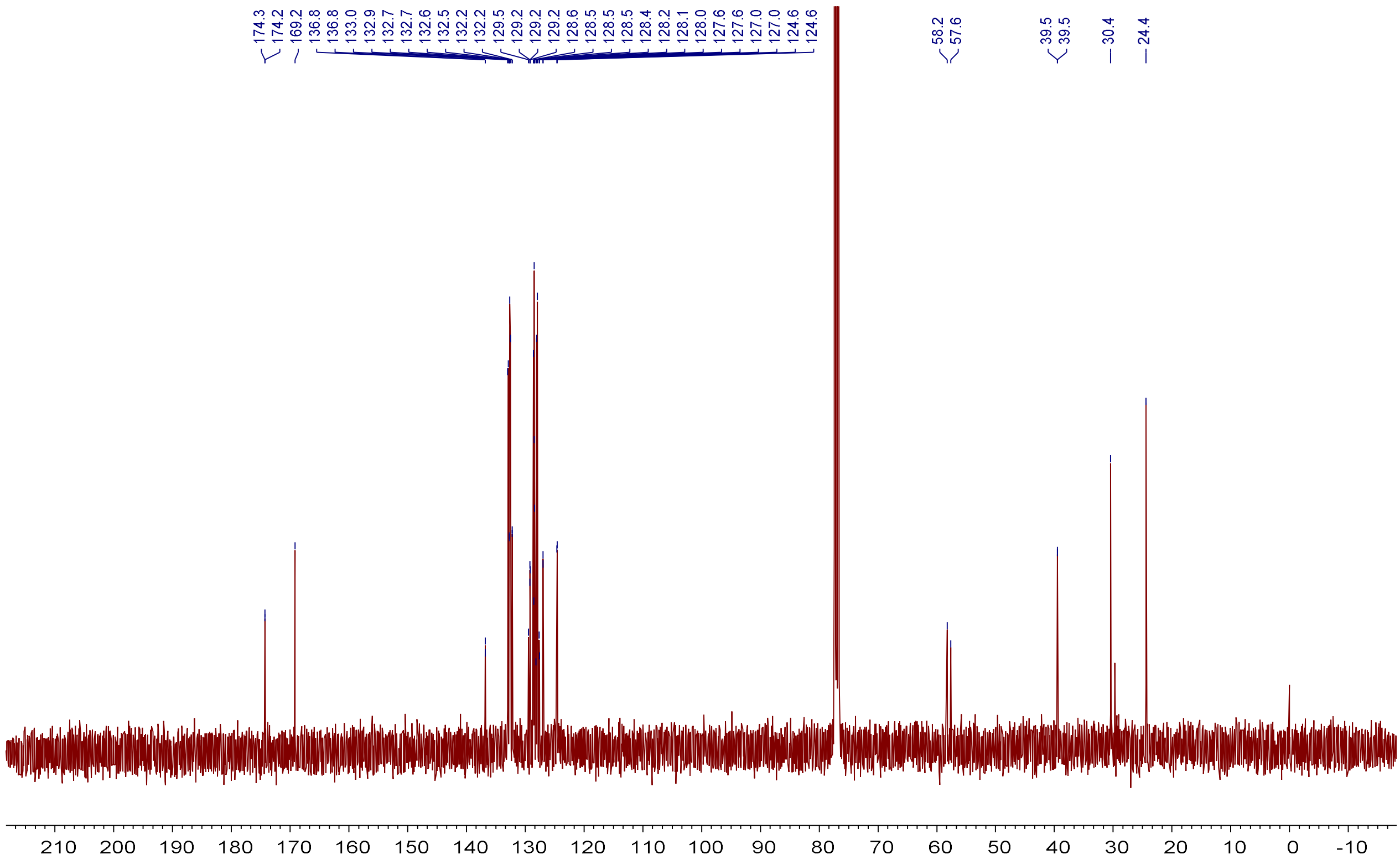
174.3 174.2 169.2 136.8 136.8 133.0 132.9 132.7 132.7 132.6 132.5 132.2 132.2 129.5 129.2 129.2 129.2 128.6 128.5 128.5 128.4 128.2 128.1 128.0 127.6 127.6 127.0 127.0 124.6 124.6 58.2 57.6 39.5 39.5 30.4 24.4
210 200 190 180 170 160 150 140 130 120 110 100 90 80 70 60 50 40 30 20 10 0 -10

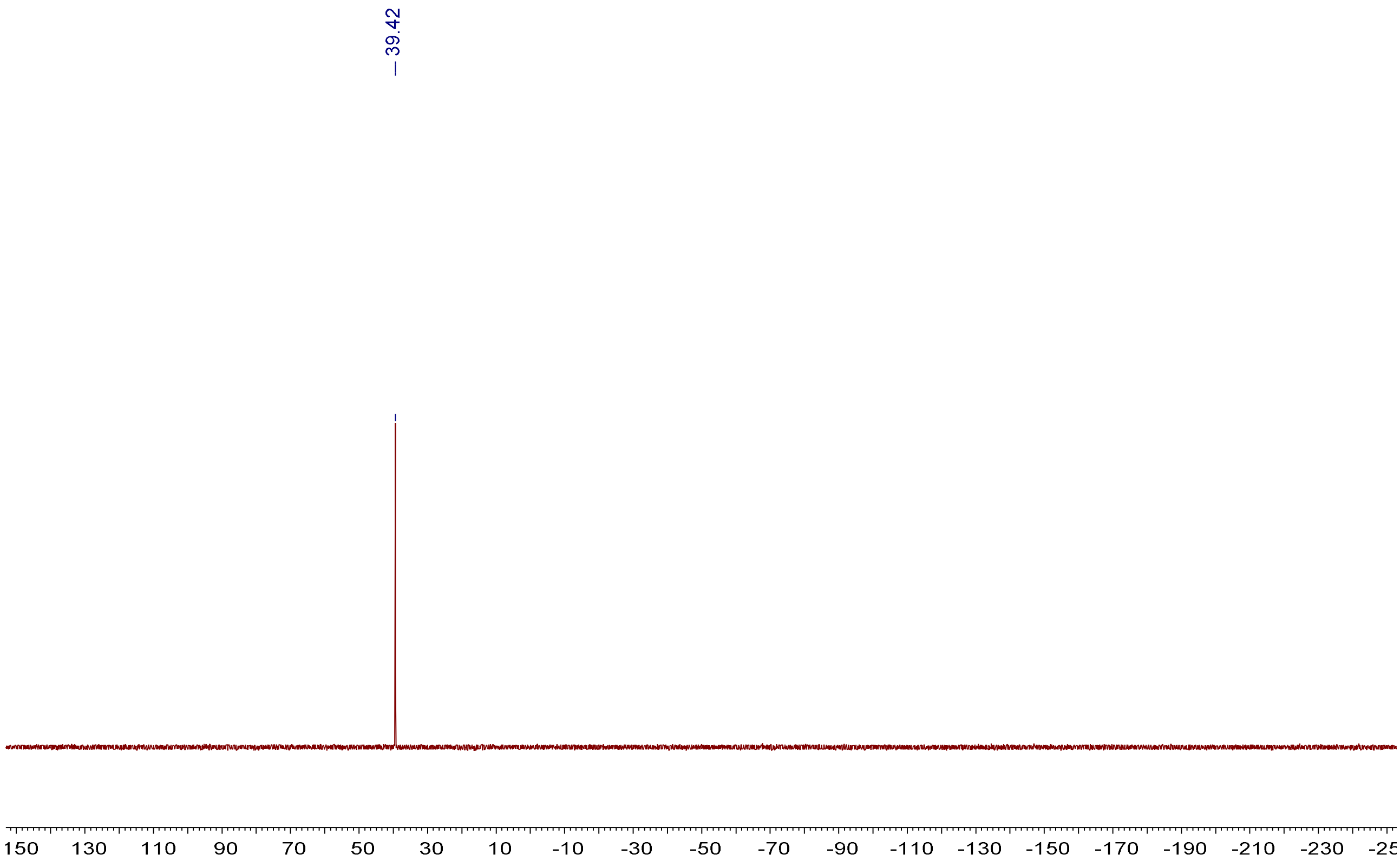
39.42
150 130 110 90 70 50 30 10 -10 -30 -50 -70 -90 -110 -130 -150 -170 -190 -210 -230 -25

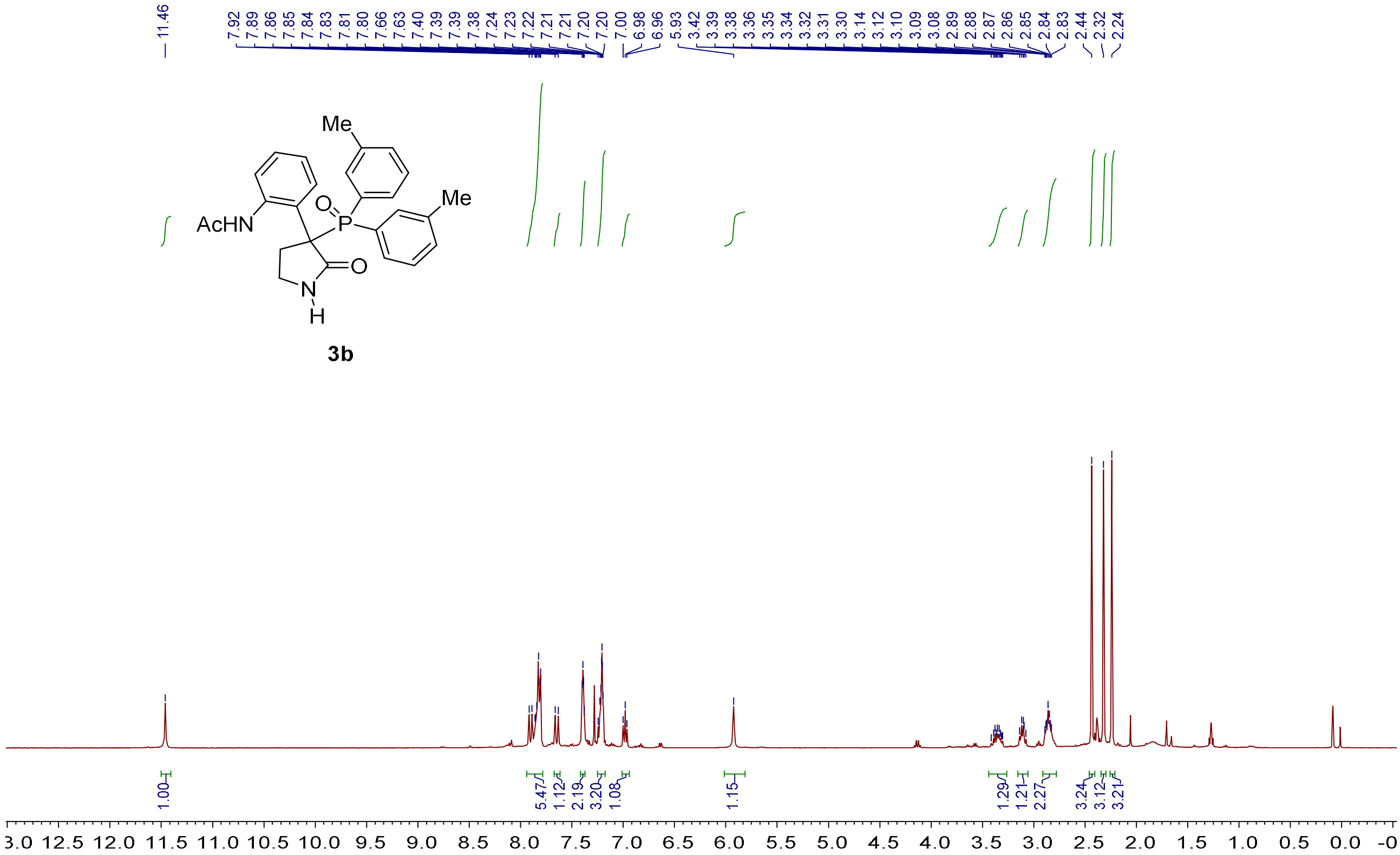

3b

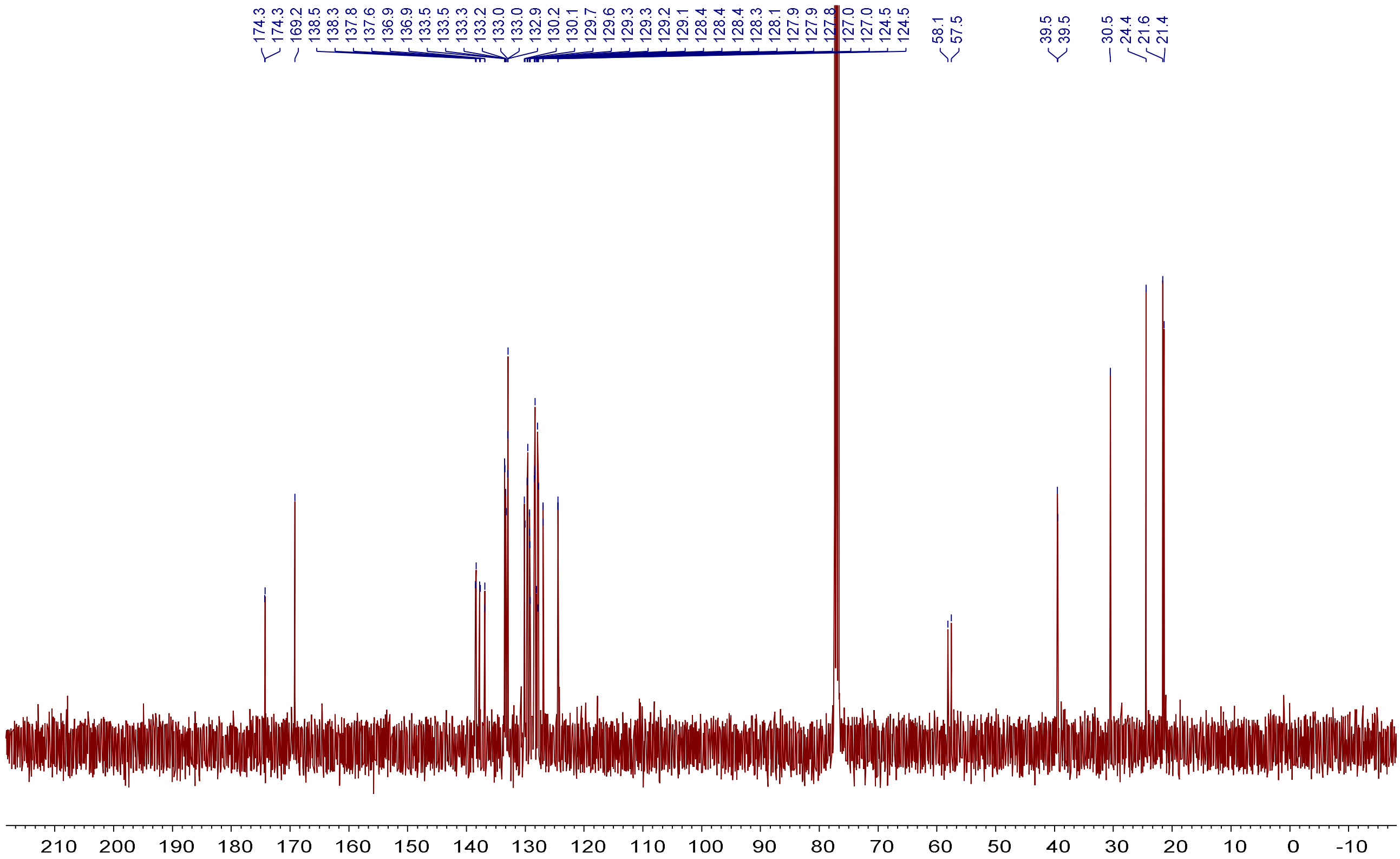

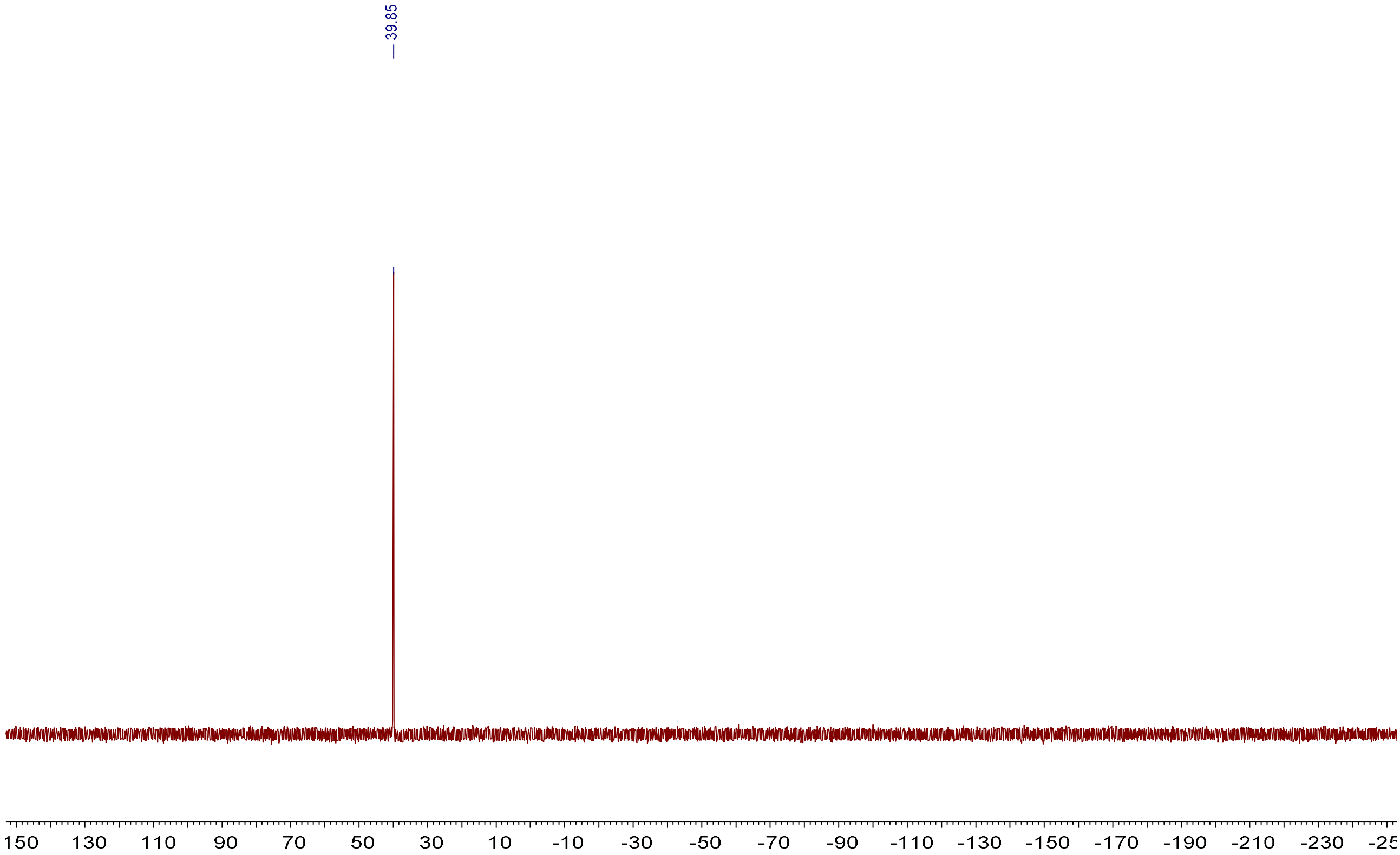

39.85
150 130 110 90 70 50 30 10 -10 -30 -50 -70 -90 -110 -130 -150 -170 -190 -210 -230

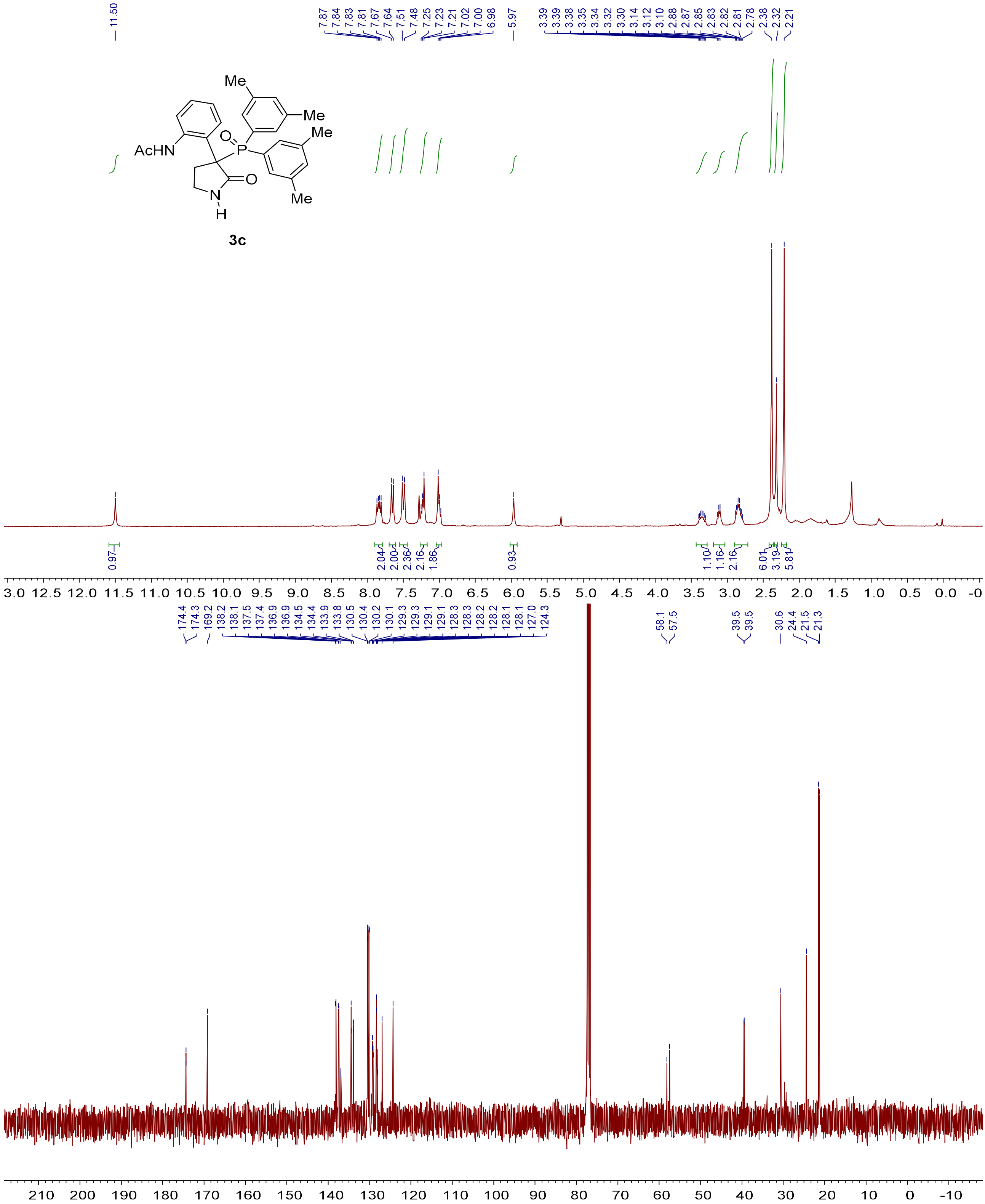

11.50
7.87 7.84 7.83 7.81 7.67 7.64 7.51 7.48 7.25 7.23 7.21 7.02 7.00 6.98
5.97
3.39 3.39 3.38 3.35 3.34 3.32 3.30 3.14 3.12 3.10 2.88 2.87 2.85 2.83 2.82 2.81 2.78 2.38 2.32 2.21
Me
Me
Me
Me
AcHN
O
P
O
N
H
3c
0.97
2.04 2.00 2.36 2.16 1.86
0.93
1.10 1.16 2.16
6.01 3.19 5.81
3.0 12.5 12.0 11.5 11.0 10.5 10.0 9.5 9.0 8.5 8.0 7.5 7.0 6.5 6.0 5.5 5.0 4.5 4.0 3.5 3.0 2.5 2.0 1.5 1.0 0.5 0.0 -0
174.4 174.3 169.2 138.2 138.1 137.5 137.4 136.9 136.9 134.5 134.4 133.9 133.8 130.5 130.4 130.2 130.1 129.3 129.3 129.1 129.1 128.3 128.3 128.2 128.2 128.1 128.1 127.0 124.3
58.1 57.5
39.5 39.5
30.6 24.4 21.5 21.3
210 200 190 180 170 160 150 140 130 120 110 100 90 80 70 60 50 40 30 20 10 0 -10

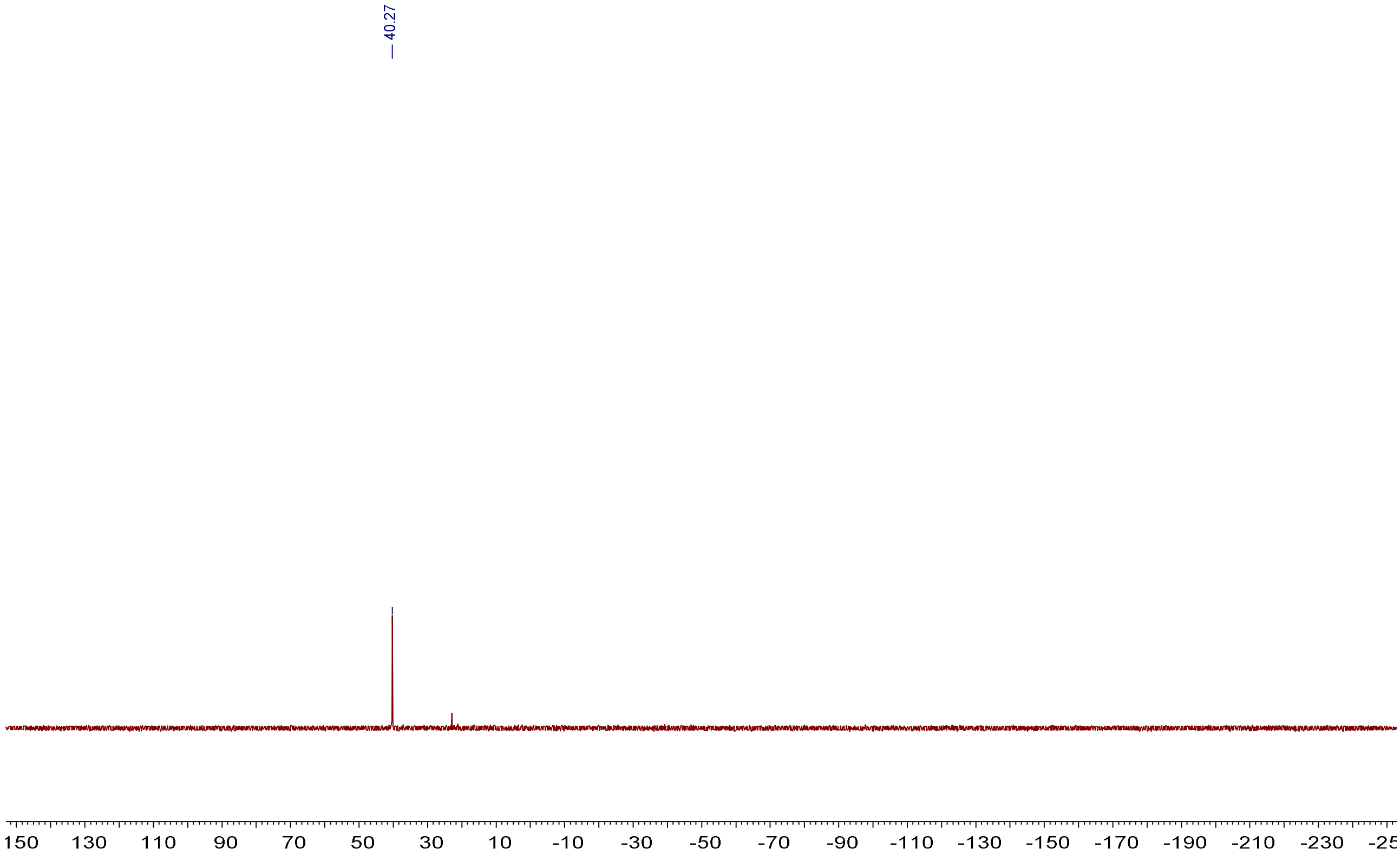

40.27
150
130
110
90
70
50
30
10
-10
-30
-50
-70
-90
-110
-130
-150
-170
-190
-210
-230

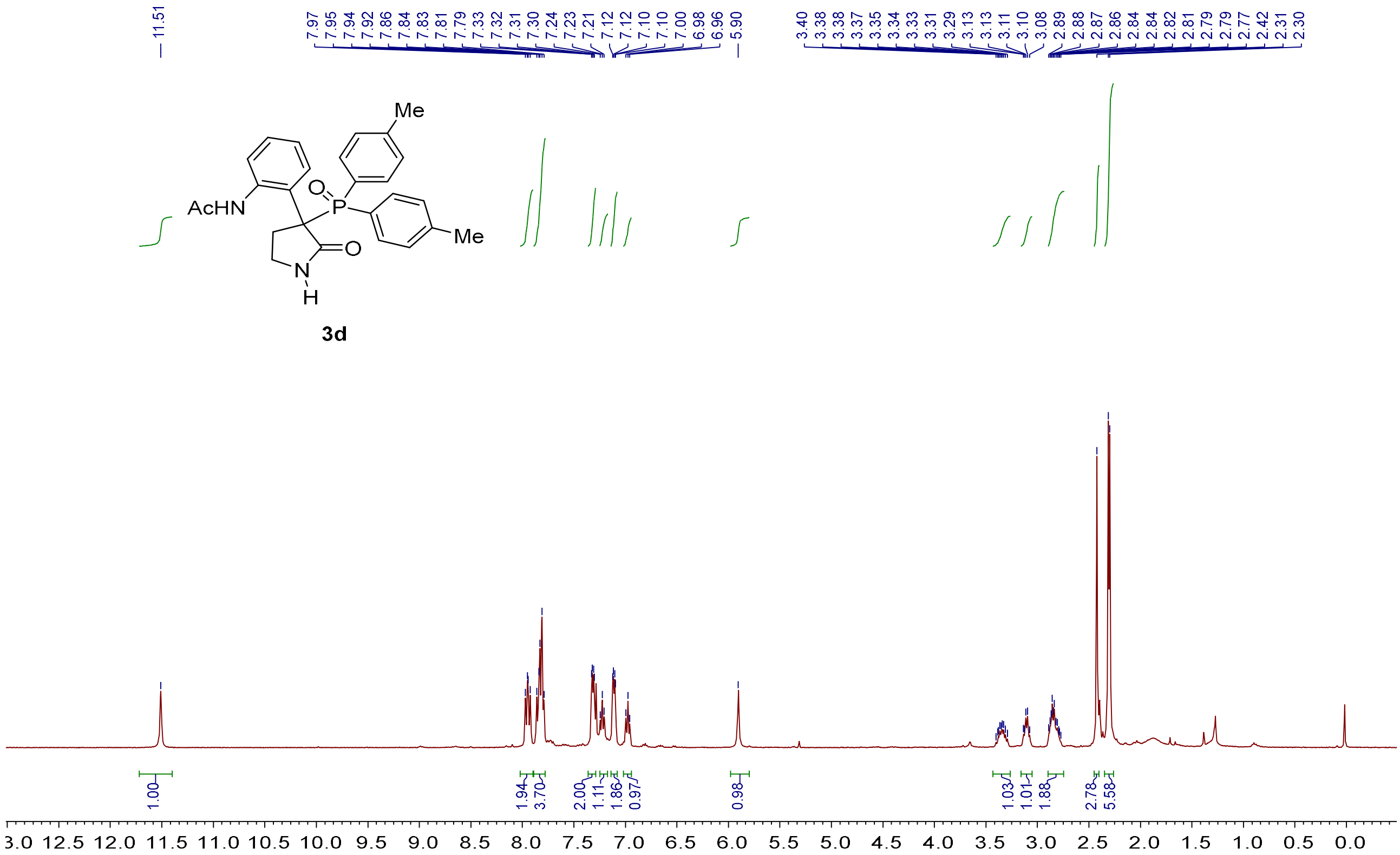

11.51
7.97 7.95 7.94 7.92 7.86 7.84 7.83 7.81 7.79 7.33 7.32 7.31 7.30 7.24 7.23 7.21 7.12 7.12 7.10 7.10 7.00 6.98 6.96
5.90
3.40 3.38 3.38 3.37 3.35 3.34 3.33 3.31 3.29 3.13 3.13 3.11 3.10 3.08 2.89 2.88 2.87 2.86 2.84 2.84 2.82 2.81 2.79 2.77 2.42 2.31 2.30
Me
AcHN
O
P
Me
N
H
O
3d
1.00
1.94
3.70
2.00
1.11
1.86
0.97
0.98
1.03
1.01
1.88
2.78
5.58
13.0 12.5 12.0 11.5 11.0 10.5 10.0 9.5 9.0 8.5 8.0 7.5 7.0 6.5 6.0 5.5 5.0 4.5 4.0 3.5 3.0 2.5 2.0 1.5 1.0 0.5 0.0

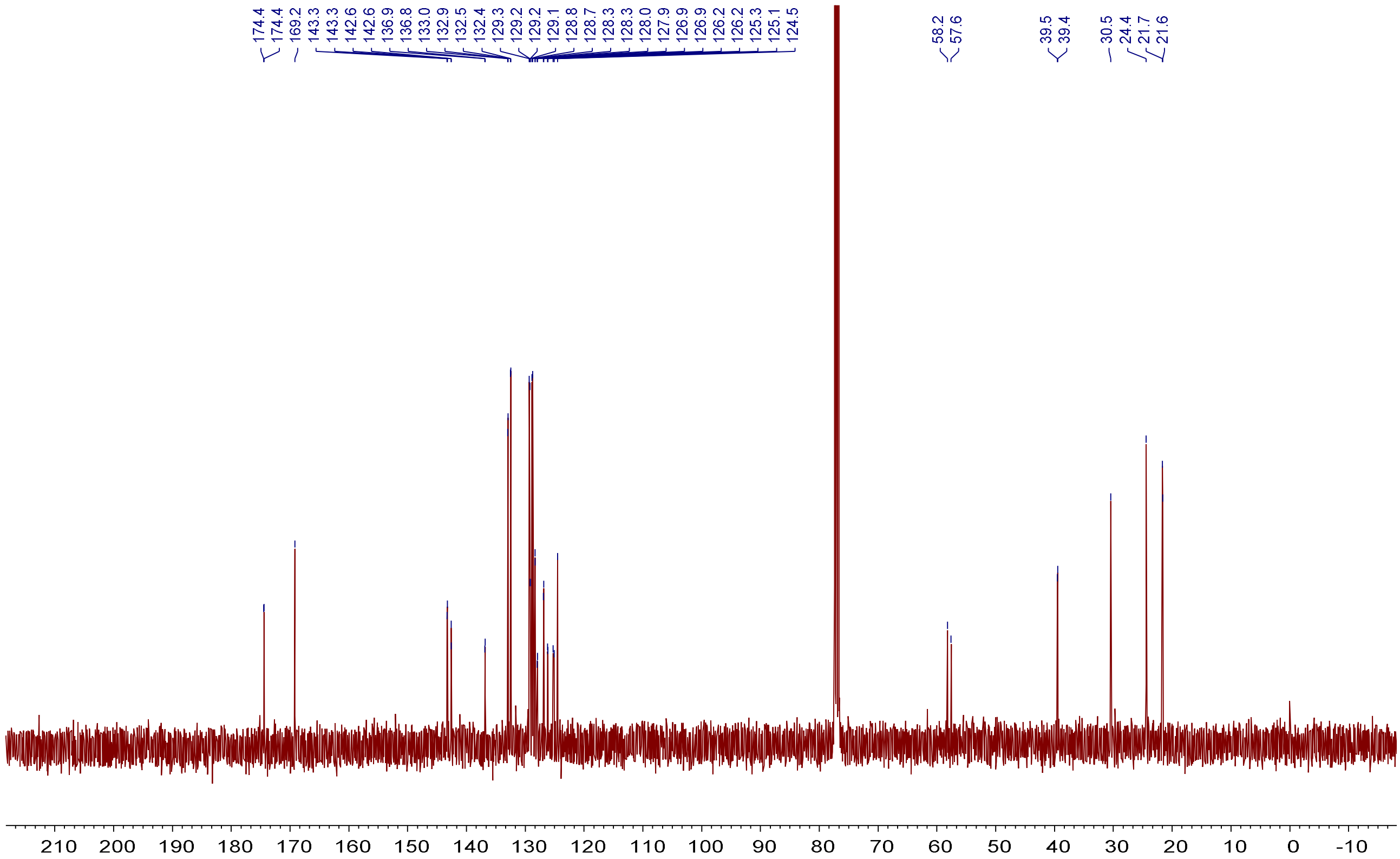

174.4 174.4 169.2
143.3 142.6 142.6 136.9 136.8 133.0 132.9 132.5 132.4 129.3 129.2 129.2 129.1 128.8 128.7 128.3 128.3 128.0 127.9 126.9 126.9 126.2 126.2 125.3 125.1 124.5
58.2 57.6
39.5 39.4
30.5 24.4 21.7 21.6
210 200 190 180 170 160 150 140 130 120 110 100 90 80 70 60 50 40 30 20 10 0 -10

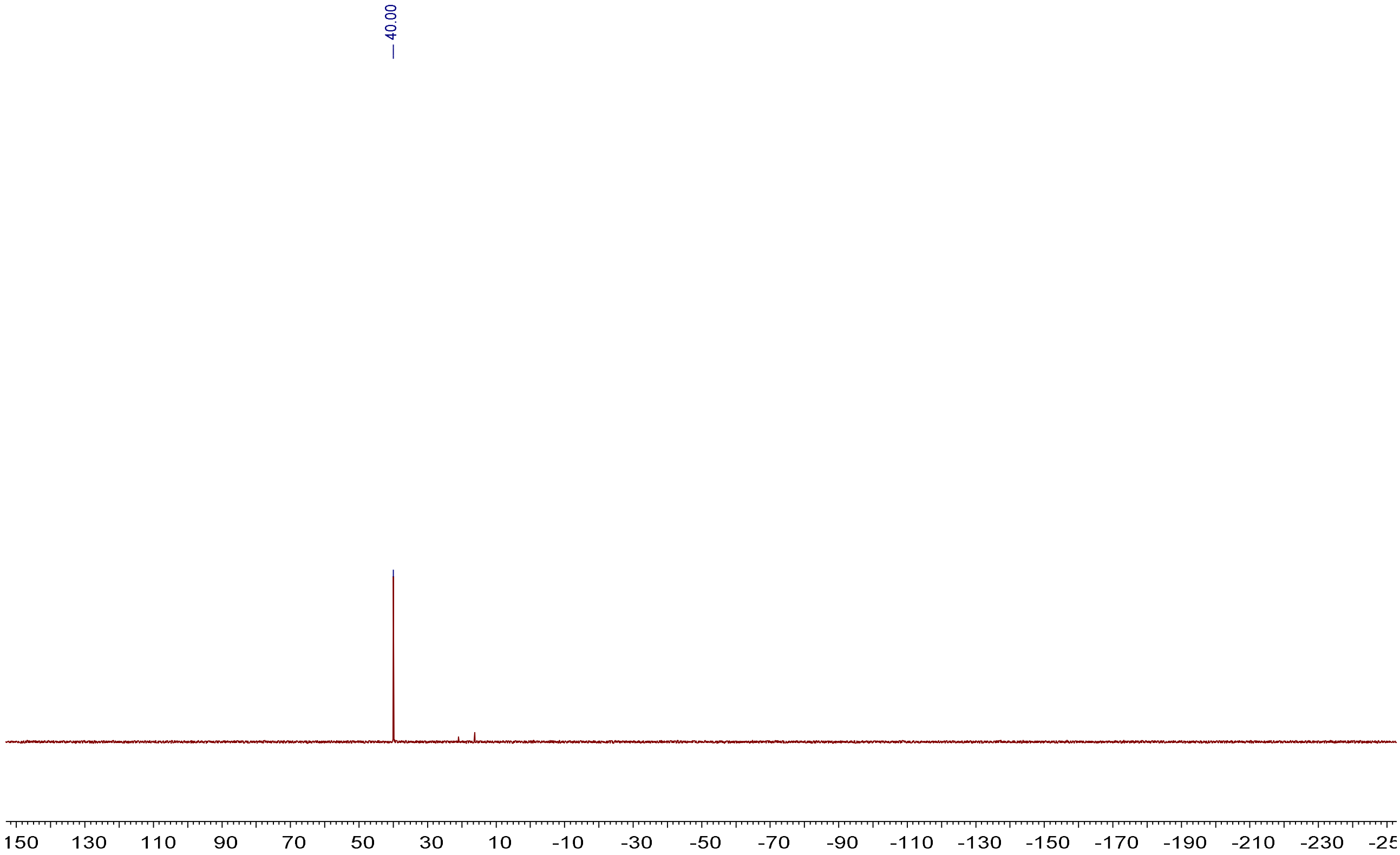

40.00
150 130 110 90 70 50 30 10 -10 -30 -50 -70 -90 -110 -130 -150 -170 -190 -210 -230 -25

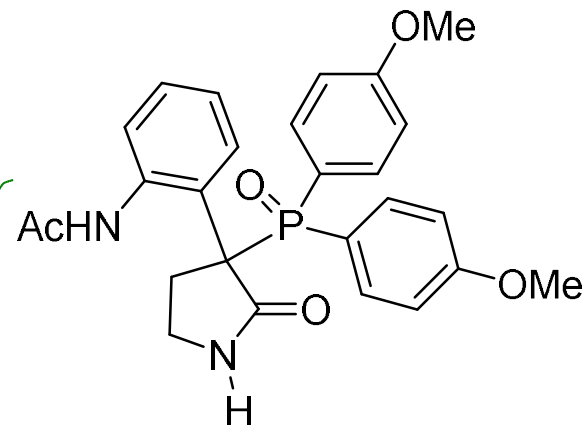
OMe
AcHN
O=P
OMe
O
N
H

3e

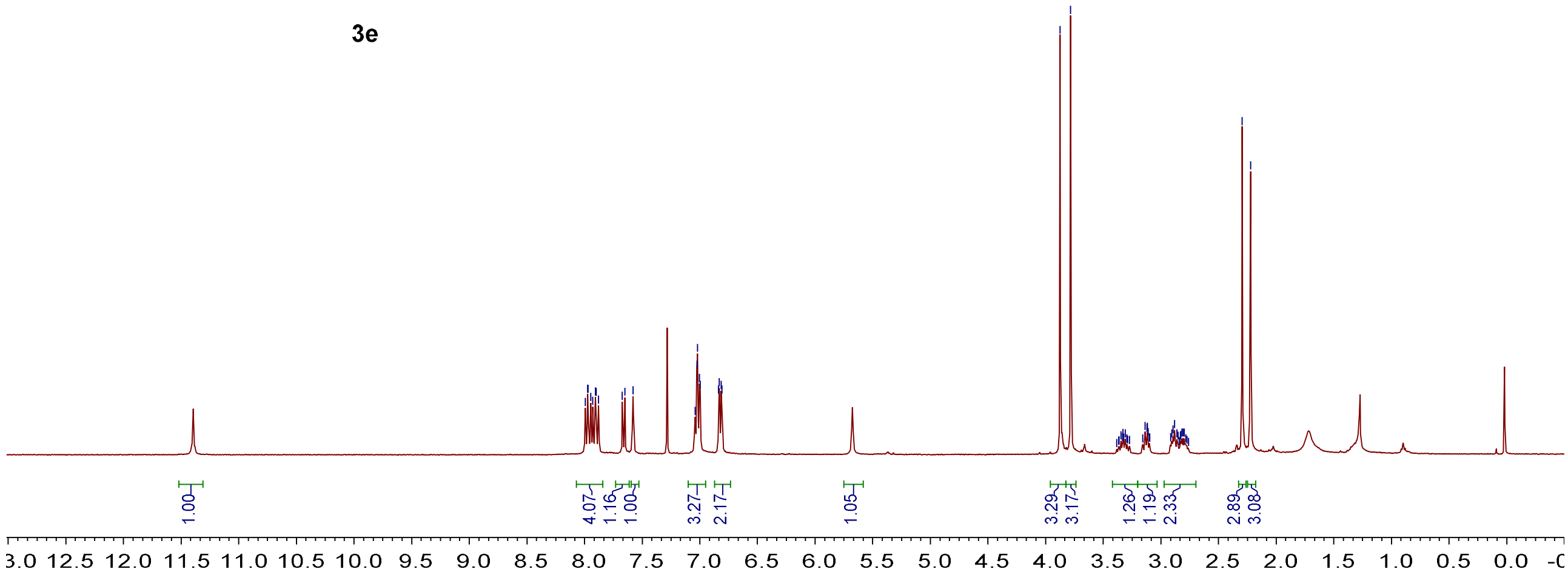
7.994
7.973
7.968
7.946
7.928
7.906
7.900
7.878
7.672
7.651
7.580
7.041
7.025
7.020
7.003
6.997
6.837
6.830
6.814
6.808
3.874
3.783
3.346
3.334
3.329
3.322
3.310
3.136
3.120
3.116
3.112
3.101
2.917
2.905
2.894
2.883
2.860
2.852
2.832
2.828
2.819
2.808
2.796
2.295

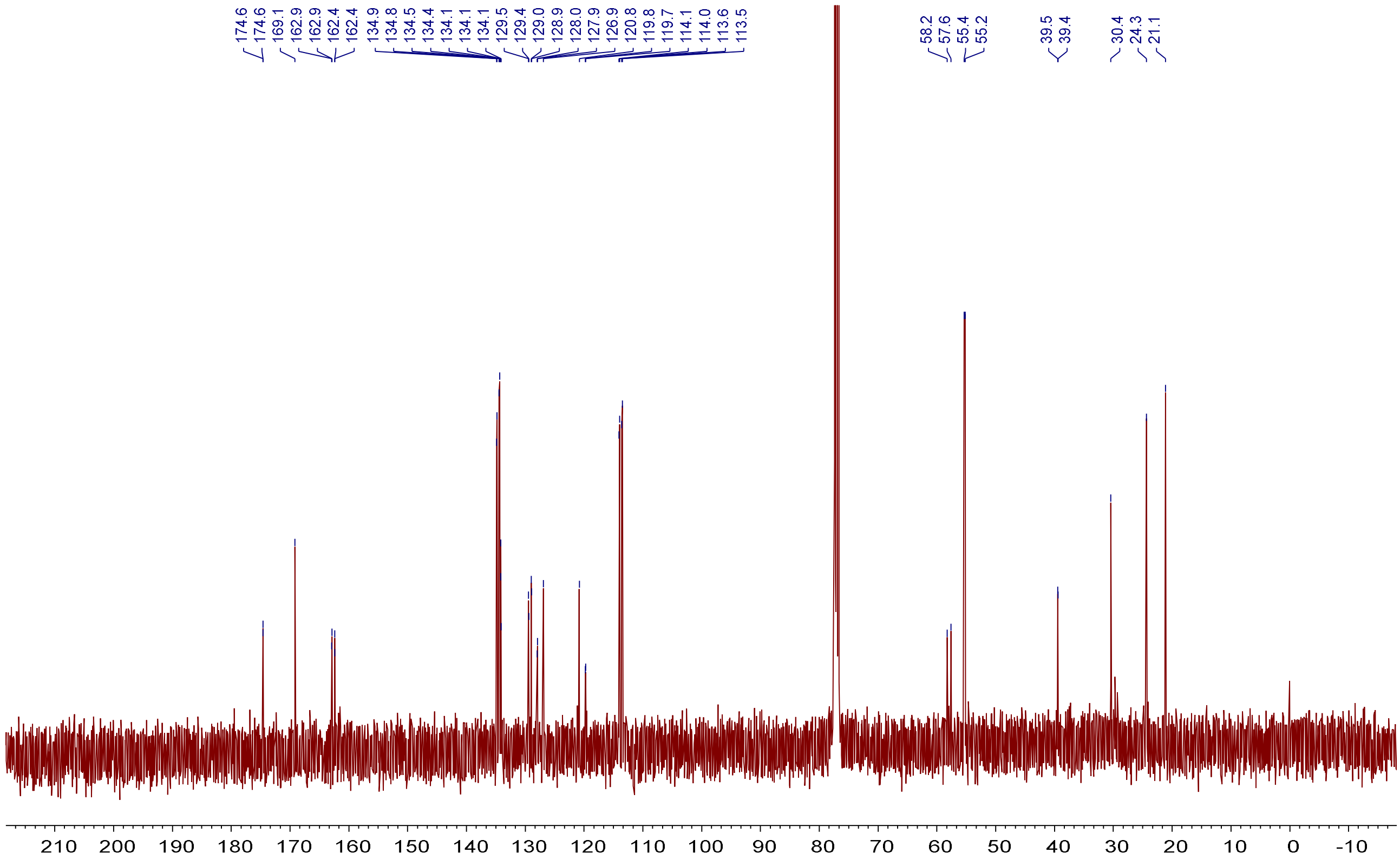
174.6
174.6
169.1
162.9
162.9
162.4
162.4
134.9
134.8
134.5
134.4
134.1
134.1
134.1
134.1
129.5
129.4
129.0
128.9
128.0
127.9
126.9
120.8
119.8
119.7
114.1
114.0
113.6
113.5
58.2
57.6
55.4
55.2
39.5
39.4
30.4
24.3
21.1

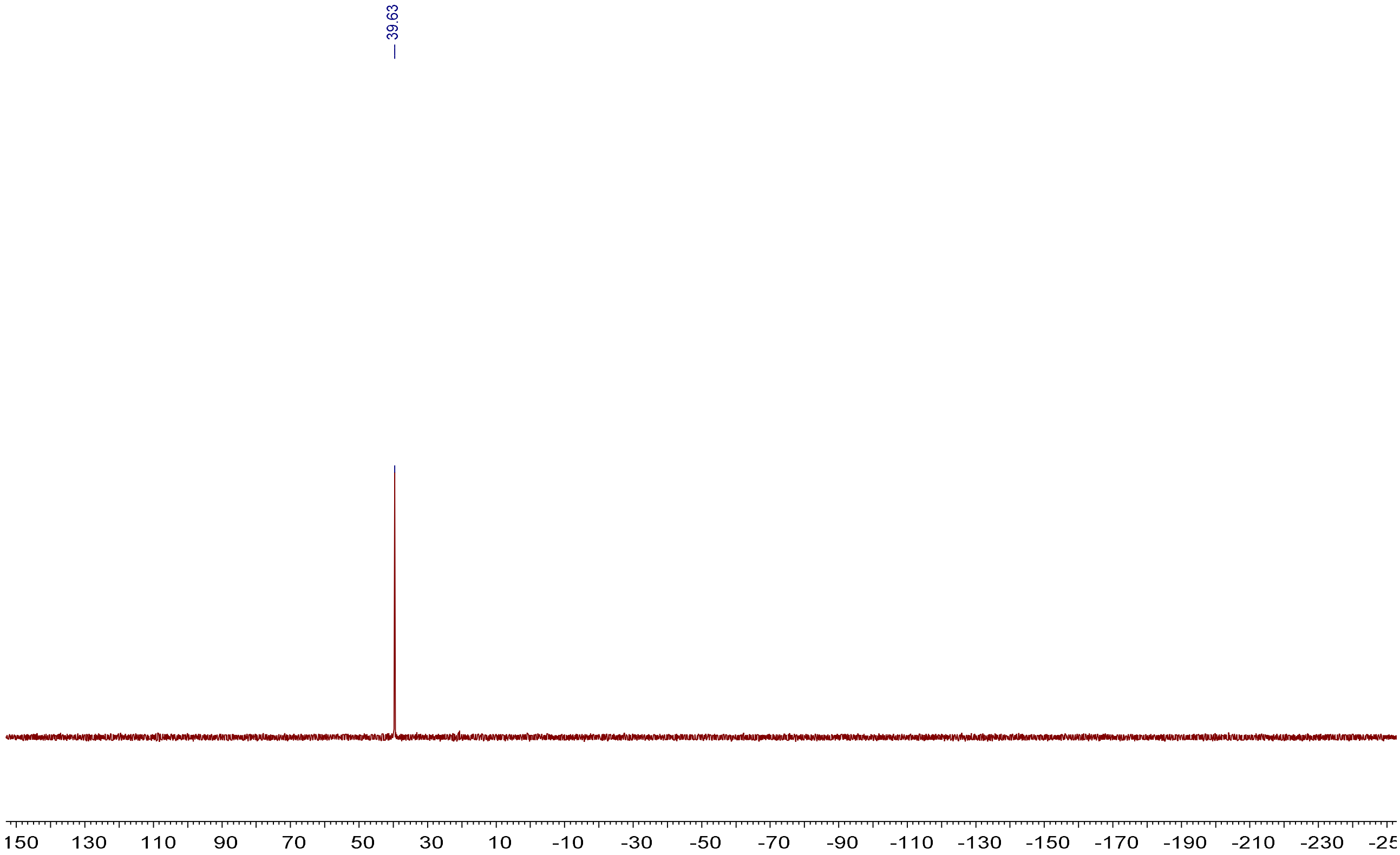
39.63
150 130 110 90 70 50 30 10 -10 -30 -50 -70 -90 -110 -130 -150 -170 -190 -210 -230

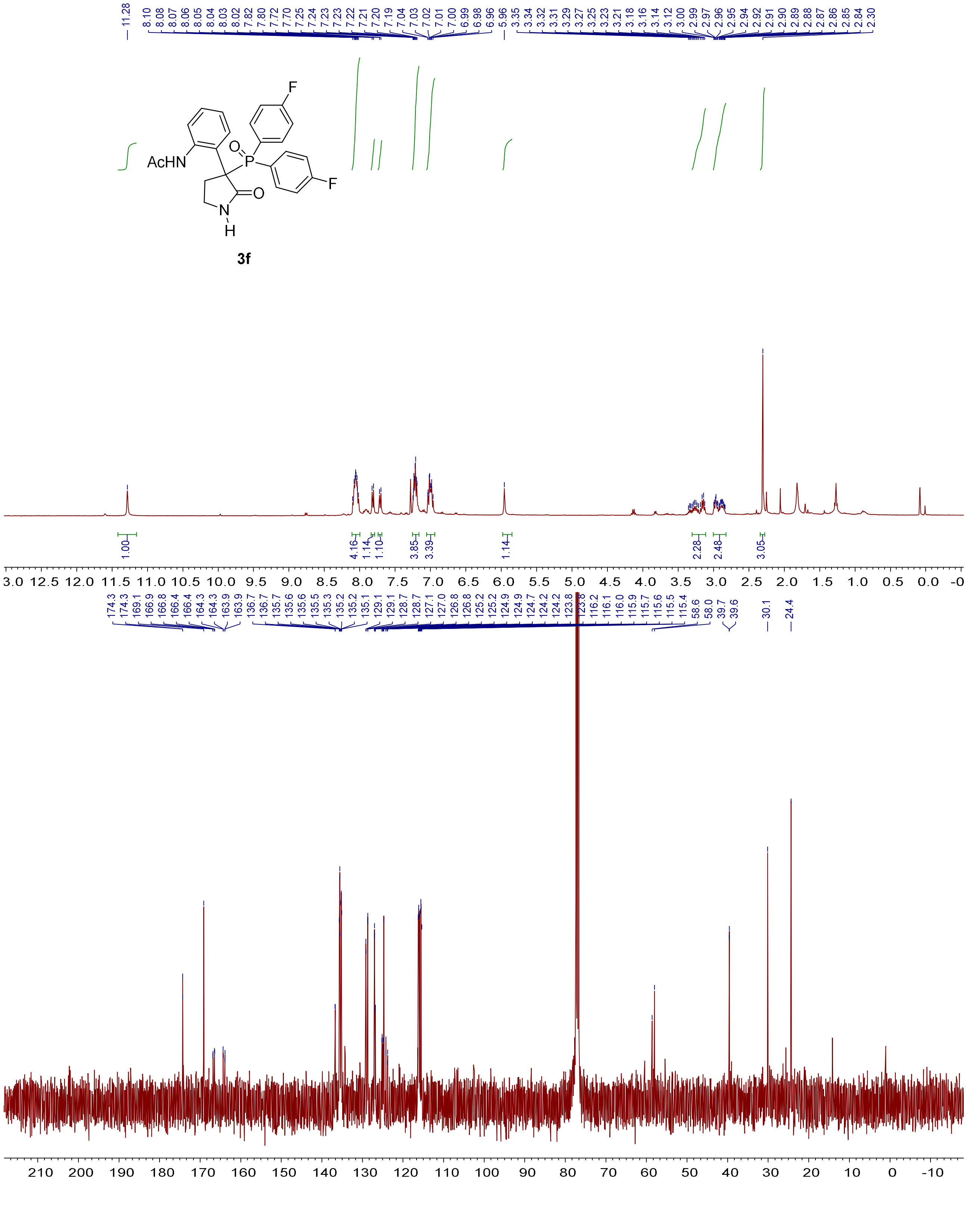
AcHN
F
F
O
P
O
N
H
3f

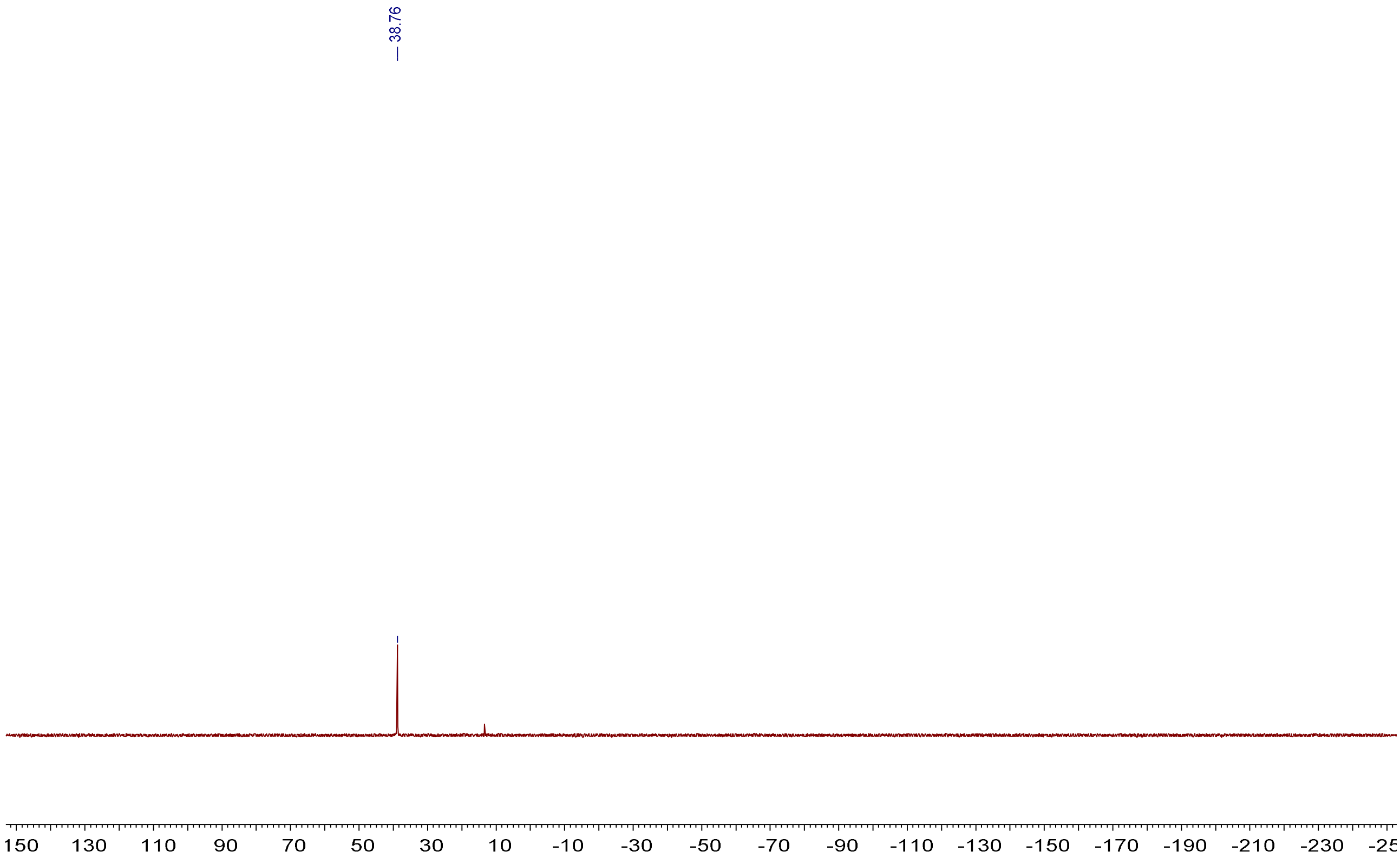
38.76
150
130
110
90
70
50
30
10
-10
-30
-50
-70
-90
-110
-130
-150
-170
-190
-210
-230

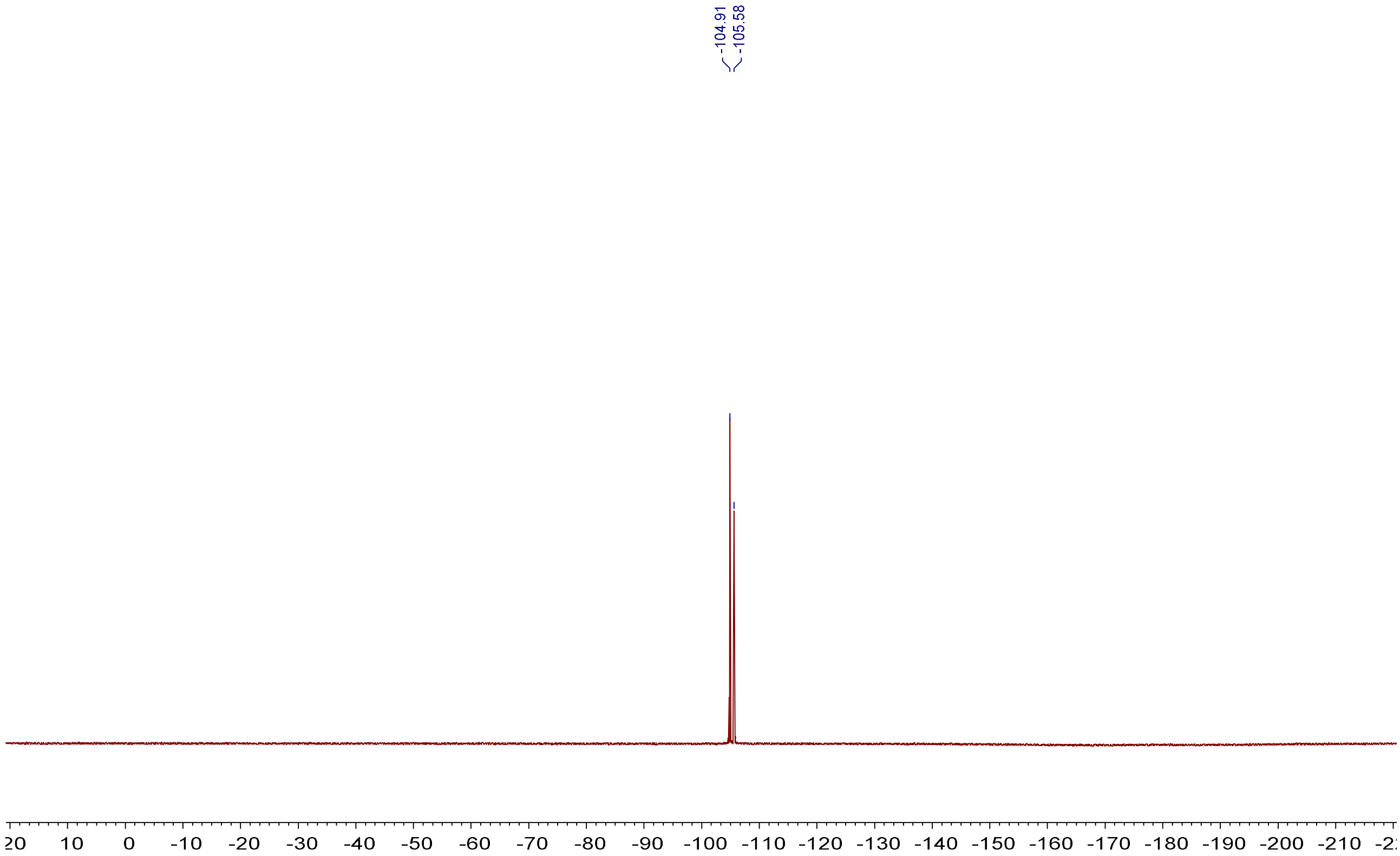
-104.91
-105.58
20
10
0
-10
-20
-30
-40
-50
-60
-70
-80
-90
-100
-110
-120
-130
-140
-150
-160
-170
-180
-190
-200
-210

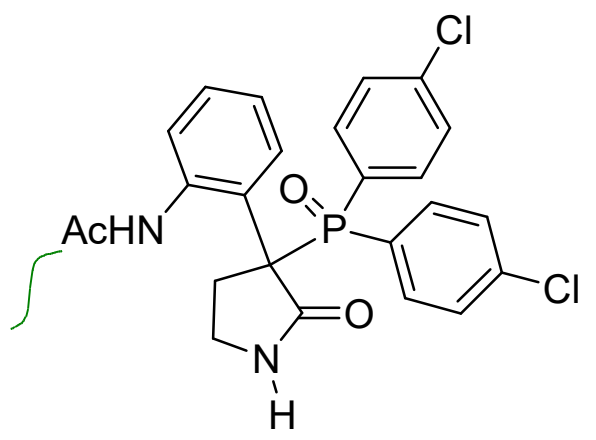
Cl
AcHN
O
P
Cl
O
N
H

3g

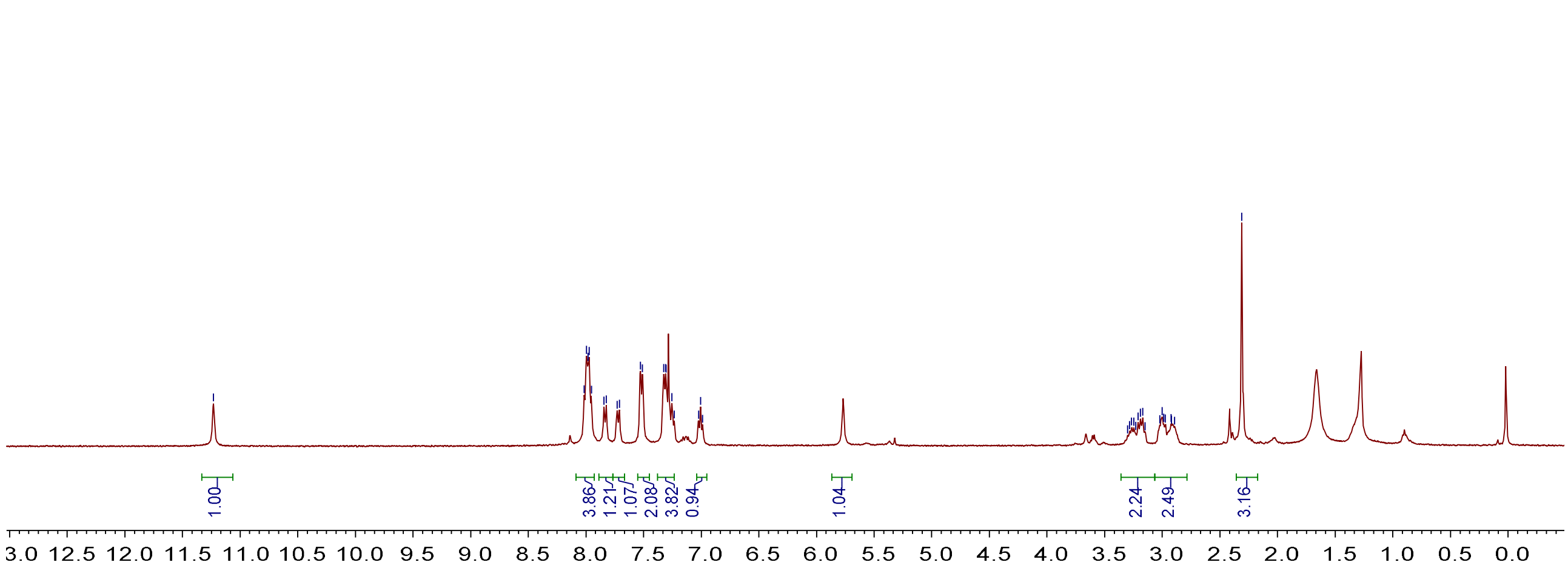
11.23
8.02 7.99 7.98 7.97 7.95 7.84 7.82 7.73 7.71 7.53 7.51 7.33 7.31 7.30 7.25 7.23 7.02 7.01 6.99
3.30 3.28 3.27 3.25 3.23 3.21 3.19 3.17 3.15 3.02 3.00 2.99 2.97 2.93 2.92 2.89
2.31
1.00 3.86 1.21 1.07 2.08 3.82 0.94 1.04 2.24 2.49 3.16

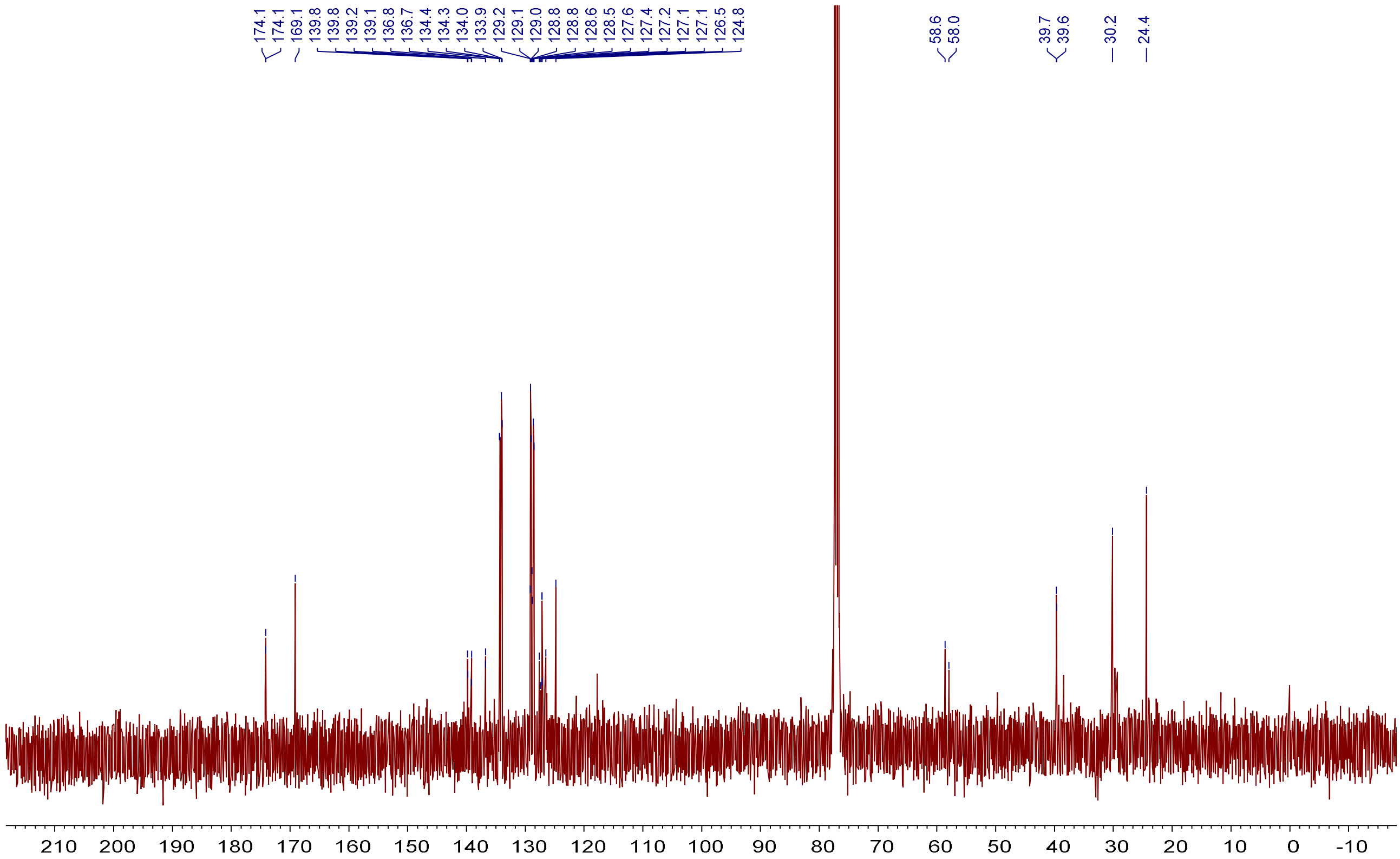
174.1 174.1 169.1 139.8 139.2 139.1 136.8 136.7 134.4 134.3 134.0 133.9 129.2 129.1 129.0 128.8 128.8 128.6 128.5 127.6 127.4 127.2 127.1 127.1 126.5 124.8
58.6 58.0
39.7 39.6
30.2
24.4

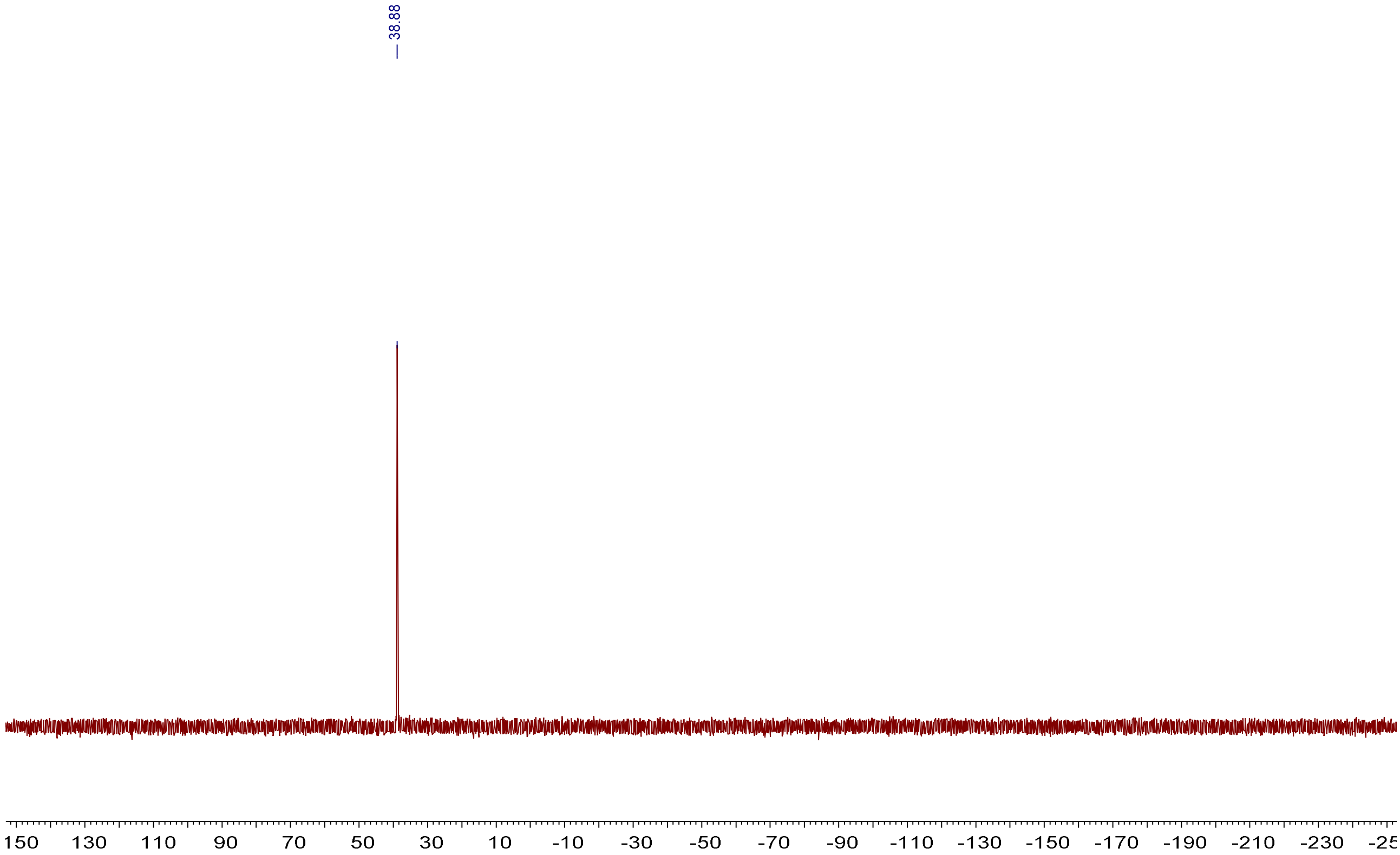

38.88
150 130 110 90 70 50 30 10 -10 -30 -50 -70 -90 -110 -130 -150 -170 -190 -210 -230 -25

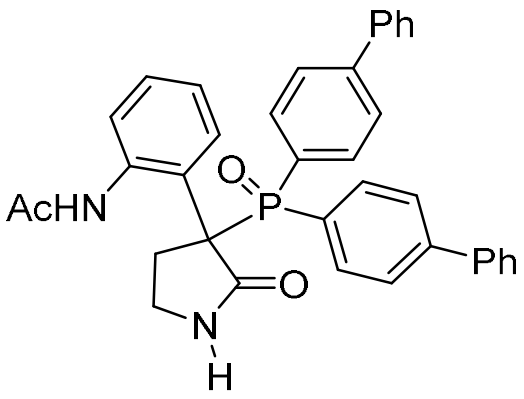

Ph
AcHN
O
P
Ph
O
N
H

3h

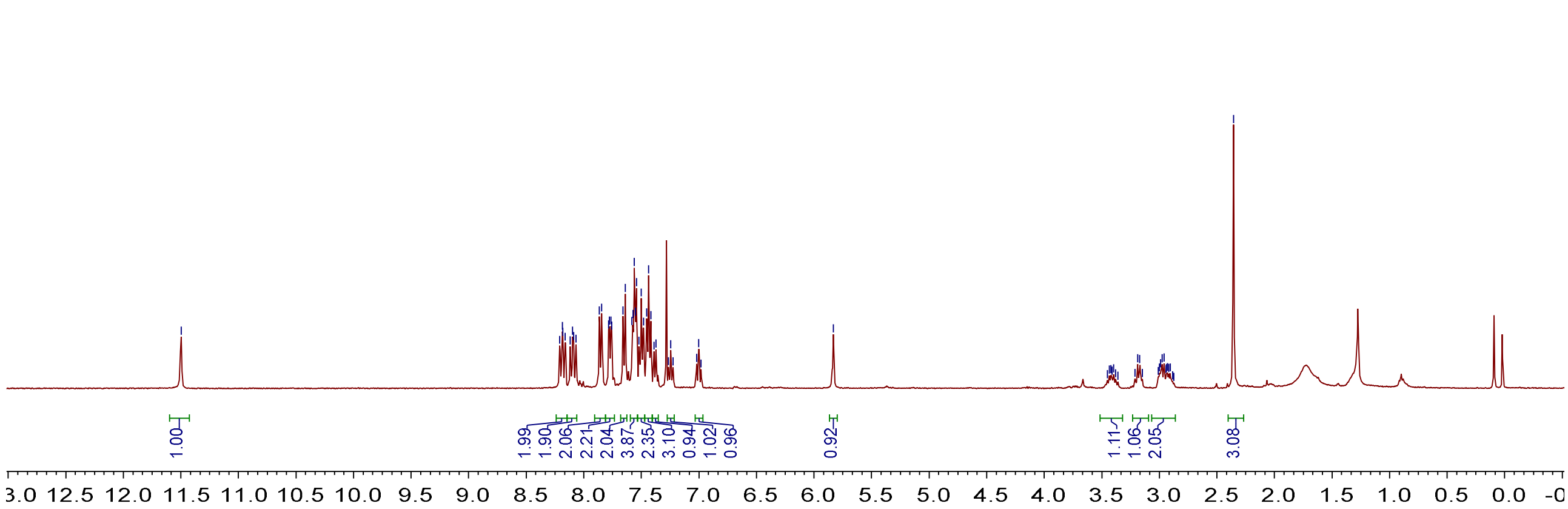

11.50 8.21 8.19 8.18 8.16 8.12 8.10 8.09 8.07 7.86 7.84 7.78 7.78 7.76 7.66 7.64 7.58 7.57 7.56 7.55 7.54 7.52 7.50 7.48 7.45 7.44 7.42 7.39 7.37 7.26 7.24 7.23 7.02 7.00 6.98 5.83 3.45 3.43 3.42 3.41 3.40 3.38 3.36 3.21 3.19 3.17 3.15 3.01 3.00 2.99 2.98 2.96 2.94 2.94 2.93 2.92 2.91 2.88 2.87 2.36
1.00
1.99 1.90 2.06 2.21 2.04 3.87 2.35 3.10 0.94 1.02 0.96
0.92
1.11 1.06 2.05
3.08
3.0 12.5 12.0 11.5 11.0 10.5 10.0 9.5 9.0 8.5 8.0 7.5 7.0 6.5 6.0 5.5 5.0 4.5 4.0 3.5 3.0 2.5 2.0 1.5 1.0 0.5 0.0 -0

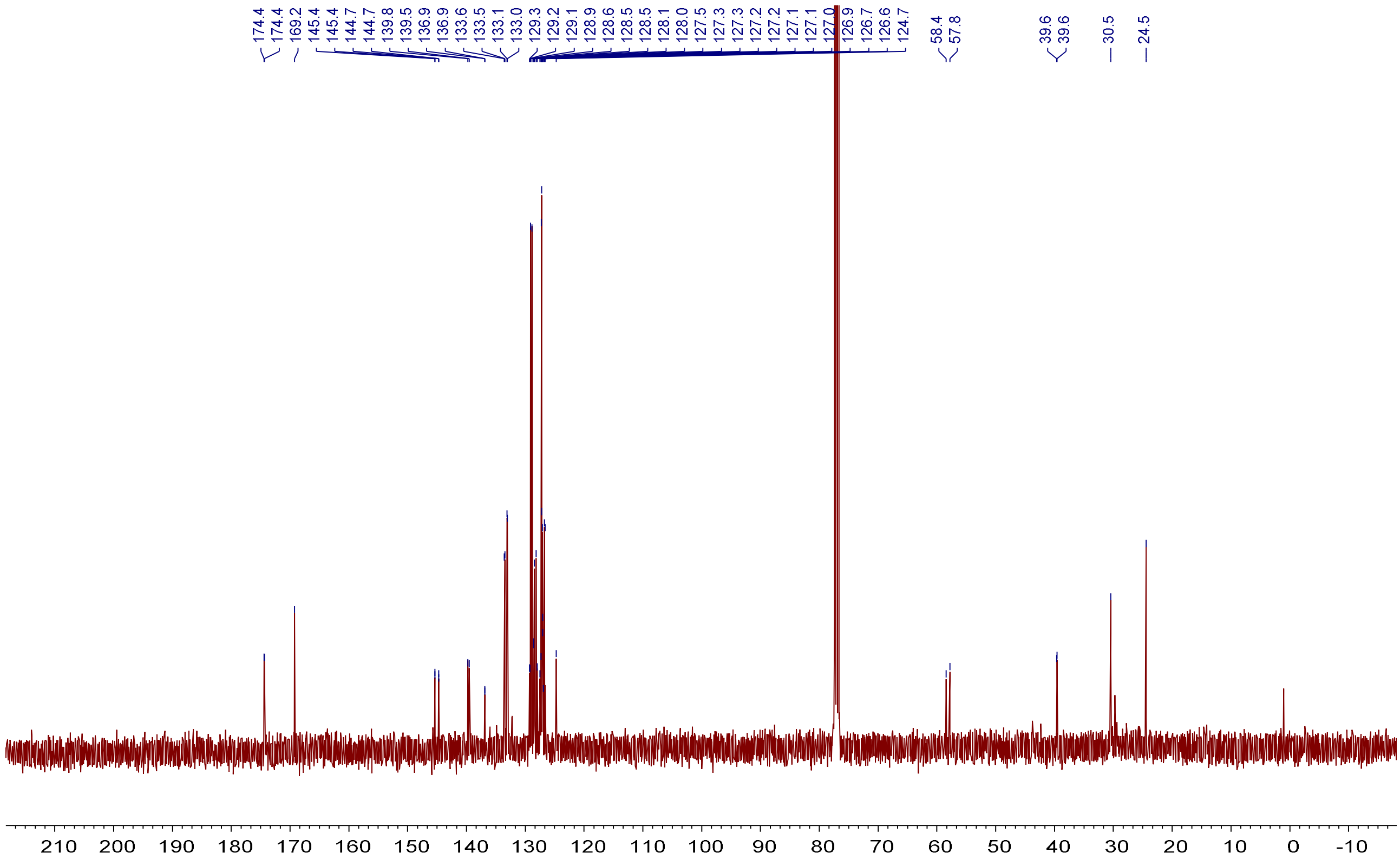

174.4 174.4 169.2 145.4 145.4 144.7 144.7 139.8 139.5 136.9 136.9 133.6 133.5 133.1 133.0 129.3 129.2 129.1 128.9 128.6 128.5 128.5 128.1 128.0 127.5 127.3 127.2 127.2 127.1 127.1 127.0 126.9 126.7 126.6 124.7
58.4 57.8
39.6 39.6
30.5
24.5
210 200 190 180 170 160 150 140 130 120 110 100 90 80 70 60 50 40 30 20 10 0 -10

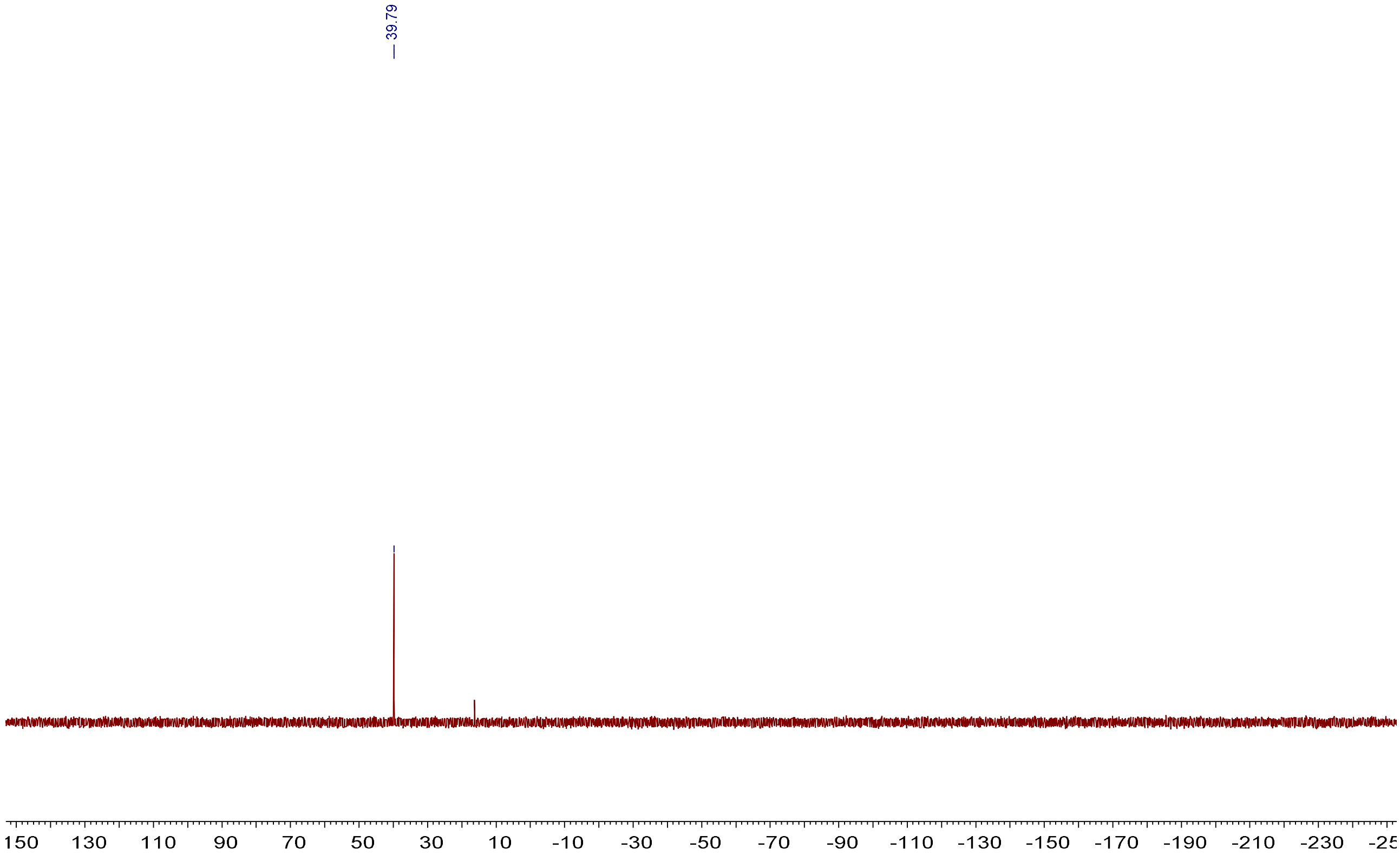
39.79
150 130 110 90 70 50 30 10 -10 -30 -50 -70 -90 -110 -130 -150 -170 -190 -210 -230 -25

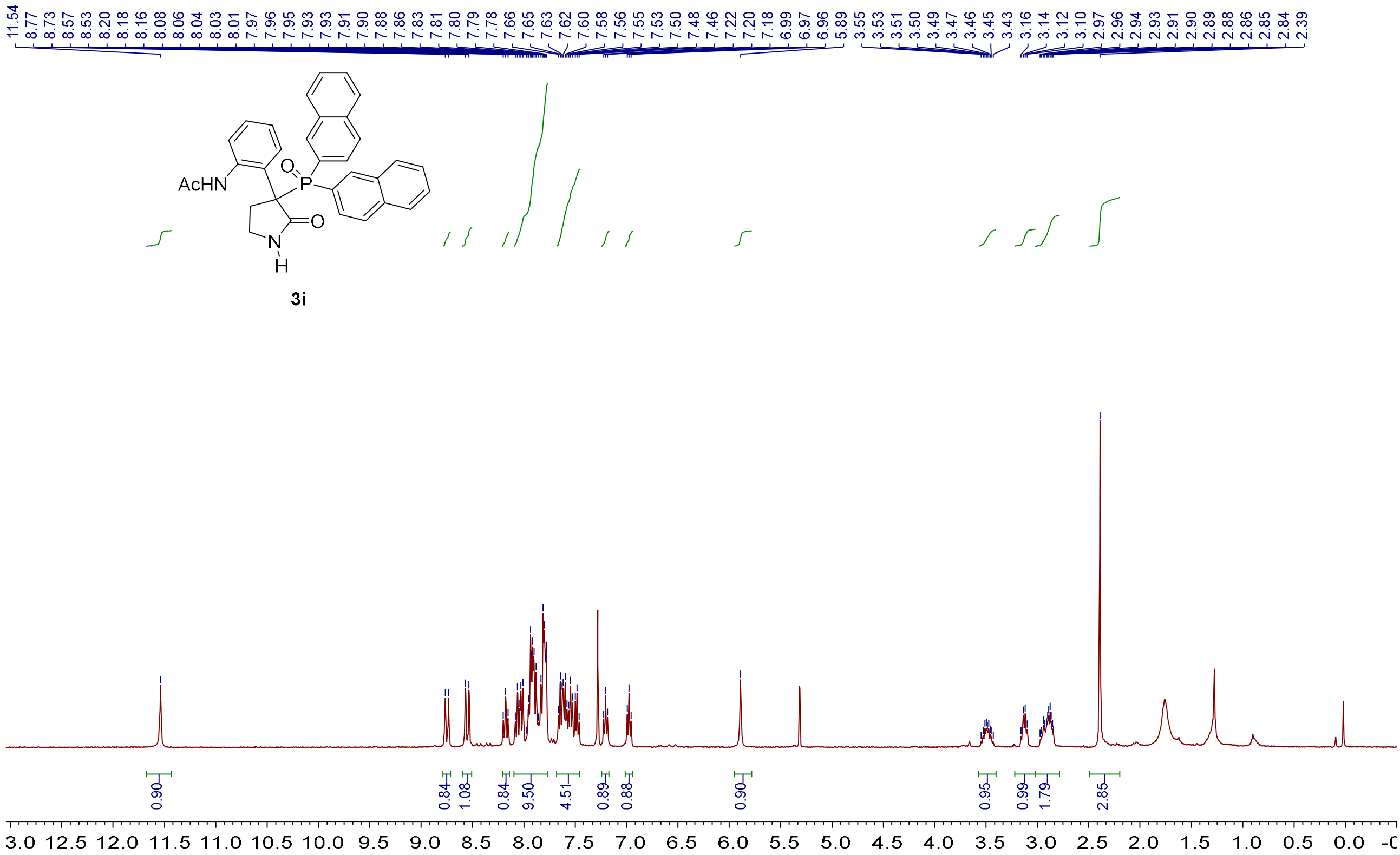
11.54 8.77 8.73 8.57 8.53 8.20 8.18 8.16 8.08 8.06 8.04 8.03 8.01 7.97 7.96 7.95 7.93 7.93 7.91 7.90 7.88 7.86 7.83 7.81 7.80 7.79 7.78 7.66 7.65 7.63 7.62 7.60 7.58 7.56 7.55 7.53 7.50 7.48 7.46 7.22 7.20 7.18 6.99 6.97 6.96 5.89 3.55 3.53 3.51 3.50 3.49 3.47 3.46 3.45 3.43 3.16 3.14 3.12 3.10 2.97 2.96 2.94 2.93 2.91 2.90 2.89 2.88 2.86 2.85 2.84 2.39
0.90 0.84 1.08 0.84 9.50 4.51 0.89 0.88 0.90 0.95 0.99 1.79 2.85
3.0 12.5 12.0 11.5 11.0 10.5 10.0 9.5 9.0 8.5 8.0 7.5 7.0 6.5 6.0 5.5 5.0 4.5 4.0 3.5 3.0 2.5 2.0 1.5 1.0 0.5 0.0 -0
AcHN
O
P
O
N
H

3i

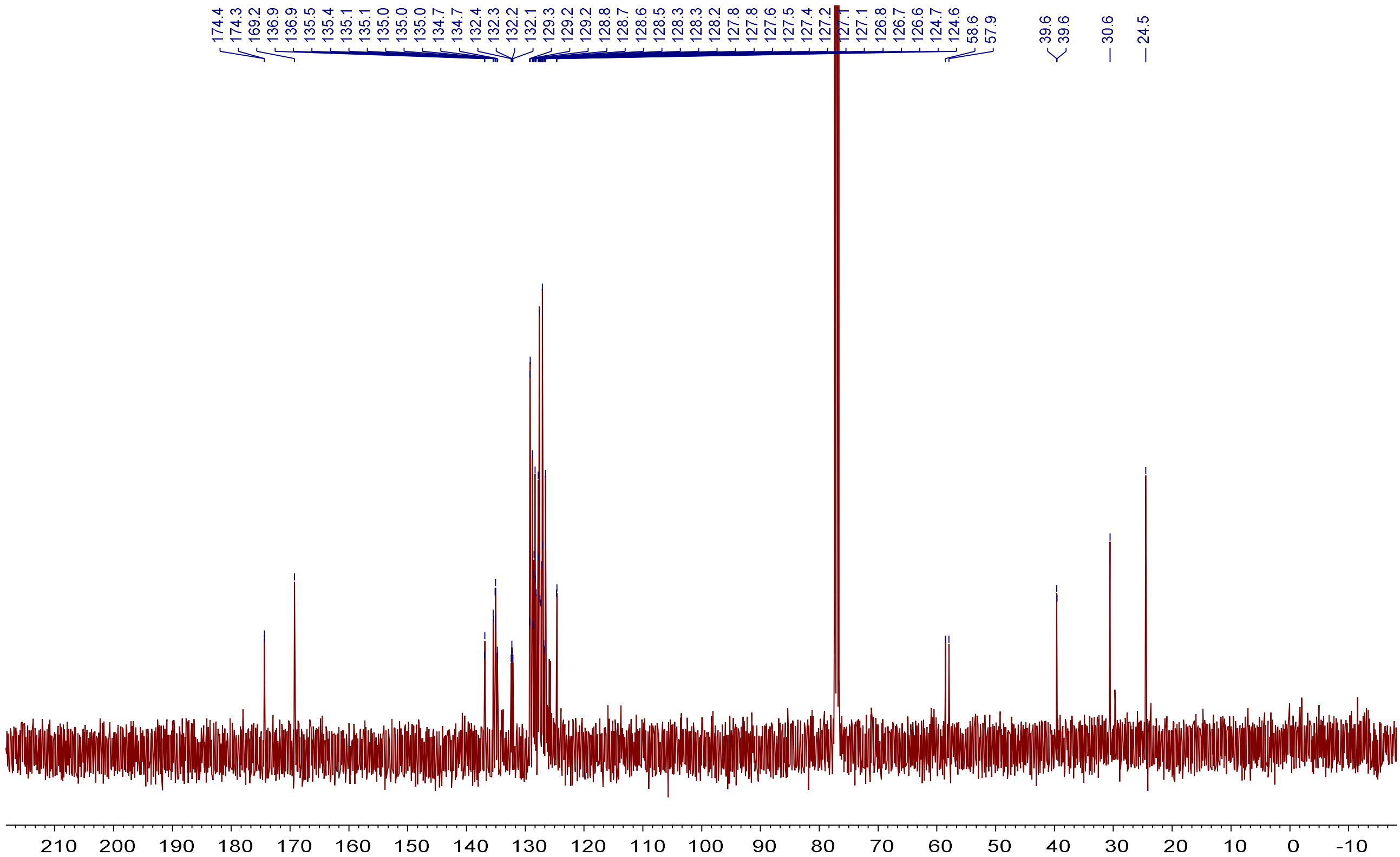
174.4 174.3 169.2 136.9 136.9 135.5 135.4 135.1 135.0 135.0 134.7 134.7 132.4 132.3 132.2 132.1 129.3 129.2 129.2 128.8 128.7 128.6 128.5 128.3 128.2 127.8 127.6 127.5 127.4 127.2 127.1 126.8 126.7 126.6 124.7 124.6 58.6 57.9 39.6 39.6 30.6 24.5
210 200 190 180 170 160 150 140 130 120 110 100 90 80 70 60 50 40 30 20 10 0 -10

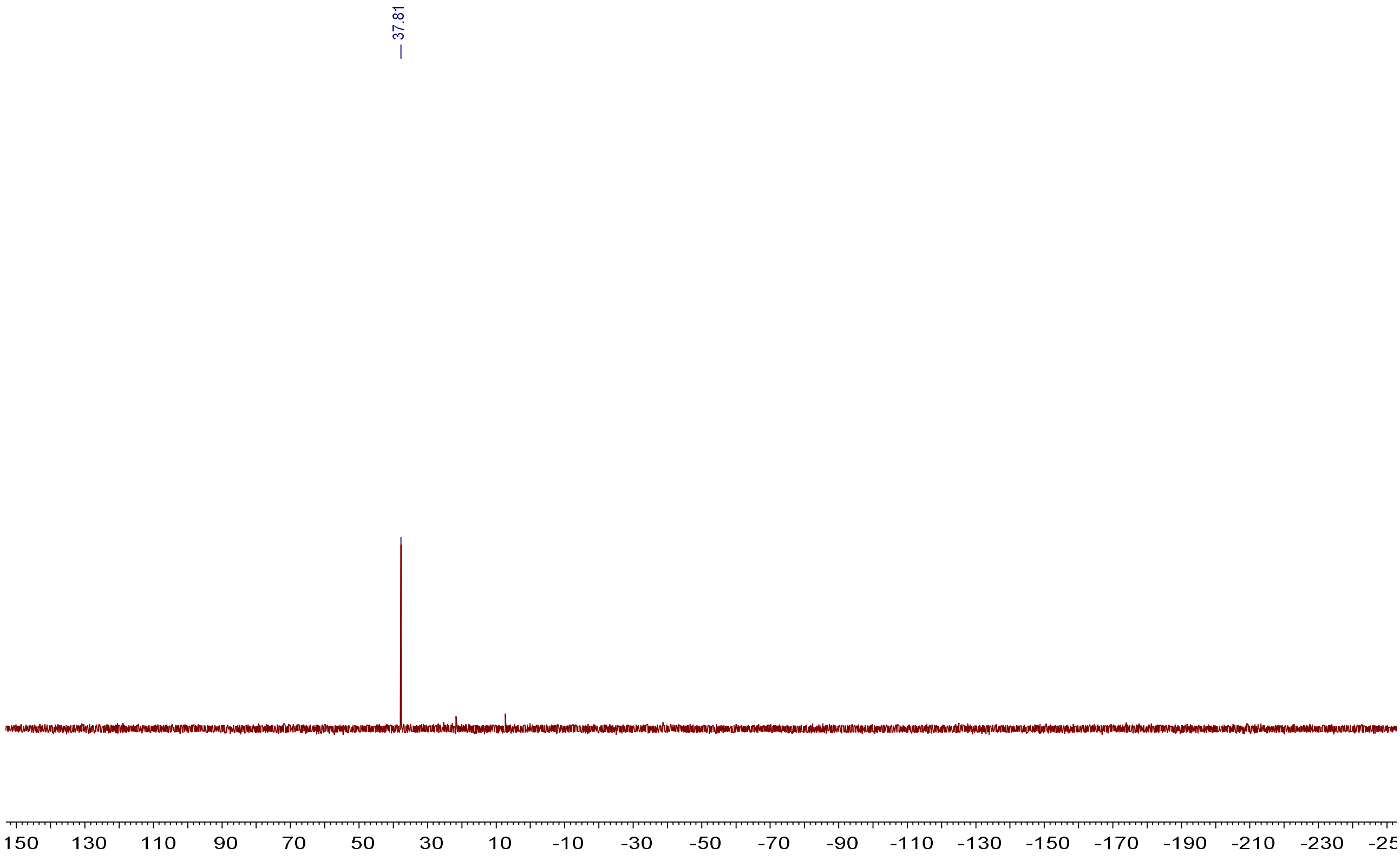
37.81
150 130 110 90 70 50 30 10 -10 -30 -50 -70 -90 -110 -130 -150 -170 -190 -210 -230

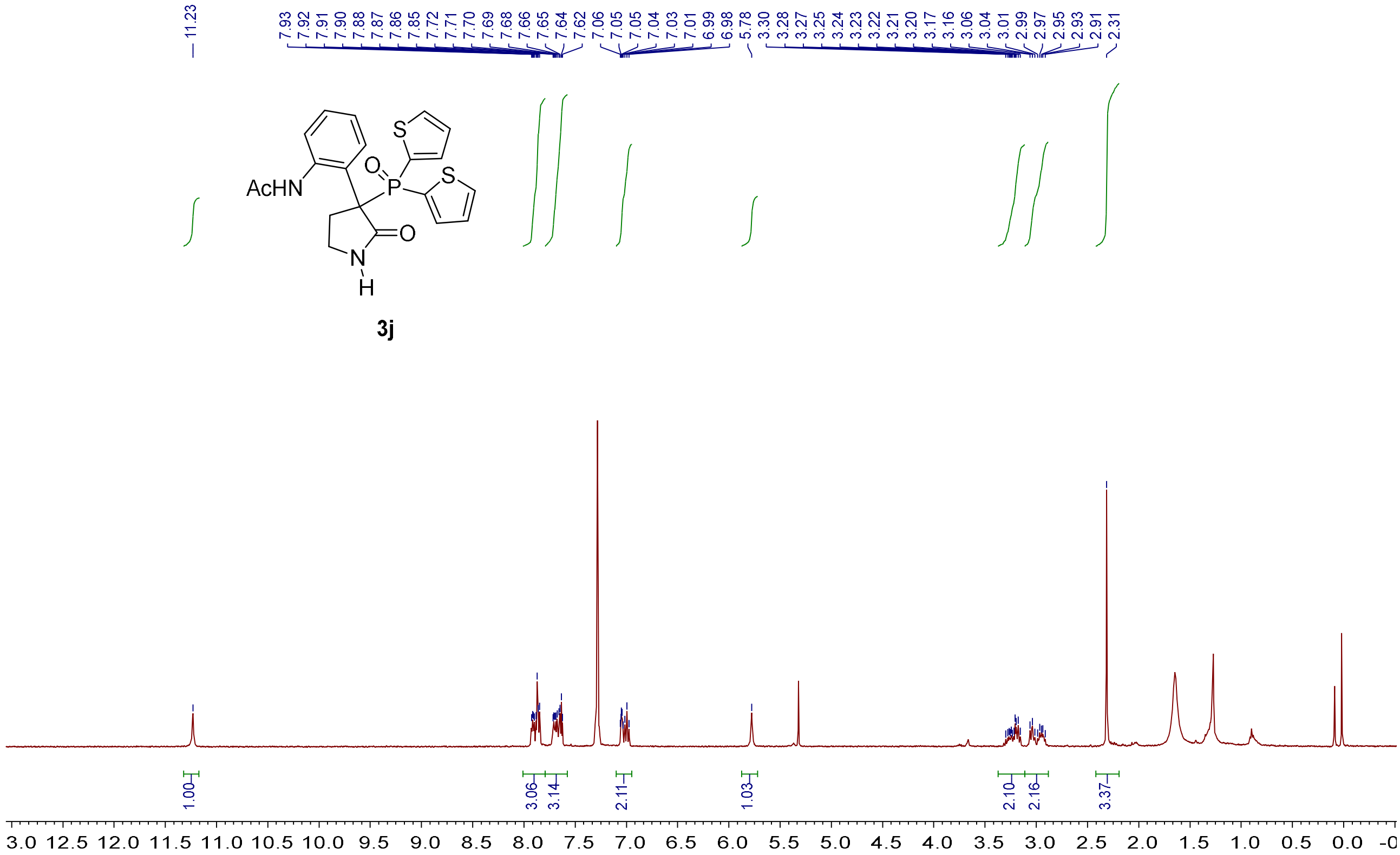

11.23
7.93 7.92 7.91 7.90 7.88 7.87 7.86 7.85 7.72 7.71 7.70 7.69 7.68 7.66 7.65 7.64 7.62 7.06 7.05 7.05 7.04 7.03 7.01 6.99 6.98 5.78 3.30 3.28 3.27 3.25 3.24 3.23 3.22 3.21 3.20 3.17 3.16 3.06 3.04 3.01 2.99 2.97 2.95 2.93 2.91 2.31
AcHN
O
P
S
S
O
N
H
1.00 3.06 3.14 2.11 1.03 2.10 2.16 3.37
3.0 12.5 12.0 11.5 11.0 10.5 10.0 9.5 9.0 8.5 8.0 7.5 7.0 6.5 6.0 5.5 5.0 4.5 4.0 3.5 3.0 2.5 2.0 1.5 1.0 0.5 0.0 -0

3j

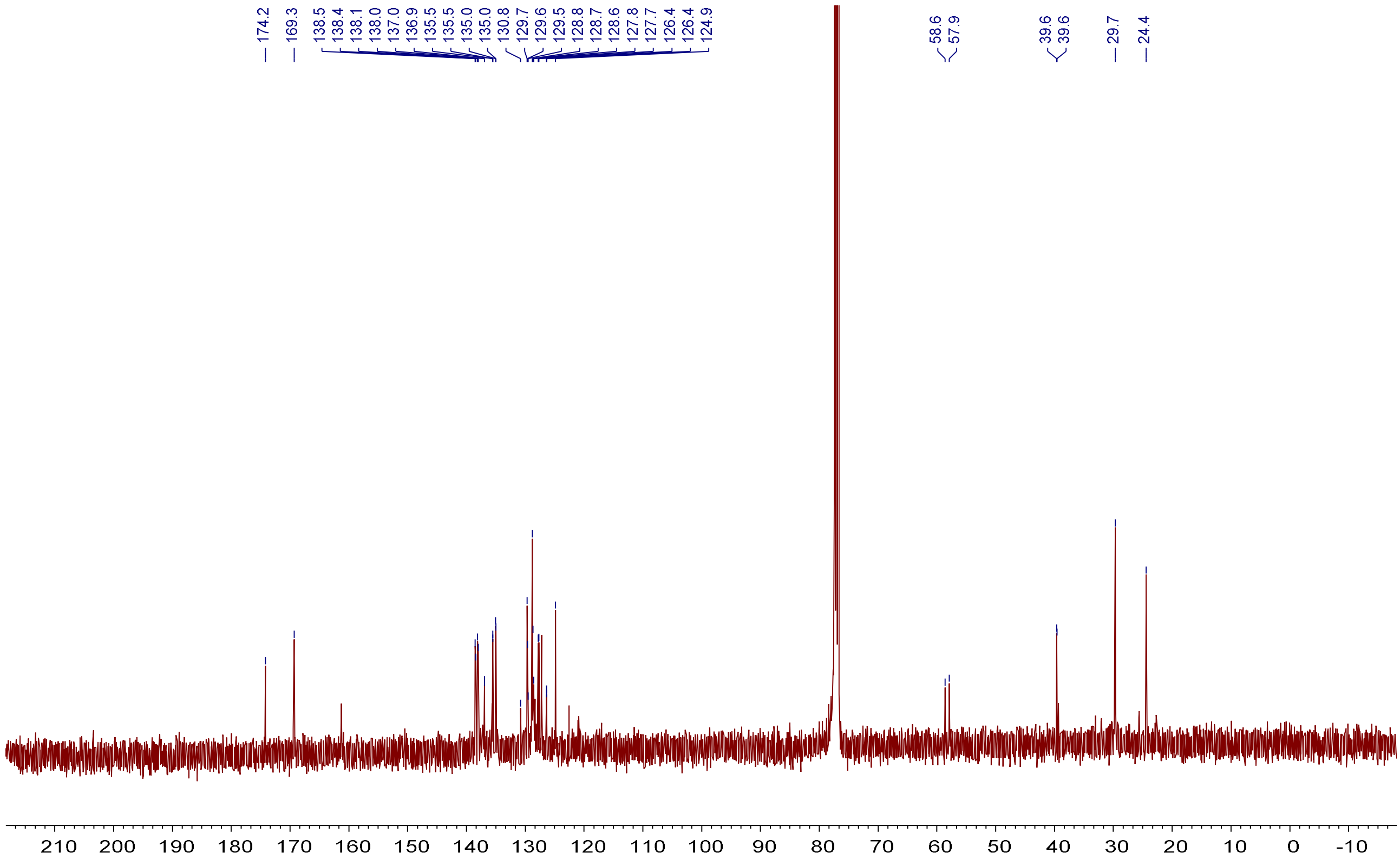

174.2 169.3 138.5 138.4 138.1 138.0 137.0 136.9 135.5 135.5 135.0 135.0 130.8 129.7 129.6 129.5 128.8 128.7 128.6 127.8 127.7 126.4 126.4 124.9
58.6 57.9
39.6 39.6
29.7
24.4
210 200 190 180 170 160 150 140 130 120 110 100 90 80 70 60 50 40 30 20 10 0 -10

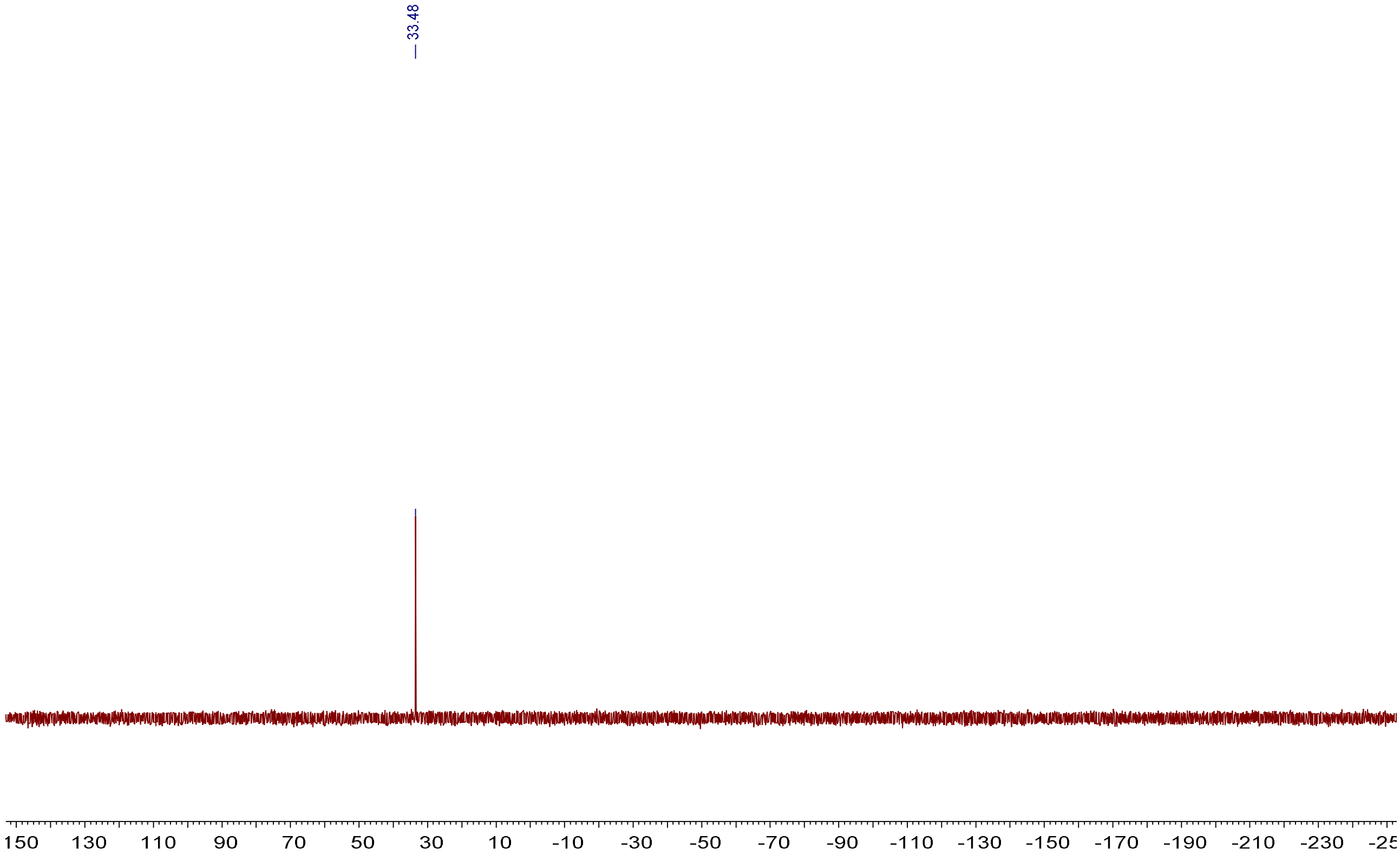
33.48
150 130 110 90 70 50 30 10 -10 -30 -50 -70 -90 -110 -130 -150 -170 -190 -210 -230 -25

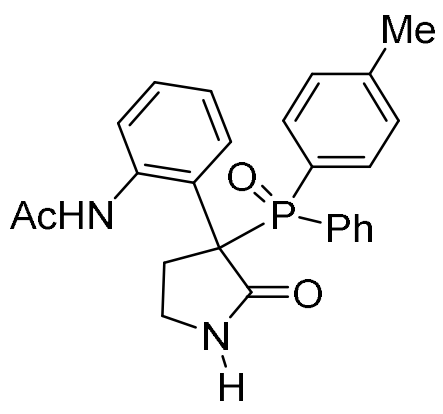
Me
AcHN
O
P
Ph
O
N
H

3k

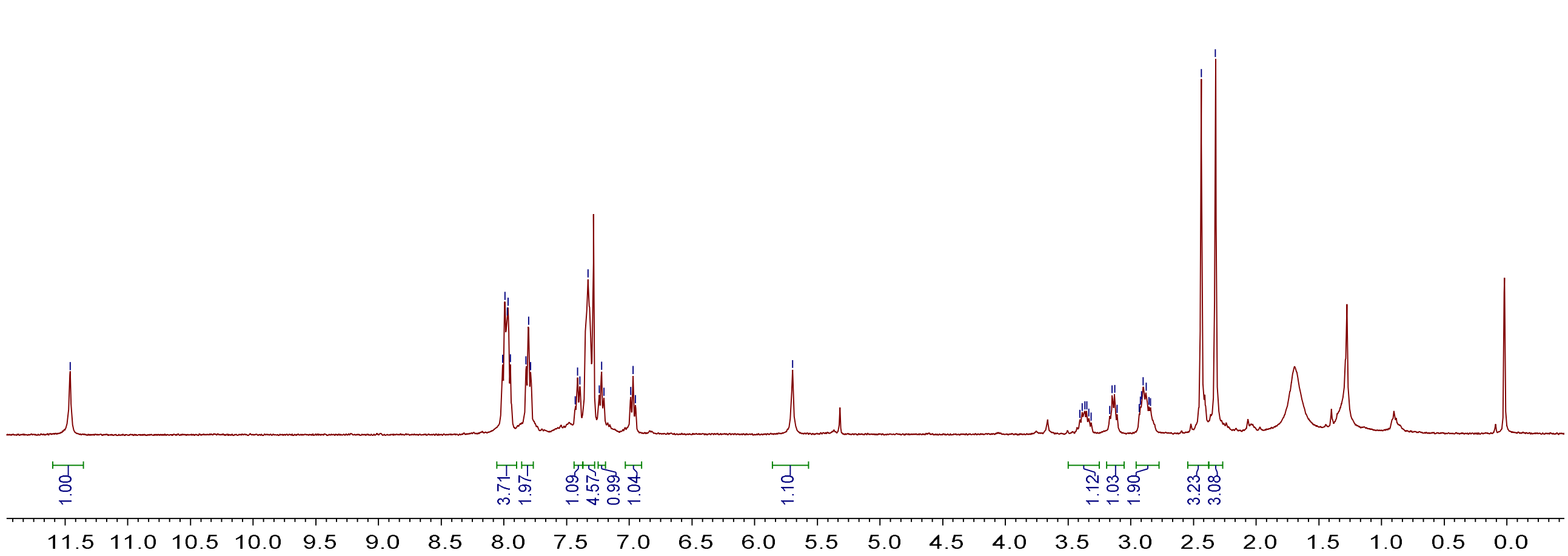
11.46
8.01 7.99 7.97 7.97 7.95 7.82 7.80 7.79 7.43 7.41 7.39 7.33 7.24 7.22 7.20 6.99 6.97 6.95
5.70
3.41 3.39 3.37 3.35 3.34 3.32 3.17 3.15 3.13 3.11 2.93 2.92 2.91 2.90 2.88 2.86 2.84 2.44 2.32
1.00 3.71 1.97 1.09 4.57 0.99 1.04 1.10 1.12 1.03 1.90 3.23 3.08
11.5 11.0 10.5 10.0 9.5 9.0 8.5 8.0 7.5 7.0 6.5 6.0 5.5 5.0 4.5 4.0 3.5 3.0 2.5 2.0 1.5 1.0 0.5 0.0

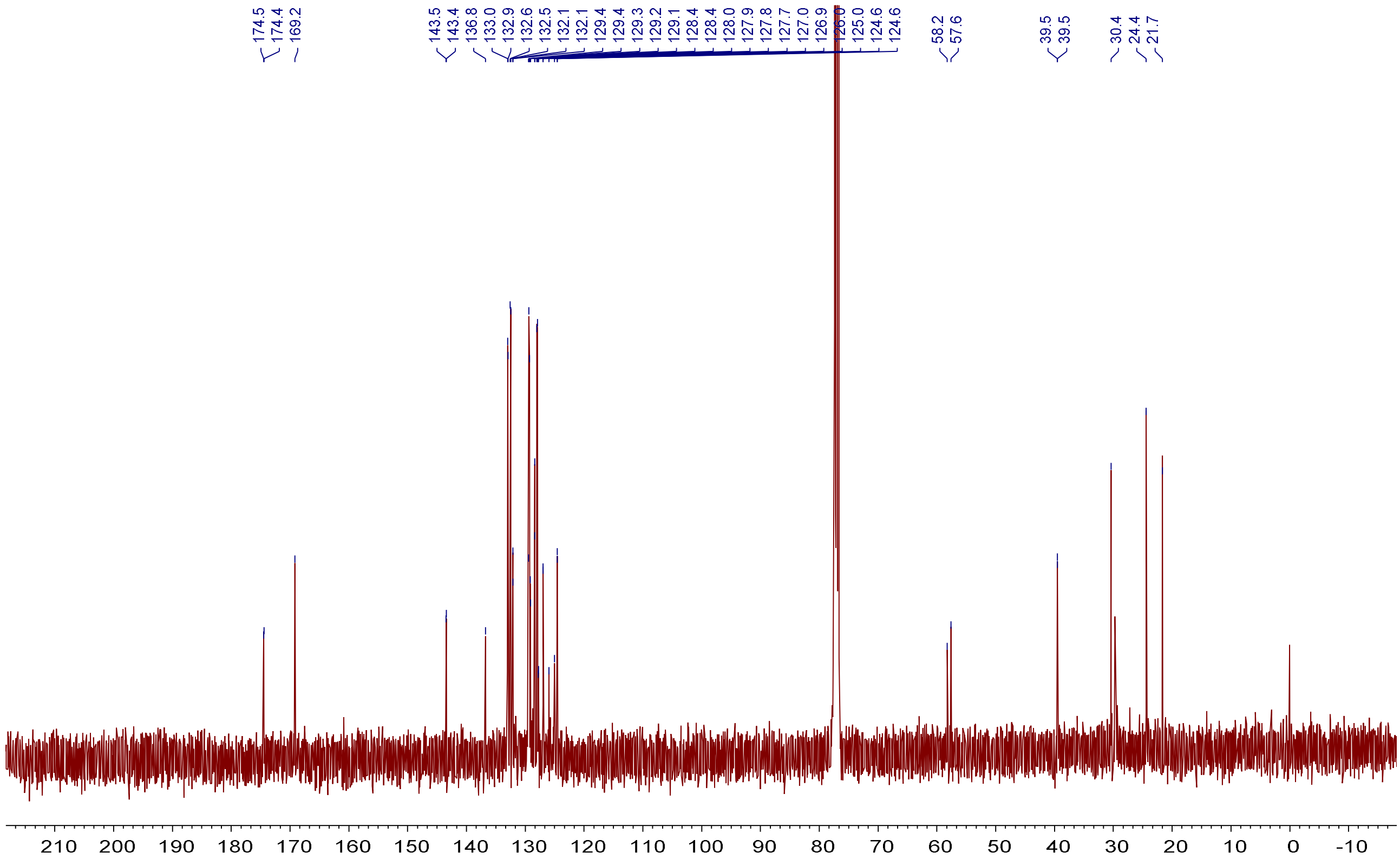
174.5 174.4 169.2
143.5 143.4 136.8 133.0 132.9 132.6 132.5 132.1 132.1 129.4 129.4 129.3 129.2 129.1 128.4 128.0 127.9 127.8 127.7 127.0 126.9 125.0 125.0 124.6 124.6
58.2 57.6
39.5 39.5
30.4 24.4 21.7
210 200 190 180 170 160 150 140 130 120 110 100 90 80 70 60 50 40 30 20 10 0 -10

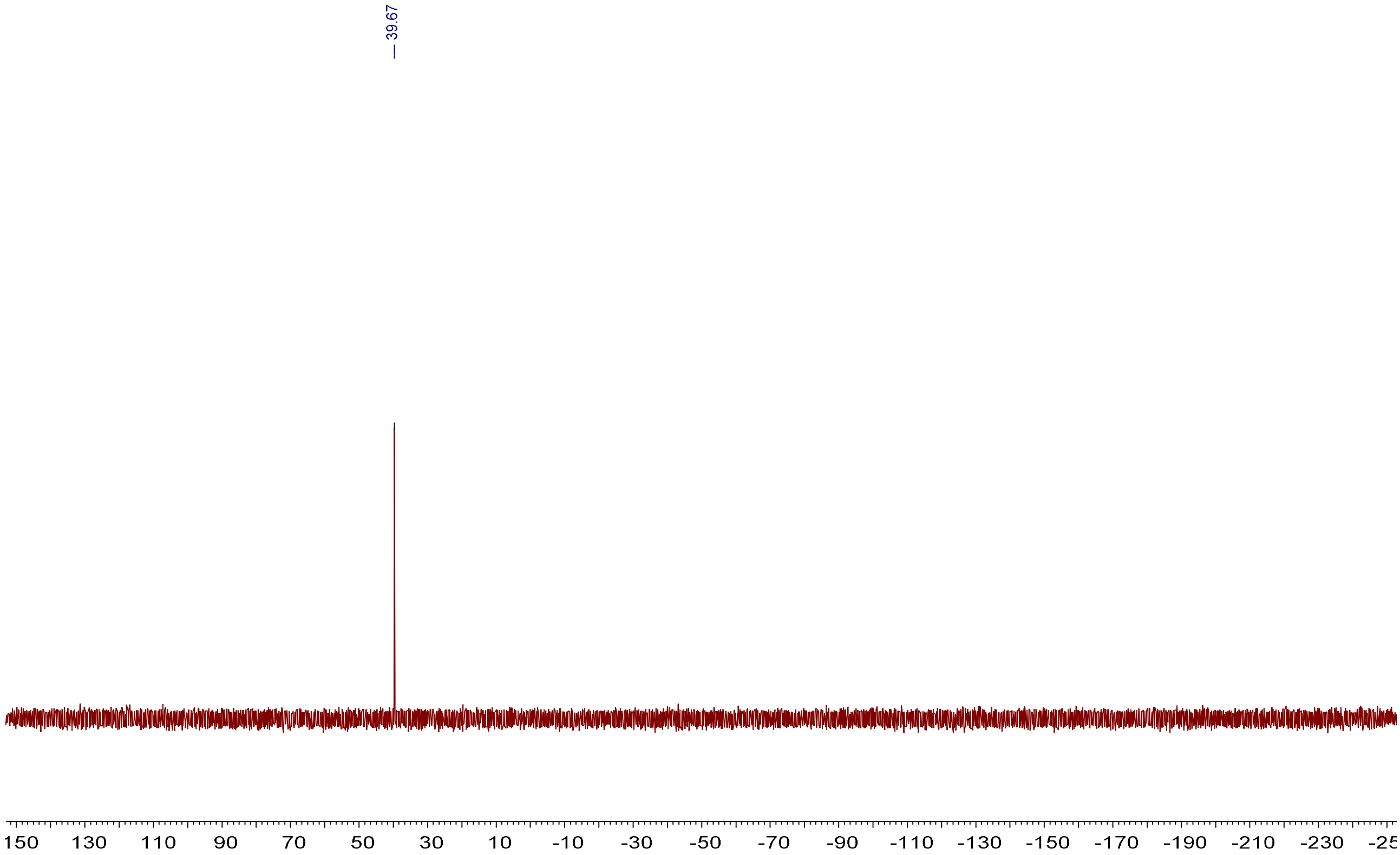

39.67
150 130 110 90 70 50 30 10 -10 -30 -50 -70 -90 -110 -130 -150 -170 -190 -210 -230 -25

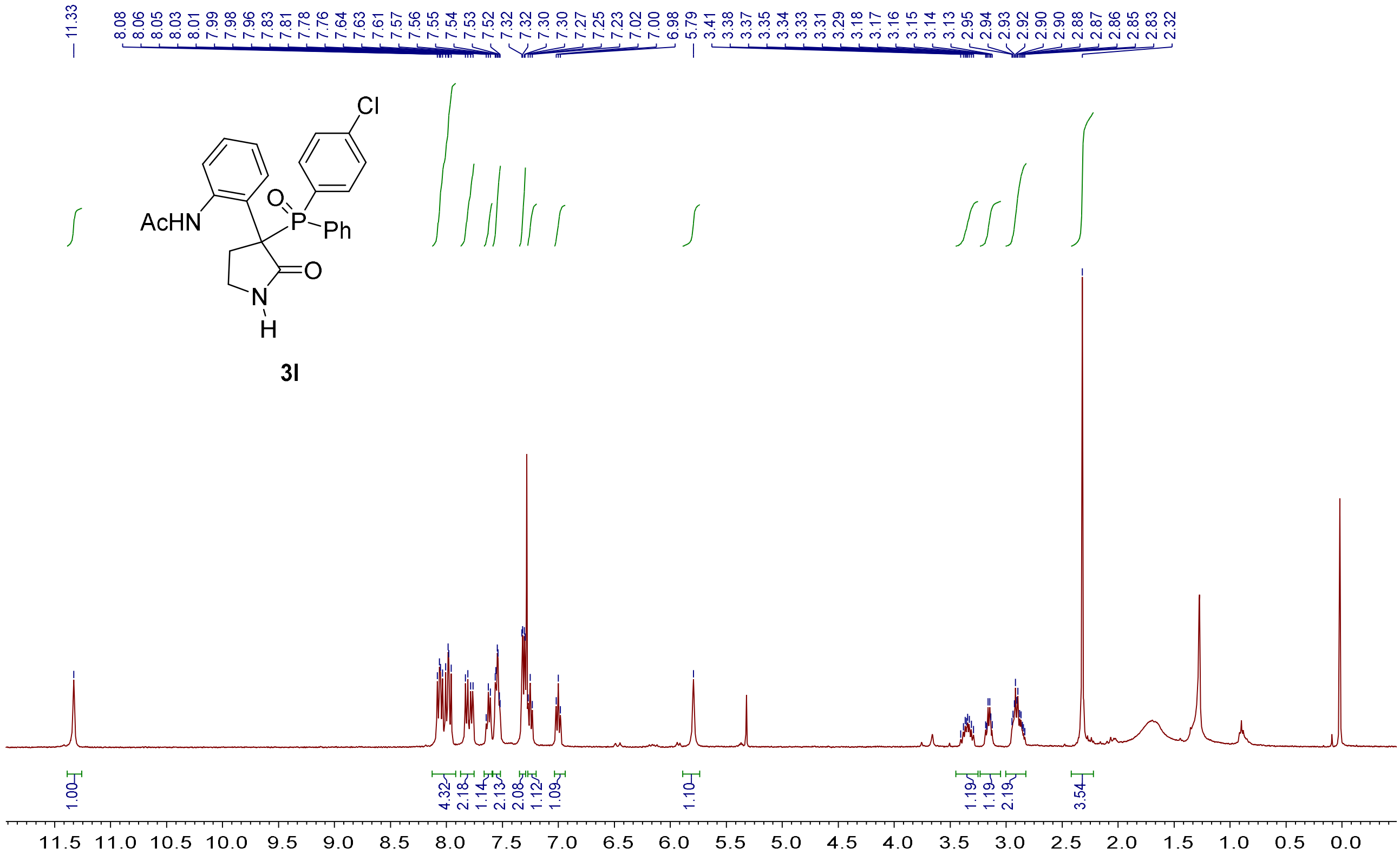

Cl
AcHN
O
P
Ph
O
N
H
3l
11.33
8.08 8.06 8.05 8.03 8.01 7.99 7.98 7.96 7.83 7.81 7.78 7.76 7.64 7.63 7.61 7.57 7.56 7.55 7.54 7.53 7.52 7.32 7.32 7.30 7.30 7.27 7.25 7.23 7.02 7.00 6.98
5.79
3.41 3.38 3.37 3.35 3.34 3.33 3.31 3.29 3.18 3.17 3.16 3.15 3.14 3.13 2.95 2.94 2.93 2.92 2.90 2.90 2.88 2.87 2.86 2.85 2.83 2.32
1.00
4.32 2.18 1.14 2.13 2.08 1.12 1.09
1.10
1.19 1.19 2.19
3.54
11.5 11.0 10.5 10.0 9.5 9.0 8.5 8.0 7.5 7.0 6.5 6.0 5.5 5.0 4.5 4.0 3.5 3.0 2.5 2.0 1.5 1.0 0.5 0.0

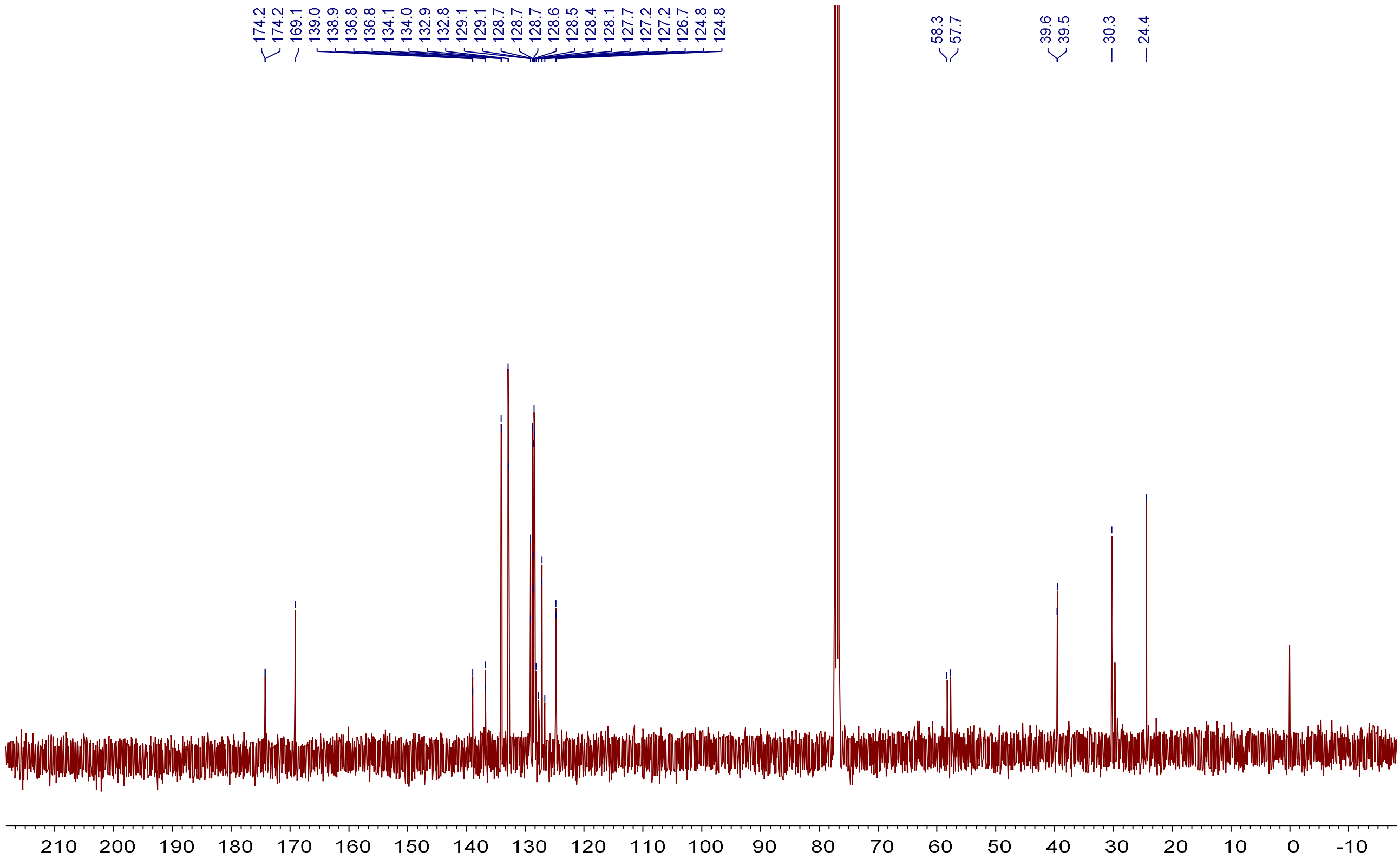

174.2 174.2 169.1
139.0 138.9 136.8 136.8 134.1 134.0 132.9 132.8 129.1 129.1 128.7 128.7 128.7 128.6 128.5 128.4 128.1 127.7 127.2 127.2 126.7 124.8 124.8
58.3 57.7
39.6 39.5
30.3
24.4
210 200 190 180 170 160 150 140 130 120 110 100 90 80 70 60 50 40 30 20 10 0 -10

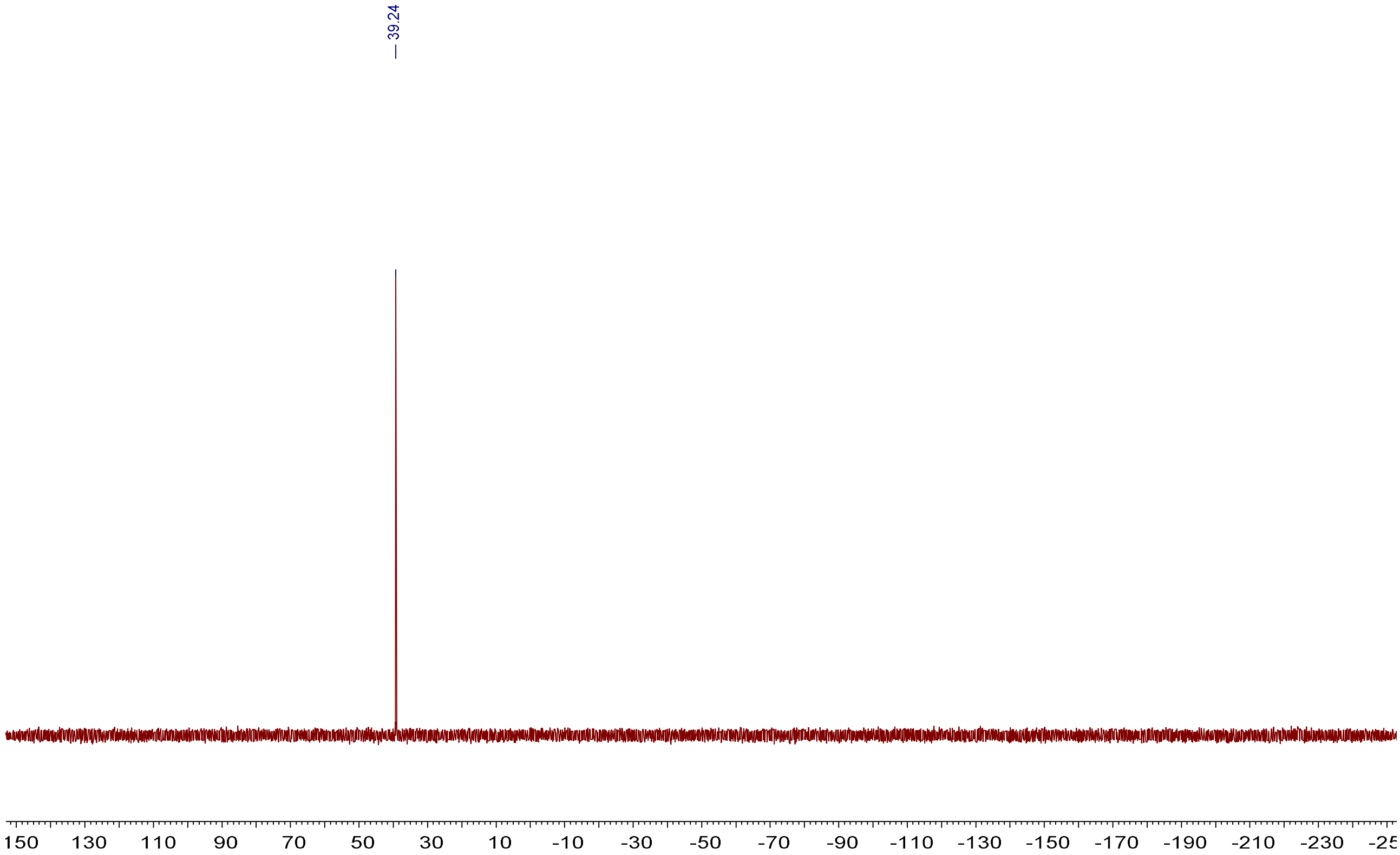
39.24
150
130
110
90
70
50
30
10
-10
-30
-50
-70
-90
-110
-130
-150
-170
-190
-210
-230
-25

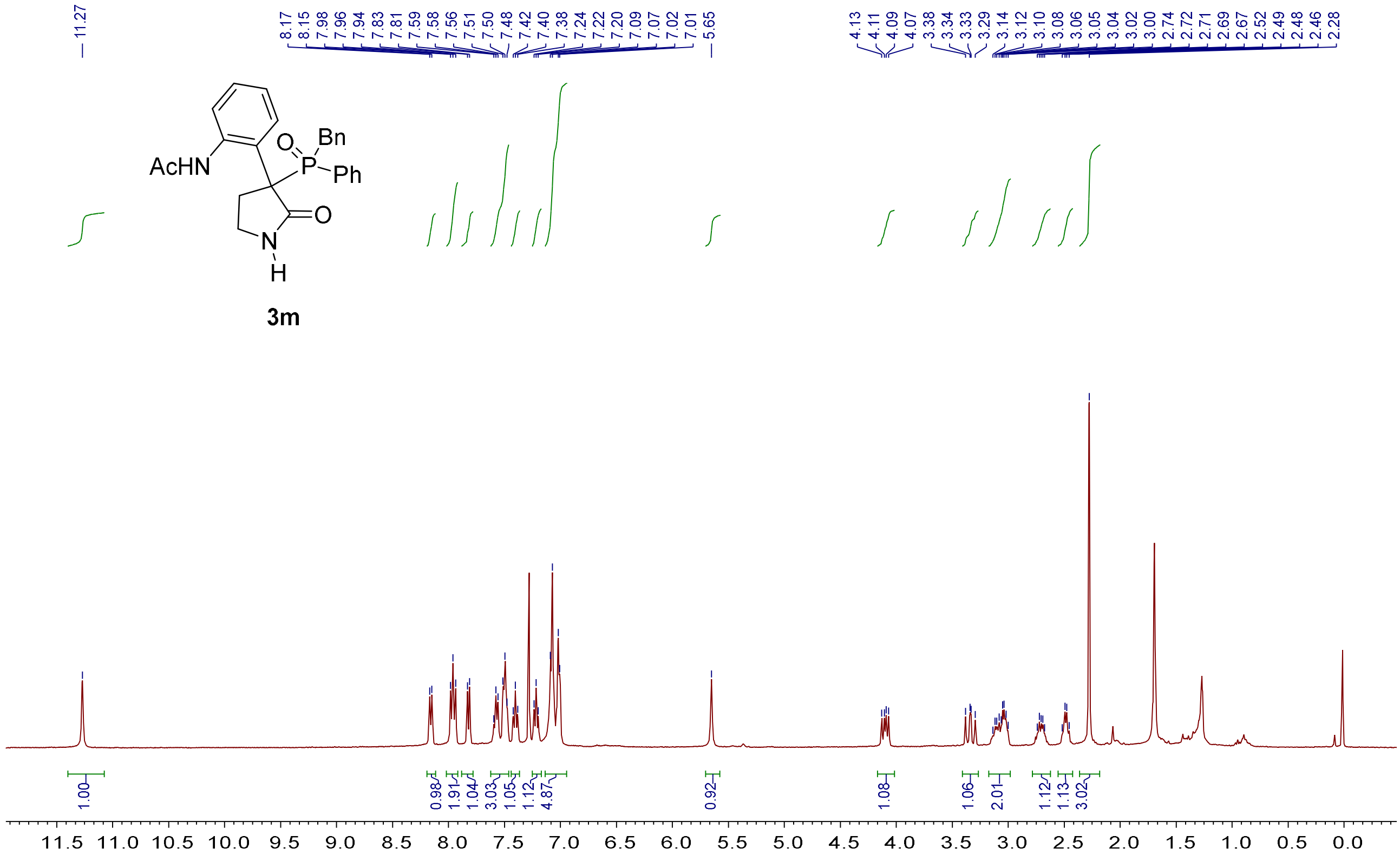

3m

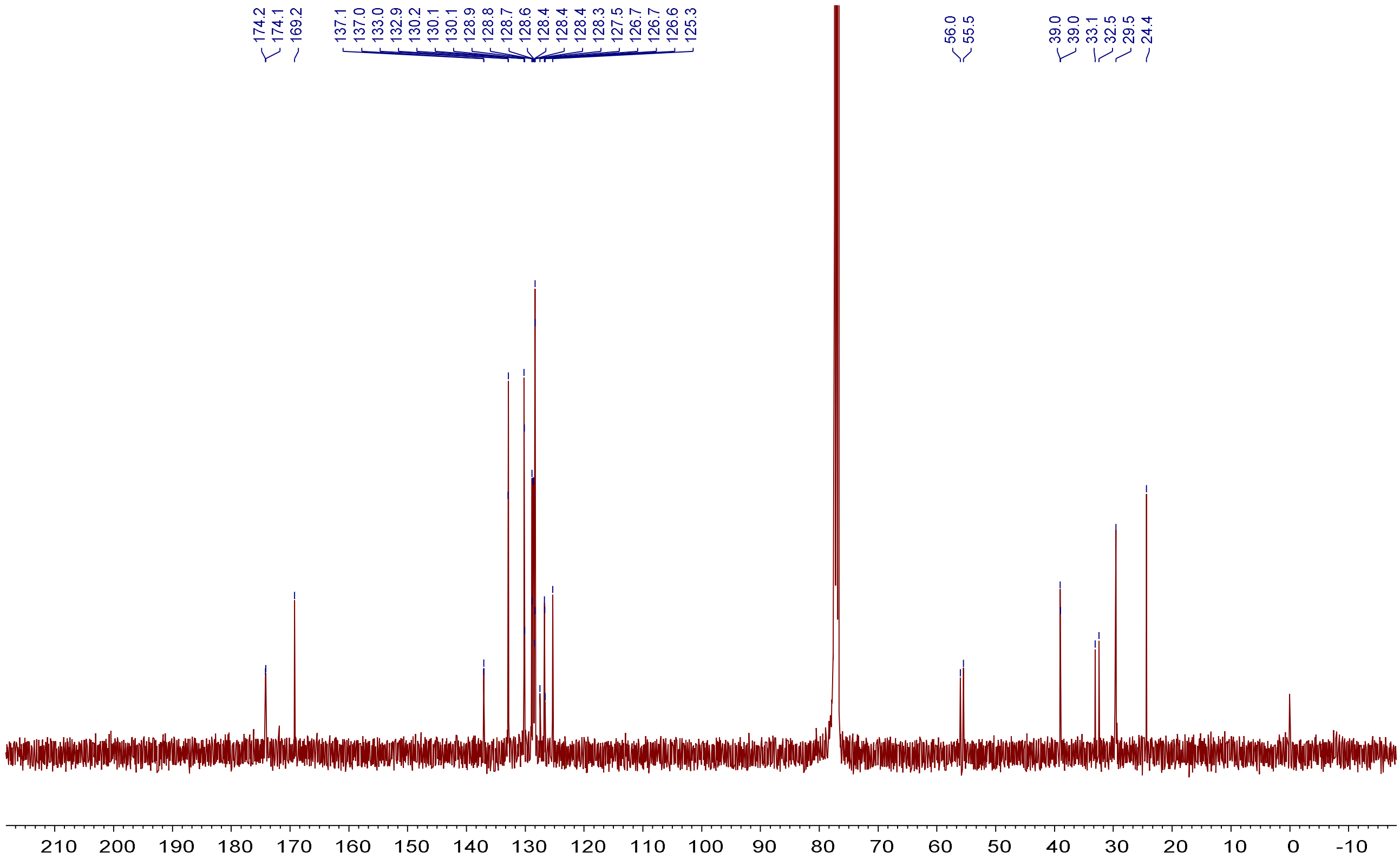

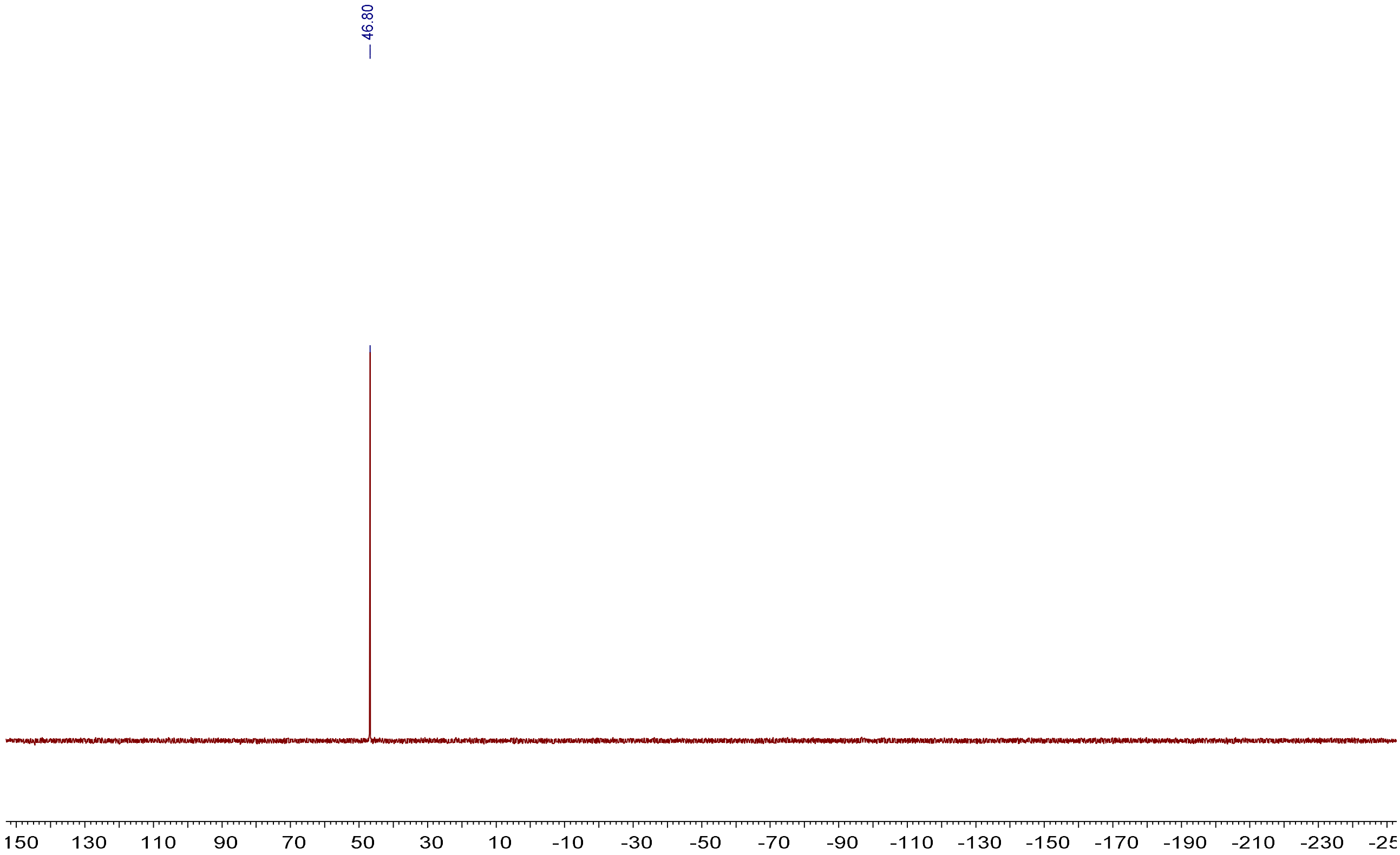
46.80
150 130 110 90 70 50 30 10 -10 -30 -50 -70 -90 -110 -130 -150 -170 -190 -210 -230 -25

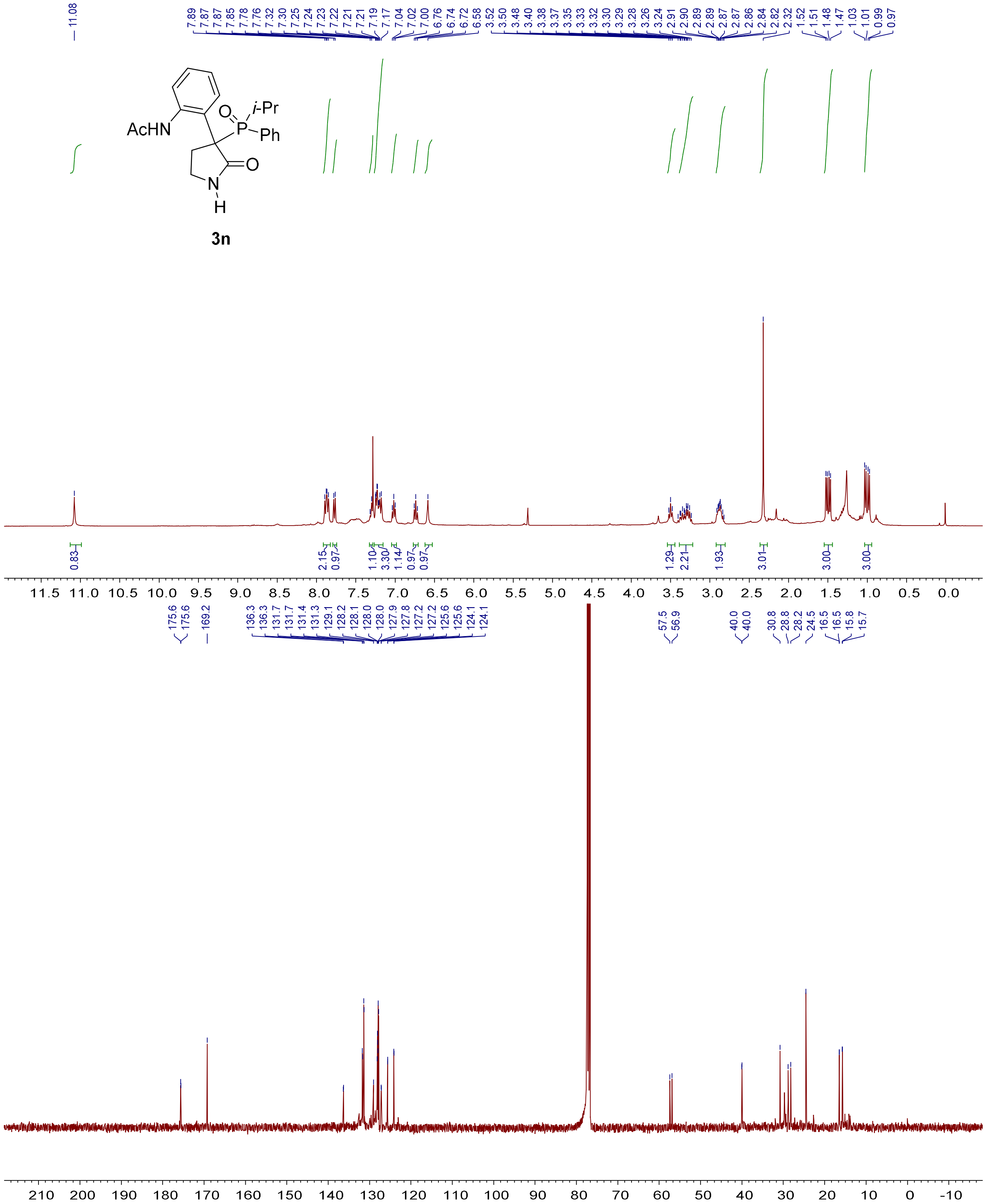
11.08
7.89 7.87 7.87 7.85 7.78 7.76 7.32 7.30 7.25 7.24 7.23 7.22 7.21 7.21 7.19 7.17 7.04 7.02 7.00 6.76 6.74 6.72 6.58 3.52 3.50 3.48 3.40 3.38 3.37 3.35 3.33 3.32 3.30 3.29 3.28 3.26 3.24 2.91 2.90 2.89 2.89 2.87 2.87 2.86 2.84 2.82 2.32 1.52 1.51 1.48 1.47 1.03 1.01 0.99 0.97
AcHN
O
P
i-Pr
Ph
N
H
O
3n
0.83 2.15 0.97 1.10 3.30 1.14 0.97 0.97 1.29 2.21 1.93 3.01 3.00 3.00
11.5 11.0 10.5 10.0 9.5 9.0 8.5 8.0 7.5 7.0 6.5 6.0 5.5 5.0 4.5 4.0 3.5 3.0 2.5 2.0 1.5 1.0 0.5 0.0
175.6 175.6 169.2 136.3 136.3 131.7 131.4 131.3 129.1 128.2 128.1 128.0 128.0 127.9 127.8 127.2 127.2 125.6 125.6 124.1 124.1 57.5 56.9 40.0 40.0 30.8 28.8 28.2 24.5 16.5 16.5 15.8 15.7
210 200 190 180 170 160 150 140 130 120 110 100 90 80 70 60 50 40 30 20 10 0 -10

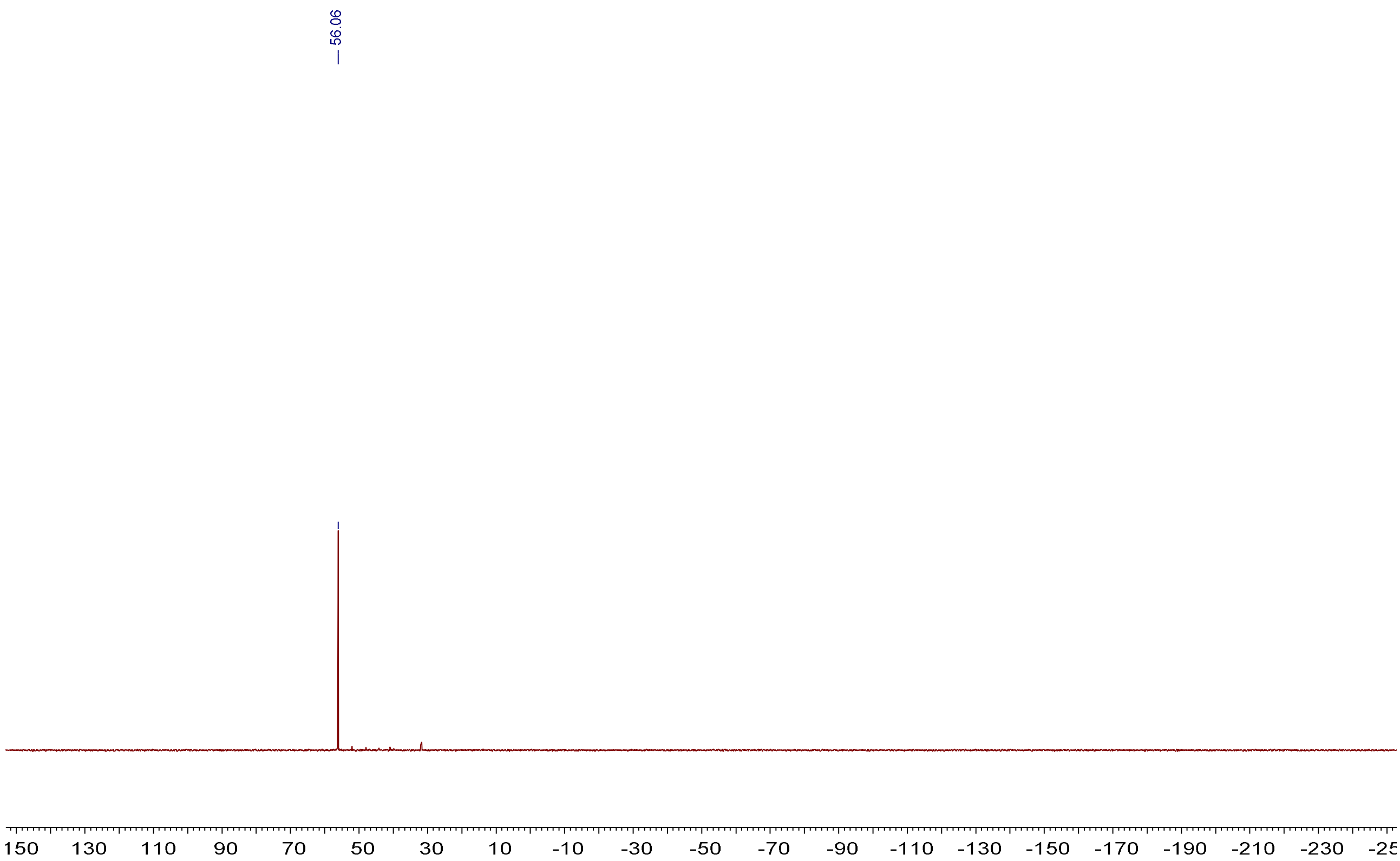
56.06
150
130
110
90
70
50
30
10
-10
-30
-50
-70
-90
-110
-130
-150
-170
-190
-210
-230

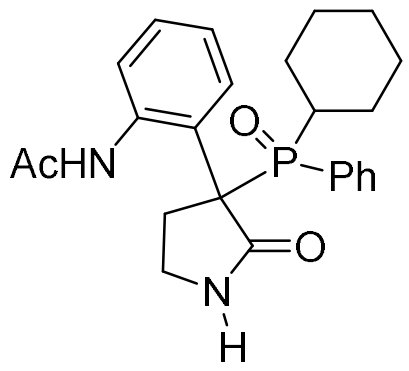
AcHN
O
P
Ph
O
N
H

3o

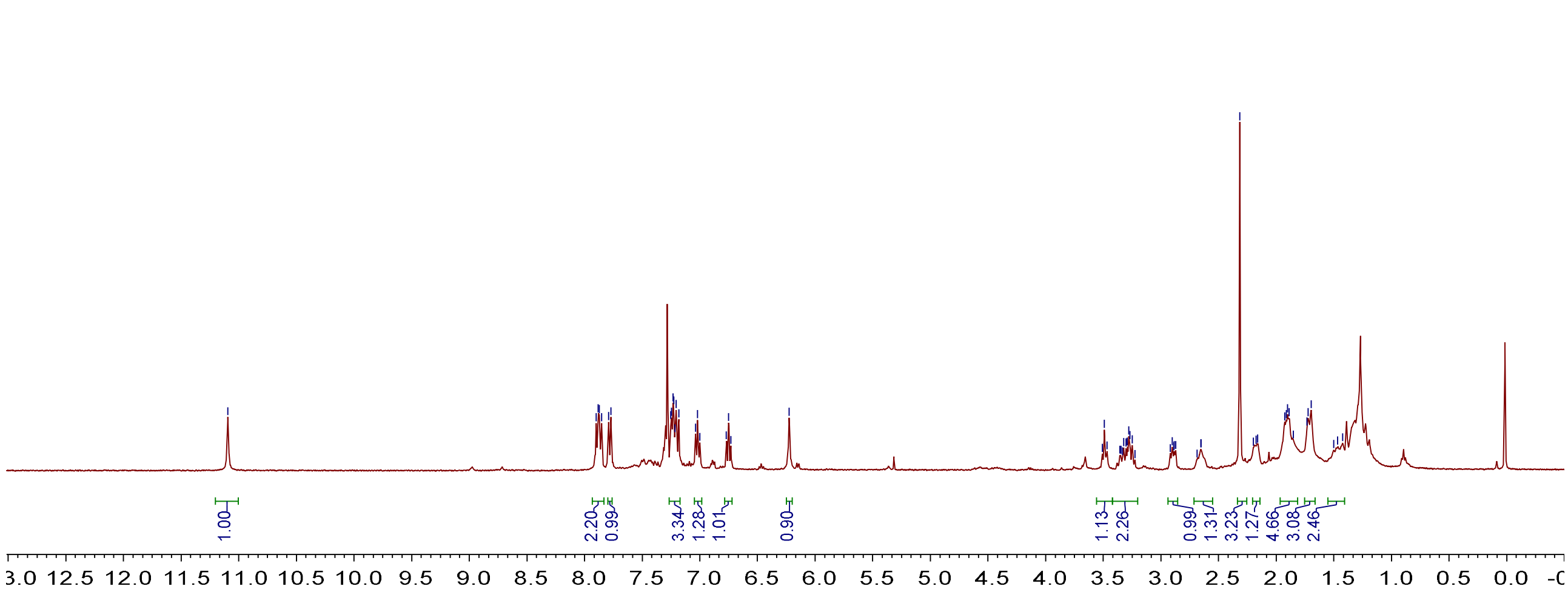
11.09
7.90 7.88 7.87 7.85 7.79 7.77 7.25 7.25 7.23 7.23 7.21 7.20 7.18 7.04 7.02 7.00 6.77 6.75 6.73 6.22
3.51 3.49 3.47 3.36 3.35 3.33 3.32 3.30 3.29 3.28 3.27 3.25 3.23 2.92 2.90 2.89 2.88 2.87 2.69 2.65 2.65 2.32 2.20 2.18 2.16 1.93 1.91 1.90 1.89 1.85 1.73 1.72 1.70 1.50 1.47 1.42
1.00 2.20 0.99 3.34 1.28 1.01 0.90 1.13 2.26 0.99 1.31 3.23 1.27 4.66 3.08 2.46
3.0 12.5 12.0 11.5 11.0 10.5 10.0 9.5 9.0 8.5 8.0 7.5 7.0 6.5 6.0 5.5 5.0 4.5 4.0 3.5 3.0 2.5 2.0 1.5 1.0 0.5 0.0

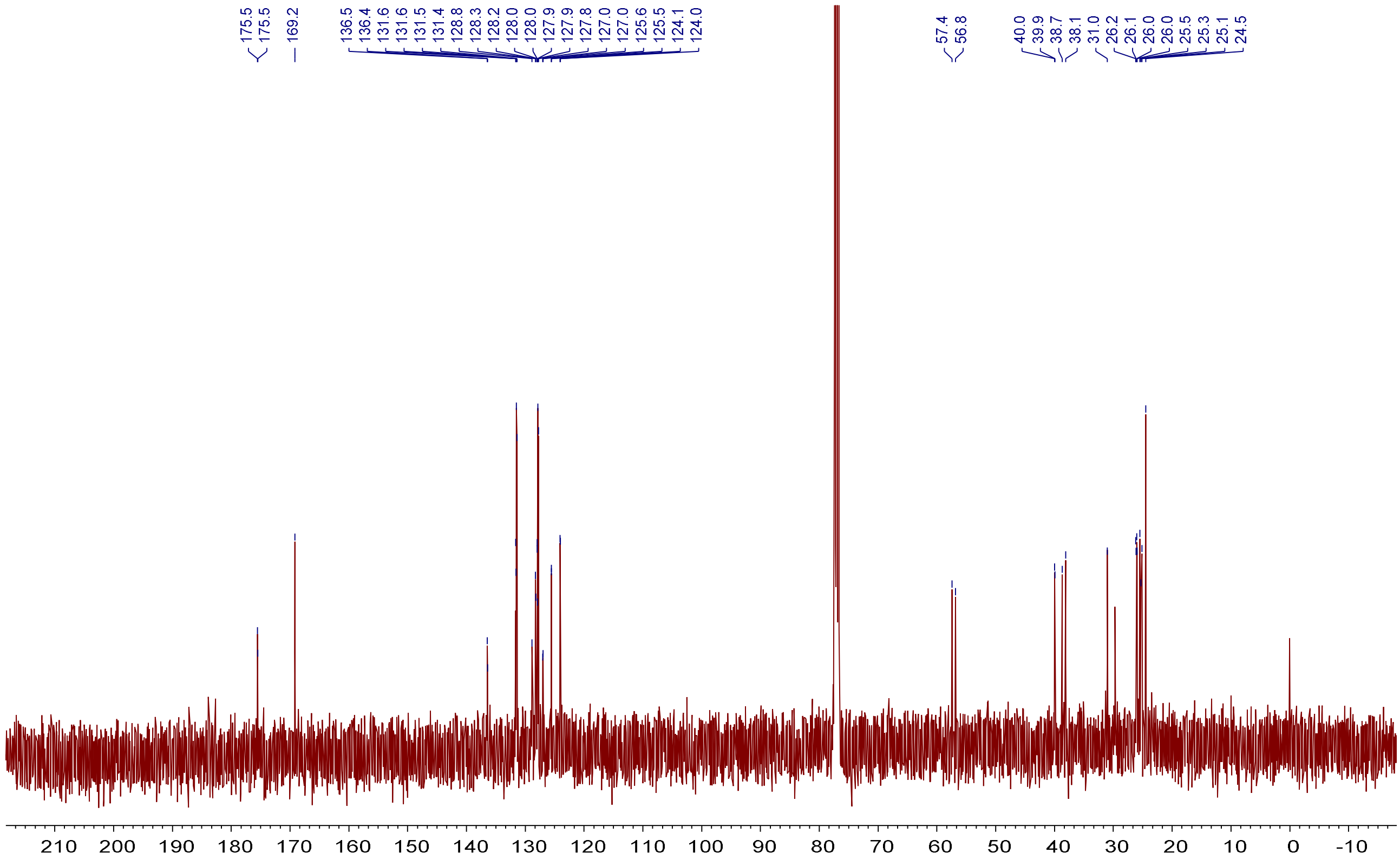
175.5 175.5 169.2
136.5 136.4 131.6 131.5 131.4 128.8 128.3 128.2 128.0 128.0 127.9 127.8 127.0 127.0 125.6 125.5 124.1 124.0
57.4 56.8
40.0 39.9 38.7 38.1 31.0 26.2 26.1 26.0 26.0 25.5 25.3 25.1 24.5
210 200 190 180 170 160 150 140 130 120 110 100 90 80 70 60 50 40 30 20 10 0 -10

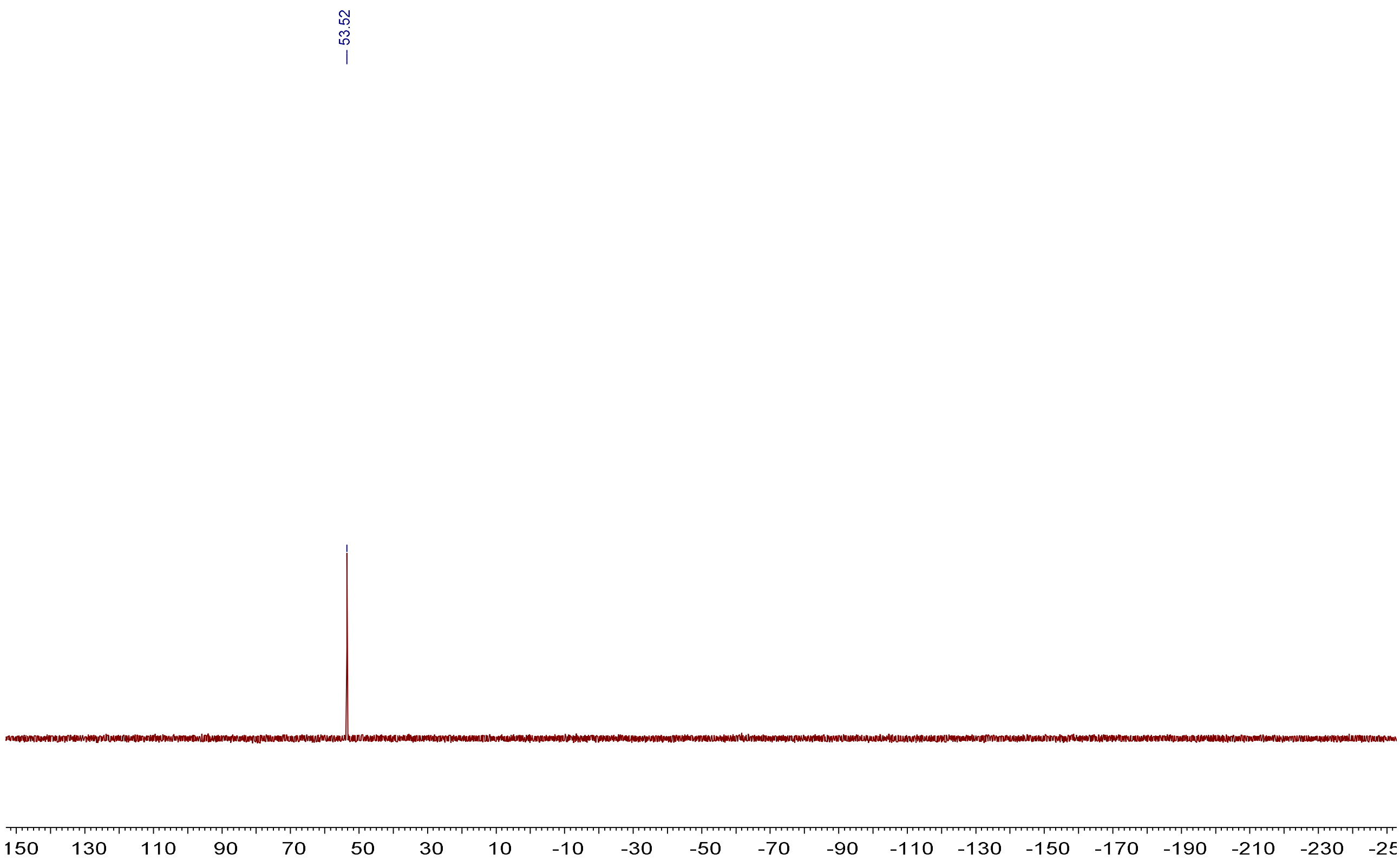
53.52
150 130 110 90 70 50 30 10 -10 -30 -50 -70 -90 -110 -130 -150 -170 -190 -210 -230 -25

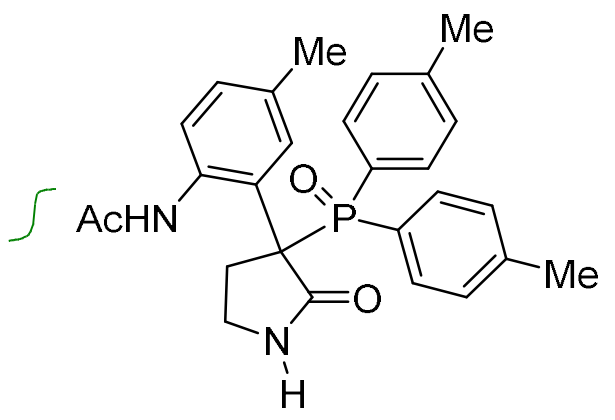

Me
Me
AcHN
O
P
Me
O
N
H

3p

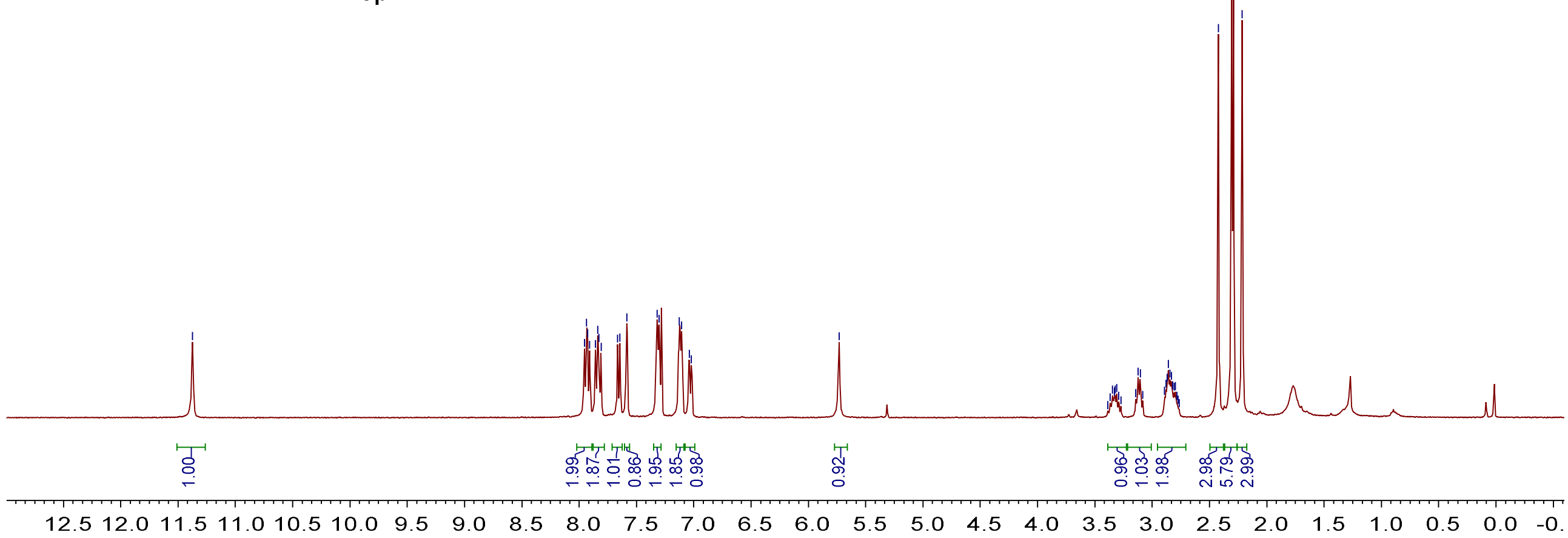

11.37
7.95 7.93 7.93 7.91 7.86 7.84 7.83 7.81 7.66 7.64 7.58 7.32 7.30 7.13 7.11 7.04 7.02
5.73
3.39 3.37 3.35 3.33 3.32 3.31 3.29 3.27 3.14 3.12 3.10 3.08 2.89 2.88 2.87 2.86 2.85 2.83 2.82 2.81 2.80 2.79 2.77 2.76 2.42 2.31 2.29 2.21
1.00
1.99 1.87 1.01 0.86 1.95 1.85 0.98
0.92
0.96 1.03 1.98 2.98 5.79 2.99
12.5 12.0 11.5 11.0 10.5 10.0 9.5 9.0 8.5 8.0 7.5 7.0 6.5 6.0 5.5 5.0 4.5 4.0 3.5 3.0 2.5 2.0 1.5 1.0 0.5 0.0

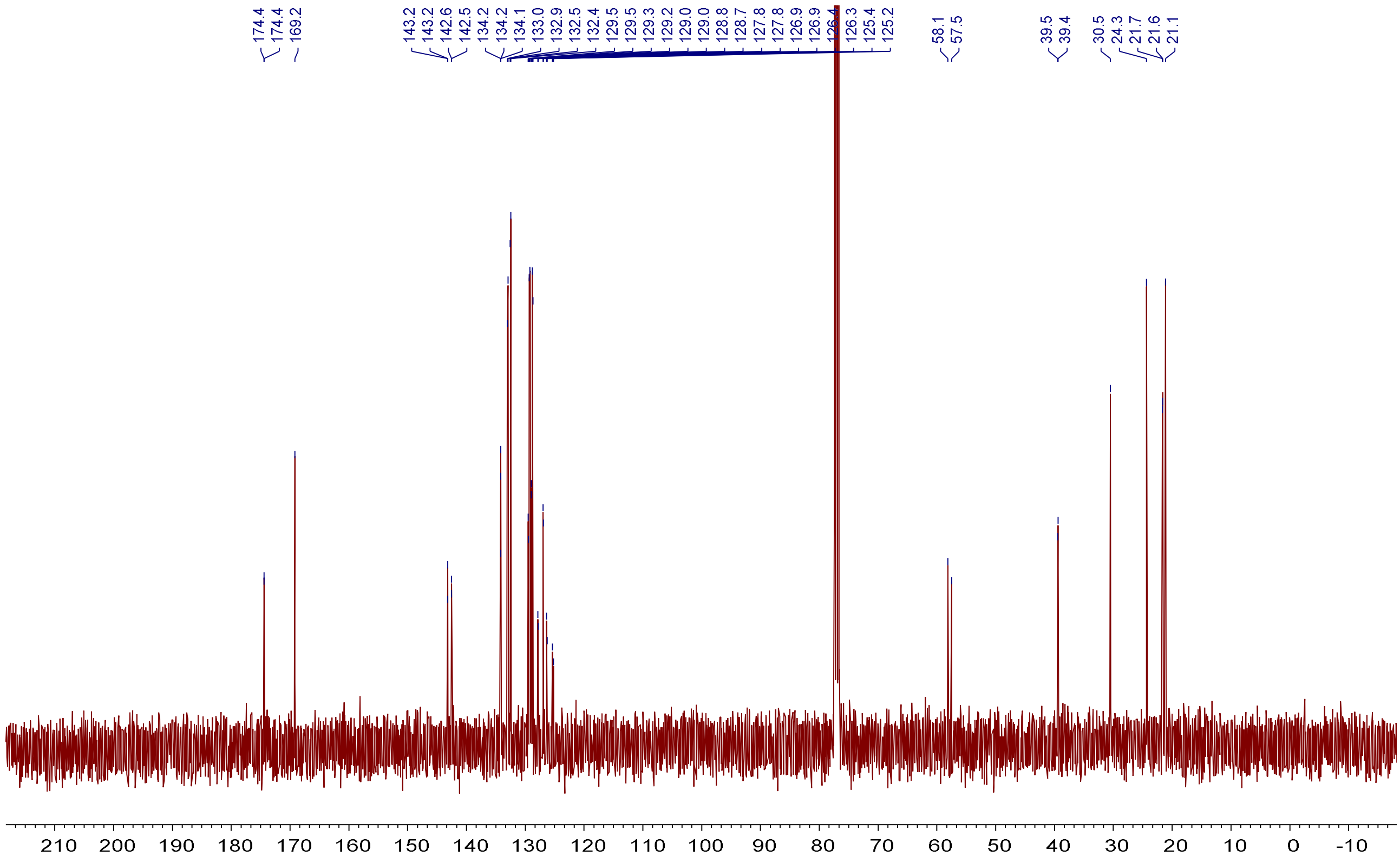

174.4 174.4 169.2
143.2 143.2 142.6 142.5 134.2 134.2 134.1 133.0 132.9 132.5 132.4 129.5 129.5 129.3 129.2 129.0 129.0 128.8 128.7 127.8 126.9 126.9 126.4 126.3 125.4 125.2
58.1 57.5
39.5 39.4
30.5 24.3 21.7 21.6 21.1
210 200 190 180 170 160 150 140 130 120 110 100 90 80 70 60 50 40 30 20 10 0 -10

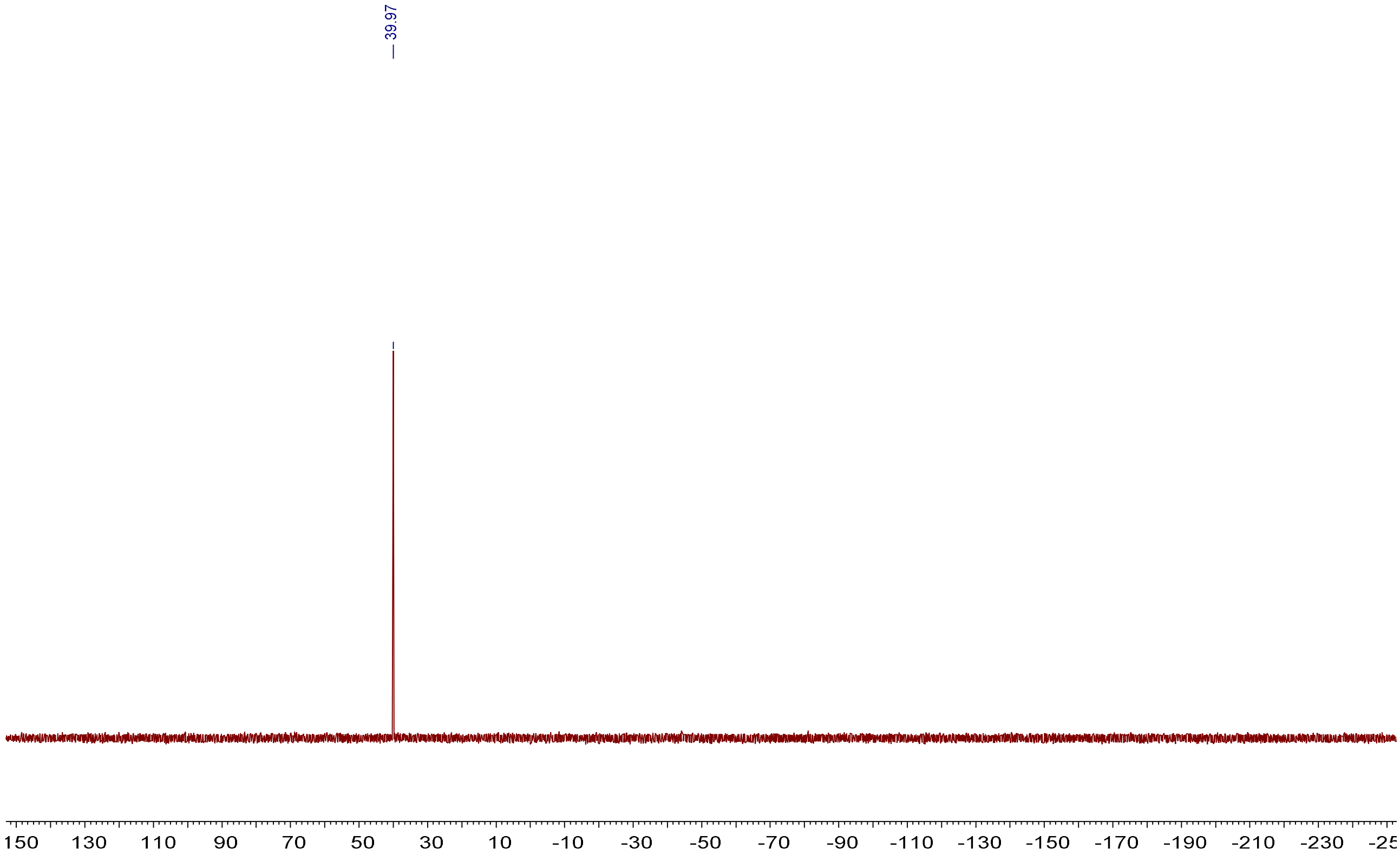
39.97
150 130 110 90 70 50 30 10 -10 -30 -50 -70 -90 -110 -130 -150 -170 -190 -210 -230 -25

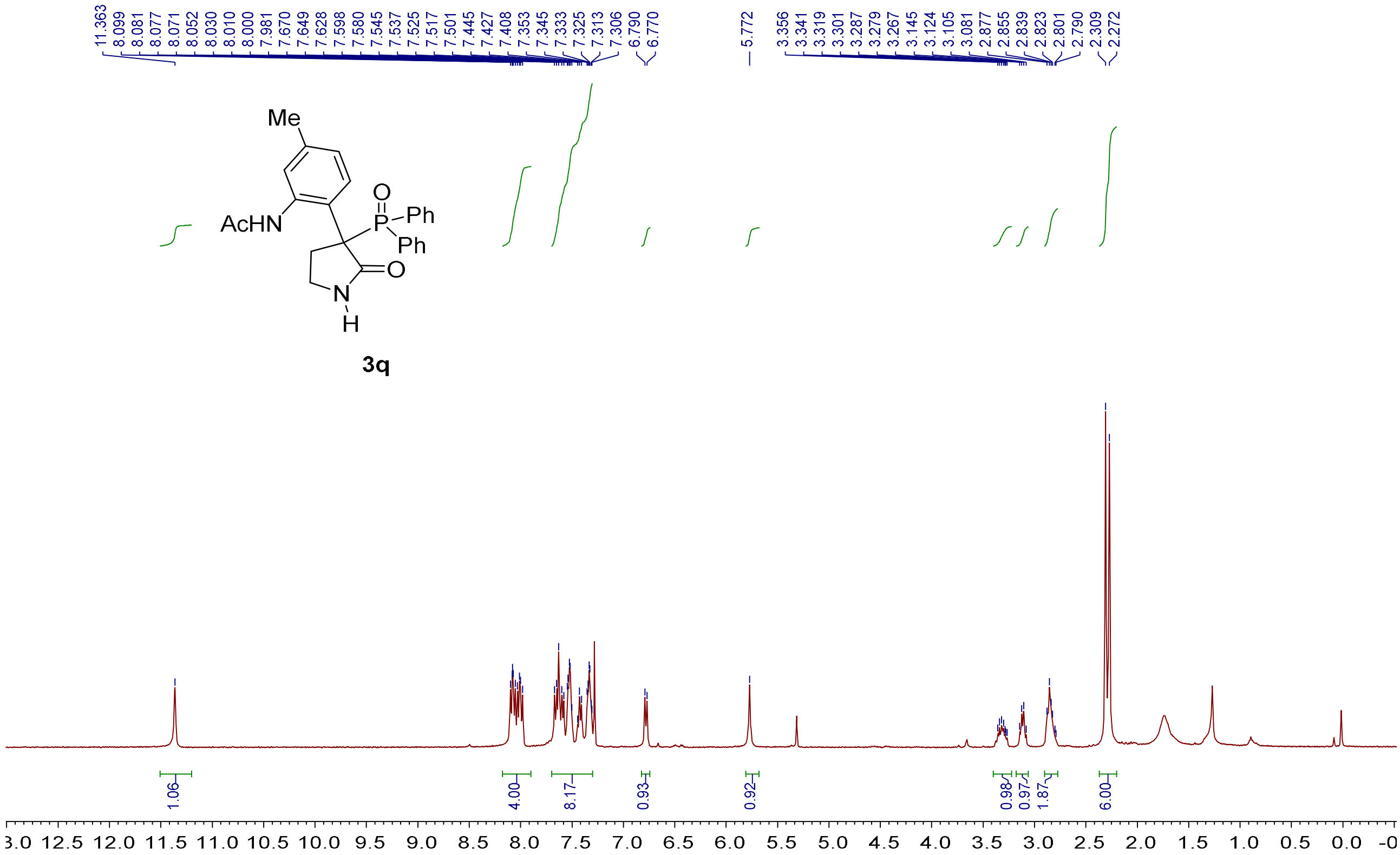
11.363
8.099
8.081
8.077
8.071
8.052
8.030
8.010
8.000
7.981
7.670
7.649
7.628
7.598
7.580
7.545
7.537
7.525
7.517
7.501
7.445
7.427
7.408
7.353
7.345
7.333
7.325
7.313
7.306
6.790
6.770
5.772
3.356
3.341
3.319
3.301
3.287
3.279
3.267
3.145
3.124
3.105
3.081
2.877
2.855
2.839
2.823
2.801
2.790
2.309
2.272
Me
AcHN
O
P
Ph
Ph
O
N
H
3q
1.06
4.00
8.17
0.93
0.92
0.98
0.97
1.87
6.00
13.0 12.5 12.0 11.5 11.0 10.5 10.0 9.5 9.0 8.5 8.0 7.5 7.0 6.5 6.0 5.5 5.0 4.5 4.0 3.5 3.0 2.5 2.0 1.5 1.0 0.5 0.0 -0

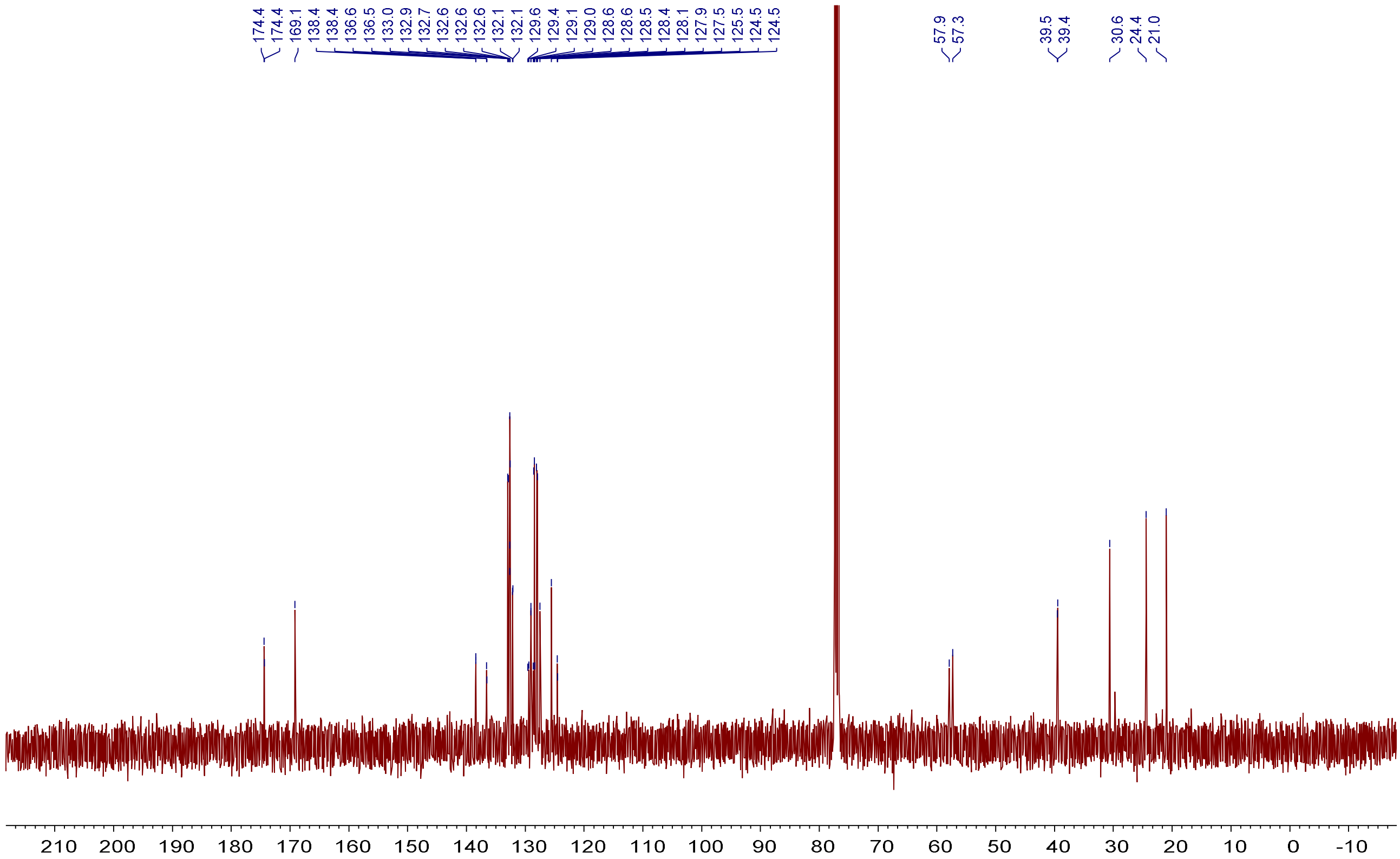
174.4
174.4
169.1
138.4
138.4
136.6
136.5
133.0
132.9
132.7
132.6
132.6
132.6
132.1
132.1
129.6
129.4
129.1
129.0
128.6
128.6
128.5
128.4
128.1
127.9
127.5
125.5
124.5
124.5
57.9
57.3
39.5
39.4
30.6
24.4
21.0
210 200 190 180 170 160 150 140 130 120 110 100 90 80 70 60 50 40 30 20 10 0 -10

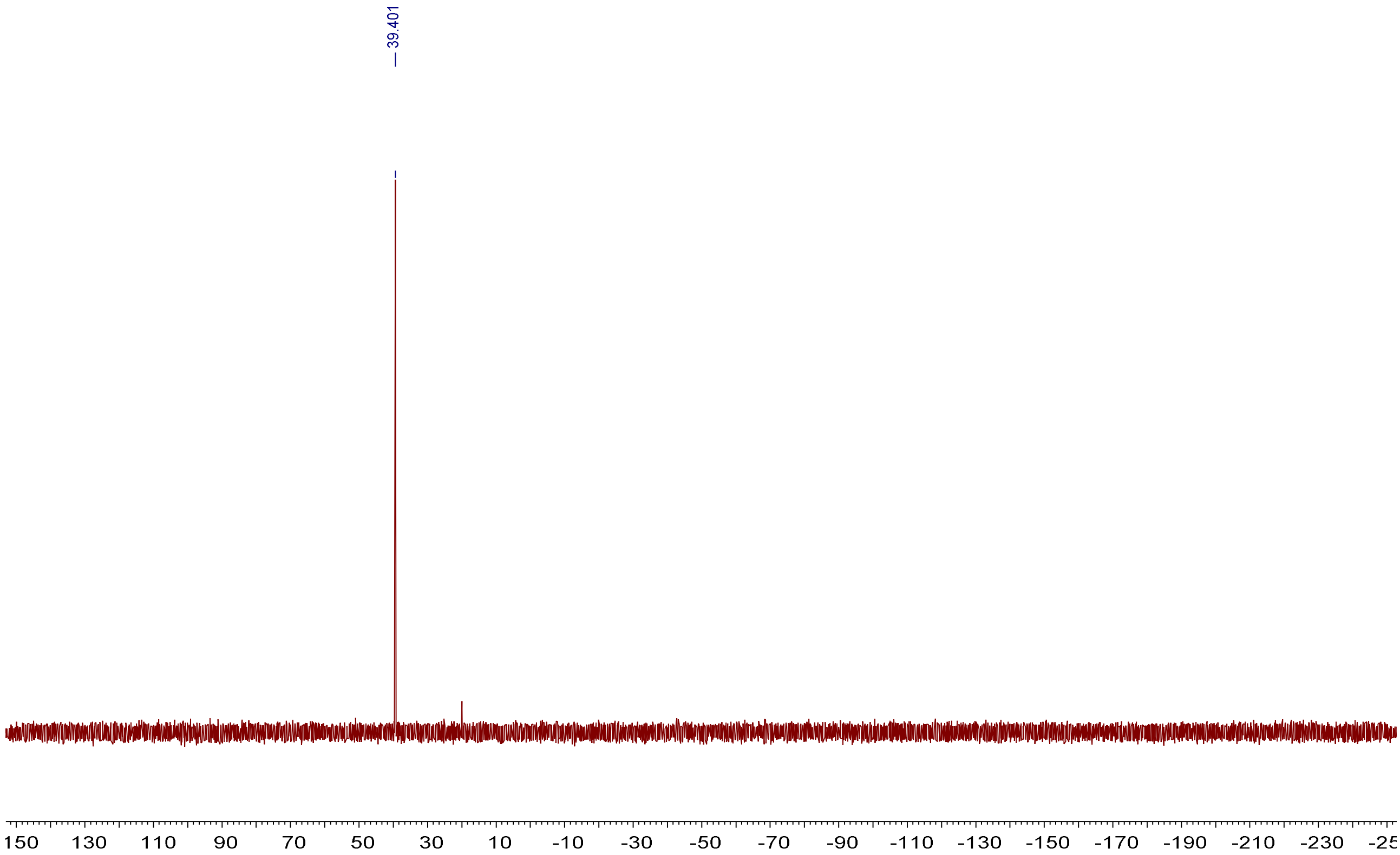
39.401
150 130 110 90 70 50 30 10 -10 -30 -50 -70 -90 -110 -130 -150 -170 -190 -210 -230 -25

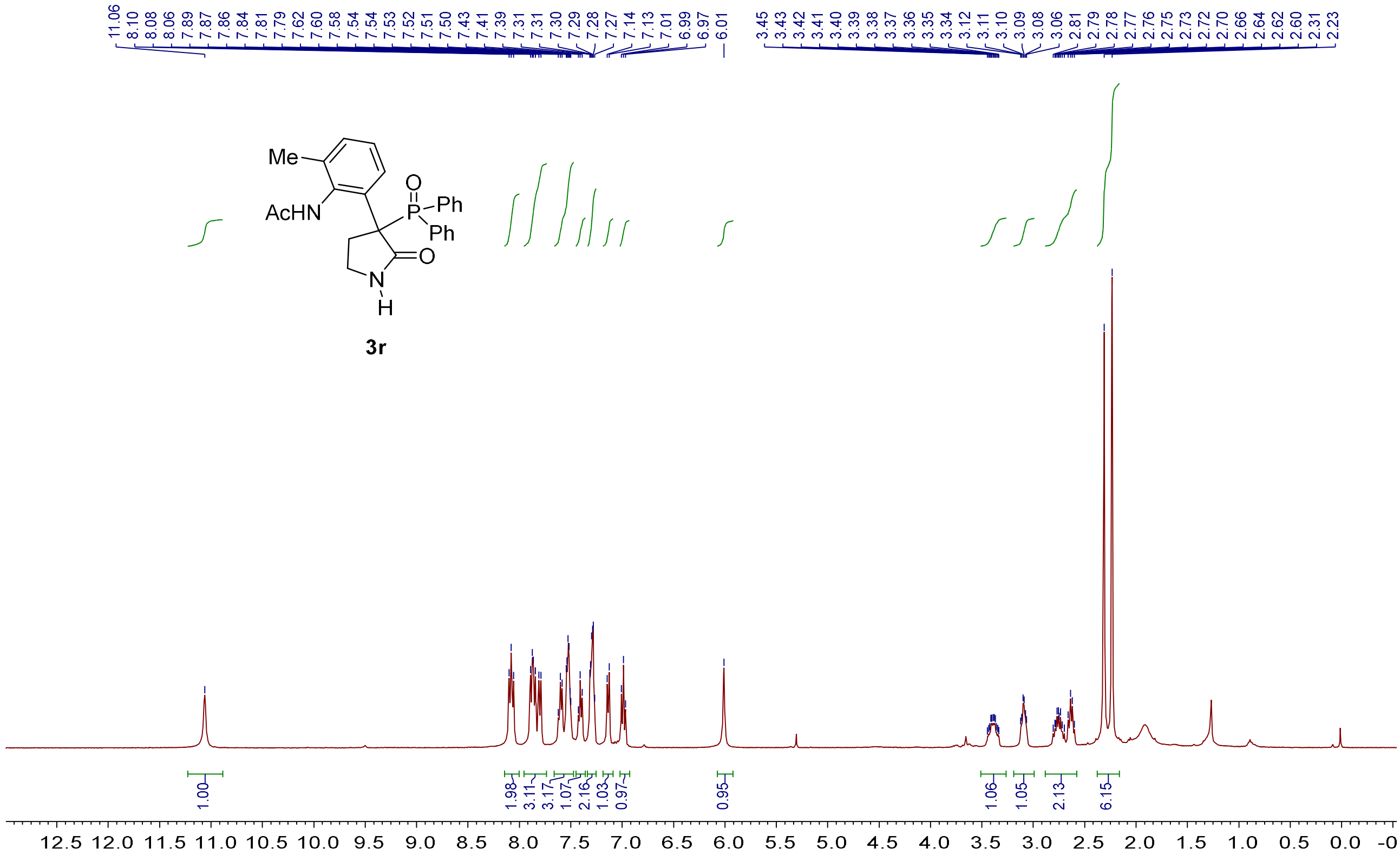
11.06
8.10
8.08
8.06
7.89
7.87
7.86
7.84
7.81
7.79
7.62
7.60
7.58
7.54
7.54
7.53
7.52
7.51
7.50
7.43
7.41
7.39
7.31
7.30
7.29
7.28
7.27
7.14
7.13
7.01
6.99
6.97
6.01
3.45
3.43
3.42
3.41
3.40
3.39
3.38
3.37
3.36
3.35
3.34
3.12
3.11
3.10
3.09
3.08
3.06
2.81
2.79
2.78
2.77
2.76
2.75
2.73
2.72
2.70
2.66
2.64
2.62
2.60
2.31
2.23
Me
AcHN
O
P
Ph
Ph
O
N
H
3r
1.00
1.98
3.11
3.17
1.07
2.16
1.03
0.97
0.95
1.06
1.05
2.13
6.15
12.5 12.0 11.5 11.0 10.5 10.0 9.5 9.0 8.5 8.0 7.5 7.0 6.5 6.0 5.5 5.0 4.5 4.0 3.5 3.0 2.5 2.0 1.5 1.0 0.5 0.0 -0

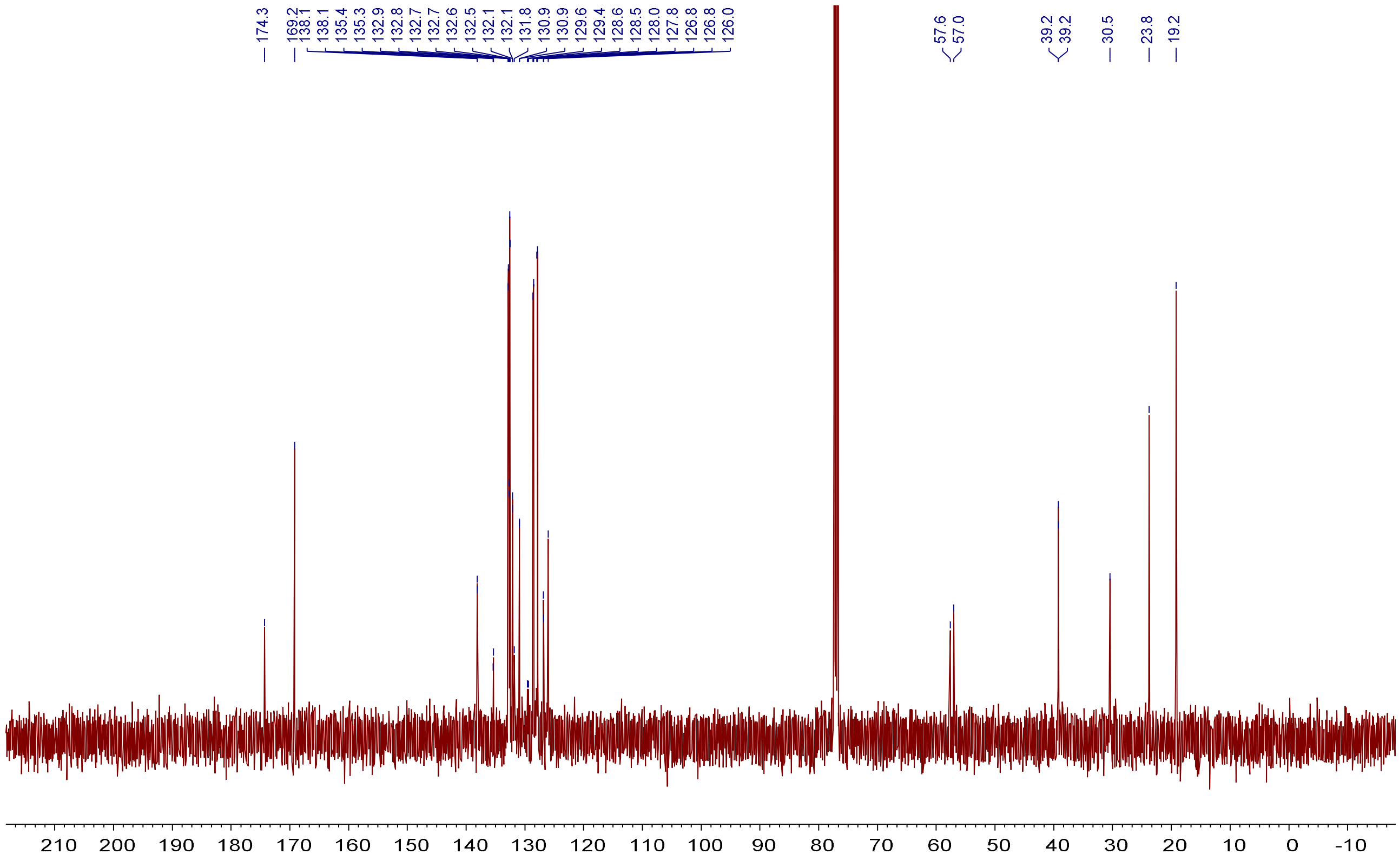
174.3
169.2
138.1
138.1
135.4
135.3
132.9
132.8
132.7
132.7
132.6
132.5
132.1
132.1
131.8
130.9
130.9
129.6
129.4
128.6
128.5
128.0
127.8
126.8
126.0
57.6
57.0
39.2
39.2
30.5
23.8
19.2
210 200 190 180 170 160 150 140 130 120 110 100 90 80 70 60 50 40 30 20 10 0 -10

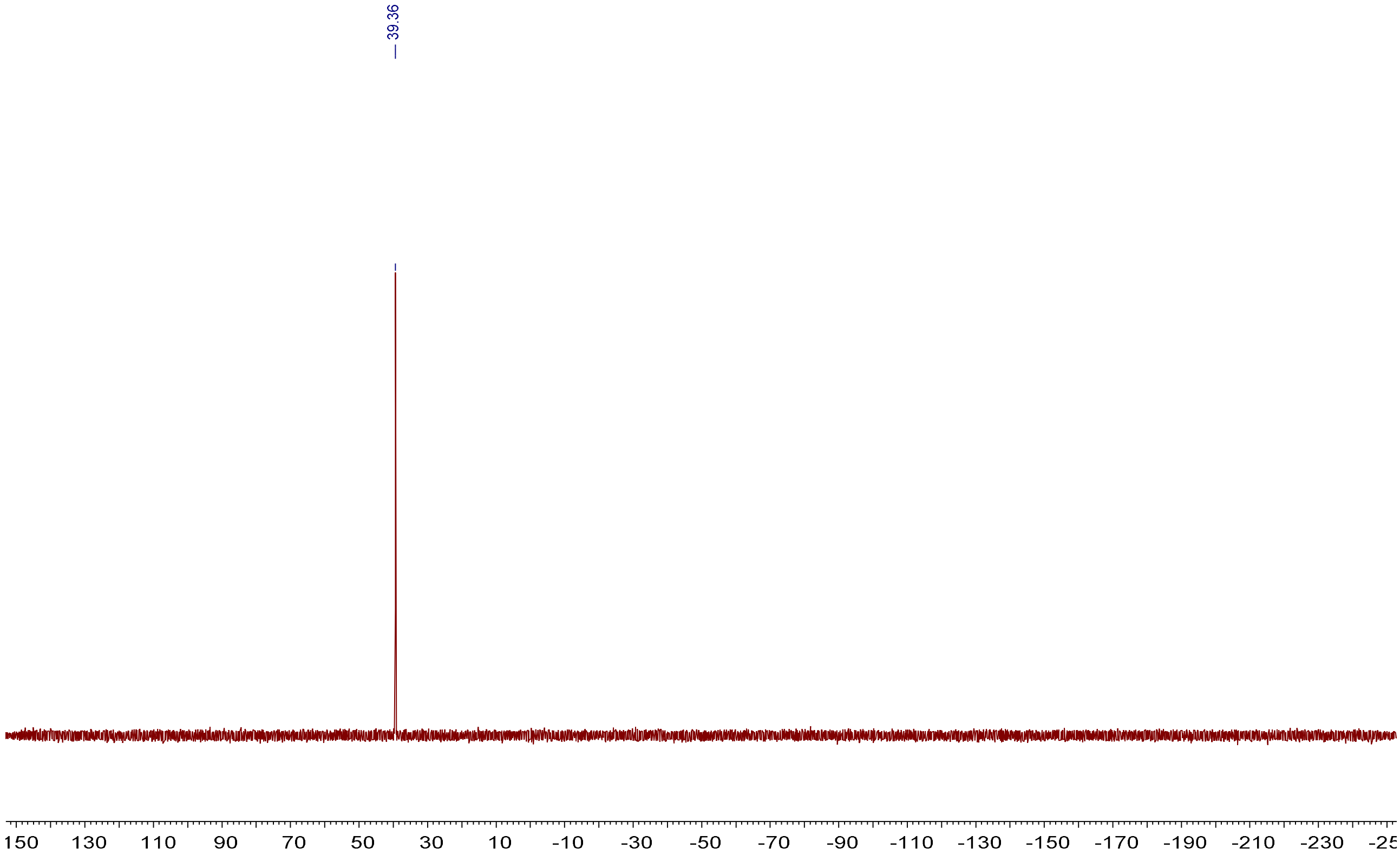
39.36
150 130 110 90 70 50 30 10 -10 -30 -50 -70 -90 -110 -130 -150 -170 -190 -210 -230 -25

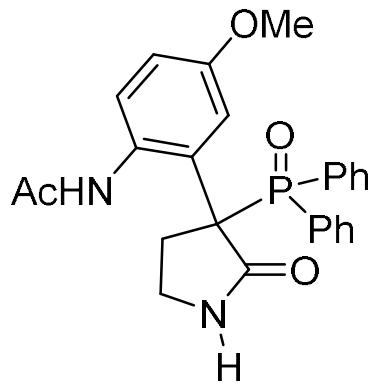
OMe
O
P
Ph
Ph
AcHN
O
N
H

3s

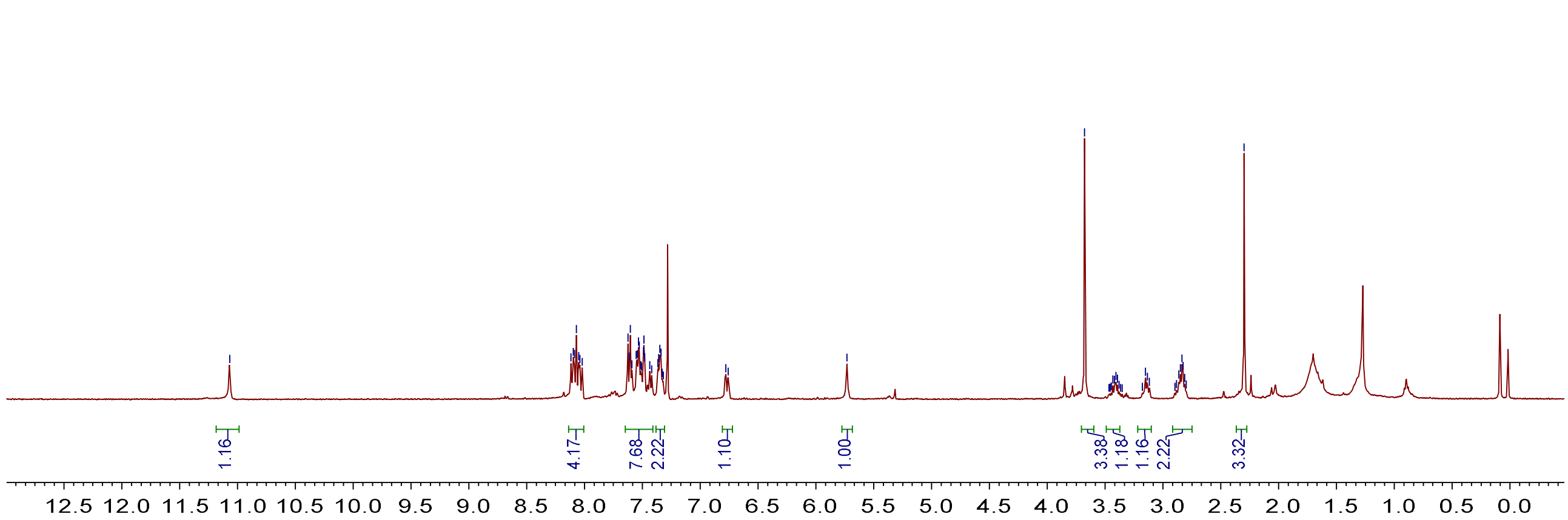
11.07
8.12 8.10 8.09 8.07 8.05 8.04 8.02
7.62 7.61 7.60 7.59 7.55 7.54 7.53 7.53 7.51 7.51 7.49 7.48 7.48 7.44 7.42 7.37 7.36 7.35 7.34 7.33 7.32
6.78 6.76
5.73
3.68 3.46 3.45 3.43 3.42 3.41 3.39 3.38 3.37 3.18 3.15 3.14 3.12
2.90 2.88 2.86 2.85 2.84 2.83 2.81 2.80
1.16
4.17
7.68
2.22
1.10
1.00
3.38
1.18
1.16
2.22
3.32
12.5 12.0 11.5 11.0 10.5 10.0 9.5 9.0 8.5 8.0 7.5 7.0 6.5 6.0 5.5 5.0 4.5 4.0 3.5 3.0 2.5 2.0 1.5 1.0 0.5 0.0

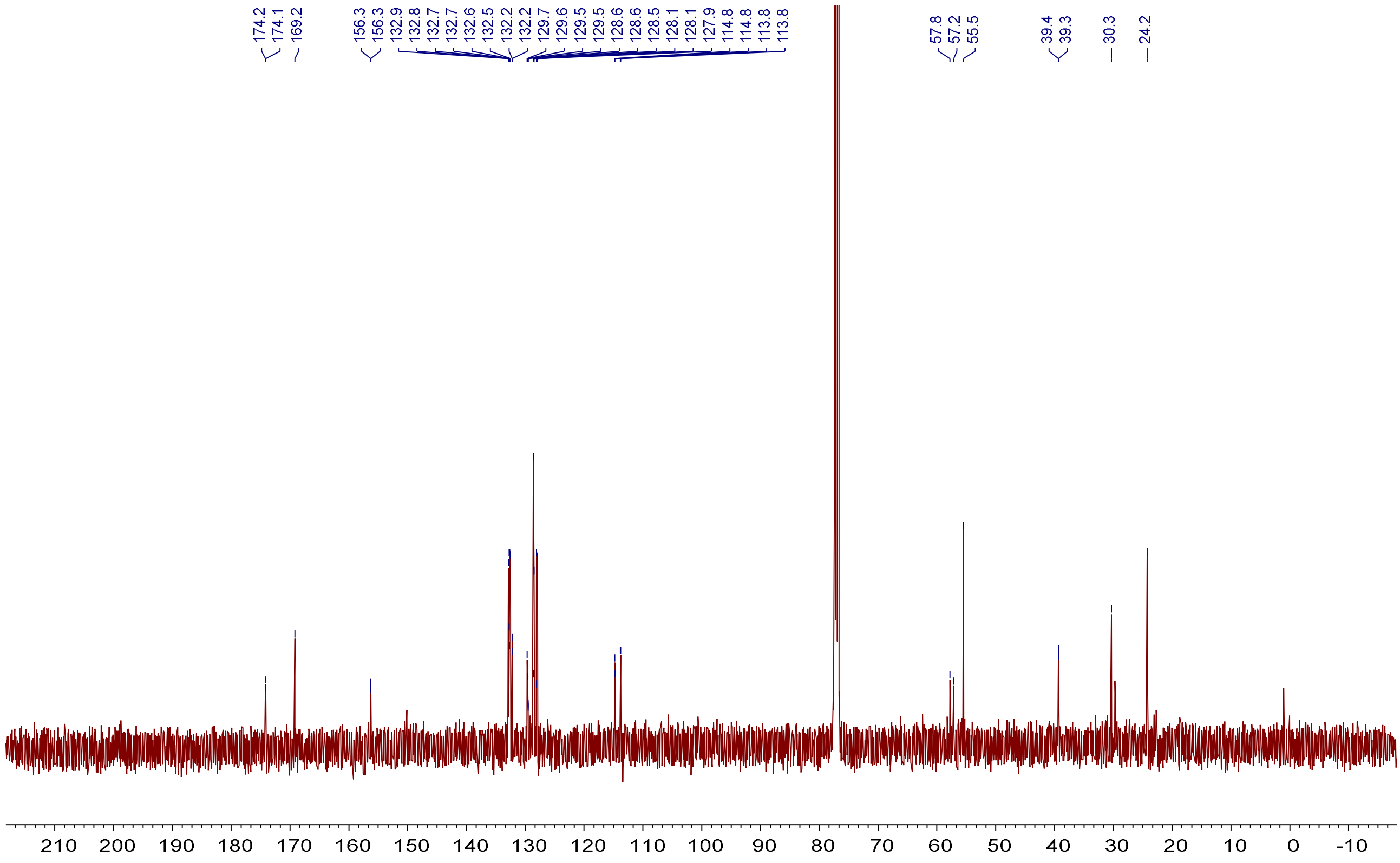
174.2 174.1 169.2
156.3 156.3
132.9 132.8 132.7 132.7 132.6 132.5 132.2 132.2 129.7 129.6 129.5 129.5 128.6 128.5 128.1 128.1 127.9
114.8 114.8 113.8 113.8
57.8 57.2 55.5
39.4 39.3
30.3
24.2
210 200 190 180 170 160 150 140 130 120 110 100 90 80 70 60 50 40 30 20 10 0 -10

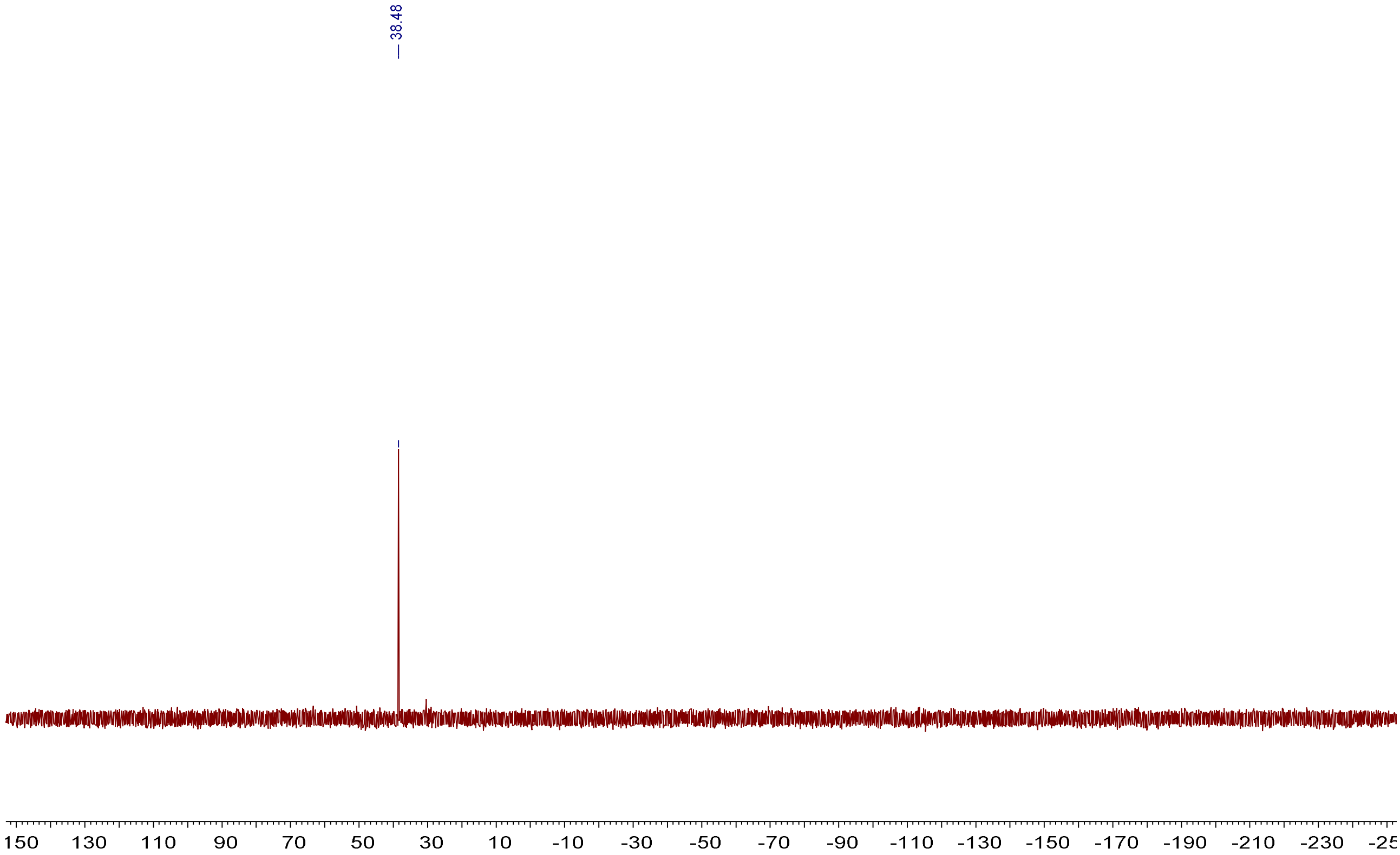
38.48
150
130
110
90
70
50
30
10
-10
-30
-50
-70
-90
-110
-130
-150
-170
-190
-210
-230

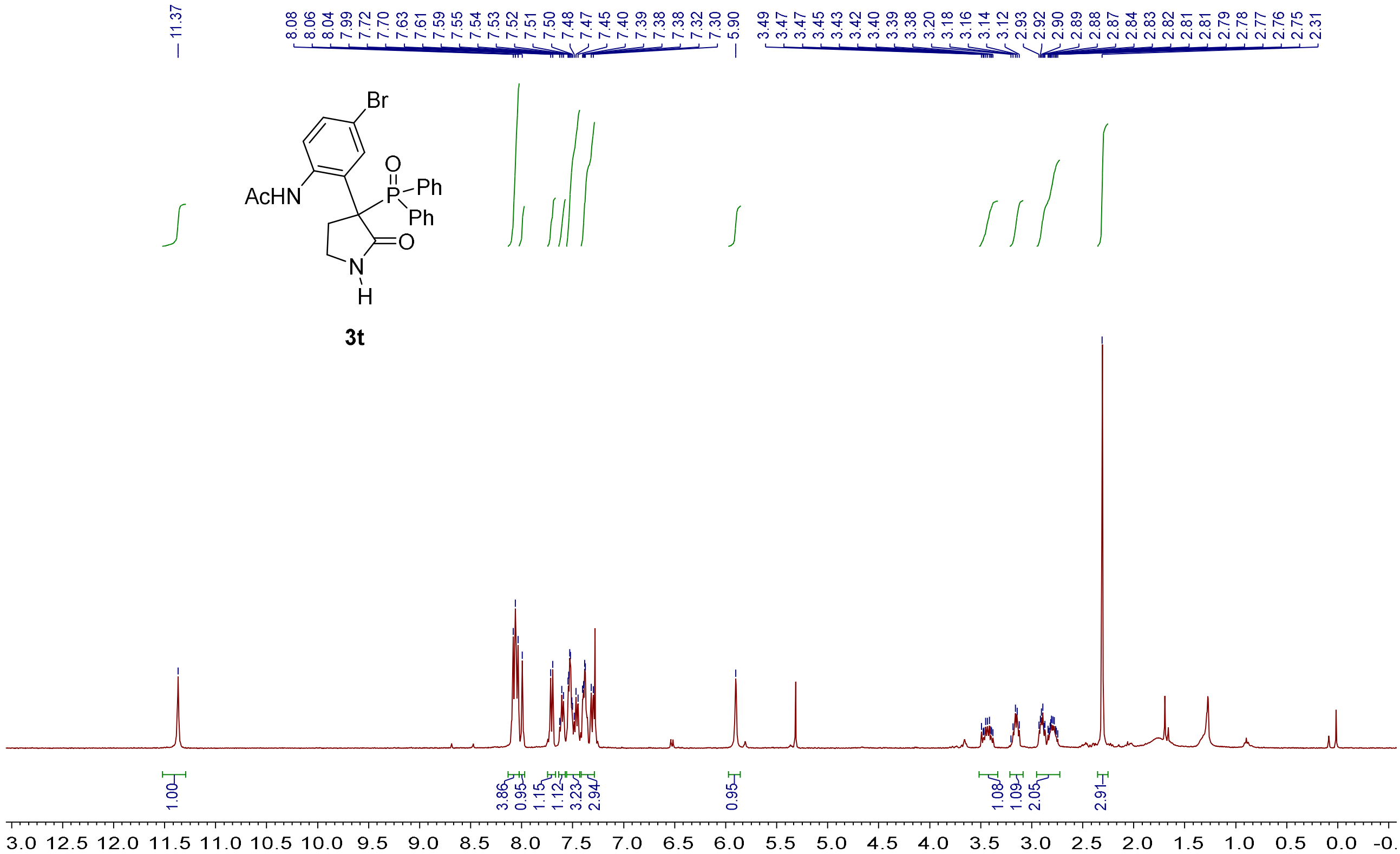

3t

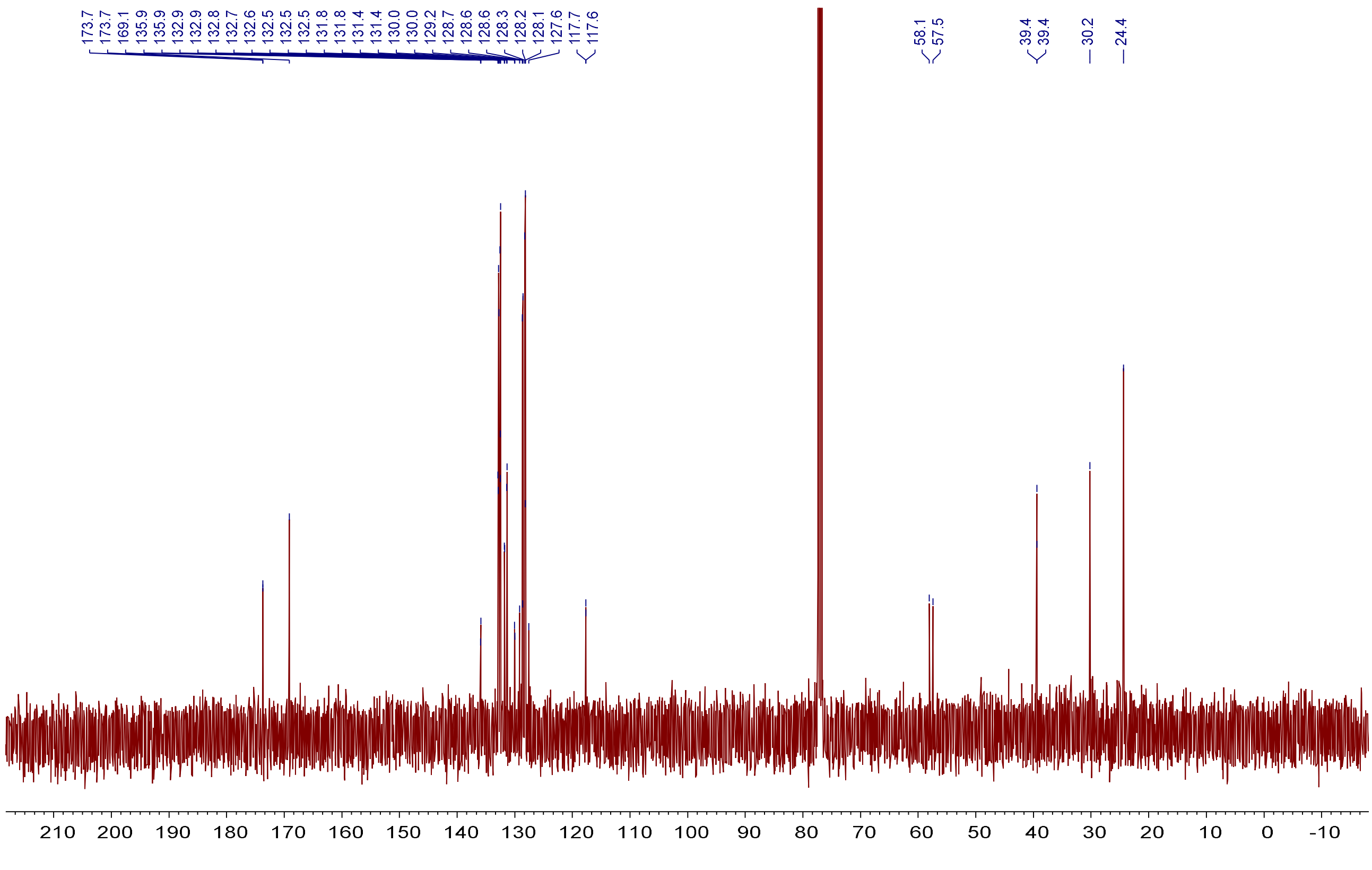

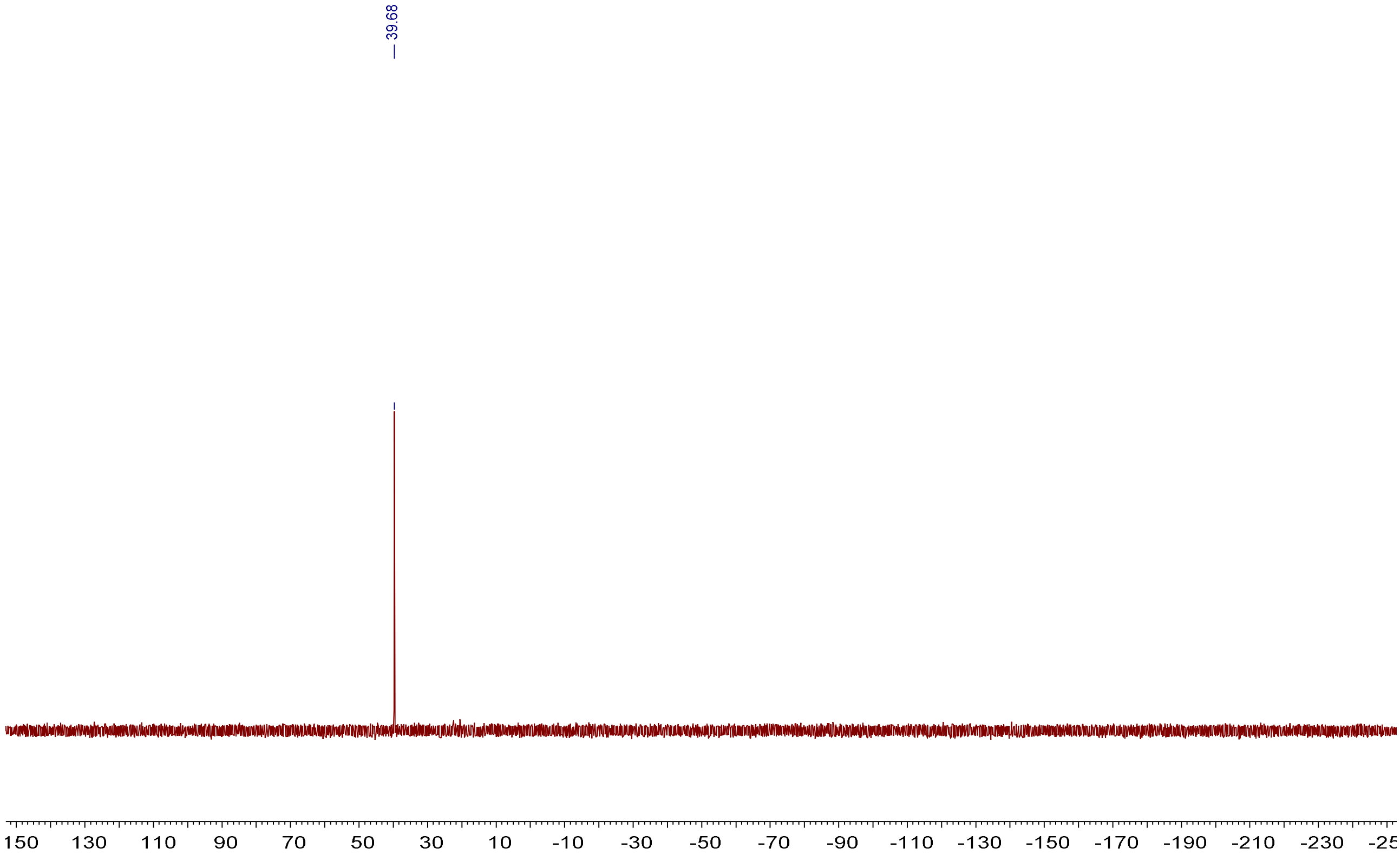

39.68
150 130 110 90 70 50 30 10 -10 -30 -50 -70 -90 -110 -130 -150 -170 -190 -210 -230

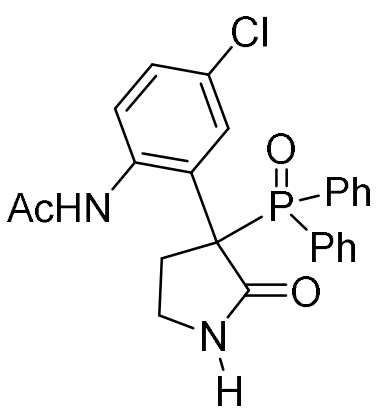
Cl
AcHN
O
P
Ph
Ph
O
N
H

3u

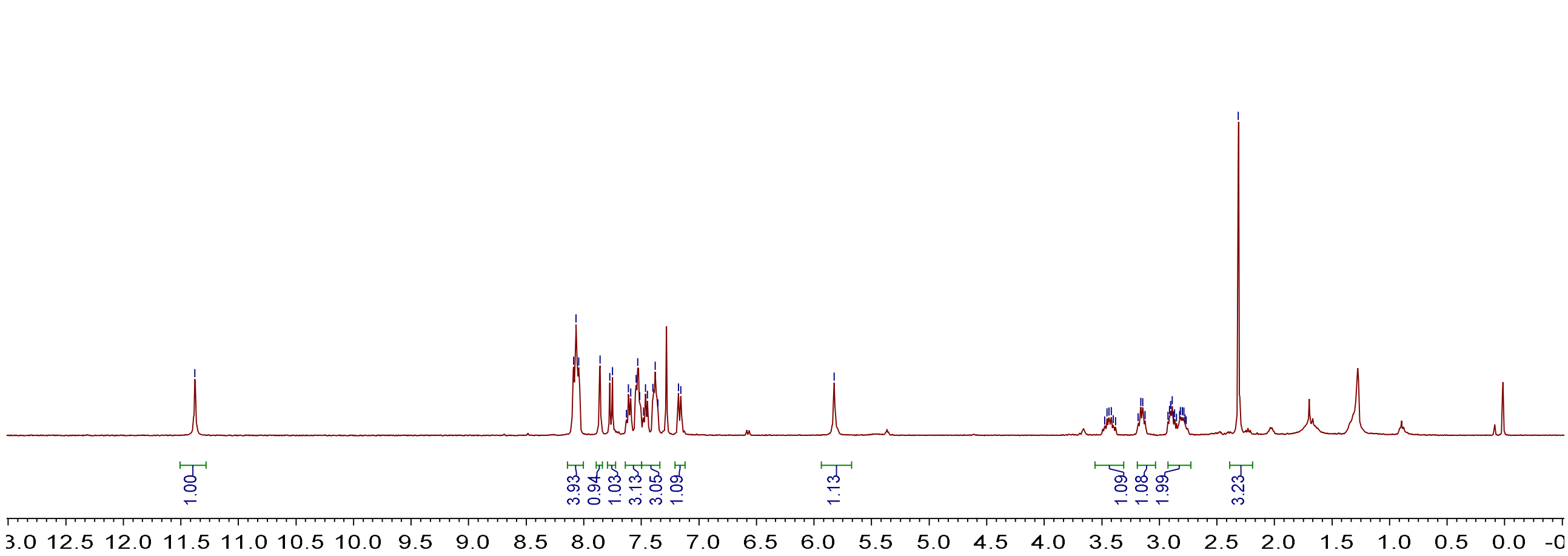
11.38
8.09
8.07
8.05
7.86
7.77
7.75
7.63
7.61
7.59
7.54
7.53
7.51
7.48
7.46
7.45
7.40
7.38
7.35
7.18
7.16
5.83
3.47
3.46
3.44
3.42
3.40
3.38
3.18
3.16
3.14
3.12
2.92
2.91
2.90
2.89
2.88
2.87
2.85
2.83
2.82
2.80
2.79
2.78
2.77
2.31
1.00
3.93
0.94
1.03
3.13
3.05
1.09
1.13
1.09
1.08
1.99
3.23
3.0 12.5 12.0 11.5 11.0 10.5 10.0 9.5 9.0 8.5 8.0 7.5 7.0 6.5 6.0 5.5 5.0 4.5 4.0 3.5 3.0 2.5 2.0 1.5 1.0 0.5 0.0 -0

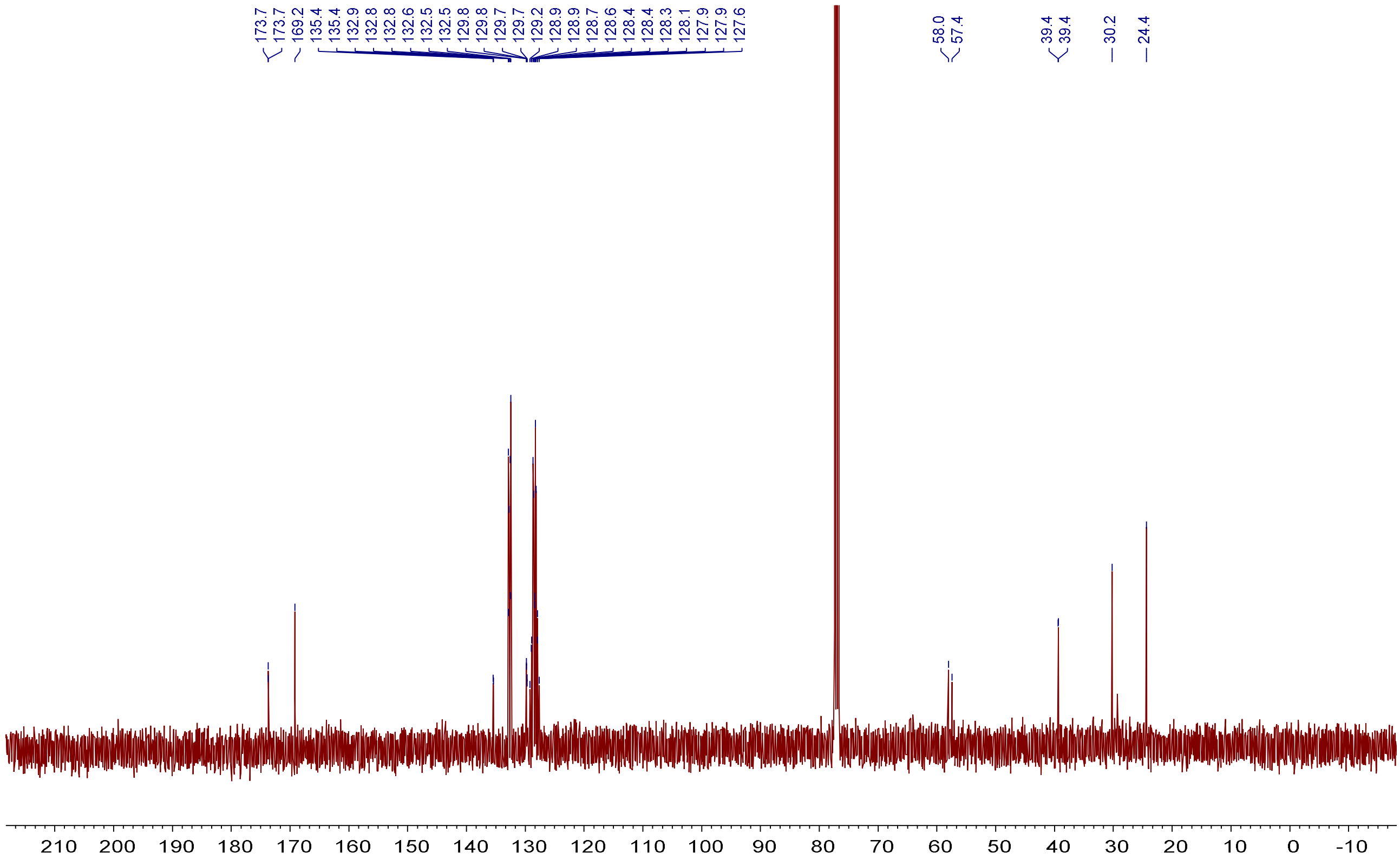
173.7
173.7
169.2
135.4
132.9
132.8
132.8
132.6
132.5
132.5
129.8
129.8
129.7
129.7
129.2
128.9
128.9
128.7
128.6
128.4
128.4
128.3
128.1
127.9
127.6
58.0
57.4
39.4
39.4
30.2
24.4
210 200 190 180 170 160 150 140 130 120 110 100 90 80 70 60 50 40 30 20 10 0 -10

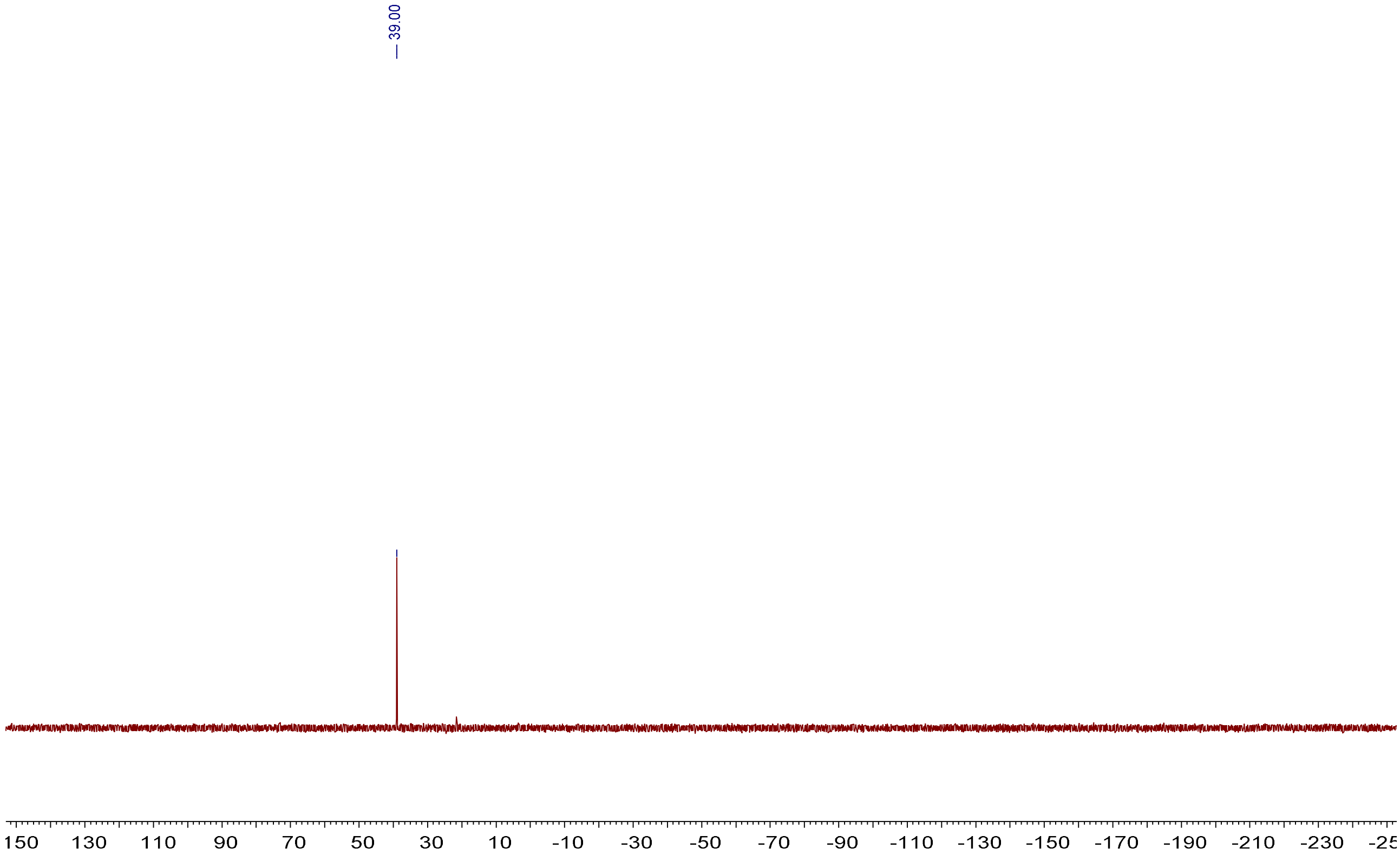
39.00
150 130 110 90 70 50 30 10 -10 -30 -50 -70 -90 -110 -130 -150 -170 -190 -210 -230

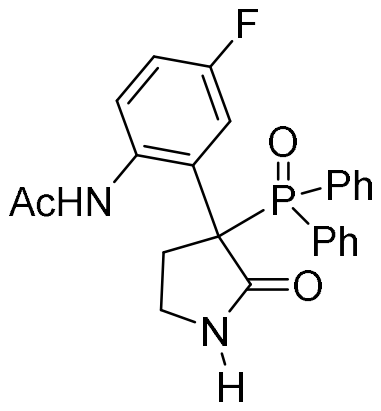

3v

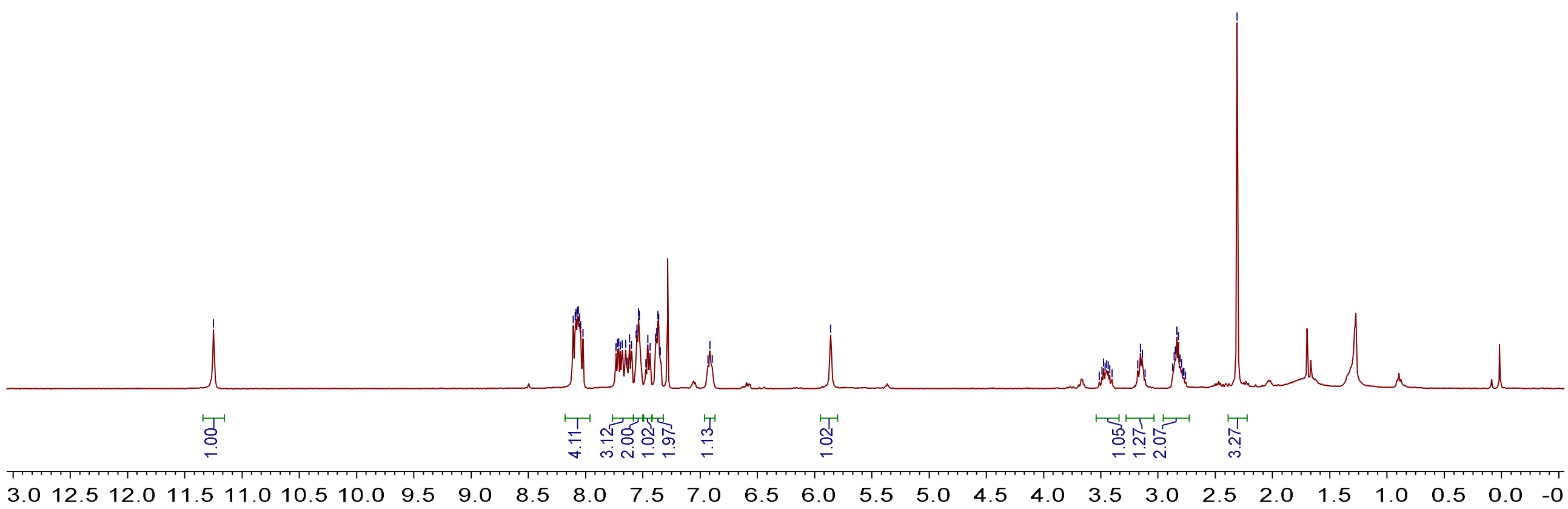

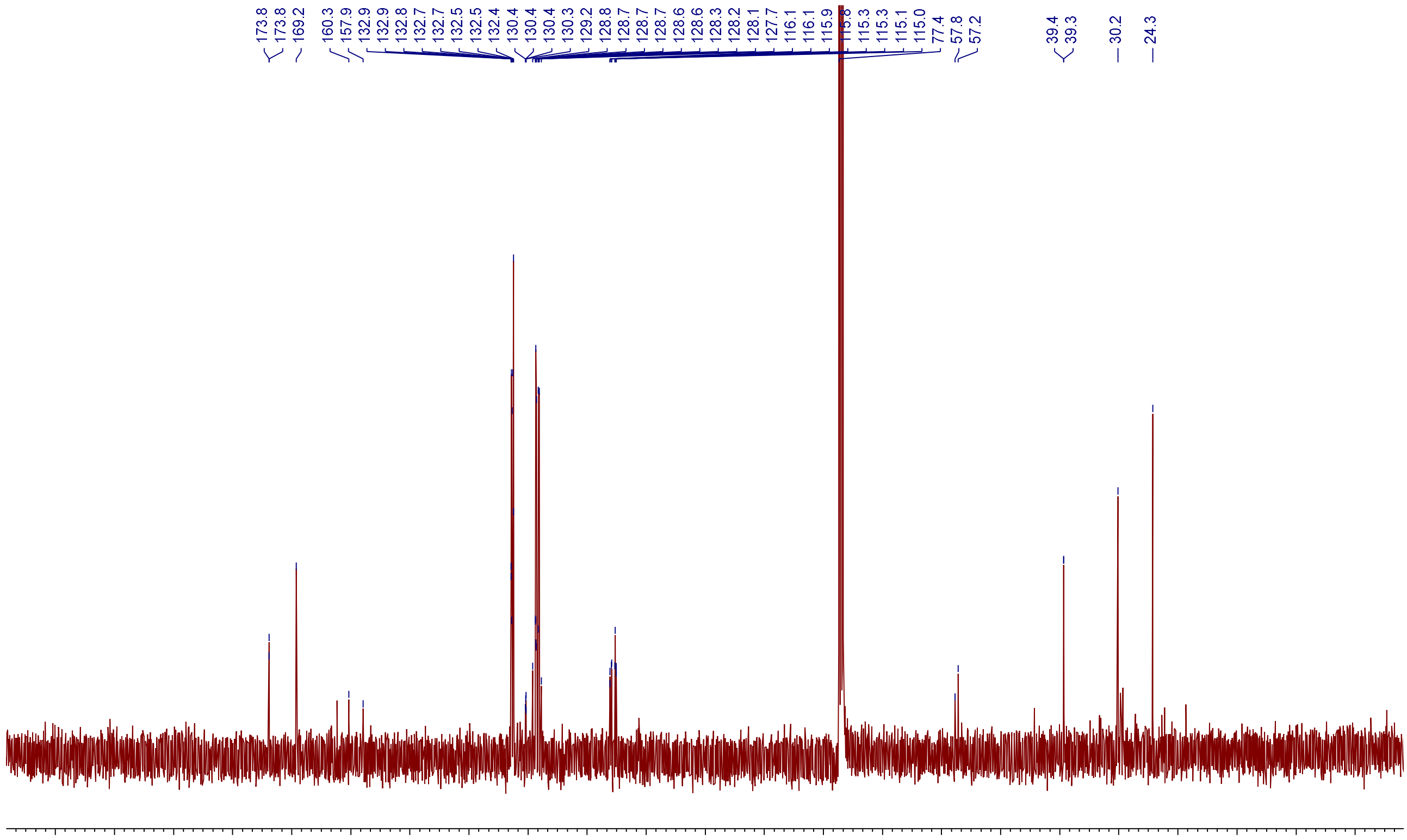

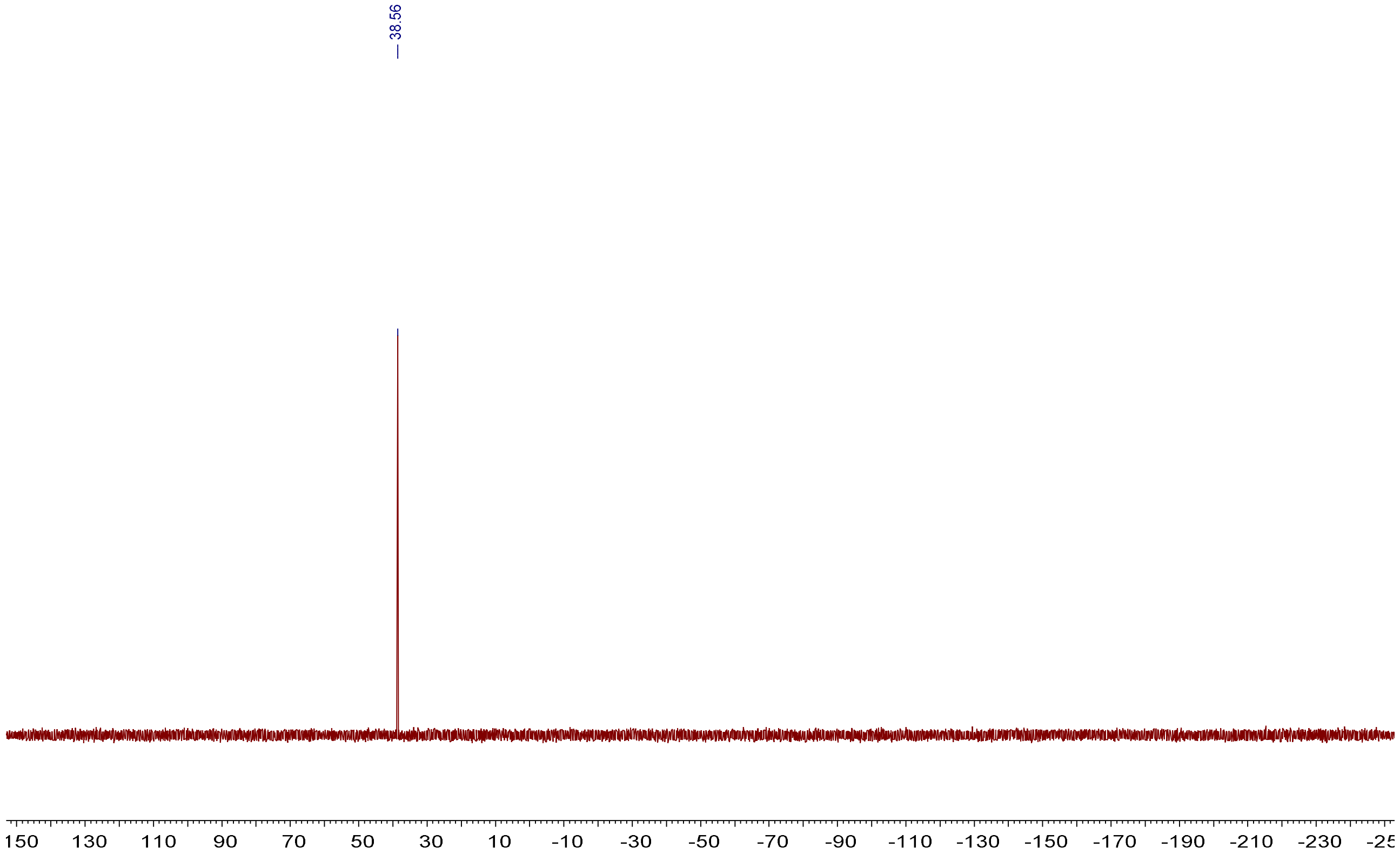
38.56
150 130 110 90 70 50 30 10 -10 -30 -50 -70 -90 -110 -130 -150 -170 -190 -210 -230

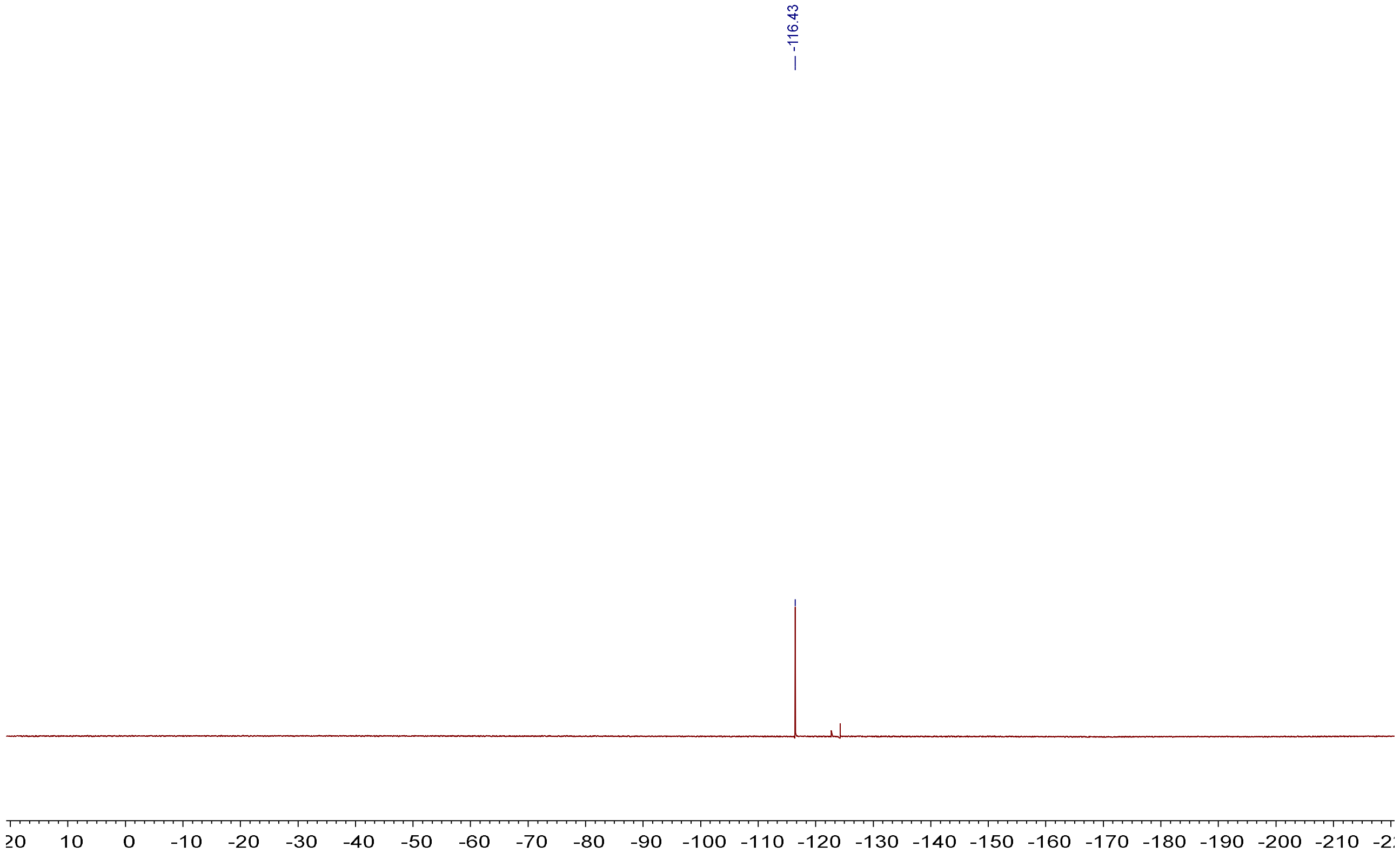
-116.43
20 10 0 -10 -20 -30 -40 -50 -60 -70 -80 -90 -100 -110 -120 -130 -140 -150 -160 -170 -180 -190 -200 -210

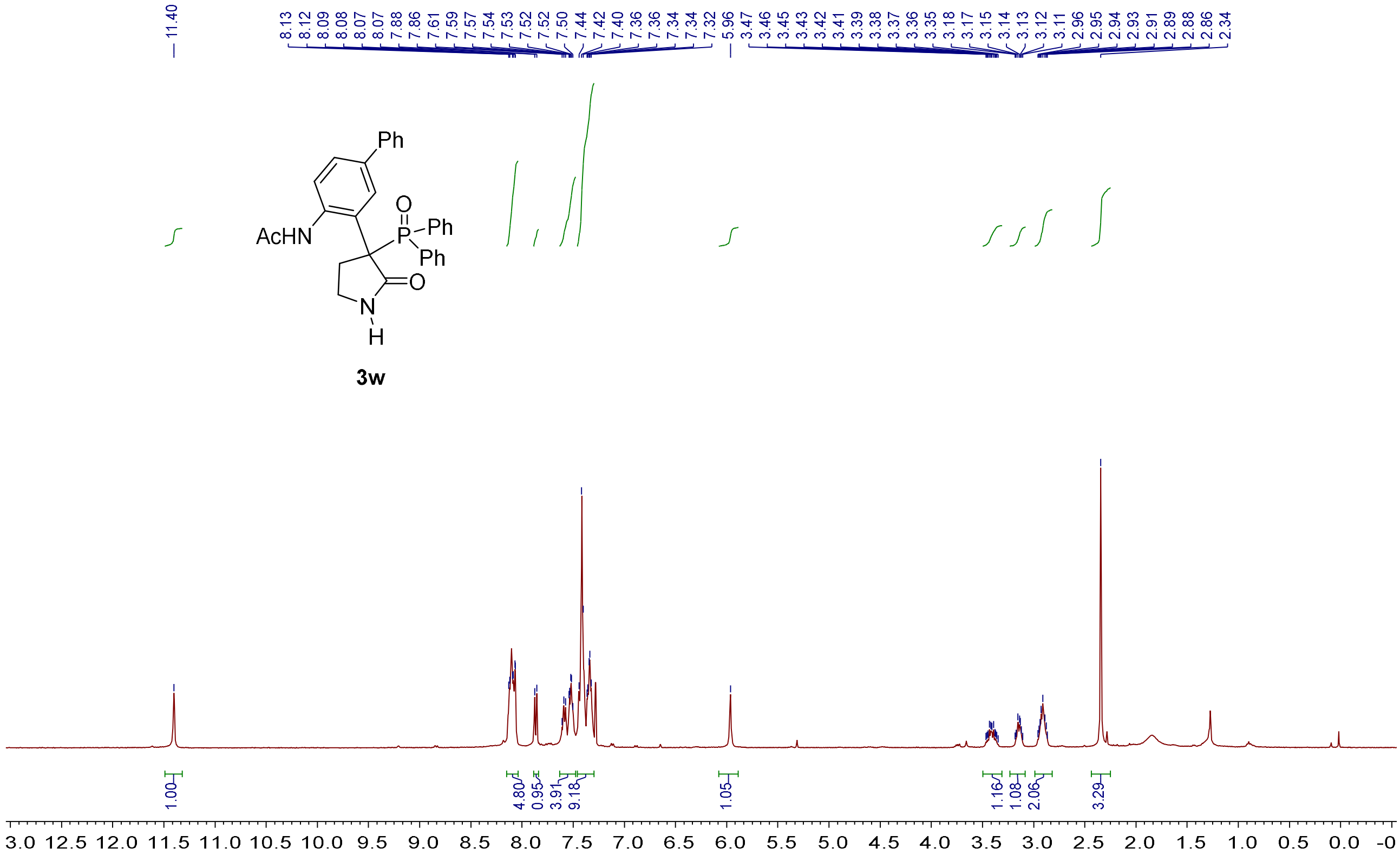

11.40
8.13 8.12 8.09 8.08 8.07 8.07 7.88 7.86 7.61 7.59 7.57 7.54 7.53 7.52 7.52 7.50 7.44 7.42 7.40 7.36 7.36 7.34 7.34 7.32
5.96
3.47 3.46 3.45 3.43 3.42 3.41 3.39 3.38 3.37 3.36 3.35 3.18 3.17 3.15 3.14 3.13 3.12 3.11 2.96 2.95 2.94 2.93 2.91 2.89 2.88 2.86 2.34
Ph
O
P
Ph
Ph
AcHN
O
N
H
3w
1.00
4.80
0.95
3.91
9.18
1.05
1.16
1.08
2.06
3.29
3.0 12.5 12.0 11.5 11.0 10.5 10.0 9.5 9.0 8.5 8.0 7.5 7.0 6.5 6.0 5.5 5.0 4.5 4.0 3.5 3.0 2.5 2.0 1.5 1.0 0.5 0.0 -0

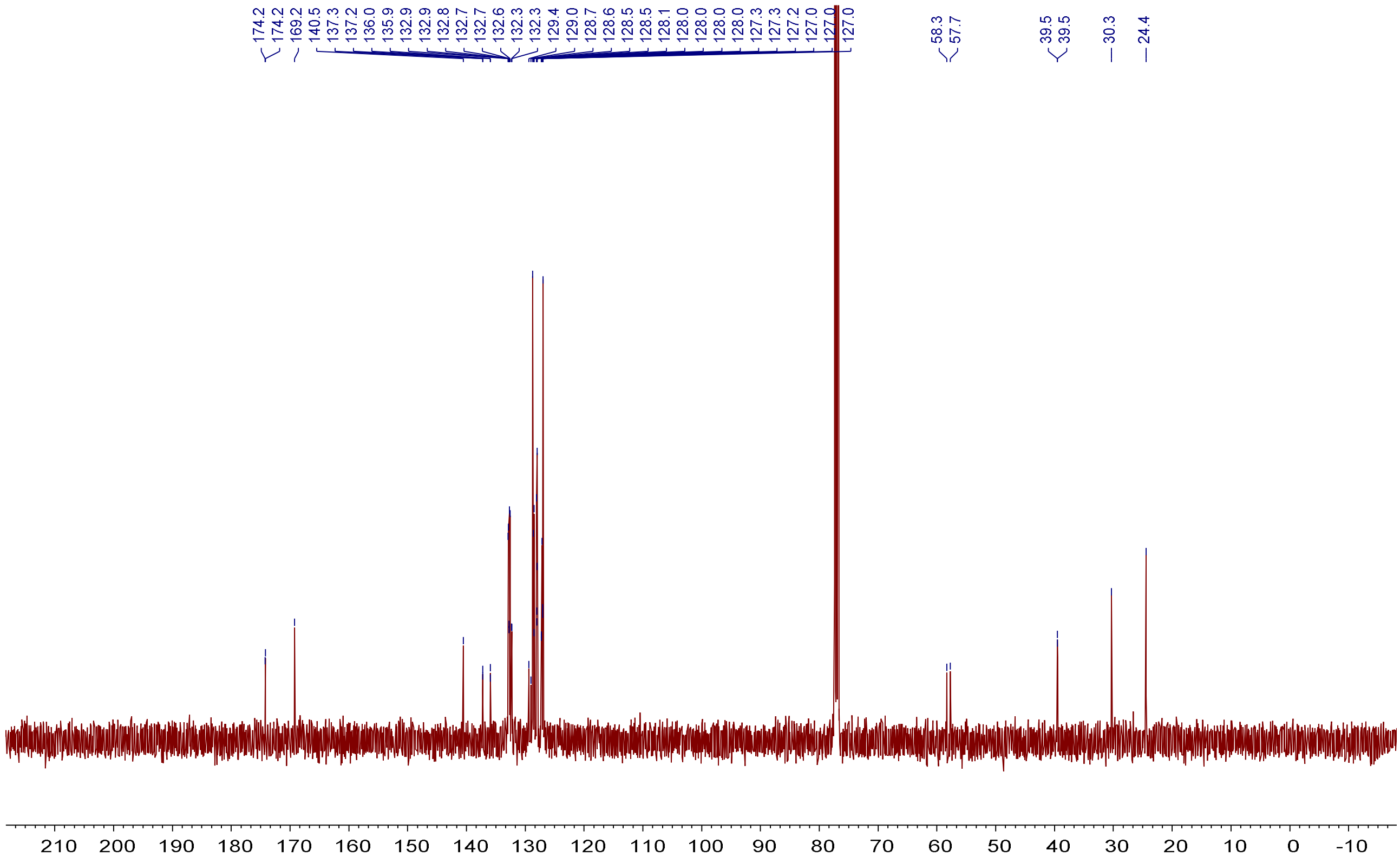

174.2 174.2 169.2 140.5 137.3 137.2 136.0 135.9 132.9 132.9 132.8 132.7 132.7 132.6 132.3 132.3 129.4 129.0 128.7 128.6 128.5 128.5 128.1 128.0 128.0 128.0 128.0 127.3 127.3 127.2 127.0 127.0 127.0
58.3 57.7
39.5 39.5
30.3
24.4
210 200 190 180 170 160 150 140 130 120 110 100 90 80 70 60 50 40 30 20 10 0 -10

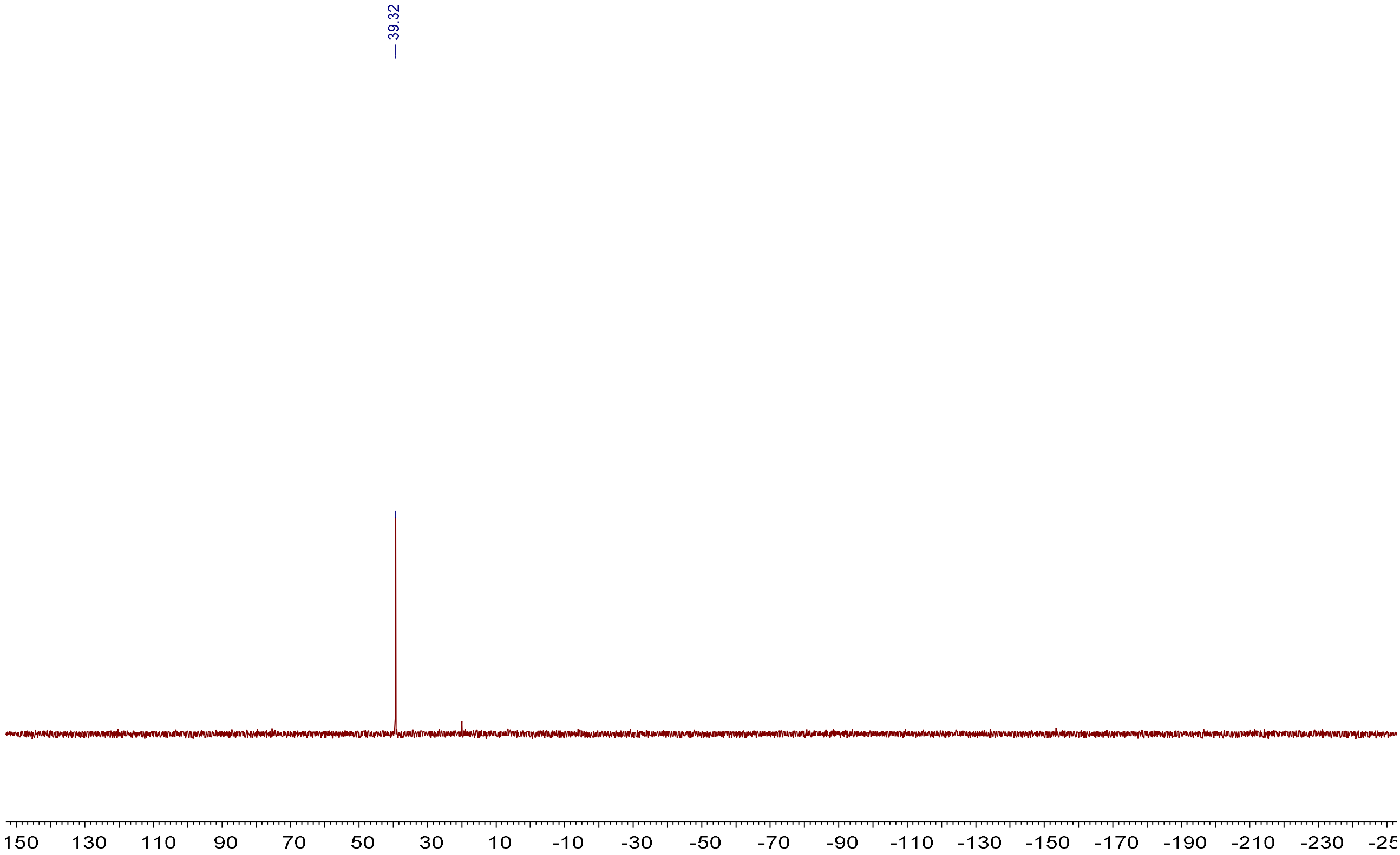
39.32
150 130 110 90 70 50 30 10 -10 -30 -50 -70 -90 -110 -130 -150 -170 -190 -210 -230

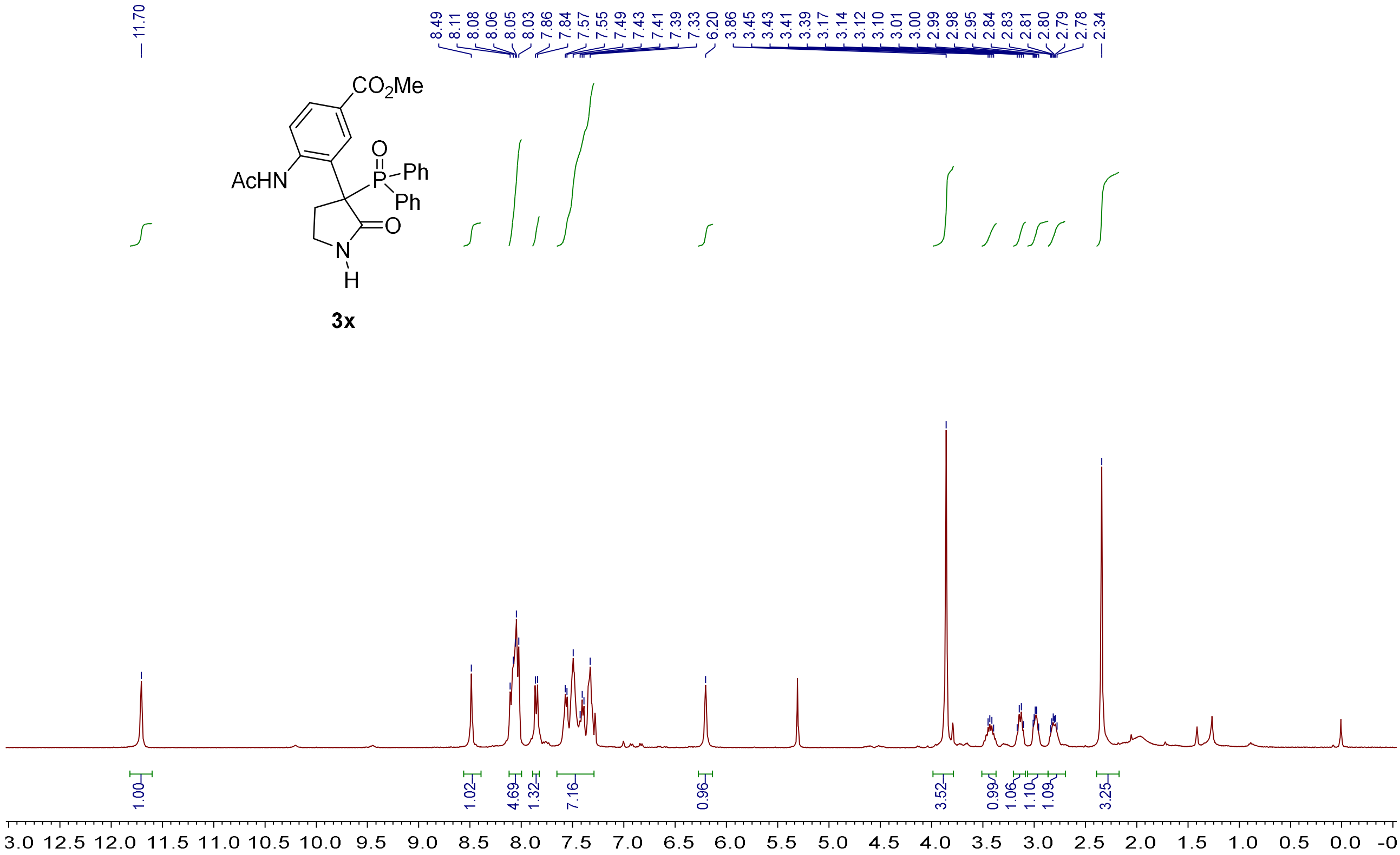
11.70
8.49 8.11 8.08 8.06 8.05 8.03 7.86 7.84 7.57 7.55 7.49 7.43 7.41 7.39 7.33 6.20 3.86 3.45 3.43 3.41 3.39 3.17 3.14 3.12 3.10 3.01 3.00 2.99 2.98 2.95 2.84 2.83 2.81 2.80 2.79 2.78 2.34
CO_2Me
AcHN
O
P
Ph
Ph
O
N
H
3x
1.00 1.02 4.69 1.32 7.16 0.96 3.52 0.99 1.06 1.10 1.09 3.25
3.0 12.5 12.0 11.5 11.0 10.5 10.0 9.5 9.0 8.5 8.0 7.5 7.0 6.5 6.0 5.5 5.0 4.5 4.0 3.5 3.0 2.5 2.0 1.5 1.0 0.5 0.0 -0

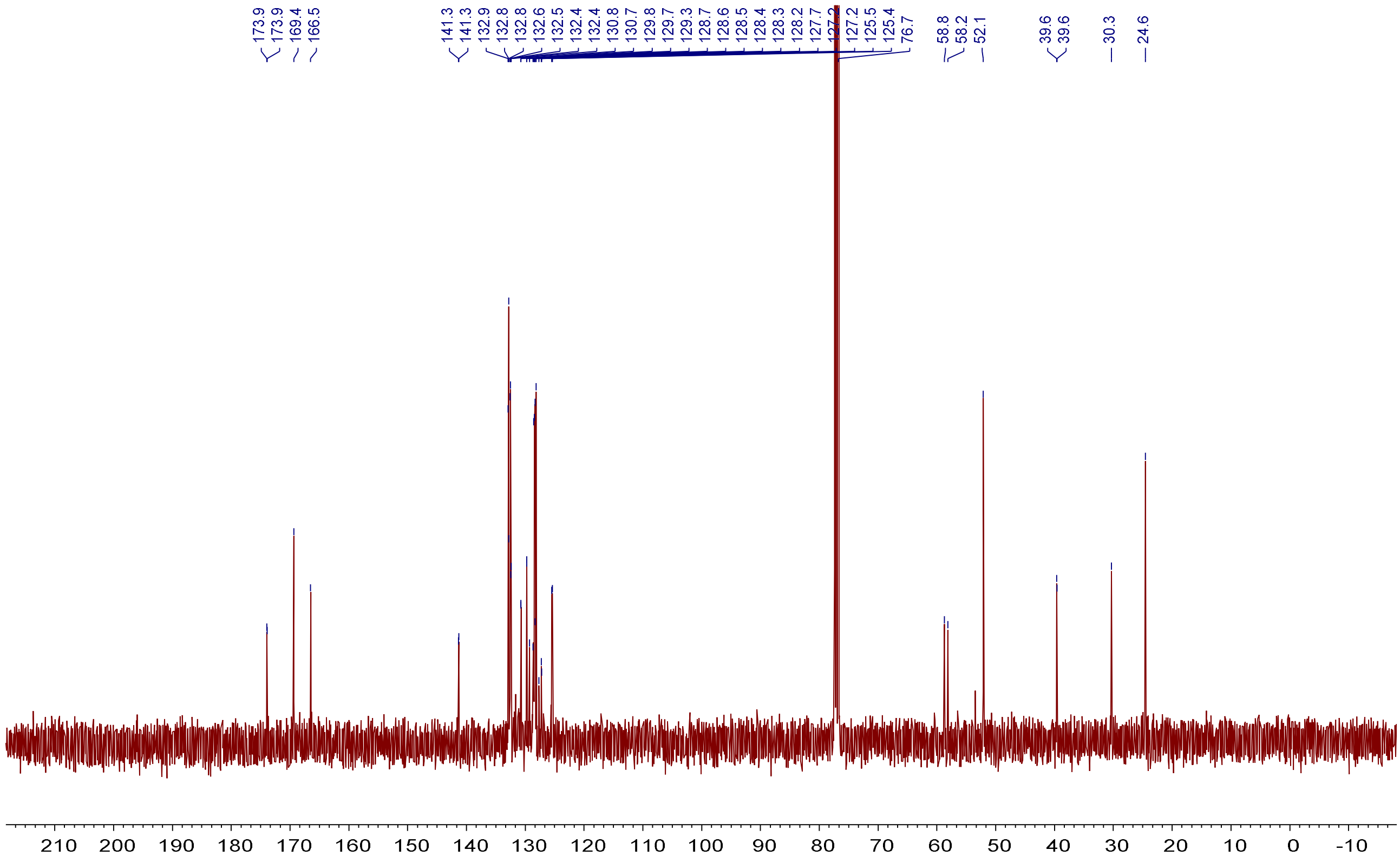
173.9 173.9 169.4 166.5
141.3 141.3 132.9 132.8 132.8 132.6 132.5 132.4 132.4 130.8 130.7 129.8 129.7 129.3 129.3 128.7 128.6 128.5 128.4 128.3 128.2 127.7 127.2 125.5 125.4 76.7
58.8 58.2 52.1
39.6 39.6
30.3
24.6
210 200 190 180 170 160 150 140 130 120 110 100 90 80 70 60 50 40 30 20 10 0 -10

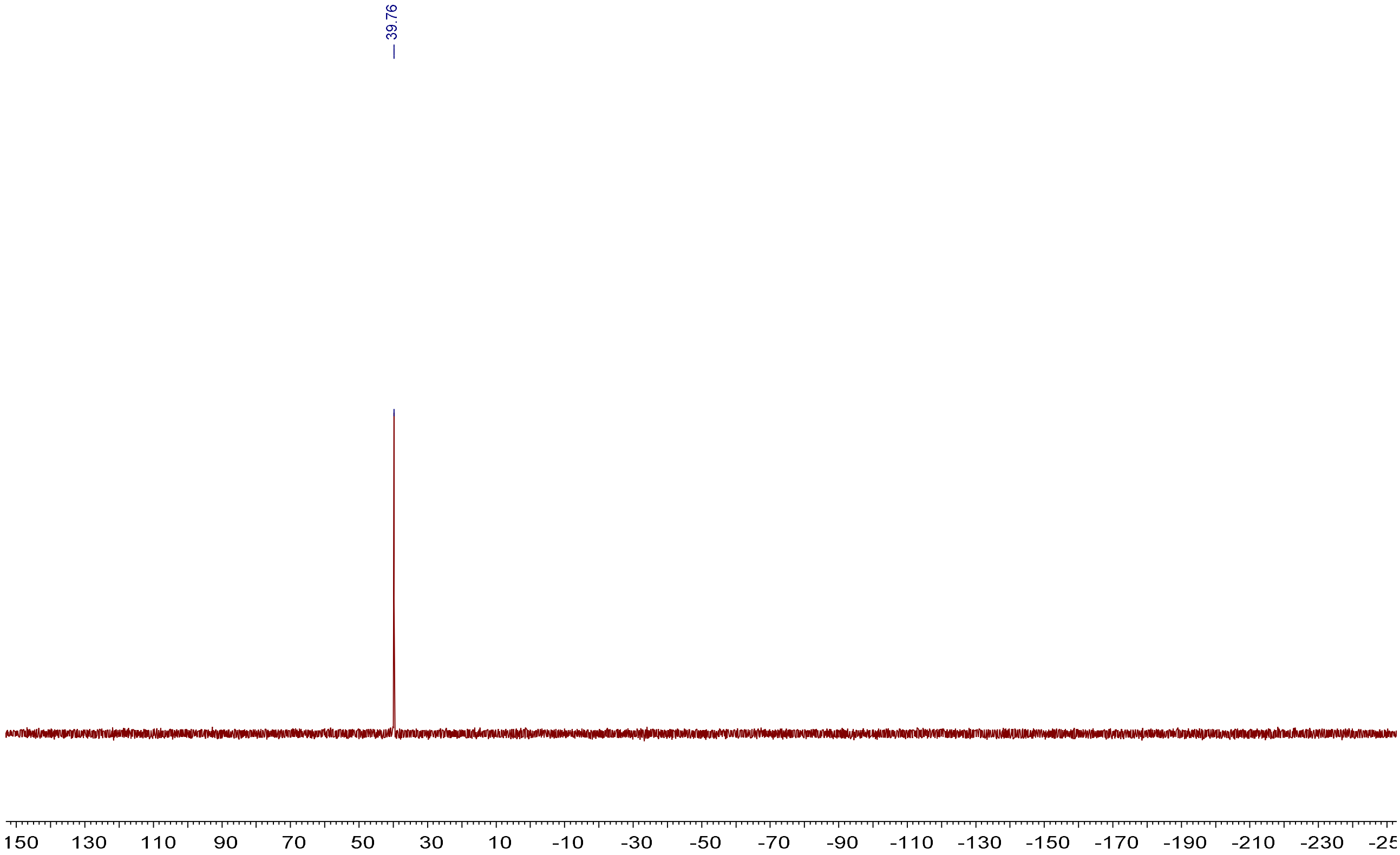
39.76
150
130
110
90
70
50
30
10
-10
-30
-50
-70
-90
-110
-130
-150
-170
-190
-210
-230

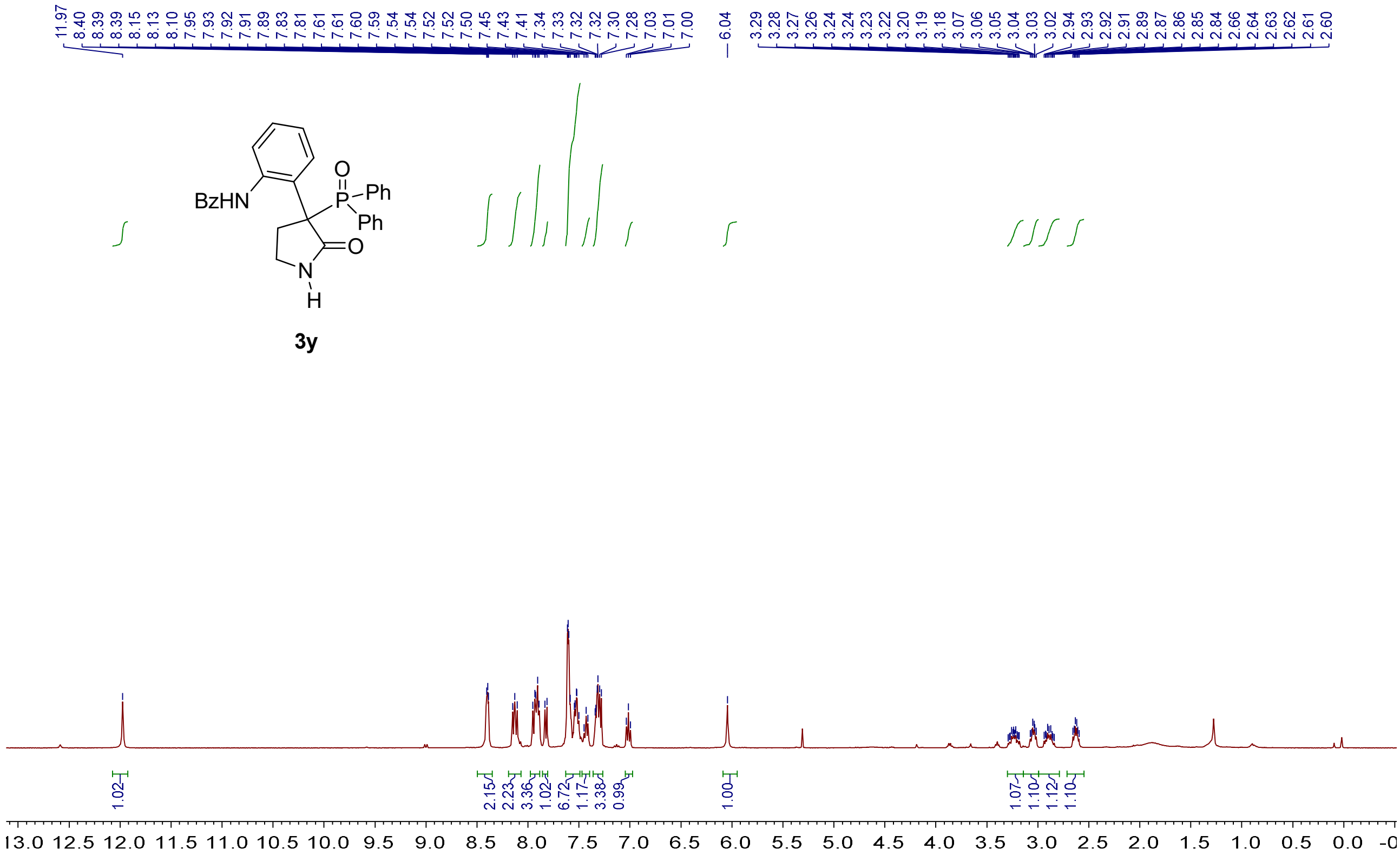

BzHN
O
P
Ph
Ph
O
N
H

3y

11.97 8.40 8.39 8.39 8.15 8.13 8.10 7.95 7.93 7.92 7.91 7.89 7.83 7.81 7.61 7.61 7.60 7.59 7.54 7.54 7.52 7.52 7.50 7.45 7.43 7.41 7.34 7.33 7.32 7.32 7.30 7.28 7.03 7.01 7.00
6.04
3.29 3.28 3.27 3.26 3.24 3.24 3.23 3.22 3.20 3.19 3.18 3.07 3.06 3.05 3.04 3.03 3.02 2.94 2.93 2.92 2.91 2.89 2.87 2.86 2.85 2.84 2.66 2.64 2.63 2.62 2.61 2.60
1.02
2.15 2.23 3.36 1.02 6.72 1.17 3.38 0.99
1.00
1.07 1.10 1.12 1.10
13.0 12.5 12.0 11.5 11.0 10.5 10.0 9.5 9.0 8.5 8.0 7.5 7.0 6.5 6.0 5.5 5.0 4.5 4.0 3.5 3.0 2.5 2.0 1.5 1.0 0.5 0.0

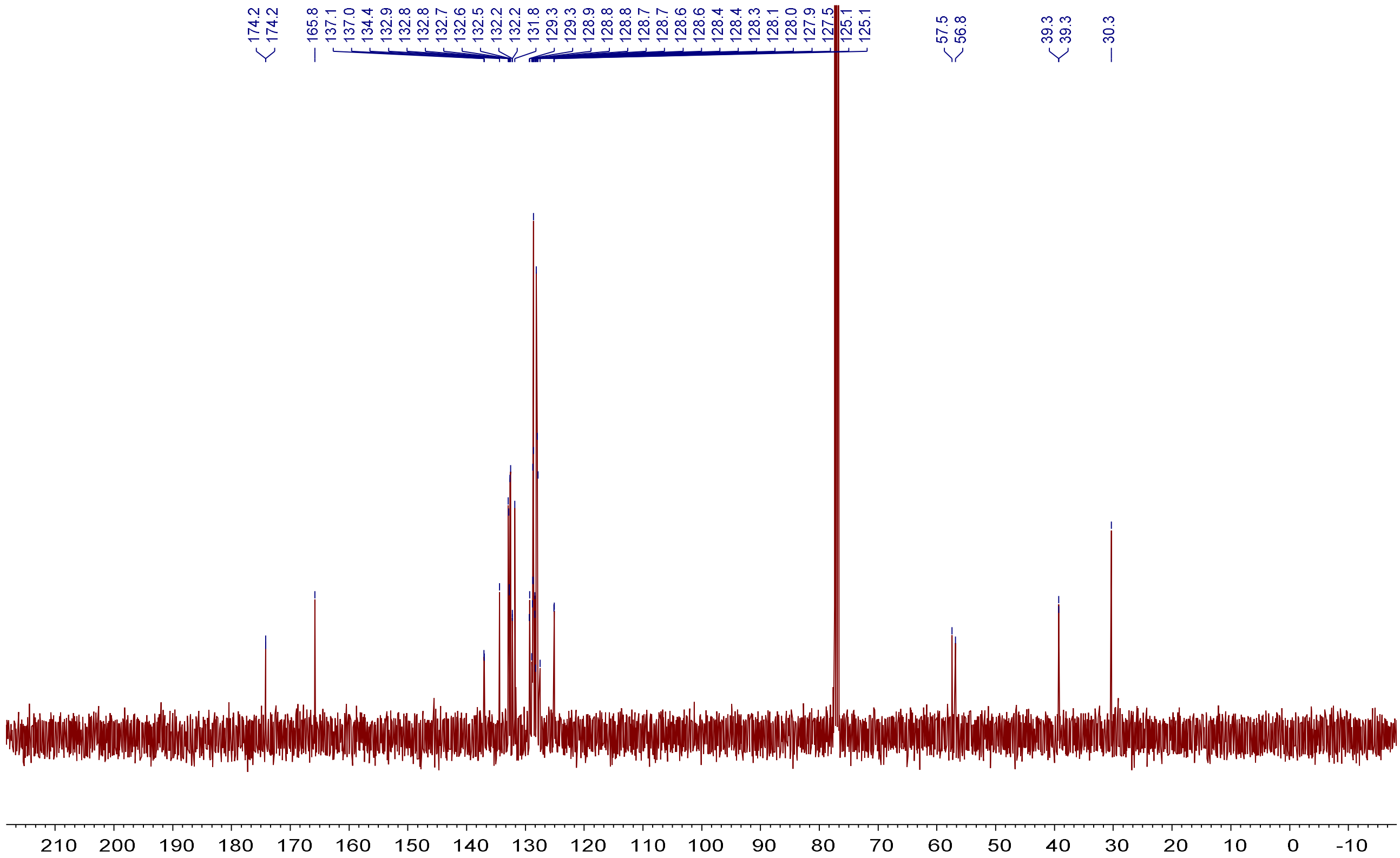

174.2 174.2
165.8
137.1 137.0 134.4 132.9 132.8 132.7 132.6 132.5 132.2 132.2 131.8 129.3 129.3 128.9 128.8 128.8 128.7 128.7 128.6 128.4 128.4 128.3 128.1 128.0 127.9 127.5 125.1 125.1
57.5 56.8
39.3 39.3
30.3
210 200 190 180 170 160 150 140 130 120 110 100 90 80 70 60 50 40 30 20 10 0 -10

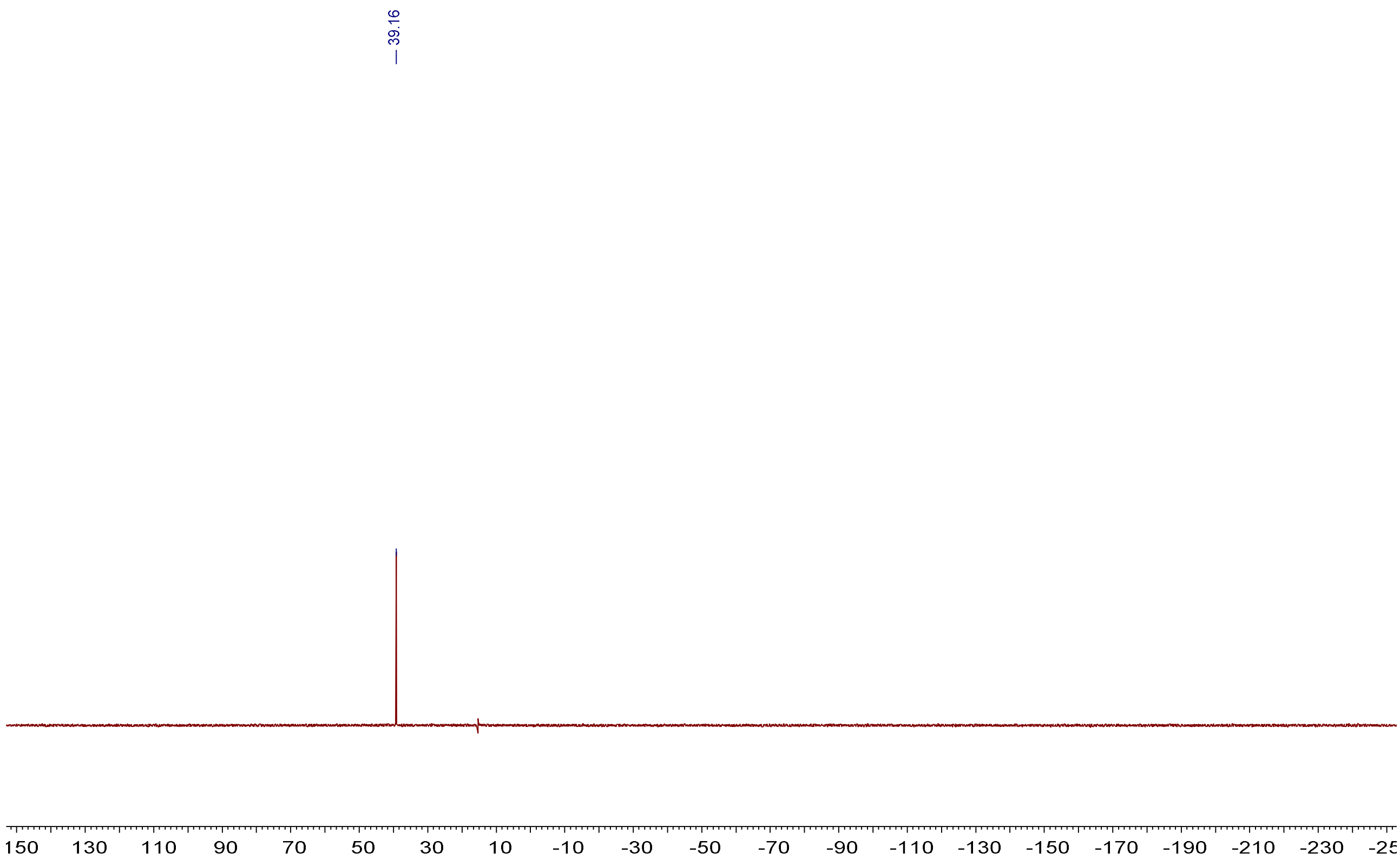
39.16
150 130 110 90 70 50 30 10 -10 -30 -50 -70 -90 -110 -130 -150 -170 -190 -210 -230 -25

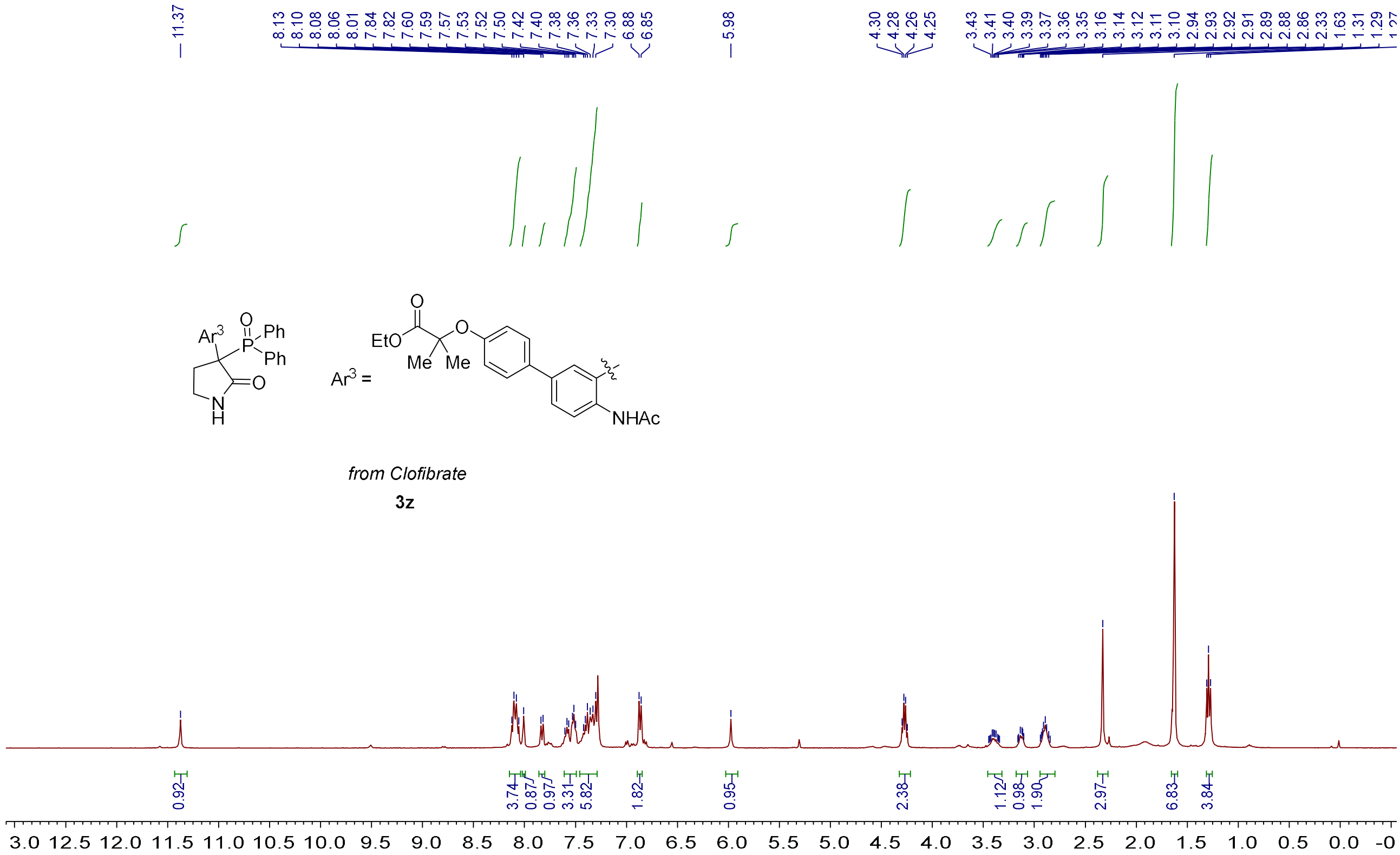

from Clofibrate

3z

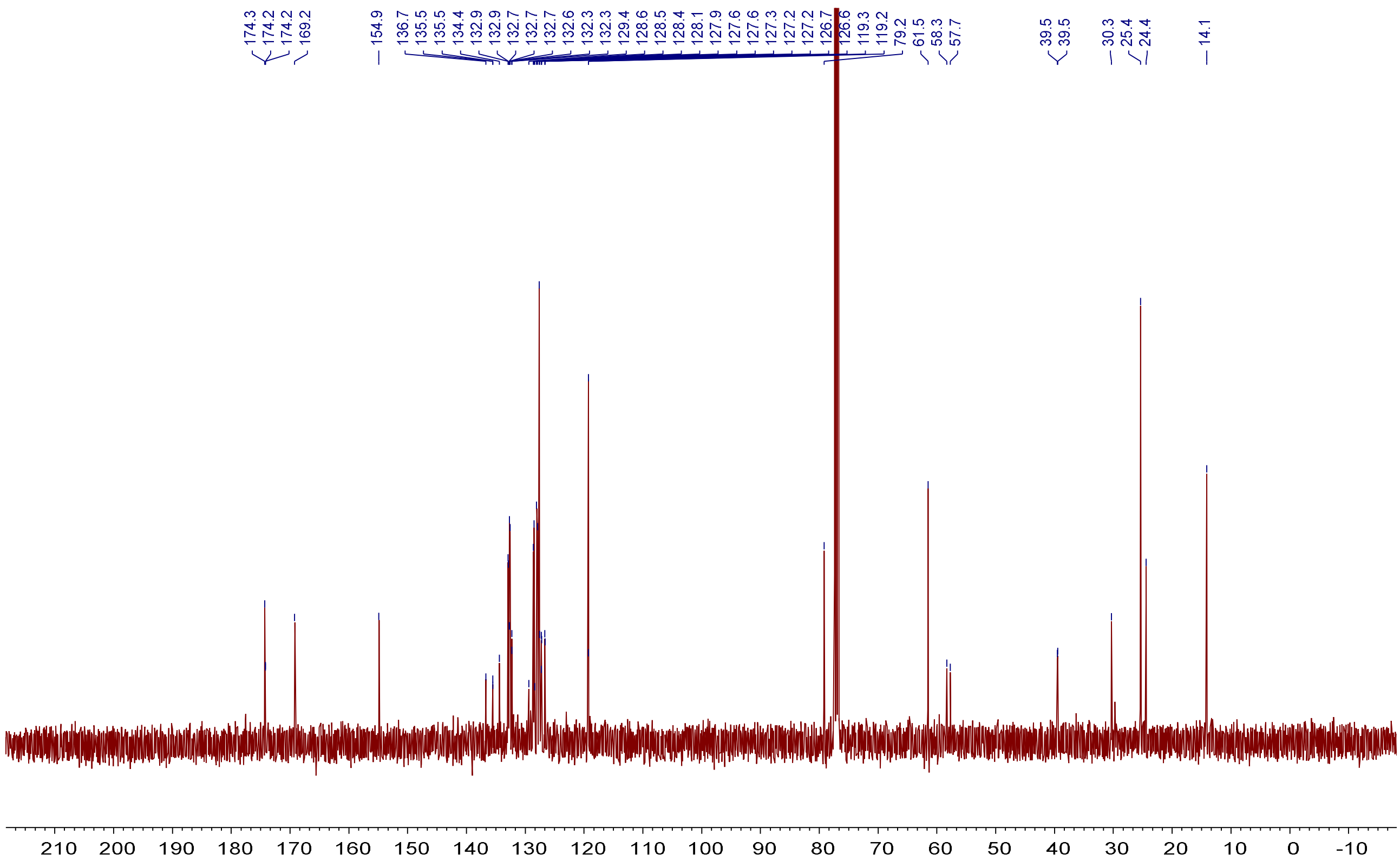

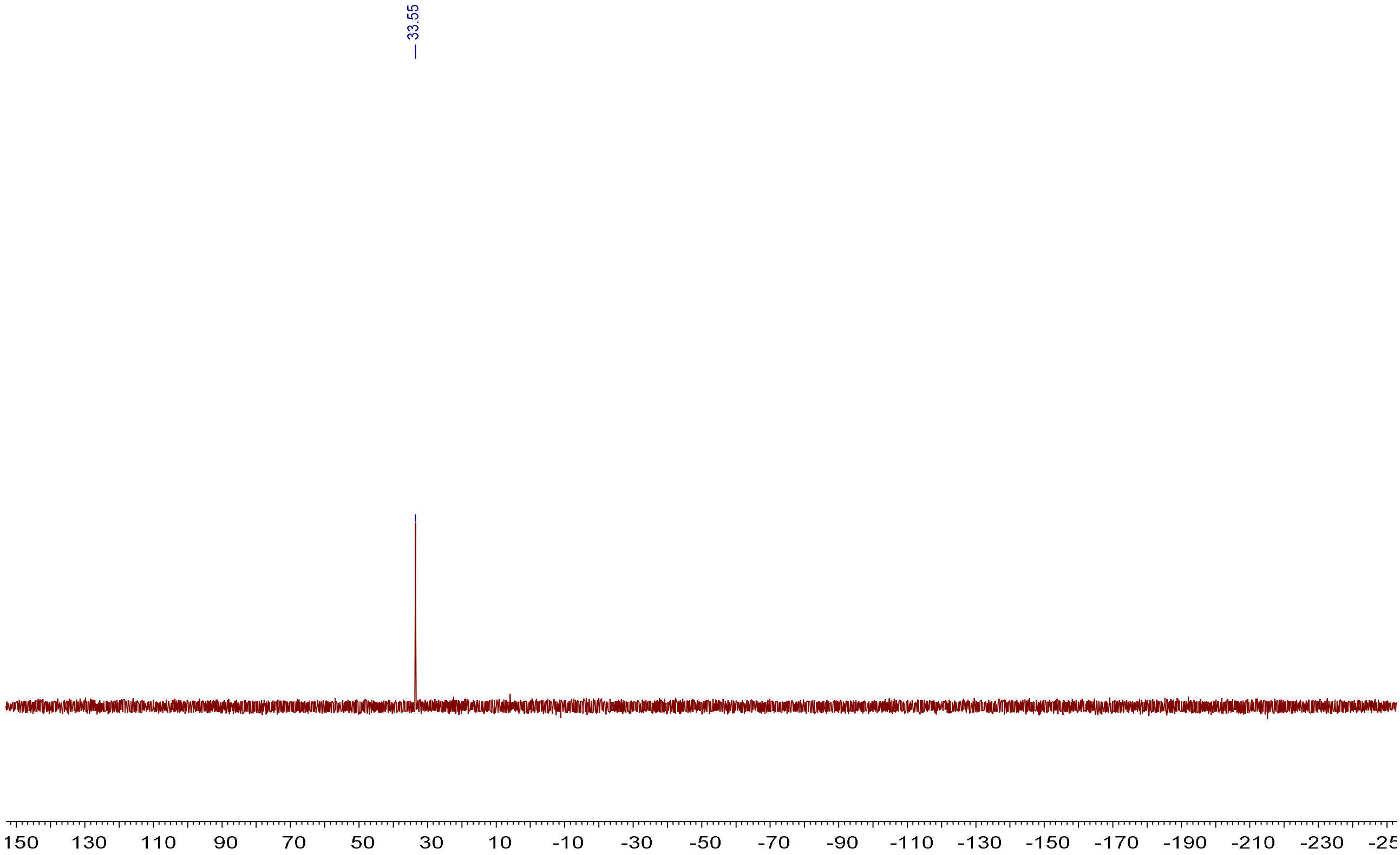
33.55
150
130
110
90
70
50
30
10
-10
-30
-50
-70
-90
-110
-130
-150
-170
-190
-210
-230
-25

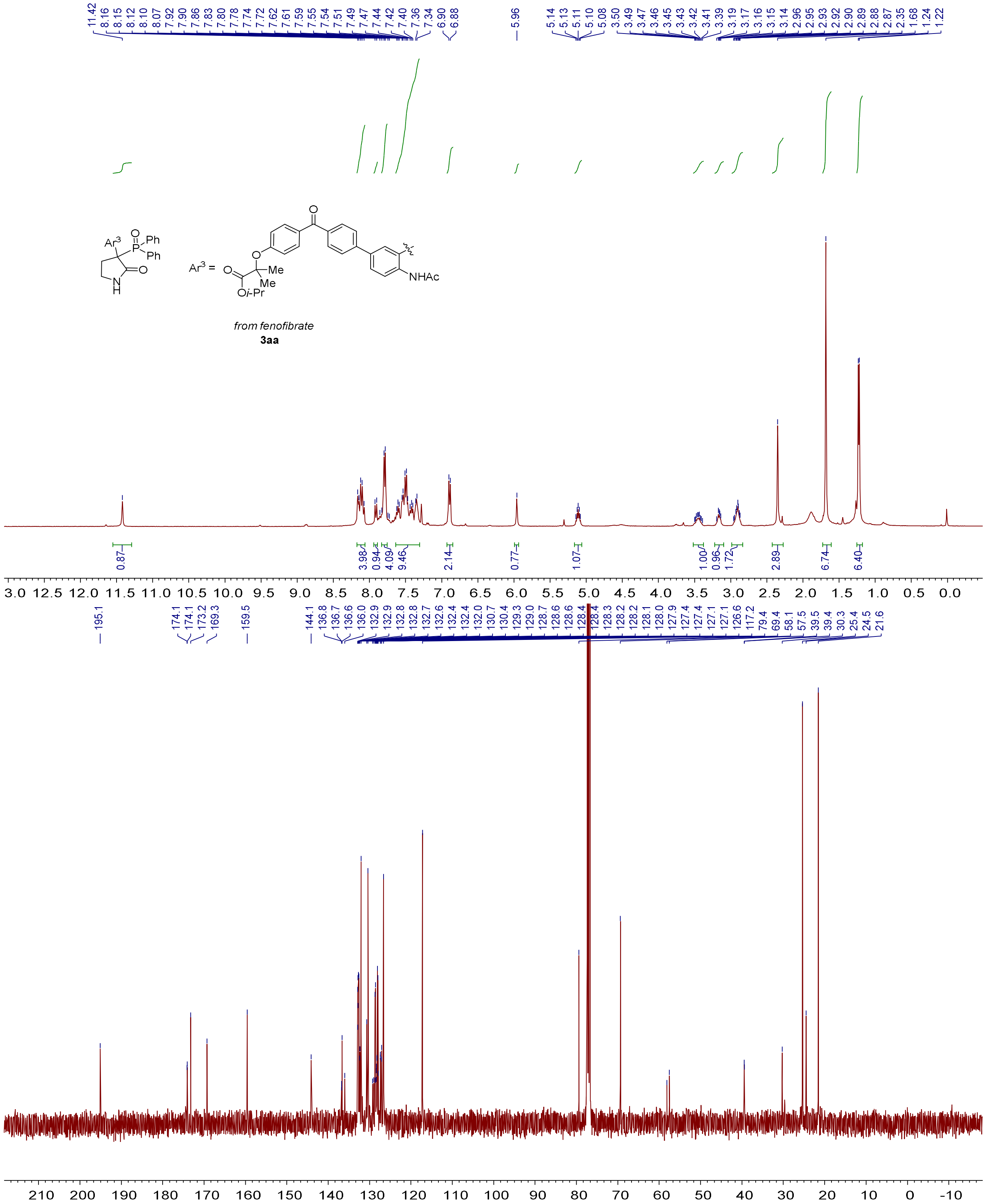
from fenofibrate
3aa
NHAc
Oi-Pr

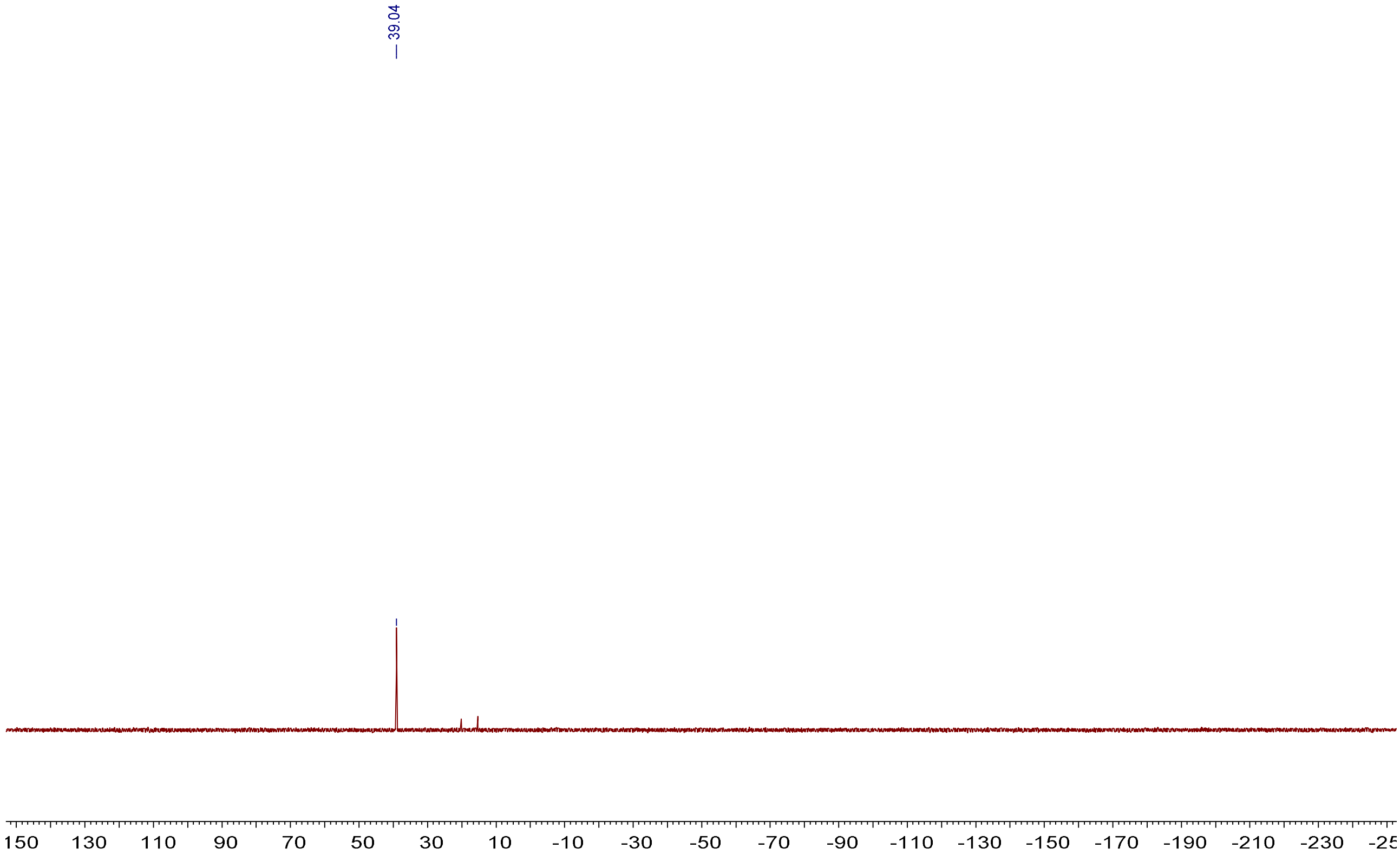
39.04
150 130 110 90 70 50 30 10 -10 -30 -50 -70 -90 -110 -130 -150 -170 -190 -210 -230 -25

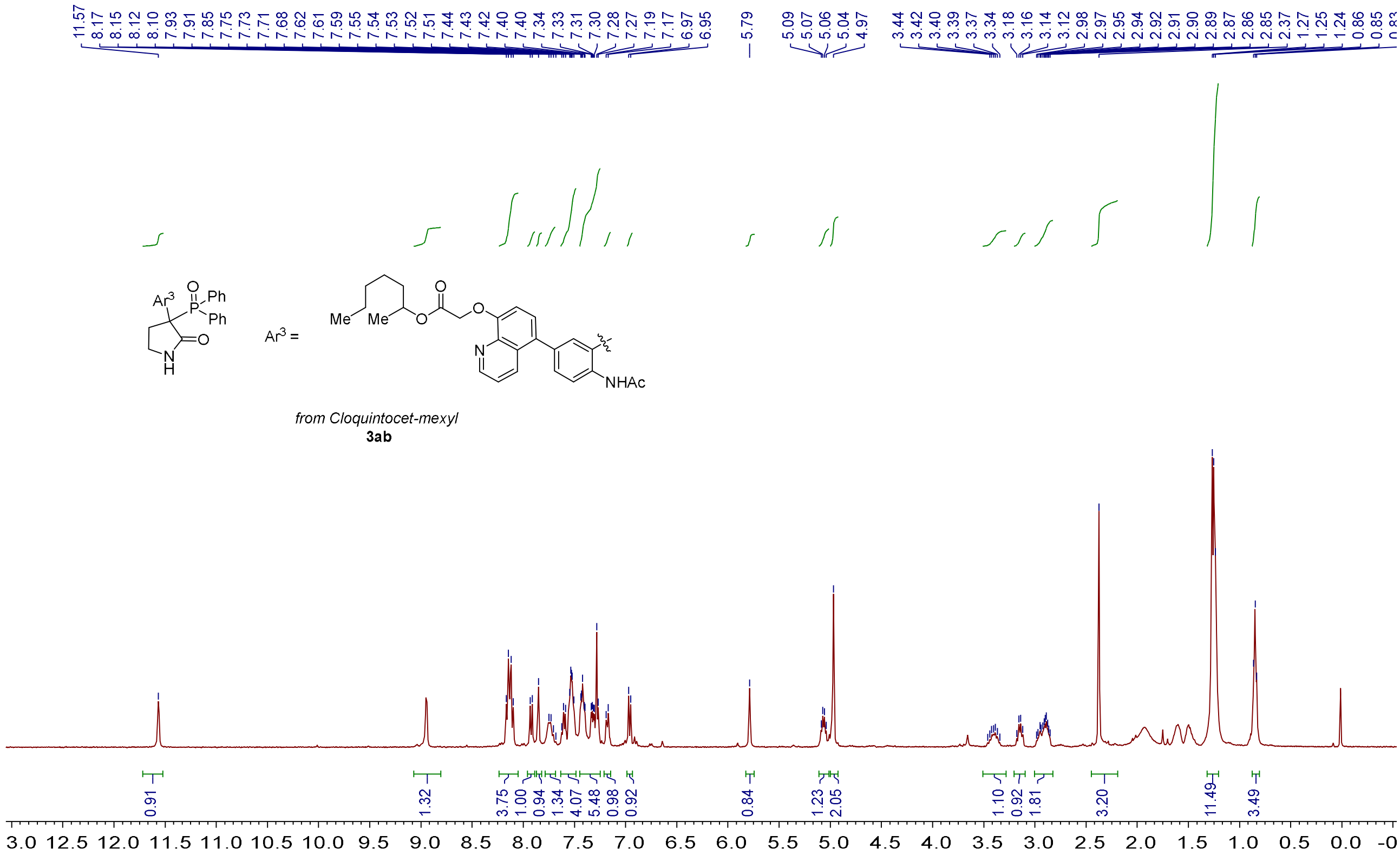
11.57 8.17 8.15 8.12 8.10 7.93 7.91 7.85 7.75 7.73 7.71 7.68 7.62 7.61 7.59 7.55 7.54 7.53 7.52 7.51 7.44 7.43 7.42 7.40 7.40 7.34 7.33 7.31 7.30 7.28 7.27 7.19 7.17 6.97 6.95
5.79
5.09 5.07 5.06 5.04 4.97
3.44 3.42 3.40 3.39 3.37 3.34 3.18 3.16 3.14 3.12 2.98 2.97 2.95 2.94 2.92 2.91 2.90 2.89 2.87 2.86 2.85 2.37 1.27 1.25 1.24 0.86 0.85
Ar3 P Ph Ph O N H O
Ar3 =
Me Me O O O N NHAc
from Cloquintocet-mexyl
3ab
0.91 1.32 3.75 1.00 0.94 1.34 4.07 5.48 0.98 0.92 0.84 1.23 2.05 1.10 0.92 1.81 3.20 11.49 3.49
13.0 12.5 12.0 11.5 11.0 10.5 10.0 9.5 9.0 8.5 8.0 7.5 7.0 6.5 6.0 5.5 5.0 4.5 4.0 3.5 3.0 2.5 2.0 1.5 1.0 0.5 0.0 -0

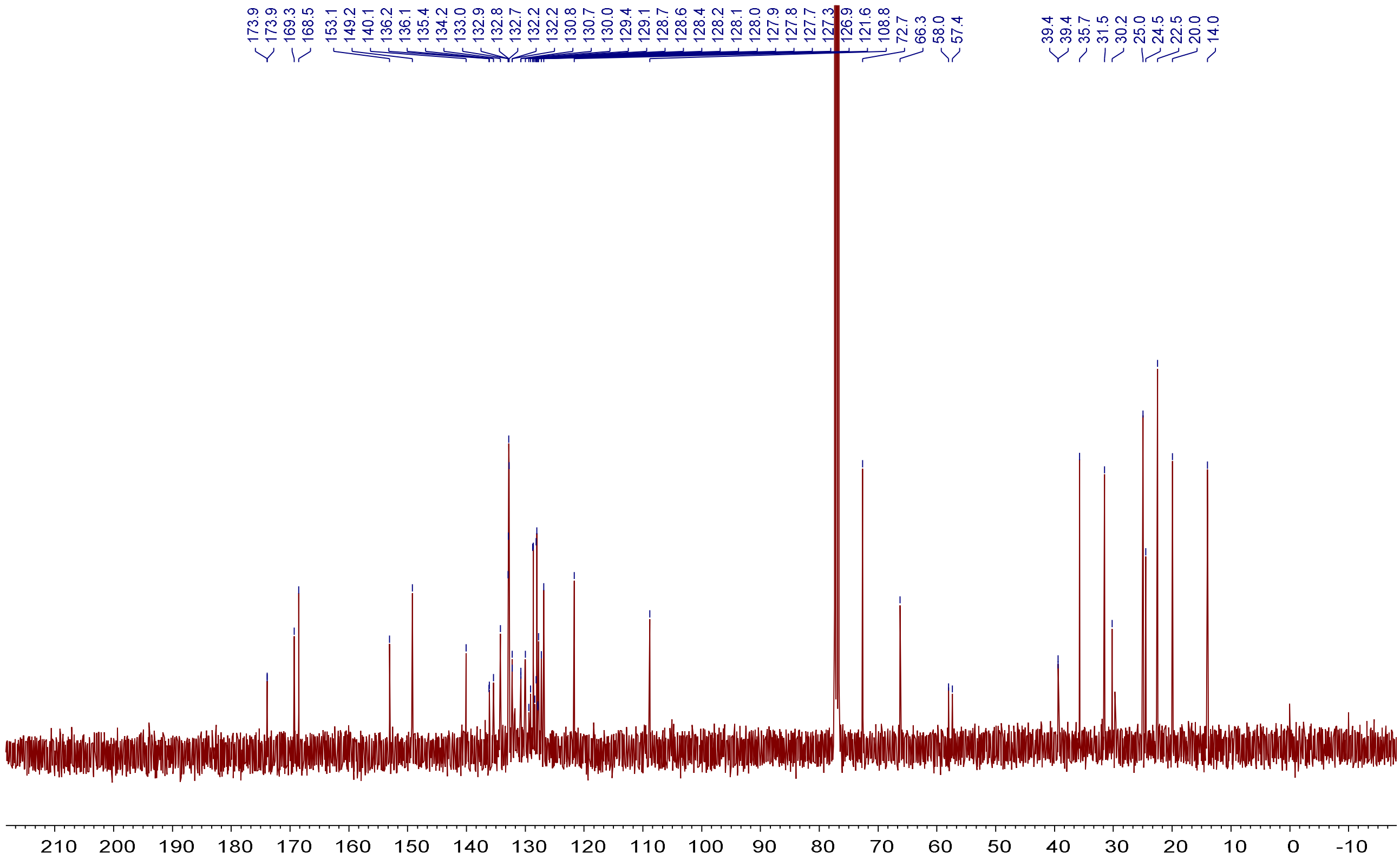
173.9 173.9 169.3 168.5
153.1 149.2 140.1 136.2 136.1 135.4 134.2 133.0 132.9 132.8 132.7 132.2 132.2 130.8 130.7 130.0 129.4 129.1 128.7 128.6 128.4 128.2 128.1 128.0 127.9 127.8 127.7 127.3 126.9 121.6 108.8 72.7 66.3 58.0 57.4
39.4 39.4 35.7 31.5 30.2 25.0 24.5 22.5 20.0 14.0
210 200 190 180 170 160 150 140 130 120 110 100 90 80 70 60 50 40 30 20 10 0 -10

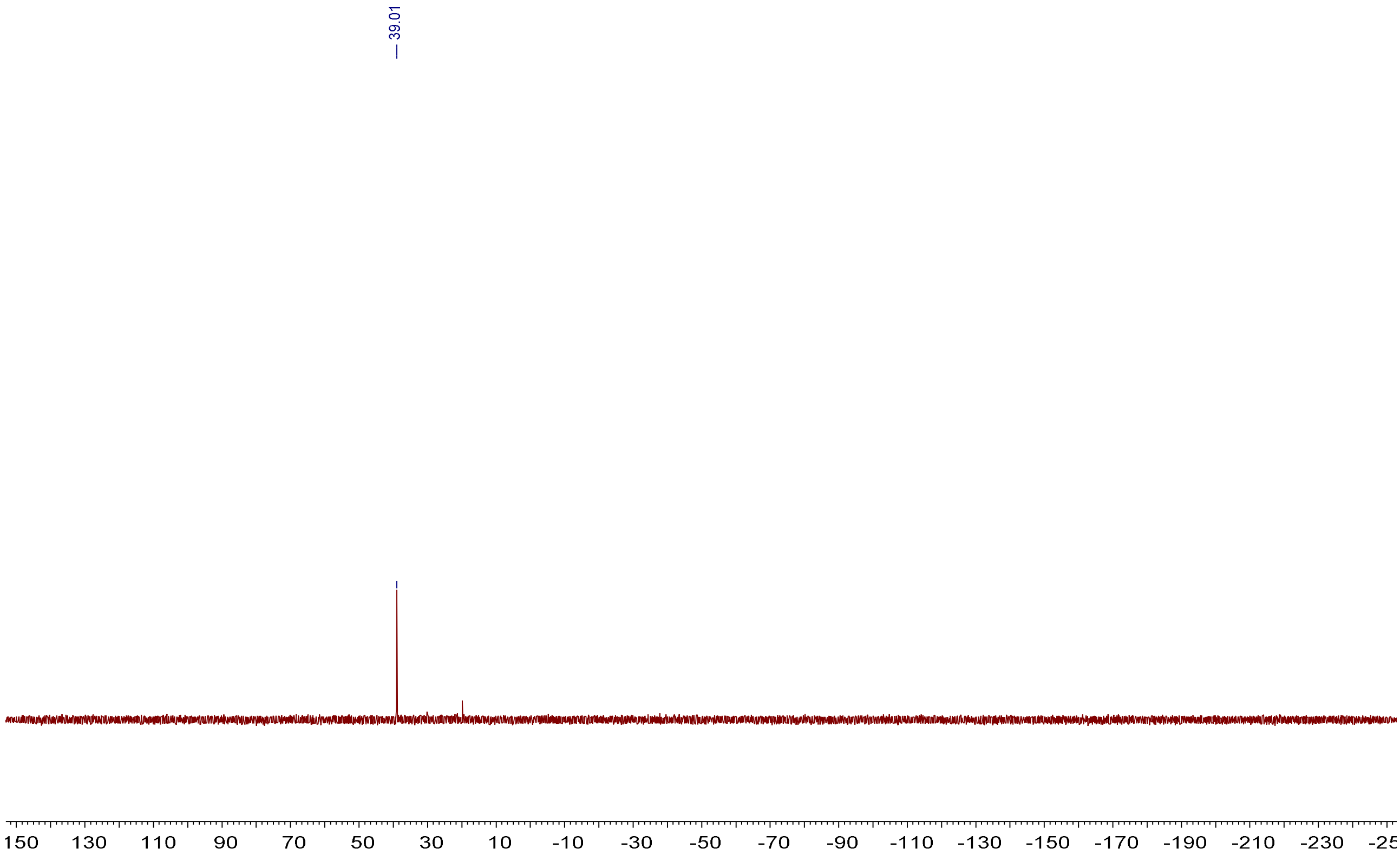
39.01
150
130
110
90
70
50
30
10
-10
-30
-50
-70
-90
-110
-130
-150
-170
-190
-210
-230
-25

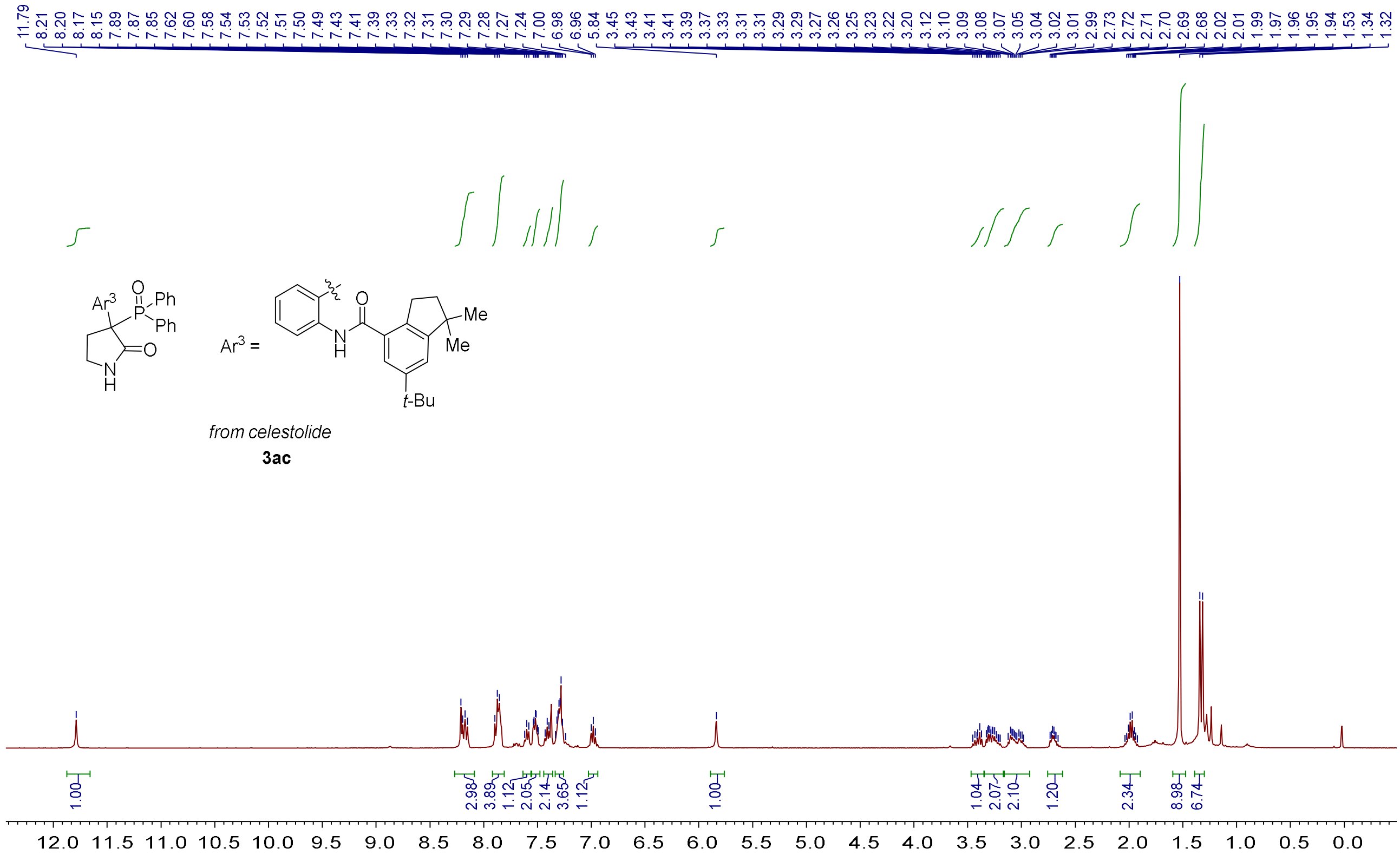

from celestolide

3ac

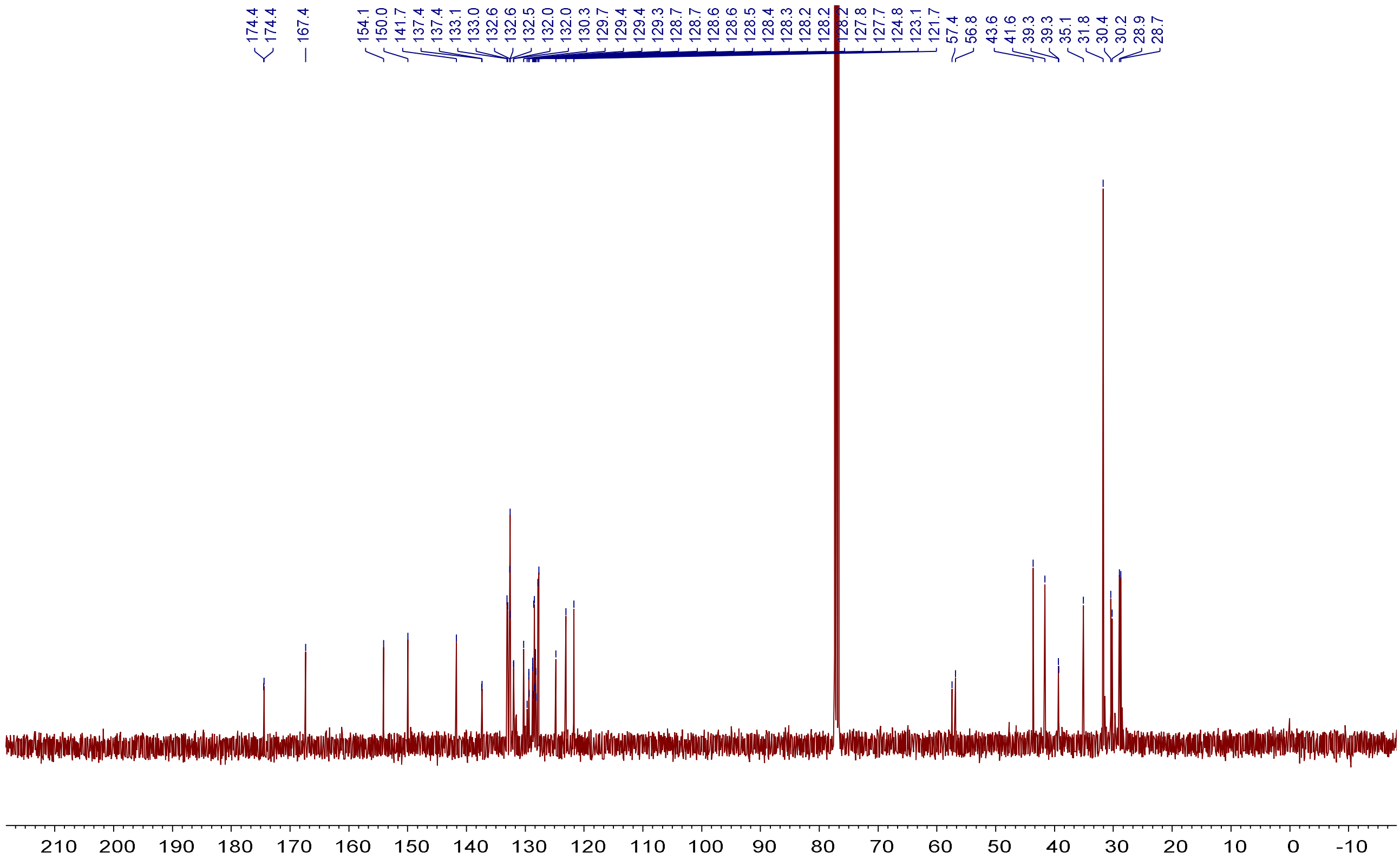

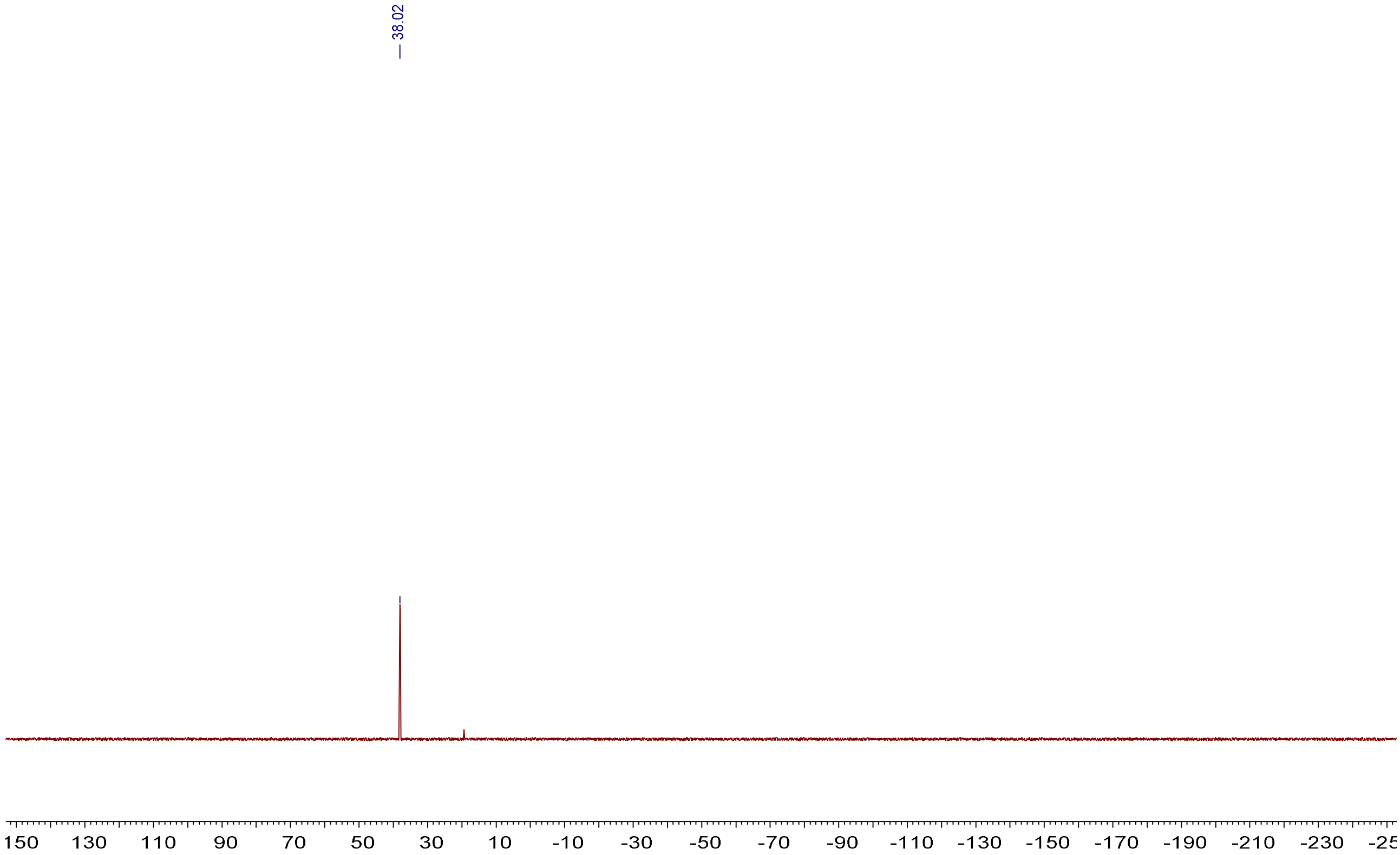
38.02
150
130
110
90
70
50
30
10
-10
-30
-50
-70
-90
-110
-130
-150
-170
-190
-210
-230

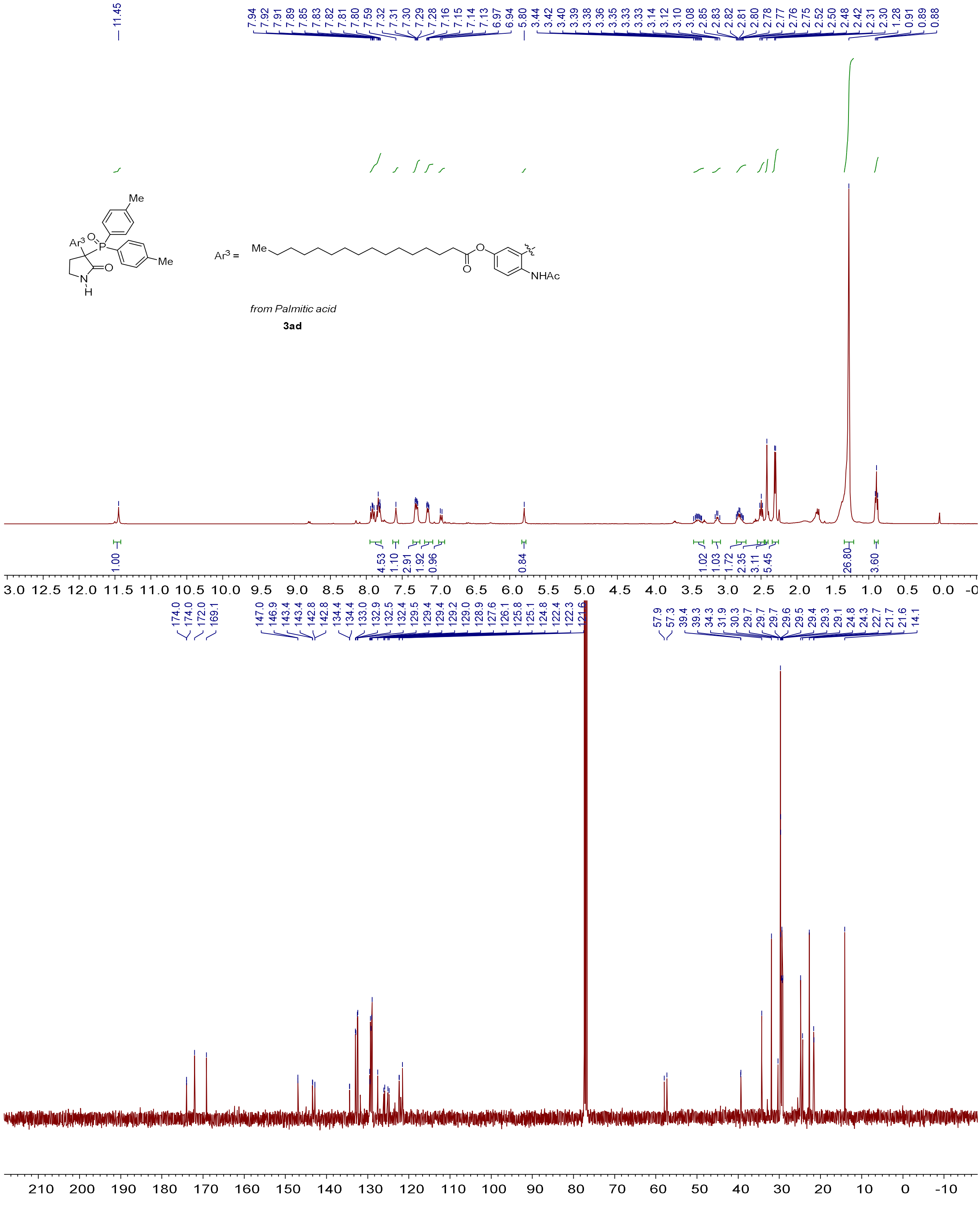

Me
Ar³
O
P
Me
O
N
H
Ar³ =
Me
O
O
NHAc
from Palmitic acid
3ad

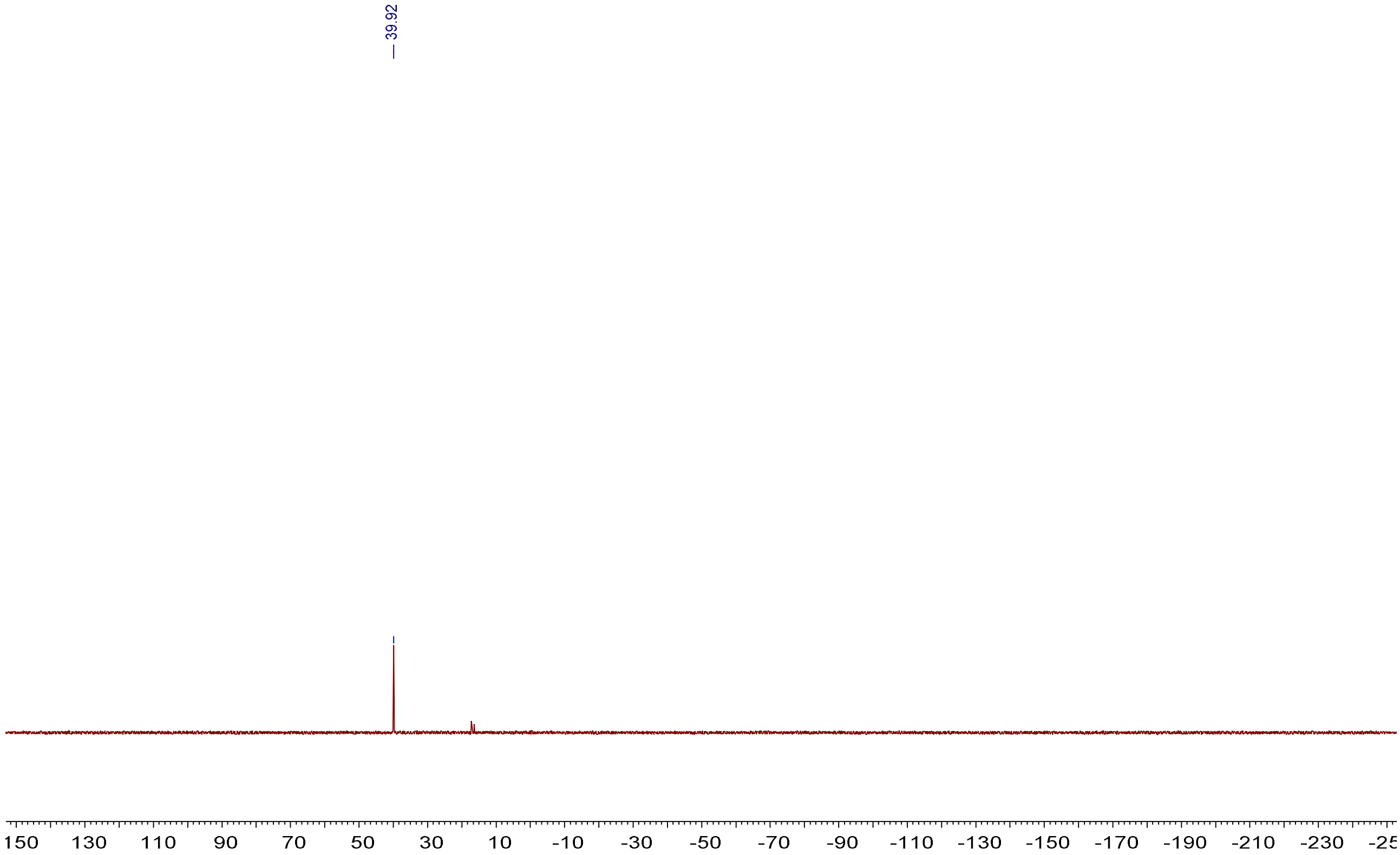

39.92
150
130
110
90
70
50
30
10
-10
-30
-50
-70
-90
-110
-130
-150
-170
-190
-210
-230

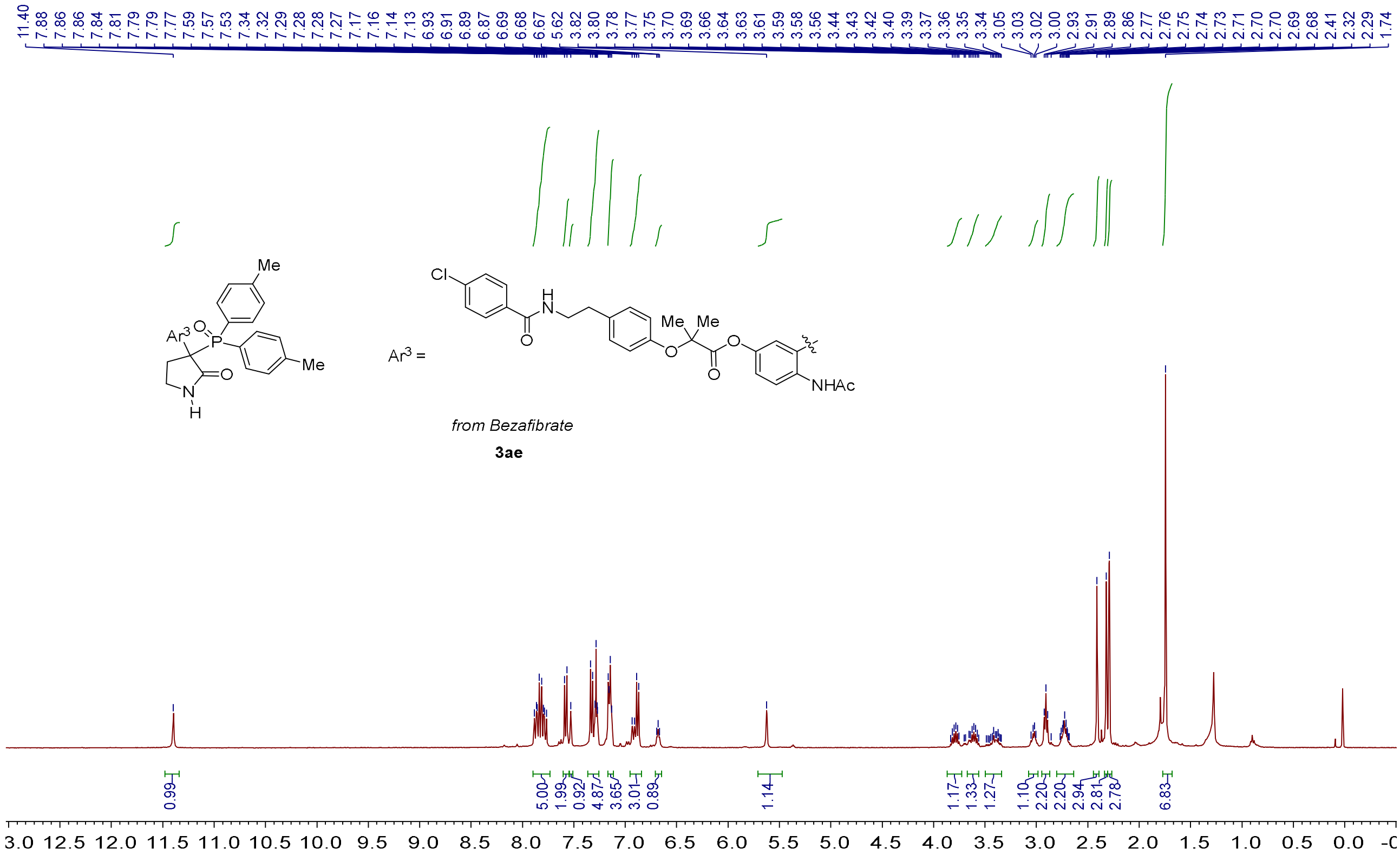

from Bezafibrate

3ae

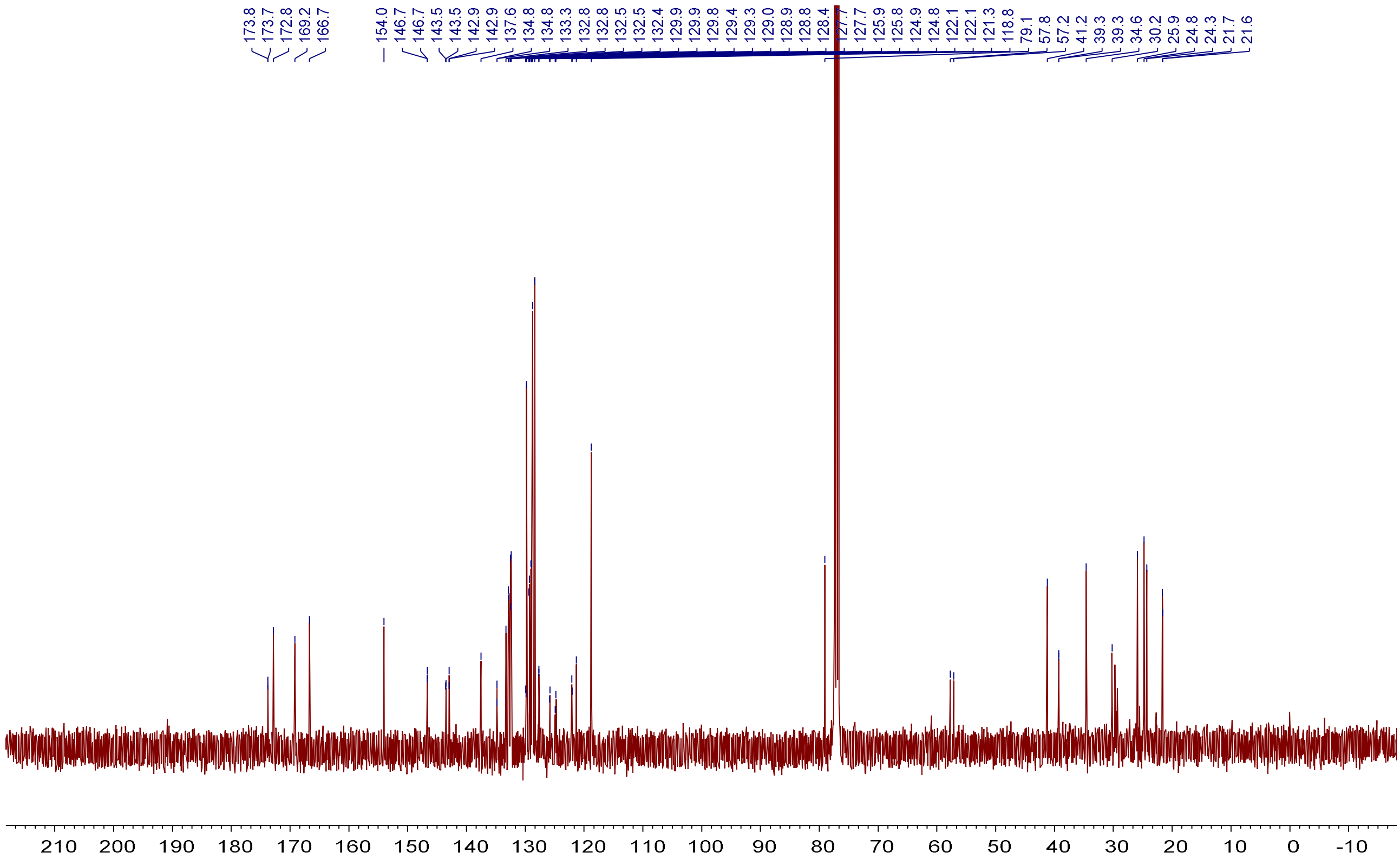

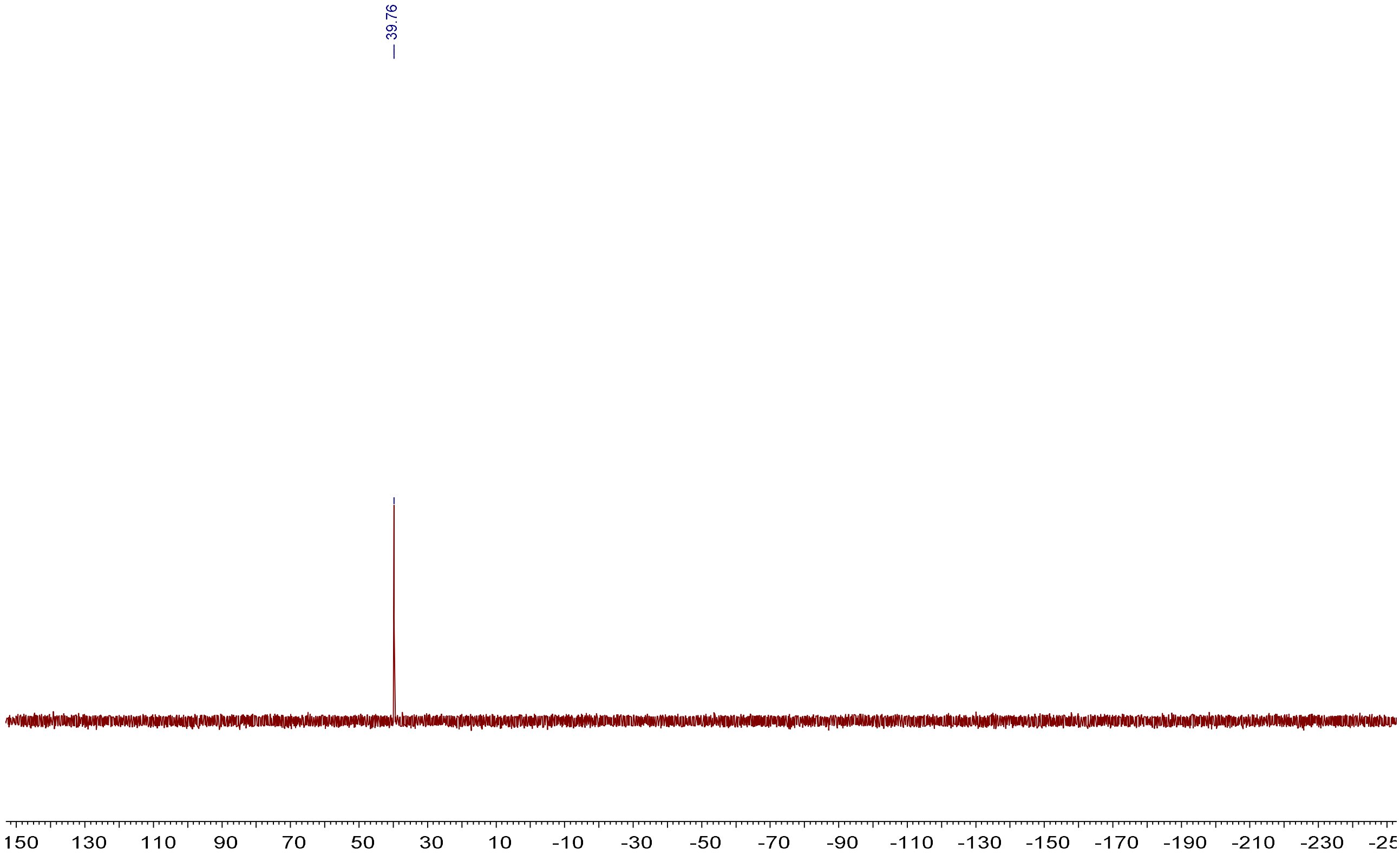
39.76
150
130
110
90
70
50
30
10
-10
-30
-50
-70
-90
-110
-130
-150
-170
-190
-210
-230

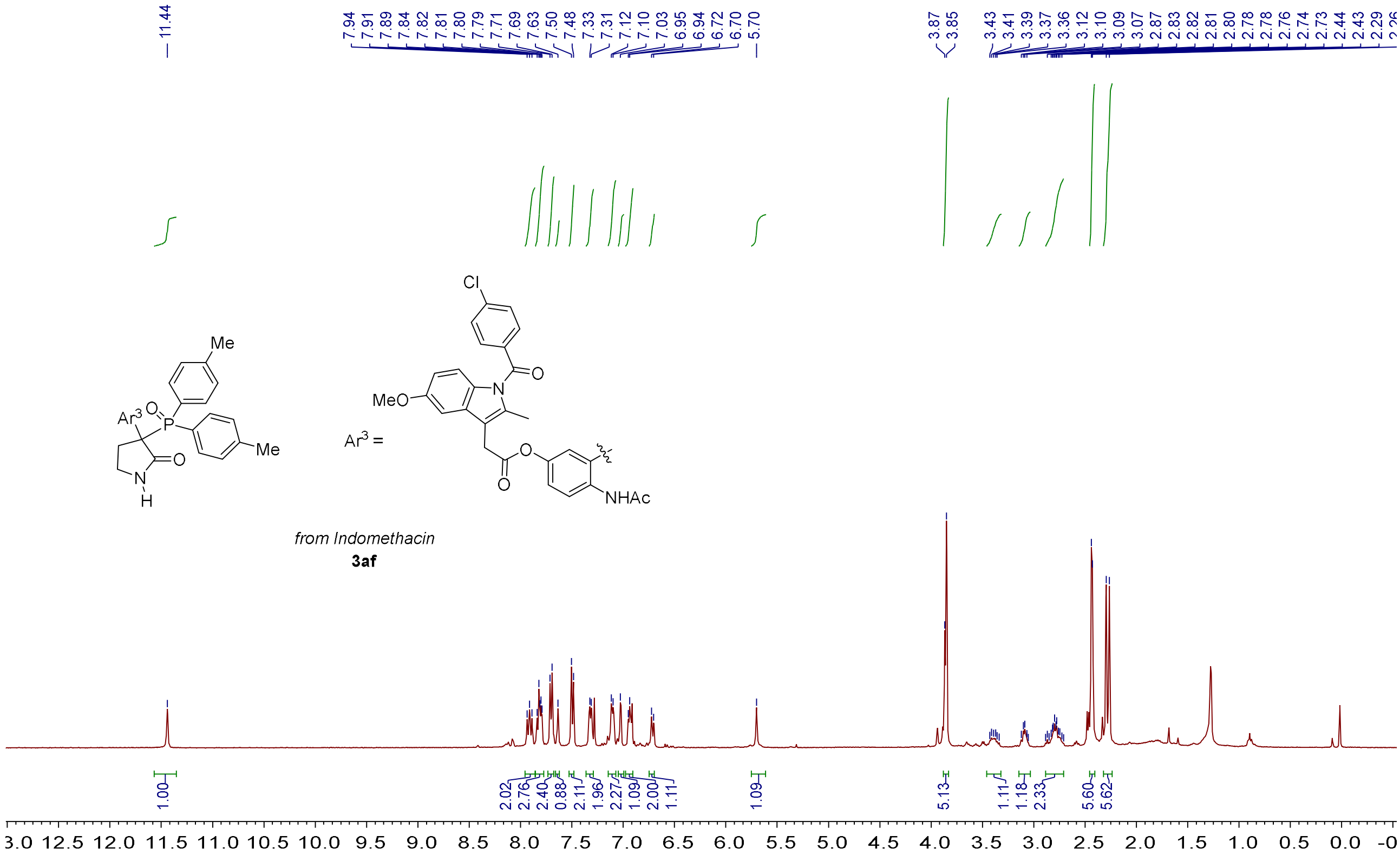

11.44
7.94 7.91 7.89 7.84 7.82 7.81 7.80 7.79 7.71 7.69 7.63 7.50 7.48 7.33 7.31 7.12 7.10 7.03 6.95 6.94 6.72 6.70 5.70
3.87 3.85 3.43 3.41 3.39 3.37 3.36 3.12 3.10 3.09 3.07 2.87 2.83 2.82 2.81 2.80 2.78 2.78 2.76 2.74 2.73 2.44 2.43 2.29
Cl
Me
MeO
N
O
Ar3
P
Me
N
H
Ar3 =
NHAc
from Indomethacin
3af
1.00
2.02 2.76 2.40 0.88 2.11 1.96 2.27 1.09 2.00 1.11
1.09
5.13
1.11 1.18 2.33
5.60 5.62
12.5 12.0 11.5 11.0 10.5 10.0 9.5 9.0 8.5 8.0 7.5 7.0 6.5 6.0 5.5 5.0 4.5 4.0 3.5 3.0 2.5 2.0 1.5 1.0 0.5 0.0

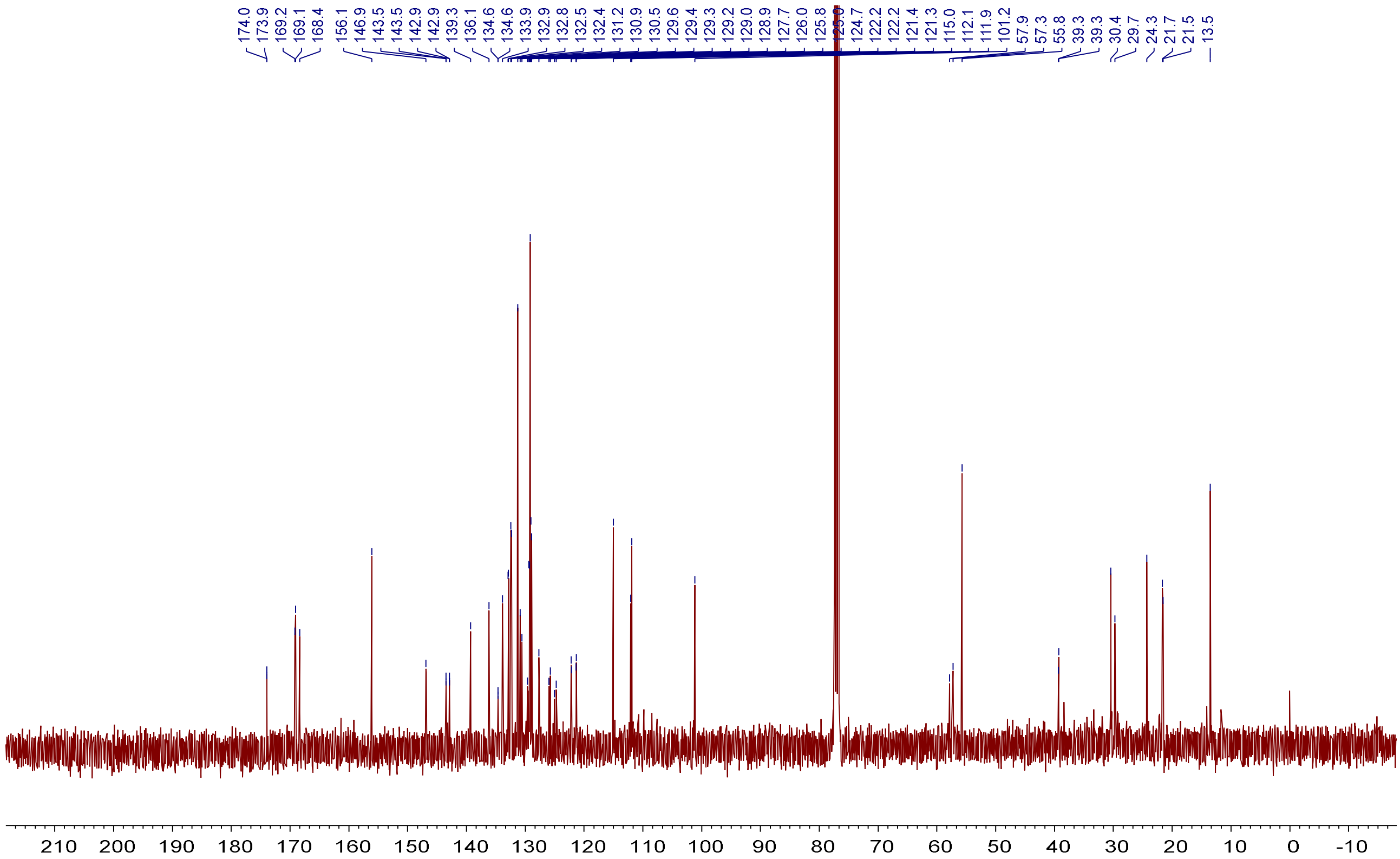

174.0 173.9 169.2 169.1 168.4 156.1 146.9 143.5 143.5 142.9 142.9 139.3 136.1 134.6 134.6 133.9 132.9 132.8 132.5 132.4 131.2 130.9 130.5 129.6 129.4 129.3 129.2 129.0 128.9 127.7 126.0 125.8 124.7 122.2 122.2 121.4 121.3 115.0 112.1 111.9 101.2 57.9 57.3 55.8 39.3 39.3 30.4 29.7 24.3 21.7 21.5 13.5
210 200 190 180 170 160 150 140 130 120 110 100 90 80 70 60 50 40 30 20 10 0 -10

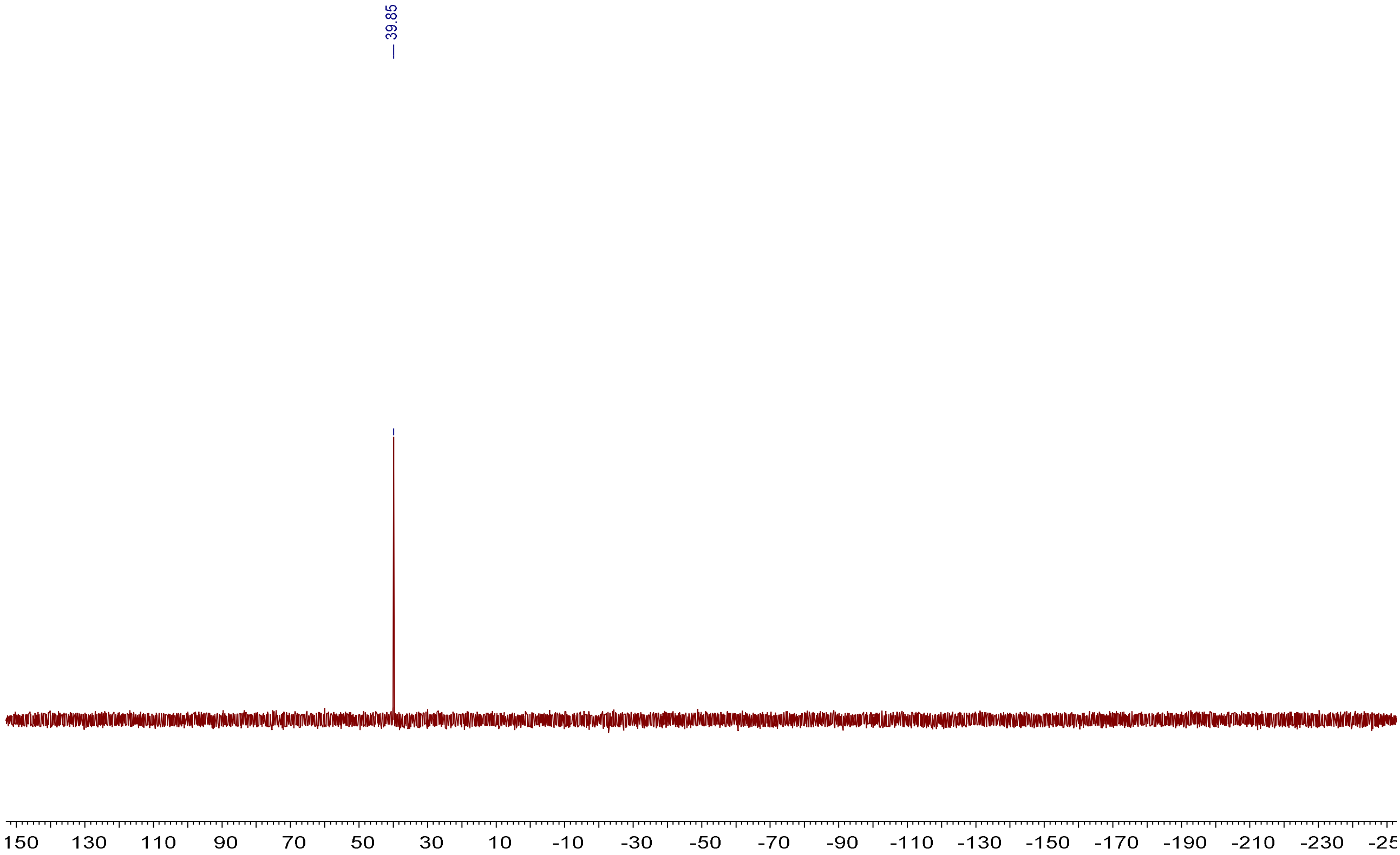
39.85
150
130
110
90
70
50
30
10
-10
-30
-50
-70
-90
-110
-130
-150
-170
-190
-210
-230

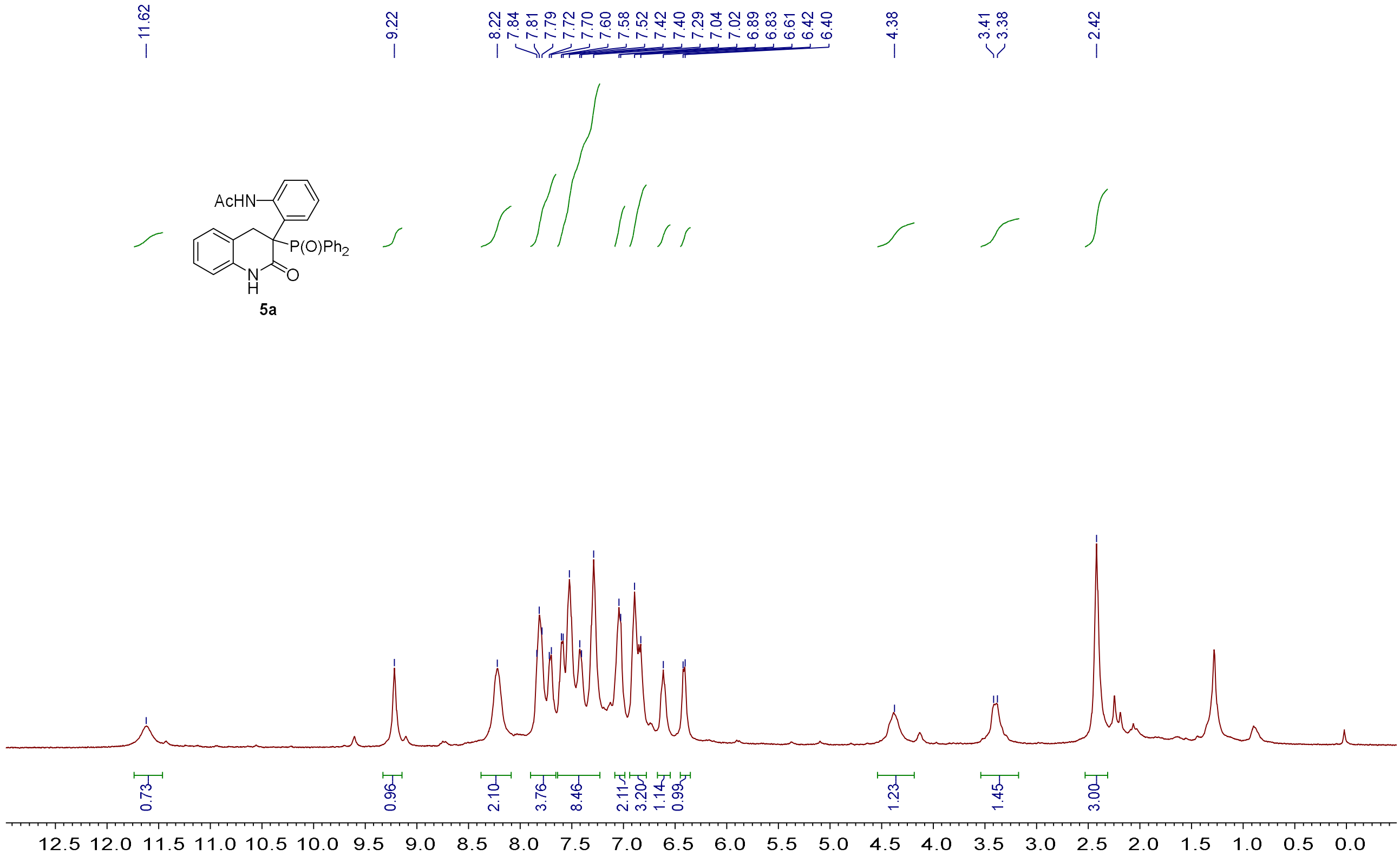
AcHN
P(O)Ph2
N
H
O
5a
11.62
9.22
8.22
7.84
7.81
7.79
7.72
7.70
7.60
7.58
7.52
7.42
7.40
7.29
7.04
7.02
6.89
6.83
6.61
6.42
6.40
4.38
3.41
3.38
2.42
0.73
0.96
2.10
3.76
8.46
2.11
3.20
1.14
0.99
1.23
1.45
3.00
12.5 12.0 11.5 11.0 10.5 10.0 9.5 9.0 8.5 8.0 7.5 7.0 6.5 6.0 5.5 5.0 4.5 4.0 3.5 3.0 2.5 2.0 1.5 1.0 0.5 0.0

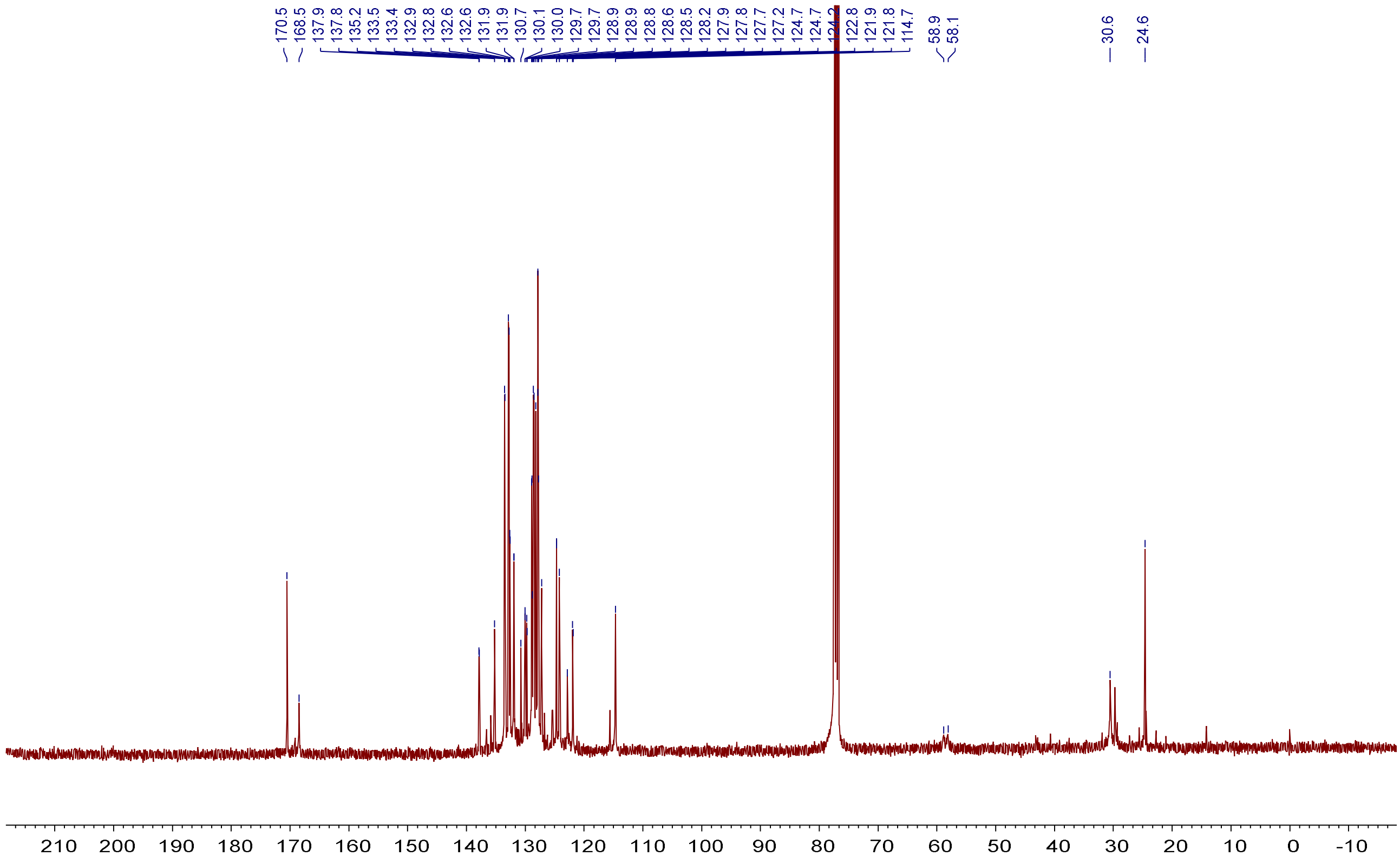
170.5
168.5
137.9
137.8
135.2
133.5
133.4
132.9
132.8
132.6
132.6
131.9
131.9
130.7
130.1
130.0
129.7
129.7
128.9
128.9
128.8
128.6
128.5
128.2
127.9
127.8
127.7
127.2
124.7
124.7
124.2
122.8
121.9
121.8
114.7
58.9
58.1
30.6
24.6
210 200 190 180 170 160 150 140 130 120 110 100 90 80 70 60 50 40 30 20 10 0 -10

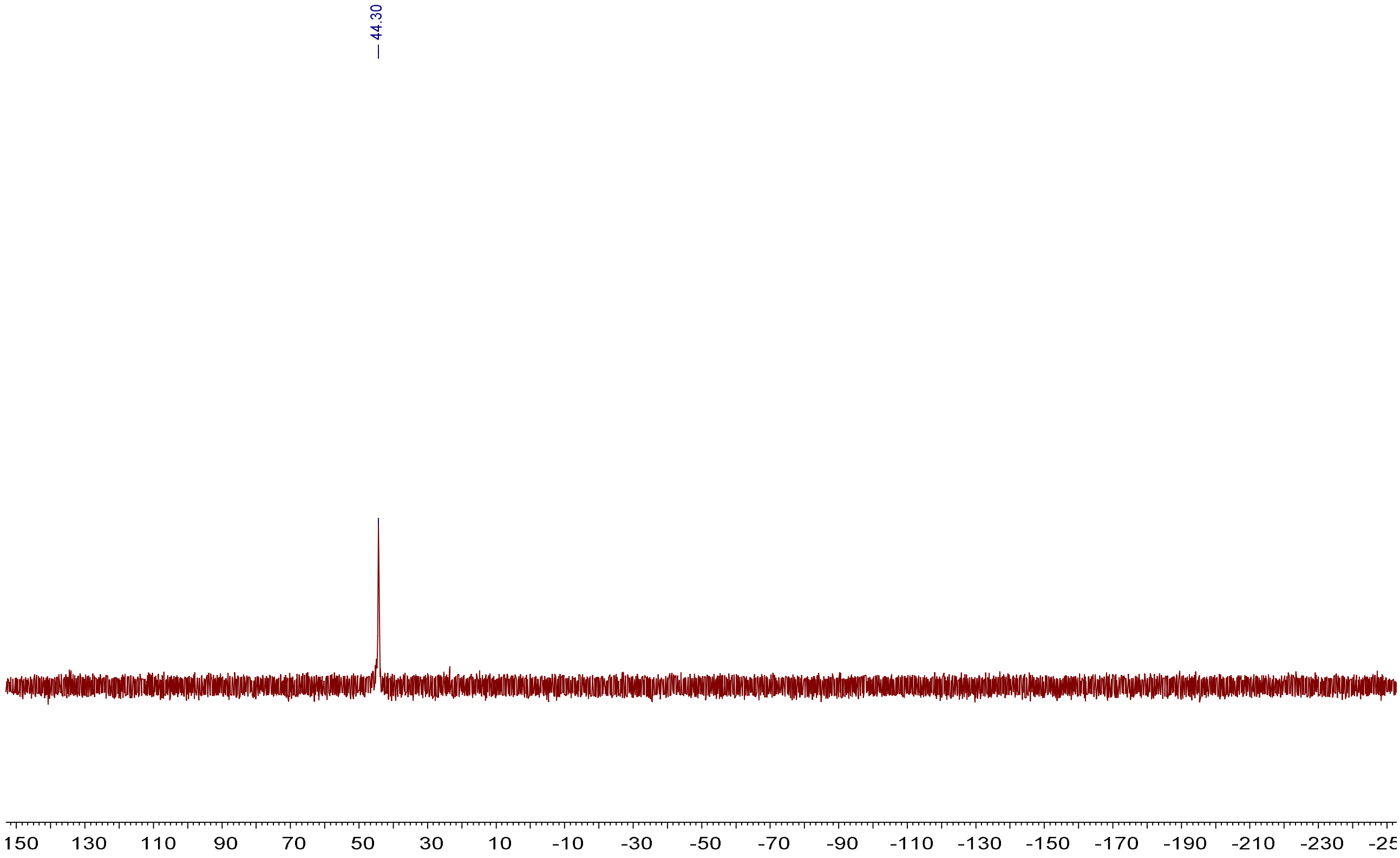
44.30
150 130 110 90 70 50 30 10 -10 -30 -50 -70 -90 -110 -130 -150 -170 -190 -210 -230 -25

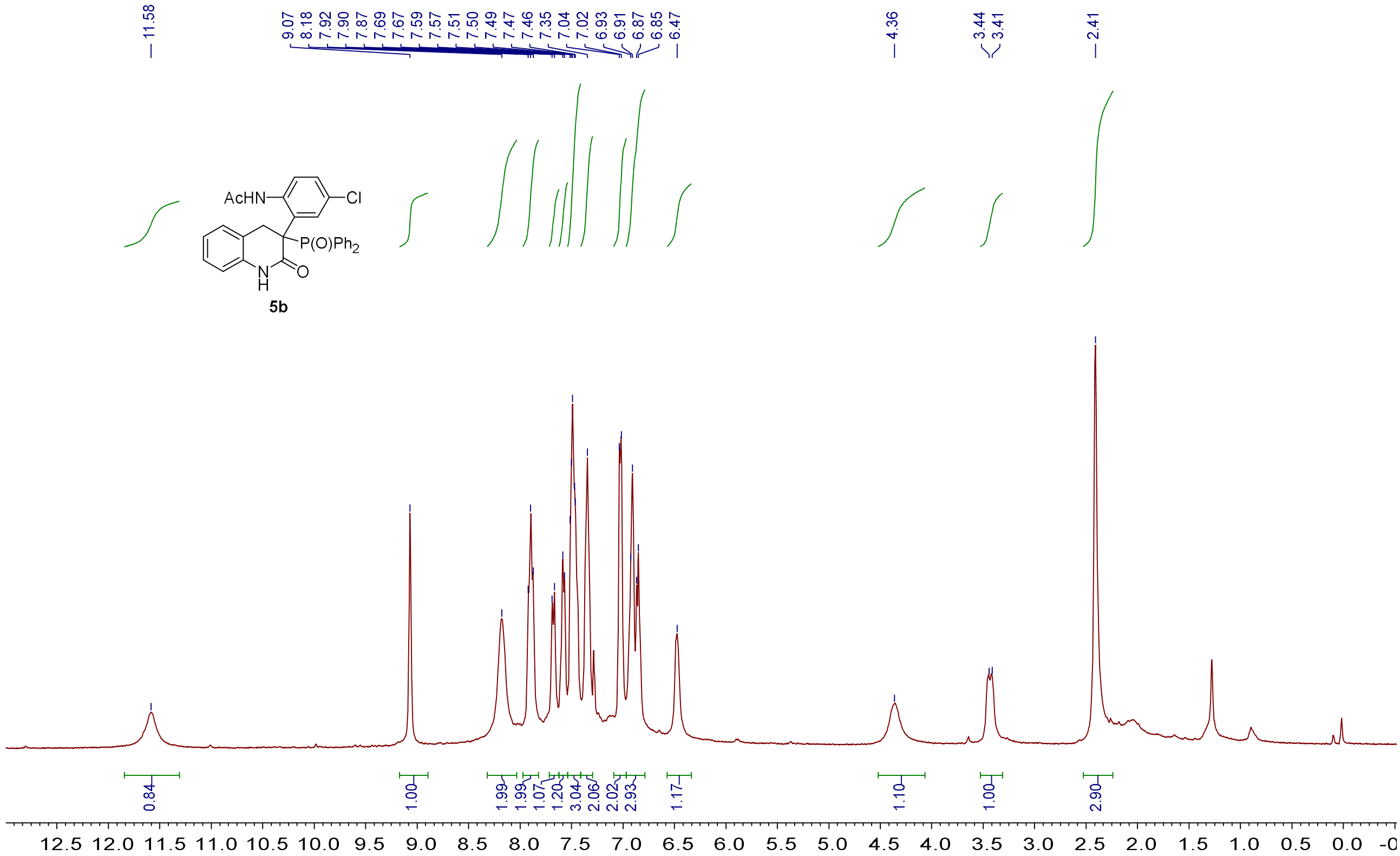
11.58
9.07 8.18 7.92 7.90 7.87 7.69 7.67 7.59 7.57 7.51 7.50 7.49 7.47 7.46 7.35 7.04 7.02 6.93 6.91 6.87 6.85 6.47
4.36
3.44 3.41
2.41
AcHN
Cl
P(O)Ph2
N
H
O
5b
0.84 1.00 1.99 1.99 1.07 1.20 3.04 2.06 2.02 2.93 1.17 1.10 1.00 2.90
12.5 12.0 11.5 11.0 10.5 10.0 9.5 9.0 8.5 8.0 7.5 7.0 6.5 6.0 5.5 5.0 4.5 4.0 3.5 3.0 2.5 2.0 1.5 1.0 0.5 0.0
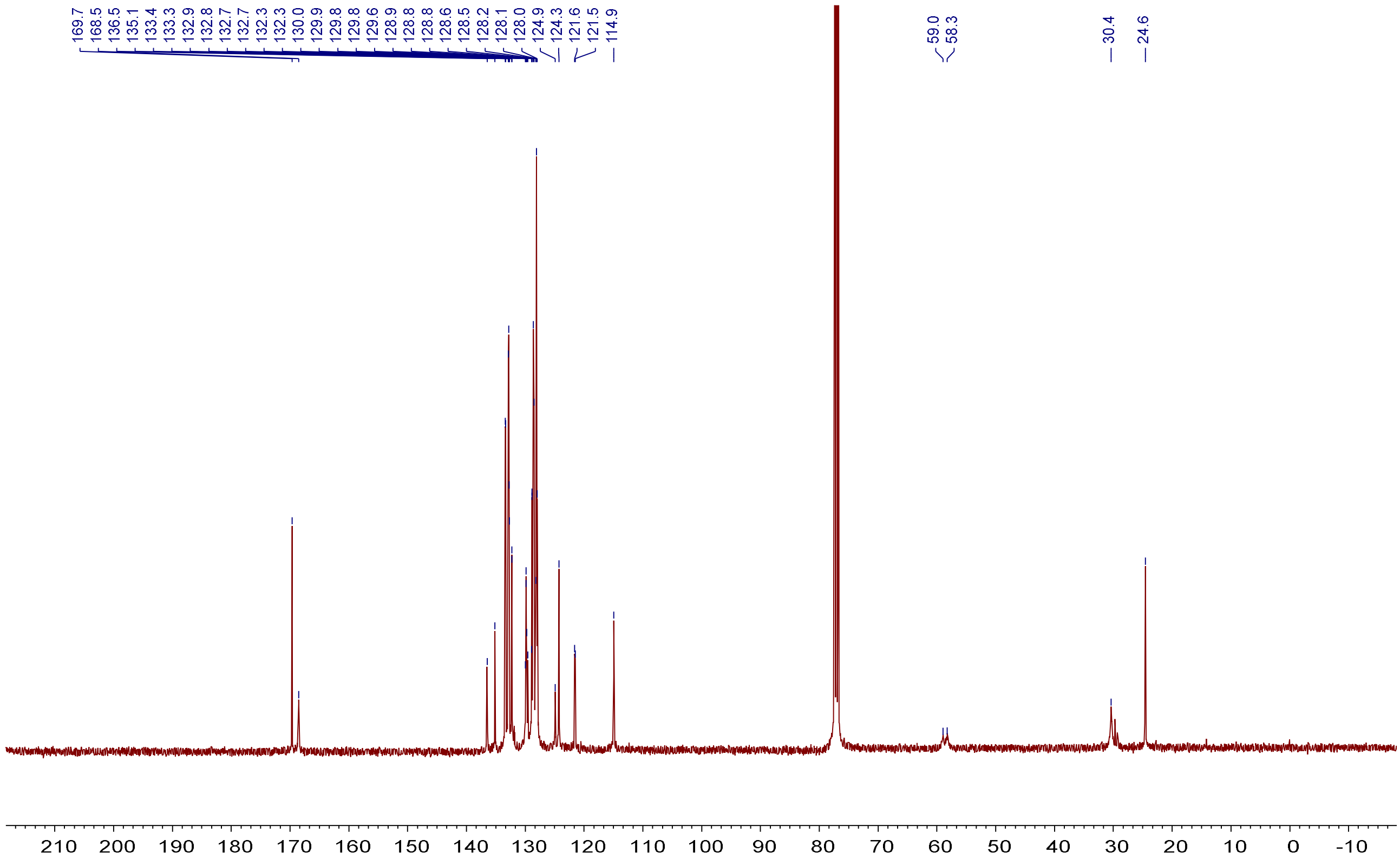
169.7 168.5 136.5 135.1 133.4 133.3 132.9 132.8 132.7 132.7 132.3 132.3 130.0 129.9 129.8 129.8 129.6 128.9 128.8 128.8 128.6 128.5 128.2 128.1 128.0 124.9 124.3 121.6 121.5 114.9
59.0 58.3
30.4
24.6
210 200 190 180 170 160 150 140 130 120 110 100 90 80 70 60 50 40 30 20 10 0 -10

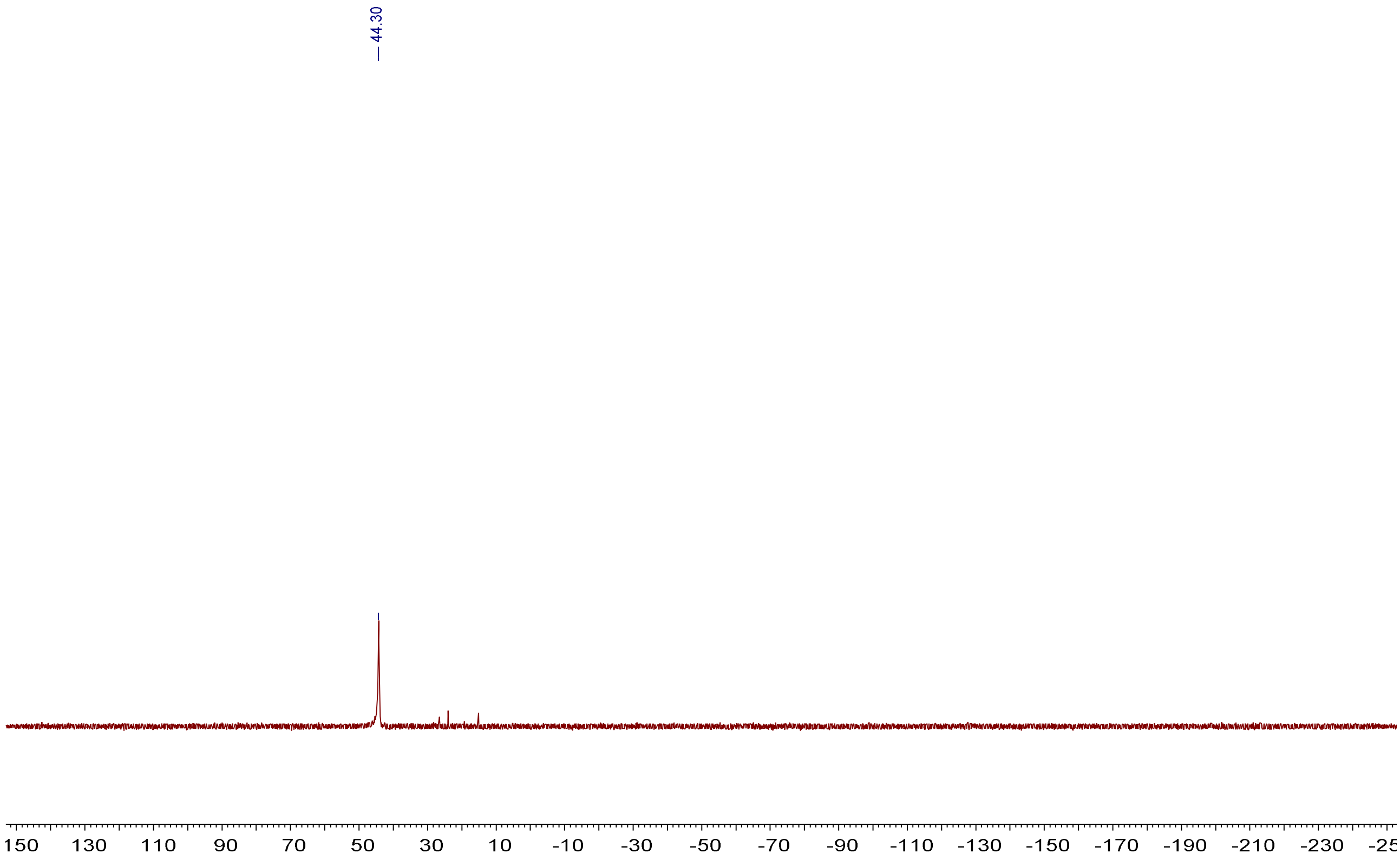
44.30
150 130 110 90 70 50 30 10 -10 -30 -50 -70 -90 -110 -130 -150 -170 -190 -210 -230

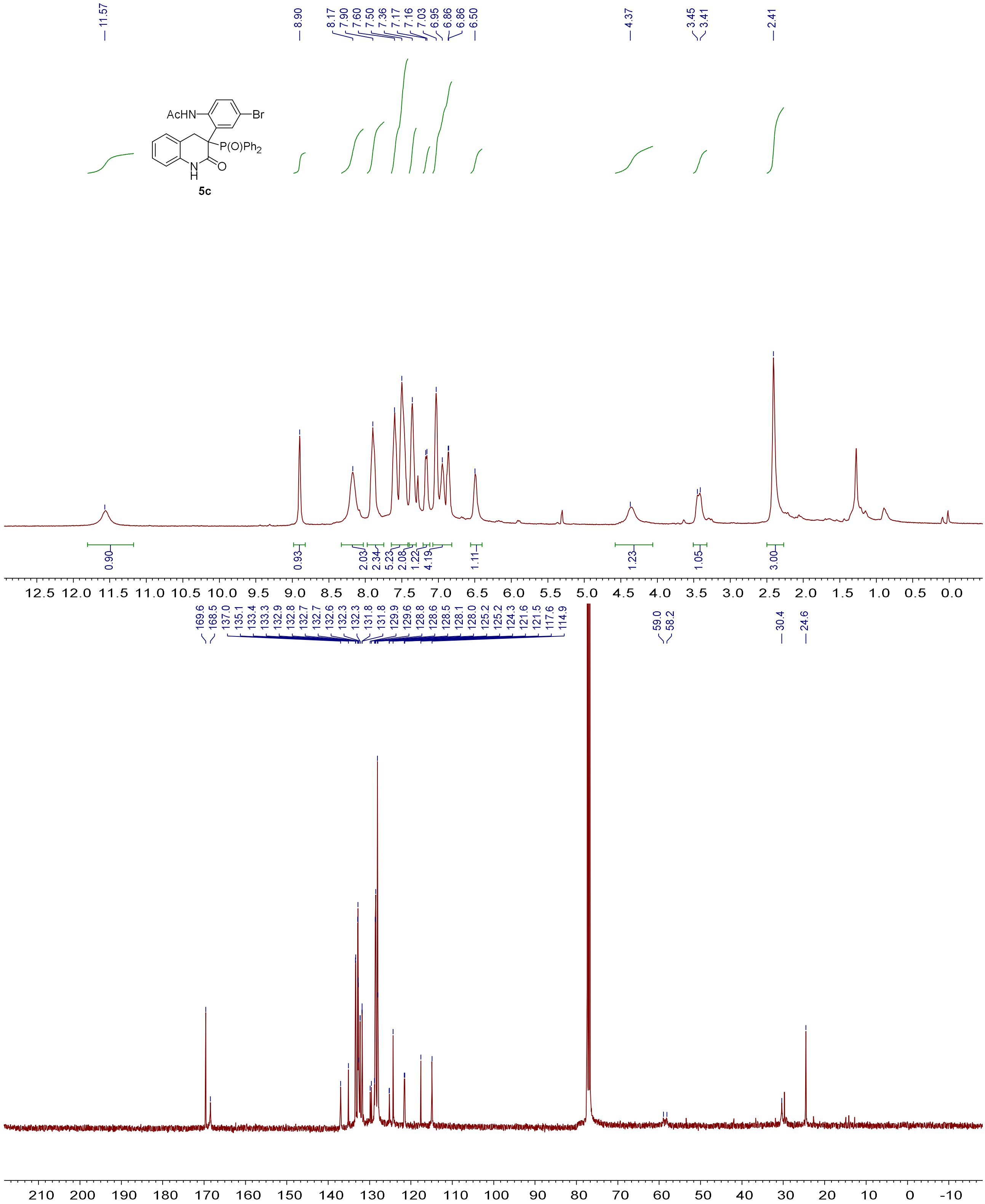

11.57
8.90
8.17
7.90
7.60
7.50
7.36
7.17
7.16
7.03
6.95
6.86
6.50
4.37
3.45
3.41
2.41
AcHN
Br
P(O)Ph2
N
H
O
5c
0.90
0.93
2.03
2.34
5.23
2.08
1.22
4.19
1.11
1.23
1.05
3.00
12.5 12.0 11.5 11.0 10.5 10.0 9.5 9.0 8.5 8.0 7.5 7.0 6.5 6.0 5.5 5.0 4.5 4.0 3.5 3.0 2.5 2.0 1.5 1.0 0.5 0.0
169.6
168.5
137.0
135.1
133.4
133.3
132.9
132.8
132.7
132.7
132.6
132.3
132.3
131.8
131.8
129.9
129.6
128.8
128.6
128.5
128.1
128.0
125.2
125.2
124.3
121.6
121.5
117.6
114.9
59.0
58.2
30.4
24.6
210 200 190 180 170 160 150 140 130 120 110 100 90 80 70 60 50 40 30 20 10 0 -10

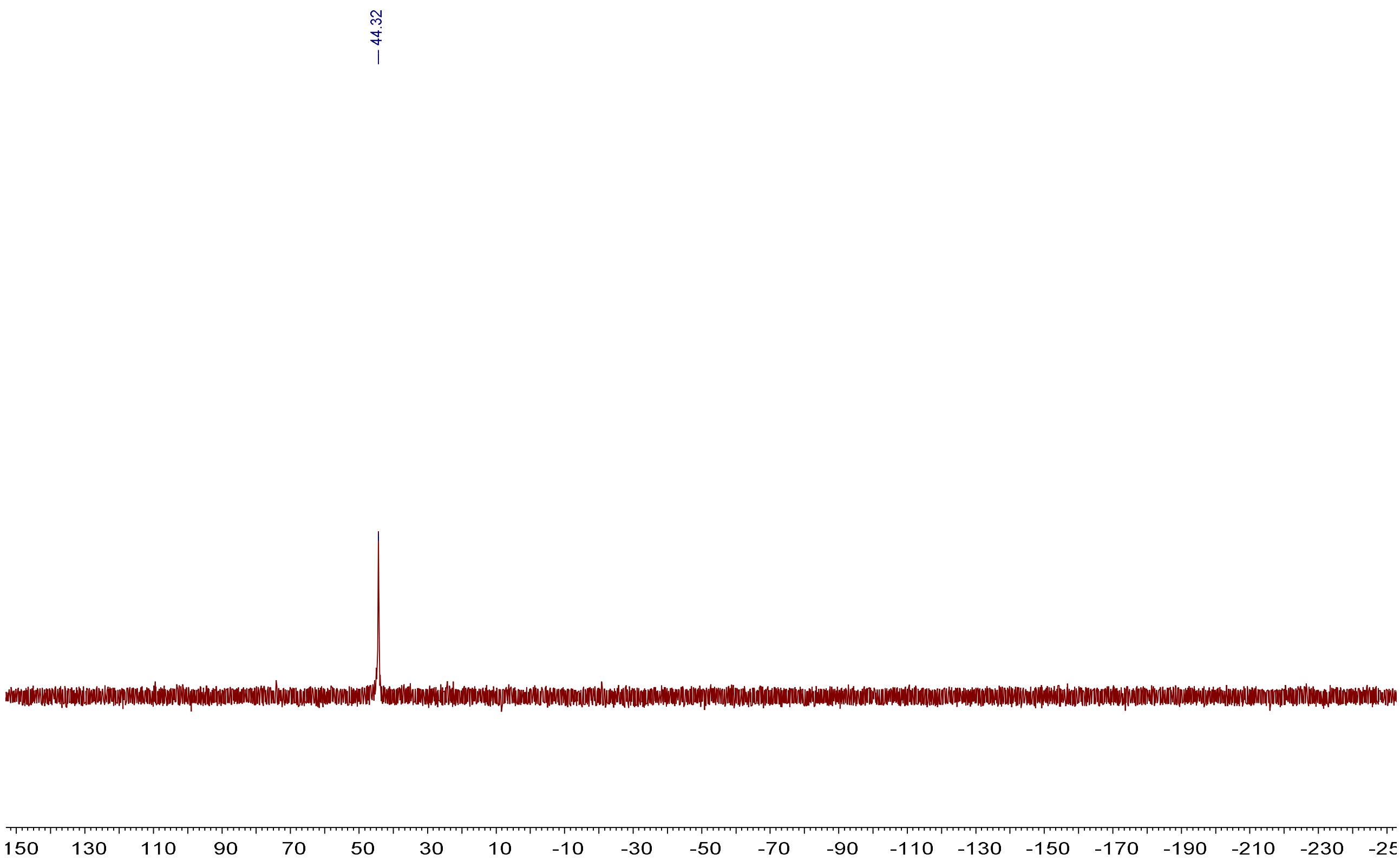
44.32
150 130 110 90 70 50 30 10 -10 -30 -50 -70 -90 -110 -130 -150 -170 -190 -210 -230 -25

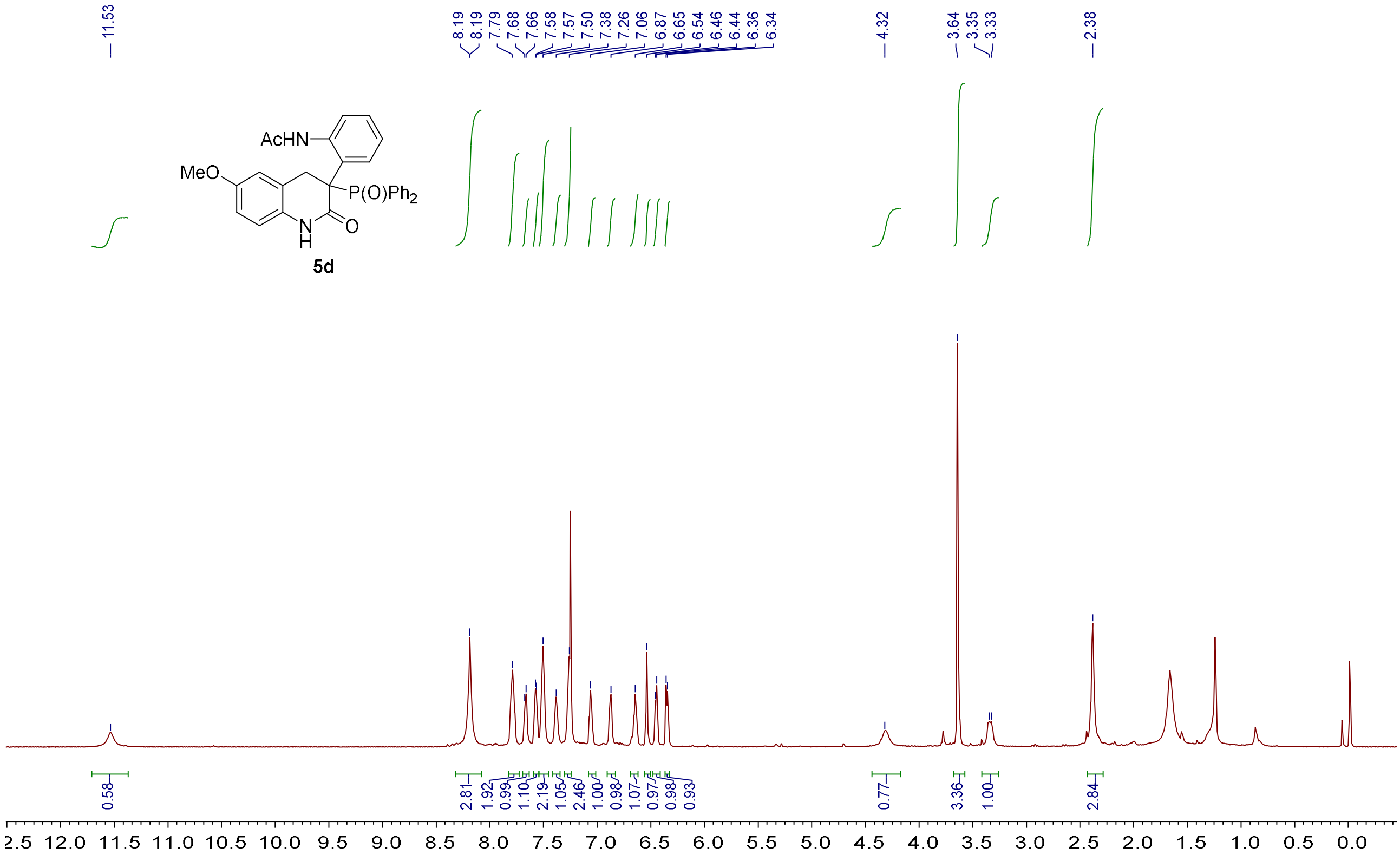

11.53
8.19 8.19 7.79 7.68 7.66 7.58 7.57 7.50 7.38 7.26 7.06 6.87 6.65 6.54 6.46 6.44 6.36 6.34
4.32
3.64 3.35 3.33
2.38
AcHN
MeO
P(O)Ph2
N H
O
5d
0.58
2.81 1.92 0.99 1.10 2.19 1.05 2.46 1.00 0.98 1.07 0.97 0.98 0.93
0.77
3.36 1.00
2.84
2.5 12.0 11.5 11.0 10.5 10.0 9.5 9.0 8.5 8.0 7.5 7.0 6.5 6.0 5.5 5.0 4.5 4.0 3.5 3.0 2.5 2.0 1.5 1.0 0.5 0.0

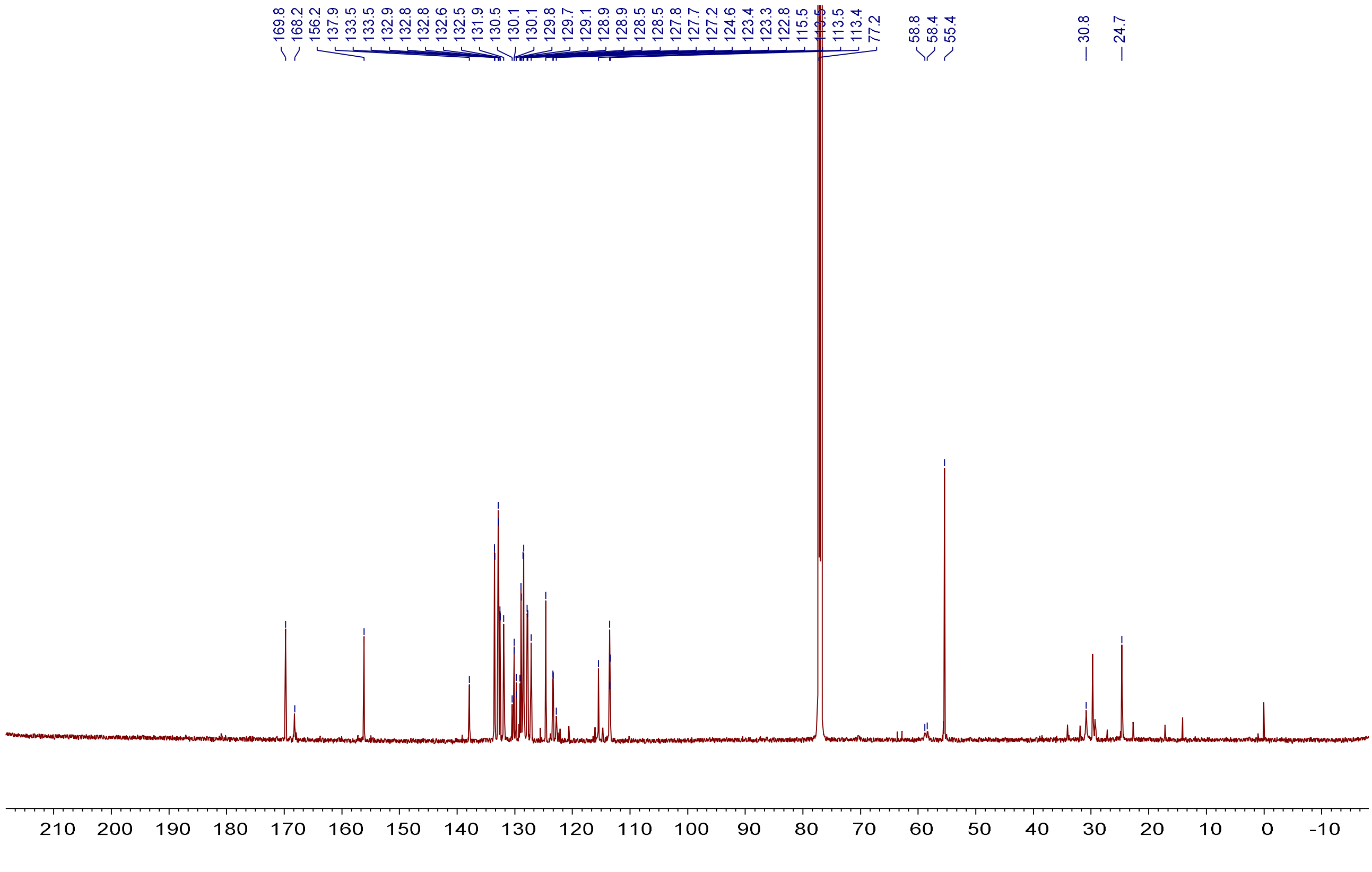

169.8 168.2 156.2 137.9 133.5 133.5 132.9 132.8 132.8 132.6 132.5 131.9 130.5 130.1 130.1 129.8 129.7 129.1 128.9 128.9 128.5 128.5 127.8 127.7 127.2 124.6 123.4 123.3 122.8 115.5 113.5 113.4 77.2
58.8 58.4 55.4
30.8 24.7
210 200 190 180 170 160 150 140 130 120 110 100 90 80 70 60 50 40 30 20 10 0 -10

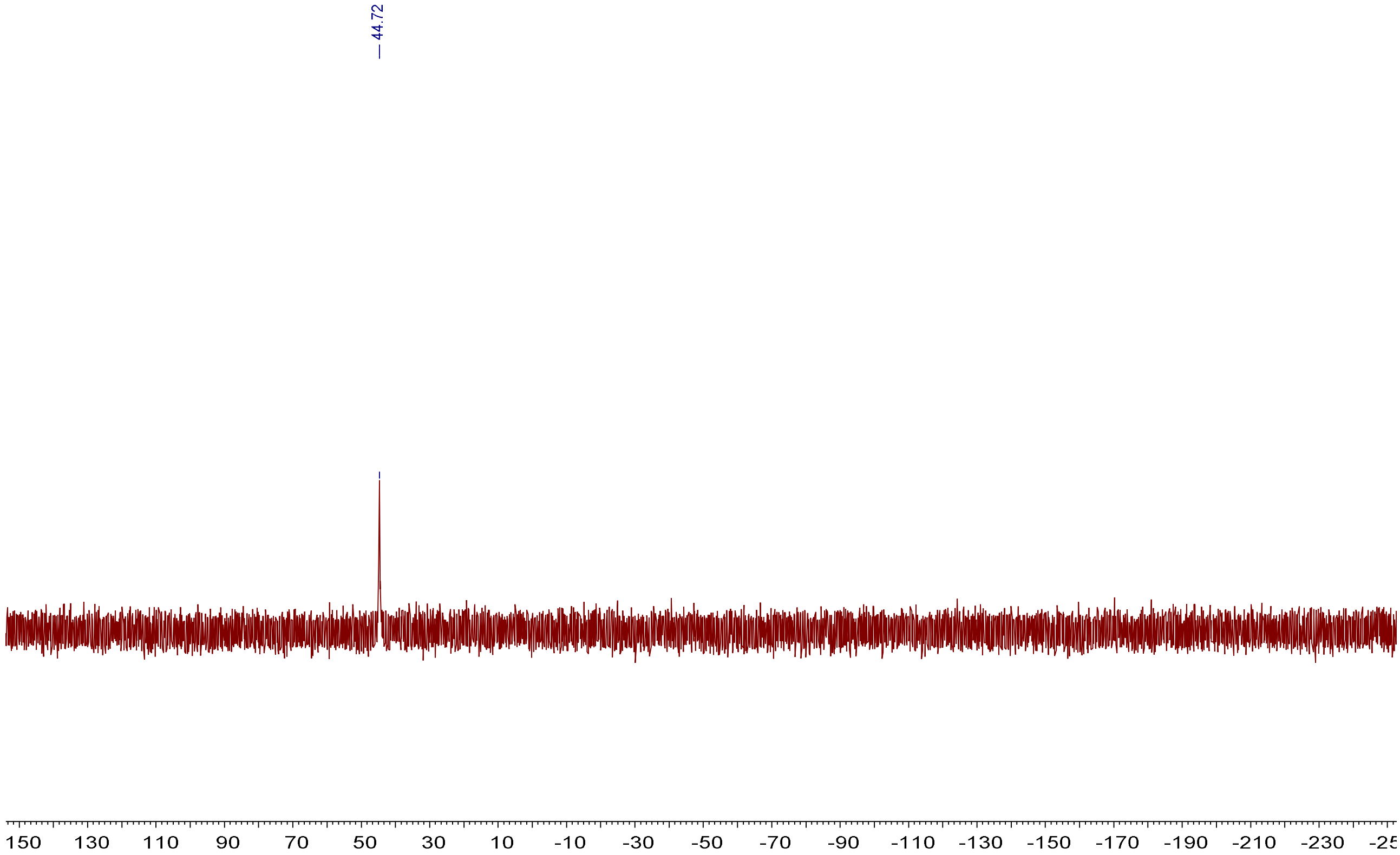
44.72
150
130
110
90
70
50
30
10
-10
-30
-50
-70
-90
-110
-130
-150
-170
-190
-210
-230
-25

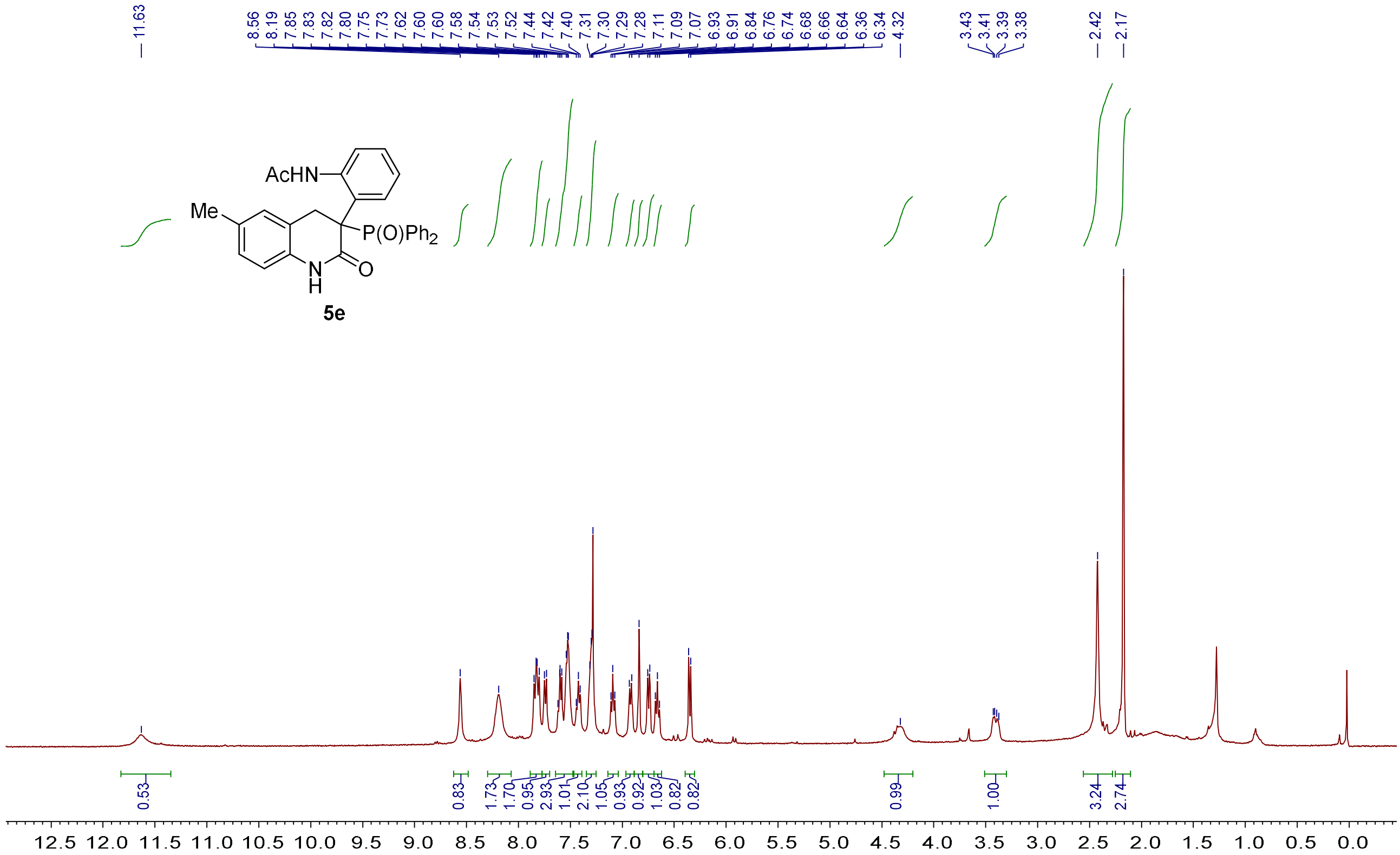
11.63
8.56 8.19 7.85 7.83 7.82 7.80 7.75 7.73 7.62 7.60 7.60 7.58 7.54 7.53 7.52 7.44 7.42 7.40 7.31 7.30 7.29 7.28 7.11 7.09 7.07 6.93 6.91 6.84 6.76 6.74 6.68 6.66 6.64 6.36 6.34 4.32
3.43 3.41 3.39 3.38
2.42 2.17
AcHN
Me
P(O)Ph2
N
H
O
5e
0.53 0.83 1.73 1.70 0.95 2.93 1.01 2.10 1.05 0.93 0.92 1.03 0.82 0.82 0.99 1.00 3.24 2.74
12.5 12.0 11.5 11.0 10.5 10.0 9.5 9.0 8.5 8.0 7.5 7.0 6.5 6.0 5.5 5.0 4.5 4.0 3.5 3.0 2.5 2.0 1.5 1.0 0.5 0.0

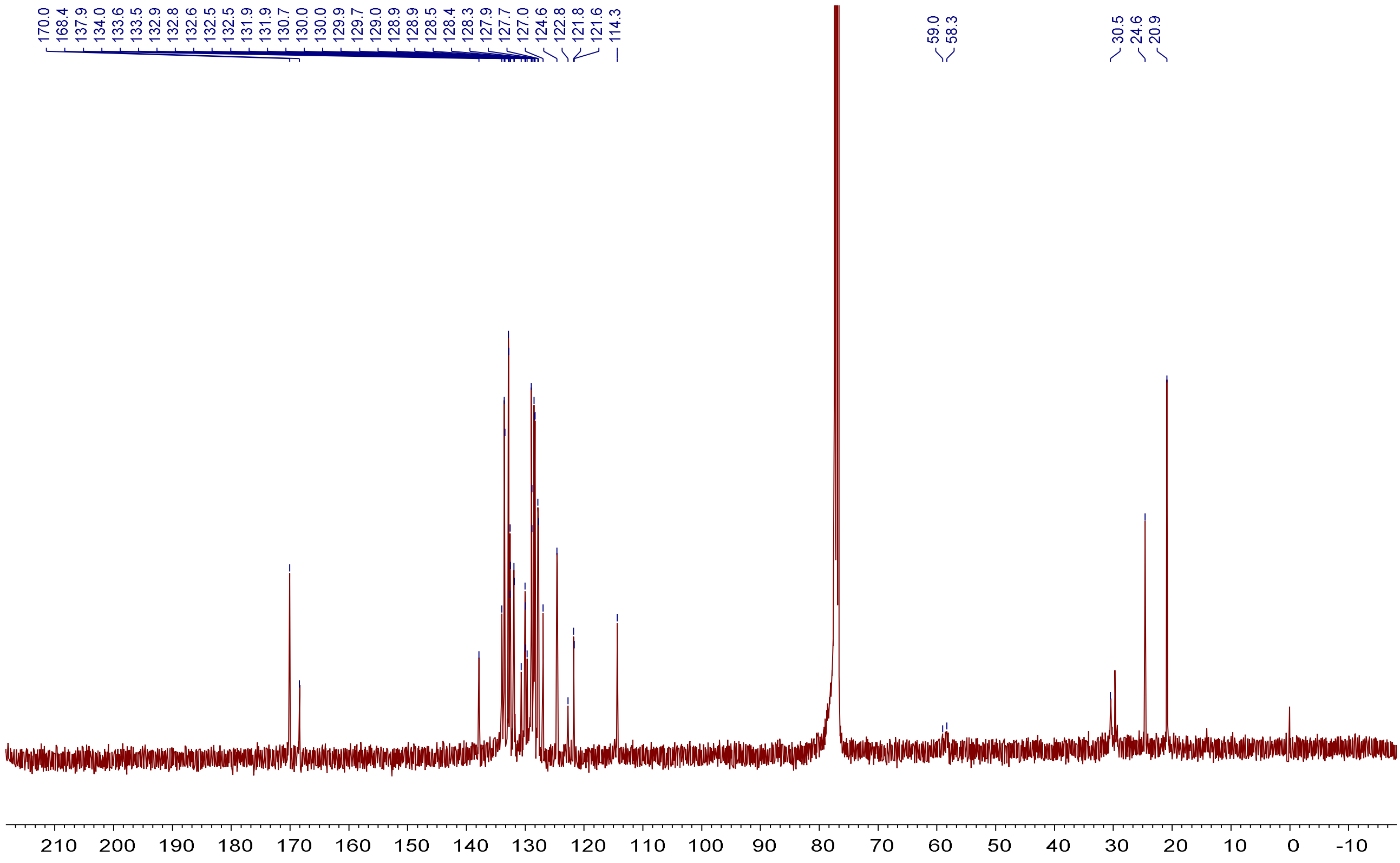
170.0 168.4 137.9 134.0 133.6 133.5 132.9 132.8 132.6 132.5 132.5 131.9 131.9 130.7 130.0 130.0 129.9 129.7 129.0 128.9 128.9 128.5 128.4 128.3 127.9 127.7 127.0 124.6 122.8 121.8 121.6 114.3
59.0 58.3
30.5 24.6 20.9
210 200 190 180 170 160 150 140 130 120 110 100 90 80 70 60 50 40 30 20 10 0 -10

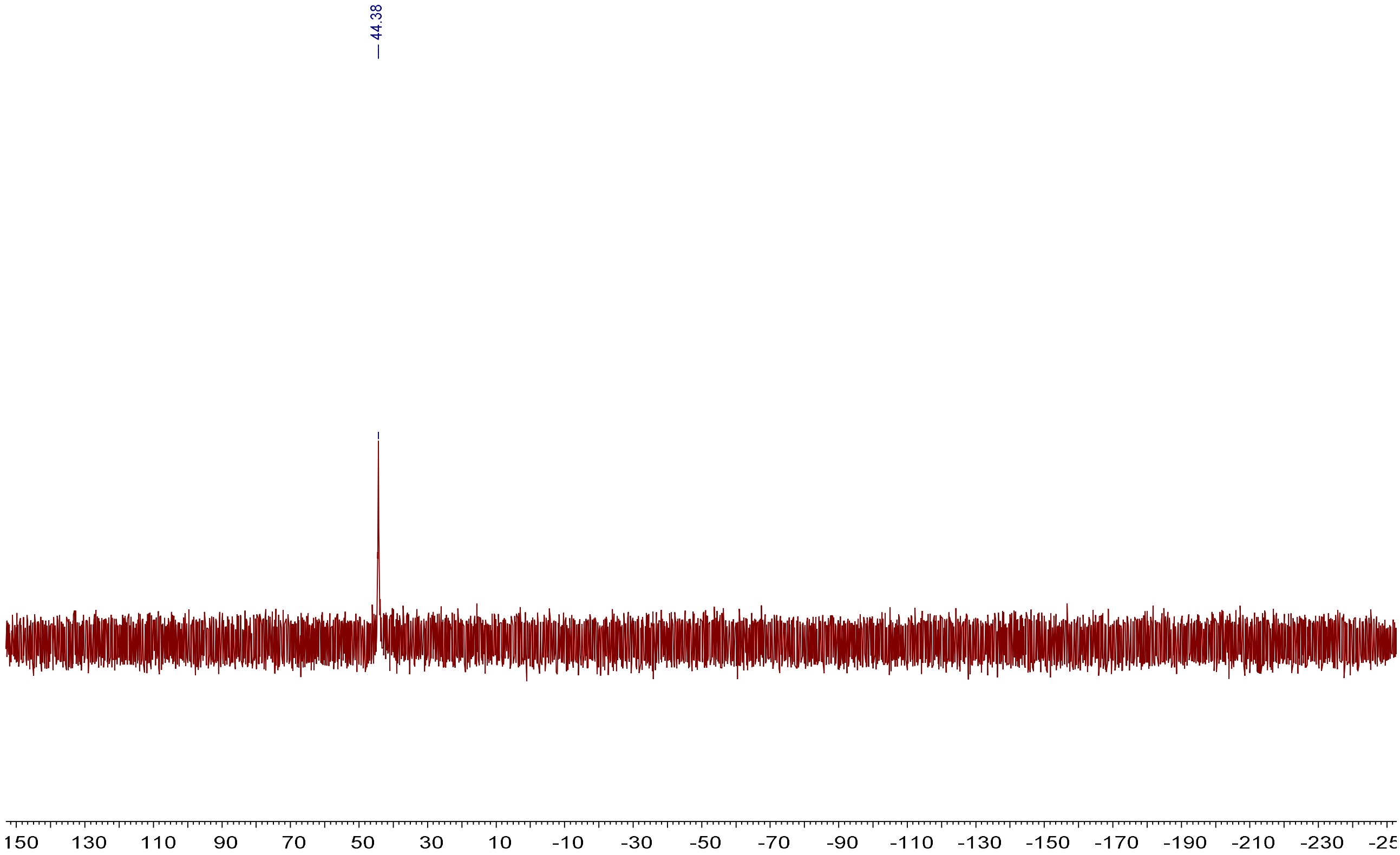
44.38
150
130
110
90
70
50
30
10
-10
-30
-50
-70
-90
-110
-130
-150
-170
-190
-210
-230
-25

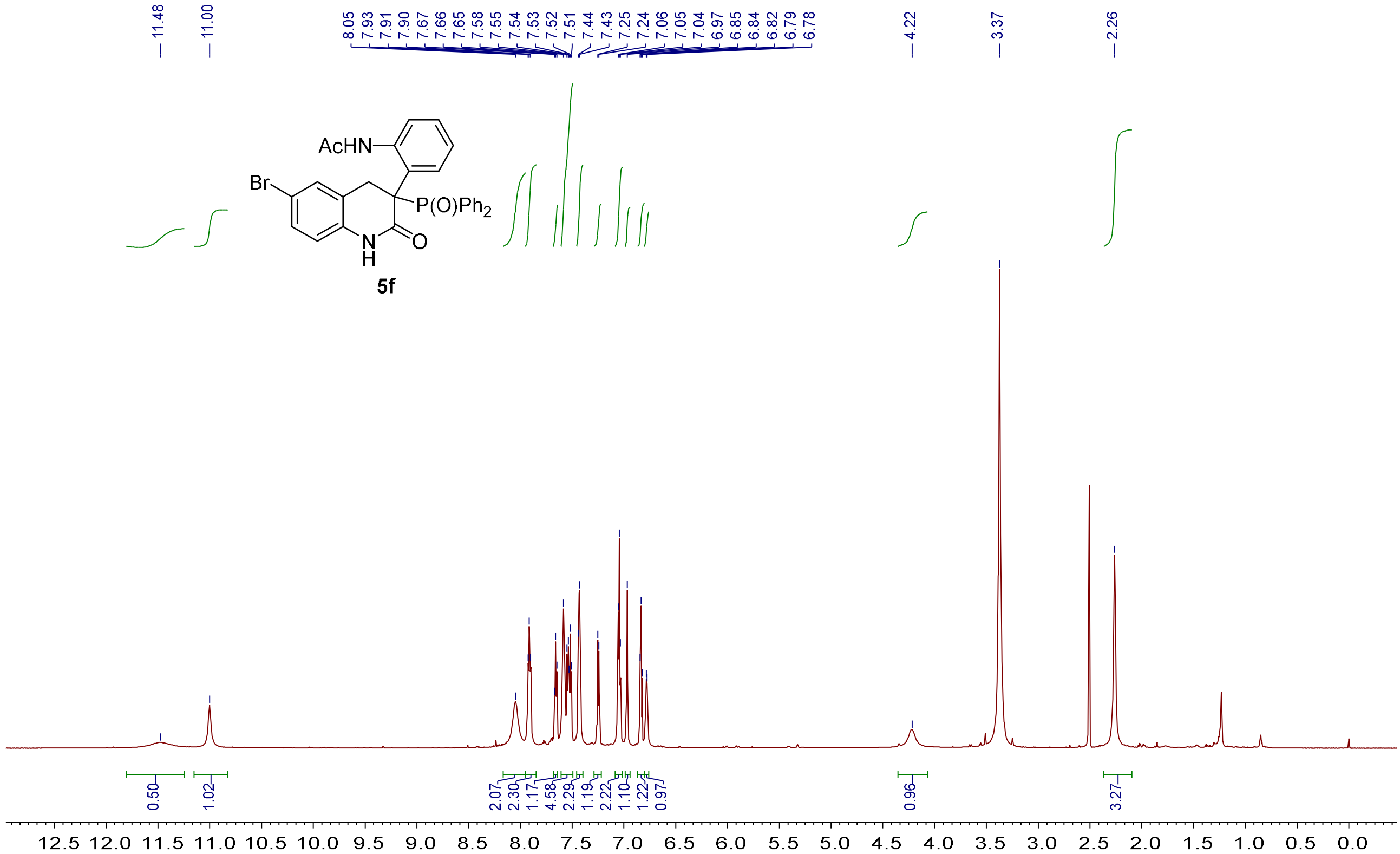
11.48
11.00
8.05 7.93 7.91 7.90 7.67 7.66 7.65 7.58 7.55 7.54 7.53 7.52 7.51 7.44 7.43 7.25 7.24 7.06 7.05 7.04 6.97 6.85 6.84 6.82 6.79 6.78
4.22
3.37
2.26
AcHN
Br
P(O)Ph2
N
H
O
5f
0.50
1.02
2.07 2.30 1.17 4.58 2.29 1.19 2.22 1.10 1.22 0.97
0.96
3.27
12.5 12.0 11.5 11.0 10.5 10.0 9.5 9.0 8.5 8.0 7.5 7.0 6.5 6.0 5.5 5.0 4.5 4.0 3.5 3.0 2.5 2.0 1.5 1.0 0.5 0.0

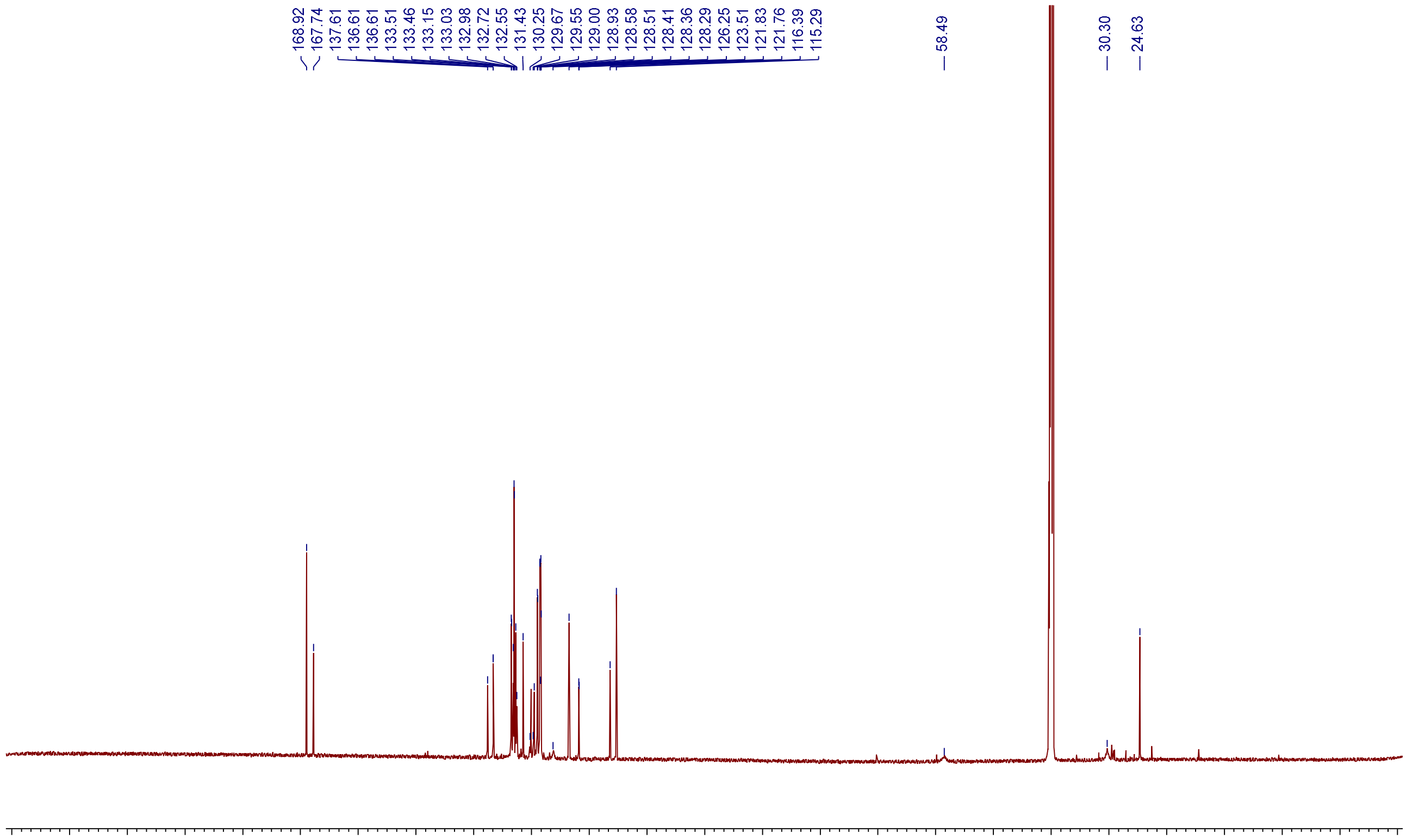
168.92 167.74 137.61 136.61 136.61 133.51 133.46 133.15 133.03 132.98 132.72 132.55 131.43 130.25 129.67 129.55 129.00 128.93 128.58 128.51 128.41 128.36 128.29 126.25 123.51 121.83 121.76 116.39 115.29
58.49
30.30
24.63
220 210 200 190 180 170 160 150 140 130 120 110 100 90 80 70 60 50 40 30 20 10 0 -10 -2

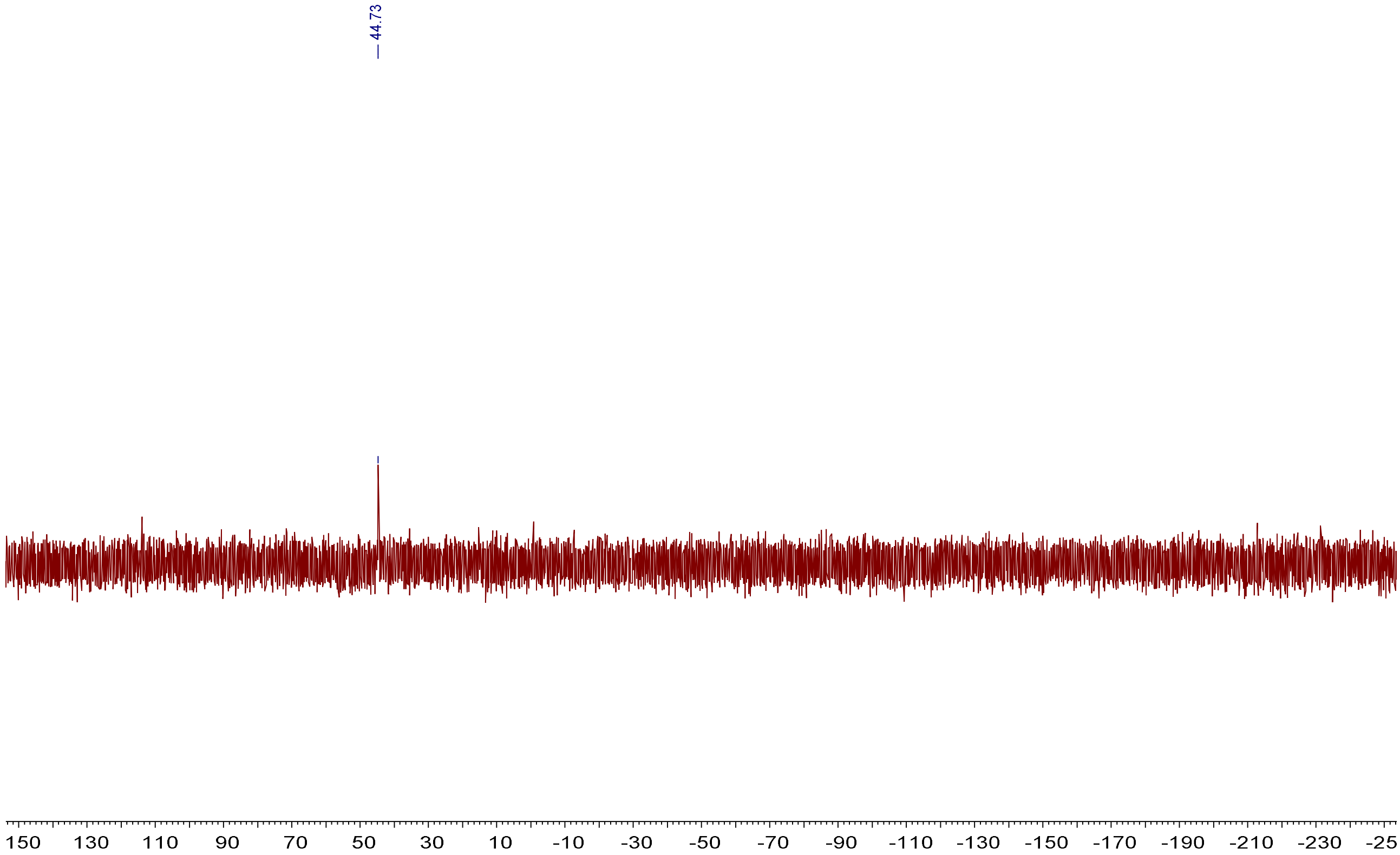
44.73
150
130
110
90
70
50
30
10
-10
-30
-50
-70
-90
-110
-130
-150
-170
-190
-210
-230
-25

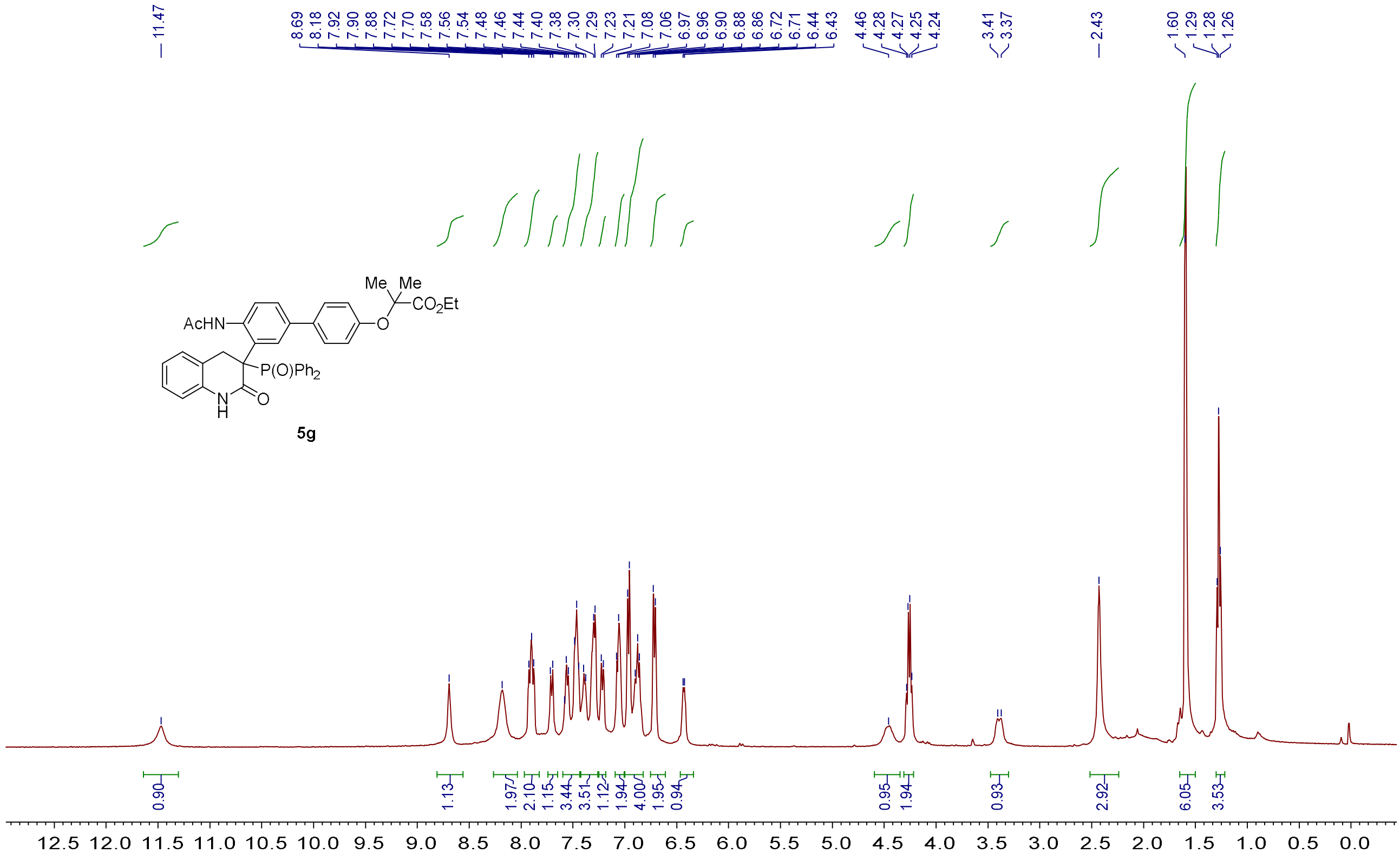
11.47
8.69 8.18 7.92 7.90 7.88 7.72 7.70 7.58 7.56 7.54 7.48 7.46 7.44 7.40 7.38 7.30 7.29 7.23 7.21 7.08 7.06 6.97 6.96 6.90 6.88 6.86 6.72 6.71 6.44 6.43
4.46 4.28 4.27 4.25 4.24
3.41 3.37
2.43
1.60 1.29 1.28 1.26
Me Me
CO2Et
O
AcHN
P(O)Ph2
N
H
O
5g
0.90 1.13 1.97 2.10 1.15 3.44 3.51 1.12 1.94 4.00 1.95 0.94 0.95 1.94 0.93 2.92 6.05 3.53
12.5 12.0 11.5 11.0 10.5 10.0 9.5 9.0 8.5 8.0 7.5 7.0 6.5 6.0 5.5 5.0 4.5 4.0 3.5 3.0 2.5 2.0 1.5 1.0 0.5 0.0

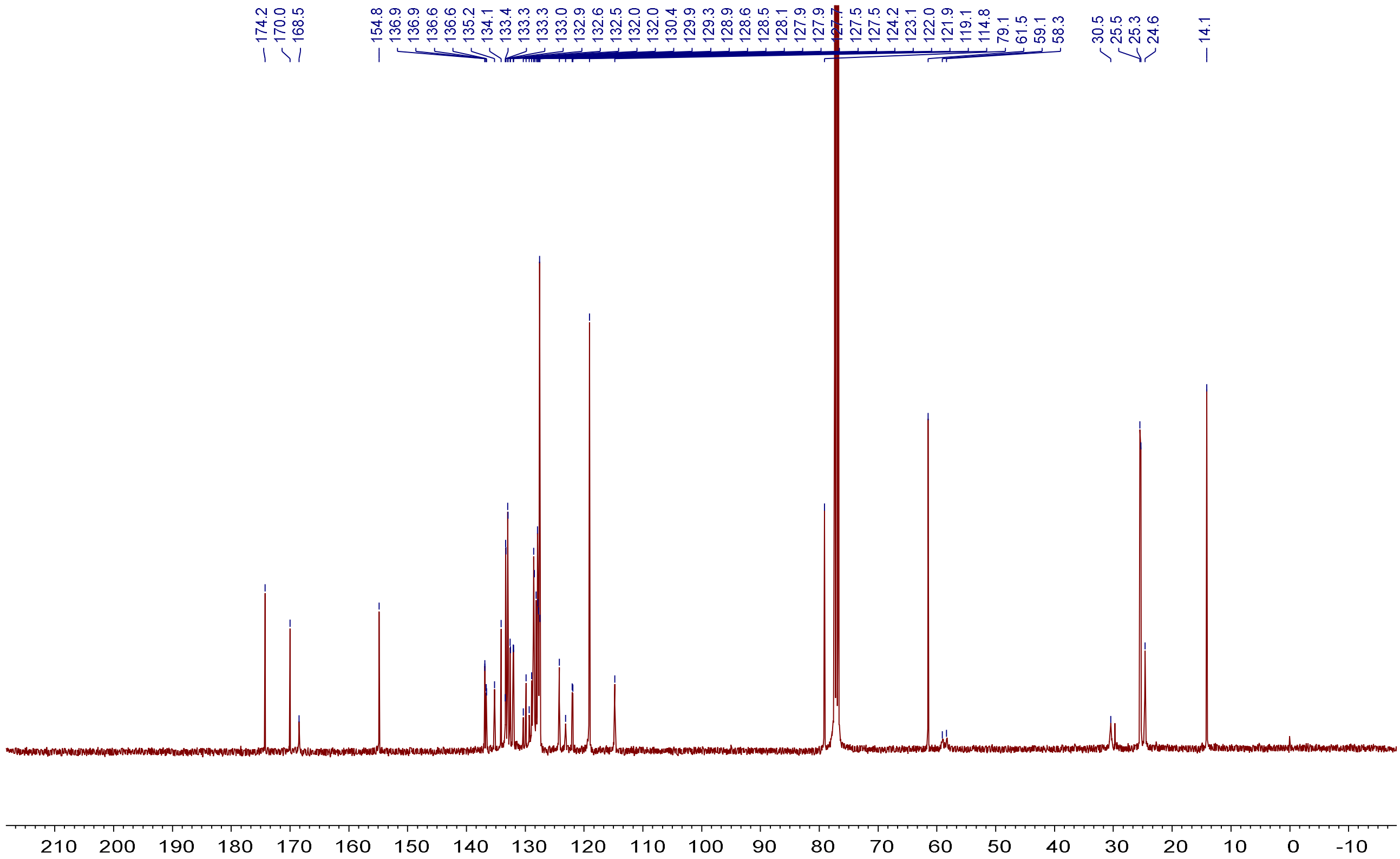
174.2 170.0 168.5
154.8 136.9 136.9 136.6 136.6 135.2 134.1 133.4 133.3 133.3 133.0 132.9 132.6 132.5 132.0 132.0 130.4 129.9 129.3 128.9 128.6 128.5 128.1 127.9 127.9 127.7 127.5 127.5 124.2 123.1 122.0 121.9 119.1 114.8 79.1 61.5 59.1 58.3
30.5 25.5 25.3 24.6
14.1
210 200 190 180 170 160 150 140 130 120 110 100 90 80 70 60 50 40 30 20 10 0 -10

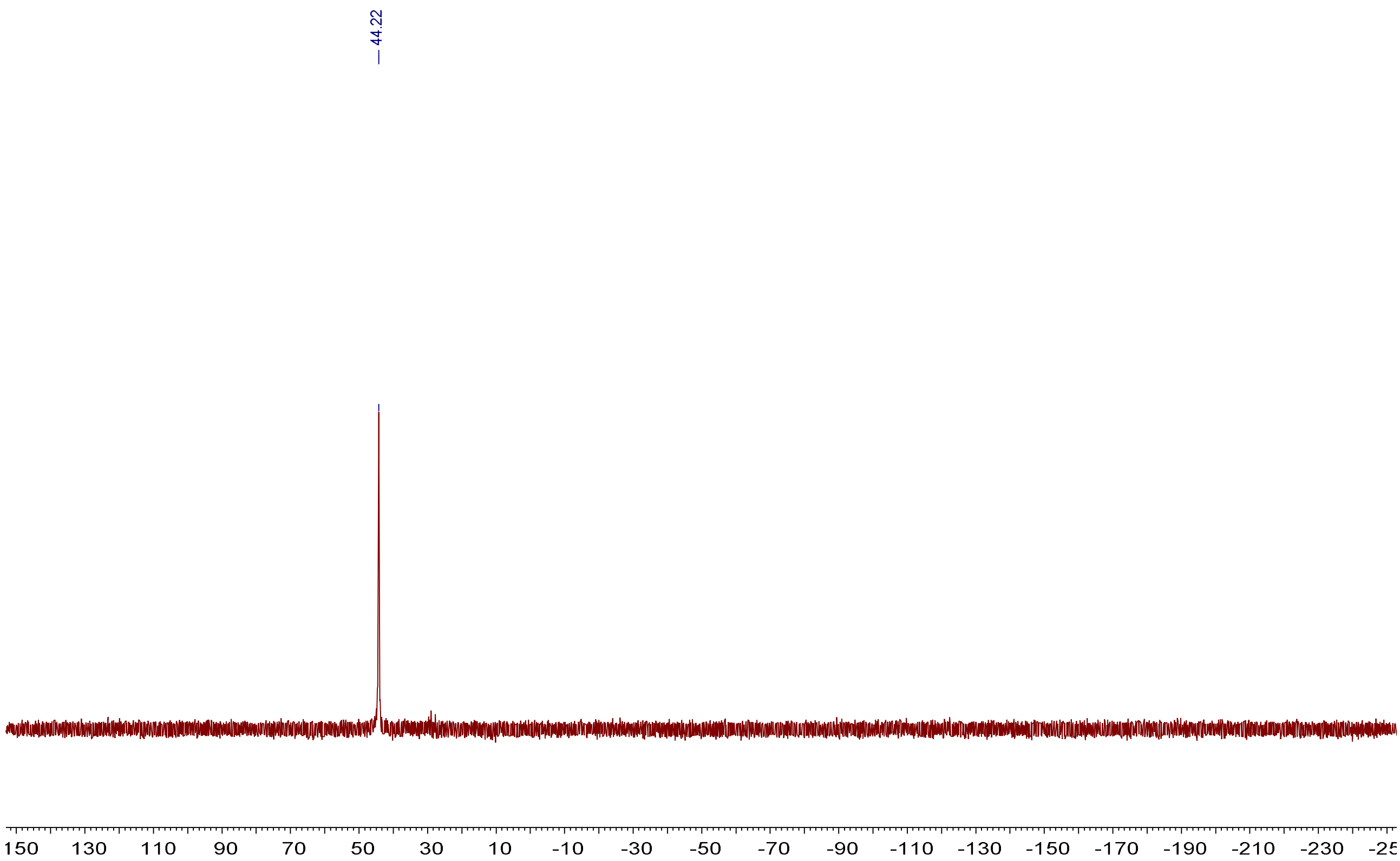
44.22
150
130
110
90
70
50
30
10
-10
-30
-50
-70
-90
-110
-130
-150
-170
-190
-210
-230
-25

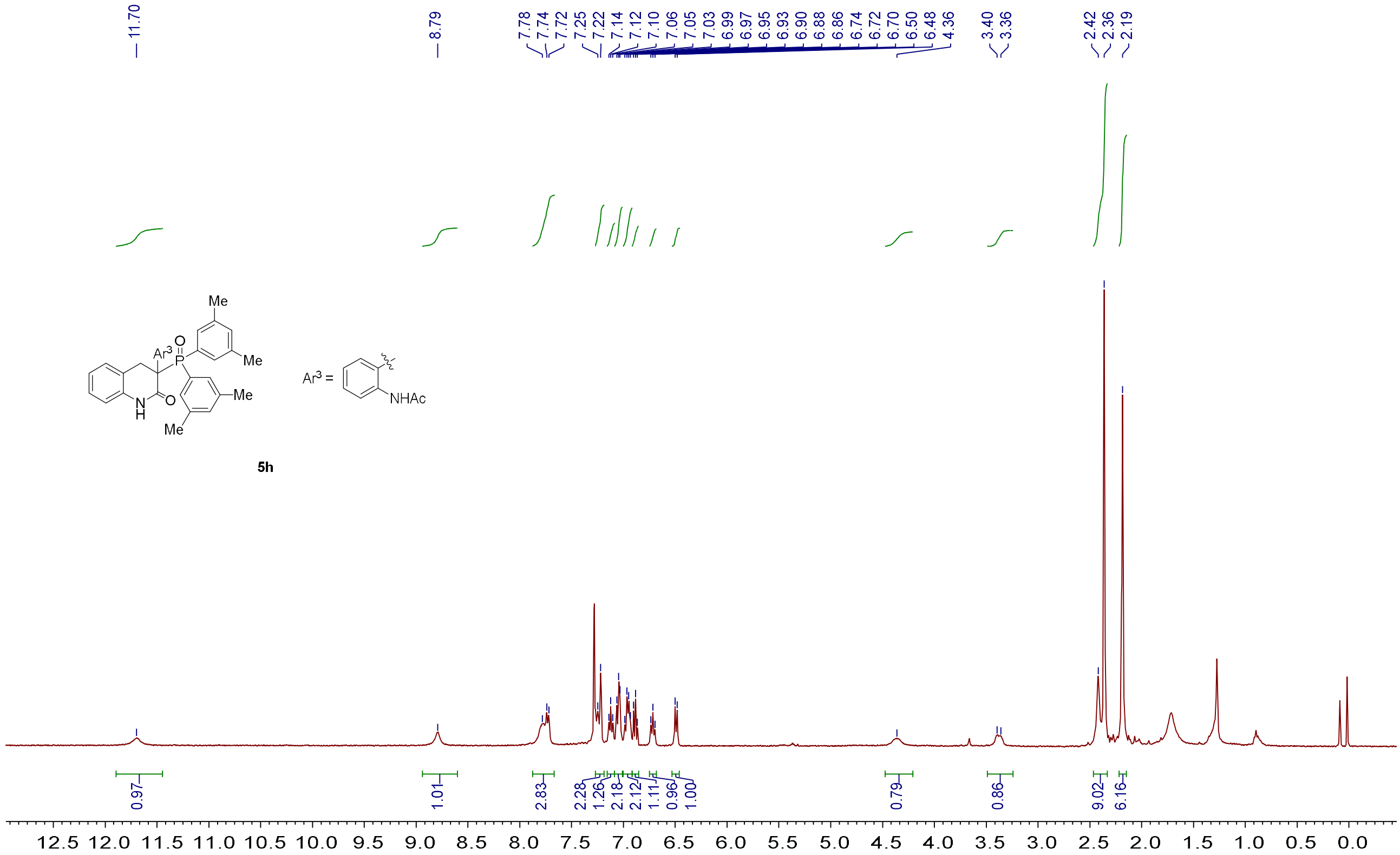
11.70
8.79
7.78
7.74
7.72
7.25
7.22
7.14
7.12
7.10
7.06
7.05
7.03
6.99
6.97
6.95
6.93
6.90
6.88
6.86
6.74
6.72
6.70
6.50
6.48
4.36
3.40
3.36
2.42
2.36
2.19
0.97
1.01
2.83
2.28
1.26
2.18
2.12
1.11
0.96
1.00
0.79
0.86
9.02
6.16
12.5 12.0 11.5 11.0 10.5 10.0 9.5 9.0 8.5 8.0 7.5 7.0 6.5 6.0 5.5 5.0 4.5 4.0 3.5 3.0 2.5 2.0 1.5 1.0 0.5 0.0
Me
Ar3
O
P
Me
Me
N
H
O
Me
Ar3 =
NHAc

5h

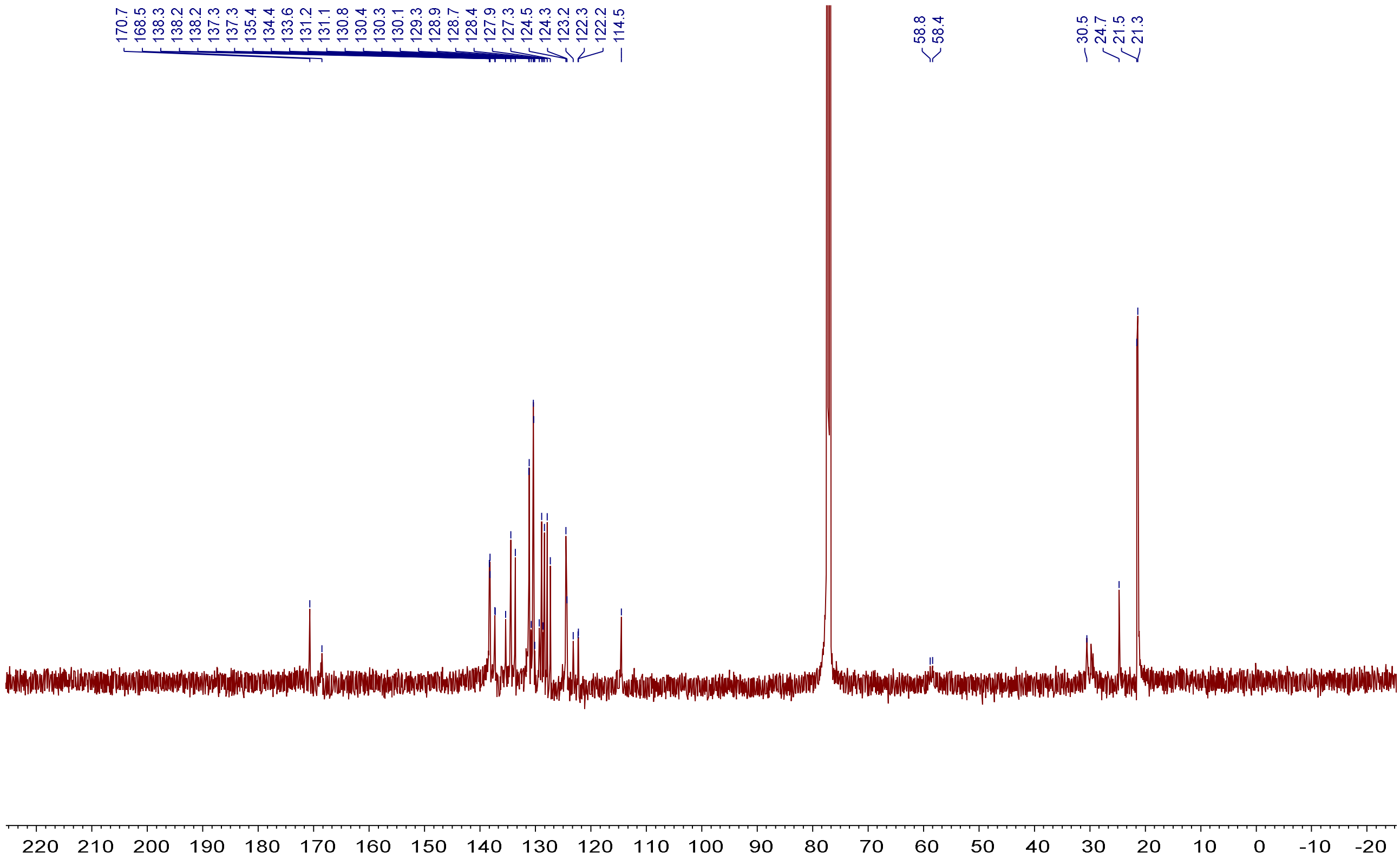
170.7
168.5
138.3
138.2
138.2
137.3
137.3
135.4
134.4
133.6
131.2
131.1
130.8
130.4
130.3
130.1
129.3
128.9
128.7
128.4
127.9
127.3
124.5
124.3
123.2
122.3
122.2
114.5
58.8
58.4
30.5
24.7
21.5
21.3
220 210 200 190 180 170 160 150 140 130 120 110 100 90 80 70 60 50 40 30 20 10 0 -10 -20

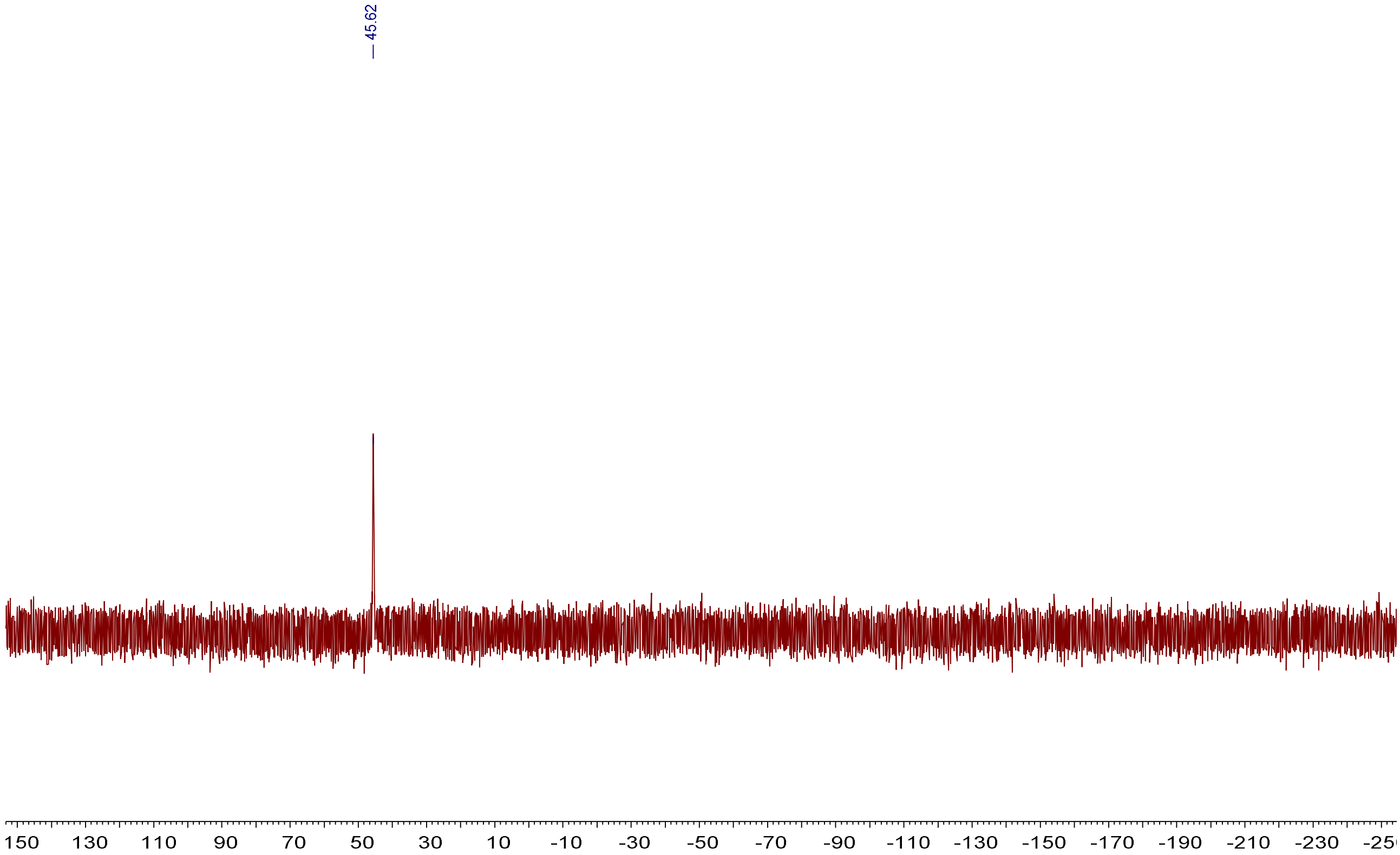
45.62
150
130
110
90
70
50
30
10
-10
-30
-50
-70
-90
-110
-130
-150
-170
-190
-210
-230

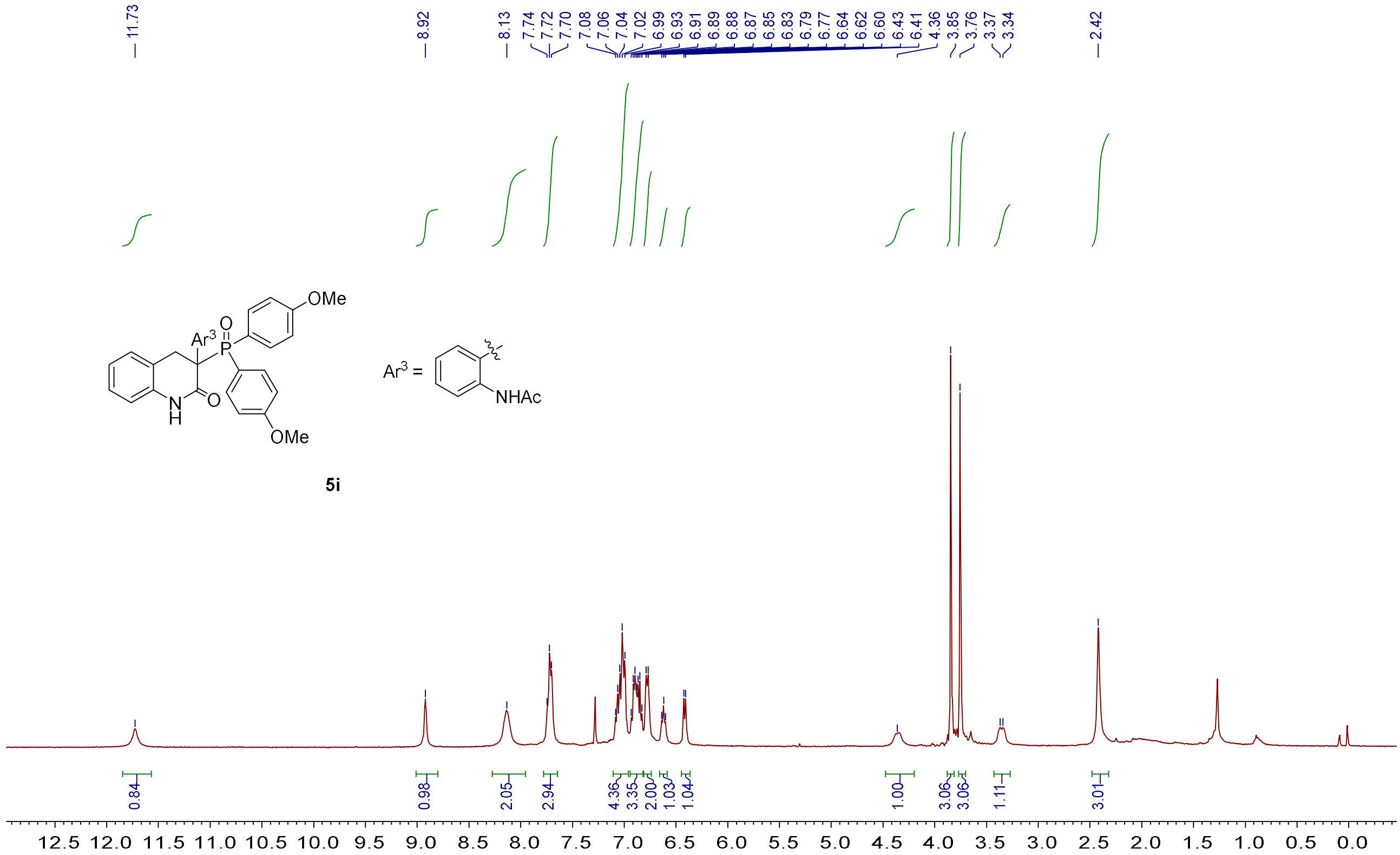
11.73
8.92
8.13 7.74 7.72 7.70 7.08 7.06 7.04 7.02 6.99 6.93 6.91 6.89 6.88 6.87 6.85 6.83 6.79 6.77 6.64 6.62 6.60 6.43 6.41 4.36 3.85 3.76 3.37 3.34
2.42
0.84 0.98 2.05 2.94 4.36 3.35 2.00 1.03 1.04 1.00 3.06 3.06 1.11 3.01
12.5 12.0 11.5 11.0 10.5 10.0 9.5 9.0 8.5 8.0 7.5 7.0 6.5 6.0 5.5 5.0 4.5 4.0 3.5 3.0 2.5 2.0 1.5 1.0 0.5 0.0
OMe
Ar3
O
P
N
H
O
OMe
Ar3 =
NHAc

5i

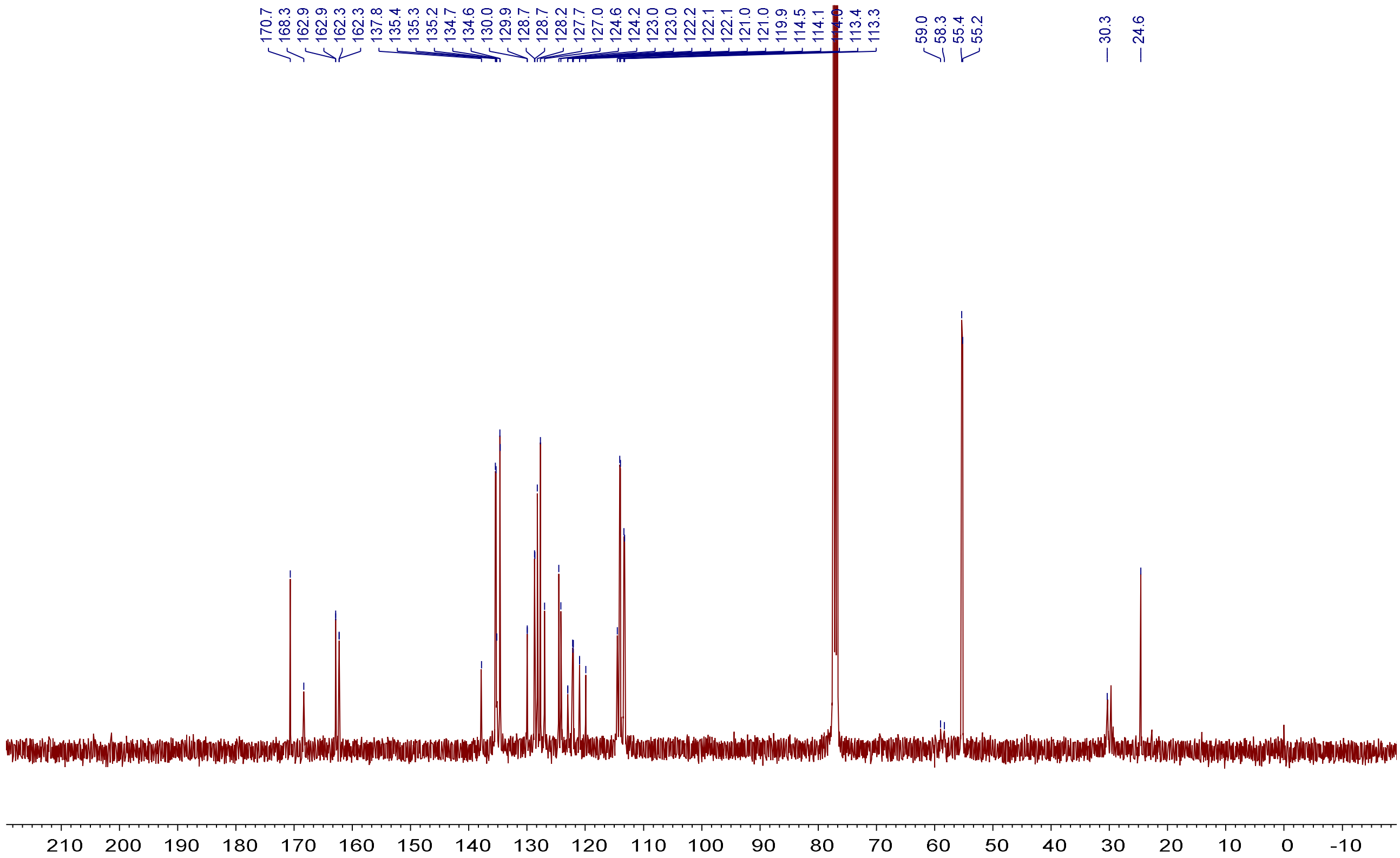
170.7 168.3 162.9 162.9 162.3 162.3 137.8 135.4 135.3 135.2 134.7 134.6 130.0 129.9 128.7 128.7 128.2 127.7 127.0 124.6 124.2 123.0 123.0 122.2 122.1 122.1 121.0 121.0 119.9 114.5 114.1 114.0 113.4 113.3
59.0 58.3 55.4 55.2
30.3
24.6
210 200 190 180 170 160 150 140 130 120 110 100 90 80 70 60 50 40 30 20 10 0 -10

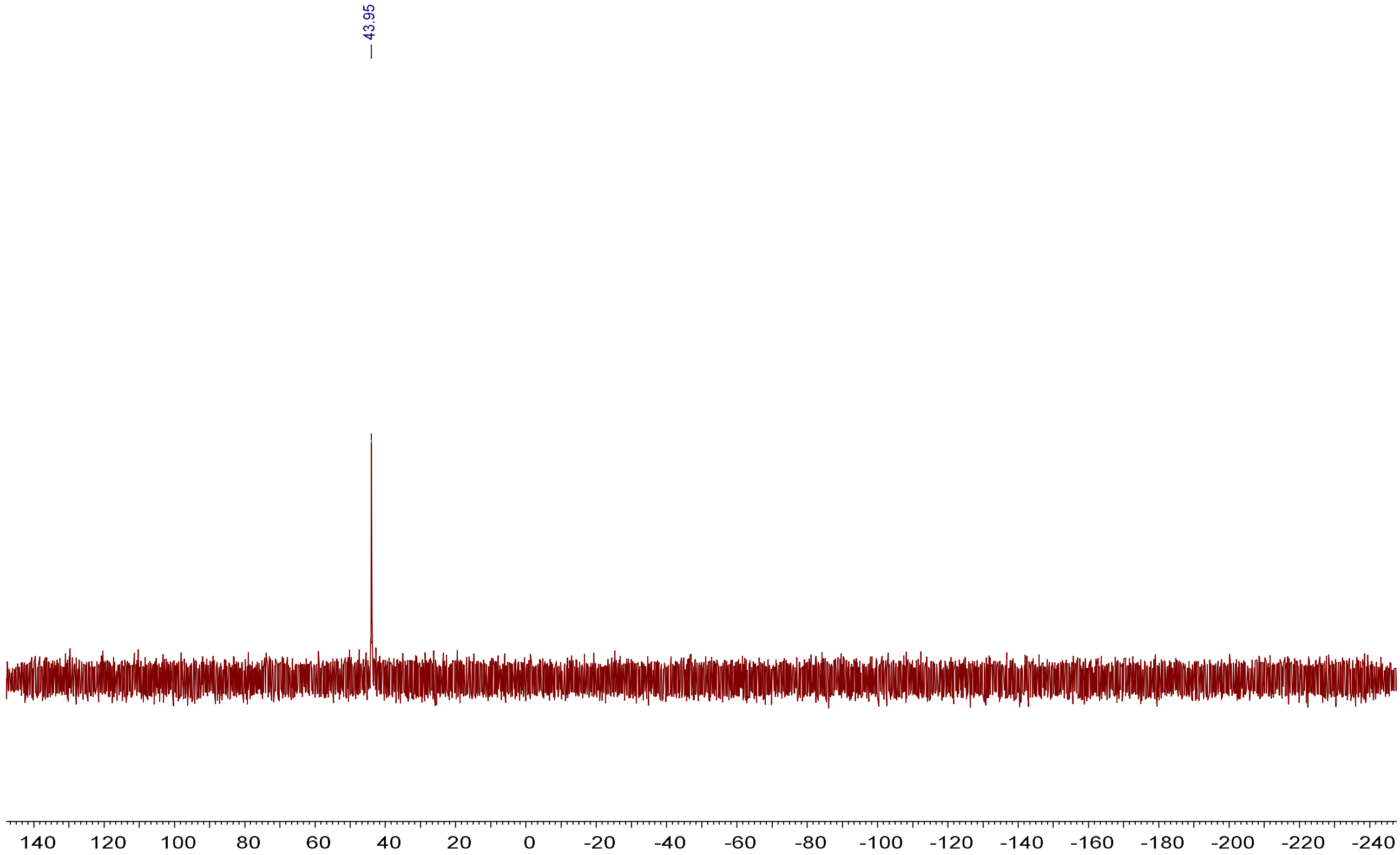
43.95
140 120 100 80 60 40 20 0 -20 -40 -60 -80 -100 -120 -140 -160 -180 -200 -220 -240

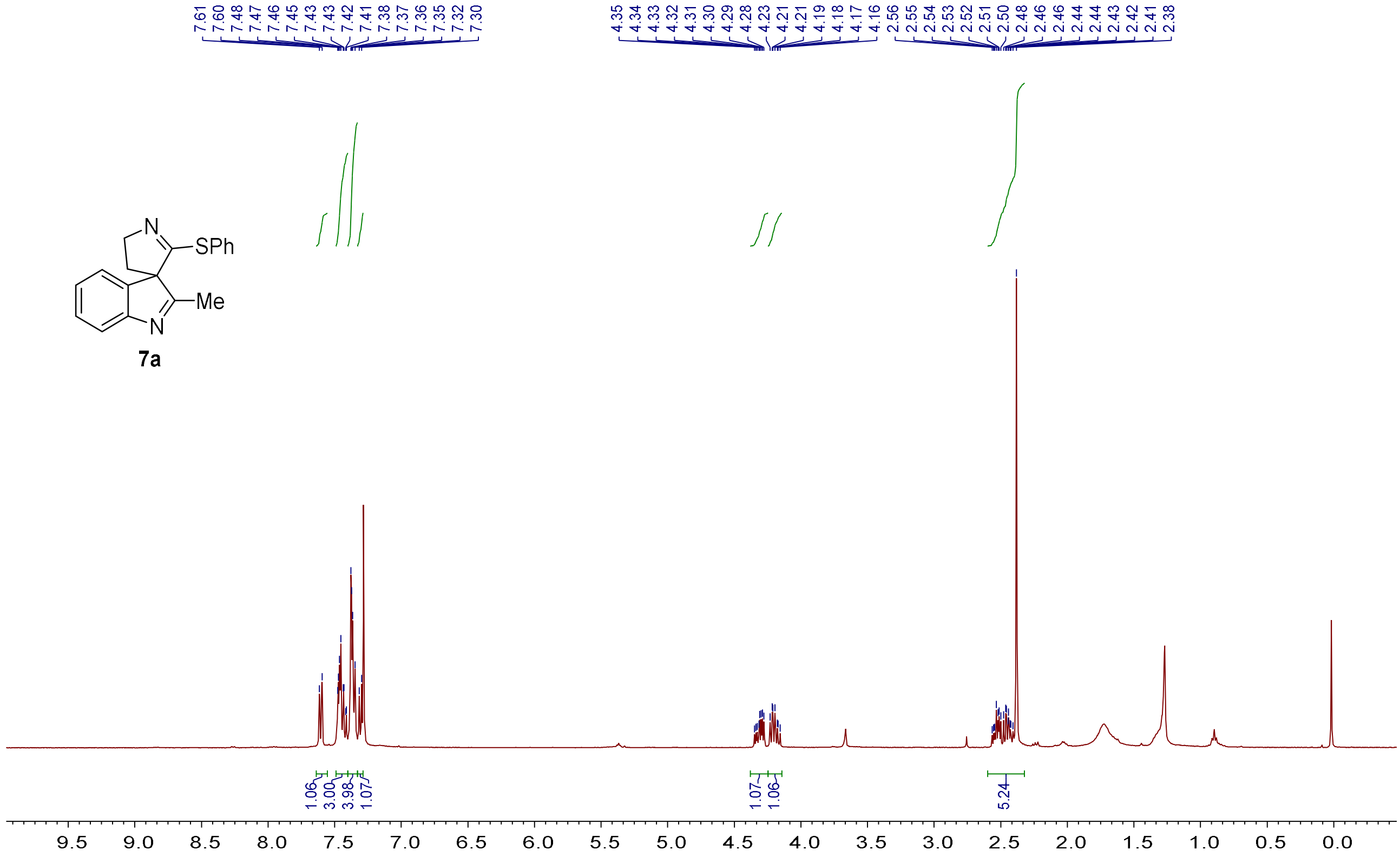

7.61 7.60 7.48 7.47 7.46 7.45 7.43 7.42 7.41 7.38 7.37 7.36 7.35 7.32 7.30
4.35 4.34 4.33 4.32 4.31 4.30 4.29 4.28 4.23 4.21 4.21 4.19 4.18 4.17 4.16
2.56 2.55 2.54 2.53 2.52 2.51 2.50 2.48 2.46 2.46 2.44 2.44 2.43 2.42 2.41 2.38
N
SPh
Me
N
1.06 3.00 3.98 1.07
1.07 1.06
5.24

7a

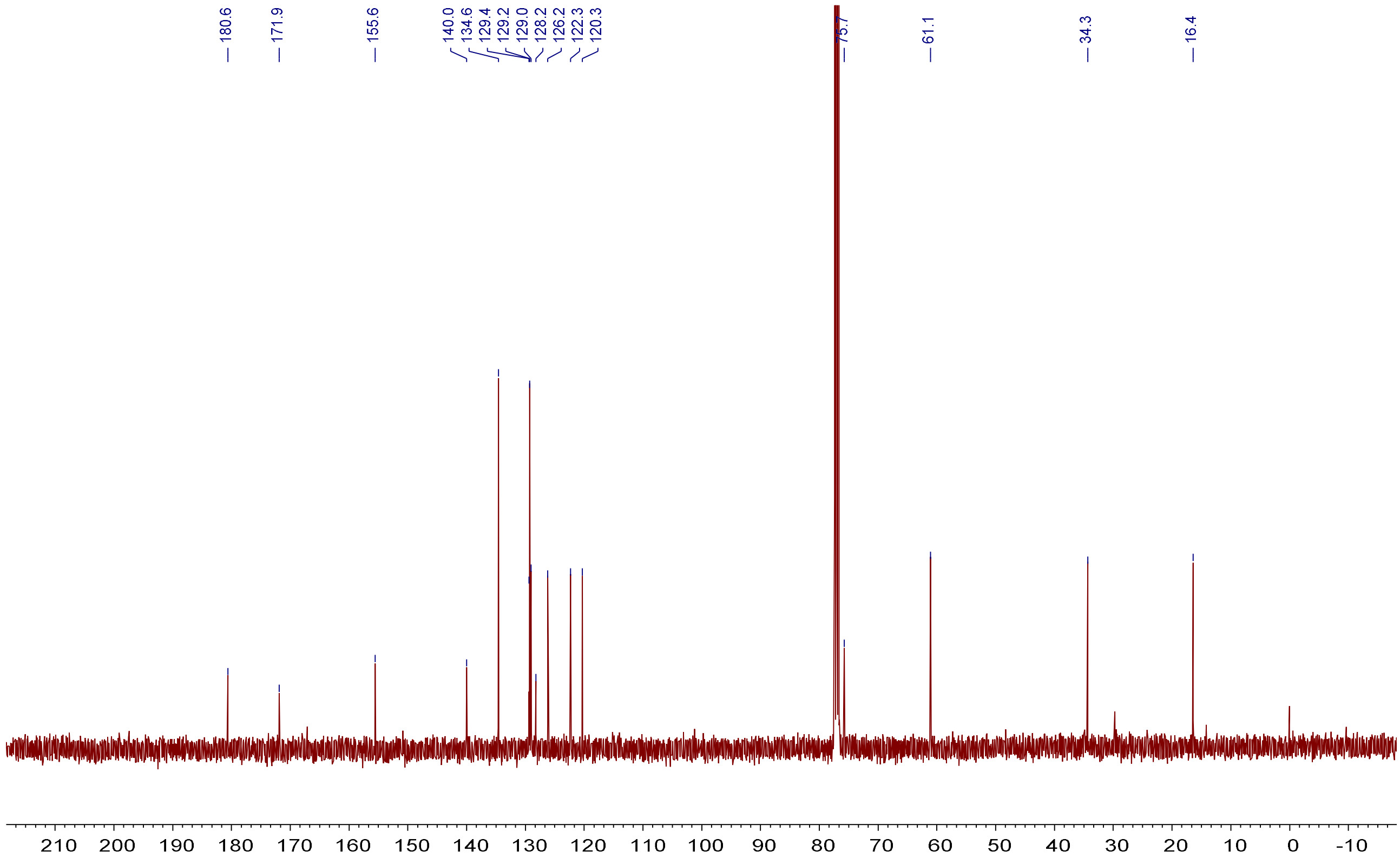

180.6
171.9
155.6
140.0 134.6 129.4 129.2 129.0 128.2 126.2 122.3 120.3
76.7
61.1
34.3
16.4

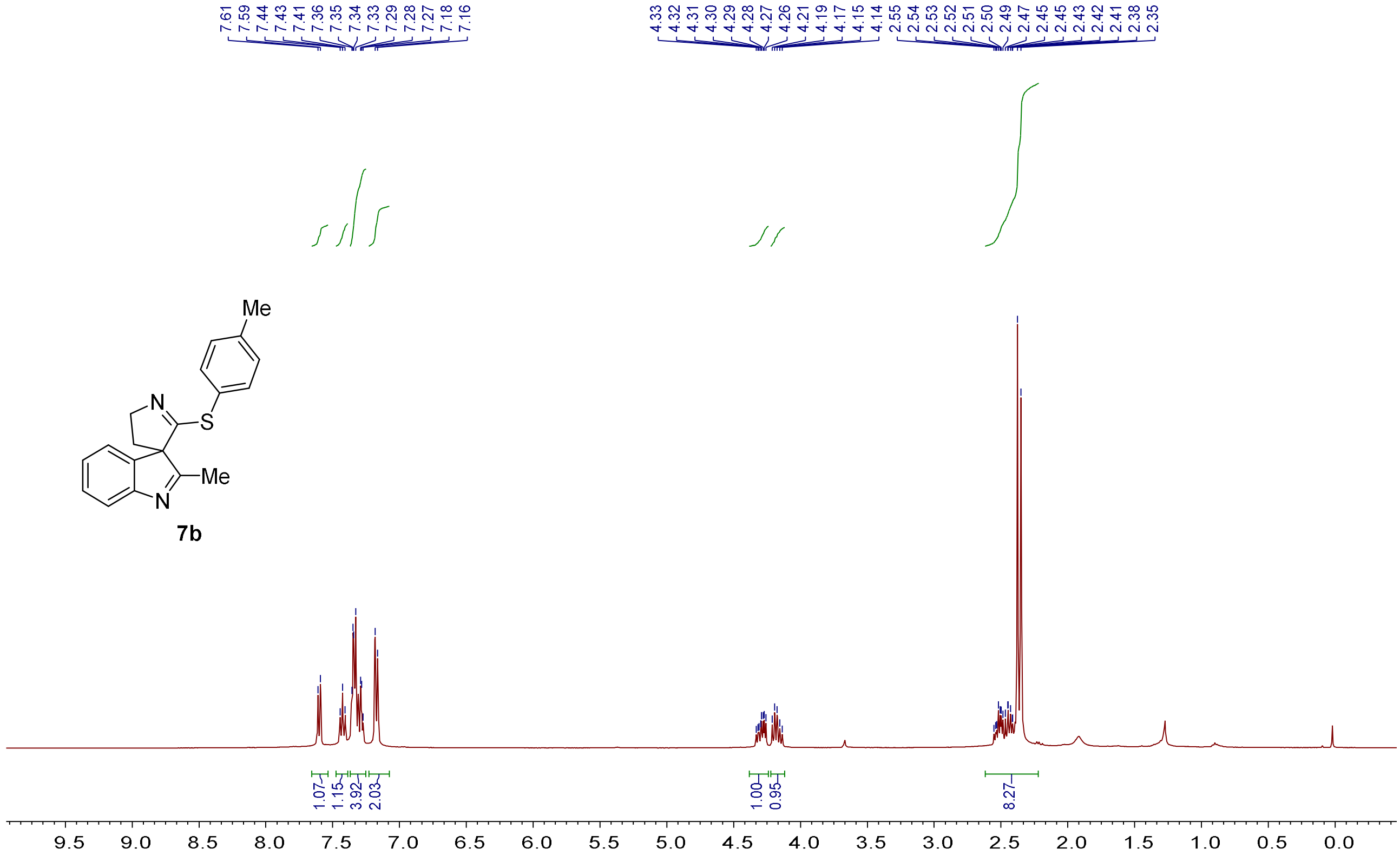

7.61 7.59 7.44 7.43 7.41 7.36 7.35 7.34 7.33 7.29 7.28 7.27 7.18 7.16
4.33 4.32 4.31 4.30 4.29 4.28 4.27 4.26 4.21 4.19 4.17 4.15 4.14
2.55 2.54 2.53 2.52 2.51 2.50 2.49 2.47 2.45 2.45 2.43 2.42 2.41 2.38 2.35
Me
N
S
Me
N
1.07 1.15 3.92 2.03
1.00 0.95
8.27
9.5 9.0 8.5 8.0 7.5 7.0 6.5 6.0 5.5 5.0 4.5 4.0 3.5 3.0 2.5 2.0 1.5 1.0 0.5 0.0

7b

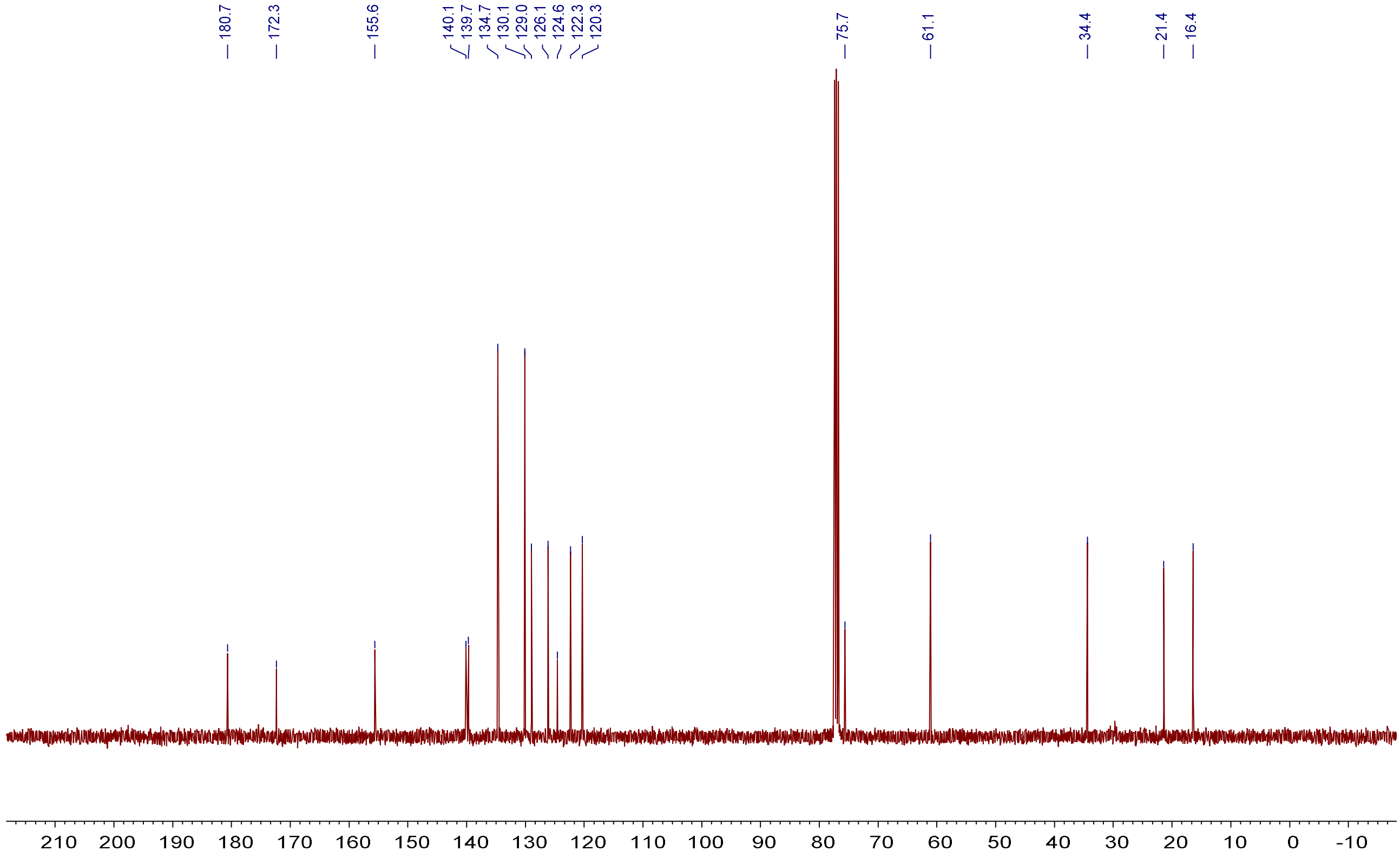

180.7
172.3
155.6
140.1 139.7 134.7 130.1 129.0 126.1 124.6 122.3 120.3
75.7
61.1
34.4
21.4 16.4
210 200 190 180 170 160 150 140 130 120 110 100 90 80 70 60 50 40 30 20 10 0 -10

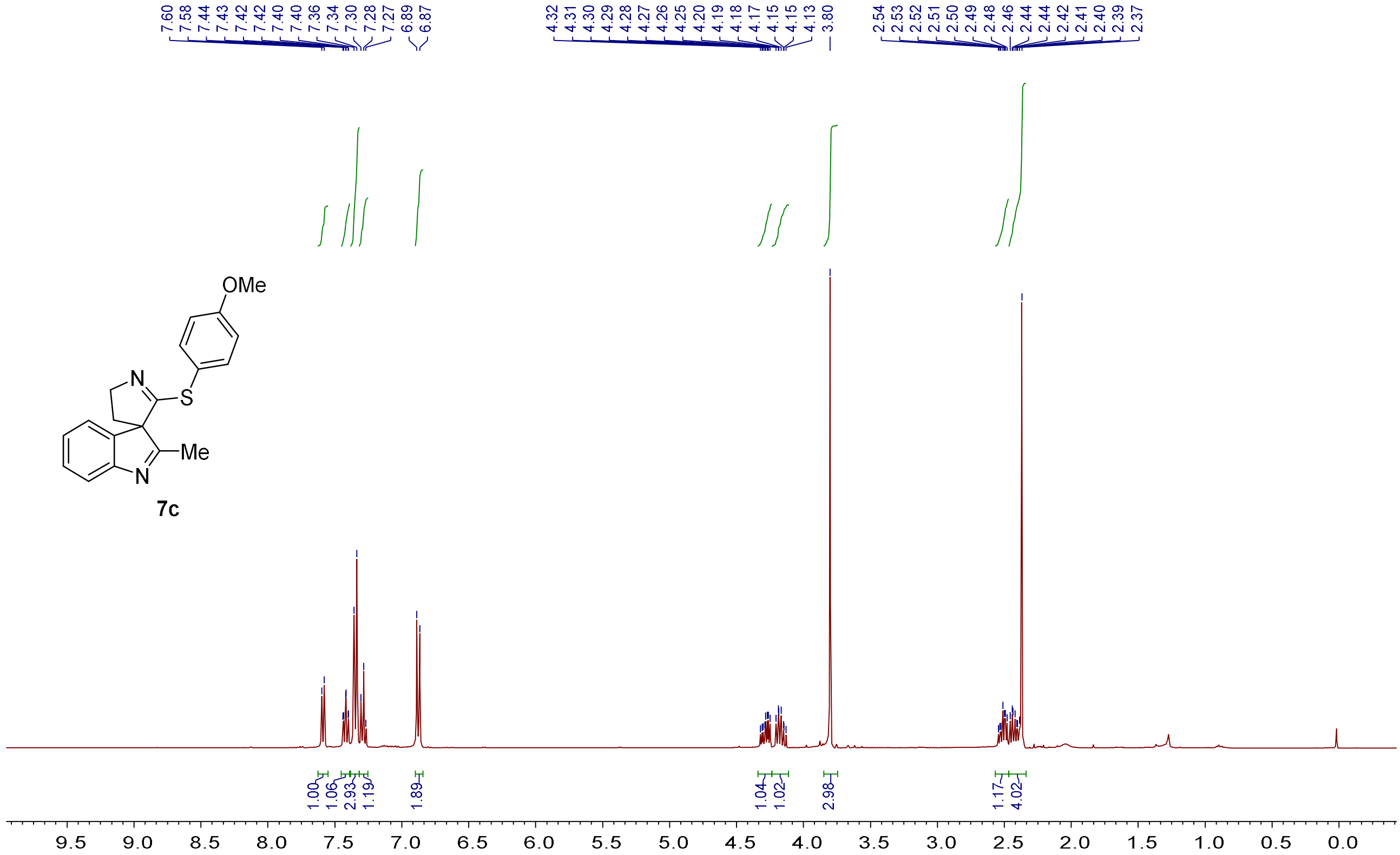

OMe
N
S
Me
N
7c
7.60 7.58 7.44 7.43 7.42 7.42 7.40 7.40 7.36 7.34 7.30 7.28 7.27 6.89 6.87
4.32 4.31 4.30 4.29 4.28 4.27 4.26 4.25 4.20 4.19 4.18 4.17 4.15 4.15 4.13 3.80
2.54 2.53 2.52 2.51 2.50 2.49 2.48 2.46 2.44 2.44 2.42 2.41 2.40 2.39 2.37
1.00 1.06 2.93 1.19 1.89 1.04 1.02 2.98 1.17 4.02
9.5 9.0 8.5 8.0 7.5 7.0 6.5 6.0 5.5 5.0 4.5 4.0 3.5 3.0 2.5 2.0 1.5 1.0 0.5 0.0

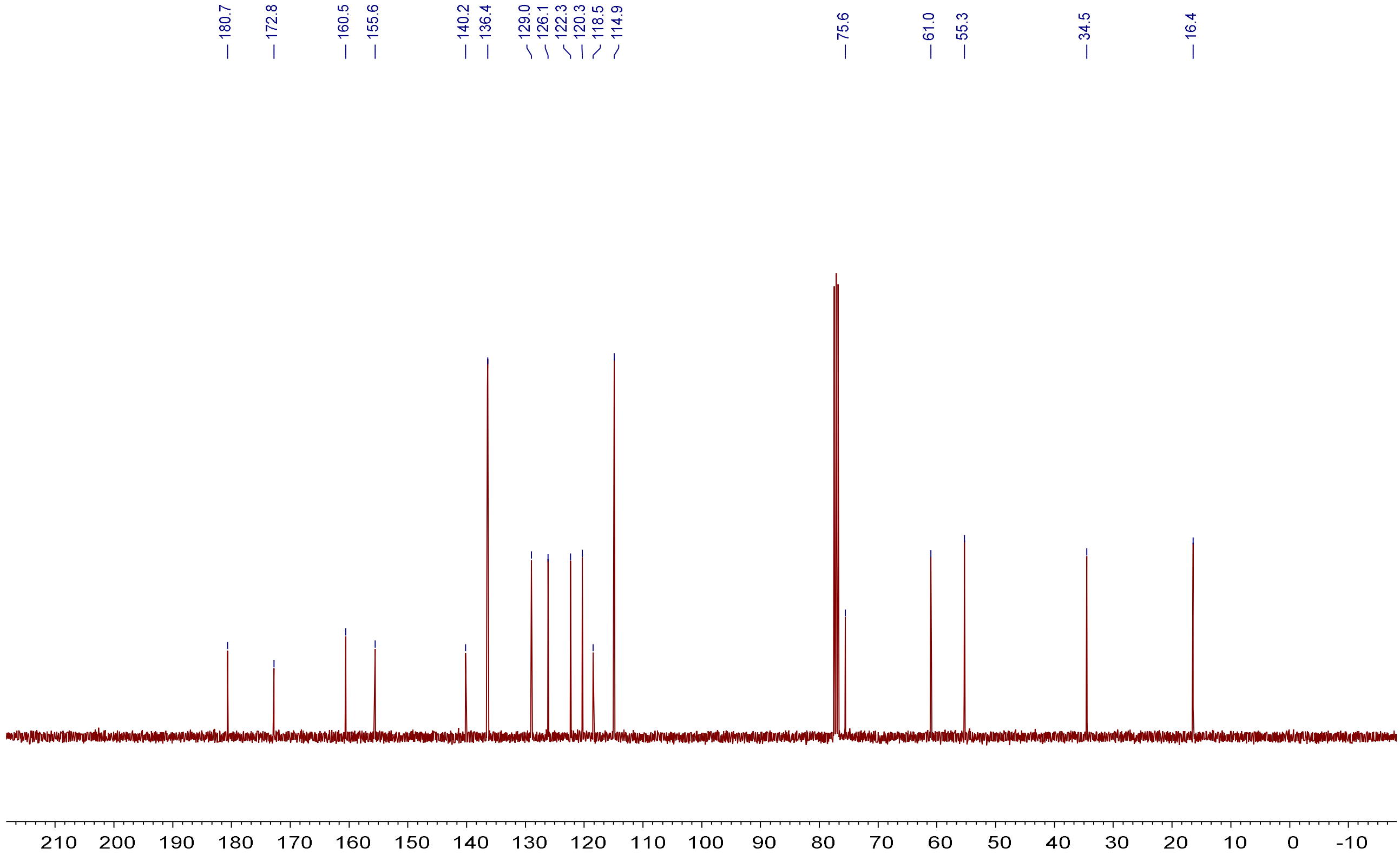

180.7 172.8 160.5 155.6 140.2 136.4 129.0 126.1 122.3 120.3 118.5 114.9 75.6 61.0 55.3 34.5 16.4
210 200 190 180 170 160 150 140 130 120 110 100 90 80 70 60 50 40 30 20 10 0 -10

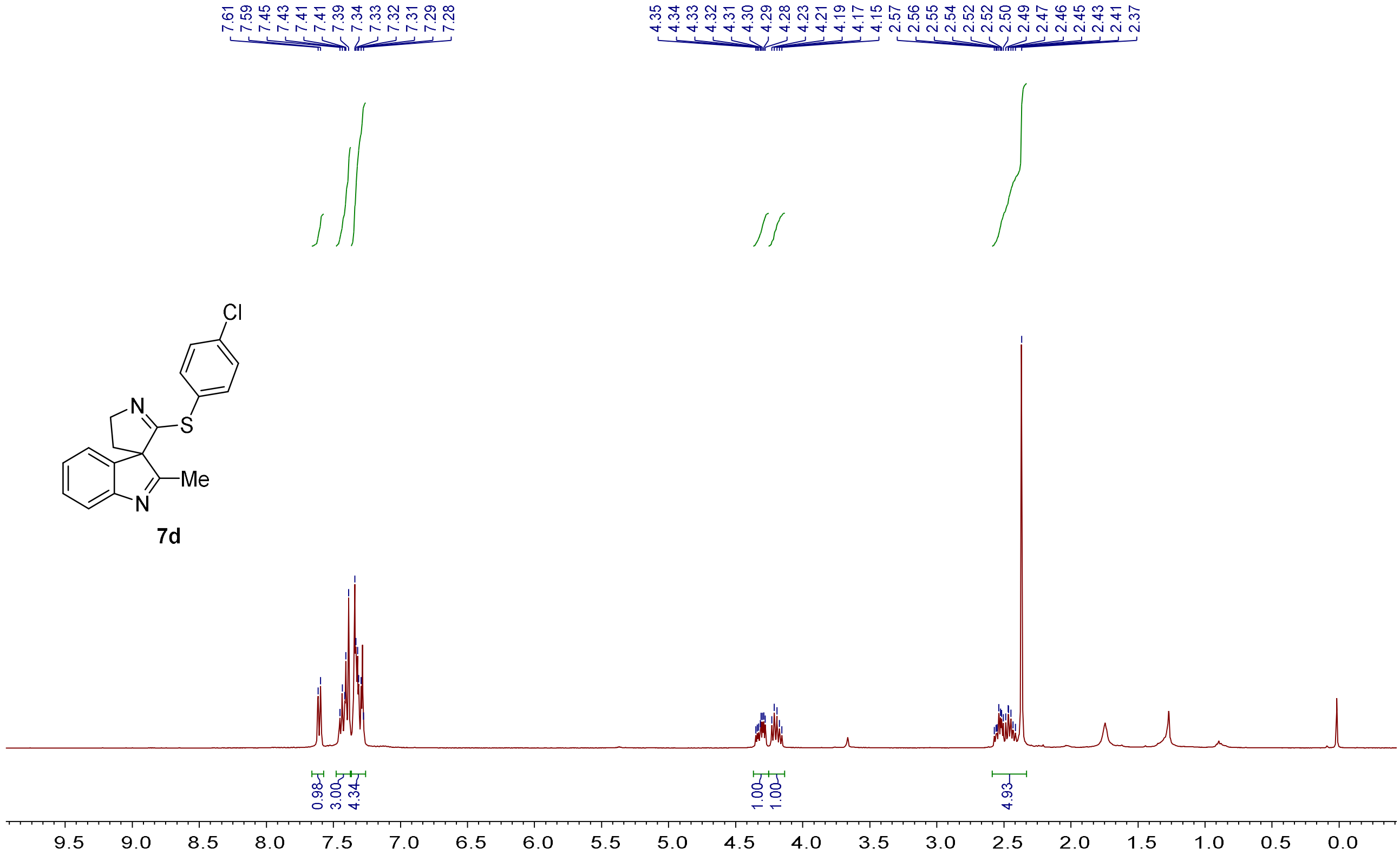
7.61 7.59 7.45 7.43 7.41 7.41 7.39 7.34 7.33 7.32 7.31 7.29 7.28
4.35 4.34 4.33 4.32 4.31 4.30 4.29 4.28 4.23 4.21 4.19 4.17 4.15
2.57 2.56 2.55 2.54 2.52 2.52 2.50 2.49 2.47 2.46 2.45 2.43 2.41 2.37
Cl
N
S
Me
N
7d
0.98 3.00 4.34 1.00 1.00 4.93
9.5 9.0 8.5 8.0 7.5 7.0 6.5 6.0 5.5 5.0 4.5 4.0 3.5 3.0 2.5 2.0 1.5 1.0 0.5 0.0

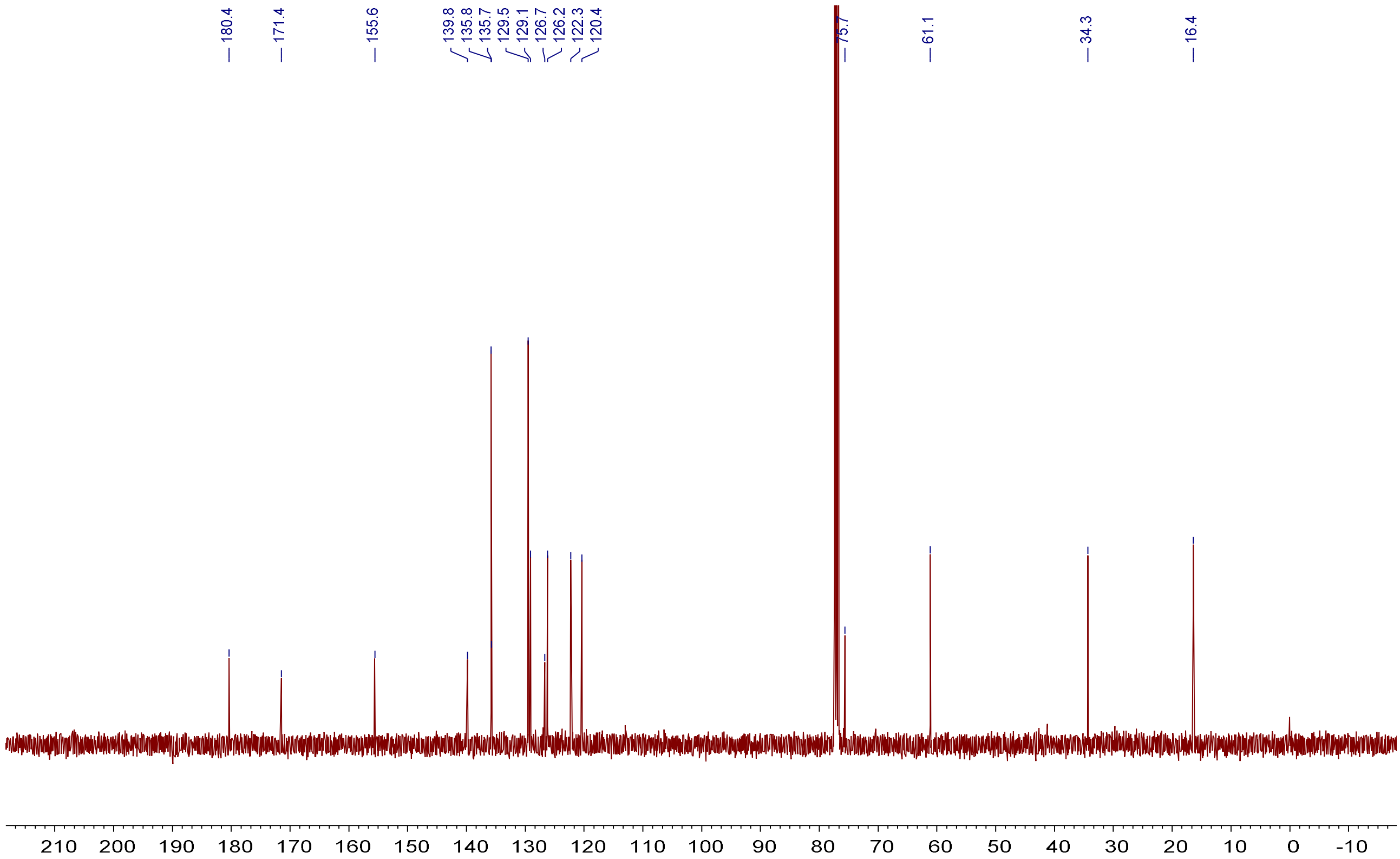
180.4 171.4 155.6 139.8 135.8 135.7 129.5 129.1 126.7 126.2 122.3 120.4 75.7 61.1 34.3 16.4
210 200 190 180 170 160 150 140 130 120 110 100 90 80 70 60 50 40 30 20 10 0 -10

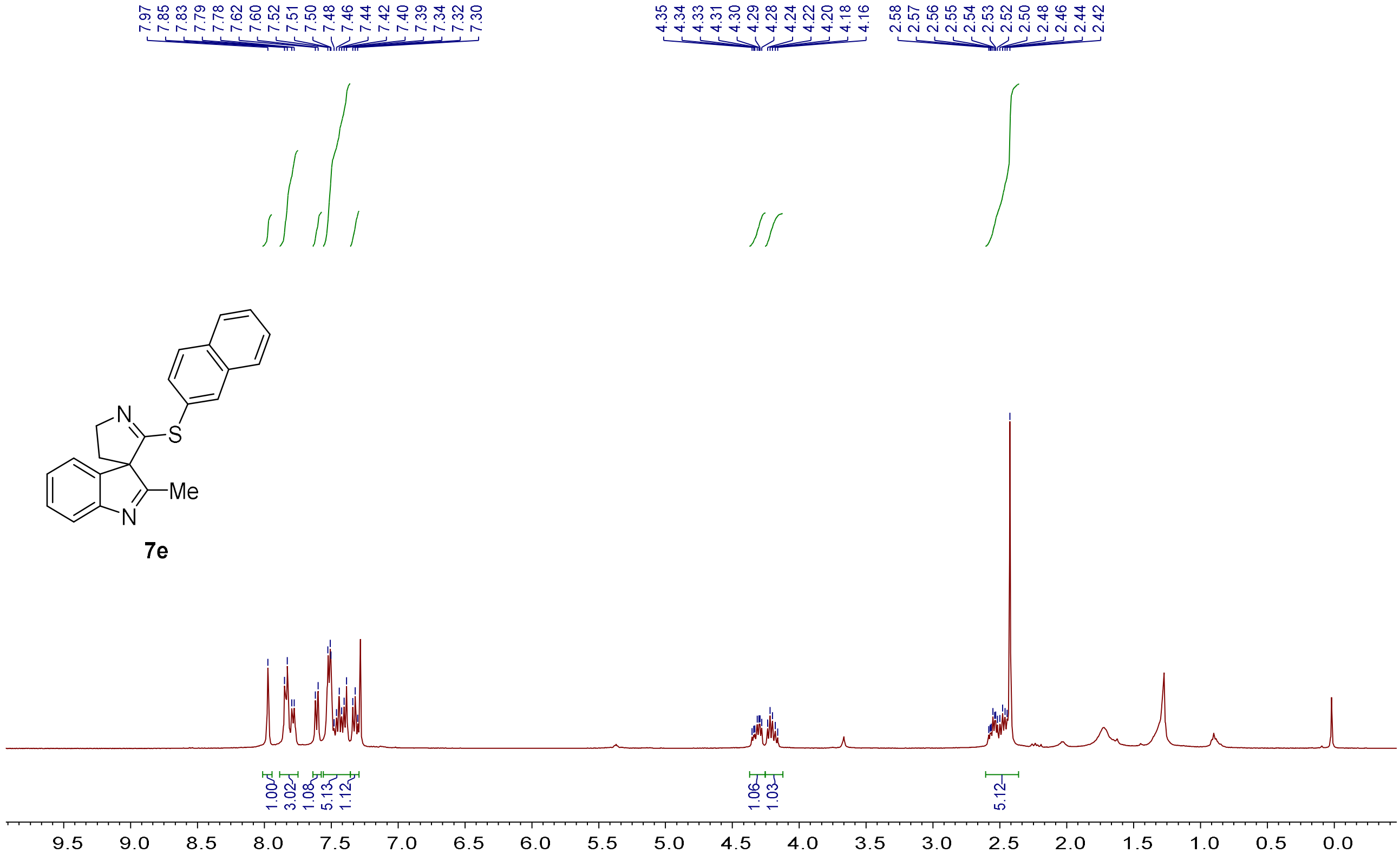
7.97
7.85
7.83
7.79
7.78
7.62
7.60
7.52
7.51
7.50
7.48
7.46
7.44
7.42
7.40
7.39
7.34
7.32
7.30
4.35
4.34
4.33
4.31
4.30
4.29
4.28
4.24
4.22
4.20
4.18
4.16
2.58
2.57
2.56
2.55
2.54
2.53
2.52
2.50
2.48
2.46
2.44
2.42
N
S
Me
N
7e
1.00
3.02
1.08
5.13
1.12
1.06
1.03
5.12
9.5 9.0 8.5 8.0 7.5 7.0 6.5 6.0 5.5 5.0 4.5 4.0 3.5 3.0 2.5 2.0 1.5 1.0 0.5 0.0

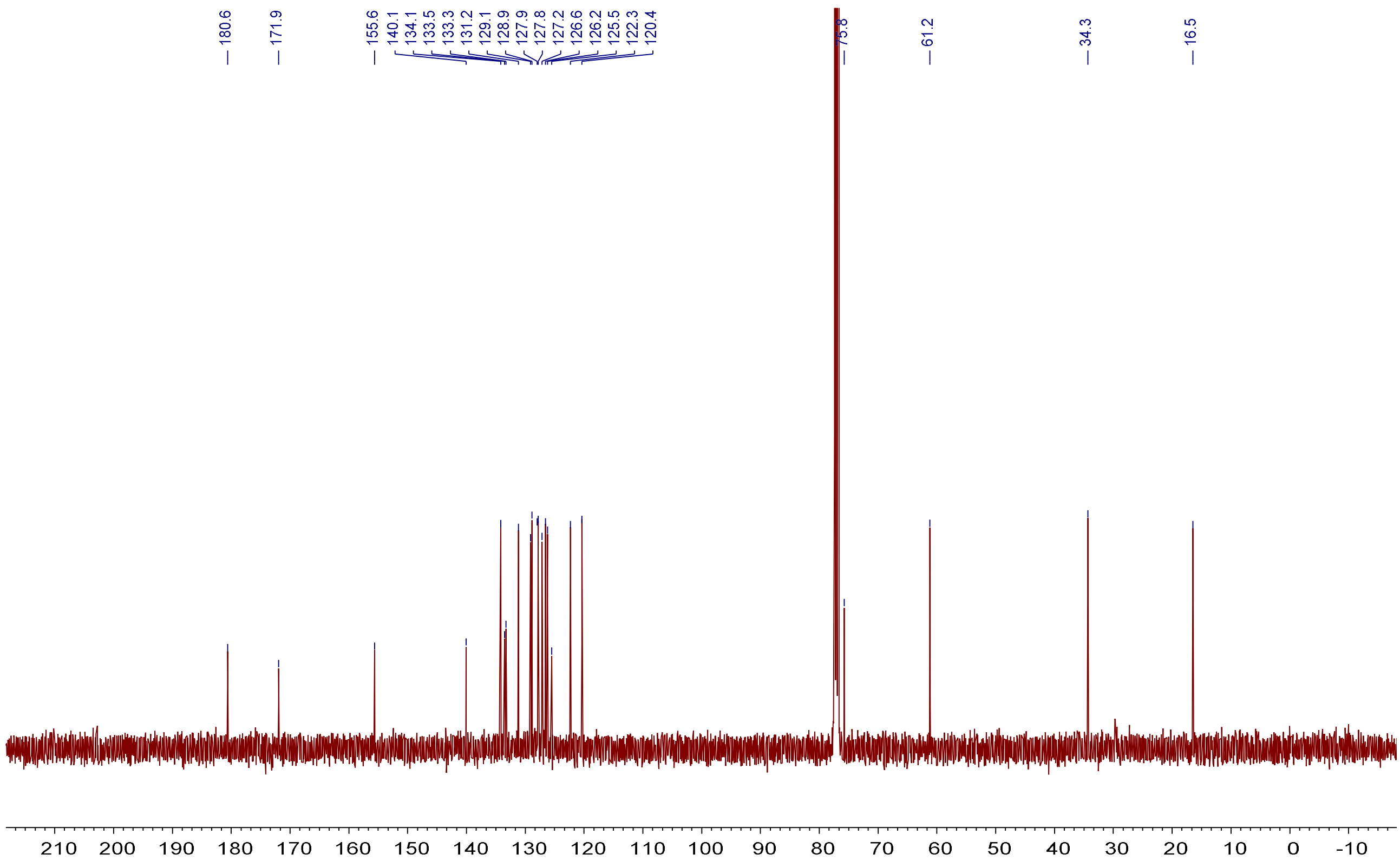
180.6
171.9
155.6
140.1
134.1
133.5
133.3
131.2
129.1
128.9
127.9
127.8
127.2
126.6
126.2
125.5
122.3
120.4
75.8
61.2
34.3
16.5
210 200 190 180 170 160 150 140 130 120 110 100 90 80 70 60 50 40 30 20 10 0 -10

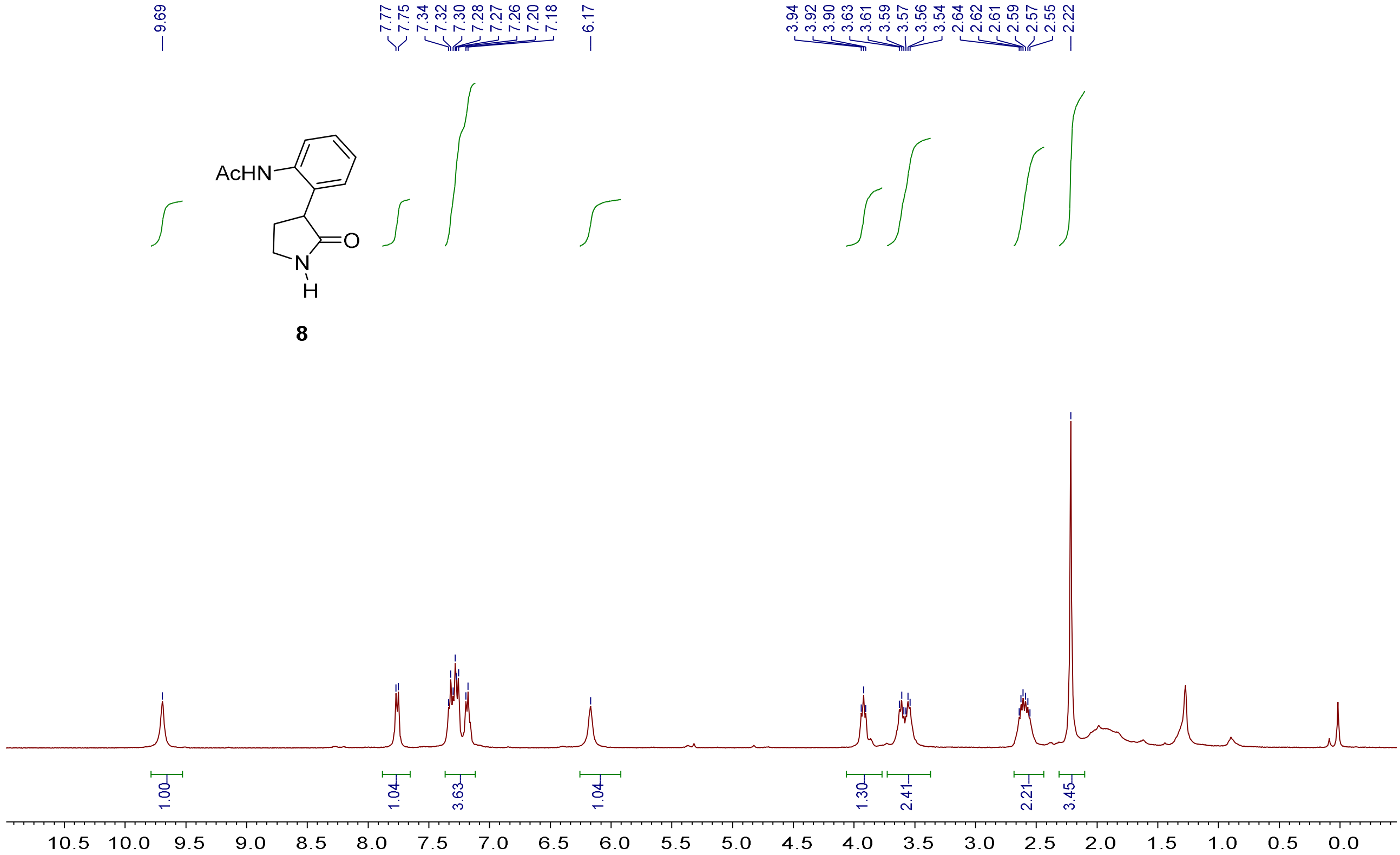

9.69
7.77
7.75
7.34
7.32
7.30
7.28
7.27
7.26
7.20
7.18
6.17
3.94
3.92
3.90
3.63
3.61
3.59
3.57
3.56
3.54
2.64
2.62
2.61
2.59
2.57
2.55
2.22
AcHN
O
N
H
8
1.00
1.04
3.63
1.04
1.30
2.41
2.21
3.45
10.5 10.0 9.5 9.0 8.5 8.0 7.5 7.0 6.5 6.0 5.5 5.0 4.5 4.0 3.5 3.0 2.5 2.0 1.5 1.0 0.5 0.0

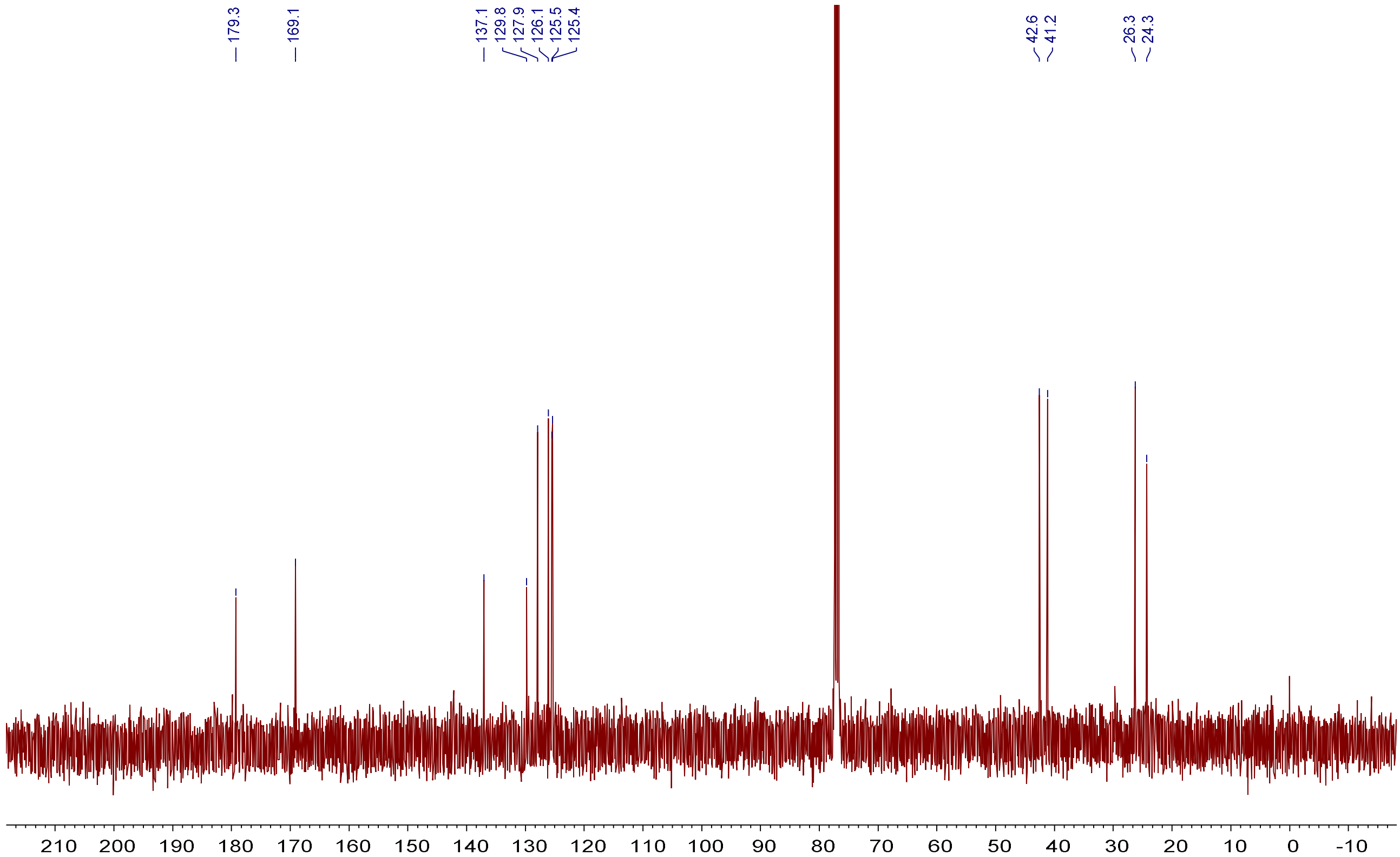

179.3
169.1
137.1
129.8
127.9
126.1
125.5
125.4
42.6
41.2
26.3
24.3
210 200 190 180 170 160 150 140 130 120 110 100 90 80 70 60 50 40 30 20 10 0 -10

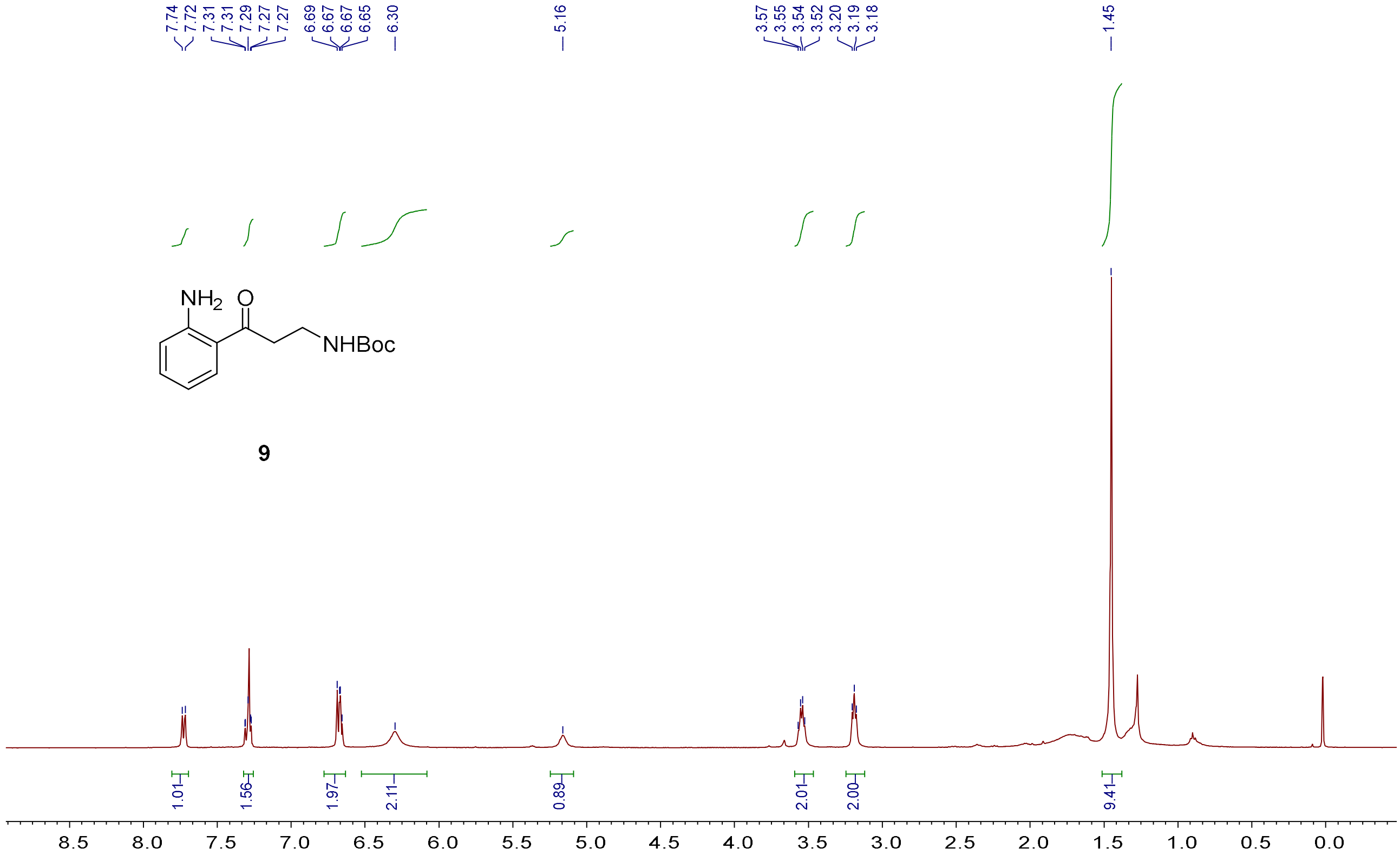
7.74
7.72
7.31
7.31
7.29
7.27
7.27
6.69
6.67
6.67
6.65
6.30
5.16
3.57
3.55
3.54
3.52
3.20
3.19
3.18
1.45
NH2
O
NHBoc
1.01
1.56
1.97
2.11
0.89
2.01
2.00
9.41
8.5
8.0
7.5
7.0
6.5
6.0
5.5
5.0
4.5
4.0
3.5
3.0
2.5
2.0
1.5
1.0
0.5
0.0

9

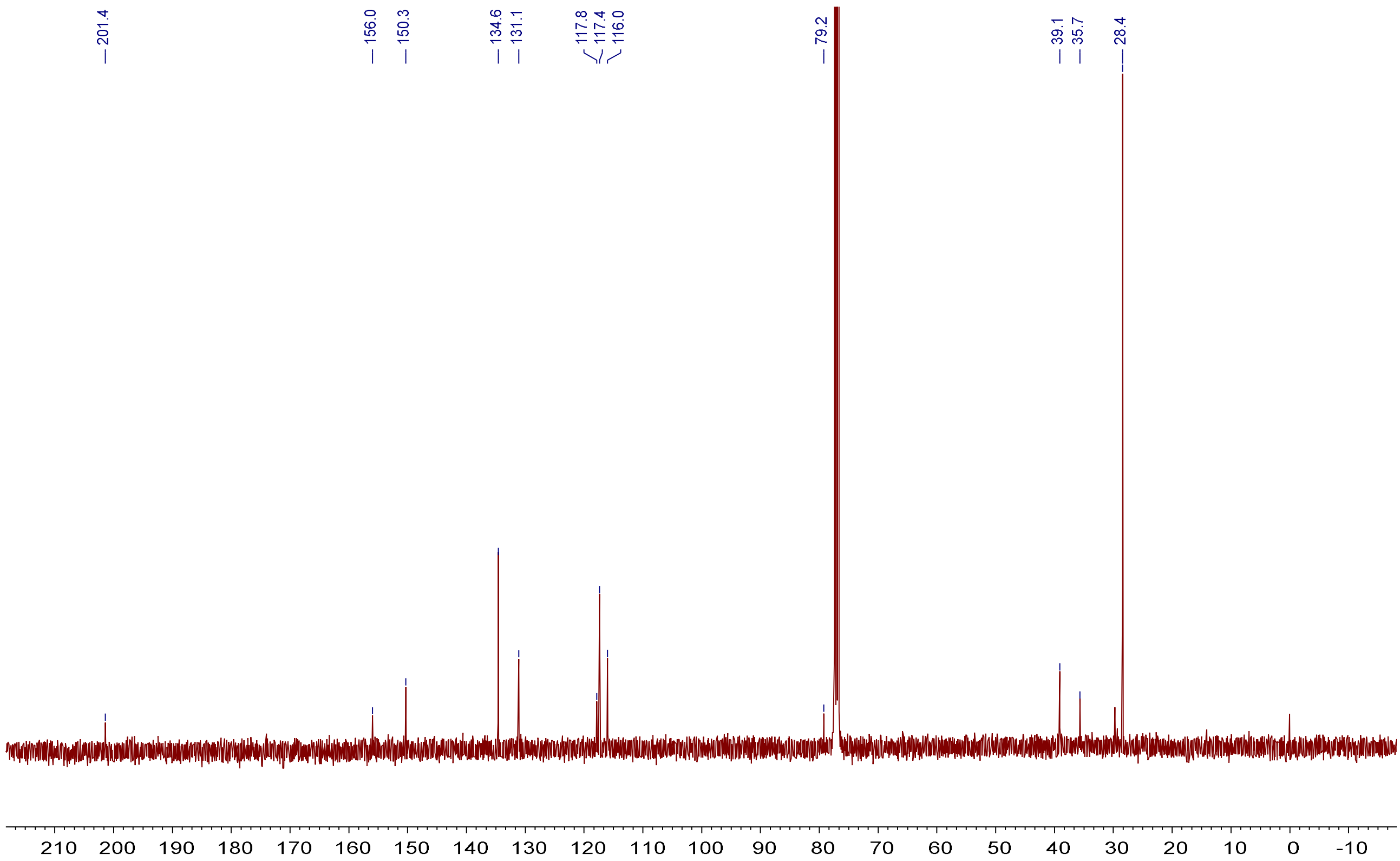
201.4
156.0
150.3
134.6
131.1
117.8
117.4
116.0
79.2
39.1
35.7
28.4
210
200
190
180
170
160
150
140
130
120
110
100
90
80
70
60
50
40
30
20
10
0
-10

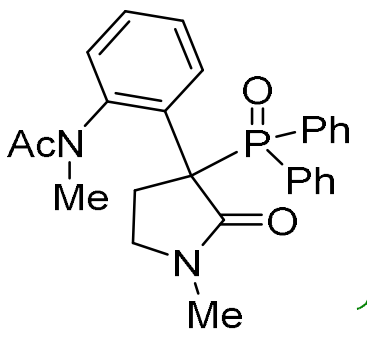
AcN
Me
O
P
Ph
Ph
O
N
Me

10

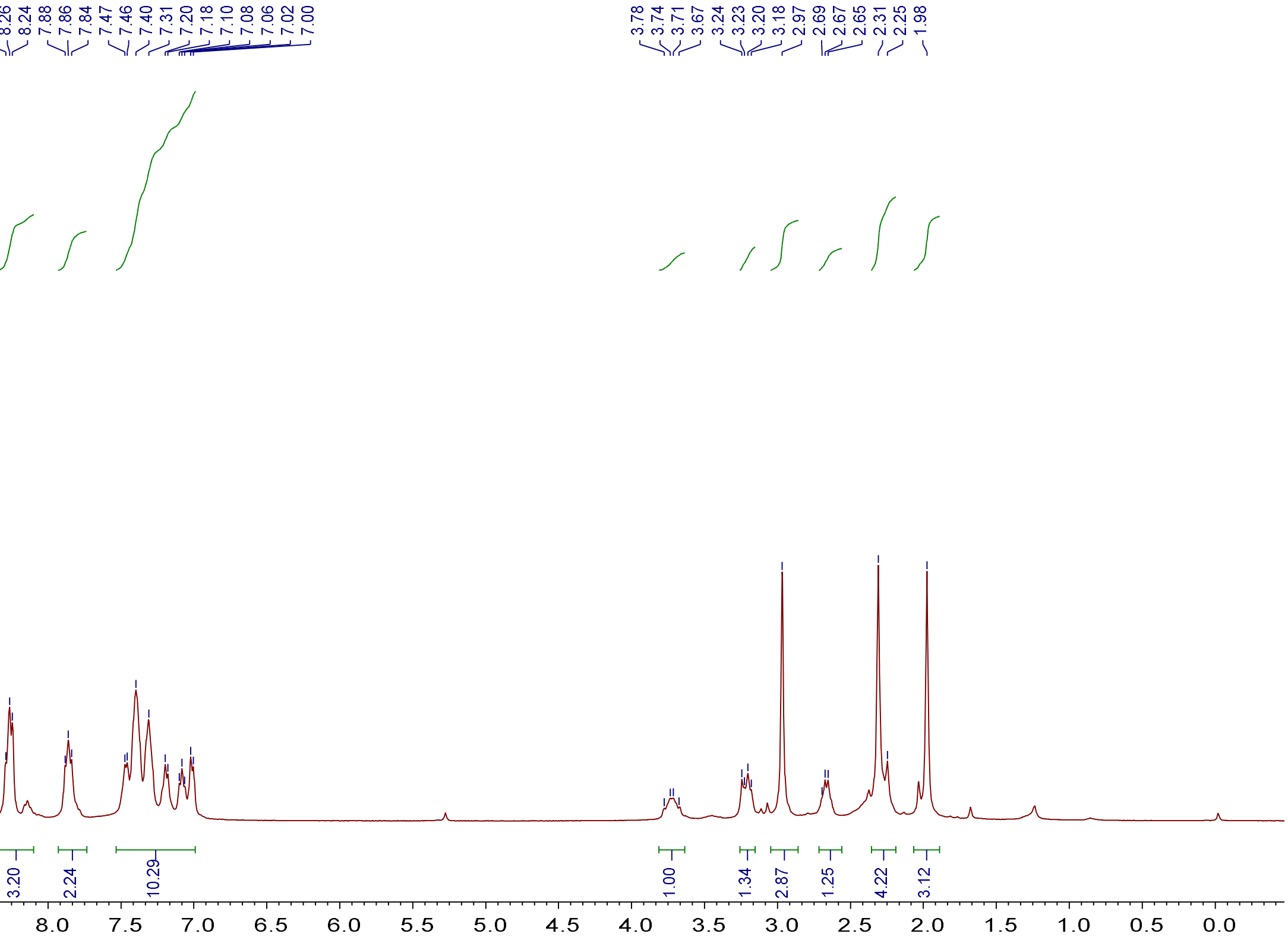
8.29 8.26 8.24 7.88 7.86 7.84 7.47 7.46 7.40 7.31 7.20 7.18 7.10 7.08 7.06 7.02 7.00
3.78 3.74 3.71 3.67 3.24 3.23 3.20 3.18 2.97 2.69 2.67 2.65 2.31 2.25 1.98
3.20
2.24
10.29
1.00
1.34
2.87
1.25
4.22
3.12
9.5 9.0 8.5 8.0 7.5 7.0 6.5 6.0 5.5 5.0 4.5 4.0 3.5 3.0 2.5 2.0 1.5 1.0 0.5 0.0

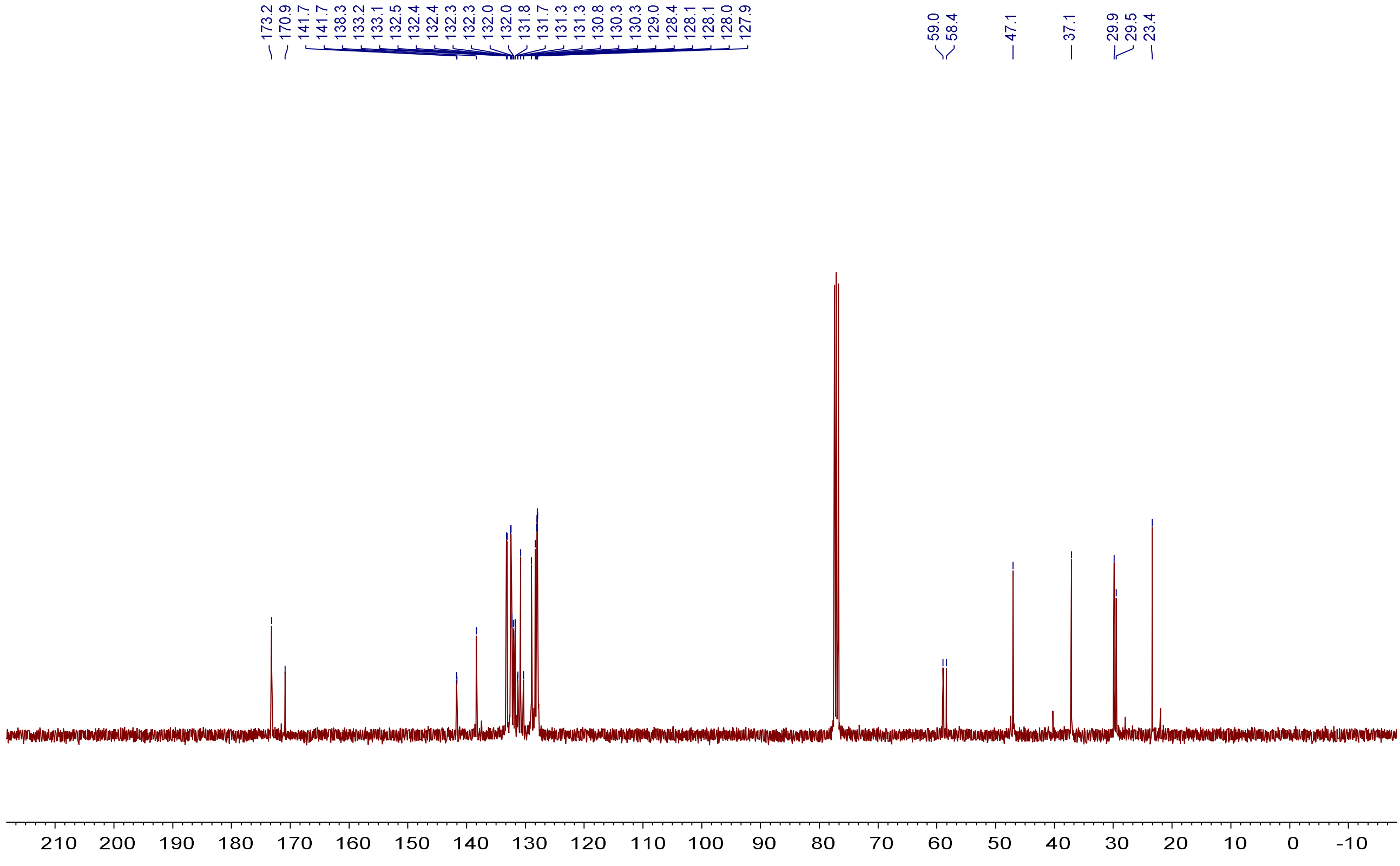
173.2 170.9 141.7 141.7 138.3 133.2 133.1 132.5 132.4 132.4 132.3 132.3 132.0 132.0 131.8 131.7 131.3 131.3 130.8 130.3 130.3 129.0 128.4 128.1 128.1 128.0 127.9
59.0 58.4
47.1
37.1
29.9 29.5 23.4
210 200 190 180 170 160 150 140 130 120 110 100 90 80 70 60 50 40 30 20 10 0 -10

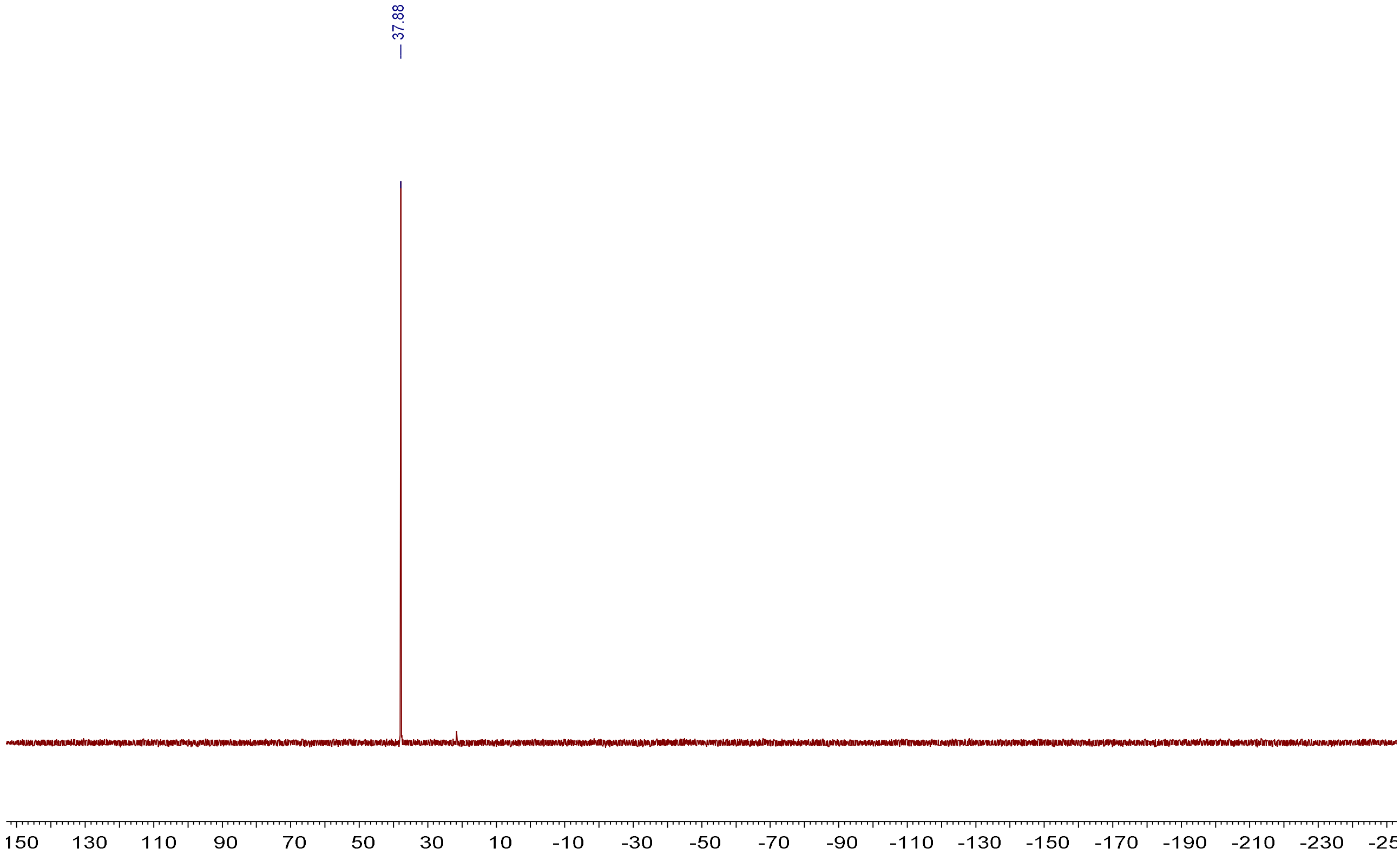
37.88
150
130
110
90
70
50
30
10
-10
-30
-50
-70
-90
-110
-130
-150
-170
-190
-210
-230
-25

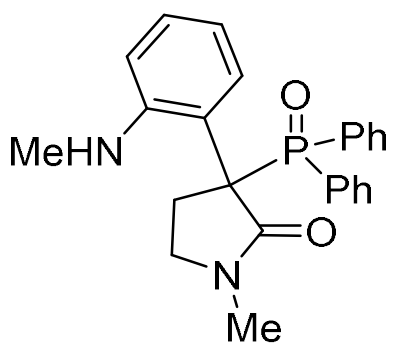

11

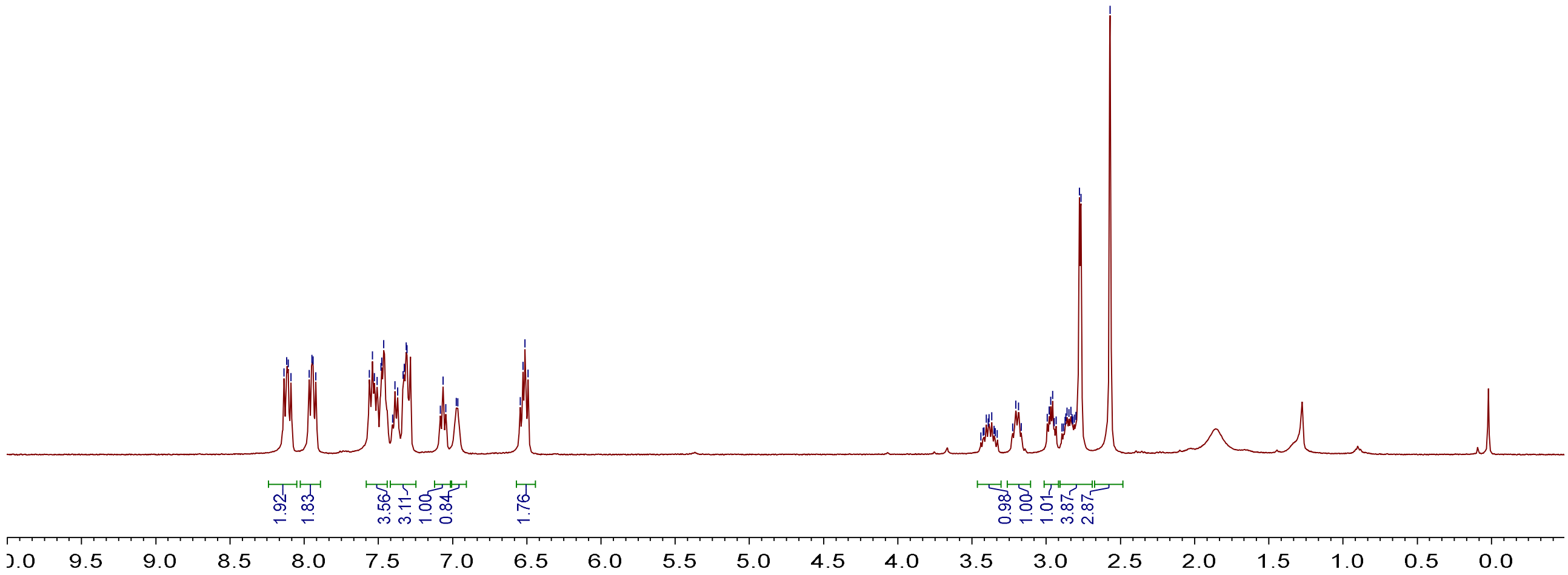

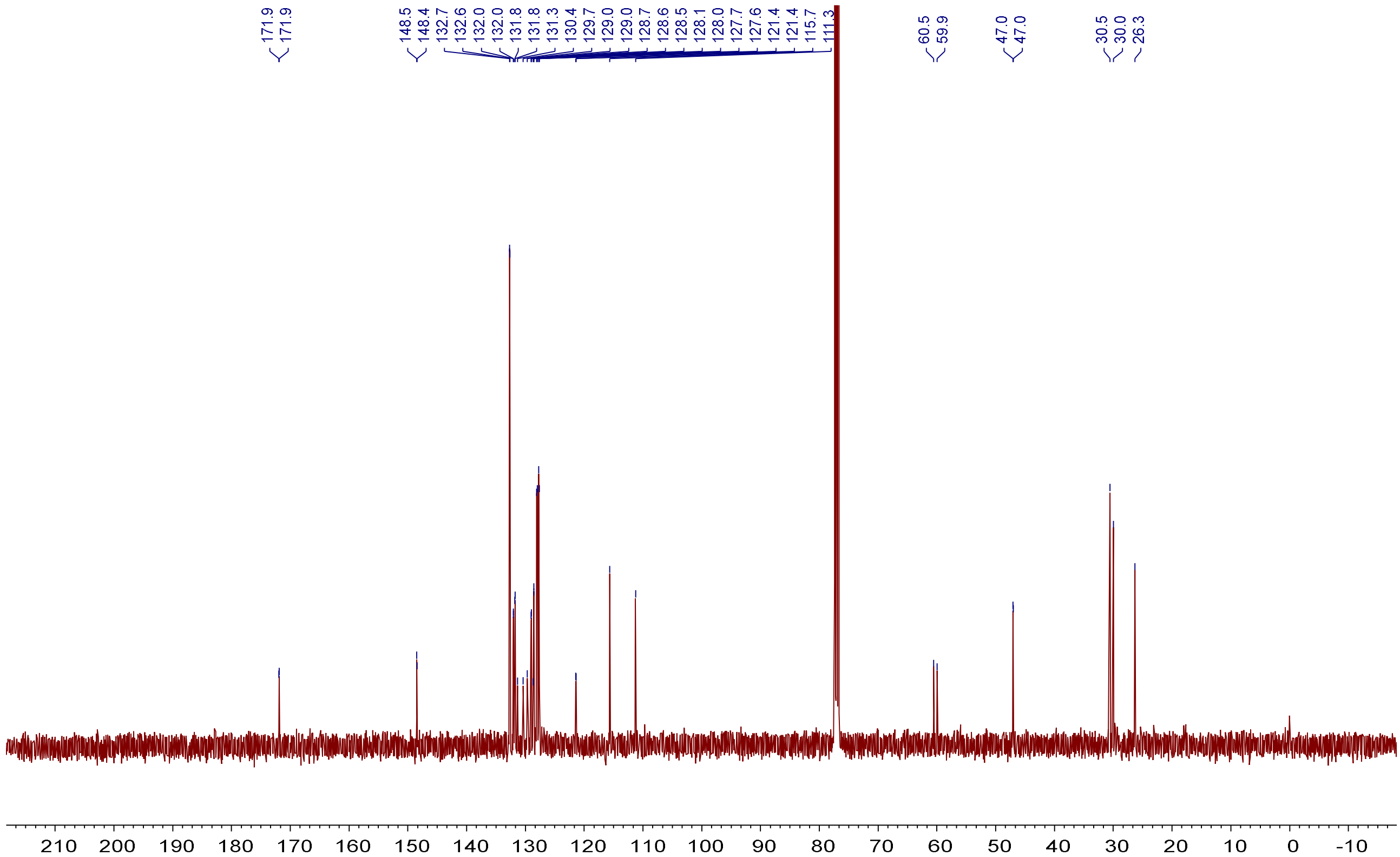

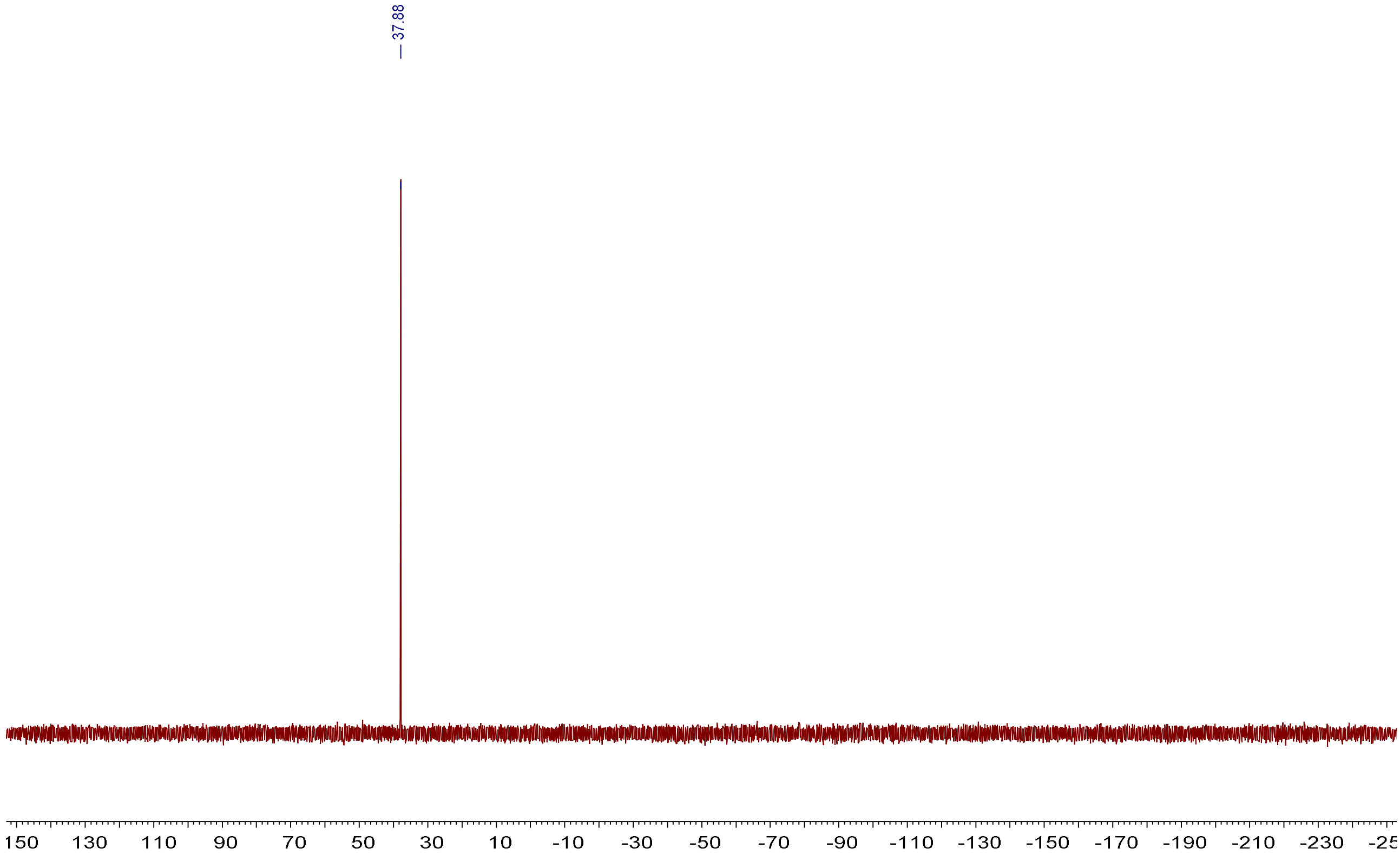

37.88
150 130 110 90 70 50 30 10 -10 -30 -50 -70 -90 -110 -130 -150 -170 -190 -210 -230 -25

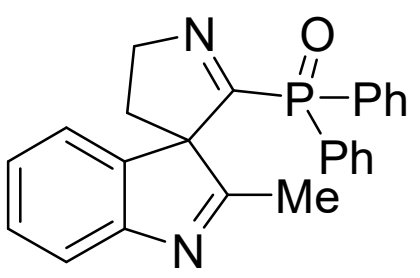
N
O
P
Ph
Ph
Me
N

12

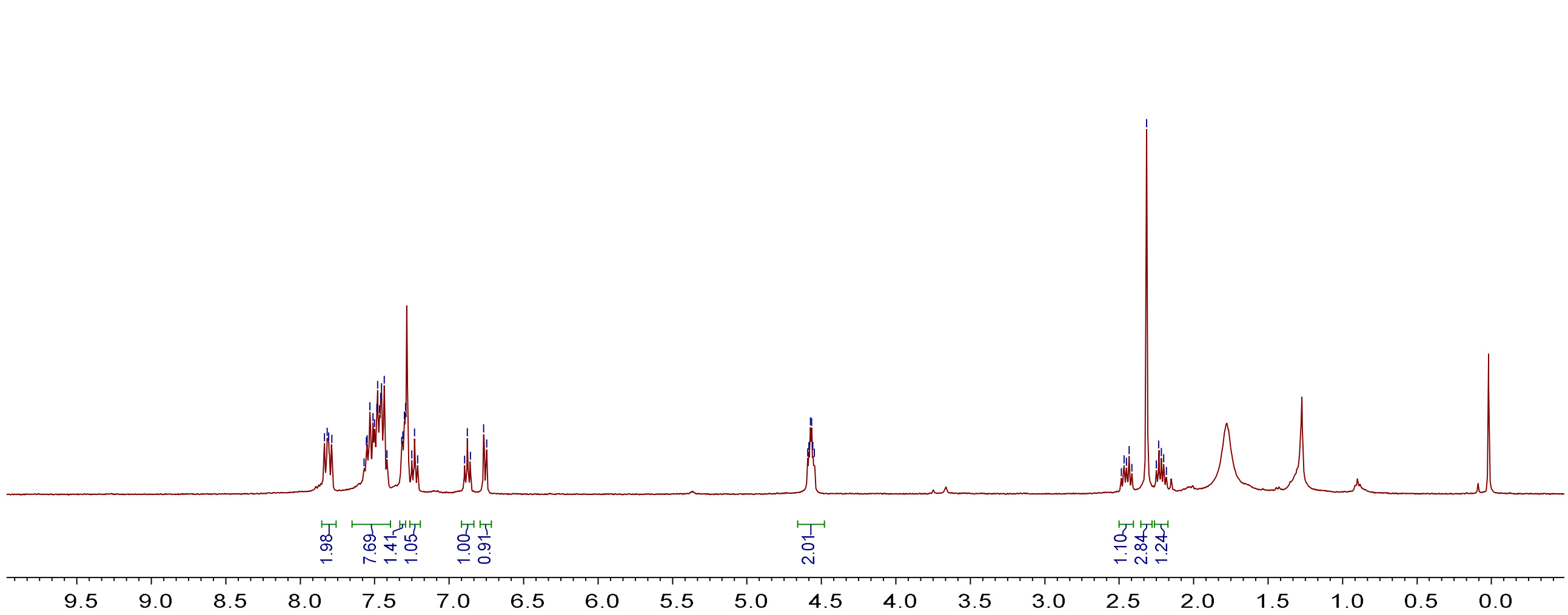
7.84 7.82 7.81 7.79 7.55 7.55 7.53 7.51 7.50 7.49 7.48 7.47 7.46 7.45 7.44 7.42 7.32 7.31 7.30 7.29 7.23 6.90 6.88 6.86 6.77 6.75
4.59 4.58 4.57 4.57 4.56 4.55
2.49 2.47 2.45 2.43 2.41 2.32 2.25 2.23 2.22 2.20 2.18
1.98 7.69 1.41 1.05 1.00 0.91 2.01 1.10 2.84 1.24
9.5 9.0 8.5 8.0 7.5 7.0 6.5 6.0 5.5 5.0 4.5 4.0 3.5 3.0 2.5 2.0 1.5 1.0 0.5 0.0

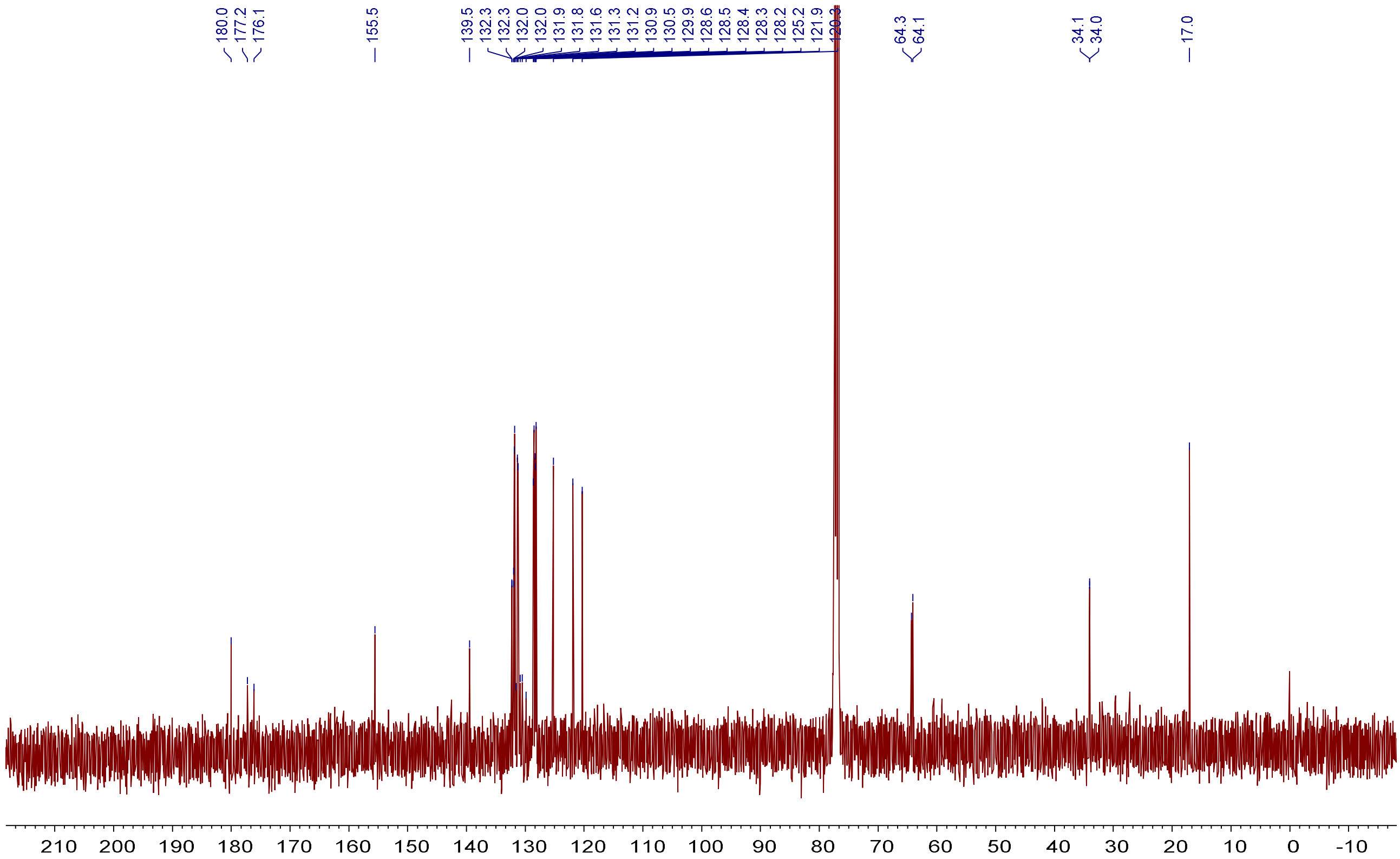
180.0 177.2 176.1
155.5
139.5 132.3 132.3 132.0 132.0 131.9 131.8 131.6 131.3 131.2 130.9 130.5 129.9 128.6 128.5 128.4 128.3 128.2 125.2 121.9
64.3 64.1
34.1 34.0
17.0
210 200 190 180 170 160 150 140 130 120 110 100 90 80 70 60 50 40 30 20 10 0 -10

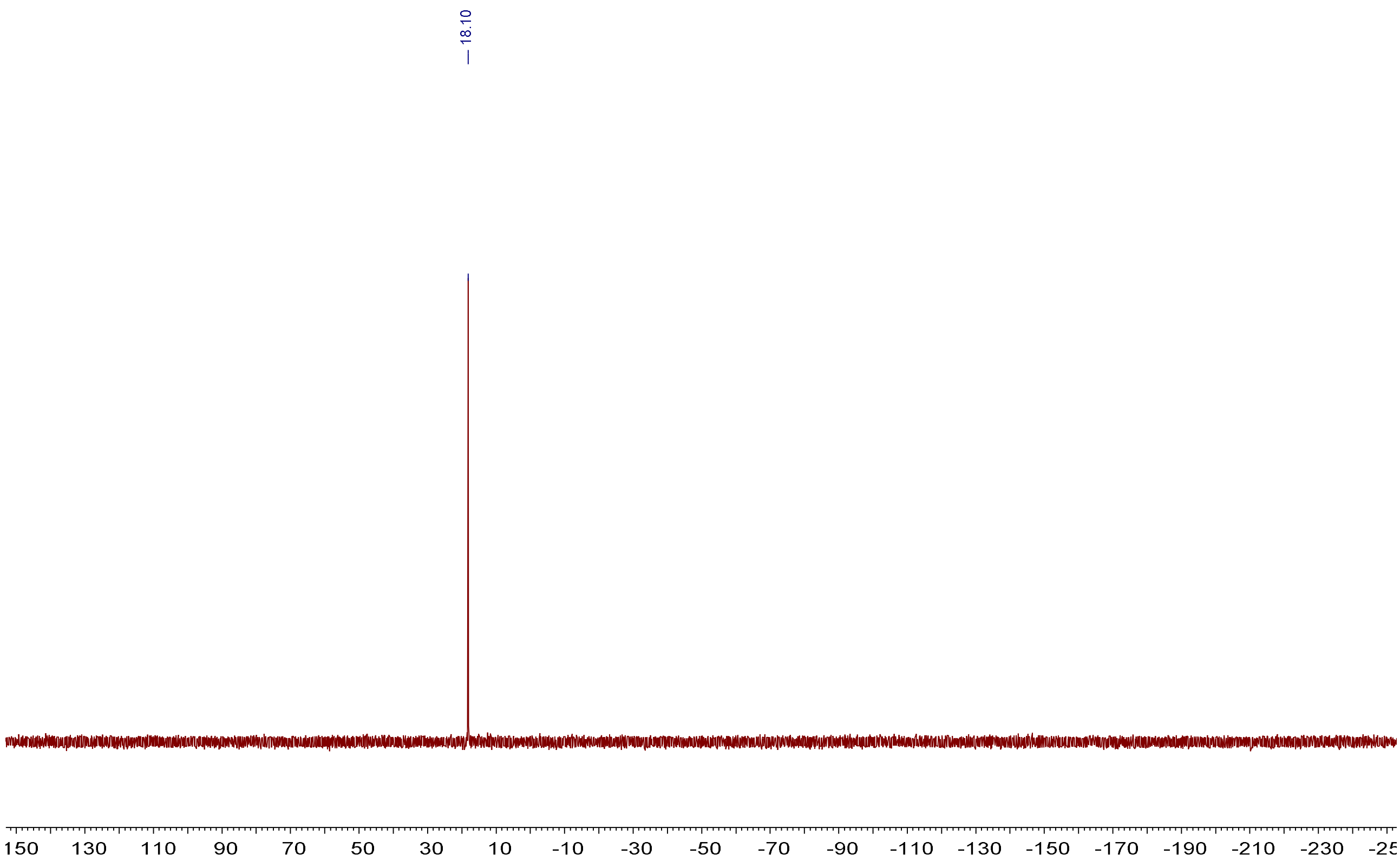

Supplementary Fig. 32 NMR spectra.

7. Supplementary references

1. Auras, F. et. al. Synchronized offset stacking: a concept for growing large-domain and highly crystalline 2D covalent organic frameworks. *J. Am. Chem. Soc.* **138**, 16703–16710 (2016).

2. Prier, C. K., Rankic, D. A. & MacMillan, D. W. C. Visible light photoredox catalysis with transition metal complexes: applications in organic synthesis. *Chem. Rev.* **113**, 5322–5363 (2013).

3. Luo, J. & Zhang, J. Donor–acceptor fuorophores for visible-light-promoted organic synthesis: photoredox/Ni dual catalytic C(sp3)–C(sp2) cross-coupling. *ACS Catal.* **6**, 873–877 (2016).

4. Gu, X., Mo, X., Bai, W. -J., Xie, P., Hu, W. & Jiang, J. Catalytic asymmetric P-H insertion reactions. *J. Am. Chem. Soc.* **145**, 20031–20040 (2023).
